# Photocatalytic Cross‐Coupling of Phenols and Heteroaryl Halides With Machine Learning‐Guided Reaction Prediction

**DOI:** 10.1002/anie.8222003

**Published:** 2026-03-05

**Authors:** Matthew C. Carson, Alice Wu, Kalyana B. Duggal, Madeline E. Rotella, Marisa C. Kozlowski

**Affiliations:** ^1^ Department of Chemistry University of Pennsylvania Philadelphia Pennsylvania USA

**Keywords:** aryl radicals, machine learning, phenols, photochemistry

## Abstract

Developing sustainable methods for C(sp^2^)─C(sp^2^) bond formation that avoid transition‐metals and prefunctionalized substrates remains a central goal in synthetic chemistry. Phenols and *N*‐heteroarenes (azines) are abundantly available, yet their cross‐coupling is hindered by mismatched redox properties and chemoselectivity issues. Herein, we report a photochemical strategy that couples phenols with heteroaryl halides under redox‐neutral conditions using an organic dye photocatalyst and base. Concurrent oxidation of the phenol component and reduction of the azine component generates complementary radicals that cross‐couple efficiently, delivering moderate to high yields (up to 91%) with high functional group tolerance. Mechanistic experiments and density functional theory (DFT) studies elucidate the radical reaction pathways, while substrate clustering, high‐throughput experimentation (HTE), and machine learning (ML) enable prediction of C–C versus S_N_Ar reactivity across broad chemical space.

## Graphical abstract

**Photocatalytic cross‐coupling**: A redox‐neutral photochemical method enables direct C(sp^2^)─C(sp^2^) bond formation between phenols and heteroaryl halides using an organic dye and base. Complementary radical generation allows efficient cross‐coupling in up to 91% yield. Mechanistic studies, DFT, HTE, and machine learning rationalize and predict reactivity, offering a sustainable approach to this challenging transformation.

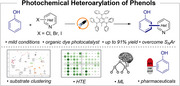

## 1 Introduction

Phenols and nitrogen‐containing heterocycles are privileged motifs in medicinal chemistry, offering hydrogen‐bonding, redox activity, and the ability for innate C(sp^2^)─H functionalization [1, 2]. Native enzymes are known to metabolically oxidize phenol functional groups, but these reactive moieties are increasingly seen in drugs. Remarkably, 62% of the small molecule FDA‐approved drugs in 2020 contain a phenol or phenol ether [3]. *N*‐Heterocycles, that is, pyridines, are persistently found across the pharmaceutical and agrochemical industries [2]. When the two scaffolds are conjoined via a biaryl bond, even more promising therapeutic and material science properties can be realized [4, 5]. Phenol‐derived pyridines that can chelate also serve as useful ligands for the activation of boron‐derived reagents [6].

**FIGURE 1:**
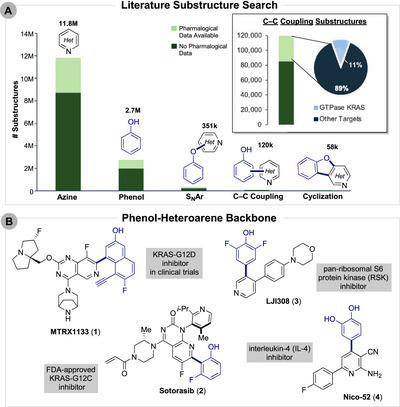
(A) Literature substructure search for phenol‐heteroarene backbone. (B) Examples of phenol‐heteroarene backbone in pharmaceuticals.

Owning to their prevalence, a chemical literature analysis of the commercial availability of phenols and six‐membered *N*‐heterocycles (azines) yielded millions of entries. From this search, around 12M azines were tabulated, while only 3M phenols were present (Figure 1A) (see Supporting Information for search specifics). Structures uniting these motifs (Figure 1A), most commonly do so via a C─O bond between the pyridine ring and phenolic oxygen (351k compounds). Other unions including C─C bonds (120k) or both C─O and C─C bonds (cyclization) (58k) are less common (Figure 1A). Notably, pharmacological data are reported for nearly a third of these C─C products indicating interest from the medicinal chemistry community.

**SCHEME 1:**
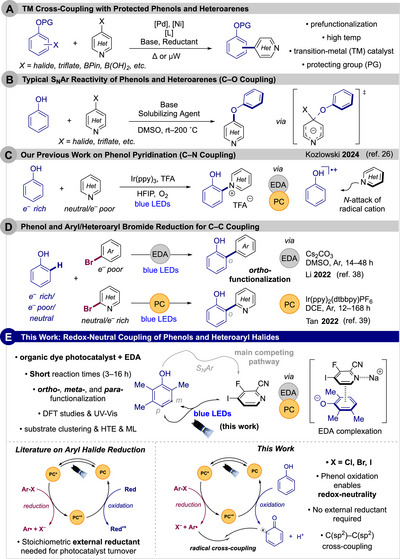
(A) Transition‐metal (TM) cross‐coupling of protected phenols and heteroarenes. (B) Conventional S_N_Ar reactivity of phenols and heteroarenes. (C) Phenol pyridination via dual‐photocatalytic manifold. (D) Phenol C─C cross‐coupling with electron‐poor aryl bromides and neutral heteroaryl bromides. (E) This work on redox‐neutral cross‐coupling of phenols and heteroaryl halides.

In particular, these C─C bonds are found in the GTPase KRAS inhibitors MRTX1133 (**1**) [7] and sotorasib (**2**) [8] (Figure 1B) and other oncology/immunology targets (PRT3789, [9] AZD4144 [10]). LJI308 (**3**) [11], a ribosomal S6 kinase (RSK) inhibitor with broad activity, contains a key phenol‐pyridine bond as does the first small molecule interleukin‐4 (IL‐4) inhibitor, nico‐52 (**4**) [12] (Figure 1B). These compounds are typically forged through metal catalyzed (Pd, Ni) Suzuki‐Miyaura coupling (Scheme 1A). As such, both starting materials must be furnished with tailored functional groups (halides, triflates, organoborons, etc.). Further, the phenols typically require protection to avoid undesired C─O coupling and interference with other reagents. For example, Amgen forged the biaryl bond of sotorasib (**2**) via a Suzuki‐Miyaura coupling with a carefully optimized cyclic boroxine partner that incorporated in situ protection of the phenol [8]. This approach required careful solvent control to avoid protodeboronation. Removal of residual metal impurities from such reactions can also be challenging [13].

Furthermore, the more common synthetic disconnection, the C─O linkage, is most often established via S_N_Ar (Scheme 1B), Ullman couplings with Cu [14]/Pd [15], or electron donor‐acceptor (EDA) complexes [16]. On the other hand, construction of the C─C bonds is much more challenging due to this competitive S_N_Ar. Innovative strategies have recently utilized functional group interconversion [17, 18] or aryne chemistry [19] to afford this union. However, these approaches utilize lengthy syntheses and unstable precursors.

In a more atom economical fashion, the low oxidation potentials of phenols have enabled a broad range of C(sp^2^)─H functionalization reactions that exploit their intrinsic radical reactivity [20]. The resultant phenoxyl radicals have found widespread application in the total synthesis of natural products [21, 22, 23, 24]. In 2020, our laboratory introduced photochemical oxidative phenol coupling as a new avenue to accomplish phenol oxidation selectively with low catalyst loading. Specifically, an acridinium dye‐based photocatalyst delivered efficient homo‐ and cross‐coupling [25].

Related investigations have also elucidated that phenol intermediates can engage in alternate reaction pathways. Under acidic conditions, phenol radical cation intermediates were found to be stabilized and could be intercepted by weakly nucleophilic pyridines (Scheme 1C) [26]. This C─N coupling was achieved under dual‐photocatalysis with concurrent photocatalytic and EDA cycles to afford phenol‐pyridinium salts. Subsequently, the use of catalytic pyridinium additives was extended to C(sp^2^)─H amination via in situ EDA activation [27] and S_N_Ar of halophenols [28].

Our focus then shifted to forming biaryl bonds between phenols and heteroarenes. A wealth of literature describes the generation of aryl radicals from aryl halides via photoredox [29], electrochemical, [30] thermal (CO_2_^•–^) [31], and EDA [32] methods. Halogen‐atom transfer (XAT) reagents derived from boron [33], amine [34], and silyl [35, 36] scaffolds have also been developed to access such radicals. Much of this prior photochemical work relies on stoichiometric external reductants, typically tertiary amines, to achieve catalyst turnover [37].

In 2022, redox‐neutral strategies were demonstrated by Li [38] and Tan [39], who reported the introduction of phenols in C─C cross‐coupling with electron‐poor aryl bromides and electron‐neutral heteroaryl bromides, respectively (Scheme 1D). With an EDA complex, Li and co‐workers disclosed a base‐mediated cross‐coupling of phenols and electron‐poor (CN, ketone, and CF_3_) aryl bromides to afford only *ortho*‐functionalization of phenols [38]. The work of Tan and co‐workers requires halogenated solvents such as 1,2‐dichloroethane (DCE), expensive iridium‐based photocatalysts, and long reaction times (up to 168 h) while only achieving *ortho*‐functionalization of phenols [39]. The scarcity of iridium highlights the need for more sustainable alternatives [40]. Although iridium complexes are potent photoreductants, their use often requires careful tailoring of the ligand framework [41].

Alternatively, organic dyes have emerged as powerful redox tools in synthetic chemistry. With photoreductants rivaling transition‐metal complexes now accessible, challenging redox transformations can be addressed [42]. In addition, the properties of these dyes are readily modified through straightforward S_N_Ar chemistry [43].

This current work introduces a photochemical, transition‐metal‐free C─C coupling of phenols and halogenated azines (Scheme 1E) [44]. Using only an organic dye photocatalyst and base, the reaction generates phenoxyl and heteroaryl radicals via concurrent oxidation of phenols and reduction of azines, which then undergo radical cross‐coupling mechanisms. This redox‐neutral method operates with chloro‐, bromo‐, and iodo‐azines along with those bearing electron‐withdrawing groups, delivers shorter reaction times (3–16 h) than prior approaches [38, 39], and effectively functionalizes the *ortho‐*, *meta‐*, and *para*‐positions of phenols. DFT and mechanistic studies provide insight into the reaction pathways, while substrate clustering, high‐throughput experimentation (HTE), and machine learning (ML) analysis enable rapid mapping of chemical space and prediction of C─C versus S_N_Ar selectivity.

## 2 Results and Discussion

Carbazole‐based photocatalysts including 3DPA2FBN were identified as promising candidates for development of this transformation due to their versatile and tunable redox potentials [43, 45]. Importantly, 3DPA2FBN is significantly more cost‐effective ($15/100 mg) [46] than commonly used iridium photocatalysts such as [Ir(ppy)_2_(dtbbpy)]PF_6_ ($226/100 mg) [47] which were employed in prior heteroaryl bromide coupling work [39]. Overcoming limitations in substrate scope requires catalysts with greater oxidation and reduction potentials to act on the phenols and pyridines, respectively. The redox profile of 3DPA2FBN enables coupling of heteroaryl halides (−1.60 V vs. SCE) and phenols (+1.24 V vs. SCE) via an oxidative quenching cycle, with a potent reductive quenching cycle (+0.92; −1.92 V vs. SCE) also possible [45].

**SCHEME 2:**
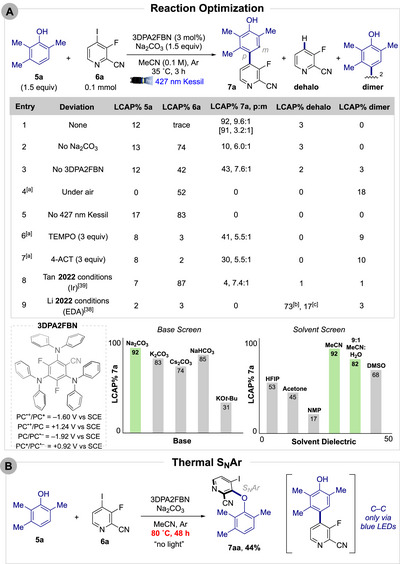
(A) Reaction optimization for cross‐coupling of phenol **5a** and pyridine **6a**. LCAP% = area percent of UPLC at 254 nm, omitting photocatalyst. Brackets denote product isolation after column chromatography with p:m ratio determined by ^19^F{^1^H}‐NMR. Data obtained from a single trial. ^[a]^Significant byproducts observed. ^[b]^S_N_Ar product **7aa** with protodehalogenation. ^[c]^S_N_Ar product **7aa**. (B) Thermal S_N_Ar of phenol **5a** and pyridine **6a** under model reaction conditions without light.

Using 2,3,6‐trimethylphenol (**5A**) and 3‐fluoro‐4‐iodopicolinonitrile (**6a**), reaction parameters were modulated to evaluate yield and selectivity (Scheme 2A). The main byproducts observed during optimization were protodehalogenation of the heteroaryl halide (**dehalo**) and phenol dimerization (**dimer**). Under the optimized conditions with a 1.5:1 phenol:pyridine ratio, cross‐coupled dimer **7A** was obtained in 92% (UV area percent) with minimal byproduct formation (entry 1). Both *para*‐ and *meta‐*isomers were detected, with the *para*‐position favored 9.6:1 by LCMS. Isolation by column chromatography afforded **7A** in 91% yield with a 3.2:1 *para*:*meta* (p:m) ratio by ^19^F{^1^H}‐NMR.

The presence of base is critical for reactivity: In its absence **6a** was poorly consumed and conversion to **7a** was low (10%) (entry 2). Carbonate and bicarbonate bases with various counterions proved effective, whereas stronger bases such as KO*t*‐Bu led to decreased yield (Scheme 2A, base screen). The reaction is largely tolerant to water, which aids base dosing on an HTE scale. High‐dielectric solvents enhance reactivity, likely by stabilizing charged intermediates, prolonging radical lifetimes, and facilitating electron transfer (Scheme 2A, solvent screen) [48].

Considerable product formation was observed without photocatalyst present (43%) alongside a lower p:m ratio (7.6:1). This result suggests a reactive EDA complex between the phenol and heteroaryl halide, potentially through π‐π stacking [16, 26, 27, 38] or halogen‐bonding [49]. Phenolate anions have also recently been shown to act as efficient visible‐light photocatalysts [50]. Under air, product formation is completely suppressed and phenol dimerization predominates with negligible pyridine consumption, aligning with competitive reduction of O_2_ (entry 4). No reaction occurred in the absence of blue light, confirming that the transformation is not a two‐electron process (entry 5). TEMPO and 4‐acetamido‐TEMPO (4‐ACT) lower the yield of **7a** and promote phenol dimerization (entries 6–7). Incomplete suppression suggests a solvent‐caged radical pair, consistent with our inability to trap either radical.

To benchmark our system, we evaluated the Li [38] and Tan [39] conditions, both of which showed substantially lower/no reactivity after 3 h of irradiation (entries 8–9). For the EDA conditions [38], S_N_Ar of the fluorine in **6a** was observed (17%) alongside protodehalogenation of this C─O product (73%) (see Supporting Information). These results indicate that our system is kinetically more favorable likely because it leverages both reactivity pathways concurrently and avoids competitive S_N_Ar.

Under harsher thermal conditions (80°C), the reaction proceeds exclusively via S_N_Ar, forging the C─O bond in 44% yield (Scheme 2B). Because the photochemical reaction remains below 80°C, pyridine **6a** exhibits high selectivity for C─C bond formation under blue light irradiation.

**SCHEME 3:**
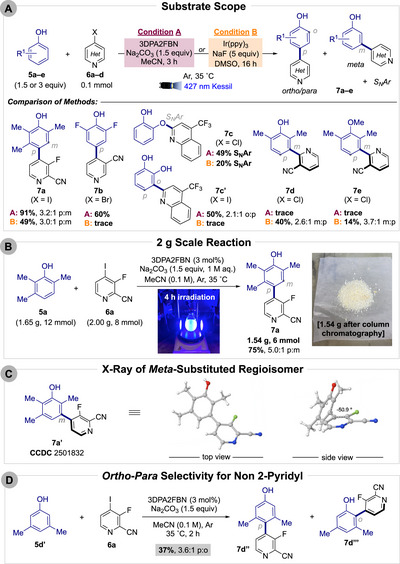
(A) Substrate scope and comparison of optimized cross‐coupling conditions. Isolated yields are shown. Halide starting materials shown in parentheses. (B) Gram‐scale (8 mmol) reaction for the cross‐coupling of phenol **5a** and pyridine **6a**. (C) X‐ray crystal structure of *meta*‐substituted isomer **7a′** (CCDC 2501832). (D) Selectivity of *ortho* versus *para* position for the coupling of **5d′** and **6a**.

Extensive bench‐top and HTE screening of photocatalyst, base, additive, solvent, and stoichiometry (see Supporting Information) identified two optimal reaction conditions, which were subsequently compared across a set of substrates (Scheme 3A). Under optimized conditions, we achieve productive C─C coupling across heteroaryl iodides (**6a**, **6c′**), bromides (**6b**), and even chlorides (**6d**). Although the regioselectivity is fixed for a given pairing, yields vary. 2,6‐Difluorophenol (**5B**) was *para*‐functionalized with 3‐cyano‐5‐bromopyridine (**6b**) in 60% yield (>20:1 p:m) with the 3DPA2FBN conditions, but no yield was observed with Ir(ppy)_3_. The use of Ir(ppy)_3_ generally results in low‐yielding reactions, likely because its lower oxidation potential (+0.77 V vs. SCE) [51] renders phenol oxidation inefficient. If S_N_Ar can occur below 35°C, then C─O coupling will predominate (**7c**). Installing heteroaryl bromides or iodides can circumvent this reactivity as these halides are less prone to S_N_Ar and are more readily reduced, as seen with chloro‐quinoline **6c** (S_N_Ar selective) versus iodo‐quinoline **6c′** (C─C selective).

2‐Pyridyl radicals exhibit a strong preference for *meta*‐type coupling (**7c′**, **7d**, **7e**), enabling access to motifs that are notoriously challenging in transition‐metal‐catalyzed processes [52]. Equivalents to the *meta*‐functionalization of phenols/phenol ethers have gained momentum through transient *para*‐blocking [53, 54] and migration of the substituents [55, 56]. In contrast, this method delivers direct *meta*‐functionalization. For example, 2‐chloro‐3‐cyanopyridine (**6d**) afforded the *meta*‐selective products **7d** (40%, 2.6:1 m:p) and **7e** (14%, 3.7:1 m:p) from corresponding pyridyl radical under Ir(ppy)_3_ catalysis.

The model reaction proved readily scalable (2 g, 8 mmol) furnishing **7a** in 75% yield (1.54 g isolated) after just 4 h with an improved p:m ratio (5.0:1) (Scheme 3B). This scalability is notable as related systems require up to 48 h on a 6 mmol scale [38]. Crystallization enabled us to obtain the single‐crystal X‐ray structure of minor *meta*‐isomer **7a′** (CCDC 2501832) [57], unambiguously confirming the regioisomer and its twisted biaryl bond (−50.9°) (Scheme 3C). While prior methods disclose exclusively *ortho*‐functionalization across all substrates [38, 39], the work herein enabled *para*‐selective coupling of 3,5‐dimethylphenol (**5d′**) with **6a**, favoring the *para*‐ over the *ortho*‐position in a 3.6:1 p:o ratio with 37% overall yield (Scheme 3D). This behavior however is limited for 2‐pyridyl radicals as evidenced above with the synthesis of **7c′** (2.1:1 o:p).

**SCHEME 4:**
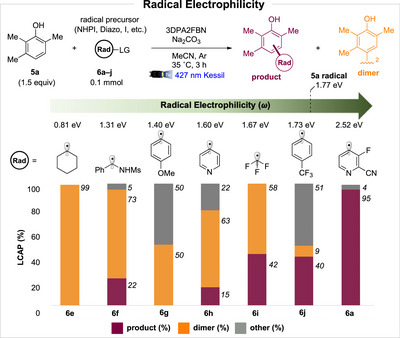
Radical electrophilicity as a function of reaction selectivity for cross‐coupling versus phenol homocoupling. LCAP = liquid chromatography area percent at 254 nm. Percentages refer to area percentages of reaction products only, starting materials and photocatalyst were omitted for this analysis. Other refers to unidentified reaction byproducts. Data obtained from a single trial.

Radical polarity plays a central role in governing radical reactivity and selectivity, a principle that underlies numerous synthetic transformations [58, 59]. Guided by these insights, we selected a suite of radical precursors spanning a range of electrophilicities and accessible reduction potentials (Scheme 4). These precursors were activated through diazo precursors [60], aryl sulfonium salts [61], and other related platforms [62, 63].

Increasing radical electrophilicity enhances cross‐coupling between the phenol and the corresponding radical whereas the phenol tends to dimerize in the presence of more nucleophilic radicals. The phenoxyl radical of **5a** has an electrophilicity of 1.77 eV. Conceptually, this phenoxyl radical acts as the nucleophilic partner requiring a more electrophilic heteroaryl radical for efficient coupling.

**SCHEME 5:**
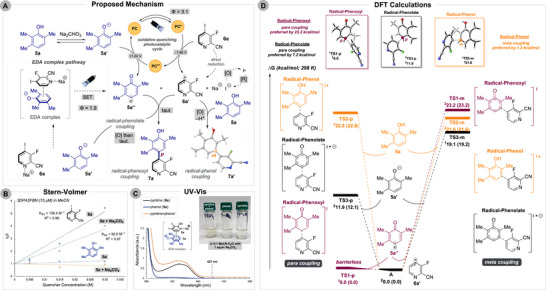
(A) Proposed reaction mechanism involving electron donor‐acceptor complexation as well as photocatalytic cycle (PC = 3DPA2FBN). (B) Stern‐Volmer analysis of pyridine **6a** and phenol **5a** in absence and presence of Na_2_CO_3_ for 3DPA2FBN (15 µM) in MeCN. Average of (*n* = 3) trials for each concentration. (C) UV‐Vis analysis of pyridine **6a**, phenol **5a**, and a 1:1 mixture in 9:1 MeCN:H_2_O (0.1 M) with 1 equiv Na_2_CO_3_ present. (D) DFT calculations for the addition of pyridyl radical **6a′** to phenol **5a**, phenolate **5a′**, and phenoxyl radical **5a″**. Free energies shown in kcal/mol at 298 K and were computed using UB3LYP‐D3/6‐311++G(d,p)‐CPCM(solvent)//UB3LYP‐D3/6‐31G(d,p). Values are shown for MeCN (DMSO).

Given the experimental evidence, we propose the reaction mechanism shown in Scheme 5A. Stern‐Volmer analysis of the photocatalyst in MeCN reveals strong‐quenching by pyridine and minimal quenching by phenol, with base having little effect–likely due to solubility limitations (Scheme 5B). Consistent with an oxidative quenching cycle (3DPA2FBN^•+^/3DPA2FBN* = –1.60 V vs. SCE; 3DPA2FBN^•+^/3DPA2FBN = +1.24 V vs. SCE) [45], the pyridyl radical **6a′** is generated via reduction, followed by phenolate **5a′** oxidation to form the phenoxyl radical **5a″**. Cross‐coupling of these radicals may then proceed through radical‐phenoxyl, radical‐phenolate, or radical‐phenol pathways.

UV‐Vis analysis of phenol **5a** and pyridine **6a** in the basic reaction media shows a slight bathochromic shift and color change in a 1:1 mixture, suggesting EDA complex formation (Scheme 5C), albeit not definitive [64]. While **6a** could be directly excited at 427 nm, the complex absorbs more strongly at this wavelength. The **5a** phenolate absorbs only below 330 nm, ruling out excited‐state phenolate chemistry. Reactivity without photocatalyst thus arises from either EDA excitation or direct pyridine reduction.

Quantum yield measurements indicate chain propagation in both the photocatalytic (*Φ* = 3.1) and EDA (*Φ* = 1.9) processes, likely contributing to their high efficiency [65]. Even in low‐yield EDA systems, chain mechanisms have been proposed [38]. After radical addition, oxidation of radical‐phenol or radical‐phenolate intermediates may proceed via pyridine reduction to initiate the chain. Iodide released post‐reduction may generate iodine radicals (I•) that can perform hydrogen atom transfer (HAT) or phenol oxidation in a chain‐like manner [37, 66]. Supporting studies include Tan's radical‐phenol coupling mechanism [39] and Li's radical‐phenolate and radical‐phenoxyl proposals [38]. We propose that all three pathways may operate concurrently in our system and we subsequently evaluated their feasibility and influence on regiochemical outcomes through computational analysis.

DFT calculations (see Supporting Information for full computational details) of the pyridyl radical addition lend evidence toward the observed regioselectivity (Scheme 5D). Cross‐coupling of pyridyl radical **6a′** and phenoxyl radical **5a″** (radical‐phenoxyl pathway) favors *para*‐addition by ∼23 kcal/mol via an essentially barrierless transformation. The radical‐phenolate pathway also favors the *para*‐position by 7.2 kcal/mol. Interestingly, pyridyl radical coupling to a neutral phenol (radical‐phenol) favors *meta*‐coupling by 1.2 kcal/mol. Alternative mechanisms such as phenoxyl radical addition to the heteroaryl halide are unlikely based on Stern‐Volmer quenching experiments.

Given the base equilibrium and excess phenol present, neutral phenols, phenolates, and phenoxyl radicals are all expected to exist in solution, giving rise to the observed regioselectivity spectrum for both of the conditions. However, the radical‐phenol coupling must contribute to a significant portion (roughly 30%) of the reaction outcome to account for the amount of *meta* product generated (**7a**, 3.2:1 p:m). As phenol dimerization also occurs, which indicates the presence of the phenoxyl radical, we hypothesize that the dominant pathway is likely the radical‐phenoxyl coupling (roughly 70%).

Together, these mechanistic insights motivated a systematic exploration of the broader chemical space. Data science has recently flourished in synthetic chemistry, particularly in the selection of robust substrate scope and thereby accelerating the development of innovative synthesis [67]. The recent chemical literature has also emphasized the minimization of redundancy in substrate scope and reduction of associated resource burden [68]. We therefore sought to explore the breadth of chemical space accessible by this transformation in respect to both phenol and heteroaryl halide, focusing primarily on six‐membered azine rings. We then gathered the top 1250 commercially available substrates for both classes, limited molecular weight to below 250 Da, and ranked them by number of vendors (see Supporting Information for search specifics).

**FIGURE 2:**
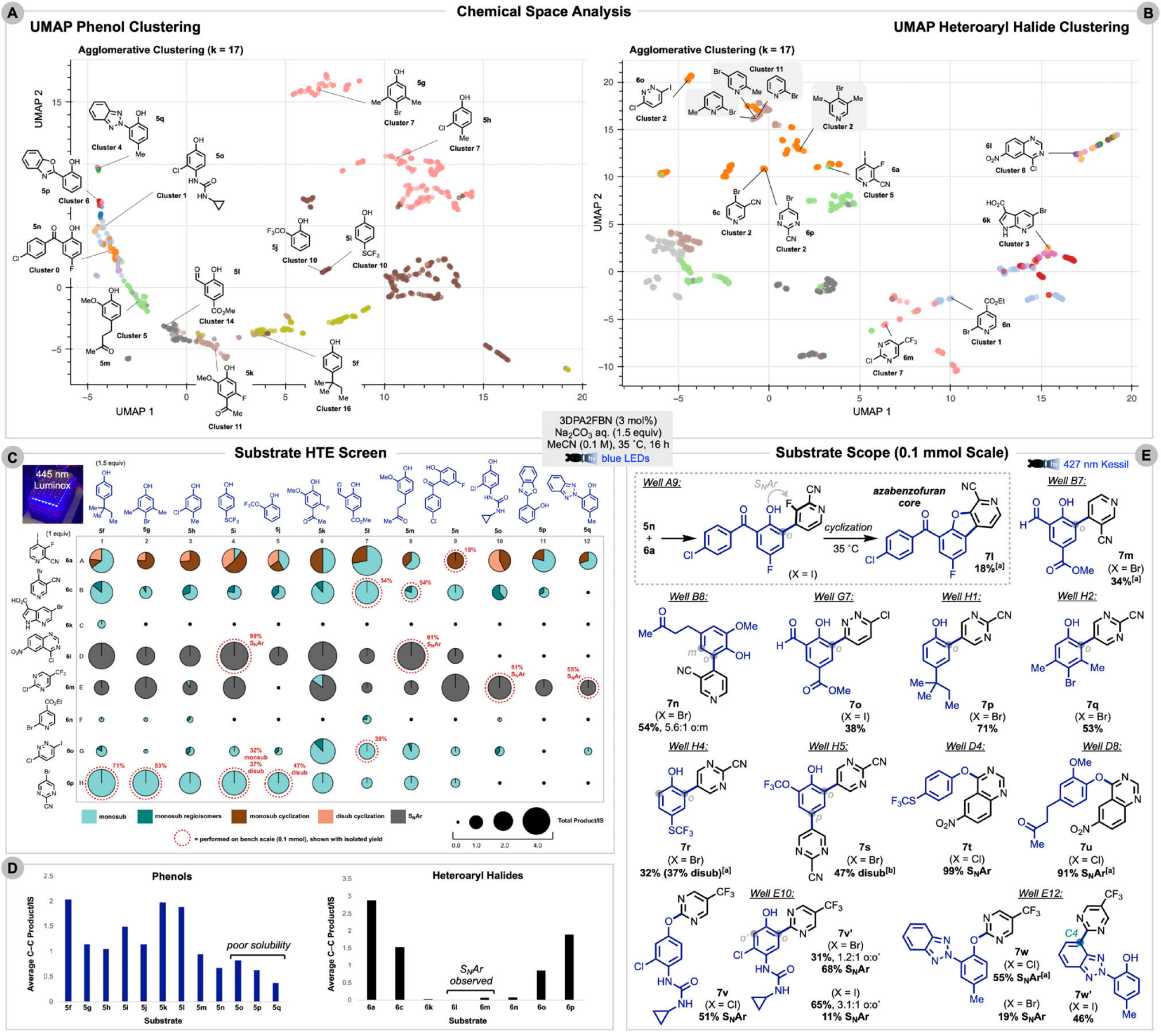
(A) Substrate clustering of 1250 commercially available phenols using Uniform Manifold Approximation and Projection (UMAP). Twelve phenols highlighted are shown with associated clusters. (B) Substrate clustering of 1250 commercially available heteroaryl halides using UMAP. Eight heteroaryl halides are shown with associated clusters. Four substrates from Tan and co‐workers [39] highlighted in gray. (C) 96‐well HTE plate. Product to internal standard ratio represented by size of the pie charts. Isolated yields are shown in red for wells highlighted with dashed circles. 4,4′‐Di‐*tert*‐butylbiphenyl utilized as the internal standard for the analysis. Reactions conditions used are shown in the central gray box with 1.5:1 phenol:pyridine ratio. Data obtained from a single trial. (D) Average product to internal standard ratio for each substrate. The average is of (*n* = 8) trials for phenols and (*n* = 12) for heteroaryl halides. Isolated yields and product structures shown for the 12 reactions repeated on bench‐scale (0.1 mmol). ^[a]^1:1.5 phenol:pyridine ratio. ^[b]^1:2.2 phenol:pyridine ratio.

Using this collection, physicochemical descriptors for each substrate were generated from associated SMILES strings with RDKit [69]. Descriptors with missing values or high correlations (assessed via Pearson correlation) were dropped. Uniform Manifold Approximation and Projection (UMAP) [70] was employed to visualize the agglomerative clustering (k = 17) of the substrates. To ensure coverage of chemical diversity, 12 phenols (**5f–q**) were selected from distinct clusters (Figure 2A). Figure 2B shows the heteroaryl halide chemical space from which we selected two heteroarenes for our initial scope (**6a**, **6c**) and six additional substrates (**6k–p**). Four of the substrates reported by Tan and co‐workers [39] occupy clusters 2 and 11 (gray), while our selections probe further chemical space.

To rapidly survey the chemical landscape of our system, we evaluated the curated set of 12 phenols (**5f–q**) and eight heteroaryl halides (**6a**, **6c**, **6k–p**) in a 96‐well HTE format [71] (Figure 2C). This approach circumvents the labor‐intensive purification and characterization otherwise required for each reaction. For operational simplicity, we used 1.0 M aqueous Na_2_CO_3_ as the base and conducted the reactions in a Lumidox LED photoreactor (see Supporting Information). Product formation was observed across the plate using LCMS with an internal standard. Several reactions produced complex mixtures of multiple regioisomers, disubstitution, or combinations of S_N_Ar and C─C coupling. Notably, phenols in the same clusters (**5g**/**5h**, **5j**/**5i**) and pyridines in the same cluster (**6c**/**6o**/**6p**) performed similarly, reinforcing that the clustering captures the underlying similarities of the substrates.

The average performance of each substrate for C─C coupling is shown in Figure 2D. All of the phenols demonstrated some product formation across the tested heteroarenes with a great tolerance to varying electronic and steric effects, enabled by the high oxidation potential of 3DPA2FBN^•+^/3DPA2FBN (+1.24 V vs. SCE). Phenols **5o**, **5p**, and **5q** suffered from low yields likely due to their poor solubility in the reaction mixture. Only half of the tested heteroaryl halides (**6a**, **6c**, **6o**, **6p**) formed appreciable amounts of the desired C─C coupling. After isolation on bench‐scale (0.1 mmol), it was discovered that heteroarenes **6l** and **6m** were undergoing selective S_N_Ar.

Several reactions were repeated on a 0.1 mmol scale, delivering moderate to good yields for *ortho*‐functionalization with a range of *N*‐heterocycles (Figure 2E). With pyridine **6A**, the subsequent adjacent fluorine underwent intramolecular S_N_Ar post C─C coupling to construct azabenzofuran **7l** in 18% yield. Forging the fused heterocycle in one‐pot, this approach offers an alternative to recent efforts in Ni‐catalyzed ring contraction [72]. Selectivity for *ortho*‐ over *meta*‐functionalization was observed for the couplings resulting in products **7m**, **7n**, **7o**, and **7p**. Compound **7o**, obtained in 38% yield, features a phenol‐pyridazine bond reminiscent of that found in the pharmaceuticals PRT3789 [9] and AZD4144 [10]. Coupling electron‐poor phenols (**5i**, **5j**) with pyrimidine **6p** led to disubstitution, affording **7r** in 69% yield (1.2:1 disub:monosub) and trimer **7s** in 47% yield.

Halide swapping was explored to favor C─C coupling over the previously observed S_N_Ar of **6l** (**7t**, **7u**) and **6m** (**7v**, **7w**). The iodo‐quinazoline derivative was difficult to access due to hydrolysis, but bromo‐ and iodo‐pyrimidine analogues delivered C─C product (**7v′**) in 31% and 65% yield, respectively, albeit with some S_N_Ar. For drometrizole (**5q**), S_N_Ar was observed for the chloride and bromide pyrimidine derivatives. With the iodide, no C─H functionalization of the phenolic backbone was observed; instead, coupling occurred at C4 of the benzotriazole ring to form **7w′** in 46% yield (Figure 2E). The radical addition to neutral benzotriazole is feasible (10.5 kcal/mol) (see Supporting Information), highlighting its potential in conjugated polymer synthesis [73] and as a ligand for photoactive borates [74].

**SCHEME 6:**
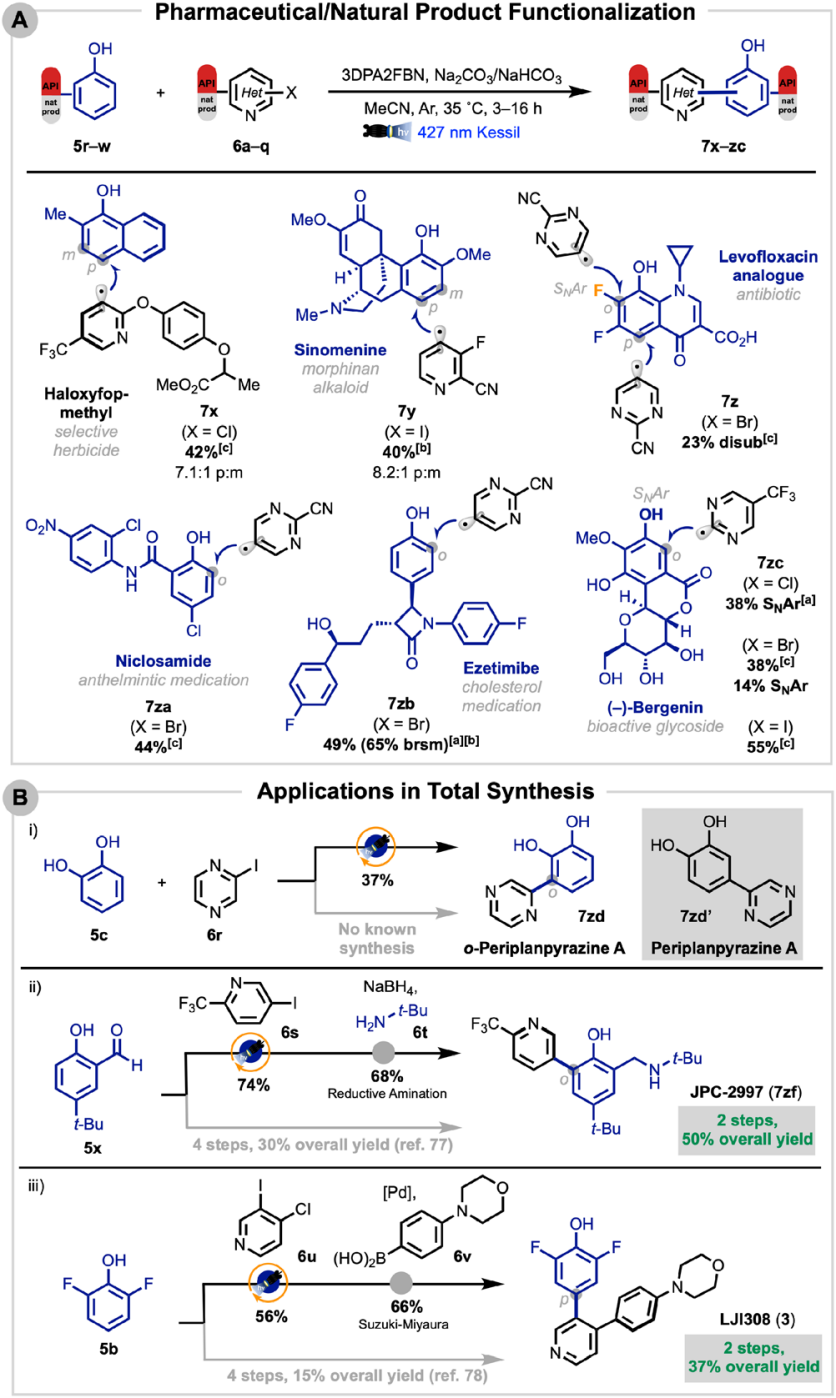
(A) Late‐stage functionalization of pharmaceuticals and natural products. Heteroaryl halides shown as their associated radicals with parent halides illustrated in parentheses. Isolated yields are shown. Reactions performed on a 0.10 mmol scale. S_N_Ar of (–)‐bergenin (**5w**) occurred on phenol with label depicted. ^[a]^NaHCO_3_ used as base. ^[b]^3 h reaction time. ^[c]^16 h reaction time. (B) Application of reaction in total synthesis of pharmaceuticals and natural products. i) One step synthesis of *ortho*‐periplanpyrazine A (**7zd**). ii) Two step synthesis of antimalarial JPC‐2997 (**7zf**). iii) Two step synthesis of RSK inhibitor LJI308 (**3**).

After applying this method to a diverse range of commercially available substrates, the scope was examined in the late‐stage functionalization of pharmaceuticals and natural products. Using biologically relevant heteroaryl halides (haloxyfop‐methyl, **6q**) and phenolic compounds **5s–5w**, we were able to furnish C─C cross‐coupling products in moderate yields (Scheme 6A). The weaker base NaHCO_3_ was utilized instead of Na_2_CO_3_ for certain base‐sensitive compounds.

Selective C─C coupling of naphthol **5r** and the heteroaryl chloride in haloxyfop‐methyl (**6q**) afforded **7x** in 42% yield (7.1:1 p:m). The morphinan alkaloid sinomenine (**5s**) was preferentially *para*‐functionalized in 40% yield (8.2:1 p:m). An analogue of the antibiotic levofloxacin (**5t**) was difunctionalized at the *para‐* and *ipso*‐fluoro‐positions in 23% overall yield. This S_N_Ar mirrors previous findings on pyridination [26] and amination [27, 28, 75]. The approved phenolic drugs niclosamide (**5u**) and ezetimibe (**5v**) were both *ortho*‐functionalized in moderate yields with pyrimidine **6p**. The bioactive glucoside (–)‐bergenin (**5w**) underwent S_N_Ar or C─C coupling depending on the halide of 5‐(trifluoromethyl)pyrimidine.

The synthetic utility of this method was secured across the total synthesis of several natural products and bioactive compounds (Scheme 6B). *ortho*‐Periplanpyrazine A (**7zd**), a previously unknown derivative of periplanpyrazine A [76], was obtained in a single step in 37% yield. Cross‐coupling of **5x** and **6s** established the key biaryl bond in JPC‐2997 (**7zf**) [77] in 74% yield. Subsequent reductive amination with *tert*‐butylamine furnished JPC‐2997 (**7zf**) in 50% overall yield over two steps, halving the step count and improving yield from prior work [77]. Selective C─C coupling of the iodide in dihalide **6u** gave the LJI308 intermediate (**7zg**) in 56% yield and a Suzuki‐Miyaura coupling completed the synthesis of potent RSK inhibitor LJI308 (**3**) [11] in 37% overall yield over two steps, nearly doubling reported yield while reducing step count [78].

**SCHEME 7:**
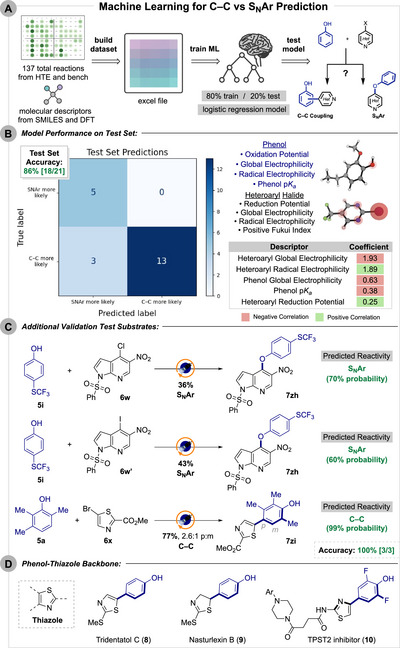
(A) Machine learning workflow for prediction of C─C versus S_N_Ar reaction selectivity given a pairing of phenol and heteroaryl halide. (B) Model performance on a random test set. Model parameters for each phenol and heteroaryl halide shown. Top four descriptors and their respective coefficient for the logistic regression model with sign of correlation depicted by color. (C) Additional test substrate pairings and their predicted reactivity based on the machine learning model. Isolated yields shown below the reaction arrow. (D) Phenol‐thiazole backbone.

ML has recently emerged as a powerful tool in organic synthesis, enabling advances in reaction prediction and planning [79, 80, 81, 82]. After exploring a broad substrate set and observing a continuum of C─C versus S_N_Ar selectivity, a ML model was developed to predict product outcomes for a given phenol/heteroaryl halide pairing for the standard (3DPA2FBN/MeCN) conditions (Scheme 7a). A dataset of 137 reactions from both HTE‐scale (0.1 µmol) and bench‐scale (>0.1 mmol) using these conditions was then compiled.

Selected molecular descriptors from *Rowan Scientific* online platform and DFT were chosen to reflect key mechanistic features [83, 84, 85, 86, 87, 88, 89]. Oxidation potential, global and radical electrophilicity, and p*K_a_* were selected for phenols, whereas reduction potential, global and radical electrophilicity, and the positive Fukui index were selected for heteroaryl halides (Scheme 7B).

After removing zero yield cases, a five feature logistic regression model trained with 80% of the data gave the most reliable performance among the algorithms tested (see Supporting Information). Scheme 7B visualizes the binary confusion matrix for the model, which achieved 86% accuracy (18/21) on the test set. Cross‐validation confirmed the model was both robust and reliable, with a low standard deviation for the training set (accuracy: 0.89 ± 0.09).

The table in Scheme 7B lists the top five descriptors and their corresponding coefficients, highlighting the dominant influence of heteroaryl properties on model performance. Global electrophilicity of the heteroarene strongly tracks with S_N_Ar, while higher radical electrophilicity and lower reduction potential (more easily reduced) favor C─C coupling. The lower radical electrophilicity for 2‐pyridyl radicals [58] may influence their regioselectivity, driving toward more *meta*‐functionalization. Phenol global electrophilicity and p*K_a_* both correlate negatively with C─C selectivity. The three misclassified cases correspond to low‐yielding reactions (<5% conversion) (see Supporting Information).

Additional validation cases were run under standard bench‐scale conditions (Scheme 7C). The chloro (**6w**) and iodo (**6w′**) variants of a 1*H*‐pyrrolo[2,3‐*b*]pyridine analogue provided the S_N_Ar adduct **7zh** in 36% and 43% yield, with the ML model correctly predicting this selectivity (70% and 60% probability). To probe reactivity beyond the six‐membered heterocycles tested throughout this study, a five‐membered heterocycle (**6x**) was examined, affording the C─C product **7zi** in 77% yield (2.6:1 p:m), which the model also correctly predicted (99% probability). The phenol‐thiazole scaffold accessed here appears in biologically relevant compounds, including natural product tridentatol C (**8**) [90] and nasturlexin B (**9**) [91] as well as recently reported TPST2 inhibitor **10** [92] (Scheme 7D). Overall, the model performed well on the test set and external validation cases, including substrates beyond the trained chemical space, while remaining computationally inexpensive.

## 3 Conclusion

Phenols and *N*‐heteroarenes are ubiquitous in medicinally relevant compounds and the method developed herein enables their direct C(sp^2^)─C(sp^2^) coupling to access congested biaryls under mild, transition‐metal‐free conditions. By leveraging halide susceptibly to S_N_Ar, this competitive reaction can be overcome to favor C─C coupling. This strategy has been demonstrated across more than 25 examples (18%–91% yield), is scalable to multi‐gram quantities, and is suitable for late‐stage functionalization of natural products and pharmaceuticals. Cascading C─C and S_N_Ar provide one‐pot access to azabenzofurans, while the method also enables direct C4 functionalization of benzotriazoles. The resultant phenols can also be activated through sulfur(VI) fluoride exchange (SuFEx) chemistry for further functionalization to generate multi‐substituted aryls. Mechanistic insights from DFT, Stern‐Volmer, and UV‐Vis studies, combined with UMAP‐guided substrate selection and HTE, enabled efficient exploration of chemical space. Integrating bench‐scale and HTE data enabled a robust, predictive ML model accurate across both in‐sample and out‐of‐sample cases. Future work will expand this approach to additional heterocycles and explore its application in the total synthesis of bioactive molecules.

## Conflicts of Interest

The authors declare no conflicts of interest.

## Supporting information




**Supporting File 1**: The authors have cited additional references within the Supporting Information [1–13].

**Supporting File 2**: anie71621‐sup‐0002‐Data.zip.

## Data Availability

The data that supports the findings of this study are available in the Supporting Information of this article.
