# Supplementary material for: Photocatalytic Cross‐Coupling of Phenols and Heteroaryl Halides With Machine Learning‐Guided Reaction Prediction
Source: Angew Chem Int Ed Engl. 2026 Mar 5;65(16):e8222003. doi: 10.1002/anie.8222003 (PMC13080434; doi:10.1002/anie.8222003)
Supplement: Supplementary file 1 — Supporting File 1: The authors have cited additional references within the Supporting Information [1–13]. [file ANIE-65-e8222003-s002.pdf]

Supporting Information

Photocatalytic Cross-Coupling of Phenols and Heteroaryl Halides with Machine Learning-Guided Reaction Prediction

Matthew C. Carson,[a] Alice Wu,[a] Kalyana B. Duggal,[a] Madeline E. Rotella,[a] and Marisa C. Kozlowski*[a]

[a]Department of Chemistry, Roy and Diana Vagelos Laboratories, University of Pennsylvania, Philadelphia, Pennsylvania 19104-6323, United States

*To whom correspondence should be addressed: marisa@sas.upenn.edu

Table of Contents

1. General Information and Materials S3
2. Literature Search Results S4
3. Reaction Discovery and Optimization S7
4. Synthetic Procedures for Starting Materials S13
5. General Procedures for Photochemical Cross-Coupling S15
6. Product Characterization for Photochemical Cross-Coupling S17
7. Radical Electrophilicity Screen S39
8. Experimental Procedures for Application in Total Synthesis S40
9. UV-Vis, Fluorescence, and Quantum Yield Experiments S42
10. Mechanism Proposals S49
11. High-Throughput Experimentation (HTE) S51
12. UMAP Substrate Clustering S55
13. Machine Learning S59
14. Computational Studies S63
15. X-Ray Crystallography Data S103
16. References S109
17. HRMS Data S110
18. NMR Spectral Data S132

1. General Information and Materials

All reagents were obtained from commercial sources with the highest purity and used without further purification, unless otherwise noted. 3DPA2FBN (CAS: 1403850-00-9) was obtained from Ambeed Inc. (A1219868). Argon tanks were sourced from Airgas. When necessary, solvents were dried and purified before use via a solvent purification system (DCE) or used from an AcroSeal™ bottle (MeCN, 1,4-dioxane, NMP, acetone, DMSO). Reactions requiring heating were performed either in round-bottom flasks using a heated silicon oil bath or in Chemglass (4 mL or 8 mL or 20 mL) microwave reactions vials (borosilicate glass) with 20 mm aluminum seals and PTFE septa (rated up to 20 bar) using a Chemglass OptiChem block heated with an IKA C-MAG HS7 heat/stir plate. For light irradiation, Kessil PR160L (λ = 390 nm, 427 nm, or 440 nm) lamps (100% intensity, max 45W, 7 cm from wall of flask, no filters) were employed with a commercial blade fan for cooling in Chemglass (4 mL or 8 mL or 20 mL) microwave reactions vials (borosilicate glass) with 20 mm aluminum seals and PTFE septa (rated up to 20 bar). A 3D-printed photoreactor box fitted for one Kessil PR160L lamp (developed by Aubineau and co-workers)[1] was utilized for the reactions, using the first row of the vial wells (4 positions, see **Figure S2**). Rubber septa were placed in the empty wells to minimize the escape of light.

Analytical thin layer chromatography (TLC) was performed on EM Reagents 0.25 mm silica-gel 254-F plates. Visualization was accomplished with UV light. Liquid chromatography-mass spectrometry was performed on a Waters ACQUITY SQ (single-quadrupole) mass detector equipped with a Waters variable wavelength detector (VWD) at 254 nm. Analytical-scale liquid chromatography was performed using a Waters ACQUITY HSS C18 column (Acquity HSS C18; 1.8 µm; 50 mm x 2.1 mm; Column T = 30 ˚C). For standard LCMS analysis, the following method parameters were used: inlet flow rate = 0.75 mL/min; mobile phase = MeCN:H_2O: 0.1% formic acid (FA); detection wavelength = 190-400 nm; positive/negative-mode electrospray ionization (ESI+/-); nebulizer = 700 L/h; cone gas (N_2) = 30 L/h; source 150 ˚C; desolvation 450 ˚C. Gradient (4.00 min, 0.1% formic acid additive): 3.30 min gradient from 5% MeCN:H_2O to 99% MeCN:H_2O; 0.55 min at 99% MeCN:H_2O; 0.05 min gradient to 5% MeCN:H_2O; 0.10 min at 5% MeCN:H_2O.

Automated flash chromatography was performed using a Teledyne ISCO CombiFlash® (254 nm & 280 nm UV detector) with RediSep R_f Gold® disposable silica (normal-phase) or C18 (reverse-phase) columns (60 Å porosity, 20–40 µm) or flash chromatography with forced flow of the indicated solvent system on Silica-P flash silica gel (50-63 µm mesh particle size). For normal-phase chromatography, *n*-hexanes:EtOAc mobile phase was used, and for reverse-phase chromatography, H_2O:MeCN (both with 0.1% formic acid (FA) mobile phase was used. Unless otherwise noted, yields refer to isolated material based on product purity (≥95%) determined by ^{1}H-NMR spectroscopy following purification by column chromatography.

NMR spectra (^{1}H, ^{13}C{^{1}H}, ^{19}F{^{1}H}) were recorded on a Fourier transform NMR spectrometer at 298 K at 400/600 MHz (for ^{1}H), 101/151 MHz (for ^{13}C{^{1}H}), and 376 MHz (for ^{19}F{^{1}H}). Chemical shifts are reported relative to the solvent resonance peak ($CDCl_3$: δ = 7.26 ppm, DMSO-d_6: δ = 2.50 ppm, acetone-d_6: δ = 2.05 ppm, methanol-d_4: δ = 3.31 ppm) for ^{1}H-NMR spectra and ($CDCl_3$: δ = 77.16 ppm, DMSO-d_6: δ = 39.52 ppm, acetone-d_6: δ = 29.82 ppm, methanol-d_4: δ = 49.03 ppm) for ^{13}C{^{1}H}-NMR spectra. NMR spectra multiplicities are reported as follows: chemical shift, multiplicity (s = singlet, d = doublet, t = triplet, q = quartet, br s = broad singlet, dd = doublet of doublets, ddd = doublet of doublet of doublets, dt = doublet of triplets, td = triplet of doublets, m = multiplet, coupling constants (J, reported in Hz), and number of protons. ^{13}C{^{1}H} and ^{19}F{^{1}H} spectra were both fully proton decoupled. ^{19}F splitting was observed in the ^{13}C{^{1}H} spectra: -CF_3 (quartets), -F (doublets).

Accurate mass measurement analyses were conducted on a Bruker ScimaX Magnetic Resonance Mass Spectrometer (MRMS) (FTICR) equipped with electrospray ionization (ESI) or Thermo Scientific™ Q Exactive™ HF-X Hybrid Quadrupole-Orbitrap™ mass spectrometer. Samples were taken up in a suitable solvent for analysis (typically MeCN:H_2O) and analyzed in positive or negative modes via direct infusion. The instrument routinely achieved sub-ppm mass accuracy and Isotopic Fine Structure (IFS) resolution, enabling high-confidence molecular formula determination. The software calibrates the instruments, and reports measurements, by use of neutral atomic masses; the mass of the electron is not subtracted (positive ions) or added (negative ions).

2. Literature Search Results

The below search inquiries were utilized to gather the data on the substructures described in this study and to generate **Figure S1** and **Figure 1** of the manuscript. To gain insight on the number of substructures containing free phenols, six-membered azines, and the coupled products between the two, the database Reaxys was utilized on August 28, 2025.

Substructure search queries, as demonstrated in the screenshots, were conducted. All searches were refined to keep fragments together and limit fragments to 1.

Phenols

The oxygen atom in the phenol was locked to prevent any substituents on the O (s*), allowing for the search to be limited to free phenol substructures.

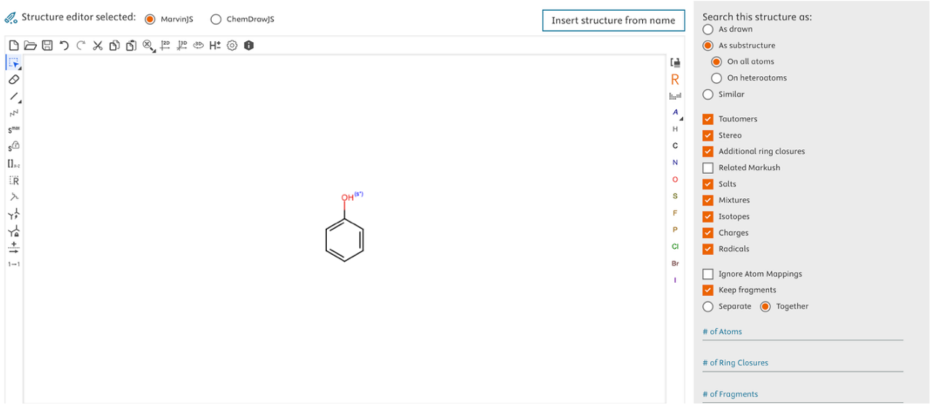

Total Substructures	2,753,948
w/ Pharmacological Data	752,009
Isolated from Natural Source	90,929

Azines

The azine search encompasses cores in the order of pyridines, pyridazines, pyrimidines, and pyrazines.

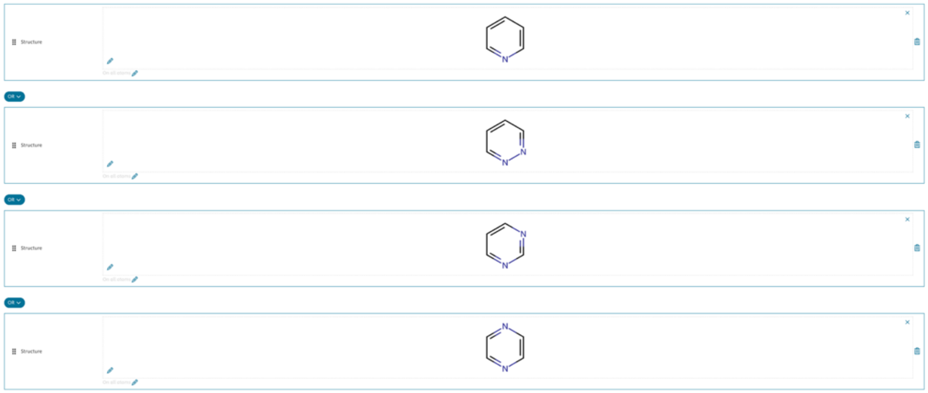

Total Substructures	11,799,443
w/ Pharmacological Data	3,068,845
Isolated from Natural Source	6,217

C-O Cyclization Products

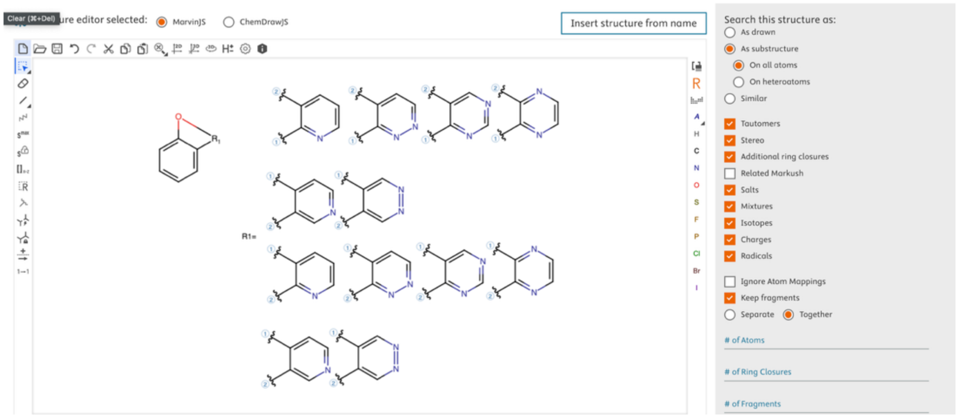

Total Substructures	58,115
w/ Pharmacological Data	2,165
Target GTPase KRAS	12

C-C Products

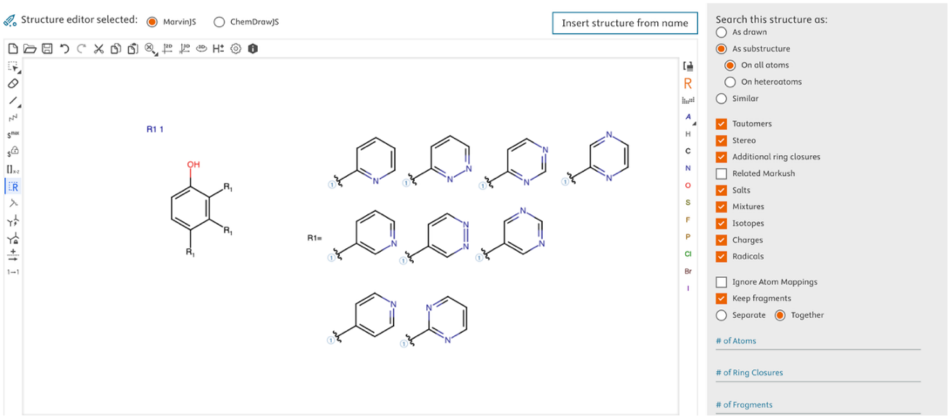

Total Substructures	119,665
w/ Pharmacological Data	34,770
Isolated from Natural Source	267
Approved Drug	1 (Sotorasib)
Most Common Target (GTPase KRAS)	3,824

S_NAr Products

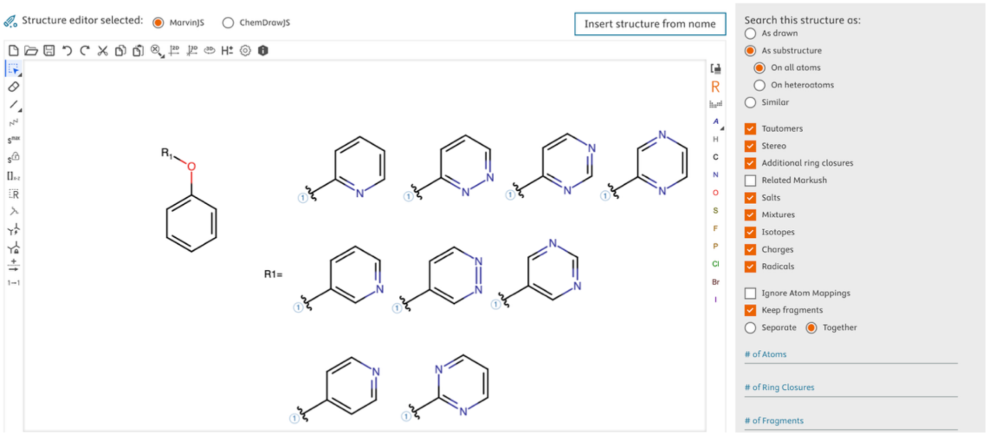

Total Substructures	351,032
w/ Pharmacological Data	126,318
Isolated from Natural Source	3

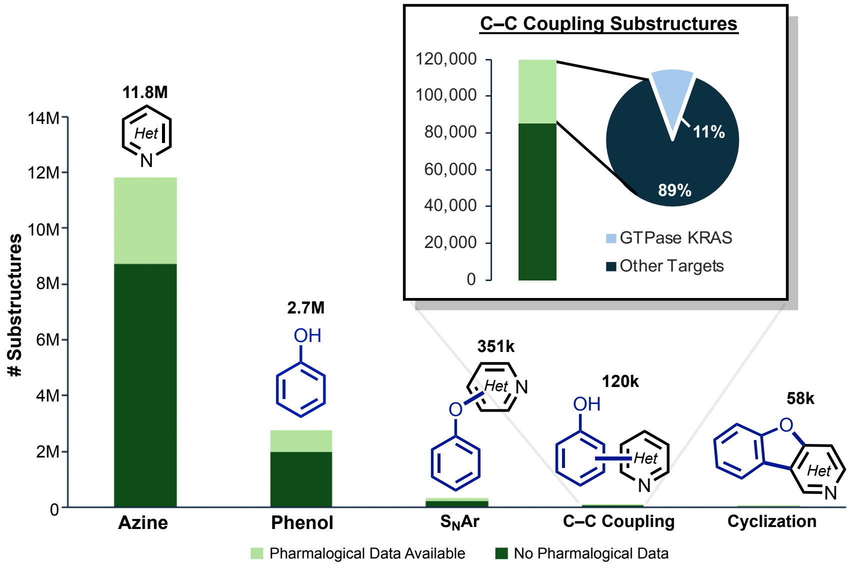

Figure S1. Substructures of azines and phenols and their associated bond formations.

3. Reaction Discovery and Optimization

[**WARNING**: Reactions with blue light require the use of special eye protection to prevent damage to vision. Special care must be taken, preferably blue light glasses.]

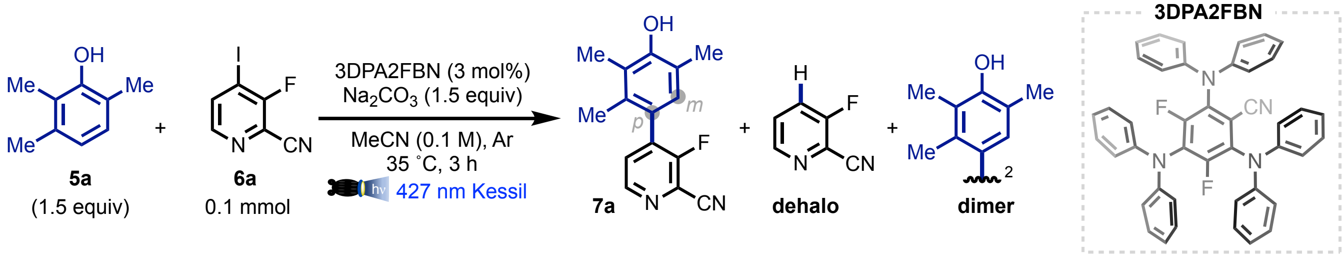

General Procedure A: Optimization and Controls

An 8 mL microwave vial equipped with a magnetic stir bar was charged with 3-fluoro-4-iodopicolinonitrile (**6a**, 0.10 mmol, 1.0 equiv), 2,3,6-trimethylphenol (**5a**, 0.15 mmol, 1.5 equiv), 3DPA2FBN (0.0030 mmol, 3.0 mol%), Na_2CO_3 (0.15 mmol, 1.5 equiv), and dry MeCN (1.0 mL, 0.10 M). The vial was crimp-capped and sparged by bubbling argon through the solvent via a needle for 5 min prior to irradiation with a 427 nm Kessil lamp (100% intensity, max 45 W, 7 cm from wall of flask) and commercial fan cooling (~35 ˚C) (see **Figure S2** for setup). After 3 h, the reaction mixture was cooled to room temperature, opened to air, and an aliquot (50 μL) of the reaction mixture was diluted with MeCN (950 μL) and analyzed by LCMS at 254 nm detection.

Note: Base additives were typically oven-dried for 24 h prior to the reaction.

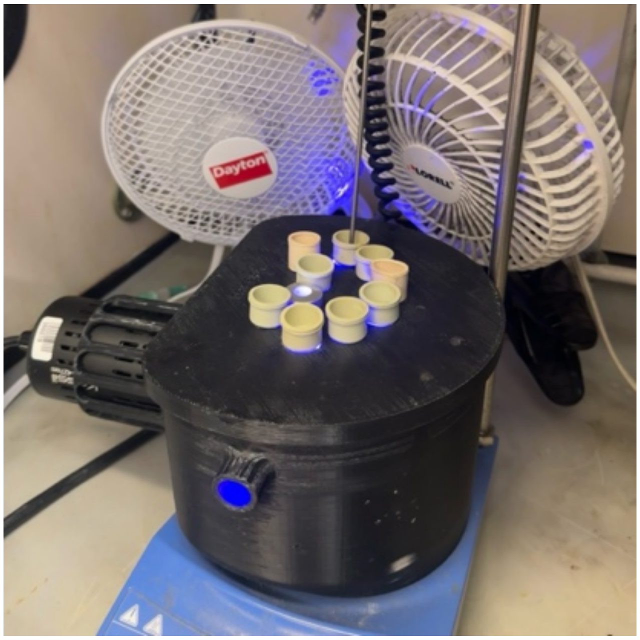

Figure S2. Typical photochemical setup used in this study.

Table S1. Additive Screening of 5a and 6a Cross-Coupling with 3DPA2FBN

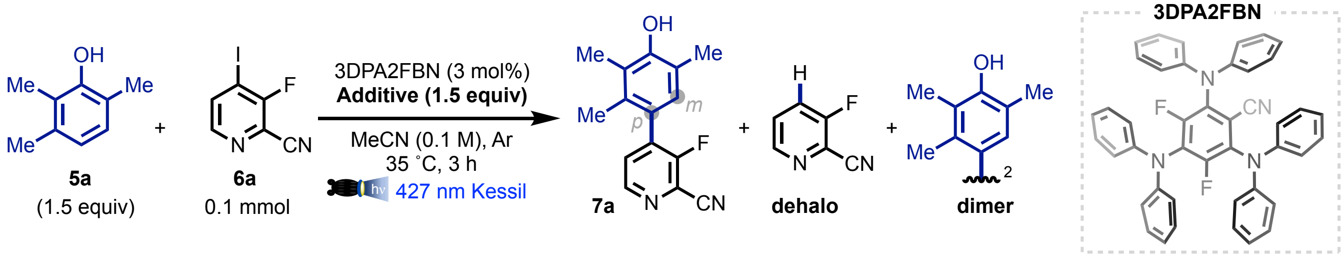

Entry	Additive	LCAP% 5a	LCAP% 6a	LCAP% 7d	LCAP% Dehalo	LCAP% Dimer
1	None	13	74	10	3	0
2	None + PTH as PC	7	77	5	1	0
3	Cs_2CO_3	4	2	74	3	17
4	Na_2CO_3	12	trace	92<br>[91, 3.2:1 p:m]	3	0
5	K_2CO_3	9	1	83	3	4
6	$NaHCO_3$	11	0	85	3	0
7	KO*t*-Bu	13	3	31	3	10
8	B(O*i*-Pr)$_3$ + Cs_2CO_3	15	1	79	6	0
9	DBU	17	6	59	0	19
10	TMG	7	33	16	0	35
11	Cp_2TiCl_2 (20 mol%)	17	76	0	7	0

LCAP% = area percent of UPLC UV trace at 254 nm, omitting photocatalyst. *meta*- and *para*-Isomers of **7a** were combined for this analysis. Brackets denote product isolation after column chromatography with p:m ratio determined by $^{19}F\{^1H\}$-NMR. PTH standards for 10-phenylphenothiazine.

Table S2. Solvent Screening of 5a and 6a Cross-Coupling with 3DPA2FBN

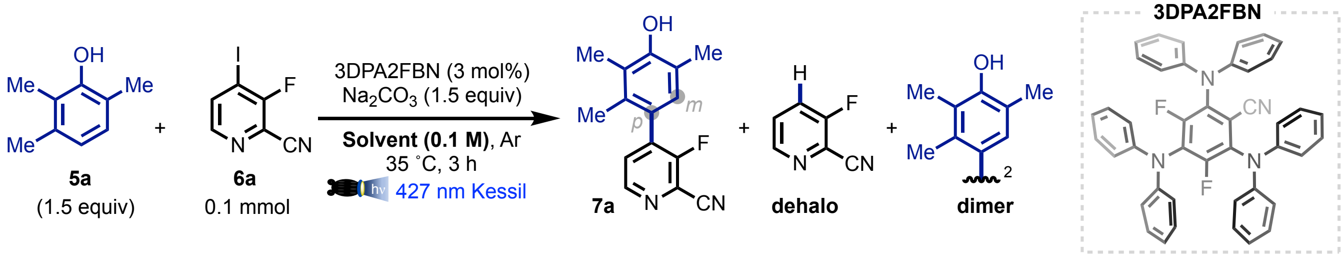

Entry	Solvent	LCAP% 5a	LCAP% 6a	LCAP% 7d	LCAP% Dehalo	LCAP% Dimer
1	MeCN	12	trace	92<br>[91, 3.2:1 p:m]	3	0
2	9:1 MeCN:H_2O	9	0	82	3	7
3	HFIP	11	9	53	4	23
4	DMSO	13	1	68	7	12
5	NMP	23	0	17	51	10
6	Acetone	12	41	45	2	1
7	MeOH	17	4	57	15	7
8	DME	18	53	13	14	1
9	DCE	18	2	35	4	42
10	CH_2Cl_2	14	38	32	2	15
11	1,4-Dioxane	25	2	22	16	36

LCAP% = area percent of UPLC UV trace at 254 nm, omitting photocatalyst. *Meta*- and *para*-isomers of **7a** were combined for this analysis. Brackets denote product isolation after column chromatography with p:m ratio determined by $^{19}F\{^1H\}$-NMR.

Table S3. Controls Reactions of 5a and 6a Cross-Coupling with 3DPA2FBN

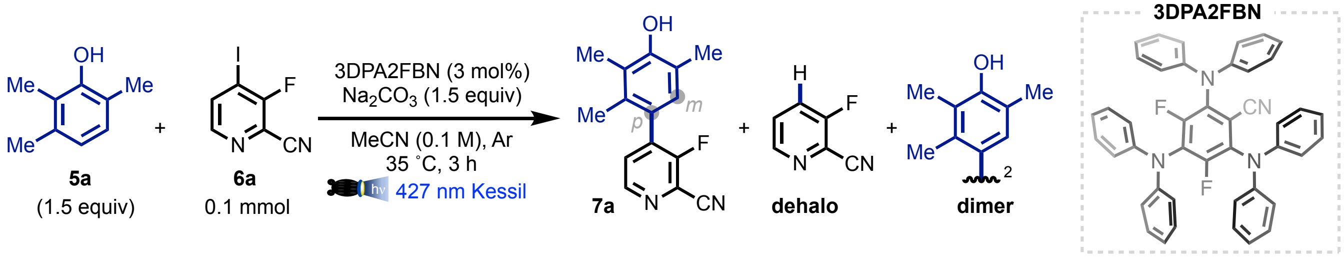

Entry	Deviation	LCAP% 5a	LCAP% 6a	LCAP% 7d, p:m	LCAP% Dehalo	LCAP% Dimer
1	None	12	trace	92, 9.6:1<br>[91, 3.2:1]	3	0
2	No Na_2CO_3	13	74	10, 6.0:1	3	0
3	No 3DPA2FBN	12	42	43, 7.6:1	2	3
4[a]	Under air	0	52	0	0	18
5	No 427 nm Kessil	17	83	0	0	0
6[a]	TEMPO (3 equiv)	8	3	41, 5.5:1	0	9
7[a]	4-ACT (3 equiv)	8	2	30, 5.5:1	0	10
8	Tan **2022** conditions[2] at 3 h	7	87	4, 7.4:1	1	1
9	Li **2022** conditions[3] at 3 h	2	3	0	73,[b] 17[c]	3

LCAP% = area percent of UPLC UV trace at 254 nm, omitting photocatalyst. Brackets denote product isolation after column chromatography with p:m ratio determined by $^{19}F\{^1H\}$-NMR. [a]Significant byproducts observed. [b]S_NAr product with protodehalogenation. [c]S_NAr product. Tan **2022** conditions[2]: $[Ir(ppy)_2(dtbbpy)]PF_6$ (3 mol%), DCE (0.1 M), Ar, 35 ˚C, 427 nm Kessil. Li **2022** conditions[3]: Cs_2CO_3 (1.5 equiv), DMSO (0.4 M), Ar, 35 ˚C, 427 nm Kessil.

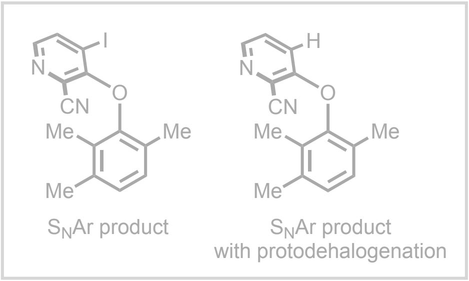

Table S4. Initial Screening of 5d and 6d Cross-Coupling

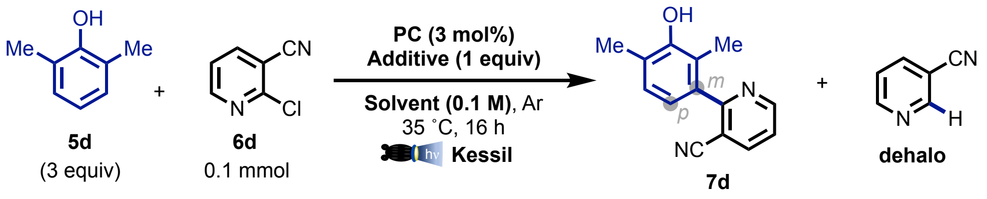

Entry	PC	Additive	Solvent	Kessil	LCAP% 7d, m:p	LCAP% Dehalo
1[4]	NPh_3 (20 mol%)	KF (3 equiv)	6:1 MeCN:H_2O	390 nm	10, 2.3:1	26
2	Ir(ppy)$_3$	KF (3 equiv)	6:1 MeCN:H_2O	390 nm	9, 3.5:1	30
3	Ir(dFppy)$_3$	KF (3 equiv)	6:1 MeCN:H_2O	390 nm	9, 3.5:1	27
4	Ir(ppy)$_3$	Sc(OTf)$_3$	6:1 MeCN:H_2O	390 nm	trace	4
5	Ir(dFppy)$_3$	$B(C_6F_5)_3$	MeCN	390 nm	trace	trace
6	Ir(dFppy)$_3$	$LiClO_4$	MeCN	390 nm	trace	5
7	Ir(dFppy)$_3$	BF_3•OEt_2	MeCN	390 nm	0	trace
8	Ir(dFppy)$_3$	Cs Triazole	MeCN	390 nm	7, 2.5:1	42
9	PTH	KF	MeCN	390 nm	4, 3.0:1	26
10	Ir(ppy)$_3$	KF	HFIP	390 nm	0	0
11	Ir(ppy)$_3$	KF	NMP	390 nm	0	17
12	Ir(ppy)$_3$	KF	DCE	390 nm	4, 1:1	13
13	Ir(ppy)$_3$	KF	DMSO	390 nm	12, 2.0:1	34
14	Ir(ppy)$_3$	KF	6:1 MeCN:H_2O	427 nm	9, 3.5:1	22

LCAP% = area percent of UPLC UV trace at 254 nm, omitting photocatalyst. PTH standards for 10-phenylphenothiazine. See ref. 4 for entry 1 conditions.

Table S5. Additive Screening of 5d and 6d Cross-Coupling with $Ir(ppy)_3$

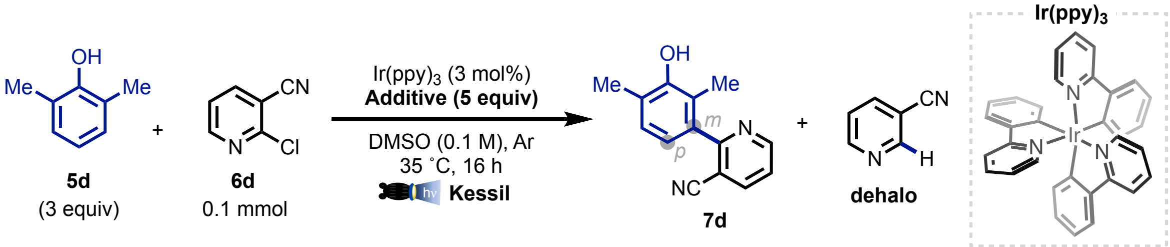

Entry	Additive	Kessil	LCAP% 7d, m:p	LCAP% Dehalo
1	KF	390 nm	10, 2.3:1	34
2	KOAc	427 nm	7, 1.3:1	27
3	LiI	427 nm	3, >20:1	5
4	$NaBF_4$	427 nm	18, 3.5:1	22
5	NaCl	427 nm	18, 3.5:1	26
6	Na_2SO_4	427 nm	19, 3.8:1	24
7	$Na_2B_4O_7$	427 nm	12, 3.0:1	28
8	NaBr	427 nm	5, 4.0:1	27
9	NaF	427 nm	16, 3.0:1<br>[40, 2.3:1]	28
10	$NaBPh_4$	427 nm	13, 1.8:1	18
11	NaOTf (3 equiv)	427 nm	20, 3.0:1	20
12	TFA	427 nm	14, 2.5:1	21
13	TfOH	427 nm	14, 3.7:1	12
14	B(O*i*-Pr)$_3$ (3 equiv)	427 nm	9, 2.0:1	30
15	$(TMS)_3SiH$	427 nm	3, 2.8:1	68

LCAP% = area percent of UPLC UV trace at 254 nm, omitting photocatalyst. Yield in parentheses corresponds to yield after isolation by column chromatography where m:p ratio determined by ^{1}H-NMR.

4. Synthetic Procedures for Starting Materials

All other starting materials not mentioned in this synthesis section were purchased from commercially available vendors in the highest purity (>95%) and used without further purification.

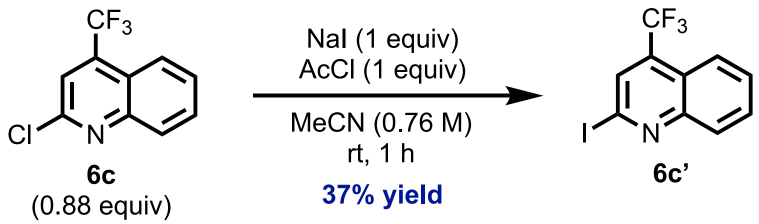

2-Iodo-4-(trifluoromethyl)quinoline (**6c’**)

To an 8 mL scintillation vial equipped with a magnetic stir bar was charged 2-chloro-4-(trifluoromethyl)quinoline (**6c**, 156 mg, 0.88 equiv, 0.67 mmol), sodium iodide (114 mg, 1.0 equiv, 0.76 mmol), and MeCN (1.0 mL, 0.76 M). To this stirred solution was then added acetyl chloride (54 µL, 1.0 equiv, 0.76 mmol) and the reaction mixture was stirred at room temperature for 1 h. LCMS analysis of the reaction mixture after 1 h indicated almost full conversion of the starting material to the desired product mass. The reaction was quenched with satd aq sodium thiosulfate and extracted with EtOAc (3x). The combined organic layers were dry loaded onto Celite™ and purified by reverse-phase column chromatography (90% MeCN:H_2O-0.1% FA) to afford the title compound **6c’** as an amorphous off-white solid (81 mg, 0.25 mmol, 37% yield).

^{1}H-NMR (400 MHz, acetone-*d$_6$*) δ 8.18 (s, 1H), 8.16 – 8.11 (m, 2H), 7.98 – 7.93 (m, 1H), 7.89 – 7.83 (m, 1H).

^{13}C{^{1}H}-NMR (101 MHz, acetone-*d$_6$*) δ 150.9, 134.9 (q, J = 31.8 Hz), 132.3, 130.5, 130.1, 129.9 (q, J = 5.6 Hz), 124.9 (q, J = 2.3 Hz), 123.3 (q, J = 274.0 Hz), 122.5 (q, J = 1.1 Hz), 117.6.

^{19}F{^{1}H}-NMR (376 MHz, acetone-*d$_6$*) δ –62.1.

HRMS (ESI-FTICR) *m/z* = 323.9492 calcd for $C_{10}H_6F_3IN^+$ $[M+H]^+$, found 323.9493.

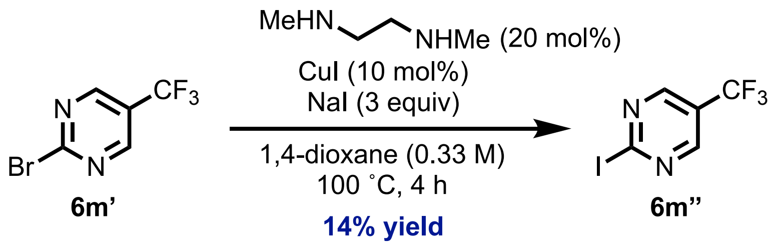

2-Iodo-5-(trifluoromethyl)pyrimidine (**6m’’**)

To a 20 mL microwave vial equipped with a magnetic stir bar was charged 2-bromo-5-(trifluoromethyl)pyrimidine (**6m’**, 380 mg, 1.0 equiv, 1.7 mmol), sodium iodide (753 mg, 3.0 equiv, 5.0 mmol), copper(I) iodide (32 mg, 10 mol%, 0.17 mmol), dry 1,4-dioxane (5.0 mL, 0.33 M), and then *N,N'*-dimethylethylenediamine (36 µL, 20 mol%, 0.34 mmol). The vial was crimp-capped and sparged with argon for 5 min prior to heating to 100 ˚C for 4 h with a heating block. LCMS analysis of the reaction mixture after 4 h indicated >80% conversion of the starting material. The reaction mixture was cooled to room temperature, quenched with H_2O, and extracted with EtOAc (3x). The combined organic layers were dry loaded onto silica and purified by normal-phase column chromatography (25% EtOAc:*n*-hexanes) to afford the title compound **6m’’** as an amorphous off-white solid (65 mg, 0.24 mmol, 14% yield).

^{1}H-NMR (400 MHz, $CDCl_3$) δ 8.69 (s, 2H).

Spectral data were in agreement with those reported.[5]

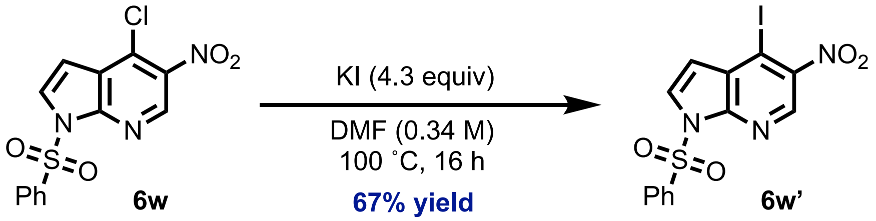

4-Iodo-5-nitro-1-(phenylsulfonyl)-1H-pyrrolo[2,3-b]pyridine (**6w'**)

To a 20 mL scintillation vial equipped with a magnetic stir bar was charged 4-chloro-5-nitro-1-(phenylsulfonyl)-1*H*-pyrrolo[2,3-*b*]pyridine (**6w**, 637 mg, 1.89 mmol, 1.0 equiv), potassium iodide (1.35 g, 8.13 mmol, 4.31 equiv), and dry DMF (5.5 mL, 0.34 M). The vial was capped and sparged with argon for 5 min prior to heating at 100 ˚C with a heating block. After 16 h, the reaction mixture was cooled to room temperature and diluted with H_2O to precipitate a solid that was collected by vacuum filtration (washed with H_2O three times) to afford the title compound **6w'** as an amorphous light yellow solid (542 mg, 1.26 mmol, 67% yield).

^{1}H-NMR (400 MHz, $CDCl_3$) δ 8.88 (s, 1H), 8.23 – 8.17 (m, 2H), 7.97 (d, *J* = 4.1 Hz, 1H), 7.69 – 7.60 (m, 1H), 7.57 – 7.50 (m, 2H), 6.72 (d, *J* = 4.1 Hz, 1H).

Spectral data were in agreement with those reported.[6]

5. General Procedures for Photochemical Cross-Coupling

[**WARNING**: Reactions with blue light require the use of special eye protection to prevent damage to vision. Special care must be taken, preferably blue light glasses.]

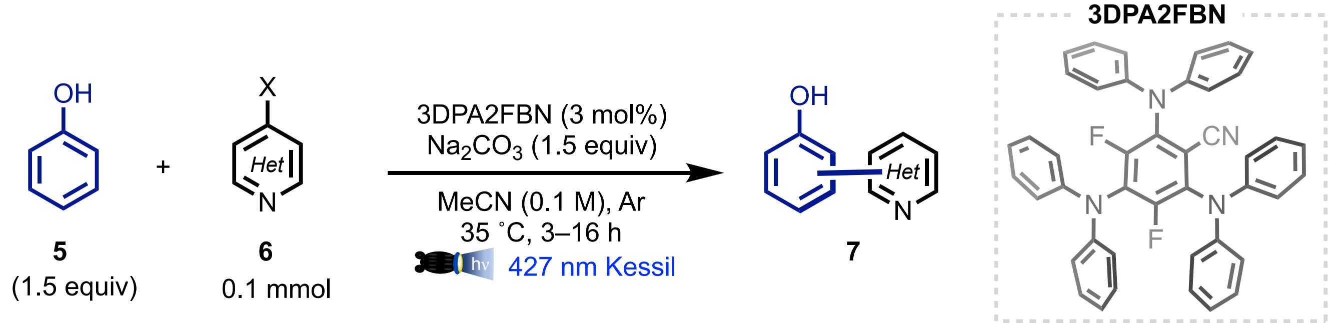

General Procedure B: Substrate Scope with 3DPA2FBN

An 8 mL microwave vial equipped with a magnetic stir bar was charged with phenol derivative (**5**, 0.15 mmol, 1.5 equiv), pyridine derivative (**6**, 0.10 mmol, 1.0 equiv), 3DPA2FBN (0.0030 mmol, 3.0 mol%), Na_2CO_3 (0.15 mmol, 1.5 equiv), and dry MeCN (1.0 mL, 0.10 M). The vial was crimp-capped and sparged by bubbling argon through the solvent via a needle for 5 min prior to irradiation with a 427 nm Kessil lamp (100% intensity, max 45 W, 7 cm from wall of flask) and commercial fan cooling (~35 ˚C) until reaction completion (see **Figure S2** for setup). After the desired reaction time, the reaction mixture was cooled to room temperature, opened to air, and analyzed by LCMS. The reaction mixture was dry loaded onto loaded onto Celite™ (reverse-phase) or silica (normal-phase) for purification by column chromatography to afford pure (>95%) cross-coupled products as determined by ^{1}H-, ^{13}C{^{1}H}-, and ^{19}F{^{1}H}-NMR (if necessary) spectroscopy.

Note: 1.0 M aqueous Na_2CO_3 can also be used for more facile base addition.

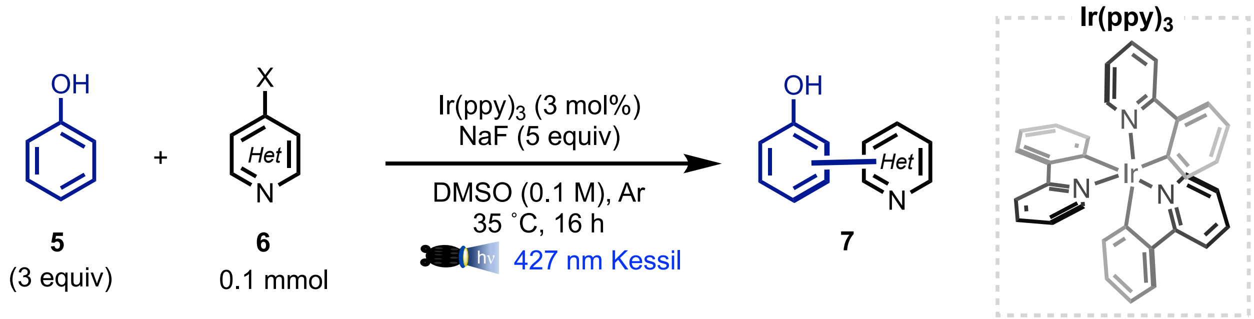

General Procedure C: Substrate Scope with Ir(ppy)₃

An 8 mL microwave vial equipped with a magnetic stir bar was charged with phenol derivative (**5**, 0.30 mmol, 3.0 equiv), pyridine derivative (**6**, 0.10 mmol, 1.0 equiv), $Ir(ppy)_3$ (0.0030 mmol, 3.0 mol%), NaF (0.50 mmol, 5.0 equiv), and dry DMSO (1.0 mL, 0.10 M). The vial was crimp-capped and sparged by bubbling argon through the solvent via a needle for 5 min prior to irradiation with a 427 nm Kessil lamp (100% intensity, max 45 W, 7 cm from wall of flask) and commercial fan cooling (~35 ˚C) for 16 h (see **Figure S2** for setup). After 16 h, the reaction mixture was cooled to room temperature, opened to air, and analyzed by LCMS. The reaction mixture was dry loaded onto loaded onto Celite™ (reverse-phase) or silica (normal-phase) for purification by column chromatography to afford pure (>95%) cross-coupled products as determined by ^{1}H-, ^{13}C{^{1}H}-, and ^{19}F{^{1}H}-NMR (if necessary) spectroscopy.

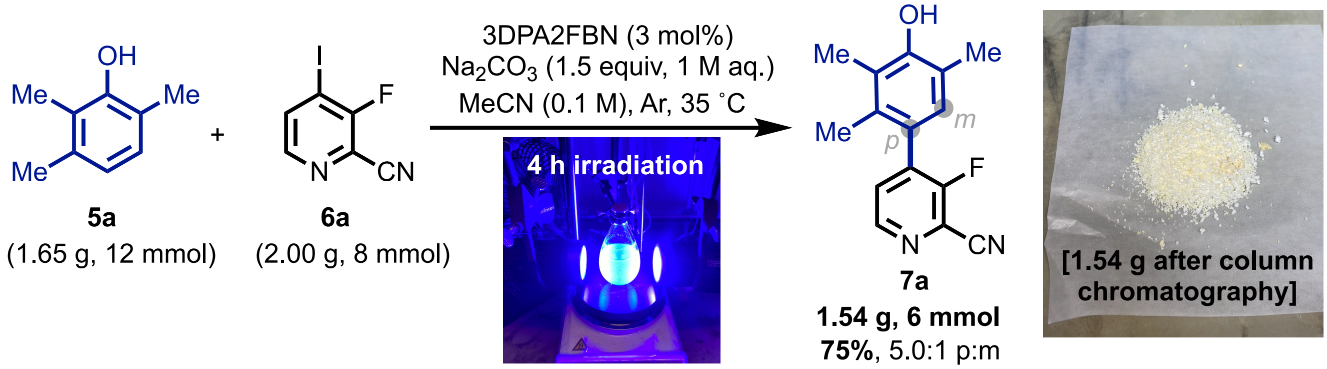

General Procedure D: Large-Scale Coupling with 3DPA2FBN

To a 100 mL pear-shaped flask equipped with a magnetic stir bar was charged 2,3,6-trimethylphenol (**5a**, 12 mmol, 1.5 equiv), 3-fluoro-4-iodopicolinonitrile (**6a**, 8.1 mmol, 1.0 equiv), 3DPA2FBN (0.24 mmol, 3.0 mol%), MeCN (81 mL, 0.10 M), and 1.0 M aqueous Na_2CO_3 (12 mmol, 1.5 equiv). The flask was capped with a septum and sparged with argon for 10 min prior to irradiation with two 440 nm Kessil lamps (40W, 100% intensity, 5 cm away) with commercial fan cooling (~35 ˚C) for 4 h. After 4 h, the reaction mixture was cooled to room temperature, opened to air, and analyzed by LCMS. The reaction mixture was dry loaded onto silica and purified by normal-phase column chromatography (15% EtOAc:*n*-hexanes) to afford an inseparable mixture of the title compounds **7a** and **7a'** as an amorphous slight yellow solid. The obtained material was analyzed by $^{19}F\{^{1}H\}$-NMR to determine the *para*- to *meta*-isomer ratio. See **Figure S3** below for the reaction workflow.

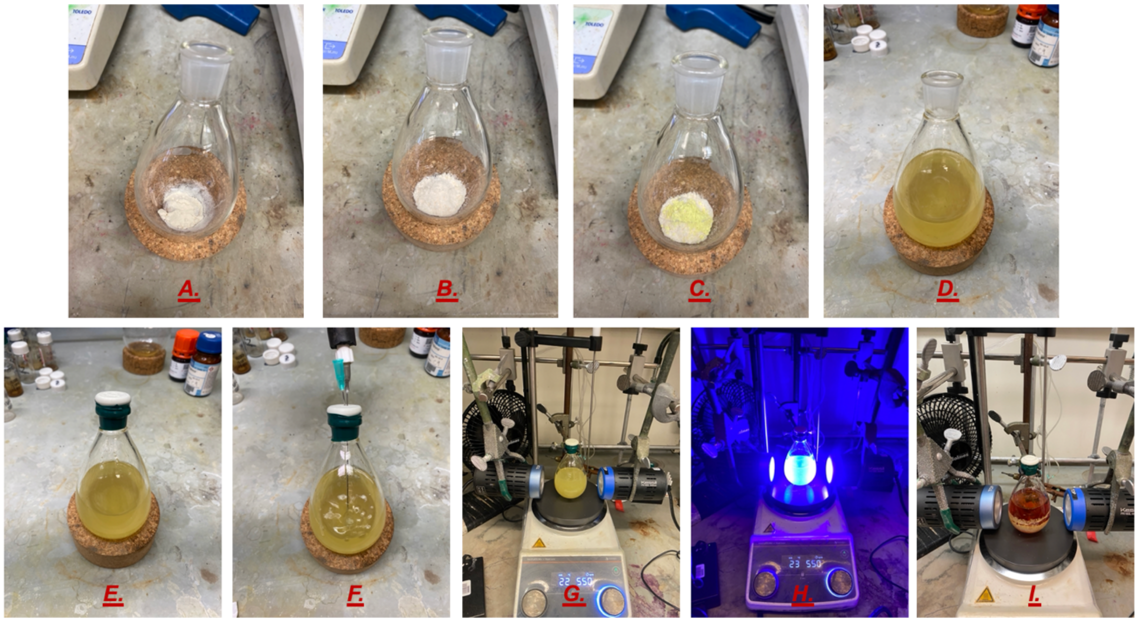

Figure S3. Reaction workflow for large-scale. a) addition of **5a** b) addition of **6a** c) addition of 3DPA2FBN d) addition of MeCN and Na_2CO_3 e) addition of septum and tape f) sparging with argon g) setup with stir plate and Kessils h) irradiation with Kessils for 4 h i) post-reaction mixture.

6. Product Characterization for Photochemical Cross-Coupling

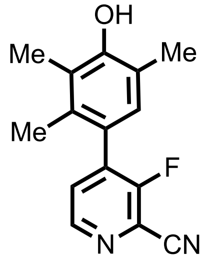

3-Fluoro-4-(4-hydroxy-2,3,5-trimethylphenyl)picolinonitrile (**7a**)

General procedure B was followed using 2,3,6-trimethylphenol (**5a**, 20 mg, 1.5 equiv), 3-fluoro-4-iodopicolinonitrile (**6a**, 25 mg, 1.0 equiv), 3DPA2FBN (1.9 mg, 3.0 mol%), Na_2CO_3 (16 mg, 1.5 equiv), and MeCN (1.0 mL, 0.10 M) for 3 h. Purification by normal-phase column chromatography (30% EtOAc:*n*-hexanes) afforded an inseparable mixture of the title compounds **7a** and **7a'** (3.2:1 **7a**:**7a'** by $^{19}F\{^1H\}$-NMR) as an amorphous light yellow solid (23 mg, 0.091 mmol, 91% yield).

General procedure C afforded **7a** and **7a'** in 49% yield with 3.0:1 **7a**:**7a'** by $^{19}F\{^1H\}$-NMR.

General procedure D afforded **7a** and **7a'** in 75% yield (1.54 g) with 5.0:1 **7a**:**7a'** by $^{19}F\{^1H\}$-NMR.

^{1}H-NMR (400 MHz, $CDCl_3$) δ 8.53 (d, *J* = 4.7 Hz, 1H), 7.46 – 7.42 (m, 1H), 6.82 (s, 1H), 4.92 (s, 1H), 2.27 (s, 3H), 2.25 (s, 3H), 2.08 (d, *J* = 1.5 Hz, 3H).

$^{13}C\{^1H\}$-NMR (101 MHz, $CDCl_3$) δ 159.0 (d, *J* = 268.1 Hz), 153.3, 146.7 (d, *J* = 5.8 Hz), 140.0 (d, *J* = 14.2 Hz), 133.8, 130.3 (d, *J* = 1.9 Hz), 128.9, 123.2, 123.0, 122.9, 120.9, 113.4 (d, *J* = 4.4 Hz), 17.4 (d, *J* = 3.6 Hz), 15.8, 12.2.

$^{19}F\{^1H\}$-NMR (376 MHz, $CDCl_3$) δ –117.97.

HRMS (ESI-FTICR) *m/z* = 255.0939 calcd for $C_{15}H_{12}FN_2O^-$ $[M–H]^-$, found 255.0941.

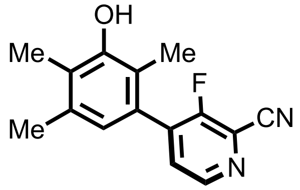

3-Fluoro-4-(3-hydroxy-2,4,5-trimethylphenyl)picolinonitrile (**7a'**)

See above procedure for details.

^{1}H-NMR (400 MHz, $CDCl_3$) δ 8.55 (d, *J* = 4.8 Hz, 1H), 7.49 – 7.46 (m, 1H), 6.62 (s, 1H), 4.92 (s, 1H), 2.30 (s, 3H), 2.24 (s, 3H), 2.06 (d, *J* = 1.5 Hz, 3H).

$^{13}C\{^1H\}$-NMR (101 MHz, $CDCl_3$) δ 158.9 (d, *J* = 269.2 Hz), 152.4, 146.8 (d, *J* = 5.4 Hz), 139.2 (d, *J* = 14.2 Hz), 135.8, 130.1 (d, *J* = 1.8 Hz), 129.0, 123.7, 123.0, 122.8, 119.2, 113.3 (d, *J* = 4.4 Hz), 20.0, 13.3 (d, *J* = 3.3 Hz), 12.0.

$^{19}F\{^1H\}$-NMR (376 MHz, $CDCl_3$) δ –118.09.

HRMS (ESI-FTICR) *m/z* = 257.1085 calcd for $C_{15}H_{14}FN_2O^+$ $[M+H]^+$, found 257.1078.

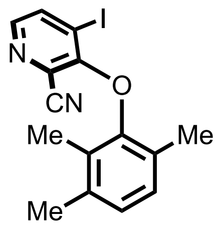

4-Iodo-3-(2,3,6-trimethylphenoxy)picolinonitrile (**7aa**)

General procedure B was followed using 2,3,6-trimethylphenol (**5a**, 20 mg, 1.5 equiv), 3-fluoro-4-iodopicolinonitrile (**6a**, 25 mg, 1.0 equiv), 3DPA2FBN (1.9 mg, 3.0 mol%), Na_2CO_3 (16 mg, 1.5 equiv), and MeCN (1.0 mL, 0.10 M). However, the reaction mixture was instead heated to 80 ˚C for 48 h with a heating block instead of irradiation. Purification by reverse-phase column chromatography (90% MeCN:H_2O-0.1% FA) afforded the title compound **7aa** as an amorphous off-white solid (16 mg, 0.044 mmol, 44% yield).

^{1}H-NMR (400 MHz, $CDCl_3$) δ 8.05 (d, *J* = 4.8 Hz, 1H), 7.97 (d, *J* = 4.8 Hz, 1H), 7.07 (d, *J* = 7.7 Hz, 1H), 7.00 (d, *J* = 7.7 Hz, 1H), 2.29 (s, 3H), 2.09 (s, 6H).

^{13}C{^{1}H}-NMR (101 MHz, $CDCl_3$) δ 156.1, 151.3, 144.4, 138.9, 136.4, 129.4, 128.4, 128.2, 127.5, 119.8, 112.6, 99.7, 20.0, 16.6, 13.0.

HRMS (ESI-FTICR) *m/z* = 365.0145 calcd for $C_{15}H_{14}IN_2O^+$ [M+H]$^+$, found 365.0146.

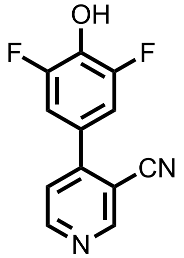

4-(3,5-Difluoro-4-hydroxyphenyl)nicotinonitrile (**7b**)

General procedure B was followed using 2,6-difluorophenol (**5b**, 20 mg, 1.5 equiv), 4-bromonicotinonitrile (**6b**, 18 mg, 1.0 equiv), 3DPA2FBN (1.9 mg, 3.0 mol%), Na_2CO_3 (16 mg, 1.5 equiv), and MeCN (1.0 mL, 0.10 M) for 3 h. Purification by normal-phase column chromatography (100% EtOAc:*n*-hexanes) afforded the title compound **7b** as a yellow oil (14 mg, 0.060 mmol, 60% yield).

General procedure C afforded **7b** in 0% yield.

^{1}H-NMR (600 MHz, DMSO-*d*$_6$) δ 10.95 (s, 1H), 9.08 (s, 1H), 8.86 (d, *J* = 5.3 Hz, 1H), 7.72 (d, *J* = 5.3 Hz, 1H), 7.52 – 7.48 (m, 2H).

^{13}C{^{1}H}-NMR (101 MHz, DMSO-*d*$_6$) δ 153.8, 153.2, 152.1 (dd, *J* = 243.1, 7.6 Hz), 149.0, 135.7 (t, *J* = 15.8 Hz), 125.1 (t, *J* = 8.7 Hz), 123.8, 116.9, 112.7 (dd, *J* = 17.4, 6.5 Hz), 107.5.

^{19}F{^{1}H}-NMR (376 MHz, DMSO-*d*$_6$) δ –131.2.

HRMS (ESI-FTICR) *m/z* = 233.05209 calcd for $C_{12}H_7F_2N_2O^+$ [M+H]$^+$, found 233.05213.

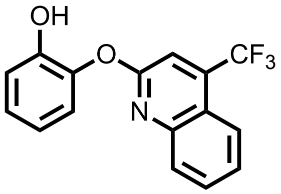

2-((4-(Trifluoromethyl)quinolin-2-yl)oxy)phenol (**7c**)

General procedure B was followed using catechol (**5c**, 17 mg, 1.5 equiv), 2-chloro-4-(trifluoromethyl)quinoline (**6c**, 23 mg, 1.0 equiv), 3DPA2FBN (1.9 mg, 3.0 mol%), Na_2CO_3 (16 mg, 1.5 equiv), and MeCN (1.0 mL, 0.10 M) for 3 h. Purification by reverse-phase column chromatography (70% MeCN:H_2O-0.1% FA) afforded the title compound **7c** as an amorphous off-white solid (15 mg, 0.049 mmol, 49% yield).

General procedure C afforded **7c** in 20% yield.

^{1}H-NMR (600 MHz, methanol-*d$_4$*) δ 8.05 (d, J = 8.4 Hz, 1H), 7.80 (d, J = 8.4 Hz, 1H), 7.74 – 7.70 (m, 1H), 7.59 – 7.55 (m, 1H), 7.53 (s, 1H), 7.19 – 7.12 (m, 2H), 6.99 (dd, J = 8.1, 1.4 Hz, 1H), 6.92 (td, J = 7.7, 1.4 Hz, 1H).

^{13}C{^{1}H}-NMR (151 MHz, methanol-*d$_4$*) δ 162.2, 150.6, 148.7, 142.1, 138.8 (q, J = 31.9 Hz), 132.0, 129.3, 127.7, 127.3, 124.8 (q, J = 2.5 Hz), 124.6 (q, J = 274.7 Hz), 124.3, 121.4, 121.1, 118.3, 112.1 (q, J = 5.8 Hz).

^{19}F{^{1}H}-NMR (376 MHz, methanol-*d$_4$*) δ –63.4.

HRMS (ESI-FTICR) *m/z* = 306.07364 calcd for $C_{16}H_{11}F_3NO_2^+$ [M+H]$^+$, found 306.07366.

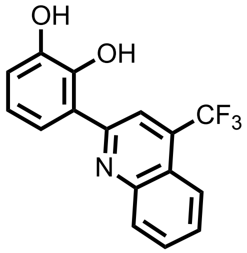

3-(4-(Trifluoromethyl)quinolin-2-yl)benzene-1,2-diol (**7c'**)

General procedure B was followed using catechol (**5c**, 17 mg, 1.5 equiv), 2-iodo-4-(trifluoromethyl)quinoline (**6c'**, 32 mg, 1.0 equiv), 3DPA2FBN (1.9 mg, 3.0 mol%), 1.0 M aqueous Na_2CO_3 (150 µL, 1.5 equiv), and MeCN (1.0 mL, 0.10 M) for 3 h. Purification by reverse-phase column chromatography (70% MeCN:H_2O-0.1% FA) afforded the title compounds **7c'** as an amorphous beige solid (10.5 mg, 0.034 mmol, 34% yield) and **7c''** as a yellow oil (5.0 mg, 0.016 mmol, 16% yield).

General procedure C afforded **7c'** and **7c''** in 0% yield.

^{1}H-NMR (400 MHz, acetone-*d$_6$*) δ 14.53 (s, 1H), 8.61 (s, 1H), 8.27 (d, J = 8.5 Hz, 1H), 8.20 (d, J = 8.5 Hz, 1H), 8.01 (t, J = 7.2 Hz, 1H), 7.86 (t, J = 7.3 Hz, 1H), 7.77 (dd, J = 8.4, 1.3 Hz, 1H), 7.65 (s, 1H), 7.03 (dd, J = 8.1, 1.4 Hz, 1H), 6.91 (t, J = 8.0 Hz, 1H).

^{13}C{^{1}H}-NMR (151 MHz, acetone-*d$_6$*) δ 159.0, 149.7, 147.7, 146.6, 136.3 (q, J = 32.3 Hz), 132.6, 129.6, 129.3, 124.4 (q, J = 274.7 Hz), 123.8 (q, J = 3.0 Hz), 121.9, 119.9, 119.2, 119.1, 118.6, 116.7 (q, J = 5.6 Hz).

^{19}F{^{1}H}-NMR (376 MHz, acetone-*d$_6$*) δ –62.17.

HRMS (ESI-FTICR) *m/z* = 306.07364 calcd for $C_{16}H_{11}F_3NO_2^+$ [M+H]$^+$, found 306.07361.

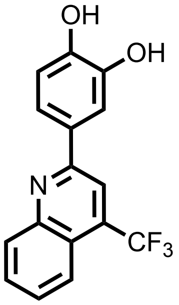

4-(4-(Trifluoromethyl)quinolin-2-yl)benzene-1,2-diol (**7c''**)
See above procedure for details.
^{1}H-NMR (600 MHz, acetone-*d$_6$*) δ 8.31 (s, 1H), 8.19 (d, *J* = 8.5 Hz, 1H), 8.14 – 8.08 (m, 1H), 7.96 (d, *J* = 2.2 Hz, 1H), 7.92 – 7.87 (m, 1H), 7.76 (dd, *J* = 8.3, 2.2 Hz, 1H), 7.75 – 7.71 (m, 1H), 7.01 (d, *J* = 8.3 Hz, 1H).
Note: Phenolic protons not observed.
^{13}C{^{1}H}-NMR (151 MHz, acetone-*d$_6$*) δ 157.1, 149.9, 148.7, 146.5, 134.9 (q, *J* = 32.3 Hz), 131.4, 131.2, 131.0, 128.5, 124.8 (q, *J* = 273.6 Hz), 124.4 (q, *J* = 2.2 Hz), 121.9, 120.7, 116.4, 116.3 (q, *J* = 5.4 Hz), 115.2.
^{19}F{^{1}H}-NMR (376 MHz, acetone-*d$_6$*) δ –62.11.
HRMS (ESI-FTICR) *m/z* = 306.07364 calcd for $C_{16}H_{11}F_3NO_2^+$ [M+H]$^+$, found 306.07359.

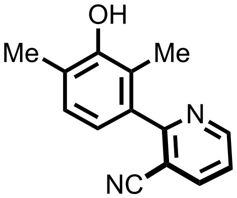

2-(3-Hydroxy-2,4-dimethylphenyl)nicotinonitrile (**7d**)
General procedure C was followed using 2,6-dimethylphenol (**5d**, 37 mg, 3.0 equiv), 2-chloronicotinonitrile (**6d**, 14 mg, 1.0 equiv), Ir(ppy)$_3$ (2.0 mg, 3.0 mol%), NaF (21 mg, 5.0 equiv), and DMSO (1.0 mL, 0.10 M) for 16 h. Purification by reverse-phase column chromatography (50% MeCN:H_2O-0.1% FA) afforded the title compounds **7d** as an amorphous beige solid (6.5 mg, 0.029 mmol, 29% yield) and **7d'** as an amorphous light brown solid (2.5 mg, 0.011 mmol, 11% yield).
General procedure B afforded **7d** and **7d'** in 0% yield.
^{1}H-NMR (600 MHz, CDCl$_3$) δ 8.87 (dd, *J* = 4.9, 1.7 Hz, 1H), 8.06 (dd, *J* = 7.9, 1.7 Hz, 1H), 7.40 (dd, *J* = 7.9, 4.9 Hz, 1H), 7.10 (d, *J* = 7.7 Hz, 1H), 6.88 (d, *J* = 7.7 Hz, 1H), 4.86 (s, 1H), 2.31 (s, 3H), 2.13 (s, 3H).
^{13}C{^{1}H}-NMR (151 MHz, CDCl$_3$) δ 163.1, 152.6, 152.2, 140.7, 136.4, 128.3, 128.1, 124.7, 121.6, 121.5, 116.9, 110.2, 16.2, 12.9.
HRMS (ESI-FTICR) *m/z* = 225.10224 calcd for $C_{14}H_{13}N_2O^+$ [M+H]$^+$, found 225.10220.

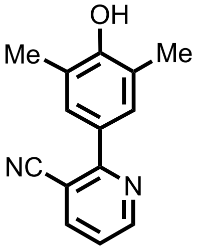

2-(4-Hydroxy-3,5-dimethylphenyl)nicotinonitrile (**7d'**)
See above procedure for details.
^{1}H-NMR (600 MHz, CDCl$_3$) δ 8.82 (dd, *J* = 4.8, 1.7 Hz, 1H), 8.03 (dd, *J* = 7.8, 1.7 Hz, 1H), 7.61 (s, 2H), 7.30 (dd, *J* = 7.8, 4.8 Hz, 1H), 4.92 (s, 1H), 2.34 (s, 6H).
^{13}C{^{1}H}-NMR (151 MHz, CDCl$_3$) δ 160.9, 154.3, 152.5, 142.0, 129.4, 129.3, 123.4, 120.8, 118.1, 106.8, 16.0.
HRMS (ESI-FTICR) *m/z* = 225.102239 calcd for $C_{14}H_{13}N_2O^+$ [M+H]$^+$, found 225.102236.

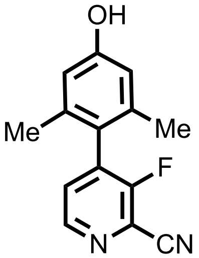

3-Fluoro-4-(4-hydroxy-2,6-dimethylphenyl)picolinonitrile (**7d''**)
General procedure B was followed using 3,5-dimethylphenol (**5d'**, 55 mg, 1.5 equiv), 3-fluoro-4-iodopicolinonitrile (**6a**, 74 mg, 1.0 equiv), 3DPA2FBN (5.8 mg, 3.0 mol%), 1.0 M aqueous Na_2CO_3 (450 µL, 1.5 equiv), and MeCN (3.0 mL, 0.10 M) for 2 h. Purification by reverse-phase column chromatography (50% MeCN:H_2O-0.1% FA) afforded the title compounds **7d''** as an amorphous beige solid (20.7 mg, 0.085 mmol, 29% yield) and **7d'''** as an amorphous yellow solid (5.8 mg, 0.024 mmol, 8% yield).
^{1}H-NMR (400 MHz, CDCl$_3$) δ 8.57 (d, *J* = 4.7 Hz, 1H), 7.38 (dd, *J* = 5.7, 4.7 Hz, 1H), 6.65 (s, 2H), 4.82 (s, 1H), 2.00 (s, 6H).
^{13}C{^{1}H}-NMR (151 MHz, CDCl$_3$) δ 159.3 (d, *J* = 267.5 Hz), 156.3, 147.1 (d, *J* = 5.7 Hz), 138.5 (d, *J* = 15.3 Hz), 137.9, 131.1, 123.6, 123.1, 115.0, 113.4, 20.6.
^{19}F{^{1}H}-NMR (376 MHz, CDCl$_3$) δ –117.8.
HRMS (ESI-Orbitrap) *m/z* = 243.0928 calcd for $C_{14}H_{12}FN_2O^+$ [M+H]$^+$, found 243.0925.

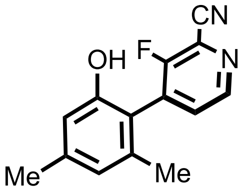

3-Fluoro-4-(2-hydroxy-4,6-dimethylphenyl)picolinonitrile (**7d'''**)
See above procedure for details.
^{1}H-NMR (400 MHz, CDCl$_3$) δ 8.54 (d, *J* = 4.8 Hz, 1H), 7.52 – 7.48 (m, 1H), 6.75 (s, 1H), 6.59 (s, 1H), 5.13 (s, 1H), 2.33 (s, 3H), 2.07 (s, 3H).
^{13}C{^{1}H}-NMR (151 MHz, CDCl$_3$) δ 159.6 (d, *J* = 268.7 Hz), 152.7, 146.7 (d, *J* = 5.5 Hz), 141.3, 137.7, 135.5 (d, *J* = 14.7 Hz), 131.6 (d, *J* = 2.0 Hz), 124.0, 123.2 (d, *J* = 17.4 Hz), 115.3, 114.1, 113.4 (d, *J* = 4.7 Hz), 21.4, 19.9.
^{19}F{^{1}H}-NMR (376 MHz, CDCl$_3$) δ –116.5.
HRMS (ESI-Orbitrap) *m/z* = 243.0928 calcd for $C_{14}H_{12}FN_2O^+$ $[M+H]^+$, found 243.0923.

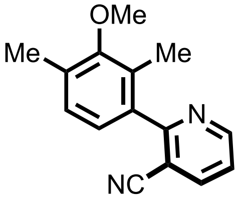

2-(3-Methoxy-2,4-dimethylphenyl)nicotinonitrile (**7e**)
General procedure C was followed using 2,6-dimethylanisole (**5e**, 41 mg, 3.0 equiv), 2-chloronicotinonitrile (**6d**, 14 mg, 1.0 equiv), Ir(ppy)$_3$ (2.0 mg, 3.0 mol%), NaF (21 mg, 5.0 equiv), and DMSO (1.0 mL, 0.10 M) for 16 h. Purification by reverse-phase column chromatography (65% MeCN:H_2O-0.1% FA) afforded the title compounds **7e** as an amorphous yellow solid (2.7 mg, 0.011 mmol, 11% yield) and **7e'** as an amorphous yellow solid (0.7 mg, 0.003 mmol, 3% yield).
General procedure B afforded **7e** and **7e'** in 0% yield.
^{1}H-NMR (600 MHz, CDCl$_3$) δ 8.86 (dd, *J* = 4.7, 1.6 Hz, 1H), 8.06 (dd, *J* = 8.0, 1.4 Hz, 1H), 7.39 (dd, *J* = 7.8, 4.9 Hz, 1H), 7.14 (d, *J* = 7.7 Hz, 1H), 7.04 (d, *J* = 7.7 Hz, 1H), 3.77 (s, 3H), 2.35 (s, 3H), 2.18 (s, 3H).
^{13}C{^{1}H}-NMR (151 MHz, CDCl$_3$) δ 163.0, 157.4, 152.2, 140.7, 136.7, 132.6, 129.5, 128.7, 124.9, 121.6, 116.9, 110.1, 59.9, 16.3, 13.2.
HRMS (ESI-FTICR) *m/z* = 239.11789 calcd for $C_{15}H_{15}N_2O^+$ $[M+H]^+$, found 239.11792.

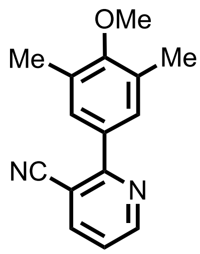

2-(4-Methoxy-3,5-dimethylphenyl)nicotinonitrile (**7e'**)
See above procedure for details.
^{1}H-NMR (600 MHz, $CDCl_3$) δ 8.84 (dd, *J* = 4.8, 1.7 Hz, 1H), 8.05 (dd, *J* = 7.9, 1.7 Hz, 1H), 7.59 (s, 2H), 7.34 (dd, *J* = 7.8, 4.8 Hz, 1H), 3.78 (s, 3H), 2.37 (s, 6H).
^{13}C{^{1}H}-NMR (151 MHz, $CDCl_3$) δ 160.9, 158.9, 152.5, 141.9, 132.6, 131.5, 129.5, 121.2, 117.9, 107.2, 59.8, 16.3.
HRMS (ESI-FTICR) *m/z* = 239.1179 calcd for $C_{15}H_{15}N_2O^+$ [M+H]$^+$, found 239.1178.

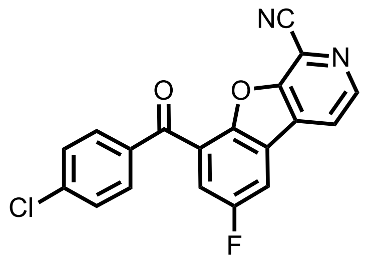

8-(4-Chlorobenzoyl)-6-fluorobenzofuro[2,3-c]pyridine-1-carbonitrile (**7l**)
General procedure B was followed using 4-chloro-5-fluoro-2-hydroxybenzophenone (**5n**, 25 mg, 1.0 equiv), 3-fluoro-4-iodopicolinonitrile (**6a**, 37 mg, 1.5 equiv), 1.0 M aqueous Na_2CO_3 (150 µL, 1.5 equiv), 3DPA2FBN (1.9 mg, 3.0 mol%), and MeCN (1.0 mL, 0.10 M) for 16 h. Purification by reverse-phase column chromatography (50% MeCN:H_2O-0.1% FA) followed by normal-phase column chromatography (50% EtOAc:*n*-hexanes) afforded the title compound **7l** as an amorphous white solid (6.4 mg, 0.018 mmol, 18% yield).
^{1}H-NMR (400 MHz, acetone-*d*$_6$) δ 8.80 (d, *J* = 5.0 Hz, 1H), 8.57 (d, *J* = 5.0 Hz, 1H), 8.45 (dd, *J* = 7.7, 2.7 Hz, 1H), 8.00 (d, *J* = 8.5 Hz, 2H), 7.86 (dd, *J* = 8.5, 2.2 Hz, 1H), 7.64 (dd, *J* = 8.5 Hz, 2H).
^{13}C{^{1}H}-NMR (151 MHz, acetone-*d*$_6$) δ 190.5, 160.0 (d, *J* = 243.8 Hz), 155.9, 151.2, 145.8, 140.7, 136.2, 133.1 (d, *J* = 3.3 Hz), 132.7, 129.9, 125.9 (d, *J* = 7.8 Hz), 125.1 (d, *J* = 11.0 Hz), 121.5, 120.3 (d, *J* = 25.9 Hz), 118.6, 114.5, 113.8 (d, *J* = 25.9 Hz).
^{19}F{^{1}H}-NMR (376 MHz, acetone-*d*$_6$) δ –117.9.
HRMS (ESI-FTICR) *m/z* = 351.0331 calcd for $C_{19}H_9ClFN_2O_2^+$ [M+H]$^+$, found 351.0329.

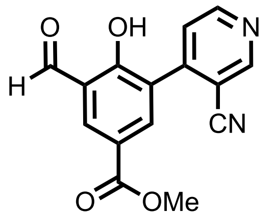

Methyl 3-(3-cyanopyridin-4-yl)-5-formyl-4-hydroxybenzoate (**7m**)
General procedure B was followed using 3-formyl-4-hydroxybenzoate (**5l**, 18 mg, 1.0 equiv), 4-bromonicotinonitrile (**6c**, 28 mg, 1.5 equiv), 3DPA2FBN (1.9 mg, 3.0 mol%), 1.0 M aqueous Na_2CO_3 (150 µL, 1.5 equiv), and MeCN (1.0 mL, 0.10 M) for 16 h. Purification by reverse-phase column chromatography (50% MeCN:H_2O-0.1% FA) followed by normal-phase column chromatography (50% EtOAc:*n*-hexanes) afforded the title compound **7m** as an amorphous white solid (9.6 mg, 0.034 mmol, 34% yield).
^{1}H-NMR (400 MHz, acetone-*d$_6$*) δ 10.71 (s, 1H), 9.50 (s, 1H), 9.19 (d, *J* = 2.2 Hz, 1H), 9.11 (d, *J* = 5.5 Hz, 1H), 8.64 (d, *J* = 2.0 Hz, 1H), 8.52 (d, *J* = 5.4 Hz, 1H), 4.00 (s, 3H).
^{13}C{^{1}H}-NMR (151 MHz, acetone-*d$_6$*) δ 187.4, 165.6, 158.7, 157.6, 156.0, 153.0, 141.2, 132.1, 131.6, 127.7, 125.6, 118.9, 117.4, 117.2, 53.1.
HRMS (ESI-FTICR) *m/z* = 283.0713 calcd for $C_{15}H_{11}N_2O_4^+$ [M+H]$^+$, found 283.0715.

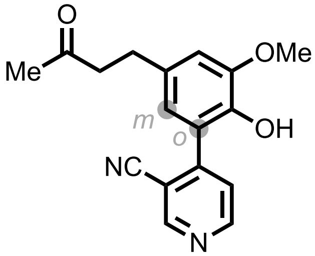

4-(2-Hydroxy-3-methoxy-5-(3-oxobutyl)phenyl)nicotinonitrile (**7n**)
General procedure B was followed using 4-(4-hydroxy-3-methoxyphenyl)butan-2-one (**5m**, 29 mg, 1.5 equiv), 4-bromonicotinonitrile (**6c**, 18 mg, 1.0 equiv), 3DPA2FBN (1.9 mg, 3.0 mol%), 1.0 M aqueous Na_2CO_3 (150 µL, 1.5 equiv), and MeCN (1.0 mL, 0.10 M) for 16 h. Purification by reverse-phase column chromatography (50% MeCN:H_2O-0.1% FA) afforded the title compound **7n** as an amorphous light tan solid (16 mg, 0.054 mmol, 54% yield, 5.6:1 o:m ratio by ^{1}H-NMR).
^{1}H-NMR (600 MHz, methanol-*d$_4$*) δ 8.95 (s, 1H), 8.78 (d, *J* = 5.1 Hz, 1H), 7.51 (d, *J* = 5.1 Hz, 1H), 6.96 (s, 1H), 6.66 (s, 1H), 3.92 (s, 3H), 2.71 – 2.63 (m, 4H), 2.03 (s, 3H).
Note: Presence of meta-isomer in 1:5.6 ratio by ^{1}H-NMR. Integrating 8.99 ppm singlet (meta) versus 8.95 ppm singlet (ortho).
^{13}C{^{1}H}-NMR (151 MHz, methanol-*d$_4$*) δ 210.4, 155.1, 154.0, 153.4, 150.3, 146.2, 131.3, 129.1, 127.0, 117.1, 117.1, 113.8, 112.5, 56.5, 45.5, 29.9, 27.4.
HRMS (ESI-FTICR) *m/z* = 297.1234 calcd for $C_{17}H_{17}N_2O_3^+$ [M+H]$^+$, found 297.1240.

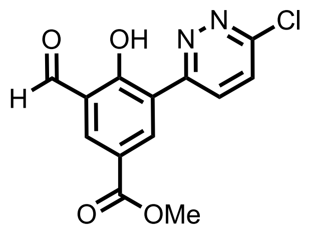

Methyl 3-(6-chloropyridazin-3-yl)-5-formyl-4-hydroxybenzoate (**7o**)

General procedure B was followed using methyl 3-formyl-4-hydroxybenzoate (**5l**, 27 mg, 1.5 equiv), 3-chloro-6-iodopyridazine (**6o**, 24 mg, 1.0 equiv), 3DPA2FBN (1.9 mg, 3.0 mol%), 1.0 M aqueous Na_2CO_3 (150 µL, 1.5 equiv), and MeCN (1.0 mL, 0.10 M) for 16 h. Purification by reverse-phase column chromatography (65% MeCN:H_2O-0.1% FA) afforded the title compound **7o** as a brown oil (11 mg, 0.038 mmol, 38% yield).

^{1}H-NMR (600 MHz, acetone-*d*$_6$) δ 10.44 (s, 1H), 8.88 (d, *J* = 1.8 Hz, 1H), 8.67 (d, *J* = 9.1 Hz, 1H), 8.53 (d, *J* = 1.7 Hz, 1H), 8.07 (d, *J* = 9.0 Hz, 1H), 3.93 (s, 3H).

^{13}C{^{1}H}-NMR (151 MHz, acetone-*d*$_6$) δ 192.9, 165.9, 165.6, 158.8, 156.6, 136.6, 135.2, 130.8, 130.0, 124.5, 122.5, 121.7, 52.6.

HRMS (ESI-FTICR) *m/z* = 293.0323 calcd for $C_{13}H_{10}ClN_2O_4^+$ [M+H]$^+$, found 293.0324.

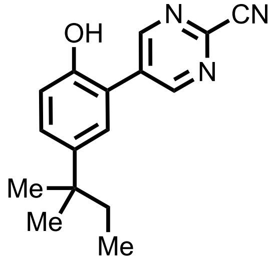

5-(2-Hydroxy-5-(tert-pentyl)phenyl)pyrimidine-2-carbonitrile (**7p**)

General procedure B was followed using 4-(*tert*-pentyl)phenol (**5f**, 25 mg, 1.5 equiv), 5-bromopyrimidine-2-carbonitrile (**6p**, 18 mg, 1.0 equiv), 3DPA2FBN (1.9 mg, 3.0 mol%), 1.0 M aqueous Na_2CO_3 (150 µL, 1.5 equiv), and MeCN (1.0 mL, 0.10 M) for 16 h. Purification by reverse-phase column chromatography (85% MeCN:H_2O-0.1% FA) afforded the title compound **7p** as an amorphous beige solid (19 mg, 0.071 mmol, 71% yield).

^{1}H-NMR (600 MHz, acetone-*d*$_6$) δ 9.23 (s, 2H), 7.51 (d, *J* = 2.4 Hz, 1H), 7.36 (dd, *J* = 8.5, 2.5 Hz, 1H), 7.04 (d, *J* = 8.5 Hz, 1H), 1.68 (q, *J* = 7.4 Hz, 2H), 1.30 (s, 6H), 0.70 (t, *J* = 7.4 Hz, 3H).

^{13}C{^{1}H}-NMR (151 MHz, acetone-*d*$_6$) δ 158.6, 153.4, 143.0, 142.5, 136.3, 130.0, 128.7, 120.0, 117.1, 116.9, 38.1, 37.3, 28.9, 9.4.

HRMS (ESI-FTICR) *m/z* = 268.1444 calcd for $C_{16}H_{18}N_3O^+$ [M+H]$^+$, found 268.1446.

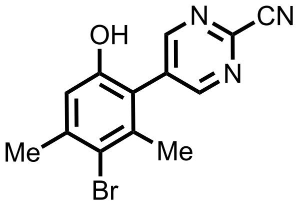

5-(3-Bromo-6-hydroxy-2,4-dimethylphenyl)pyrimidine-2-carbonitrile (**7q**)

General procedure B was followed using 4-bromo-3,5-dimethylphenol (**5g**, 30 mg, 1.5 equiv), 5-bromopyrimidine-2-carbonitrile (**6p**, 18 mg, 1.0 equiv), 3DPA2FBN (1.9 mg, 3.0 mol%), 1.0 M aqueous Na_2CO_3 (150 µL, 1.5 equiv), and MeCN (1.0 mL, 0.10 M) for 16 h. Purification by reverse-phase column chromatography (85% MeCN:H_2O-0.1% FA) afforded the title compound **7q** as a brown oil (16 mg, 0.053 mmol, 53% yield).

^{1}H-NMR (400 MHz, $CDCl_3$) δ 8.75 (s, 2H), 6.75 (s, 1H), 5.53 (s, 1H), 2.43 (s, 3H), 2.23 (s, 3H).

^{13}C{^{1}H}-NMR (101 MHz, $CDCl_3$) δ 159.3, 151.8, 143.2, 141.4, 137.4, 134.0, 119.8, 119.1, 115.7, 115.6, 24.4, 21.9.

HRMS (ESI-FTICR) *m/z* = 304.0080 calcd for $C_{13}H_{11}BrN_3O^+$ [M+H]$^+$, found 304.0079.

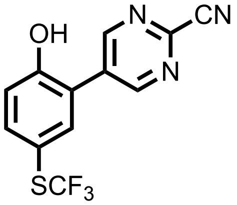

5-(2-Hydroxy-5-((trifluoromethyl)thio)phenyl)pyrimidine-2-carbonitrile (**7r**)

General procedure B was followed using 4-((trifluoromethyl)thio)phenol (**5i**, 19 mg, 1.0 equiv), 5-bromopyrimidine-2-carbonitrile (**6p**, 28 mg, 1.5 equiv), 3DPA2FBN (1.9 mg, 3.0 mol%), 1.0 M aqueous Na_2CO_3 (150 µL, 1.5 equiv), and MeCN (1.0 mL, 0.10 M) for 16 h. Purification by reverse-phase column chromatography (70% MeCN:H_2O-0.1% FA) afforded the title compounds **7r** as an amorphous beige solid (9.5 mg, 0.032 mmol, 32% yield) and **7r'** as an amorphous brown solid (15 mg, 0.037 mmol, 37% yield).

^{1}H-NMR (400 MHz, acetone-*d*$_6$) δ 10.12 (s, 1H), 9.26 (s, 2H), 7.93 (d, *J* = 2.3 Hz, 1H), 7.71 (dd, *J* = 8.5, 2.3 Hz, 1H), 7.25 (d, *J* = 8.5 Hz, 1H).

^{13}C{^{1}H}-NMR (151 MHz, acetone-*d*$_6$) δ 158.8, 158.6, 143.8, 140.7, 139.8, 134.3, 130.7 (q, *J* = 307.2 Hz), 122.6, 118.7, 117.0, 115.1 (q, *J* = 2.1 Hz).

^{19}F{^{1}H}-NMR (376 MHz, acetone-*d*$_6$) δ –44.77.

HRMS (ESI-FTICR) *m/z* = 298.0256 calcd for $C_{12}H_7F_3N_3OS^+$ [M+H]$^+$, found 298.0255.

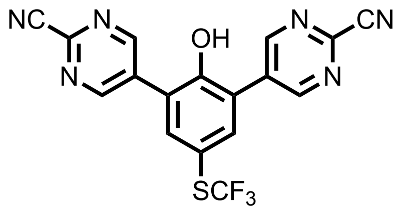

5,5'-(2-Hydroxy-5-((trifluoromethyl)thio)-1,3-phenylene)bis(pyrimidine-2-carbonitrile) (**7r'**)
See above procedure for details.
^{1}H-NMR (400 MHz, acetone-*d$_6$*) δ 9.26 (s, 4H), 8.00 (s, 2H).
^{13}C{^{1}H}-NMR (151 MHz, acetone-*d$_6$*) δ 159.3, 156.3, 144.3, 141.1, 134.2, 130.6 (q, *J* = 308.4 Hz), 125.5, 116.9, 116.2.
^{19}F{^{1}H}-NMR (376 MHz, acetone-*d$_6$*) δ –44.29.
HRMS (ESI-FTICR) *m/z* = 401.04269 calcd for $C_{17}H_8F_3N_6OS^+$ [M+H]$^+$, found 401.04274.

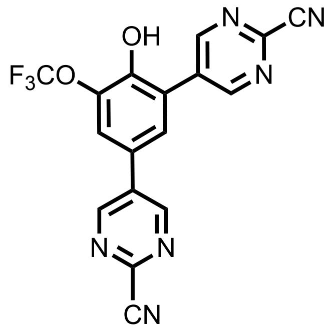

5,5'-(4-Hydroxy-5-(trifluoromethoxy)-1,3-phenylene)bis(pyrimidine-2-carbonitrile) (**7s**)
General procedure B was followed using 2-(trifluoromethoxy)phenol (**5j**, 18 mg, 1.0 equiv), 5-bromopyrimidine-2-carbonitrile (**6p**, 40 mg, 2.2 equiv), 3DPA2FBN (1.9 mg, 3.0 mol%), 1.0 M aqueous Na_2CO_3 (150 µL, 1.5 equiv), and MeCN (1.0 mL, 0.10 M) for 16 h. Purification by reverse-phase column chromatography (70% MeCN:H_2O-0.1% FA) afforded the title compound **7s** as a light yellow oil (18 mg, 0.047 mmol, 47% yield).
^{1}H-NMR (400 MHz, acetone-*d$_6$*) δ 9.43 (s, 2H), 9.38 (s, 2H), 8.23 (d, *J* = 2.2 Hz, 1H), 8.12 – 8.07 (m, 1H).
^{13}C{^{1}H}-NMR (151 MHz, acetone-*d$_6$*) δ 159.1, 156.8, 150.3, 144.1, 143.9, 139.1, 135.3, 134.1, 129.6, 125.9, 125.0, 123.7, 121.7 (q, *J* = 258.3 Hz), 117.0, 116.9.
^{19}F{^{1}H}-NMR (376 MHz, acetone-*d$_6$*) δ –58.9.
HRMS (ESI-FTICR) *m/z* = 385.0655 calcd for $C_{17}H_8F_3N_6O_2^+$ [M+H]$^+$, found 385.0664.

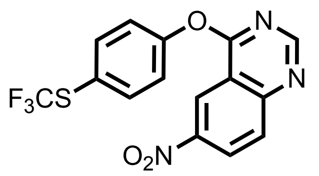

6-Nitro-4-(4-((trifluoromethyl)thio)phenoxy)quinazoline (**7t**)

General procedure B was followed using 4-((trifluoromethyl)thio)phenol (**5i**, 29 mg, 1.5 equiv), 4-chloro-6-nitroquinazoline (**6l**, 21 mg, 1.0 equiv), 3DPA2FBN (1.9 mg, 3.0 mol%), 1.0 M aqueous Na_2CO_3 (150 µL, 1.5 equiv), and MeCN (1.0 mL, 0.10 M) for 16 h. Purification by reverse-phase column chromatography (85% MeCN:H_2O-0.1% FA) afforded the title compound **7t** as a yellow oil (37 mg, 0.099 mmol, 99% yield).

^{1}H-NMR (400 MHz, $CDCl_3$) δ 9.30 (s, 1H), 8.92 (s, 1H), 8.71 (d, *J* = 8.4 Hz, 1H), 8.18 (d, *J* = 9.1 Hz, 1H), 7.88 – 7.77 (m, 2H), 7.44 – 7.35 (m, 2H).

^{13}C{^{1}H}-NMR (151 MHz, $CDCl_3$) δ 167.5, 157.1, 154.3, 153.9, 146.2, 138.2, 130.2, 129.5 (q, *J* = 308.5 Hz), 128.0, 123.0, 122.5, 120.8, 115.7.

^{19}F{^{1}H}-NMR (376 MHz, $CDCl_3$) δ –42.7.

HRMS (ESI-FTICR) *m/z* = 368.0311 calcd for $C_{15}H_9F_3N_3O_3S^+$ [M+H]$^+$, found 368.0310.

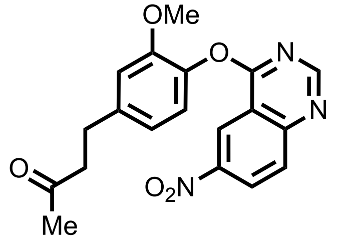

4-(3-Methoxy-4-((6-nitroquinazolin-4-yl)oxy)phenyl)butan-2-one (**7u**)

General procedure B was followed using 4-(4-hydroxy-3-methoxyphenyl)butan-2-one (**5m**, 19 mg, 1.0 equiv), 4-chloro-6-nitroquinazoline (**6l**, 31 mg, 1.5 equiv), 3DPA2FBN (1.9 mg, 3.0 mol%), 1.0 M aqueous Na_2CO_3 (150 µL, 1.5 equiv), and MeCN (1.0 mL, 0.10 M) for 16 h. Purification by reverse-phase column chromatography (50% MeCN:H_2O-0.1% FA) afforded the title compound **7u** as an amorphous yellow solid (33 mg, 0.091 mmol, 91% yield).

^{1}H-NMR (400 MHz, acetone-*d$_6$*) δ 9.22 (d, *J* = 2.6 Hz, 1H), 8.82 (s, 1H), 8.76 (dd, *J* = 9.2, 2.6 Hz, 1H), 8.22 (d, *J* = 9.2 Hz, 1H), 7.24 (d, *J* = 8.1 Hz, 1H), 7.13 (d, *J* = 1.9 Hz, 1H), 6.94 (dd, *J* = 8.1, 2.0 Hz, 1H), 3.74 (s, 3H), 2.92 – 2.89 (m, 4H), 2.15 (s, 3H).

^{13}C{^{1}H}-NMR (151 MHz, acetone-*d$_6$*) δ 207.3, 168.7, 158.3, 155.0, 152.0, 147.0, 142.2, 140.0, 130.8, 128.5, 123.4, 121.4, 121.3, 116.2, 114.1, 56.2, 45.3, 30.4, 30.3.

HRMS (ESI-FTICR) *m/z* = 368.1241 calcd for $C_{19}H_{18}N_3O_5^+$ [M+H]$^+$, found 368.1247.

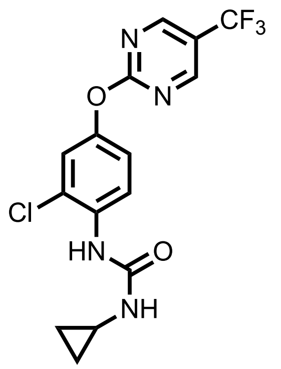

1-(2-Chloro-4-((5-(trifluoromethyl)pyrimidin-2-yl)oxy)phenyl)-3-cyclopropylurea (**7v**)

General procedure B was followed using 1-(2-chloro-4-hydroxyphenyl)-3-cyclopropylurea (**5o**, 34 mg, 1.5 equiv), 2-chloro-5-(trifluoromethyl)pyrimidine (**6m**, 18 mg, 1.0 equiv), 3DPA2FBN (1.9 mg, 3.0 mol%), 1.0 M aqueous Na_2CO_3 (150 µL, 1.5 equiv), and MeCN (1.0 mL, 0.10 M) for 16 h. Purification by reverse-phase column chromatography (80% MeCN:H_2O-0.1% FA) afforded the title compound **7v** as an amorphous yellow solid (19 mg, 0.051 mmol, 51% yield).

^{1}H-NMR (400 MHz, acetone-*d6*) δ 8.98 (s, 2H), 8.42 (d, *J* = 9.1 Hz, 1H), 7.66 (s, 1H), 7.38 (d, *J* = 2.7 Hz, 1H), 7.19 (dd, *J* = 9.1, 2.7 Hz, 1H), 6.56 (s, 1H), 2.74 – 2.63 (m, 1H), 0.82 – 0.70 (m, 2H), 0.60 – 0.51 (m, 2H).

^{13}C{^{1}H}-NMR (151 MHz, acetone-*d6*) δ 168.0, 158.7 (q, *J* = 3.3 Hz), 156.2, 147.7, 135.6, 124.4 (q, *J* = 270.3 Hz), 123.1, 122.4, 122.0, 121.7, 120.8 (q, *J* = 34.2 Hz), 23.3, 7.1.

^{19}F{^{1}H}-NMR (376 MHz, acetone-*d6*) δ –62.08.

HRMS (ESI-FTICR) *m/z* = 373.0674 calcd for $C_{15}H_{13}ClF_3N_4O_2^+$ [M+H]$^+$, found 373.0678.

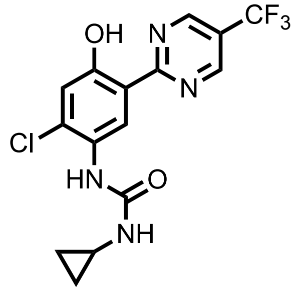

1-(2-Chloro-4-hydroxy-5-(5-(trifluoromethyl)pyrimidin-2-yl)phenyl)-3-cyclopropylurea (**7v'**)

General procedure B was followed using 1-(2-chloro-4-hydroxyphenyl)-3-cyclopropylurea (**5o**, 34 mg, 1.5 equiv), 2-bromo-5-(trifluoromethyl)pyrimidine (**6m'**, 23 mg, 1.0 equiv), 3DPA2FBN (1.9 mg, 3.0 mol%), 1.0 M aqueous Na_2CO_3 (150 µL, 1.5 equiv), and MeCN (1.0 mL, 0.10 M) for 16 h. Purification by reverse-phase column chromatography (50–80% MeCN:H_2O-0.1% FA) afforded the title compounds **7v'** as an amorphous beige solid (6.4 mg, 0.017 mmol, 17% yield), **7v''** as a yellow oil (5.3 mg, 0.014 mmol, 14% yield), and **7v** as an amorphous tan solid (25 mg, 0.068 mmol, 68% yield).

General procedure B with 2-iodo-5-(trifluoromethyl)pyrimidine (**6m''**, 27 mg, 1.0 equiv) instead afforded the title compounds **7v'** as an amorphous light brown solid (18 mg, 0.049 mmol, 49% yield), **7v''** as a yellow oil (6.0 mg, 0.016 mmol, 16% yield), and **7v** as an amorphous tan solid (4.0 mg, 0.011 mmol, 11% yield).

^{1}H-NMR (400 MHz, acetone-*d6*) δ 12.51 (s, 1H), 9.33 (s, 2H), 7.50 (s, 1H), 7.06 (s, 1H), 6.41 (s, 1H), 2.70 – 2.67 (m, 1H), 0.77 – 0.73 (m, 2H), 0.57 – 0.55 (m, 2H).

Note: Missing one urea N–H proton.

^{13}C{^{1}H}-NMR (151 MHz, acetone-*d6*) δ 167.6, 157.4, 156.6, 155.3 (q, *J* = 3.4 Hz), 130.5, 129.8, 124.0 (q, *J* = 272.5 Hz), 123.7, 122.5 (q, *J* = 33.8 Hz), 118.7, 117.9, 23.3, 7.2.

^{19}F{^{1}H}-NMR (376 MHz, acetone-*d6*) δ –62.86.

HRMS (ESI-FTICR) *m/z* = 373.06736 calcd for $C_{15}H_{13}ClF_3N_4O_2^+$ [M+H]$^+$, found 373.06741.

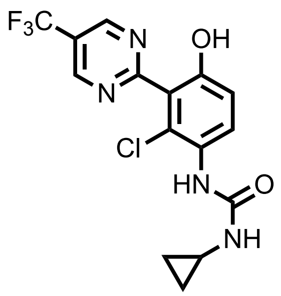

1-(2-Chloro-4-hydroxy-3-(5-(trifluoromethyl)pyrimidin-2-yl)phenyl)-3-cyclopropylurea (**7v''**)
See above procedure for details.
^{1}H-NMR (400 MHz, acetone-*d$_6$*) δ 9.35 (s, 2H), 8.19 (d, *J* = 9.1 Hz, 1H), 7.50 (s, 1H), 6.97 (d, *J* = 9.1 Hz, 1H), 6.39 (s, 1H), 2.69 – 2.63 (m, 1H), 0.75 – 0.71 (m, 2H), 0.56 – 0.52 (m, 2H).
Note: Missing phenolic O–H proton.
^{13}C{^{1}H}-NMR (151 MHz, acetone-*d$_6$*) δ 168.0, 156.6, 155.5 (q, *J* = 3.9 Hz), 152.9, 130.7, 125.3, 125.2, 124.4, 124.1 (q, *J* = 271.4 Hz), 123.2 (q, *J* = 34.9 Hz), 116.2, 23.2, 7.2.
^{19}F{^{1}H}-NMR (376 MHz, acetone-*d$_6$*) δ –62.92.
HRMS (ESI-FTICR) *m/z* = 373.06736 calcd for $C_{15}H_{13}ClF_3N_4O_2^+$ [M+H]$^+$, found 373.06742.

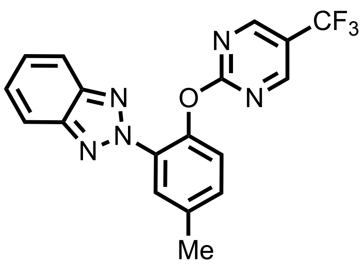

2-(5-Methyl-2-((5-(trifluoromethyl)pyrimidin-2-yl)oxy)phenyl)-2H-benzo[d][1,2,3]triazole (**7w**)
General procedure B was followed using drometrizole (**5q**, 23 mg, 1.0 equiv), 2-chloro-5-(trifluoromethyl)pyrimidine (**6m**, 27 mg, 1.5 equiv), 3DPA2FBN (1.9 mg, 3.0 mol%), 1.0 M aqueous Na_2CO_3 (150 µL, 1.5 equiv), and MeCN (1.0 mL, 0.10 M) for 16 h. Purification by reverse-phase column chromatography (50% MeCN:H_2O-0.1% FA) afforded the title compound **7w** as an amorphous yellow solid (21 mg, 0.055 mmol, 55% yield).
General procedure B with drometrizole (**5q**, 34 mg, 1.5 equiv) and 2-bromo-5-(trifluoromethyl)pyrimidine (**6m'**, 23 mg, 1.0 equiv) instead afforded the title compound **7w** as an amorphous beige solid (7.2 mg, 0.019 mmol, 19% yield).
^{1}H-NMR (400 MHz, acetone-*d$_6$*) δ 8.88 (s, 2H), 8.04 (d, *J* = 2.1 Hz, 1H), 7.81 – 7.72 (m, 2H), 7.56 – 7.47 (m, 2H), 7.46 – 7.38 (m, 2H), 2.55 (s, 3H).
^{13}C{^{1}H}-NMR (151 MHz, acetone-*d$_6$*) δ 168.0, 158.3 (q, *J* = 3.7 Hz), 145.4, 143.6, 138.1, 133.5, 132.1, 128.2, 126.9, 125.5, 124.3 (q, *J* = 270.3 Hz), 120.6 (q, *J* = 34.2 Hz), 118.9, 20.8.
^{19}F{^{1}H}-NMR (376 MHz, acetone-*d$_6$*) δ –62.06.
HRMS (ESI-FTICR) *m/z* = 372.1067 calcd for $C_{18}H_{13}F_3N_5O^+$ [M+H]$^+$, found 372.1066.

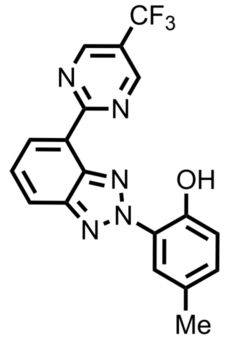

4-Methyl-2-(4-(5-(trifluoromethyl)pyrimidin-2-yl)-2H-benzo[d][1,2,3]triazol-2-yl)phenol (**7w'**)

General procedure B was followed using drometrizole (**5q**, 34 mg, 1.5 equiv), 2-iodo-5-(trifluoromethyl)pyrimidine (**6m''**, 27 mg, 1.0 equiv), 3DPA2FBN (1.9 mg, 3.0 mol%), 1.0 M aqueous Na_2CO_3 (150 µL, 1.5 equiv), and MeCN (1.0 mL, 0.10 M) for 16 h. Purification by reverse-phase column chromatography (95% MeCN:H_2O-0.1% FA) afforded the title compound **7w'** as an amorphous dark yellow solid (17 mg, 0.046 mmol, 46% yield).

^{1}H-NMR (400 MHz, acetone-*d6*) δ 11.75 (s, 1H), 9.42 (s, 2H), 8.79 (d, *J* = 7.2 Hz, 1H), 8.32 (d, *J* = 8.3 Hz, 1H), 8.27 (d, *J* = 1.3 Hz, 1H), 7.82 (dd, *J* = 8.5, 7.3 Hz, 1H), 7.29 (dd, *J* = 8.5, 1.9 Hz, 1H), 7.14 (d, *J* = 8.4 Hz, 1H), 2.43 (s, 3H).

^{13}C{^{1}H}-NMR (151 MHz, acetone-*d6*) δ 166.4, 155.9 (q, *J* = 4.4 Hz), 149.1, 145.2, 141.4, 132.7, 131.3, 130.4, 129.3, 128.5, 126.3 (q, *J* = 262.9 Hz), 123.3 (q, *J* = 28.3 Hz), 123.0, 121.7, 119.8, 118.7, 20.5.

^{19}F{^{1}H}-NMR (376 MHz, acetone-*d6*) δ –62.84.

HRMS (ESI-FTICR) *m/z* = 372.1067 calcd for $C_{18}H_{13}F_3N_5O^+$ [M+H]$^+$, found 372.1074.

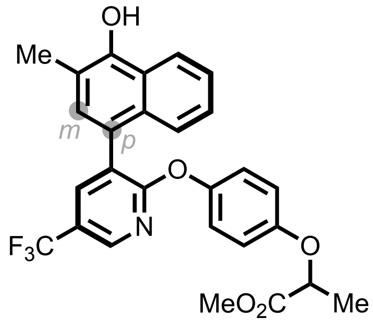

Methyl 2-(4-((3-(4-hydroxy-3-methylnaphthalen-1-yl)-5-(trifluoromethyl)pyridin-2-yl)oxy)phenoxy)propanoate (**7x**)

General procedure B was followed using 2-methyl-1-naphthol (**5r**, 63 mg, 1.5 equiv), haloxyfop-methyl (**6q**, 100 mg, 1.0 equiv), 3DPA2FBN (5.1 mg, 3.0 mol%), 1.0 M aqueous Na_2CO_3 (400 µL, 1.5 equiv), and MeCN (2.7 mL, 0.10 M) for 16 h. Purification by reverse-phase column chromatography (80% MeCN:H_2O-0.1% FA) afforded the title compound **7x** as an amorphous yellow solid (55 mg, 0.11 mmol, 42% yield, 7.1:1 p:m ratio by ^{19}F{^{1}H}-NMR).

^{1}H-NMR (400 MHz, acetone-*d6*) δ 8.49 (d, *J* = 2.4 Hz, 1H), 8.36 – 8.31 (m, 1H), 8.08 (d, *J* = 2.3 Hz, 1H), 7.66 – 7.60 (m, 1H), 7.53 – 7.44 (m, 2H), 7.40 (s, 1H), 7.02 (d, *J* = 9.0 Hz, 2H), 6.88 (d, *J* = 9.0 Hz, 2H), 4.84 (q, *J* = 6.7 Hz, 1H), 3.70 (s, 3H), 2.49 (s, 3H), 1.54 (d, *J* = 6.8 Hz, 3H).

^{13}C{^{1}H}-NMR (151 MHz, acetone-*d6*) δ 172.8, 164.9, 155.9, 151.4, 148.4, 144.6 (q, *J* = 4.3 Hz), 138.9 (q, *J* = 3.4 Hz), 132.3, 132.2, 126.5, 126.20, 126.18, 125.84, 125.79, 125.4, 124.3 (q, *J* = 260.5 Hz), 123.5, 122.9, 122.0 (q, *J* = 32.8 Hz), 118.1, 116.6, 73.5, 52.3, 18.8, 16.4.

^{19}F{^{1}H}-NMR (376 MHz, acetone-*d6*) δ –61.8.

Note: Presence of meta-isomer in 1:7.1 ratio by ^{19}F{^{1}H}-NMR. Integrating –61.83 ppm (meta) versus –61.82 ppm (para).

HRMS (ESI-FTICR) *m/z* = 498.1523 calcd for $C_{27}H_{23}F_3NO_5^+$ [M+H]$^+$, found 498.1528.

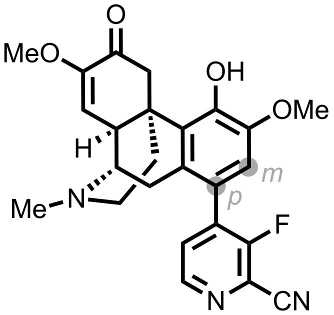

3-Fluoro-4-((4bR,8aS,9S)-4-hydroxy-3,7-dimethoxy-11-methyl-6-oxo-6,8a,9,10-tetrahydro-5H-9,4b-(epiminoethano)phenanthren-1-yl)picolinonitrile (**7y**)

General procedure B was followed using sinomenine•HCl (**5s**, 37 mg, 1.0 equiv), 3-fluoro-4-iodopicolinonitrile (**6a**, 37 mg, 1.5 equiv), 3DPA2FBN (1.9 mg, 3.0 mol%), 1.0 M aqueous Na_2CO_3 (200 µL, 2.0 equiv), and MeCN (1.0 mL, 0.10 M) for 3 h. Purification by reverse-phase column chromatography (35% MeCN:H_2O-0.1% FA) afforded the title compound as a formate salt. The combined fractions were washed with satd aq $NaHCO_3$ and extracted with EtOAc (3x). The combined organic layers were concentrated to afford the title compound **7y** as an amorphous beige solid (18 mg, 0.040 mmol, 40% yield, 8.2:1 p:m ratio by $^{19}F\{^1H\}$-NMR).

^{1}H-NMR (400 MHz, methanol-*d₄*) δ 8.60 (d, *J* = 4.6 Hz, 1H), 7.73 – 7.65 (m, 1H), 6.77 (s, 1H), 5.78 (s, 1H), 4.44 (d, *J* = 15.6 Hz, 1H), 3.83 (s, 3H), 3.53 (s, 3H), 3.47 – 3.43 (m, 1H), 3.40 – 3.33 (m, 1H), 3.19 – 3.14 (m, 1H), 2.95 – 2.85 (m, 2H), 2.78 – 2.67 (m, 1H), 2.65 – 2.60 (m, 1H), 2.58 (s, 3H), 2.43 – 2.34 (m, 1H), 2.19 – 2.12 (m, 1H), 2.06 – 1.97 (m, 1H).

$^{13}C\{^1H\}$-NMR (151 MHz, methanol-*d₄*) δ 195.3, 160.4 (d, *J* = 264.8 Hz), 153.9, 148.7 (d, *J* = 5.5 Hz), 148.2, 148.0, 140.3 (d, *J* = 14.2 Hz), 132.4, 127.7, 126.5 (d, *J* = 24.9 Hz), 123.8, 122.3 (d, *J* = 66.5 Hz), 115.2, 114.3 (d, *J* = 4.4 Hz), 112.7, 58.4, 56.7, 55.6, 44.81, 44.75, 42.5, 42.12, 42.08, 41.3, 34.7.

$^{19}F\{^1H\}$-NMR (376 MHz, methanol-*d₄*) δ –121.0.

Note: Presence of meta-isomer in 1:8.2 ratio by $^{19}F\{^1H\}$-NMR. Integrating –120.21 ppm (meta) versus –121.03 ppm (para).

HRMS (ESI-FTICR) *m/z* = 450.1824 calcd for $C_{25}H_{25}FN_3O_4^+$ [M+H]$^+$, found 450.1825.

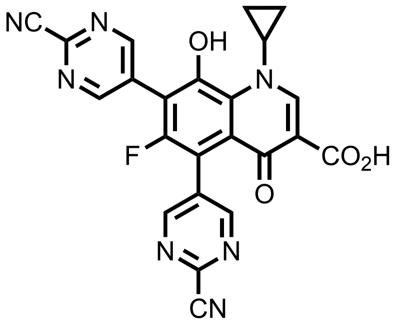

5,7-Bis(2-cyanopyrimidin-5-yl)-1-cyclopropyl-6-fluoro-8-hydroxy-4-oxo-1,4-dihydroquinoline-3-carboxylic acid (**7z**)

General procedure B was followed using 1-cyclopropyl-6,7-difluoro-8-hydroxy-4-oxo-1,4-dihydroquinoline-3-carboxylic acid (**5t**, 28 mg, 1.0 equiv), 5-bromopyrimidine-2-carbonitrile (**6p**, 55 mg, 3.0 equiv), 3DPA2FBN (1.9 mg, 3.0 mol%), 1.0 M aqueous Na_2CO_3 (400 µL, 4.0 equiv), and MeCN (1.0 mL, 0.10 M) for 16 h. Purification by reverse-phase column chromatography (65% MeCN:H_2O-0.1% FA) afforded the title compound **7z** as an amorphous yellow solid (11 mg, 0.023 mmol, 23% yield).

^{1}H-NMR (400 MHz, methanol-*d₄*) δ 9.14 (s, 2H), 8.91 (s, 1H), 8.83 (s, 2H), 4.60 – 4.49 (m, 1H), 1.33 – 1.18 (m, 4H).

Note: Trace formic acid present. Carboxylic acid and phenol protons not observed.

^{13}C{^{1}H}-NMR (151 MHz, methanol-*d₄*) δ 179.8 (d, *J* = 3.2 Hz), 169.1, 160.72, 160.71, 159.2, 157.1 (d, *J* = 246.3 Hz), 151.2, 144.6, 143.4, 135.0, 134.5, 131.0, 127.7 (d, *J* = 4.6 Hz), 117.2, 117.0, 116.6 (d, *J* = 15.5 Hz), 115.9 (d, *J* = 17.8 Hz), 108.9, 43.5, 9.9.

Note: Trace formic acid present.

^{19}F{^{1}H}-NMR (376 MHz, methanol-*d₄*) δ –116.6.

HRMS (ESI-FTICR) *m/z* = 468.0862 calcd for $C_{23}H_{11}FN_7O_4^-$ [M–H]$^-$, found 468.0861.

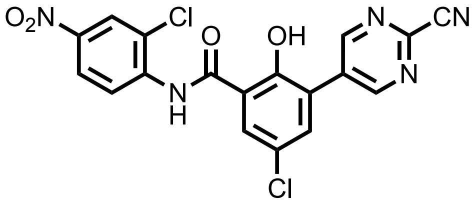

5-Chloro-N-(2-chloro-4-nitrophenyl)-3-(2-cyanopyrimidin-5-yl)-2-hydroxybenzamide (**7za**)

General procedure B was followed using niclosamide (**5u**, 33 mg, 1.0 equiv), 5-bromopyrimidine-2-carbonitrile (**6p**, 37 mg, 2.0 equiv), 3DPA2FBN (3.8 mg, 6.0 mol%), Na_2CO_3 (32 mg, 3.0 equiv), and MeCN (1.0 mL, 0.10 M) for 16 h. Purification by reverse-phase column chromatography (90% MeCN:H_2O-0.1% FA) afforded the title compound **7za** as an amorphous beige solid (19 mg, 0.044 mmol, 44% yield).

^{1}H-NMR (600 MHz, acetone-*d₆*) δ 9.25 (s, 2H), 8.52 (d, *J* = 9.0 Hz, 1H), 8.42 (d, *J* = 2.5 Hz, 1H), 8.32 (dd, *J* = 9.0, 2.5 Hz, 1H), 8.23 (d, *J* = 2.6 Hz, 1H), 7.81 (d, *J* = 2.5 Hz, 1H).

Note: Missing phenolic O–H proton.

^{13}C{^{1}H}-NMR (151 MHz, acetone-*d₆*) δ 167.1, 158.5, 143.3, 135.1, 134.82, 134.75, 131.6, 131.3, 126.0, 125.9, 125.6, 125.5, 124.3, 123.9, 120.6, 120.1, 117.0.

HRMS (ESI-FTICR) *m/z* = 427.99588 calcd for $C_{18}H_8Cl_2N_5O_4^-$ [M–H]$^-$, found 427.99595.

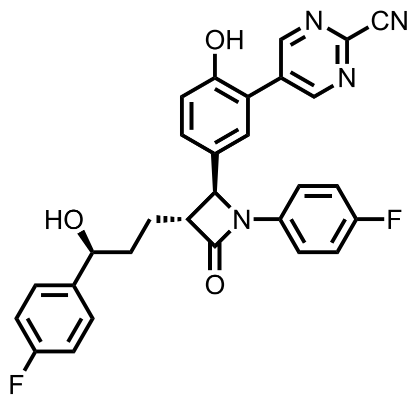

5-(5-((2S,3R)-1-(4-Fluorophenyl)-3-((S)-3-(4-fluorophenyl)-3-hydroxypropyl)-4-oxoazetidin-2-yl)-2-hydroxyphenyl)pyrimidine-2-carbonitrile (**7zb**)

General procedure B was followed using ezetimibe (**5v**, 41 mg, 1.0 equiv), 5-bromopyrimidine-2-carbonitrile (**6p**, 28 mg, 1.5 equiv), 3DPA2FBN (1.9 mg, 3.0 mol%), $NaHCO_3$ (10 mg, 1.2 equiv), and MeCN (1.0 mL, 0.10 M) for 3 h. Purification by reverse-phase column chromatography (70% MeCN:H_2O-0.1% FA) afforded the title compound **7zb** as an amorphous beige solid (25 mg, 0.049 mmol, 49% yield, 65% brsm) and ezetimibe (**5v**) as an amorphous off-white solid (10 mg, 0.024 mmol).

^{1}H-NMR (600 MHz, methanol-*d₄*) δ 9.12 (s, 2H), 7.51 (d, *J* = 2.1 Hz, 1H), 7.35 (dd, *J* = 8.4, 2.1 Hz, 1H), 7.34 – 7.28 (m, 4H), 7.04 – 6.96 (m, 5H), 4.62 (t, *J* = 6.0 Hz, 1H), 3.21 – 3.14 (m, 1H), 2.02 – 1.77 (m, 5H).

^{13}C{^{1}H}-NMR (151 MHz, methanol-*d₄*) δ 169.7, 163.5 (d, *J* = 244.1 Hz), 160.5 (d, *J* = 241.9 Hz), 158.9, 156.8, 143.6, 142.2 (d, *J* = 3.3 Hz), 136.0, 135.2 (d, *J* = 3.1 Hz), 131.0, 130.3, 129.5, 128.8 (d, *J* = 7.8 Hz), 121.6, 120.0 (d, *J* = 7.9 Hz), 118.2, 117.1, 116.7 (d, *J* = 22.9 Hz), 115.9 (d, *J* = 21.7 Hz), 73.8, 61.8, 61.2, 37.4, 26.1.

^{19}F{^{1}H}-NMR (376 MHz, methanol-*d₄*) δ –117.7, –120.1.

HRMS (ESI-FTICR) *m/z* = 513.17327 calcd for $C_{29}H_{23}F_2N_4O_3^+$ [M+H]$^+$, found 513.17326.

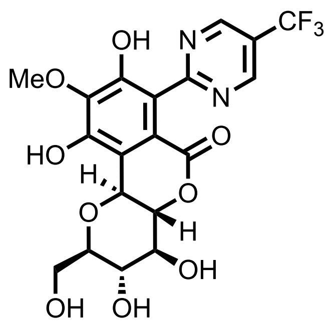

(2R,3S,4S,4aR,10bS)-3,4,8,10-Tetrahydroxy-2-(hydroxymethyl)-9-methoxy-7-(5-(trifluoromethyl)pyrimidin-2-yl)-3,4,4a,10b-tetrahydropyrano[3,2-c]isochromen-6(2H)-one (**7zc**)

General procedure B was followed using (–)-bergenin (**5w**, 49 mg, 1.5 equiv), 2-bromo-5-(trifluoromethyl)pyrimidine (**6m'**, 23 mg, 1.0 equiv), 3DPA2FBN (1.9 mg, 3.0 mol%), 1.0 M aqueous Na_2CO_3 (150 µL, 1.5 equiv), and MeCN (1.0 mL, 0.10 M) for 16 h. Purification by reverse-phase column chromatography (35% MeCN:H_2O-0.1% FA) afforded the title compound **7zc** as an amorphous brown solid (18 mg, 0.038 mmol, 38% yield) and **7zc'** as an amorphous light brown solid (6.5 mg, 0.014 mmol, 14% yield).

General procedure B with 2-iodo-5-(trifluoromethyl)pyrimidine (**6m''**, 27 mg, 1.0 equiv) instead afforded the title compound **7zc** as an amorphous brown solid (26 mg, 0.055 mmol, 55% yield).

^{1}H-NMR (600 MHz, methanol-*d₄*) δ 9.17 (s, 2H), 5.02 (d, *J* = 10.4 Hz, 1H), 4.17 (t, *J* = 9.9 Hz, 1H), 4.08 – 4.03 (m, 1H), 3.97 (s, 3H), 3.79 (t, *J* = 9.0 Hz, 1H), 3.74 – 3.68 (m, 2H), 3.45 (t, *J* = 8.6 Hz, 1H).

^{13}C{^{1}H}-NMR (151 MHz, methanol-*d₄*) δ 169.7, 164.3, 155.5 (q, *J* = 4.0 Hz), 151.4, 150.1, 141.8, 124.6 (q, *J* = 257.2 Hz), 123.8 (q, *J* = 48.0 Hz), 122.0, 118.6, 118.3, 83.2, 81.1, 75.6, 74.3, 71.9, 62.7, 61.2.

^{19}F{^{1}H}-NMR (376 MHz, methanol-*d₄*) δ –63.8.

HRMS (ESI-FTICR) *m/z* = 475.0959 calcd for $C_{19}H_{18}F_3N_2O_9^+$ [M+H]$^+$, found 475.0963.

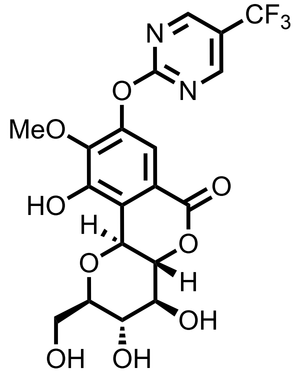

(2R,3S,4S,4aR,10bS)-3,4,10-Trihydroxy-2-(hydroxymethyl)-9-methoxy-8-((5-(trifluoromethyl)pyrimidin-2-yl)oxy)-3,4,4a,10b-tetrahydropyrano[3,2-c]isochromen-6(2H)-one (**7zc'**)

General procedure B was followed using (–)-bergenin (**5w**, 33 mg, 1.0 equiv), 2-chloro-5-(trifluoromethyl)pyrimidine (**6m**, 37 mg, 2.0 equiv), 3DPA2FBN (1.9 mg, 3.0 mol%), $NaHCO_3$ (17 mg, 2.0 equiv), and MeCN (1.0 mL, 0.10 M) for 16 h. Purification by reverse-phase column chromatography (35% MeCN:H_2O-0.1% FA) afforded the title compound **7zc'** as an amorphous yellow solid (18 mg, 0.038 mmol, 38% yield).

^{1}H-NMR (400 MHz, acetone-*d₆*) δ 9.01 (s, 2H), 7.41 (s, 1H), 5.22 (d, *J* = 10.4 Hz, 1H), 4.23 (t, *J* = 9.4 Hz, 1H), 4.18 – 4.09 (m, 1H), 3.98 – 3.85 (m, 2H), 3.81 (s, 3H), 3.77 – 3.67 (m, 1H), 3.61 – 3.49 (m, 1H).

Note: Phenol proton not observed.

^{13}C{^{1}H}-NMR (151 MHz, acetone-*d₆*) δ 167.5, 163.1, 158.8 (q, *J* = 4.1 Hz), 149.8, 146.8, 145.8, 124.3 (d, *J* = 268.8 Hz), 123.5, 121.0 (q, *J* = 34.1 Hz), 119.7, 116.7, 83.1, 80.7, 75.5, 73.8, 71.9, 62.6, 60.8.

^{19}F{^{1}H}-NMR (376 MHz, acetone-*d₆*) δ –62.0.

HRMS (ESI-FTICR) *m/z* = 475.09589 calcd for $C_{19}H_{18}F_3N_2O_9^+$ [M+H]$^+$, found 475.09592.

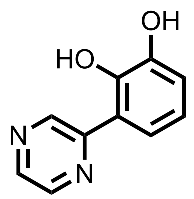

3-(Pyrazin-2-yl)benzene-1,2-diol (o-Periplanpyrazine A) (**7zd**)

General procedure B was followed using catechol (**5c**, 41 mg, 1.5 equiv), 2-iodopyrazine (**6r**, 25 µL, 1.0 equiv), 3DPA2FBN (4.8 mg, 3.0 mol%), 1.0 M aqueous Na_2CO_3 (380 µL, 1.5 equiv), and MeCN (2.5 mL, 0.10 M) for 48 h. Purification by reverse-phase column chromatography (25% MeCN:H_2O-0.1% FA) afforded the title compound **7zd** (*o*-periplanpyrazine A) as an amorphous off-white solid (17.2 mg, 0.091 mmol, 37% yield).

^{1}H-NMR (600 MHz, DMSO-*d6*) δ 9.39 (d, *J* = 1.4 Hz, 1H), 8.67 (dd, *J* = 2.5, 1.5 Hz, 1H), 8.60 (d, *J* = 2.6 Hz, 1H), 7.47 (dd, *J* = 8.0, 1.3 Hz, 1H), 6.90 (dd, *J* = 7.8, 1.3 Hz, 1H), 6.77 (t, *J* = 7.9 Hz, 1H).

Note: Missing phenolic O–H protons.

^{13}C{^{1}H}-NMR (151 MHz, DMSO-*d6*) δ 152.0, 146.6, 146.3, 143.7, 142.3, 141.8, 119.5, 119.0, 118.3, 117.1.

HRMS (ESI-FTICR) *m/z* = 189.0659 calcd for $C_{10}H_9N_2O_2^+$ [M+H]$^+$, found 189.0656.

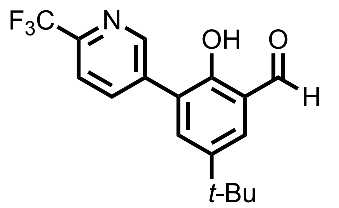

5-(tert-Butyl)-2-hydroxy-3-(6-(trifluoromethyl)pyridin-3-yl)benzaldehyde (**7ze**)

General procedure B was followed using 5-(*tert*-butyl)-2-hydroxybenzaldehyde (**5x**, 51 µL, 1.5 equiv), 5-iodo-2-(trifluoromethyl)pyridine (**6s**, 55 mg, 1.0 equiv), 3DPA2FBN (3.8 mg, 3.0 mol%), 1.0 M aqueous Na_2CO_3 (300 µL, 1.5 equiv), and MeCN (2.0 mL, 0.10 M) for 16 h. Purification by reverse-phase column chromatography (80% MeCN:H_2O-0.1% FA) afforded the title compound **7ze** as an amorphous light yellow solid (48 mg, 0.15 mmol, 74% yield).

^{1}H-NMR (400 MHz, acetone-*d6*) δ 11.58 (s, 1H), 10.12 (s, 1H), 9.00 (d, *J* = 1.9 Hz, 1H), 8.34 (dd, *J* = 8.1, 1.6 Hz, 1H), 7.99 (d, *J* = 2.5 Hz, 1H), 7.94 (d, *J* = 2.5 Hz, 1H), 7.93 (d, *J* = 8.1 Hz, 1H), 1.42 (s, 9H).

^{13}C{^{1}H}-NMR (151 MHz, acetone-*d6*) δ 199.3, 151.2, 146.9 (q, *J* = 34.1 Hz), 144.3, 139.2, 136.8, 136.1, 132.7, 126.5, 125.5, 122.9 (q, *J* = 272.6 Hz), 121.7, 120.9 (q, *J* = 2.8 Hz), 35.0, 31.5.

^{19}F{^{1}H}-NMR (376 MHz, acetone-*d6*) δ –68.3.

HRMS (ESI-FTICR) *m/z* = 324.1206 calcd for $C_{17}H_{17}F_3NO_2^+$ [M+H]$^+$, found 324.1201.

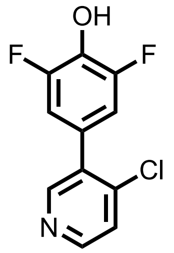

4-(4-Chloropyridin-3-yl)-2,6-difluorophenol (**7zg**)

General procedure B was followed using 2,6-difluorophenol (**5b**, 51 mg, 1.5 equiv), 4-chloro-3-iodopyridine (**6u**, 62 mg, 1.0 equiv), 3DPA2FBN (5.0 mg, 3.0 mol%), 1.0 M aqueous Na_2CO_3 (390 µL, 1.5 equiv), and MeCN (2.6 mL, 0.10 M) for 32 h. Purification by reverse-phase column chromatography (60% MeCN:H_2O-0.1% FA) afforded the title compound **7zg** as an amorphous off-white solid (35 mg, 0.14 mmol, 56% yield).

^{1}H-NMR (400 MHz, acetone-*d$_6$*) δ 9.34 (s, 1H), 8.58 (s, 1H), 8.52 (d, *J* = 5.3 Hz, 1H), 7.57 (d, *J* = 5.3 Hz, 1H), 7.26 – 7.13 (m , 2H).

^{13}C{^{1}H}-NMR (101 MHz, acetone-*d$_6$*) δ 153.0 (dd, *J* = 242.0, 7.3 Hz), 152.1, 150.7, 142.3, 135.1 (t, *J* = 15.4 Hz), 135.3, 127.0 (t, *J* = 9.0 Hz), 125.5, 114.0 (dd, *J* = 15.6, 7.6 Hz).

^{19}F{^{1}H}-NMR (376 MHz, acetone-*d$_6$*) δ –134.5.

HRMS (ESI-FTICR) *m/z* = 242.0179 calcd for $C_{11}H_7ClF_2NO^+$ $[M+H]^+$, found 242.0175.

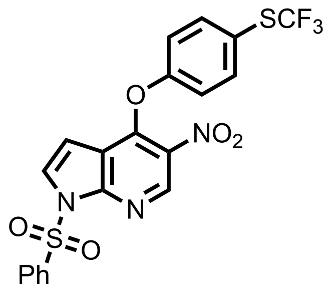

5-Nitro-1-(phenylsulfonyl)-4-(4-((trifluoromethyl)thio)phenoxy)-1H-pyrrolo[2,3-b]pyridine (**7zh**)

General procedure B was followed using 4-((trifluoromethyl)thio)phenol (**5i**, 29 mg, 1.5 equiv), 4-chloro-5-nitro-1-(phenylsulfonyl)-1*H*-pyrrolo[2,3-*b*]pyridine (**6w**, 34 mg, 1.0 equiv), 3DPA2FBN (1.9 mg, 3.0 mol%), 1.0 M aqueous Na_2CO_3 (150 µL, 1.5 equiv), and MeCN (1.0 mL, 0.10 M) for 16 h. Purification by reverse-phase column chromatography (95% MeCN:H_2O-0.1% FA) afforded the title compound **7zh** as an amorphous beige solid (18 mg, 0.036 mmol, 36% yield).

General procedure B with 4-iodo-5-nitro-1-(phenylsulfonyl)-1*H*-pyrrolo[2,3-*b*]pyridine (**6w'**, 13 mg, 1.0 equiv) and 4 h reaction time instead afforded the title compound **7zh** as an amorphous beige solid (6.4 mg, 0.013 mmol, 43% yield).

^{1}H-NMR (400 MHz, acetone-*d$_6$*) δ 9.05 (s, 1H), 8.31 – 8.24 (m, 2H), 7.93 (d, *J* = 4.1 Hz, 1H), 7.82 – 7.77 (m, 3H), 7.72 – 7.67 (m, 2H), 7.37 – 7.31 (m, 2H), 6.24 (d, *J* = 4.1 Hz, 1H).

^{13}C{^{1}H}-NMR (151 MHz, acetone-*d$_6$*) δ 159.5, 150.8, 150.0, 143.9, 140.2 (q, *J* = 251.1 Hz), 139.6, 138.4, 136.4, 136.1, 130.5, 129.6, 129.3, 120.3, 120.1, 116.0, 104.3.

^{19}F{^{1}H}-NMR (376 MHz, acetone-*d$_6$*) δ –44.3.

HRMS (ESI-FTICR) *m/z* = 496.0243 calcd for $C_{20}H_{13}F_3N_3O_5S_2^+$ $[M+H]^+$, found 496.0237.

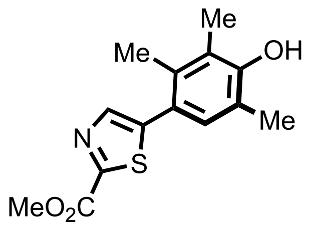

Methyl 5-(4-hydroxy-2,3,5-trimethylphenyl)thiazole-2-carboxylate (**7zi**)

General procedure B was followed using 2,3,6-trimethylphenol (**5a**, 69 mg, 1.5 equiv), methyl 5-bromothiazole-2-carboxylate (**6x**, 75 mg, 1.0 equiv), 3DPA2FBN (6.5 mg, 3.0 mol%), 1.0 M aqueous Na_2CO_3 (510 µL, 1.5 equiv), and MeCN (3.4 mL, 0.10 M) for 3 h. Purification by normal-phase column chromatography (40% EtOAc:*n*-hexanes) afforded an inseparable mixture of the title compounds **7zi** and **7zi'** as an amorphous slight yellow solid (72 mg, 0.26 mmol, 77% yield, 2.6:1 **7zi**:**7zi'** by ^{1}H-NMR).

^{1}H-NMR (600 MHz, acetone-*d*$_6$) δ 7.86 (s, 1H), 7.59 (s, 1H), 7.03 (s, 1H), 3.96 (s, 3H), 2.26 (s, 3H), 2.24 (s, 3H), 2.24 (s, 3H).

Note: Presence of meta-isomer in 1:2.6 ratio by ^{1}H-NMR. Integrating 7.94 ppm (meta) versus 7.86 ppm (para).

^{13}C{^{1}H}-NMR (151 MHz, acetone-*d*$_6$) δ 161.1, 156.8, 155.1, 144.0, 134.7, 131.2, 124.8, 124.6, 122.7, 121.7, 53.1, 17.6, 16.4, 12.8.

HRMS (ESI-FTICR) *m/z* = 278.0845 calcd for $C_{14}H_{16}NO_3S^+$ $[M+H]^+$, found 278.0851.

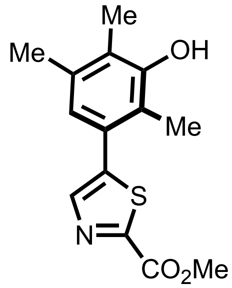

Methyl 5-(3-hydroxy-2,4,5-trimethylphenyl)thiazole-2-carboxylate (**7zi'**)

See above procedure for details.

^{1}H-NMR (600 MHz, acetone-*d*$_6$) δ 7.94 (s, 1H), 7.59 (s, 1H), 6.83 (s, 1H), 3.97 (s, 3H), 2.26 (s, 3H), 2.25 (s, 3H), 2.22 (s, 3H).

^{13}C{^{1}H}-NMR (151 MHz, acetone-*d*$_6$) δ 161.0, 157.1, 154.4, 146.4, 145.6, 144.0, 136.0, 128.0, 125.4, 121.4, 53.2, 20.0, 14.0, 12.5.

HRMS (ESI-FTICR) *m/z* = 278.0845 calcd for $C_{14}H_{16}NO_3S^+$ $[M+H]^+$, found 278.0851.

7. Radical Electrophilicity Screen

General Procedure for Radical Electrophilicity Screen

An 8 mL microwave vial equipped with a magnetic stir bar was charged with radical precursor (0.10 mmol, 1.0 equiv), 2,3,6-trimethylphenol (**5a**, 0.15 mmol, 1.5 equiv), 3DPA2FBN (0.0030 mmol, 3.0 mol%), Na_2CO_3 (0.15 mmol, 1.5 equiv), and dry MeCN (1.0 mL, 0.10 M). The vial was crimp-capped and sparged by bubbling argon through the solvent via a needle for 5 min prior to irradiation with a 427 nm Kessil lamp (100% intensity, max 45 W, 7 cm from wall of flask) and commercial fan cooling (~35 ˚C) (see **Figure S2** for setup). After 3 h, the reaction mixture was cooled to room temperature, opened to air, and an aliquot (50 µL) of the reaction mixture was diluted with MeCN (950 µL) and analyzed by LCMS at 254 nm detection. See computational section for details on the radical electrophilicity calculations.

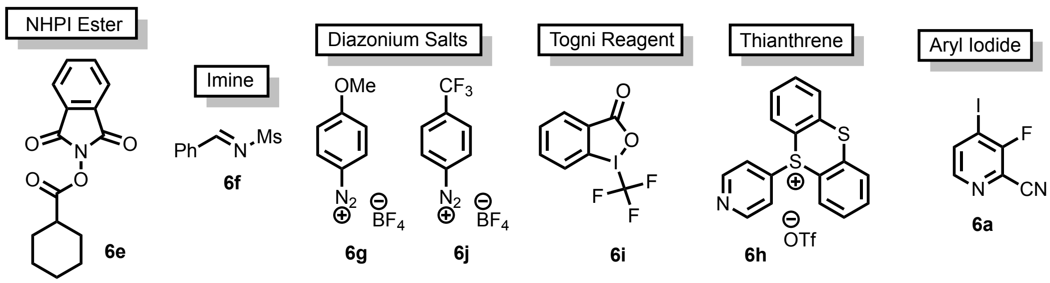

Figure S4. Radical precursors used for the screen.

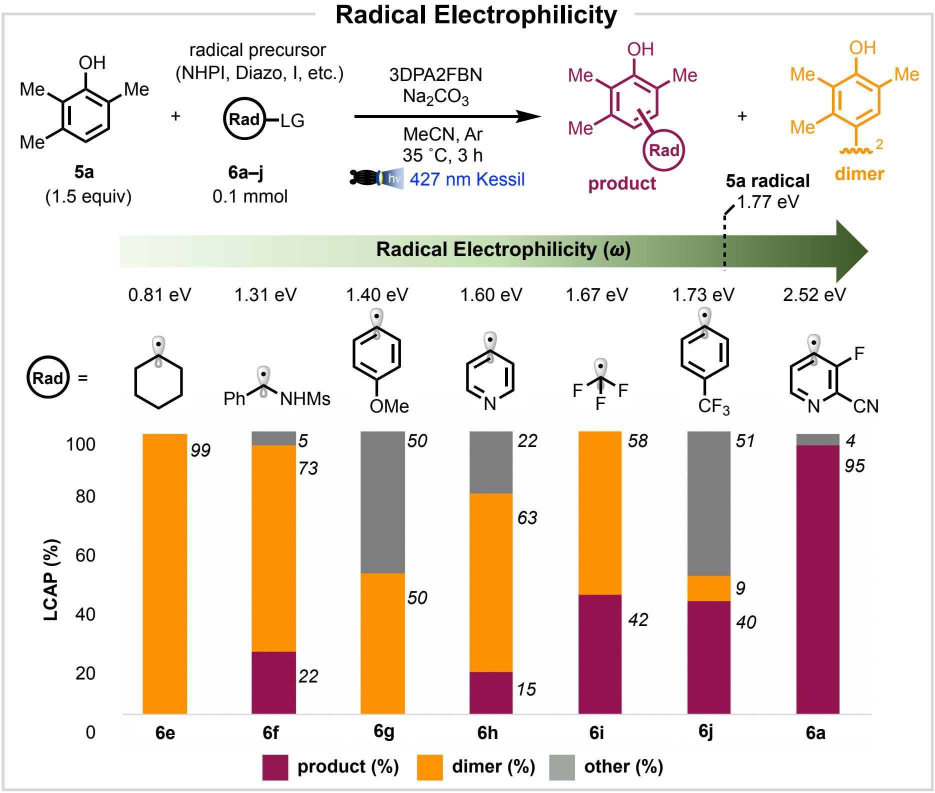

Figure S5. Radical electrophilicity as a function of reaction selectivity for cross-coupling versus phenol homocoupling. LCAP = liquid chromatography area percent at 254 nm detection. Percentages refer to area percentages of reaction products only, starting materials and photocatalyst were omitted for this analysis. Other refers to unidentified reaction byproducts. Radical electrophilicities of **6e**, **6g**, **6h**, **6i**, **6j** were sourced from literature.[7] Radicals **6f** and **6a** were calculated in this study.

8. Experimental Procedures for Application in Total Synthesis

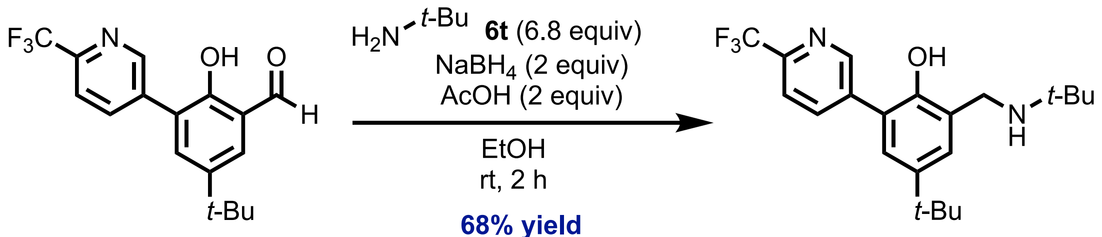

7ze **JPC-2997** (**7zf**)

4-(tert-Butyl)-2-((tert-butylamino)methyl)-6-(6-(trifluoromethyl)pyridin-3-yl)phenol (JPC-2997) (**7zf**)

To a 4 mL vial equipped with a magnetic stir bar was charged 5-(*tert*-butyl)-2-hydroxy-3-(6-(trifluoromethyl)pyridin-3-yl)benzaldehyde (**7ze**, 9.0 mg, 1.0 equiv, 28 μmol), EtOH (1.5 mL, 19 μM), and *tert*-butylamine (**6t**, 20 μL, 6.8 equiv, 0.19 mmol). The reaction mixture was stirred at room temperature. After 1 h, sodium borohydride (2.1 mg, 2.0 equiv, 56 μmol) was added to the reaction mixture and acetic acid (3.2 μL, 2.0 equiv, 56 μmol) was charged slowly. The reaction mixture was stirred at room temperature. LCMS analysis of the reaction mixture after 1 h indicated presence of the desired mass. The reaction mixture was dry loaded onto Celite™ and purified by reverse-phase column chromatography (50% MeCN:H_2O-0.1% FA) to afford the product likely as a formate/acetate salt. The combined fractions were washed with satd aq $NaHCO_3$ and extracted with EtOAc (3x). The combined organic layers were concentrated to afford the title compound **7zf** (JPC-2997) as an amorphous white solid (7.2 mg, 19 μmol, 68% yield).

^{1}H-NMR (400 MHz, DMSO-*d*$_6$) δ 8.93 (s, 1H), 8.25 (d, *J* = 8.1 Hz, 1H), 7.91 (d, *J* = 8.2 Hz, 1H), 7.26 (d, *J* = 2.2 Hz, 1H), 7.20 (d, *J* = 2.0 Hz, 1H), 5.02 (s, 1H), 3.96 (s, 2H), 1.28 (s, 9H), 1.14 (s, 9H).

Note: Missing one phenol or amine proton.

^{13}C{^{1}H}-NMR (151 MHz, DMSO-*d*$_6$) δ 154.4, 150.0, 143.9 (q, *J* = 34.3 Hz), 140.7, 138.2, 138.1, 126.1, 125.1, 124.0, 122.3, 121.9 (q, *J* = 273.6 Hz), 120.1 (q, *J* = 3.3 Hz), 50.7, 45.4, 33.8, 31.4, 27.7.

^{19}F{^{1}H}-NMR (376 MHz, DMSO-*d*$_6$) δ –66.2.

HRMS (ESI-FTICR): *m/z* = 381.2148 calcd for $C_{21}H_{28}F_3N_2O^+$ [M+H]$^+$, found 381.2141.

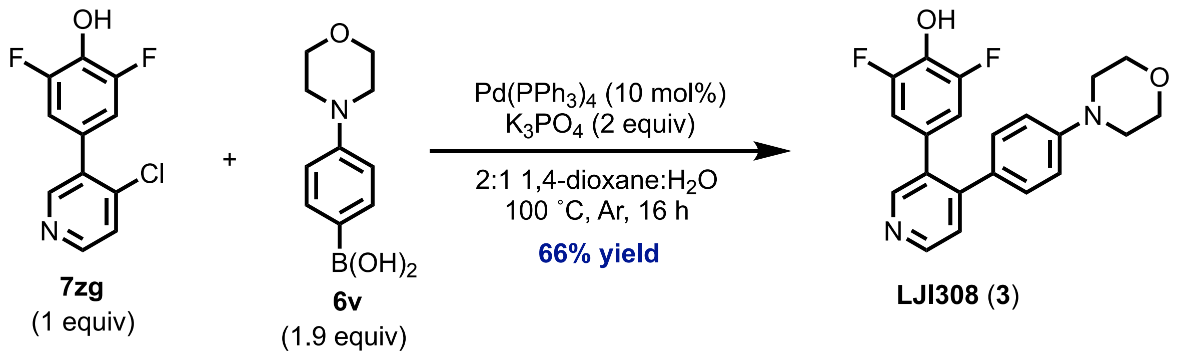

2,6-Difluoro-4-(4-(4-morpholinophenyl)pyridin-3-yl)phenol (LJI308) (**3**)

To an 8 mL microwave vial equipped with a magnetic stir bar was charged 4-(4-chloropyridin-3-yl)-2,6-difluorophenol (**7zg**, 20 mg, 1.0 equiv, 83 µmol), (4-morpholinophenyl)boronic acid (**6v**, 33 mg, 1.9 equiv, 0.16 mmol), and tetrakis(triphenylphosphine)palladium(0) (9.6 mg, 10 mol%, 8.3 µmol). The vial was crimp-capped and sparged with argon for 5 min prior to the addition of dry 1,4-dioxane (1.0 mL) and a solution of potassium phosphate, tribasic (35 mg, 2.0 equiv, 0.17 mmol) in degassed water (0.5 mL). The reaction mixture was heated to 100 ˚C. LCMS analysis of the reaction mixture after 16 h indicated the presence of the desired mass. The reaction mixture was dry loaded onto Celite™ and purified by reverse-phase column chromatography (55% MeCN:H_2O-0.1% FA) to afford the title compound **3** (LJI308) as an amorphous yellow solid (20 mg, 54 µmol, 66% yield).

^{1}H-NMR (400 MHz, DMSO-*d*$_6$) δ 10.41 (s, 1H), 8.54 (d, *J* = 5.1 Hz, 1H), 8.50 (s, 1H), 7.37 (d, *J* = 5.1 Hz, 1H), 7.06 (d, *J* = 8.8 Hz, 2H), 6.90 (d, *J* = 8.9 Hz, 2H), 6.84 (d, *J* = 8.7 Hz, 2H), 3.73 – 3.69 (m, 4H), 3.15 – 3.11 (m, 4H). Spectral data were in agreement with those reported.[8]

9. UV-Vis, Fluorescence, and Quantum Yield Experiments

UV-Vis Analysis

UV-Vis measurements were obtained on a Thermo Scientific™ GENESYS™ 150 spectrophotometer using a 1.0 mL quartz cuvette at ambient temperature and measurement range of 275–600 nm. Stock solutions of each reagent (0.10 M phenol **5a**, 0.10 M pyridine **6a**) were prepared in 9:1 MeCN:H_2O with 1 equiv Na_2CO_3, each was analyzed. A 1:1 mixture of phenol **5a** and pyridine **6a** were analyzed to probe EDA complex formation. The data was directly processed in Microsoft Excel after export from the instrument. A slight bathochromic shift is seen with the 1:1 mixture alongside a color change; however, the shift is not significant. The UV-Vis is therefore not definitive proof of EDA. The significance of this finding is discussed in the manuscript.

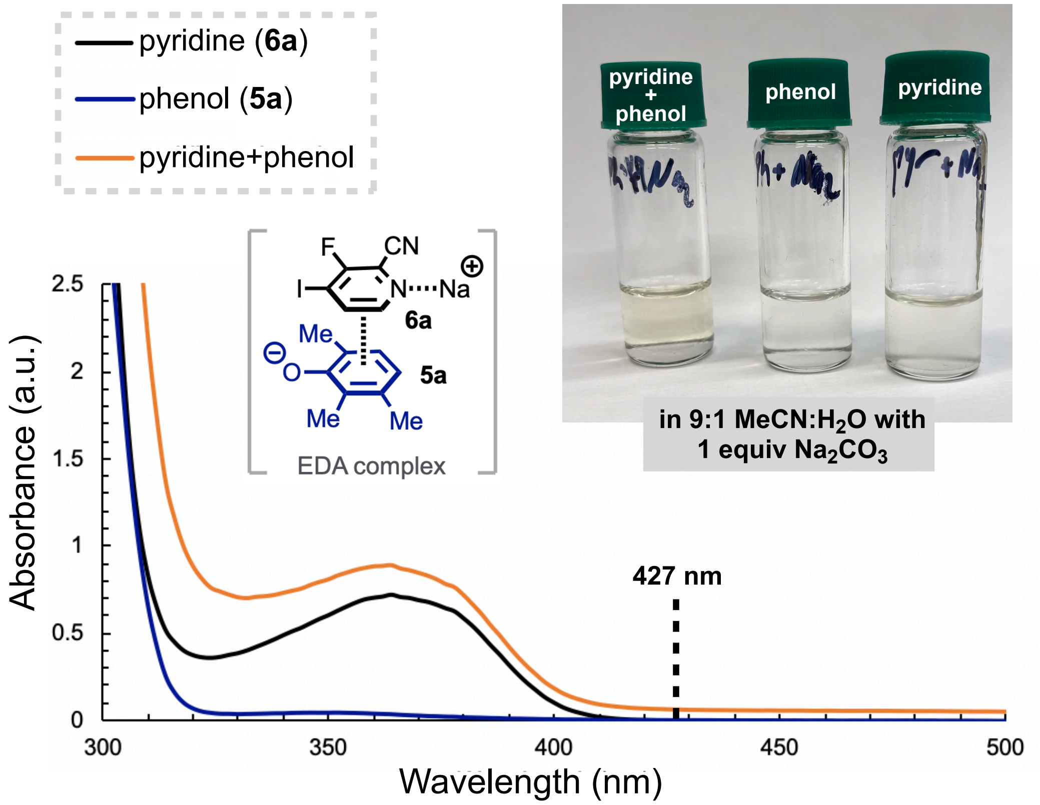

Figure S6. UV-Vis spectra for 2,3,6-trimethylphenol (**5a**) (0.10 M), 3-fluoro-4-iodopicolinonitrile (**6a**) (0.10 M), and their 1:1 mixture. a.u. = arbitrary units

Fluorescence Analysis

Steady-state fluorescence quenching measurements were obtained using a QuantaMaster™ 300 by HORIBA Scientific photoluminescence spectrometer with a pulsed Xe arc lamp at ambient temperature and the following parameters: λ_{ex} = 380 nm, emission measurement range = 400–650 nm, step size = 1 nm, integration = 0.3 sec, excitation/emission enter slit width = 1 nm, excitation/emission exit slit width = 5 nm. For each quenching experiment, a solution of 3DPA2FBN (15 μM in degassed MeCN) was measured (blank), followed by solutions of 3DPA2FBN (15 μM) with different concentrations of the specified quencher (5 mM, 15 mM, 30 mM) dissolved in degassed MeCN. All solutions were sparged with argon for 10 minutes prior to analysis. λ_{max} was observed ~485 nm for all the measurements. The data was processed in Microsoft Excel after exportation and plots were modeled according to the Stern-Volmer equation:

$$I_o/I = 1 + k_q t_0 \bullet [\text{quencher}],\ k_{SV} = k_q t_0$$

(k_q is the quencher rate coefficient, t_0 is the lifetime of excited state of photocatalyst, I_0 and I are the luminescence intensities in the absence and presence of each respective quencher at varying concentrations, and k_{SV} is the Stern-Volmer constant (M^{-1}). Data for each concentration is an average of three consecutive runs.

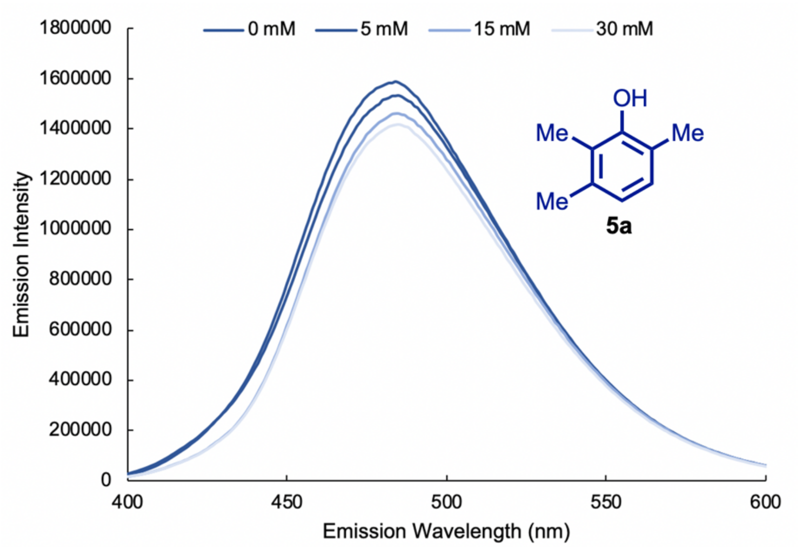

Figure S7. Fluorescence response of 3DPA2FBN (15 μM in degassed MeCN) upon titration with varying concentrations (0–30 mM) of 2,3,6-trimethylphenol (**5a**).

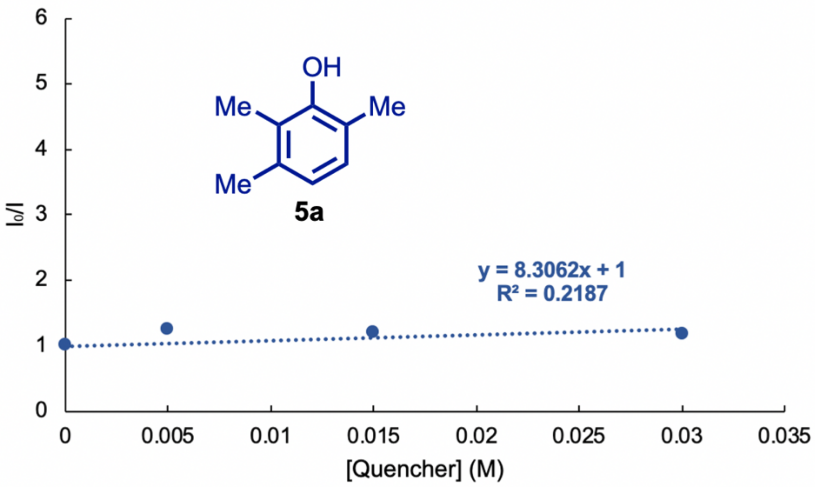

Figure S8. Stern-Volmer plots for 2,3,6-trimethylphenol (**5a**) using 3DPA2FBN in MeCN.

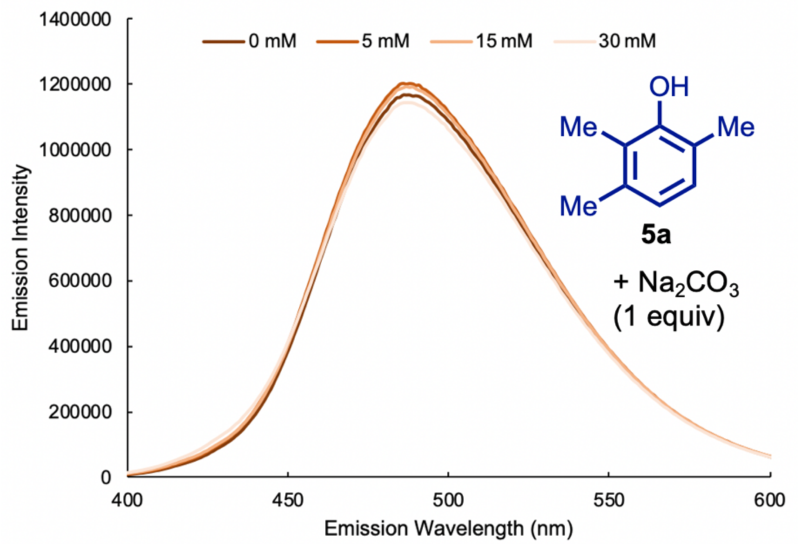

Figure S9. Fluorescence response of 3DPA2FBN (15 μM in degassed MeCN) upon titration with varying concentrations (0–30 mM) of 2,3,6-trimethylphenol (**5a**) and 1.0 equiv of 1.0 M aqueous Na_2CO_3.

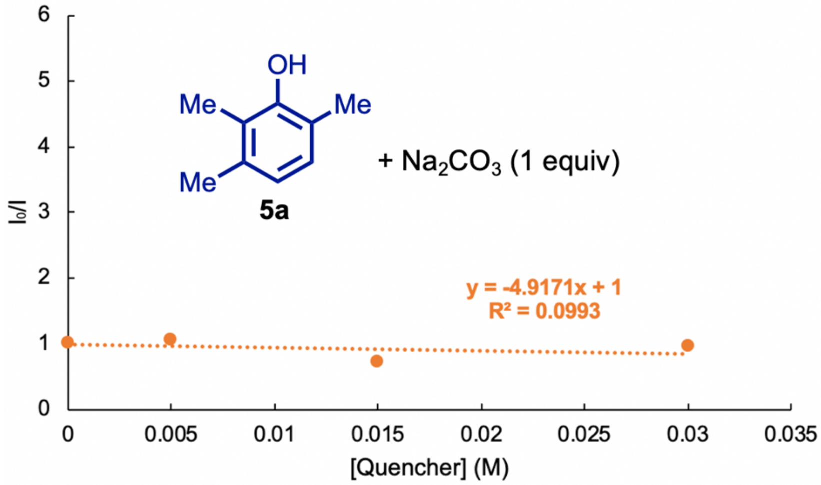

Figure S10. Stern-Volmer plots for 2,3,6-trimethylphenol (**5a**) and 1.0 equiv of 1.0 M aqueous Na_2CO_3 using 3DPA2FBN in MeCN.

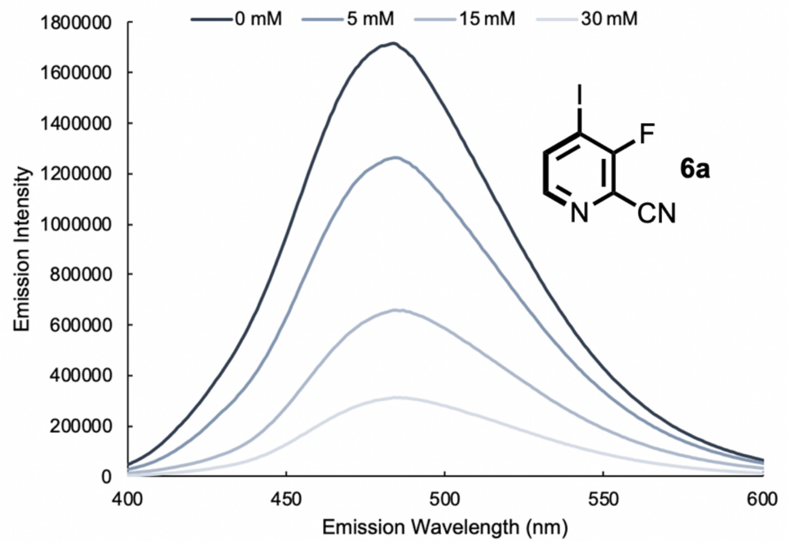

Figure S11. Fluorescence response of 3DPA2FBN (15 μM in degassed MeCN) upon titration with varying concentrations (0–30 mM) of 3-fluoro-4-iodopicolinonitrile (**6a**).

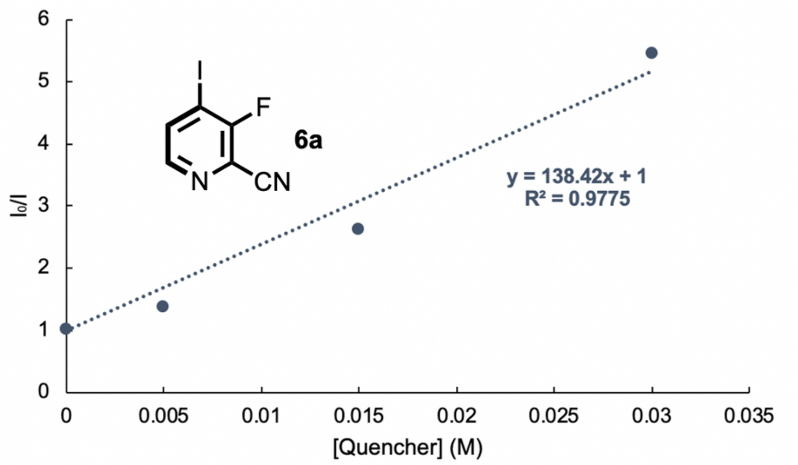

Figure S12. Stern-Volmer plots for 3-fluoro-4-iodopicolinonitrile (**6a**) using 3DPA2FBN in MeCN.

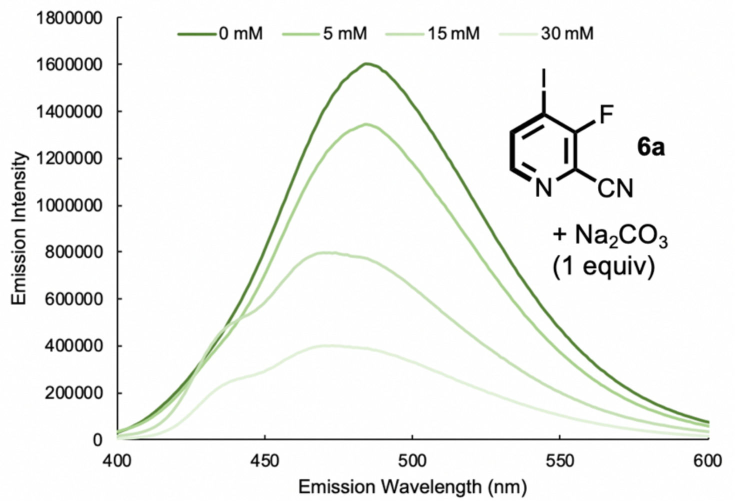

Figure S13. Fluorescence response of 3DPA2FBN (15 μM in degassed MeCN) upon titration with varying concentrations (0–30 mM) of 3-fluoro-4-iodopicolinonitrile (**6a**) and 1.0 equiv of 1.0 M aqueous Na_2CO_3.

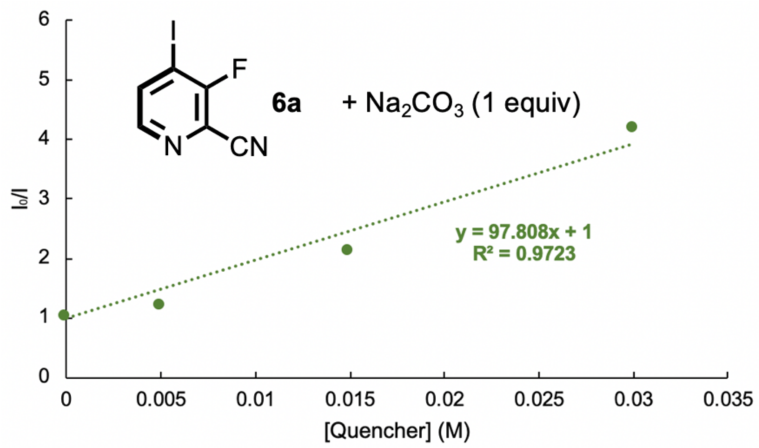

Figure S14. Stern-Volmer plots for 3-fluoro-4-iodopicolinonitrile (**6a**) and 1.0 equiv of 1.0 M aqueous Na_2CO_3 using 3DPA2FBN in MeCN.

Quantum Yield

The following procedure was adapted from the literature[9]:

The photon flux of the 427 nm Kessil setup was determined using standard ferrioxalate actinometry. A 0.15 M ferrioxalate solution was prepared by dissolving potassium ferrioxalate trihydrate (2.21 g) in 0.05 M aqueous H_2SO_4 (30 mL). A buffered phenanthroline solution was prepared by dissolving 1,10-phenanthroline (50 mg) and NaOAc•3H_2O (11.3 g) in 0.50 M aq. H_2SO_4 (50 mL). Both solutions were stored in amber bottles in the dark. Whilst working in a dark fume hood (lights off in lab), 1.0 mL of the 0.15 M ferrioxalate solution in a 8 mL microwave vial was positioned in front of the 427 nm Kessil PR160L lamp (100% intensity, max 45W, 7 cm from wall of flask, no filters). The vial was then irradiated for 20 s and 0.50 mL of the 1,10-phenanthroline solution was quickly added. This sequence was repeated for a second vial of ferrioxalate solution, irradiated for 30 s. A third vial of ferrioxalate solution was also prepared, to serve as a time = 0 s sample (no irradiation). Each mixture was wrapped in tin foil and left to stand for approximately 30 min before the absorbance at λ = 510 nm was measured by UV-Vis spectroscopy. The number of moles of Fe^{2+} formed was calculated using:

$$\text{mol Fe}^{2+} = \frac{V \times \Delta A}{l \times \varepsilon}$$

where V is the total volume of the solution after the addition of 1,10-phenanthroline (0.0015 L), ΔA is the difference in absorbance at λ = 510 nm between the irradiated and non-irradiated ferrioxalate solutions, l is the optical path length of the irradiation cell (1.0 cm), and ε is the molar absorptivity of the $Fe(phen)_3^{2+}$ complex at λ = 510 nm (11,100 L mol^{-1} cm^{-1}). The moles of Fe^{2+} were plotted as a function of time (**Figure S15**).

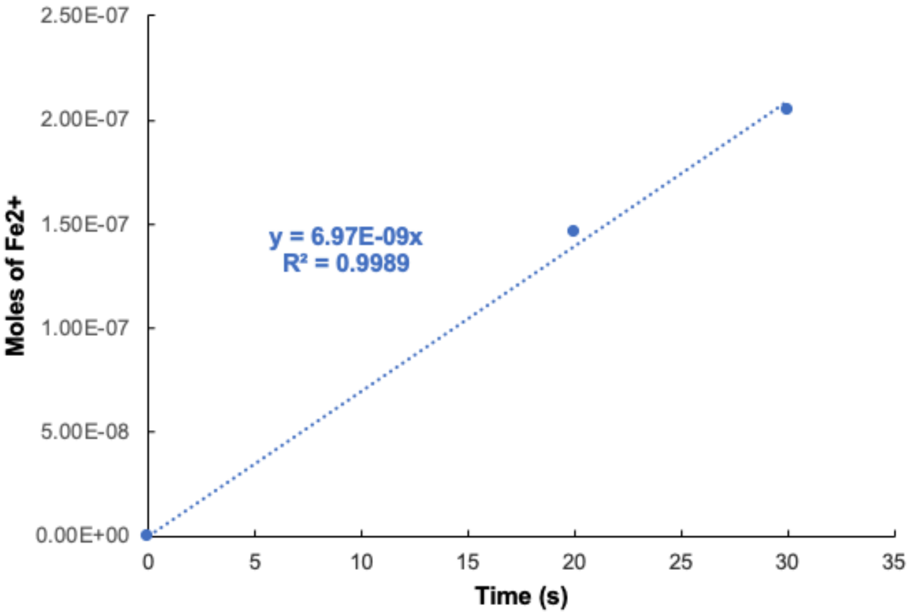

Figure S15. The moles of Fe^{2+} in the ferrioxalate actinometry plotted as a function of time (s).

The photon flux of the 427 nm Kessil PR160L lamp was then calculated using:

$$\textit{photon flux} = \frac{mol\ Fe^{2+}}{\Phi \times t \times f}$$

where Φ is the quantum yield of the ferrioxalate actinometer (1.1 at λ = 427 nm, t is the time (s), and f is the fraction of absorbed light at λ = 427 nm, where $f = 1 - 10^{-A}$. The absorbance (A) of the ferrioxalate solution at λ = 427 nm was measured by UV-Vis spectroscopy to be 3.65, so f = 0.9998.

$$\textit{photon flux} = \frac{6.97 \times 10^{-9}\ \text{mol}}{1.1 \times t\ (s) \times 0.9998} = \underline{\mathbf{6.33 \times 10^{-9}\ mol\ s^{-1}}}$$

The yield of **7a** for a standard 0.10 mmol reaction (photocatalytic) from general procedure B after 10 min (600 s) was determined by $^{19}F\{^{1}H\}$-NMR using a trifluorotoluene internal standard (12 µL, 1.0 equiv). The average yield was determined to be 11.6% (1.16×10^{-5} mol) after two trials. The yield of **7a** for a standard reaction without photocatalyst (EDA) after 20 min (1200 s) was also determined with this method to be 2.2% (2.04×10^{-6} mol). The quantum yield (Φ) for each cycle was then calculated using:

$$\Phi = \frac{mol\ product}{photon\ flux \times t \times f}$$

where t is the reaction time (s), f is the fraction of light absorbed by the 3DPA2FBN catalyst at λ = 427 nm, where $f = 1 - 10^{-A}$ (for a 0.0030 M solution in MeCN, A = 3.49, so f = 0.9997) or f is the fraction of light absorbed by the EDA complex (**5a•6a**) at λ = 427 nm, where $f = 1 - 10^{-A}$ (for a 0.10 M solution in MeCN, A = 0.066, so f = 0.1416), and *photon flux* for the Kessil setup is calculated as shown above.

$$\Phi\ (photocatalytic) = \frac{1.16 \times 10^{-5}\ \text{mol}}{6.33 \times 10^{-9}\ \text{mol s}^{-1} \times 600\ s \times 0.9997} = \underline{\mathbf{3.06}}$$

$$\Phi\ (EDA) = \frac{2.04 \times 10^{-6}\ \text{mol}}{6.33 \times 10^{-9}\ \text{mol s}^{-1} \times 1200\ s \times 0.1416} = \underline{\mathbf{1.90}}$$

The significance of this result is addressed in the main manuscript.

10. Mechanism Proposals

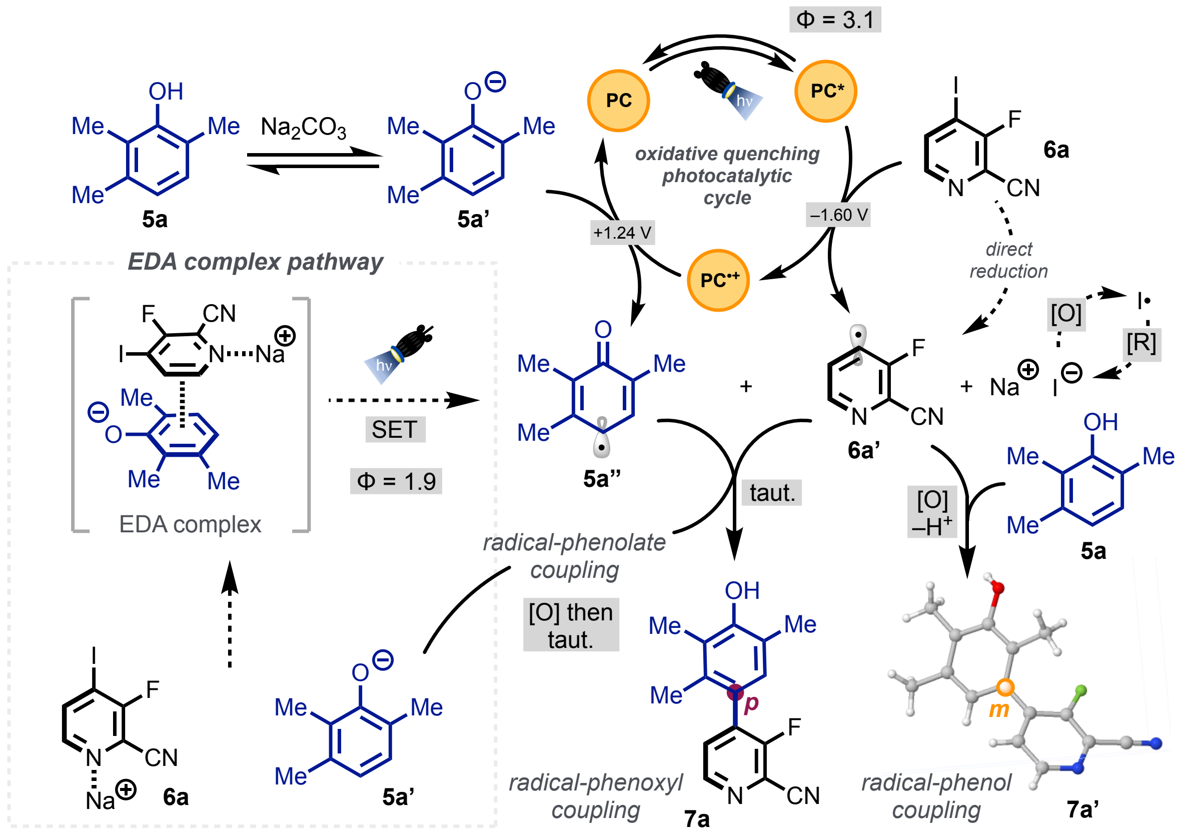

Figure S16. Proposed reaction mechanism involving electron donor-acceptor complexation as well as photocatalytic cycle.

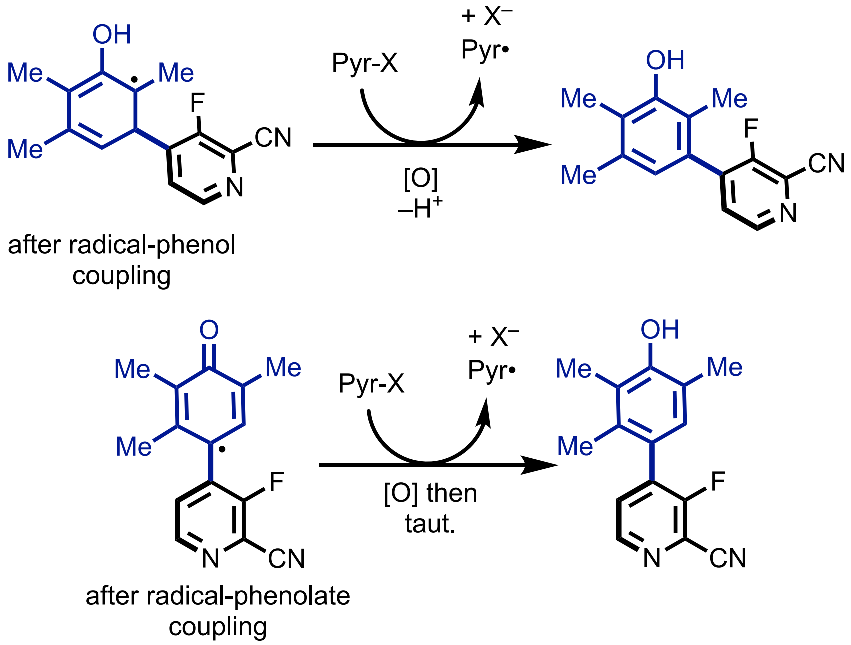

Figure S17. Potential chain mechanism for post-radical addition intermediates..

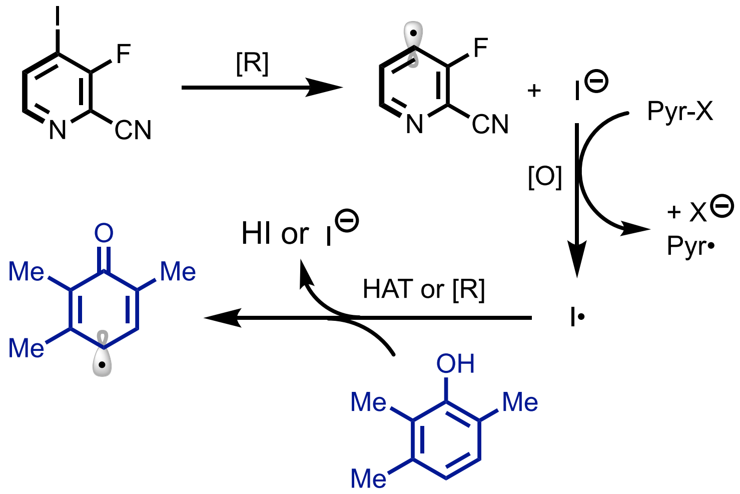

Figure S18. Potential chain mechanism involving iodide anion.

11. High-Throughput Experimentation (HTE)

General Information

Liquid solutions were dosed manually via the use of a multi-channel repeater pipette. Reactions were stirred on a tumbler stirrer (V&P Scientific) using 1.98 (d) x 4.80 (l) mm parylene-coated stir bars (V&P Scientific). Reaction vials were sealed in a standard 96-Position Photoredox Reaction Block (Analytical Sales and Services) prior to tumble stirring at 400 rpm. A 0.062 mm thick rubber gasket was placed across the bottom of the plate while a PFA film and two rubber mats (Analytical Sales and Services) were placed on top of the vials prior to sealing. The entire assembly was compressed between an aluminum top and the reactor base with nine evenly placed screws.

The reaction plate was prepared with the appropriate solutions and sealed in the glovebox and the plate was irradiated at 445 nm using a Lumidox® II 96-Well LED Array (**Figure S19**) with tumble stirring while chilled to room temperature. 50 mW per well was delivered using LUM2CON Lumidox® II LED Controller. After 16 h, each well of the plate was diluted with 4,4'-di-*tert*-butylbiphenyl internal standard in MeCN (500 µL, 0.0020 M) and centrifuged to sediment debris. An aliquot (25 µL) from each vial was added diluted in a fresh vial with additional MeCN (700 µL) to form the the LC plate. The mixtures were analyzed by LCMS (see General Information and Materials for details) and the normalized ratios of product to internal standard (comparing the absorbance chromatogram at 254 nm) were obtained.

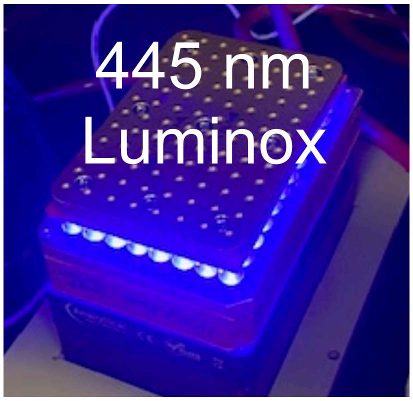

Figure S19. Lumidox® II 96-Well LED Array used for this study.

General Procedure for 96-Well HTE PC and Solvent Screen

Individual solutions of **heteroaryl halide (maroon)** (0.33 mmol, 0.30 M) [solution A], **phenol (blue)** (0.5 mmol, 0.45 M) [solution B], and photocatalysts [3DPA2FBN, 3CzEPAIPN] (32 μmol, 0.010 M) were prepared in dry MeCN and DMSO, respectively. Aqueous solutions of Na_2CO_3 (1.06 g, 10 mL, 1.0 M) were also prepared. The 1.0 mL reactor vials of a 96-well plate were dosed with solution A (300 μL), solution B (300 μL), photocatalyst solution (300 μL), and Na_2CO_3 solution (150 μL) according to a predetermined map for the screen shown below in **Figure S20**.

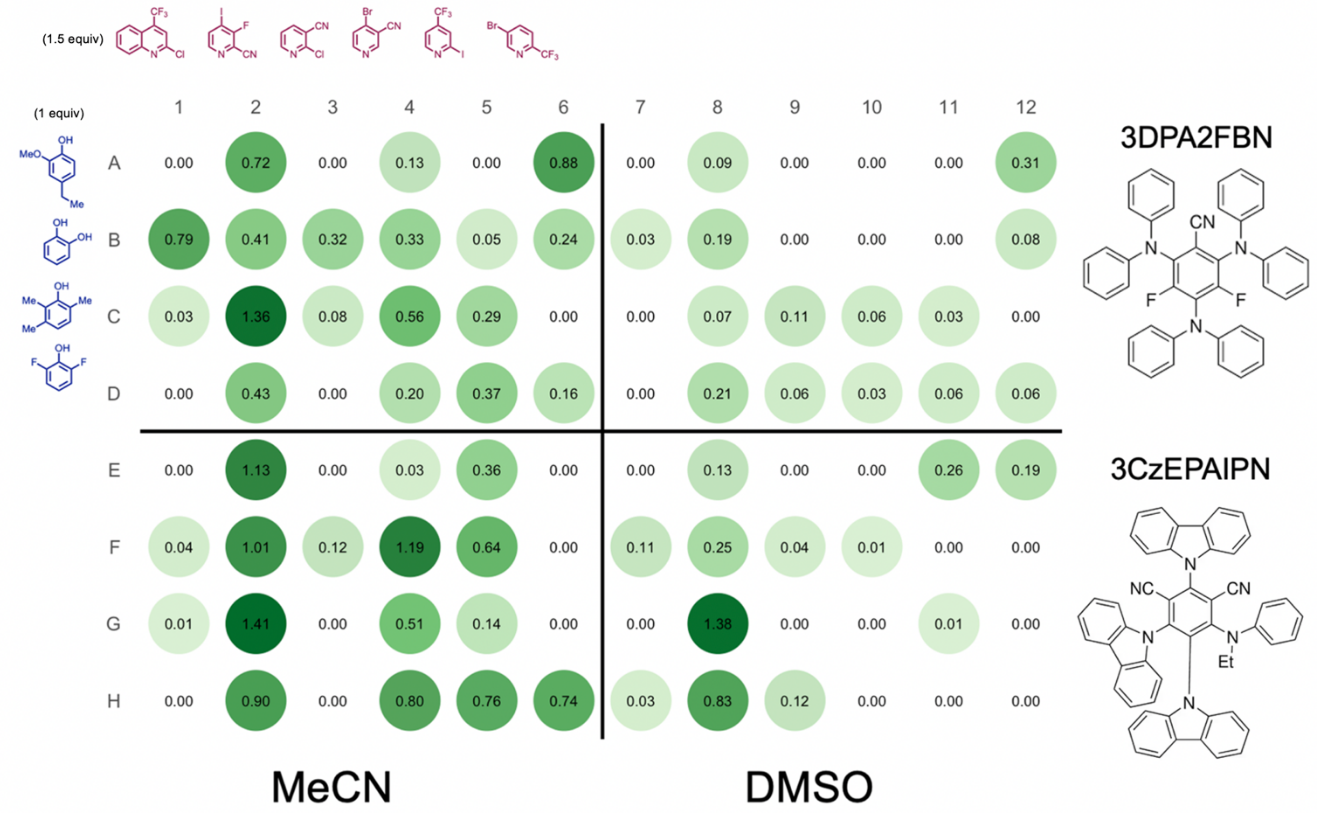

Figure S20. 96-well HTE screen with photocatalyst [3DPA2FBN, 3CzEPAIPN] and solvent [MeCN, DMSO]. Product to 4,4'-di-*tert*-butylbiphenyl internal standard ratios shown (254 nm).

General Procedure for 96-Well HTE Substrate Scope Screen

Individual solutions of **6a**, **6c**, **6k–p** (0.15 mmol, 0.30 M) [solution C], **5f–q** (0.18 mmol, 0.45 M) [solution D], and photocatalyst 3DPA2FBN (33 μmol, 21 mg, 0.010 mM) were prepared in dry MeCN. Previously prepared batch solution of Na_2CO_3 (1.0 M) was used. The 1.0 mL reactor vials of a 96-well plate were dosed with solution C (300 μL), solution D (300 μL), photocatalyst solution (300 μL), and Na_2CO_3 solution (150 μL) according to a predetermined map for the screen shown below in **Figure S21**.

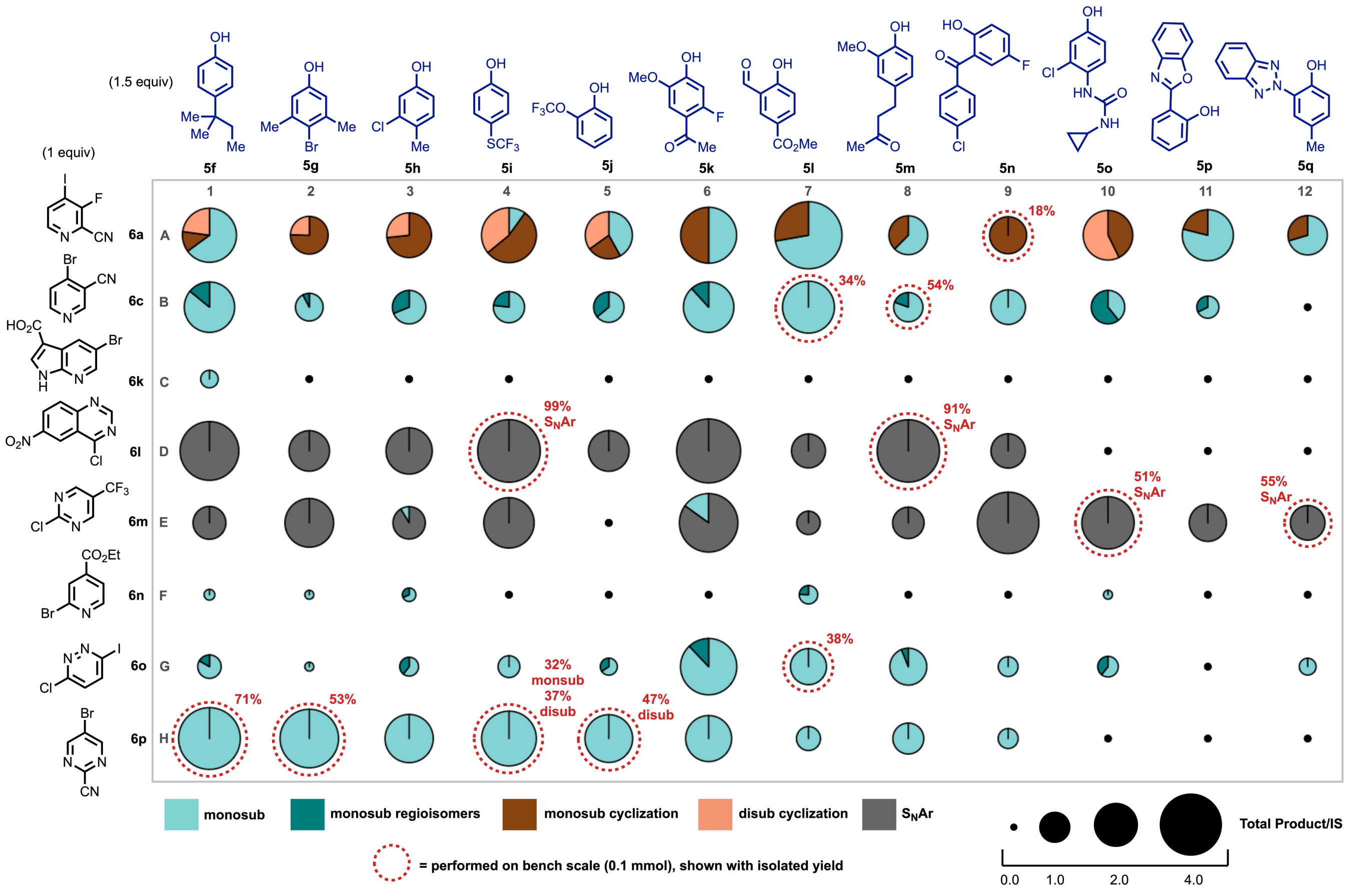

Figure S21. 96-well HTE screen for substrate scope with phenols **5f**–**q** and heteroaryl halides **6a**–**p**. Product to 4,4'-di-*tert*-butylbiphenyl internal standard ratios shown (254 nm). Isolated yields (0.10 mmol) shown in red.

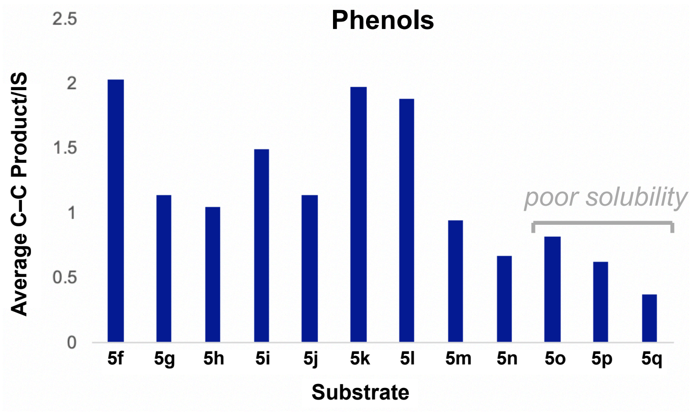

Figure S22. Average C–C product formation for each phenol tested in 96-well plate (n = 8).

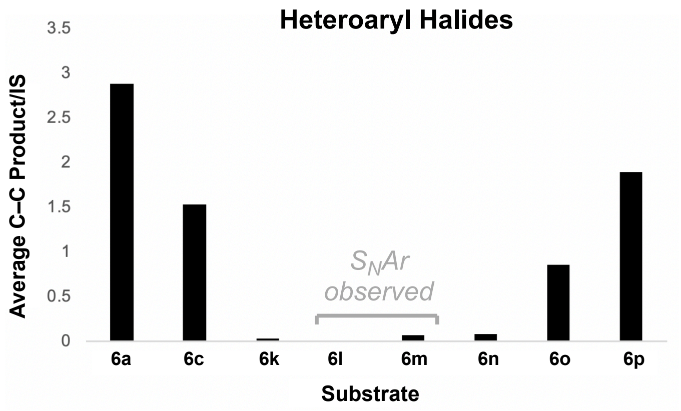

Figure S23. Average C–C product formation for each heteroaryl halide tested in 96-well plate (n = 12).

12. UMAP Substrate Clustering

The below search inquiries were utilized to gather the data on commercially available phenols and halogenated azines used in the UMAP plots.

Date: 05/24/2025 and 05/30/2025
Database: SciFinder
Search Query:

- Substructures

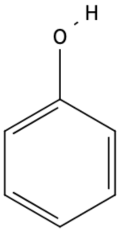

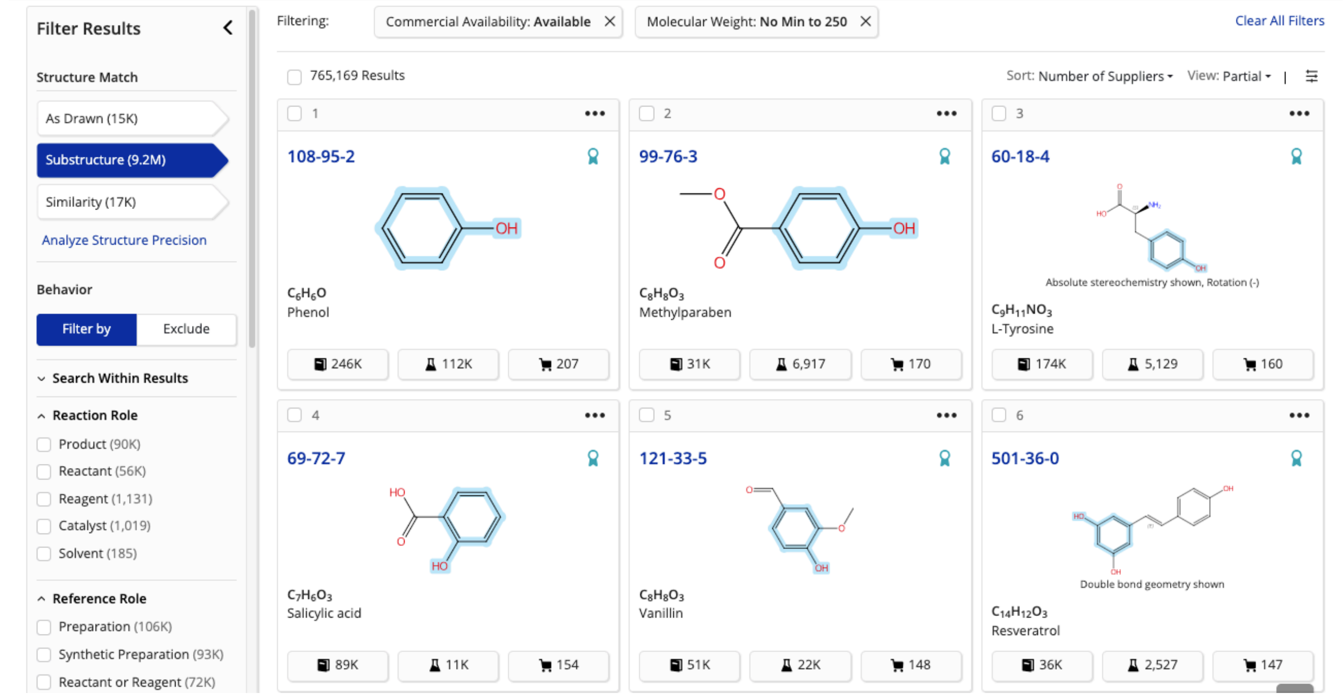

Figure S24. Search results for phenol substructures.

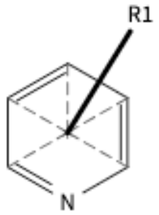

R1 = Cl, Br, I

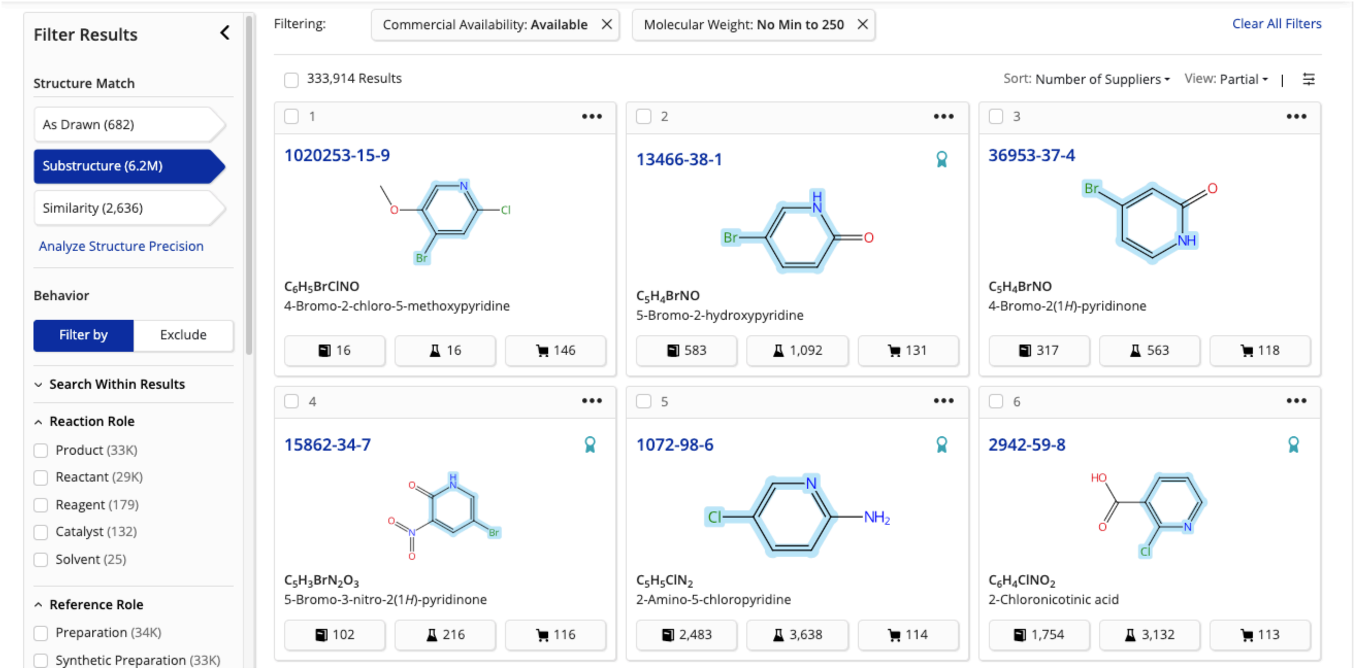

Figure S25. Search results for *N*-heteroaryl halide substructure.

General Procedures

Software versions:
Python version: 3.12.12 (main, Oct 10 2025, 08:52:57) [GCC 11.4.0]
pandas[10] version: 2.2.2
numpy version: 2.0.2
rdkit version: 2025.09.3
scikit-learn version: 1.6.1
umap-learn version: 0.5.9.post2
bokeh version: 3.7.3

For the full set of phenols and heteroarenes obtained above, RDKit 2D descriptors (excluding fragment-based descriptors) were generated. Highly correlated descriptors (Pearson correlation above 0.90) and descriptors with missing values were dropped. Next, a 2D UMAP plot was generated for each of the datasets (n_components=2, metric='cosine', random_state=42). These plots are shown in **Figures S26** and **S27**. Next, agglomerative clustering was used to split the phenols and heteroarenes into 17 clusters (arbitrarily selected based on visual examination fo clusters being distinct). The clusters are indicated by color in **Figures S26** and **S27**. The clusters were manually examined and representative phenols and heteroaryl halides were selected out of unique clusters to use in the substrate screen based on commercial availability and utility. CSV file of the data sets are attached with the Supporting Information.

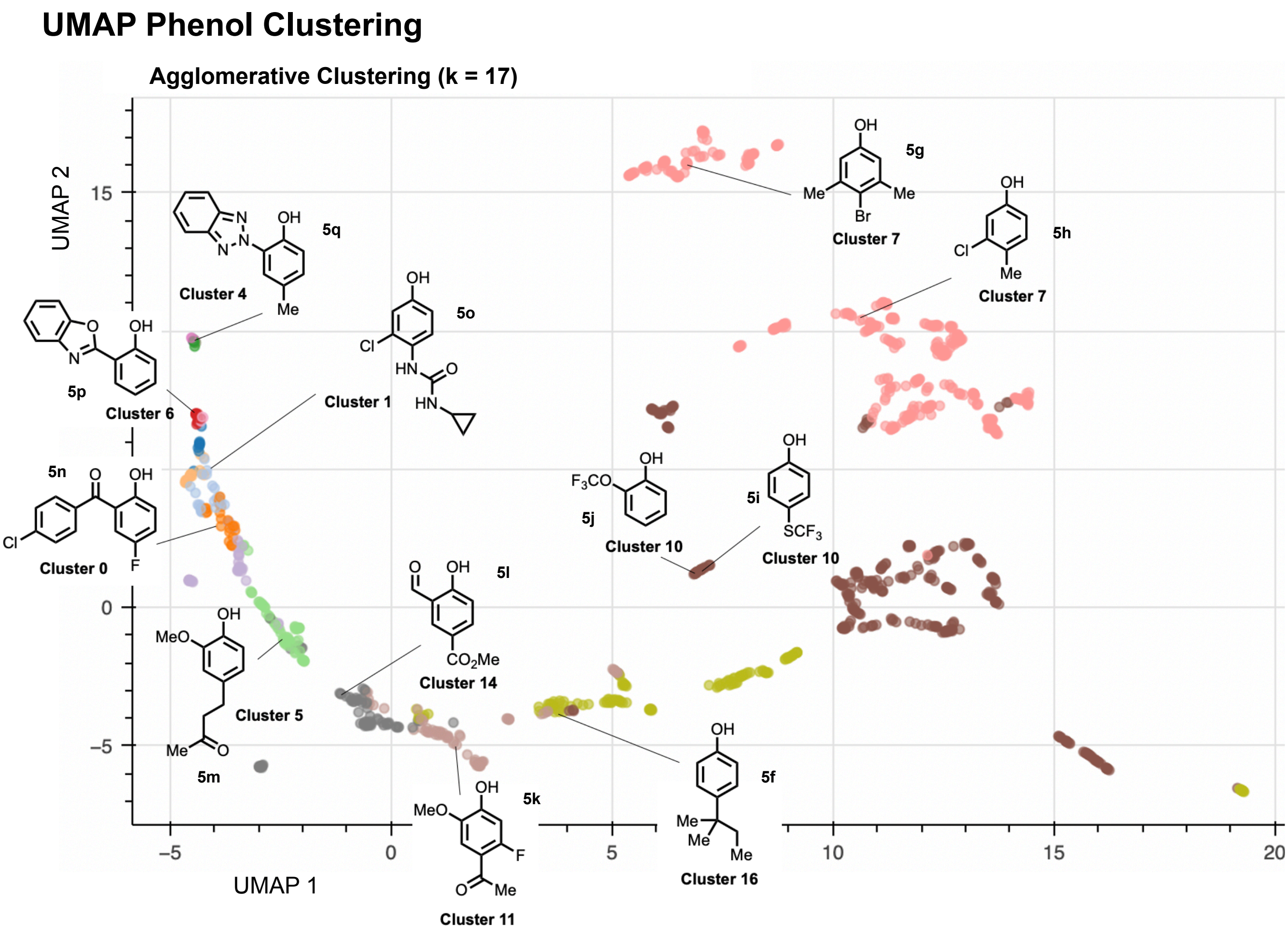

Figure S26. UMAP clustering of phenol chemical space with associated cluster number shown.

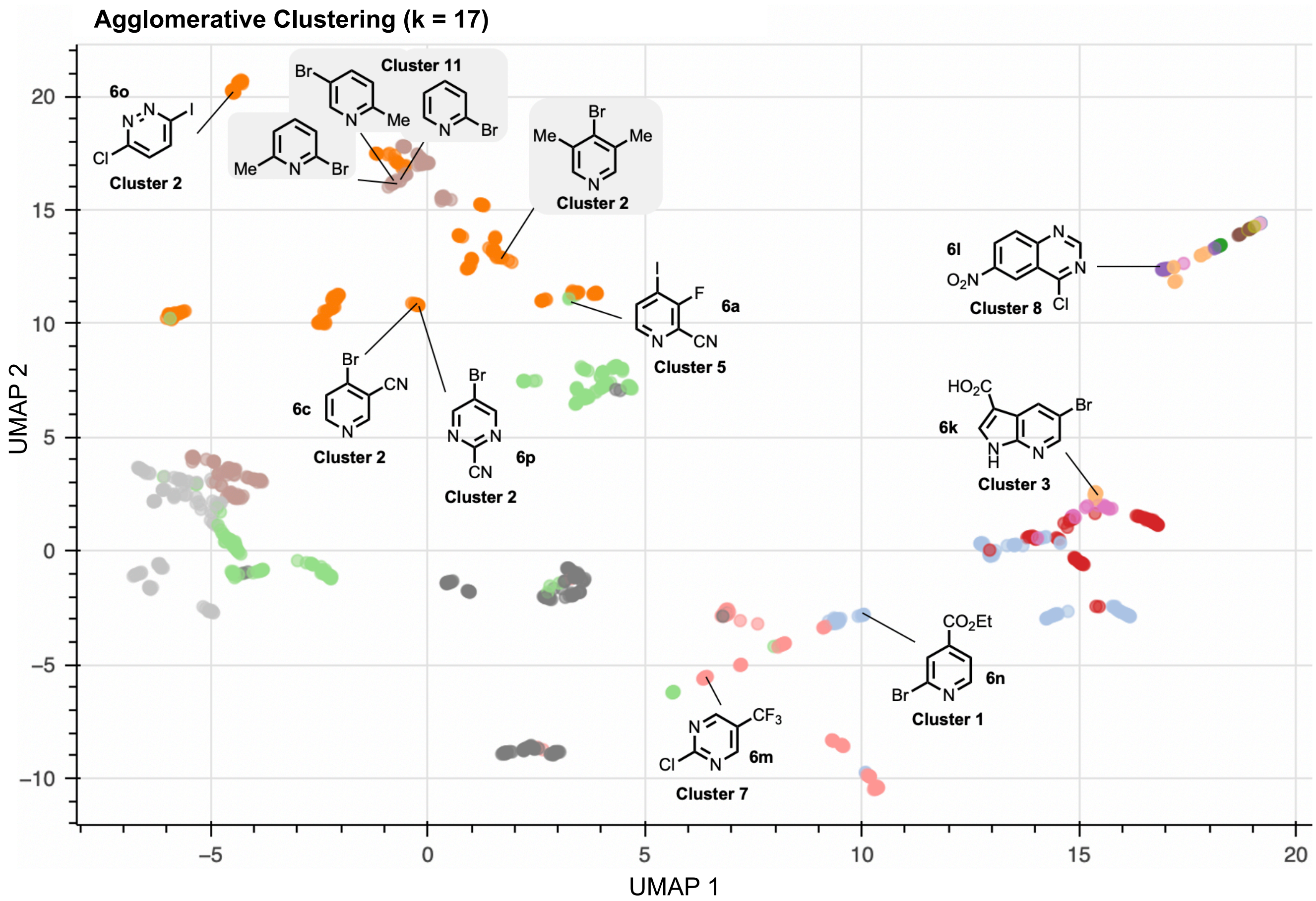

Figure S27. UMAP clustering of heteroaryl halide chemical space with associated cluster number shown. Compounds highlighted in grey are sourced from literature.[2]

13. Machine Learning

General Information

Python version: 3.7.15 (default, Nov 24 2022, 12:02:37)
[Clang 14.0.6]
pandas[10] version: 1.3.5
numpy version: 1.21.5
seaborn[11] version: 0.12.1
matplotlib[12] version: 3.5.3
scikit-learn version: 1.0.2

For each reaction, the following parameters were calculated using *Rowan Scientific* online platform[13] (https://rowansci.com/) or DFT[7]:

- oxidation potential of phenol[13] (V vs SCE)
    - Redox potential prediction
    - Redox Type: Oxidation
    - Mode: Rapid
- global electrophilicity of phenol[13] (eV)
    - Fukui index calculation
    - Optimization Level of Theory: GFN2-xTB
    - Fukui Level of Theory: GFN1-xTB
    - Solvent: None
- radical electrophilicity of phenol[7] (eV)
- pK_a of phenol[13]
    - Microscopic pK_a prediction
    - Method: Rowan pK_a (Wagen 2024) – NNP-Based Aqueous pK_a
    - Min pK_a: 2; Max pK_a: 12
    - Protonate Elements: N; Deprotonate Elements: N–O, S
    - Solvent: Water
    - Mode: Careful
- reduction potential of heteroaryl halide[13] (V vs SCE)c
    - Redox potential prediction
    - Redox Type: Reduction
    - Mode: Rapid
- global electrophilicity of heteroaryl halide[13] (eV)
    - Fukui index calculation
    - Optimization Level of Theory: GFN2-xTB
    - Fukui Level of Theory: GFN1-xTB
    - Solvent: None
- radical electrophilicity of heteroaryl halide[7] (eV)
- positive fukui index of heteroaryl halide[13]
    - Fukui index calculation
    - Optimization Level of Theory: GFN2-xTB
    - Fukui Level of Theory: GFN1-xTB
    - Solvent: None

Reactions that had more C–C coupling product than S_NAr product were deemed as "C–C more likely" and reactions that had more S_NAr product than C–C products were deemed as "S_NAr more likely". All reactions that produced no C–C coupling or S_NAr product were discarded, and the remaining reactions were split randomly into a training set (81 reactions) and a test set (21 reactions). Correlated parameters (Pearson correlation above 0.9) were removed, and the data was scaled using StandardScaler from SciKitLearn. A logistic regression was fitted on the training set, and cross validation was performed. An accuracy of 89% with a standard deviation of 9% was obtained, establishing consistency in the model. The model was then evaluated on the test set. An accuracy of 86% was obtained, with an F1 score of 0.77 for S_NAr prediction and 0.90 for C–C prediction, indicating that the model is adept at predicting both classes. The selected features and coefficients are shown in **Table S6**. The model was also successful at predicting the major product in a set of three external reactions that were run on benchtop. CSV file of the data set is attached with the Supporting Information.

Table S6. Selected Features in Logistic Regression.

Feature	Coefficient
Heteroaryl Halide Radical Electrophilicity	1.887118
Heteroaryl Halide Reduction Potential	0.252103
Phenol pK_a	–0.37868
Phenol Global Electrophilicity	–0.63239
Heteroaryl Halide Global Electrophilicity	–1.93414

Zero corresponds to S_NAr product and 1 corresponds to C–C product, so positive coefficients are predictive of C–C reactivity.

While not the most accurate, logistic regression model with five features (entry 17) was chosen as it is linear which allows one to know exactly in what way each feature is used, so the model is very interpretable. One can gain some chemical insights from knowing exactly what feature does what. It is also less prone to overfitting and computationally faster than other models.

Model comparison (cross-validated accuracy):

	Model	Top_N_Features	Mean_CV_Accuracy	Std_CV_Accuracy
0	Decision Tree	3	0.975735	0.029741
1	KNN	3	0.975735	0.029741
2	Random Forest	4	0.975735	0.029741
3	Decision Tree	4	0.975735	0.029741
4	Random Forest	3	0.975735	0.029741
5	Gradient Boosting	3	0.951471	0.044447
6	Random Forest	5	0.951471	0.044447
7	Gradient Boosting	4	0.950735	0.046597
8	Random Forest	6	0.939706	0.037247
9	Decision Tree	5	0.928676	0.086365
10	Decision Tree	6	0.928676	0.086365
11	Gradient Boosting	6	0.927206	0.069539
12	Decision Tree	7	0.916176	0.079378
13	Random Forest	7	0.916176	0.059436
14	Gradient Boosting	7	0.915441	0.060634
15	Gradient Boosting	5	0.915441	0.060634
16	Logistic Regression	3	0.904412	0.069872
17	Logistic Regression	5	0.892647	0.085667
18	Logistic Regression	7	0.880147	0.082720
19	SVM (RBF)	5	0.867647	0.066867
20	Logistic Regression	6	0.867647	0.087156
21	KNN	4	0.866912	0.097003
22	SVM (RBF)	3	0.866912	0.070004
23	Logistic Regression	4	0.855147	0.070089
24	SVM (RBF)	7	0.855147	0.080467
25	KNN	5	0.842647	0.104567
26	SVM (RBF)	4	0.842647	0.058980
27	KNN	6	0.842647	0.058980
28	SVM (RBF)	6	0.842647	0.058980
29	KNN	7	0.830147	0.069694

Figure S28. Machine learning models tested and their associated cross-validation accuracies and standard deviations.

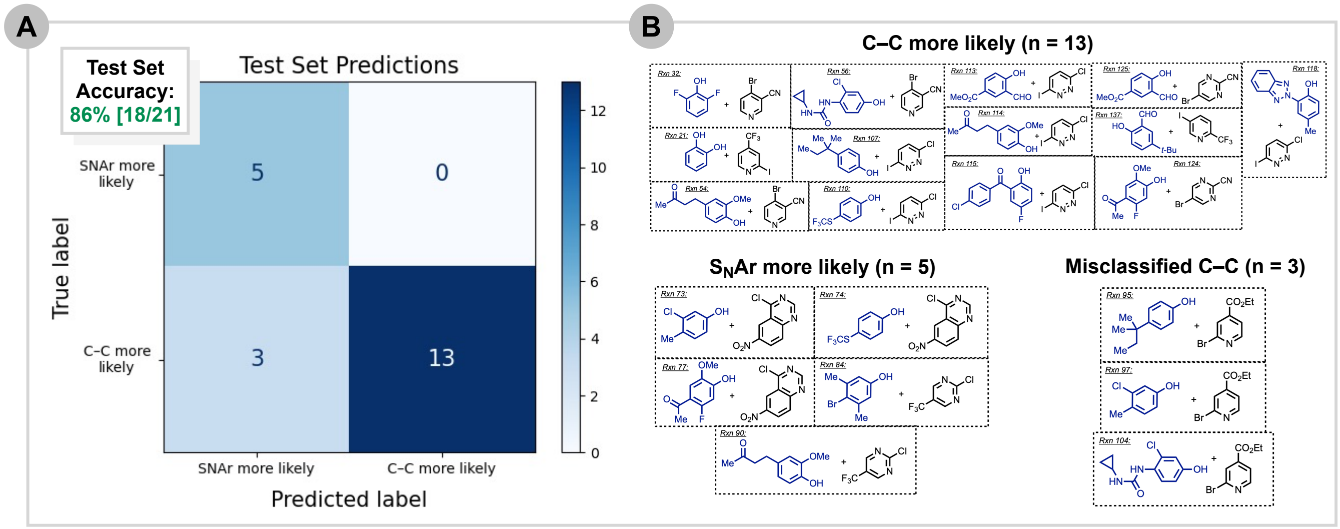

Figure S29. a) Confusion matrix of logistic regression model for random test set (n = 21). b) Starting materials for each of the test set reactions and their associated prediction.

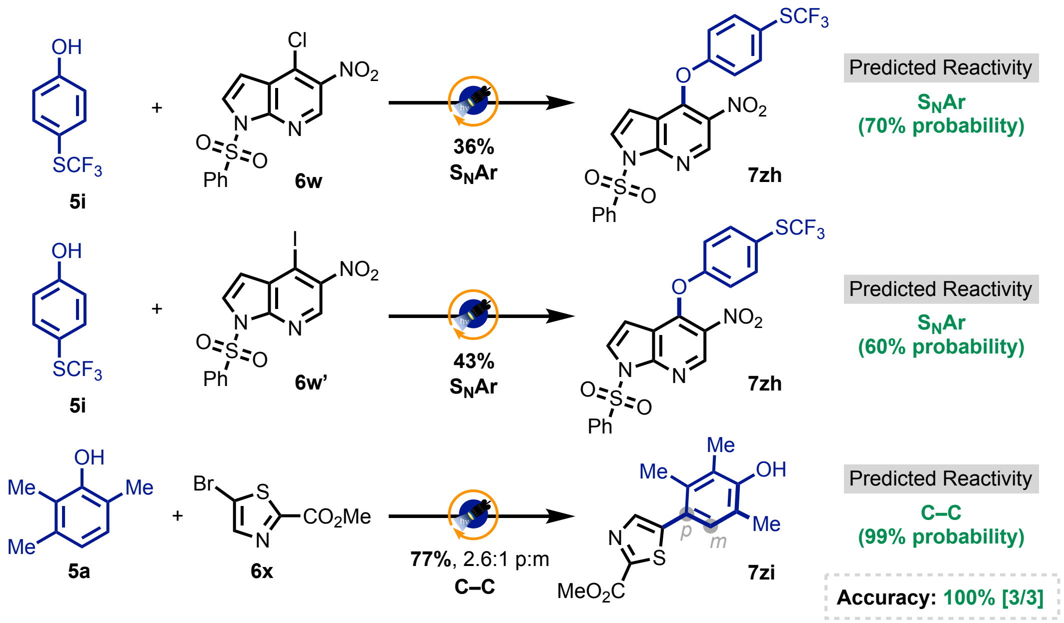

Figure S30. Additional test reactions with their experimental outcomes (under reaction arrow) and logistic regression model prediction in percent (%) probability.

14. Computational Studies

General Remarks

All optimizations of intermediates and transition states were calculated using unrestricted B3LYP-D3[i, ii] and the 6-31G(d,p)[iii] basis set for other atoms using an ultrafine (99,590) grid with the "opt=noeigen" keyword as implemented in Gaussian16. Frequency calculations, using the same method, were used to obtain thermal corrections (at 298.15K; enthalpy and free energy) and to characterize the obtained stationary points as transition states (only one single imaginary frequency) or intermediate (zero imaginary frequencies). Conformational searches were performed manually for all intermediates and transition states, and only the lowest energy species were shown and discussed. Intrinsic reaction coordinate (IRC) calculations were undertaken to ensure that the transition states connect to the correct associated local minima. Single point energy calculations using UB3LYP-D3 /6-311++G(d,p)[iv, v] with implicit solvent (acetonitrile) using CPCM[vi] were also performed on all structures. Electrophilicities were calculated as described by Nagib by calculating the energy of the N, N-1, and N+1 electron systems using B3LYP/6-311++G(d,p) on the optimized structures.[vii] All 3-D structures were generated using CYLview.[viii]

Full Reference of Gaussian 16 Software

Gaussian 16, Revision B.01, M. J. Frisch, G. W. Trucks, H. B. Schlegel, G. E. Scuseria, M. A. Robb, J. R. Cheeseman, G. Scalmani, V. Barone, G. A. Petersson, H. Nakatsuji, X. Li, M. Caricato, A. V. Marenich, J. Bloino, B. G. Janesko, R. Gomperts, B. Mennucci, H. P. Hratchian, J. V. Ortiz, A. F. Izmaylov, J. L. Sonnenberg, D. Williams-Young, F. Ding, F. Lipparini, F. Egidi, J. Goings, B. Peng, A. Petrone, T. Henderson, D. Ranasinghe, V. G. Zakrzewski, J. Gao, N. Rega, G. Zheng, W. Liang, M. Hada, M. Ehara, K. Toyota, R. Fukuda, J. Hasegawa, M. Ishida, T. Nakajima, Y. Honda, O. Kitao, H. Nakai, T. Vreven, K. Throssell, J. A. Montgomery, Jr., J. E. Peralta, F. Ogliaro, M. J. Bearpark, J. J. Heyd, E. N. Brothers, K. N. Kudin, V. N. Staroverov, T. A. Keith, R. Kobayashi, J. Normand, K. Raghavachari, A. P. Rendell, J. C. Burant, S. S. Iyengar, J. Tomasi, M. Cossi, J. M. Millam, M. Klene, C. Adamo, R. Cammi, J. W. Ochterski, R. L. Martin, K. Morokuma, O. Farkas, J. B. Foresman, and D. J. Fox, *Gaussian, Inc.,* Wallingford CT, **2016**.

Phenoxyl Radicals

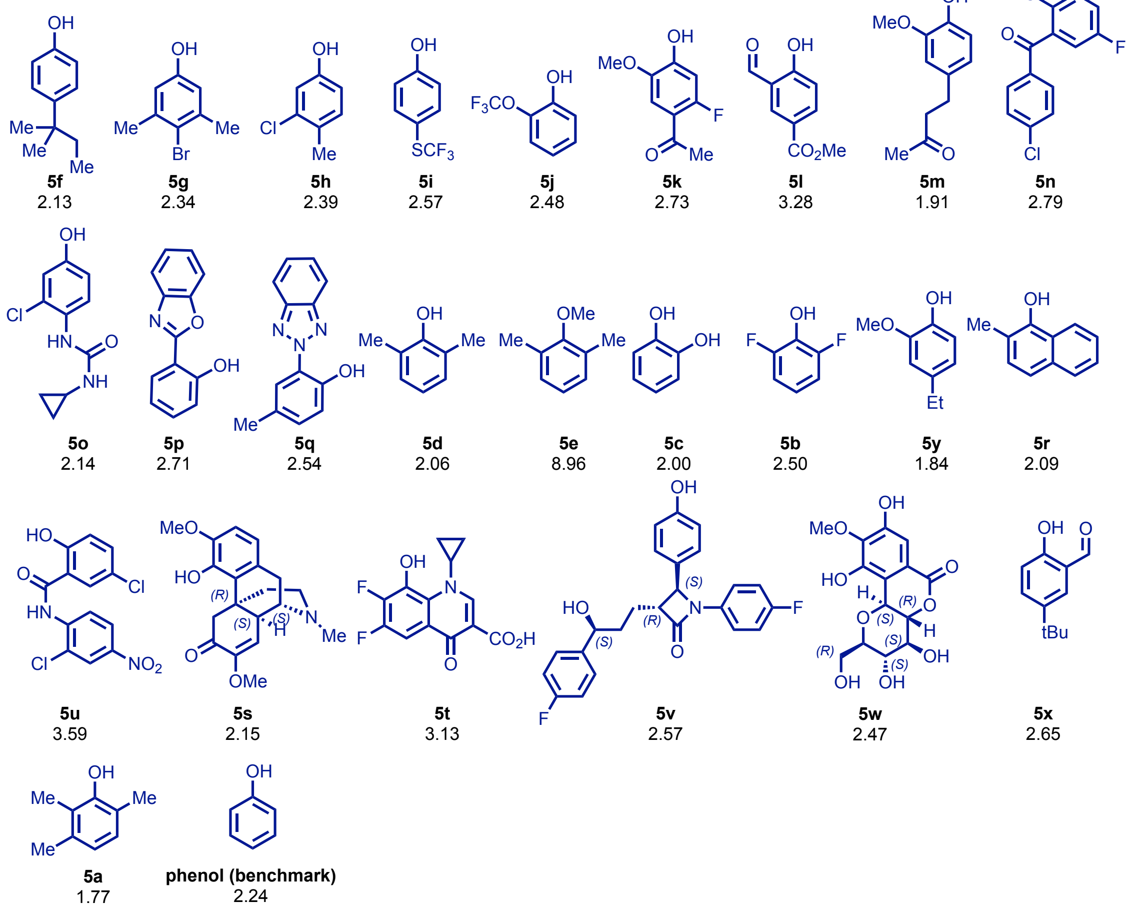

Figure S31. Radical electrophilicities of phenoxyl radicals computed using B3LYP-D3/6-311++G(d,p)//B3LYP/6-311++G(d,p).

Pyridyl Radicals

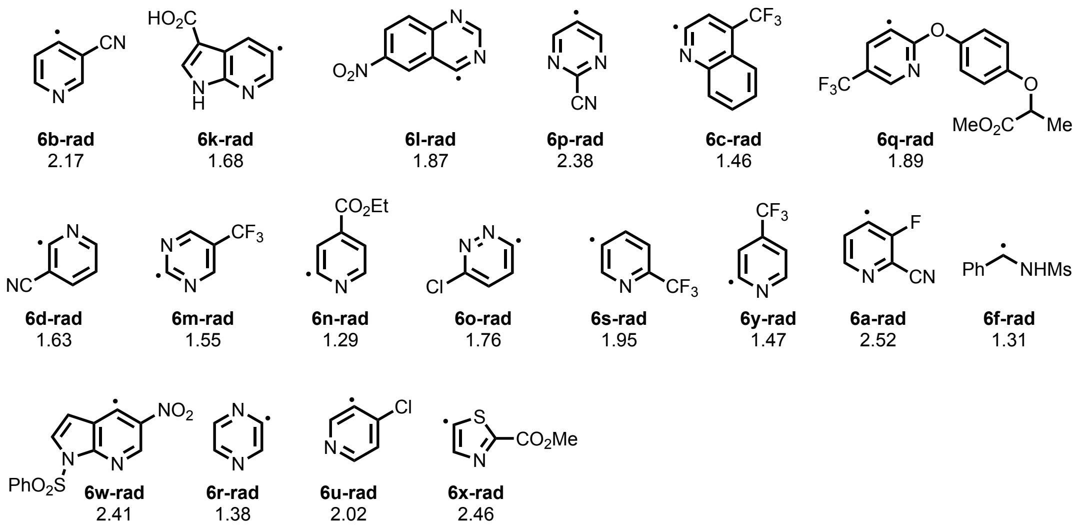

Figure S32. Radical electrophilicities of pyridyl radicals computed using B3LYP-D3/6-311++G(d,p)//B3LYP/6-311++G(d,p).

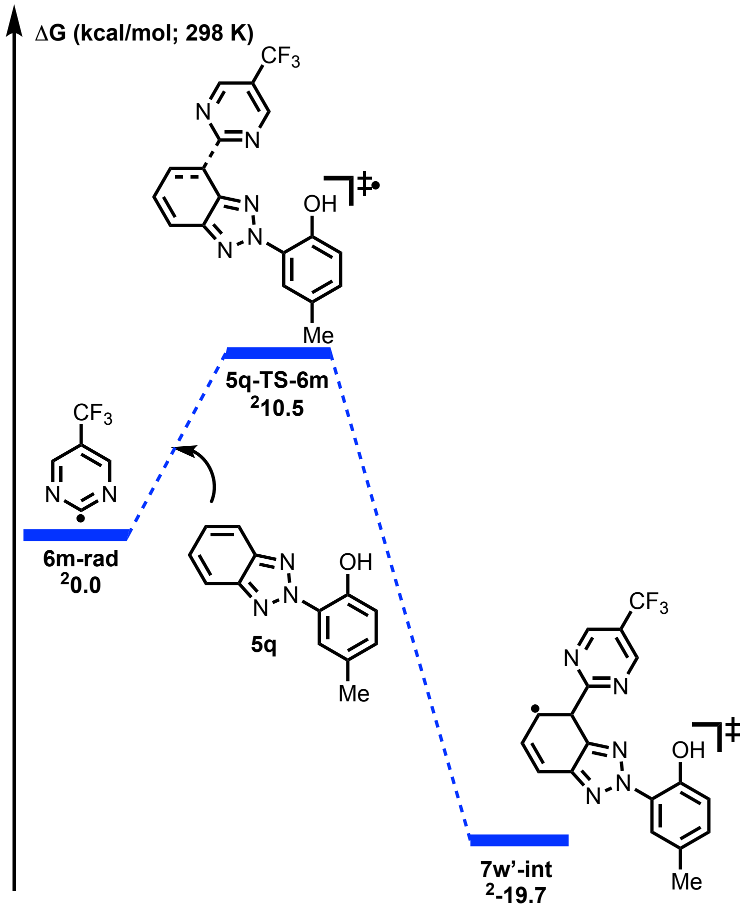

Figure S33. Benzotriazole **5q** and pyridyl **6m** radical addition computed using B3LYP-D3/6-311++G(d,p)-CPCM(ACN)//B3LY-D3P/6-311++G(d,p).

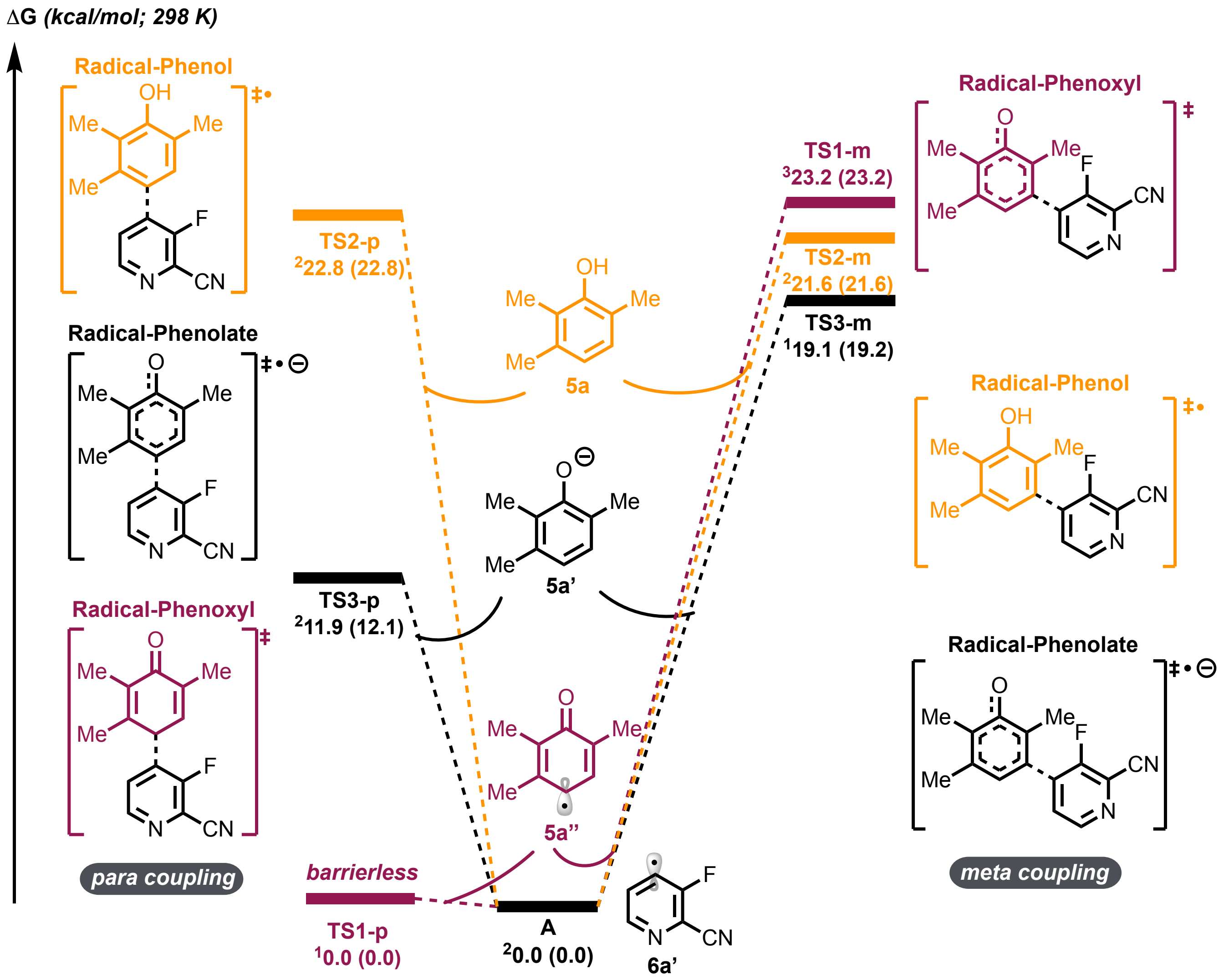

Figure S34. DFT calculations for the radical addition of pyridyl radical **6a'** to phenol **5a**, phenolate **5a'**, and phenoxyl radical **5a''**. Free energies shown in kcal/mol at 298 K and were computed using UB3LYP-D3/6-311++G(d,p)-CPCM(acetonitrile)//UB3LYP-D3/6-31G(d,p) (outside parenthesis) and UB3LYP-D3/6-311++G(d,p)-CPCM(DMSO)//UB3LYP-D3/6-31G(d,p) (parenthesis).

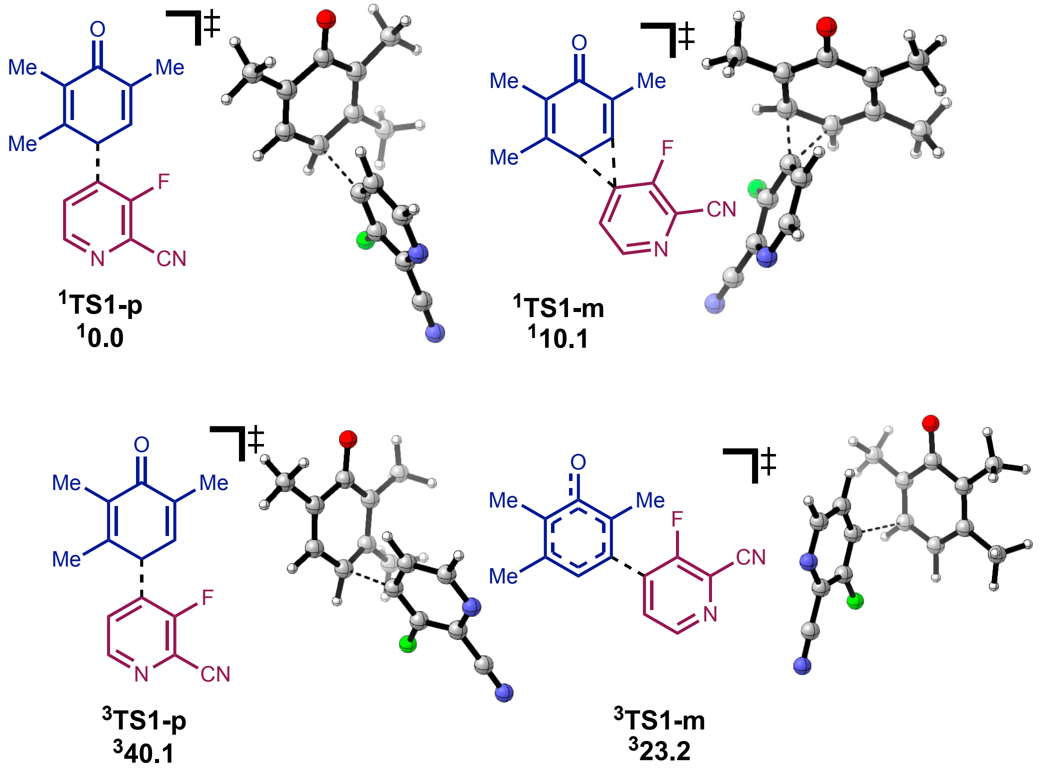

Figure S35. Structural comparison of the singlet and triplet structures of **TS1-p** and **TS1-m.** The singlet form of **TS1-m** involves an isomerization from the para to meta position. Only the triplet form of **TS1-m** involves radical-radical coupling.

Calculated Structures and Energies

5f

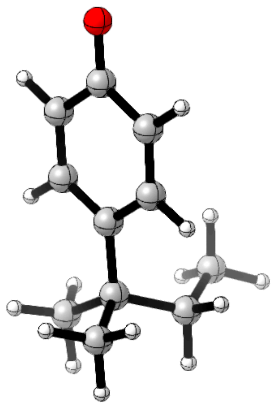

UB3LYP-D3/6-311++G(d,p)
Zero-point correction= 0.232116 (Hartree/Particle)
Thermal correction to Energy= 0.244123
Thermal correction to Enthalpy= 0.245067
Thermal correction to Gibbs Free Energy= 0.193782
Sum of electronic and zero-point Energies= -503.327995
Sum of electronic and thermal Energies= -503.315988
Sum of electronic and thermal Enthalpies= -503.315044
Sum of electronic and thermal Free Energies= -503.366329
UB3LYP/6-311++G(d,p)//UB3LYP-D3/6-311++G(d,p)
HF = -503.5341275

5g

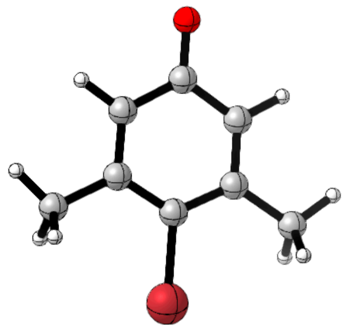

UB3LYP-D3/6-311++G(d,p)
Zero-point correction= 0.136387 (Hartree/Particle)
Thermal correction to Energy= 0.146219
Thermal correction to Enthalpy= 0.147164
Thermal correction to Gibbs Free Energy= 0.099993
Sum of electronic and zero-point Energies= -2958.992152
Sum of electronic and thermal Energies= -2958.982320
Sum of electronic and thermal Enthalpies= -2958.981376
Sum of electronic and thermal Free Energies= -2959.028546
UB3LYP/6-311++G(d,p)//UB3LYP-D3/6-311++G(d,p)
HF = -2959.112351

5h

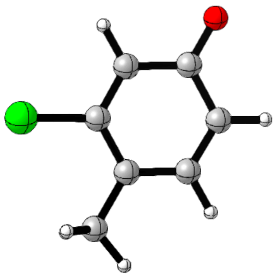

UB3LYP-D3/6-311++G(d,p)
Zero-point correction= 0.109049 (Hartree/Particle)
Thermal correction to Energy= 0.117131
Thermal correction to Enthalpy= 0.118076
Thermal correction to Gibbs Free Energy= 0.075290
Sum of electronic and zero-point Energies= -805.767488
Sum of electronic and thermal Energies= -805.759406
Sum of electronic and thermal Enthalpies= -805.758462
Sum of electronic and thermal Free Energies= -805.801248
UB3LYP/6-311++G(d,p)//UB3LYP-D3/6-311++G(d,p)
HF = -805.8649766

5i

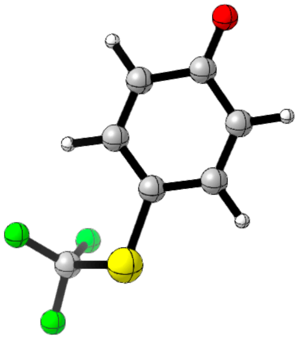

UB3LYP-D3/6-311++G(d,p)
Zero-point correction= 0.096499 (Hartree/Particle)
Thermal correction to Energy= 0.107053
Thermal correction to Enthalpy= 0.107997
Thermal correction to Gibbs Free Energy= 0.057638
Sum of electronic and zero-point Energies= -1042.178982
Sum of electronic and thermal Energies= -1042.168428
Sum of electronic and thermal Enthalpies= -1042.167484
Sum of electronic and thermal Free Energies= -1042.217843
UB3LYP/6-311++G(d,p)//UB3LYP-D3/6-311++G(d,p)
HF = -1042.2628865

5j

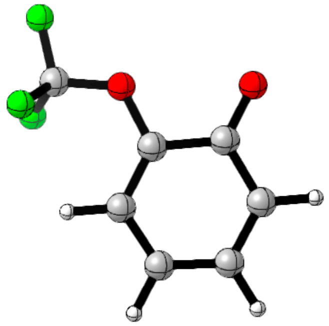

UB3LYP-D3/6-311++G(d,p)
Zero-point correction= 0.100131 (Hartree/Particle)
Thermal correction to Energy= 0.109797
Thermal correction to Enthalpy= 0.110742
Thermal correction to Gibbs Free Energy= 0.063184
Sum of electronic and zero-point Energies= -719.210381
Sum of electronic and thermal Energies= -719.200715
Sum of electronic and thermal Enthalpies= -719.199770
Sum of electronic and thermal Free Energies= -719.247328
UB3LYP/6-311++G(d,p)//UB3LYP-D3/6-311++G(d,p)
HF = -719.2987483

5k

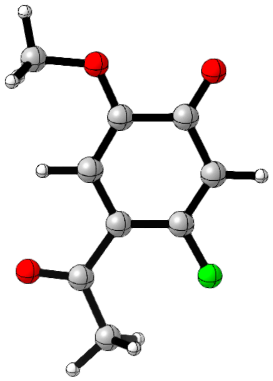

UB3LYP-D3/6-311++G(d,p)
Zero-point correction= 0.152928 (Hartree/Particle)
Thermal correction to Energy= 0.165092
Thermal correction to Enthalpy= 0.166036
Thermal correction to Gibbs Free Energy= 0.113275
Sum of electronic and zero-point Energies= -673.292552
Sum of electronic and thermal Energies= -673.280388
Sum of electronic and thermal Enthalpies= -673.279444
Sum of electronic and thermal Free Energies= -673.332205
UB3LYP/6-311++G(d,p)//UB3LYP-D3/6-311++G(d,p)
HF = -673.4279219

5l

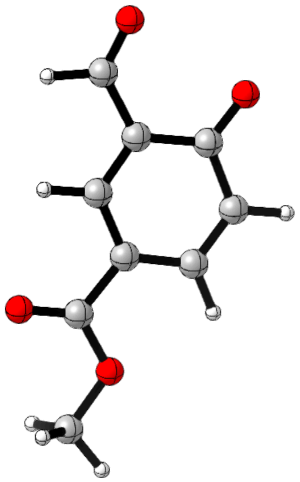

UB3LYP-D3/6-311++G(d,p)
Zero-point correction= 0.142402 (Hartree/Particle)
Thermal correction to Energy= 0.154438
Thermal correction to Enthalpy= 0.155382
Thermal correction to Gibbs Free Energy= 0.102160
Sum of electronic and zero-point Energies= -648.079108
Sum of electronic and thermal Energies= -648.067073
Sum of electronic and thermal Enthalpies= -648.066129
Sum of electronic and thermal Free Energies= -648.119350
UB3LYP/6-311++G(d,p)//UB3LYP-D3/6-311++G(d,p)
HF = -648.2055002

5m

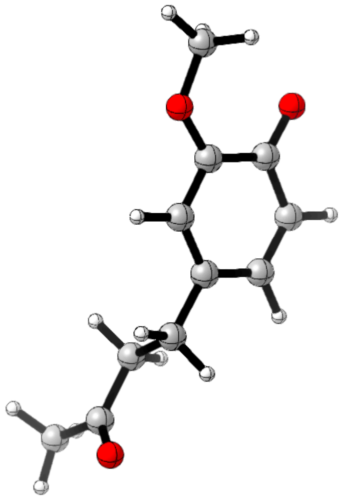

UB3LYP-D3/6-311++G(d,p)
Zero-point correction= 0.217155 (Hartree/Particle)
Thermal correction to Energy= 0.231593
Thermal correction to Enthalpy= 0.232538
Thermal correction to Gibbs Free Energy= 0.172575
Sum of electronic and zero-point Energies= -652.619267
Sum of electronic and thermal Energies= -652.604828
Sum of electronic and thermal Enthalpies= -652.603884
Sum of electronic and thermal Free Energies= -652.663847
UB3LYP/6-311++G(d,p)//UB3LYP-D3/6-311++G(d,p)
HF = -652.8135673

5n

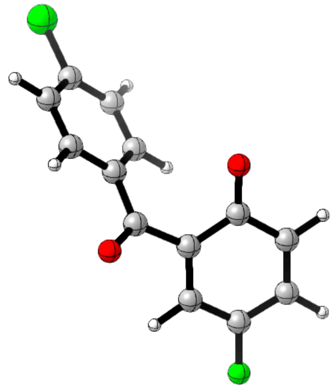

UB3LYP-D3/6-311++G(d,p)
Zero-point correction= 0.163727 (Hartree/Particle)
Thermal correction to Energy= 0.177632
Thermal correction to Enthalpy= 0.178576
Thermal correction to Gibbs Free Energy= 0.120375
Sum of electronic and zero-point Energies= -1210.131568
Sum of electronic and thermal Energies= -1210.117664
Sum of electronic and thermal Enthalpies= -1210.116719
Sum of electronic and thermal Free Energies= -1210.174921
UB3LYP/6-311++G(d,p)//UB3LYP-D3/6-311++G(d,p)
HF = -1210.272052

5o

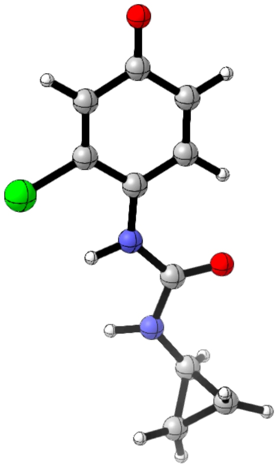

UB3LYP-D3/6-311++G(d,p)
Zero-point correction= 0.187782 (Hartree/Particle)
Thermal correction to Energy= 0.201848
Thermal correction to Enthalpy= 0.202793
Thermal correction to Gibbs Free Energy= 0.144091
Sum of electronic and zero-point Energies= -1107.235378
Sum of electronic and thermal Energies= -1107.221311
Sum of electronic and thermal Enthalpies= -1107.220367
Sum of electronic and thermal Free Energies= -1107.279068
UB3LYP/6-311++G(d,p)//UB3LYP-D3/6-311++G(d,p)
HF = -1107.4015011

5p

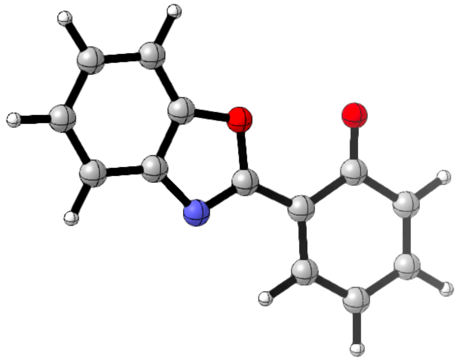

UB3LYP-D3/6-311++G(d,p)
Zero-point correction= 0.176436 (Hartree/Particle)
Thermal correction to Energy= 0.188018
Thermal correction to Enthalpy= 0.188962
Thermal correction to Gibbs Free Energy= 0.136452
Sum of electronic and zero-point Energies= -705.388113
Sum of electronic and thermal Energies= -705.376531
Sum of electronic and thermal Enthalpies= -705.375587
Sum of electronic and thermal Free Energies= -705.428098
UB3LYP/6-311++G(d,p)//UB3LYP-D3/6-311++G(d,p)
HF = -705.5469854

5q

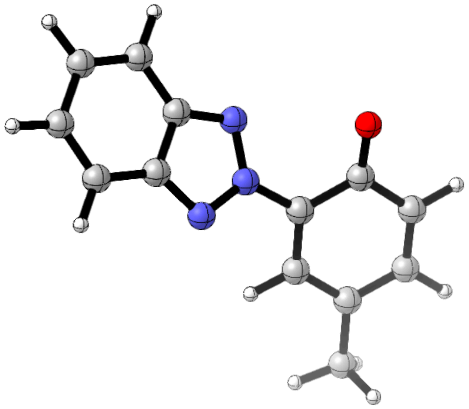

UB3LYP-D3/6-311++G(d,p)
Zero-point correction= 0.204211 (Hartree/Particle)
Thermal correction to Energy= 0.217563
Thermal correction to Enthalpy= 0.218507
Thermal correction to Gibbs Free Energy= 0.162066
Sum of electronic and zero-point Energies= -740.821307
Sum of electronic and thermal Energies= -740.807956
Sum of electronic and thermal Enthalpies= -740.807012
Sum of electronic and thermal Free Energies= -740.863452
UB3LYP/6-311++G(d,p)//UB3LYP-D3/6-311++G(d,p)
HF = -741.0044735

5d

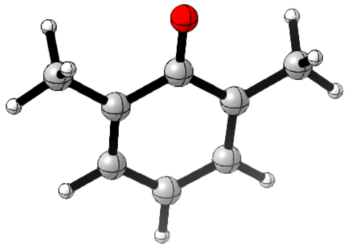

UB3LYP-D3/6-311++G(d,p)
Zero-point correction= 0.146094 (Hartree/Particle)
Thermal correction to Energy= 0.154828
Thermal correction to Enthalpy= 0.155772
Thermal correction to Gibbs Free Energy= 0.111971
Sum of electronic and zero-point Energies= -385.442015
Sum of electronic and thermal Energies= -385.433281
Sum of electronic and thermal Enthalpies= -385.432337
Sum of electronic and thermal Free Energies= -385.476138
UB3LYP/6-311++G(d,p)//UB3LYP-D3/6-311++G(d,p)
HF = -385.5758899

5e

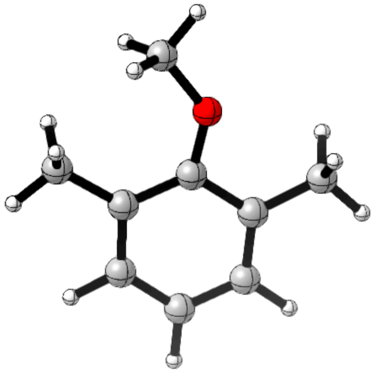

UB3LYP-D3/6-311++G(d,p)
Zero-point correction= 0.187217 (Hartree/Particle)
Thermal correction to Energy= 0.197697
Thermal correction to Enthalpy= 0.198641
Thermal correction to Gibbs Free Energy= 0.150455
Sum of electronic and zero-point Energies= -425.061272
Sum of electronic and thermal Energies= -425.050793
Sum of electronic and thermal Enthalpies= -425.049848
Sum of electronic and thermal Free Energies= -425.098035
UB3LYP/6-311++G(d,p)//UB3LYP-D3/6-311++G(d,p)
HF = -425.2318649

5c

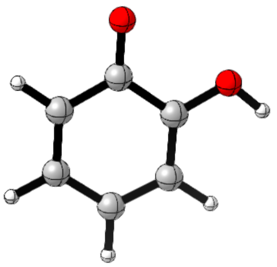

UB3LYP-D3/6-311++G(d,p)
Zero-point correction= 0.095525 (Hartree/Particle)
Thermal correction to Energy= 0.101955
Thermal correction to Enthalpy= 0.102899
Thermal correction to Gibbs Free Energy= 0.064489
Sum of electronic and zero-point Energies= -382.073844
Sum of electronic and thermal Energies= -382.067414
Sum of electronic and thermal Enthalpies= -382.066470
Sum of electronic and thermal Free Energies= -382.104881
UB3LYP/6-311++G(d,p)//UB3LYP-D3/6-311++G(d,p)
HF = -382.162062

5b

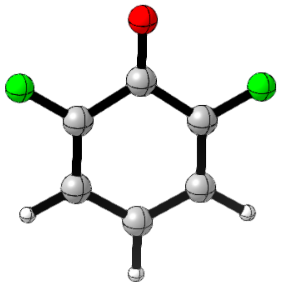

UB3LYP-D3/6-311++G(d,p)
Zero-point correction= 0.075101 (Hartree/Particle)
Thermal correction to Energy= 0.082028
Thermal correction to Enthalpy= 0.082972
Thermal correction to Gibbs Free Energy= 0.042937
Sum of electronic and zero-point Energies= -505.375590
Sum of electronic and thermal Energies= -505.368663
Sum of electronic and thermal Enthalpies= -505.367719
Sum of electronic and thermal Free Energies= -505.407754
UB3LYP/6-311++G(d,p)//UB3LYP-D3/6-311++G(d,p)
HF = -505.4436282

5y

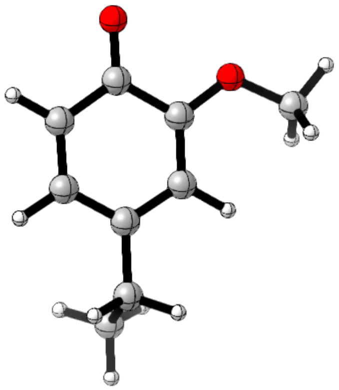

UB3LYP-D3/6-311++G(d,p)
Zero-point correction= 0.180110 (Hartree/Particle)
Thermal correction to Energy= 0.190930
Thermal correction to Enthalpy= 0.191874
Thermal correction to Gibbs Free Energy= 0.142358
Sum of electronic and zero-point Energies= -499.962141
Sum of electronic and thermal Energies= -499.951321
Sum of electronic and thermal Enthalpies= -499.950377
Sum of electronic and thermal Free Energies= -499.999893
UB3LYP/6-311++G(d,p)//UB3LYP-D3/6-311++G(d,p)
HF = -500.1258861

5r

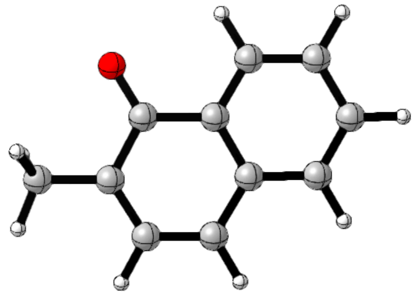

UB3LYP-D3/6-311++G(d,p)
Zero-point correction= 0.165729 (Hartree/Particle)
Thermal correction to Energy= 0.175285
Thermal correction to Enthalpy= 0.176230
Thermal correction to Gibbs Free Energy= 0.130167
Sum of electronic and zero-point Energies= -499.782979
Sum of electronic and thermal Energies= -499.773423
Sum of electronic and thermal Enthalpies= -499.772479
Sum of electronic and thermal Free Energies= -499.818541
UB3LYP/6-311++G(d,p)//UB3LYP-D3/6-311++G(d,p)
HF = -499.9330872

5u

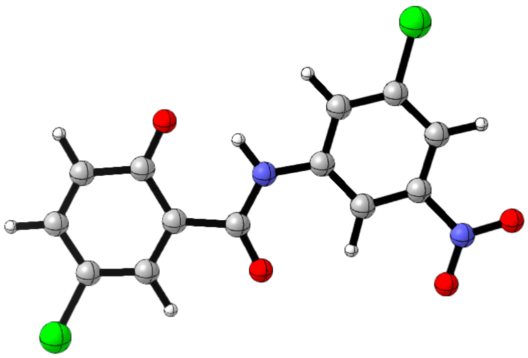

UB3LYP-D3/6-311++G(d,p)
Zero-point correction= 0.182316 (Hartree/Particle)
Thermal correction to Energy= 0.200168
Thermal correction to Enthalpy= 0.201112
Thermal correction to Gibbs Free Energy= 0.132274
Sum of electronic and zero-point Energies= -1830.434950
Sum of electronic and thermal Energies= -1830.417098
Sum of electronic and thermal Enthalpies= -1830.416154
Sum of electronic and thermal Free Energies= -1830.484992
UB3LYP/6-311++G(d,p)//UB3LYP-D3/6-311++G(d,p)
HF = -1830.5882142

5s

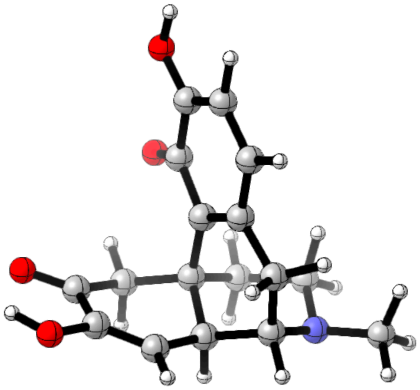

UB3LYP-D3/6-311++G(d,p)
Zero-point correction= 0.325294 (Hartree/Particle)
Thermal correction to Energy= 0.343199
Thermal correction to Enthalpy= 0.344143
Thermal correction to Gibbs Free Energy= 0.280338
Sum of electronic and zero-point Energies= -1014.245020
Sum of electronic and thermal Energies= -1014.227116
Sum of electronic and thermal Enthalpies= -1014.226172
Sum of electronic and thermal Free Energies= -1014.289976
UB3LYP/6-311++G(d,p)//UB3LYP-D3/6-311++G(d,p)
HF = -1014.5190587

5t

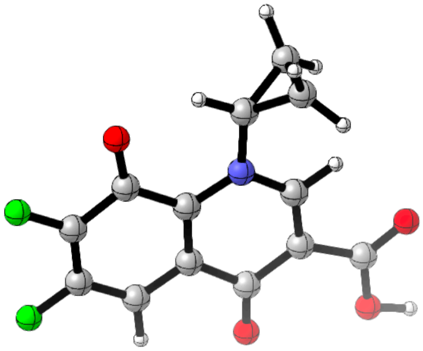

UB3LYP-D3/6-311++G(d,p)
Zero-point correction= 0.191543 (Hartree/Particle)
Thermal correction to Energy= 0.208295
Thermal correction to Enthalpy= 0.209239
Thermal correction to Gibbs Free Energy= 0.143859
Sum of electronic and zero-point Energies= -1055.597424
Sum of electronic and thermal Energies= -1055.580672
Sum of electronic and thermal Enthalpies= -1055.579728
Sum of electronic and thermal Free Energies= -1055.645108
UB3LYP/6-311++G(d,p)//UB3LYP-D3/6-311++G(d,p)
HF = -1055.7615482

5v

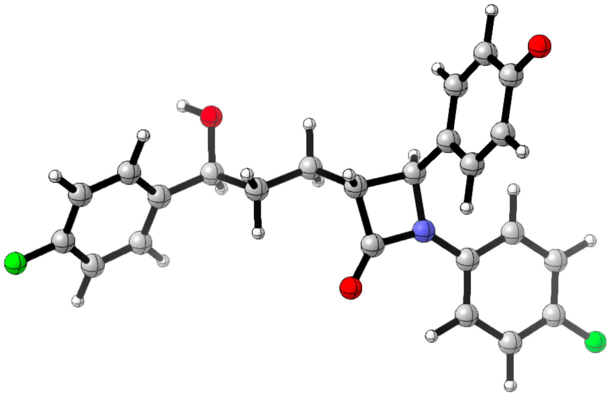

UB3LYP-D3/6-311++G(d,p)
Zero-point correction= 0.387050 (Hartree/Particle)
Thermal correction to Energy= 0.413080
Thermal correction to Enthalpy= 0.414024
Thermal correction to Gibbs Free Energy= 0.325065
Sum of electronic and zero-point Energies= -1406.713870
Sum of electronic and thermal Energies= -1406.687840
Sum of electronic and thermal Enthalpies= -1406.686896
Sum of electronic and thermal Free Energies= -1406.775855
UB3LYP/6-311++G(d,p)//UB3LYP-D3/6-311++G(d,p)
HF = -1407.0476959

5w

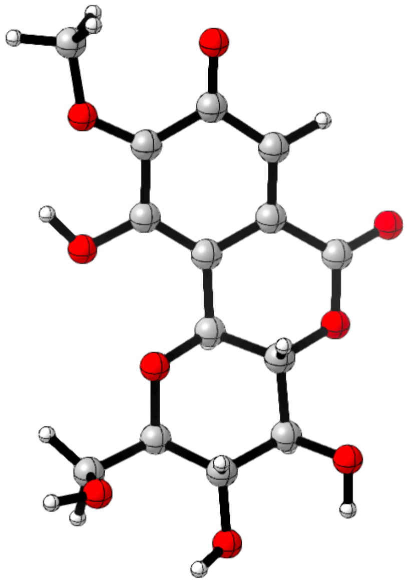

UB3LYP-D3/6-311++G(d,p)
Zero-point correction= 0.289538 (Hartree/Particle)
Thermal correction to Energy= 0.310707
Thermal correction to Enthalpy= 0.311651
Thermal correction to Gibbs Free Energy= 0.238789
Sum of electronic and zero-point Energies= -1219.577057
Sum of electronic and thermal Energies= -1219.555888
Sum of electronic and thermal Enthalpies= -1219.554944
Sum of electronic and thermal Free Energies= -1219.627806
UB3LYP/6-311++G(d,p)//UB3LYP-D3/6-311++G(d,p)
HF = -1219.8220405

5x

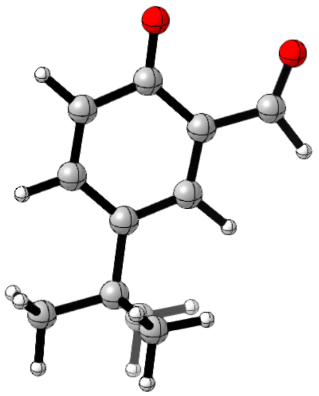

UB3LYP-D3/6-311++G(d,p)
Zero-point correction= 0.212240 (Hartree/Particle)
Thermal correction to Energy= 0.225154
Thermal correction to Enthalpy= 0.226098
Thermal correction to Gibbs Free Energy= 0.171854
Sum of electronic and zero-point Energies= -577.371874
Sum of electronic and thermal Energies= -577.358960
Sum of electronic and thermal Enthalpies= -577.358016
Sum of electronic and thermal Free Energies= -577.412260
UB3LYP/6-311++G(d,p)//UB3LYP-D3/6-311++G(d,p)
HF = -577.5596145

6b-rad

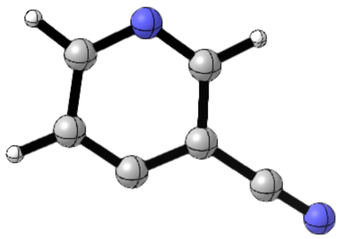

UB3LYP-D3/6-311++G(d,p)
Zero-point correction= 0.073742 (Hartree/Particle)
Thermal correction to Energy= 0.079735
Thermal correction to Enthalpy= 0.080679
Thermal correction to Gibbs Free Energy= 0.042883
Sum of electronic and zero-point Energies= -339.855966
Sum of electronic and thermal Energies= -339.849973
Sum of electronic and thermal Enthalpies= -339.849029
Sum of electronic and thermal Free Energies= -339.886824
UB3LYP/6-311++G(d,p)//UB3LYP-D3/6-311++G(d,p)
HF = -339.9244998

6k-rad

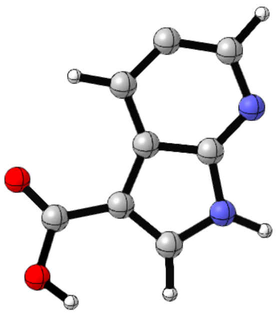

UB3LYP-D3/6-311++G(d,p)
Zero-point correction= 0.119327 (Hartree/Particle)
Thermal correction to Energy= 0.128440
Thermal correction to Enthalpy= 0.129384
Thermal correction to Gibbs Free Energy= 0.083795
Sum of electronic and zero-point Energies= -567.793414
Sum of electronic and thermal Energies= -567.784302
Sum of electronic and thermal Enthalpies= -567.783357
Sum of electronic and thermal Free Energies= -567.828947
UB3LYP/6-311++G(d,p)//UB3LYP-D3/6-311++G(d,p)
HF = -567.9002791

6l-rad

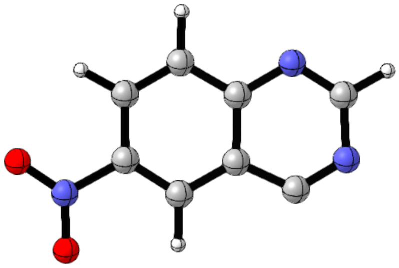

UB3LYP-D3/6-311++G(d,p)
Zero-point correction= 0.112635 (Hartree/Particle)
Thermal correction to Energy= 0.121841
Thermal correction to Enthalpy= 0.122785
Thermal correction to Gibbs Free Energy= 0.076339
Sum of electronic and zero-point Energies= -621.857462
Sum of electronic and thermal Energies= -621.848255
Sum of electronic and thermal Enthalpies= -621.847311
Sum of electronic and thermal Free Energies= -621.893758
UB3LYP/6-311++G(d,p)//UB3LYP-D3/6-311++G(d,p)
HF = -621.9575769

6p-rad

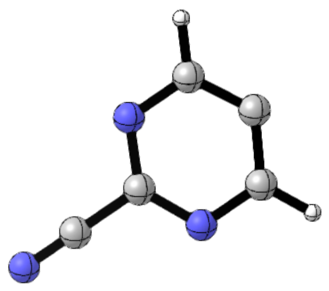

UB3LYP-D3/6-311++G(d,p)
Zero-point correction= 0.061948 (Hartree/Particle)
Thermal correction to Energy= 0.067816
Thermal correction to Enthalpy= 0.068760
Thermal correction to Gibbs Free Energy= 0.031202
Sum of electronic and zero-point Energies= -355.901180
Sum of electronic and thermal Energies= -355.895312
Sum of electronic and thermal Enthalpies= -355.894368
Sum of electronic and thermal Free Energies= -355.931927
UB3LYP/6-311++G(d,p)//UB3LYP-D3/6-311++G(d,p)
HF = -355.9586219

6c-rad

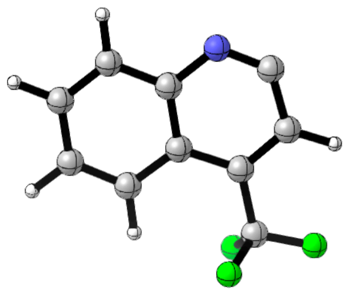

UB3LYP-D3/6-311++G(d,p)
Zero-point correction= 0.126927 (Hartree/Particle)
Thermal correction to Energy= 0.137151
Thermal correction to Enthalpy= 0.138095
Thermal correction to Gibbs Free Energy= 0.089895
Sum of electronic and zero-point Energies= -738.385117
Sum of electronic and thermal Energies= -738.374893
Sum of electronic and thermal Enthalpies= -738.373949
Sum of electronic and thermal Free Energies= -738.422148
UB3LYP/6-311++G(d,p)//UB3LYP-D3/6-311++G(d,p)
HF = -738.4964384

6q-rad

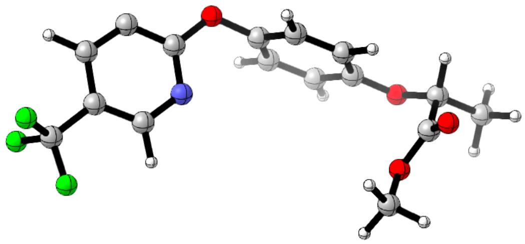

UB3LYP-D3/6-311++G(d,p)
Zero-point correction= 0.267069 (Hartree/Particle)
Thermal correction to Energy= 0.289074
Thermal correction to Enthalpy= 0.290018
Thermal correction to Gibbs Free Energy= 0.208924
Sum of electronic and zero-point Energies= -1272.749163
Sum of electronic and thermal Energies= -1272.727159
Sum of electronic and thermal Enthalpies= -1272.726215
Sum of electronic and thermal Free Energies= -1272.807309
UB3LYP/6-311++G(d,p)//UB3LYP-D3/6-311++G(d,p)
HF = -1272.9814768

6d-rad

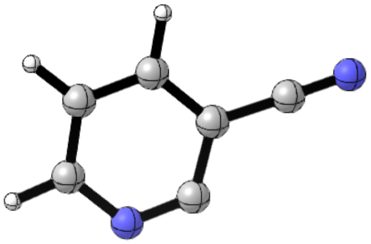

UB3LYP-D3/6-311++G(d,p)
Thermal correction to Energy= 0.080094
Thermal correction to Enthalpy= 0.081038
Thermal correction to Gibbs Free Energy= 0.043250
Sum of electronic and zero-point Energies= -339.864798
Sum of electronic and thermal Energies= -339.858802
Sum of electronic and thermal Enthalpies= -339.857858
Sum of electronic and thermal Free Energies= -339.895646
UB3LYP/6-311++G(d,p)//UB3LYP-D3/6-311++G(d,p)
HF = -339.93359

6m-rad

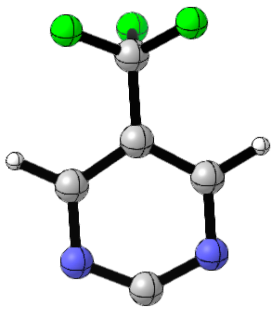

UB3LYP-D3/6-311++G(d,p)
Zero-point correction= 0.067987 (Hartree/Particle)
Thermal correction to Energy= 0.075713
Thermal correction to Enthalpy= 0.076657
Thermal correction to Gibbs Free Energy= 0.032884
Sum of electronic and zero-point Energies= -600.794383
Sum of electronic and thermal Energies= -600.786657
Sum of electronic and thermal Enthalpies= -600.785713
Sum of electronic and thermal Free Energies= -600.829486
UB3LYP/6-311++G(d,p)//UB3LYP-D3/6-311++G(d,p)
HF = -600.8545499

6n-rad

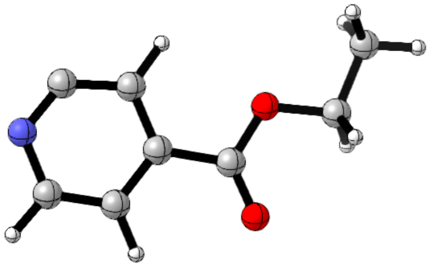

UB3LYP-D3/6-311++G(d,p)
Zero-point correction= 0.146434 (Hartree/Particle)
Thermal correction to Energy= 0.156439
Thermal correction to Enthalpy= 0.157383
Thermal correction to Gibbs Free Energy= 0.109096
Sum of electronic and zero-point Energies= -514.811767
Sum of electronic and thermal Energies= -514.801762
Sum of electronic and thermal Enthalpies= -514.800818
Sum of electronic and thermal Free Energies= -514.849105
UB3LYP/6-311++G(d,p)//UB3LYP-D3/6-311++G(d,p)
HF = -514.9449559

6o-rad

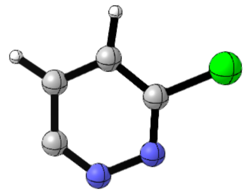

UB3LYP-D3/6-311++G(d,p)
Zero-point correction= 0.052891 (Hartree/Particle)
Thermal correction to Energy= 0.058271
Thermal correction to Enthalpy= 0.059215
Thermal correction to Gibbs Free Energy= 0.022557
Sum of electronic and zero-point Energies= -723.247097
Sum of electronic and thermal Energies= -723.241717
Sum of electronic and thermal Enthalpies= -723.240773
Sum of electronic and thermal Free Energies= -723.277431
UB3LYP/6-311++G(d,p)//UB3LYP-D3/6-311++G(d,p)
HF = -723.2951551

6s-rad

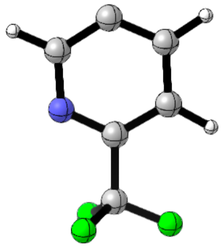

UB3LYP-D3/6-311++G(d,p)
Zero-point correction= 0.079813 (Hartree/Particle)
Thermal correction to Energy= 0.087540
Thermal correction to Enthalpy= 0.088484
Thermal correction to Gibbs Free Energy= 0.045504
Sum of electronic and zero-point Energies= -584.736214
Sum of electronic and thermal Energies= -584.728487
Sum of electronic and thermal Enthalpies= -584.727543
Sum of electronic and thermal Free Energies= -584.770523
UB3LYP/6-311++G(d,p)//UB3LYP-D3/6-311++G(d,p)
HF = -584.8080791

6y-rad

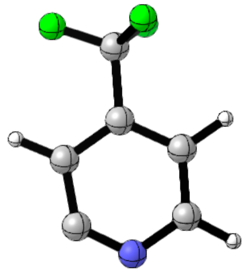

UB3LYP-D3/6-311++G(d,p)
Zero-point correction= 0.079951 (Hartree/Particle)
Thermal correction to Energy= 0.086835
Thermal correction to Enthalpy= 0.087779
Thermal correction to Gibbs Free Energy= 0.047412
Sum of electronic and zero-point Energies= -584.745437
Sum of electronic and thermal Energies= -584.738554
Sum of electronic and thermal Enthalpies= -584.737610
Sum of electronic and thermal Free Energies= -584.777977
UB3LYP/6-311++G(d,p)//UB3LYP-D3/6-311++G(d,p)
HF = -584.8169878

6w-rad

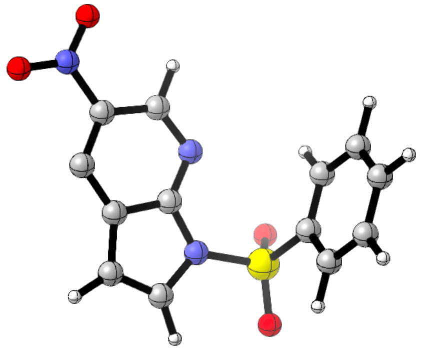

UB3LYP-D3/6-311++G(d,p)
Zero-point correction= 0.197097 (Hartree/Particle)
Thermal correction to Energy= 0.213818
Thermal correction to Enthalpy= 0.214762
Thermal correction to Gibbs Free Energy= 0.149671
Sum of electronic and zero-point Energies= -1363.397933
Sum of electronic and thermal Energies= -1363.381212
Sum of electronic and thermal Enthalpies= -1363.380268
Sum of electronic and thermal Free Energies= -1363.445360
UB3LYP/6-311++G(d,p)//UB3LYP-D3/6-311++G(d,p)
HF = -1363.5633988

6r-rad

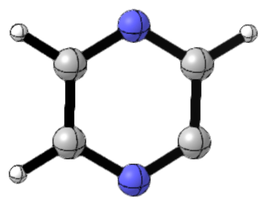

UB3LYP-D3/6-311++G(d,p)
Zero-point correction= 0.063528 (Hartree/Particle)
Thermal correction to Energy= 0.067701
Thermal correction to Enthalpy= 0.068645
Thermal correction to Gibbs Free Energy= 0.035606
Sum of electronic and zero-point Energies= -263.646842
Sum of electronic and thermal Energies= -263.642668
Sum of electronic and thermal Enthalpies= -263.641724
Sum of electronic and thermal Free Energies= -263.674763
UB3LYP/6-311++G(d,p)//UB3LYP-D3/6-311++G(d,p)
HF = -263.707285

6u-rad

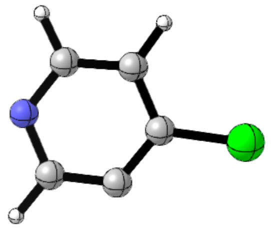

UB3LYP-D3/6-311++G(d,p)
Zero-point correction= 0.065932 (Hartree/Particle)
Thermal correction to Energy= 0.071307
Thermal correction to Enthalpy= 0.072251
Thermal correction to Gibbs Free Energy= 0.035545
Sum of electronic and zero-point Energies= -707.221043
Sum of electronic and thermal Energies= -707.215668
Sum of electronic and thermal Enthalpies= -707.214724
Sum of electronic and thermal Free Energies= -707.251430
UB3LYP/6-311++G(d,p)//UB3LYP-D3/6-311++G(d,p)
HF = -707.2817447

6x-rad

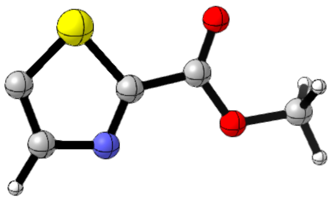

UB3LYP-D3/6-311++G(d,p)
Zero-point correction= 0.084949 (Hartree/Particle)
Thermal correction to Energy= 0.093345
Thermal correction to Enthalpy= 0.094289
Thermal correction to Gibbs Free Energy= 0.050067
Sum of electronic and zero-point Energies= -796.287844
Sum of electronic and thermal Energies= -796.279449
Sum of electronic and thermal Enthalpies= -796.278505
Sum of electronic and thermal Free Energies= -796.322726
UB3LYP/6-311++G(d,p)//UB3LYP-D3/6-311++G(d,p)
HF = -796.3651319

6a-rad

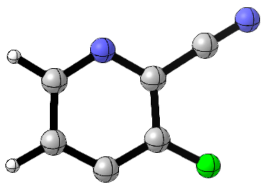

UB3LYP-D3/6-31G(d,p)
Zero-point correction= 0.066035 (Hartree/Particle)
Thermal correction to Energy= 0.072812
Thermal correction to Enthalpy= 0.073756
Thermal correction to Gibbs Free Energy= 0.033892
Sum of electronic and zero-point Energies= -439.003578
Sum of electronic and thermal Energies= -438.996801
Sum of electronic and thermal Enthalpies= -438.995857
Sum of electronic and thermal Free Energies= -439.035721
UB3LYP-D3/6-311++G(d,p)-CPCM(acetonitrile)//UB3LYP-D3/6-31G(d,p)
HF = -439.1915592
UB3LYP-D3/6-311++G(d,p)-CPCM(DMSO)//UB3LYP-D3/6-31G(d,p)
HF = -439.1916398

5a (B3LYP)

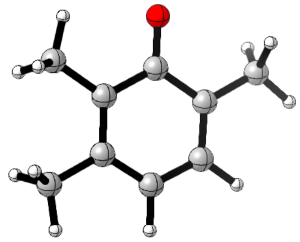

B3LYP/6-311++G(d,p)
Zero-point correction= 0.173695 (Hartree/Particle)
Thermal correction to Energy= 0.184008
Thermal correction to Enthalpy= 0.184953
Thermal correction to Gibbs Free Energy= 0.137690
Sum of electronic and zero-point Energies= -424.743991
Sum of electronic and thermal Energies= -424.733678
Sum of electronic and thermal Enthalpies= -424.732734
Sum of electronic and thermal Free Energies= -424.779996
B3LYP/6-311++G(d,p)//B3LYP/6-311++G(d,p)
HF = -424.9176866

Phenol (benchmark)

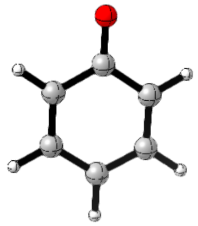

B3LYP/6-311++G(d,p)
Zero-point correction= 0.090959 (Hartree/Particle)
Thermal correction to Energy= 0.096281
Thermal correction to Enthalpy= 0.097225
Thermal correction to Gibbs Free Energy= 0.061383
Sum of electronic and zero-point Energies= -306.828524
Sum of electronic and thermal Energies= -306.823203
Sum of electronic and thermal Enthalpies= -306.822258
Sum of electronic and thermal Free Energies= -306.858100
B3LYP/6-311++G(d,p)//B3LYP/6-311++G(d,p)
HF = -306.9134496

6f-rad

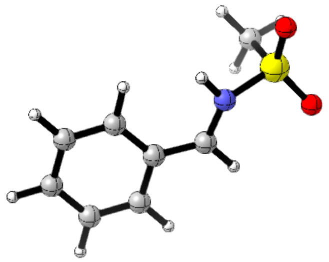

B3LYP/6-311++G(d,p)
Zero-point correction= 0.169504 (Hartree/Particle)
Thermal correction to Energy= 0.181286
Thermal correction to Enthalpy= 0.182230
Thermal correction to Gibbs Free Energy= 0.129878
Sum of electronic and zero-point Energies= -914.182451
Sum of electronic and thermal Energies= -914.170670
Sum of electronic and thermal Enthalpies= -914.169725
Sum of electronic and thermal Free Energies= -914.222078
B3LYP/6-311++G(d,p)//B3LYP/6-311++G(d,p)
HF = -914.3335747

5q (B3LYP)

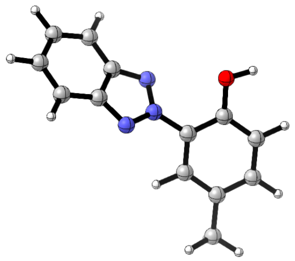

B3LYP-D3/6-311++G(d,p)
Zero-point correction= 0.217263 (Hartree/Particle)
Thermal correction to Energy= 0.230958
Thermal correction to Enthalpy= 0.231902
Thermal correction to Gibbs Free Energy= 0.175304
Sum of electronic and zero-point Energies= -741.450111
Sum of electronic and thermal Energies= -741.436417
Sum of electronic and thermal Enthalpies= -741.435473
Sum of electronic and thermal Free Energies= -741.492071
B3LYP-D3/6-311++G(d,p)-CPCM(ACN)//B3LY-D3P/6-311++G(d,p)
HF = -741.6803529

6m-rad (B3LYP)

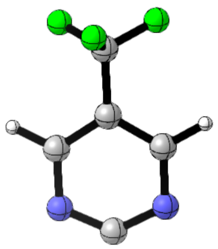

B3LYP-D3/6-311++G(d,p)
Zero-point correction= 0.067988 (Hartree/Particle)
Thermal correction to Energy= 0.075712
Thermal correction to Enthalpy= 0.076656
Thermal correction to Gibbs Free Energy= 0.032944
Sum of electronic and zero-point Energies= -600.794382
Sum of electronic and thermal Energies= -600.786658
Sum of electronic and thermal Enthalpies= -600.785714
Sum of electronic and thermal Free Energies= -600.829425
B3LYP-D3/6-311++G(d,p)-CPCM(ACN)//B3LY-D3P/6-311++G(d,p)
HF = -600.8691065

5q-TS-6m

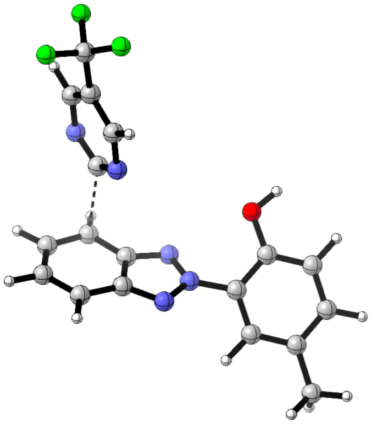

B3LYP-D3/6-311++G(d,p)
Imaginary frequency = -233.30 cm^{-1}
Zero-point correction= 0.285186 (Hartree/Particle)
Thermal correction to Energy= 0.308001
Thermal correction to Enthalpy= 0.308946
Thermal correction to Gibbs Free Energy= 0.225414
Sum of electronic and zero-point Energies= -1342.246569
Sum of electronic and thermal Energies= -1342.223754
Sum of electronic and thermal Enthalpies= -1342.222809
Sum of electronic and thermal Free Energies= -1342.306341
B3LYP-D3/6-311++G(d,p)-CPCM(ACN)//B3LY-D3P/6-311++G(d,p)
HF = -1342.5499368

7w'-int

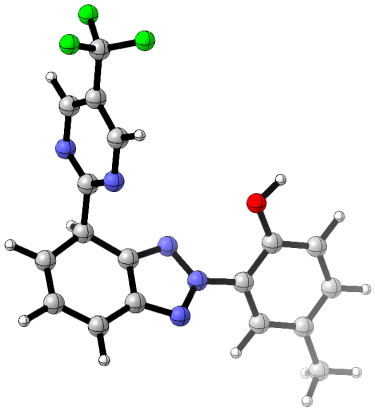

B3LYP-D3/6-311++G(d,p)
Zero-point correction= 0.287604 (Hartree/Particle)
Thermal correction to Energy= 0.310154
Thermal correction to Enthalpy= 0.311098
Thermal correction to Gibbs Free Energy= 0.229338
Sum of electronic and zero-point Energies= -1342.296837
Sum of electronic and thermal Energies= -1342.274287
Sum of electronic and thermal Enthalpies= -1342.273343
Sum of electronic and thermal Free Energies= -1342.355104
B3LYP-D3/6-311++G(d,p)-CPCM(ACN)//B3LY-D3P/6-311++G(d,p)
HF = -1342.6018659

5a'' (phenoxyl)

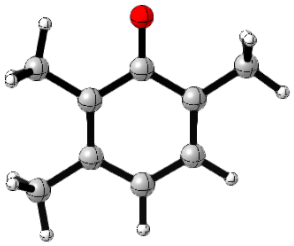

UB3LYP-D3/6-31G(d,p)
Zero-point correction= 0.174950 (Hartree/Particle)
Thermal correction to Energy= 0.185188
Thermal correction to Enthalpy= 0.186132
Thermal correction to Gibbs Free Energy= 0.139081
Sum of electronic and zero-point Energies= -424.644326
Sum of electronic and thermal Energies= -424.634089
Sum of electronic and thermal Enthalpies= -424.633144
Sum of electronic and thermal Free Energies= -424.680195
UB3LYP-D3/6-311++G(d,p)-CPCM(acetonitrile)//UB3LYP-D3/6-31G(d,p)
HF = -424.9087476
UB3LYP-D3/6-311++G(d,p)-CPCM(DMSO)//UB3LYP-D3/6-31G(d,p)
HF = -424.9088085

5a (UB3LYP)

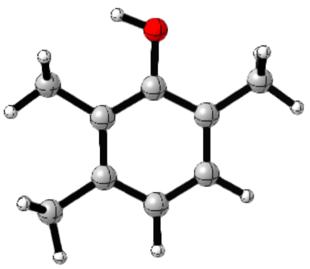

UB3LYP-D3/6-31G(d,p)
Zero-point correction= 0.187955 (Hartree/Particle)
Thermal correction to Energy= 0.198496
Thermal correction to Enthalpy= 0.199440
Thermal correction to Gibbs Free Energy= 0.152644
Sum of electronic and zero-point Energies= -425.266508
Sum of electronic and thermal Energies= -425.255967
Sum of electronic and thermal Enthalpies= -425.255023
Sum of electronic and thermal Free Energies= -425.301820
UB3LYP-D3/6-311++G(d,p)-CPCM(acetonitrile)//UB3LYP-D3/6-31G(d,p)
HF = -425.5448456
UB3LYP-D3/6-311++G(d,p)-CPCM(DMSO)//UB3LYP-D3/6-31G(d,p)
HF =-425.544893

5a' (phenolate)

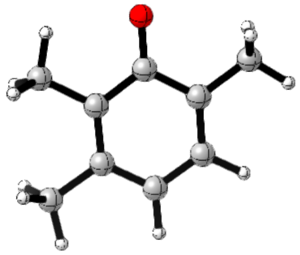

UB3LYP-D3/6-31G(d,p)
Zero-point correction= 0.173610 (Hartree/Particle)
Thermal correction to Energy= 0.183741
Thermal correction to Enthalpy= 0.184685
Thermal correction to Gibbs Free Energy= 0.138778
Sum of electronic and zero-point Energies= -424.698939
Sum of electronic and thermal Energies= -424.688809
Sum of electronic and thermal Enthalpies= -424.687864
Sum of electronic and thermal Free Energies= -424.733772
UB3LYP-D3/6-311++G(d,p)-CPCM(acetonitrile)//UB3LYP-D3/6-31G(d,p)
HF =-425.0587504
UB3LYP-D3/6-311++G(d,p)-CPCM(DMSO)//UB3LYP-D3/6-31G(d,p)
HF = -425.0593639

^{1}TS1-p

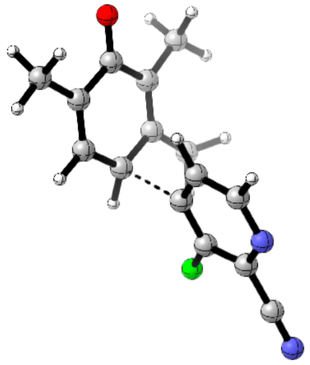

UB3LYP-D3/6-31G(d,p)
Imaginary frequency = -183.74 cm^{-1}
Zero-point correction= 0.243544 (Hartree/Particle)
Thermal correction to Energy= 0.261644
Thermal correction to Enthalpy= 0.262588
Thermal correction to Gibbs Free Energy= 0.195174
Sum of electronic and zero-point Energies= -863.698681
Sum of electronic and thermal Energies= -863.680582
Sum of electronic and thermal Enthalpies= -863.679638
Sum of electronic and thermal Free Energies= -863.747051
UB3LYP-D3/6-311++G(d,p)-CPCM(acetonitrile)//UB3LYP-D3/6-31G(d,p)
HF = -864.140506
UB3LYP-D3/6-311++G(d,p)-CPCM(DMSO)//UB3LYP-D3/6-31G(d,p)
HF = -864.1406394

^{3}TS1-p

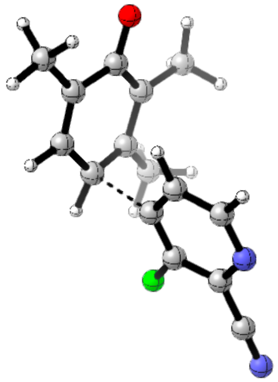

UB3LYP-D3/6-31G(d,p)
Imaginary frequency = -651.36 cm^{-1}
Zero-point correction= 0.240839 (Hartree/Particle)
Thermal correction to Energy= 0.259081
Thermal correction to Enthalpy= 0.260025
Thermal correction to Gibbs Free Energy= 0.192048
Sum of electronic and zero-point Energies= -863.635488
Sum of electronic and thermal Energies= -863.617246
Sum of electronic and thermal Enthalpies= -863.616301
Sum of electronic and thermal Free Energies= -863.684278
UB3LYP-D3/6-311++G(d,p)-CPCM(acetonitrile)//UB3LYP-D3/6-31G(d,p)
HF = -864.0734675
UB3LYP-D3/6-311++G(d,p)-CPCM(DMSO)//UB3LYP-D3/6-31G(d,p)
HF = -864.0736117

^{1}TS1-m

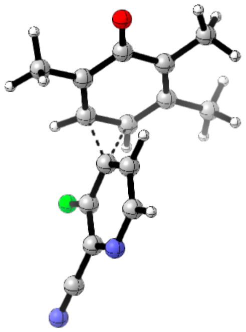

UB3LYP-D3/6-31G(d,p)
Imaginary frequency = -497.26 cm^{-1}
Zero-point correction= 0.243915 (Hartree/Particle)
Thermal correction to Energy= 0.261471
Thermal correction to Enthalpy= 0.262415
Thermal correction to Gibbs Free Energy= 0.197959
Sum of electronic and zero-point Energies= -863.682085
Sum of electronic and thermal Energies= -863.664529
Sum of electronic and thermal Enthalpies= -863.663585
Sum of electronic and thermal Free Energies= -863.728041
UB3LYP-D3/6-311++G(d,p)-CPCM(acetonitrile)//UB3LYP-D3/6-31G(d,p)
HF = -864.1272392

^{3}TS1-m

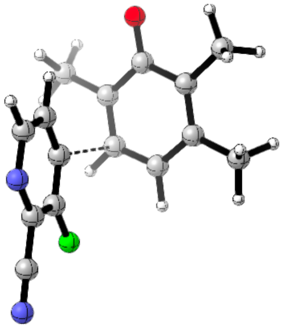

UB3LYP-D3/6-31G(d,p)
Imaginary frequency = -56.39 cm^{-1}
Zero-point correction= 0.241420 (Hartree/Particle)
Thermal correction to Energy= 0.259921
Thermal correction to Enthalpy= 0.260866
Thermal correction to Gibbs Free Energy= 0.190466
Sum of electronic and zero-point Energies= -863.659455
Sum of electronic and thermal Energies= -863.640953
Sum of electronic and thermal Enthalpies= -863.640009
Sum of electronic and thermal Free Energies= -863.710409
UB3LYP-D3/6-311++G(d,p)-CPCM(acetonitrile)//UB3LYP-D3/6-31G(d,p)
HF = -864.098832
UB3LYP-D3/6-311++G(d,p)-CPCM(DMSO)//UB3LYP-D3/6-31G(d,p)
HF =-864.0989555

^{2}TS2-p

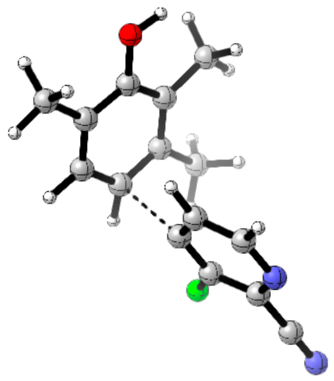

UB3LYP-D3/6-31G(d,p)
Imaginary frequency = -116.6 cm^{-1}
Zero-point correction= 0.254811 (Hartree/Particle)
Thermal correction to Energy= 0.273314
Thermal correction to Enthalpy= 0.274259
Thermal correction to Gibbs Free Energy= 0.205870
Sum of electronic and zero-point Energies= -864.284708
Sum of electronic and thermal Energies= -864.266204
Sum of electronic and thermal Enthalpies= -864.265260
Sum of electronic and thermal Free Energies= -864.333649
UB3LYP-D3/6-311++G(d,p)-CPCM(acetonitrile)//UB3LYP-D3/6-31G(d,p)
UB3LYP-D3/6-311++G(d,p)-CPCM(DMSO)//UB3LYP-D3/6-31G(d,p)

^{2}TS2-m

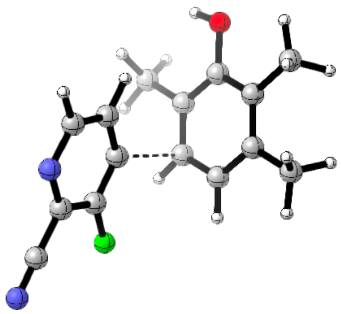

UB3LYP-D3/6-31G(d,p)
Imaginary frequency = -134.72 cm^{-1}
Zero-point correction= 0.254286 (Hartree/Particle)
Thermal correction to Energy= 0.273038
Thermal correction to Enthalpy= 0.273982
Thermal correction to Gibbs Free Energy= 0.202707
Sum of electronic and zero-point Energies= -864.282161
Sum of electronic and thermal Energies= -864.263409
Sum of electronic and thermal Enthalpies= -864.262465
Sum of electronic and thermal Free Energies= -864.333740
UB3LYP-D3/6-311++G(d,p)-CPCM(acetonitrile)//UB3LYP-D3/6-31G(d,p)
HF = -864.7361838
UB3LYP-D3/6-311++G(d,p)-CPCM(DMSO)//UB3LYP-D3/6-31G(d,p)
HF =-864.7363032

^{2}TS3-p

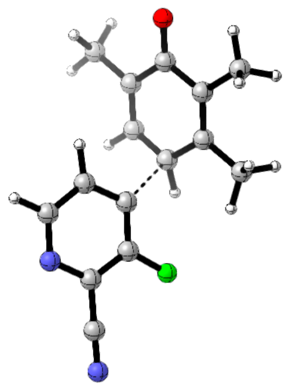

UB3LYP-D3/6-31G(d,p)
Imaginary frequency = -85.92 cm^{-1}
Zero-point correction= 0.241150 (Hartree/Particle)
Thermal correction to Energy= 0.259403
Thermal correction to Enthalpy= 0.260347
Thermal correction to Gibbs Free Energy= 0.191696
Sum of electronic and zero-point Energies= -863.761097
Sum of electronic and thermal Energies= -863.742844
Sum of electronic and thermal Enthalpies= -863.741900
Sum of electronic and thermal Free Energies= -863.810551
UB3LYP-D3/6-311++G(d,p)-CPCM(acetonitrile)//UB3LYP-D3/6-31G(d,p)
HF = -864.7373445
UB3LYP-D3/6-311++G(d,p)-CPCM(DMSO)//UB3LYP-D3/6-31G(d,p)
HF =-864.2688085

^{2}TS3-m

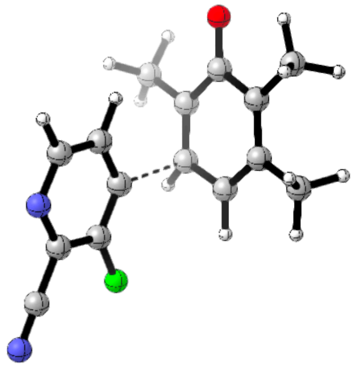

UB3LYP-D3/6-31G(d,p)
Imaginary frequency = -223.51 cm^{-1}
Zero-point correction= 0.240483 (Hartree/Particle)
Thermal correction to Energy= 0.258648
Thermal correction to Enthalpy= 0.259593
Thermal correction to Gibbs Free Energy= 0.191709
Sum of electronic and zero-point Energies= -863.746741
Sum of electronic and thermal Energies= -863.728575
Sum of electronic and thermal Enthalpies= -863.727631
Sum of electronic and thermal Free Energies= -863.795515
UB3LYP-D3/6-311++G(d,p)-CPCM(acetonitrile)//UB3LYP-D3/6-31G(d,p)
HF = -864.2569431
UB3LYP-D3/6-311++G(d,p)-CPCM(DMSO)//UB3LYP-D3/6-31G(d,p)
HF = -864.2574468

Computational References

[i] (a) Lee, C.; Yang, W.; Parr, R. G., Development of the Colle-Salvetti Correlation- Energy Formula into a Functional of the Electron Density. *Phys. Rev. B*. **1988**, *37,* 785-789. (b) Becke, A. D., Density Functional Thermochemistry. III. The Role of Exact Exchange. *J.Chem. Phys*. **1993**, *98*, 5648-5652.

[ii] Grimme, S.; Antony, J.; Ehrlich, S.; Krieg, H. A consistent and accurate ab initio parametrization of density functional dispersion correction (DFT-D) for the 94 elements H-Pu. *J. Chem. Phys*. **2010**, *132*, 154104.

[iii] David, F.; The role of databases in support of computational chemistry calculations. *J. Comput. Chem*. **1996**, *17*, 1571-1586.

[iv] (a) Lee, C.; Yang, W.; Parr, R. G., Development of the Colle-Salvetti Correlation- Energy Formula into a Functional of the Electron Density. *Phys. Rev. B*. **1988**, *37,* 785-789. (b) Becke, A. D., Density Functional Thermochemistry. III. The Role of Exact Exchange. *J.Chem. Phys*. **1993**, *98*, 5648-5652.

[v] (a) McLean, A. D.; Chandler, G. S. Contracted Gaussian-basis sets for molecular calculations. 1. 2nd row atoms, Z=11-18. *J. Chem. Phys*. **1980**, *72*, 5639-5648. (b) Raghavachari, K.; Binkley, J. S.; Seeger, R.; Pople, J. A. Self-Consistent Molecular Orbital Methods. 20. Basis set for correlated wave-functions. *J. Chem. Phys*. **1980**, *72*, 650654.

[vi] Cossi, M.; Rega, N.; Scalmani, G.; Barone, V. Energies, structures, and electronic properties of molecules in solution with the C-PCM solvation model. *J. Comput. Chem*. **2003**, *24*, 669-681.

[vii] Garwood, J. J. A.; Chen, A. D.; Nagib, D. A. Radical Polarity. *J. Am. Chem. Soc.* **2024**, *146*, 28034-28059

[viii] CYLview20; Legault, C. Y., Université de Sherbrooke, 2020 (http://www.cylview.org).

15. X-Ray Crystallography Data

X-Ray Structure Determination of Compound 7a'

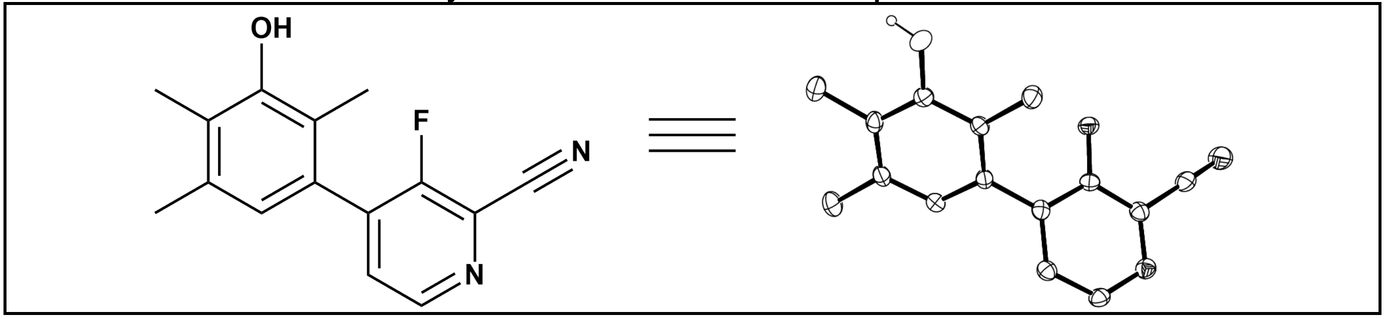

After following general procedure D and obtaining the title compounds **7a** and **7a'** in 75% yield (1.54 g) with 5.0:1 **7a**:**7a'** by $^{19}F\{^{1}H\}$-NMR, the material was subjected to normal-phase column chromatography (30% EtOAc:*n*-hexanes). A single column fraction with enriched **7a'** content (1:1 **7a**:**7a'** by LCMS) was concentrated and recrystallized with a minimum amount of EtOH to afford off-white crystals. From this material, a pure *meta*-isomer **7a'** plate-like crystal was used for analysis. A sample of the pure **7a** isomer from a separate column fraction had two different morphologies (plates and needles), both different from the morphology of crystals from **7a'**.

Compound **7a**', $C_{15}H_{13}FN_2O$, crystallizes in the orthorhombic space group $Pca2_1$ (systematic absences h0l: h=odd, 0kl: l=odd) with a=16.6221(2)Å, b=8.75030(10)Å, c=8.72730(10)Å, α=90°, β=90°, γ=90°, V=1269.37(3)Å^3, Z=4, and d_{calc}=1.341 g/cm^3. X-ray intensity data were collected on a Rigaku XtaLAB Synergy-S diffractometer[i] equipped with an HPC area detector (HyPix-6000HE) and employing confocal multilayer optic-monochromated Cu-Kα radiation (λ=1.54184 Å) at a temperature of 100K. Preliminary indexing was performed from a series of sixty 0.5° rotation frames with exposures of 5 seconds for θ = ±47.612° and 20 seconds for θ = 113.25°. A total of 4668 frames (44 runs) were collected employing ω scans with a crystal to detector distance of 34.0 mm, rotation widths of 0.5° and exposures of 18 seconds.

Rotation frames were integrated using CrysAlisPro,[ii] producing a listing of unaveraged F^2 and $\sigma(F^2)$ values. A total of 19970 reflections were measured over the ranges 10.108 ≤ 2θ ≤ 148.902°, -14 ≤ h ≤ 20, -10 ≤ k ≤ 10, -10 ≤ l ≤ 10 yielding 2556 unique reflections (R_{int} = 0.0400). The intensity data were corrected for Lorentz and polarization effects and for absorption using SCALE3 ABSPACK[iii] (minimum and maximum transmission 0.79673, 1.00000). The structure was solved by dual space methods – SHELXT.[iv] Refinement was by full-matrix least squares based on F^2 using SHELXL.[v] All reflections were used during refinement. The weighting scheme used was $w=1/[\sigma^2(F_o^2)+(0.0356P)^2 + 0.2641P]$ where $P = (F_o^2 + 2F_c^2)/3$. Non-hydrogen atoms were refined anisotropically and hydrogen atoms were refined using a riding model. Refinement converged to R1=0.0287 and wR2=0.0684 for 2412 observed reflections for which F > 4σ(F) and R1=0.0317 and wR2=0.0705 and GOF =1.060 for all 2556 unique, non-zero reflections and 176 variables. The maximum Δ/σ in the final cycle of least squares was 0.000 and the two most prominent peaks in the final difference Fourier were +0.14 and -0.18 e/Å^3.

Table S7. lists cell information, data collection parameters, and refinement data. Final positional and equivalent isotropic thermal parameters are given in **Tables S8** and **S9**. Anisotropic thermal parameters are in **Table S10**. **Tables S11** and **S12** list bond distances and bond angles. **Figure S36** is an ORTEP representation of the molecule with 50% probability thermal ellipsoids displayed.

This report has been created with Olex2,[vi] compiled on 2022.04.07 svn.rca3783a0 for OlexSys.

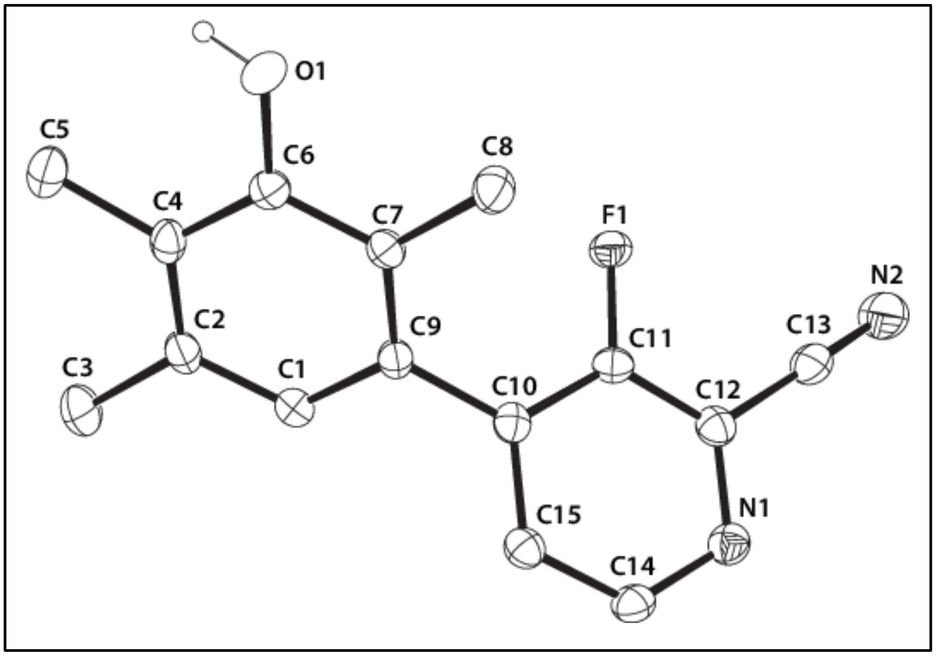

Figure S36. ORTEP drawing of the title compound **7a’** with 50% thermal ellipsoids.

Table S7. Summary of Structure Determination of Compound 7a'

Empirical formula	$C_{15}H_{13}FN_2O$
Formula weight	256.27
Diffractometer	Rigaku XtaLAB Synergy-S (HyPix-6000HE)
Temperature/K	100
Crystal system	orthorhombic
Space group	$Pca2_1$
a	16.6221(2)Å
b	8.75030(10)Å
c	8.72730(10)Å
α	90°
β	90°
γ	90°
Volume	1269.37(3)Å^3
Z	4
d_{calc}	1.341 g/cm^3
μ	0.789 mm^{-1}
F(000)	536.0
Crystal size, mm	0.047 × 0.041 × 0.023
2θ range for data collection	10.108 - 148.902°
Index ranges	-14 ≤ h ≤ 20, -10 ≤ k ≤ 10, -10 ≤ l ≤ 10
Reflections collected	19970
Independent reflections	2556[R(int) = 0.0400]
Data/restraints/parameters	2556/1/176
Goodness-of-fit on F^2	1.060
Final R indexes [I>=2σ (I)]	R_1 = 0.0287, wR_2 = 0.0684
Final R indexes [all data]	R_1 = 0.0317, wR_2 = 0.0705
Largest diff. peak/hole	0.14/-0.18 eÅ^{-3}
Flack parameter	0.02(6)

Table S8. Refined Positional Parameters for Compound 7a'

Atom	*x*	*y*	*z*	U(eq)
F1	0.83686(6)	0.27764(13)	0.45923(14)	0.0243(3)
O1	0.67457(8)	0.73602(15)	0.44417(18)	0.0245(3)
N1	0.80255(10)	-0.04491(18)	0.7048(2)	0.0228(4)
N2	0.98868(11)	0.0284(2)	0.5533(3)	0.0394(5)
C1	0.59610(11)	0.2898(2)	0.4257(2)	0.0208(4)
C2	0.54985(11)	0.4006(2)	0.3532(2)	0.0216(4)
C3	0.47205(13)	0.3553(3)	0.2758(3)	0.0328(5)
C4	0.57544(11)	0.5530(2)	0.3555(2)	0.0210(4)
C5	0.52975(13)	0.6773(3)	0.2728(3)	0.0285(5)
C6	0.64585(11)	0.5894(2)	0.4353(2)	0.0192(4)
C7	0.69143(11)	0.4800(2)	0.5135(2)	0.0179(4)
C8	0.76198(12)	0.5336(2)	0.6075(3)	0.0250(4)
C9	0.66671(11)	0.3275(2)	0.5040(2)	0.0183(4)
C10	0.71367(11)	0.2012(2)	0.5746(2)	0.0185(4)
C11	0.79576(11)	0.1814(2)	0.5504(2)	0.0198(4)
C12	0.83720(11)	0.0608(2)	0.6149(2)	0.0216(4)
C13	0.92204(12)	0.0426(2)	0.5821(3)	0.0268(4)
C14	0.72334(12)	-0.0299(2)	0.7257(2)	0.0226(4)
C15	0.67785(12)	0.0878(2)	0.6634(2)	0.0211(4)

Table S9. Positional Parameters for Hydrogens in Compound 7a'

Atom	*x*	*y*	*z*	U(eq)
H1	0.6565	0.787402	0.37051	0.037
H1A	0.579331	0.186029	0.422014	0.025
H3A	0.475628	0.377415	0.165983	0.049
H3B	0.427438	0.413324	0.320594	0.049
H3C	0.462663	0.245728	0.290812	0.049
H5A	0.52874	0.76969	0.3362	0.043
H5B	0.474542	0.643021	0.253498	0.043
H5C	0.556315	0.699689	0.175139	0.043
H8A	0.805211	0.567536	0.539129	0.037
H8B	0.781424	0.449431	0.671605	0.037
H8C	0.745205	0.61888	0.672969	0.037
H14	0.696571	-0.104056	0.786688	0.027
H15	0.621528	0.090946	0.681627	0.025

Table S10. Refined Thermal Parameters (U's) for Compound 7a'

Atom	U_{11}	U_{22}	U_{33}	U_{23}	U_{13}	U_{12}
F1	0.0220(5)	0.0215(5)	0.0293(6)	0.0054(5)	0.0052(5)	0.0010(4)
O1	0.0294(7)	0.0171(6)	0.0270(8)	0.0033(6)	-0.0041(6)	0.0006(5)
N1	0.0248(8)	0.0188(8)	0.0249(8)	-0.0007(7)	-0.0012(7)	0.0007(7)
N2	0.028(1)	0.0284(10)	0.0619(15)	0.0094(10)	0.0062(9)	0.0078(8)
C1	0.0185(8)	0.0214(9)	0.0224(10)	0.0004(8)	0.0000(8)	-0.0015(7)
C2	0.0158(9)	0.0286(10)	0.0204(9)	0.0008(8)	-0.0008(8)	0.0003(8)
C3	0.0226(10)	0.0380(13)	0.0377(13)	0.0011(10)	-0.0106(9)	-0.0023(9)
C4	0.0172(9)	0.0274(10)	0.0184(9)	0.0027(8)	0.0021(7)	0.0053(7)
C5	0.0268(11)	0.0292(11)	0.0294(11)	0.0038(9)	-0.0039(8)	0.0068(9)
C6	0.0201(8)	0.0194(9)	0.0180(9)	-0.0001(7)	0.0025(7)	0.0008(7)
C7	0.0166(8)	0.0197(9)	0.0174(9)	-0.0013(7)	0.0014(7)	0.0000(7)
C8	0.0259(10)	0.0202(9)	0.0288(10)	-0.0015(8)	-0.0081(8)	0.0000(8)
C9	0.0161(8)	0.0214(9)	0.0175(9)	0.0008(8)	-0.0002(7)	0.0015(7)
C10	0.0194(9)	0.0172(9)	0.0188(9)	-0.0035(7)	-0.0029(7)	-0.0002(7)
C11	0.0215(9)	0.0169(9)	0.0209(10)	-0.0006(7)	0.0010(7)	-0.0021(7)
C12	0.0207(9)	0.0196(9)	0.0244(10)	-0.0020(8)	-0.0003(7)	-0.0005(7)
C13	0.0263(11)	0.0195(10)	0.0345(11)	0.0042(9)	0.0008(9)	0.0035(8)
C14	0.0258(10)	0.0168(9)	0.0251(10)	0.0017(8)	0.0001(8)	-0.0019(7)
C15	0.0204(9)	0.0206(9)	0.0224(10)	-0.0020(8)	-0.0004(7)	-0.0015(7)

Table S11. Bond Distances in Compound 7a', Å

F1-C11	1.345(2)	O1-C6	1.371(2)	N1-C12	1.343(3)
N1-C14	1.336(3)	N2-C13	1.143(3)	C1-C2	1.390(3)
C1-C9	1.398(3)	C2-C3	1.512(3)	C2-C4	1.400(3)
C4-C5	1.509(3)	C4-C6	1.399(3)	C6-C7	1.399(3)
C7-C8	1.506(3)	C7-C9	1.398(3)	C9-C10	1.487(3)
C10-C11	1.392(3)	C10-C15	1.393(3)	C11-C12	1.380(3)
C12-C13	1.448(3)	C14-C15	1.388(3)		

Table S12. Bond Angles in Compound 7a', °

C14-N1-C12	115.77(17)	C2-C1-C9	121.46(17)	C1-C2-C3	119.56(18)
C1-C2-C4	119.36(17)	C4-C2-C3	121.07(18)	C2-C4-C5	121.76(18)
C6-C4-C2	118.56(17)	C6-C4-C5	119.67(18)	O1-C6-C4	122.16(17)
O1-C6-C7	115.13(16)	C7-C6-C4	122.70(17)	C6-C7-C8	118.29(16)
C9-C7-C6	117.72(17)	C9-C7-C8	123.94(17)	C1-C9-C7	120.08(17)
C1-C9-C10	117.90(16)	C7-C9-C10	122.02(16)	C11-C10-C9	122.98(17)
C11-C10-C15	114.50(18)	C15-C10-C9	122.42(16)	F1-C11-C10	120.66(17)
F1-C11-C12	117.80(16)	C12-C11-C10	121.51(18)	N1-C12-C11	123.43(17)
N1-C12-C13	117.23(18)	C11-C12-C13	119.32(18)	N2-C13-C12	178.7(3)
N1-C14-C15	123.81(18)	C14-C15-C10	120.90(18)		

X-Ray References

[i] CrysAlisPro 1.171.44.108a: Rigaku Oxford Diffraction, Rigaku Corporation, Oxford, UK. (2025).
[ii] CrysAlisPro 1.171.44.108a: Rigaku Oxford Diffraction, Rigaku Corporation, Oxford, UK. (2025).
[iii] SCALE3 ABSPACK v1.0.7: an Oxford Diffraction program; Oxford Diffraction Ltd: Abingdon, UK, 2005.
[iv] SHELXT v2018/2: Sheldrick, G.M., Acta Cryst., A, 71, 3-8 (2015).
[v] SHELXL-2019/3: Sheldrick, G.M., Acta Cryst., A, 71, 3-8 (2015).
[vi] Olex2: Dolomanov,O.V., Bourhis, L.J., Gildea, R.J., Howard, J.A.K., Puschmann, H., *J. Appl. Cryst.* **2009**, 42, 339-341.

16. References

[1] T. Aubineau, J. Laurent, L. Olanier, A. Guérinot, *Chem.-Methods* **2023**, *3*, e202300002, https://doi.org/10.1002/cmtd.202300002.
[2] H.-H. Li, S. Li, J. K. Cheng, S.-H. Xiang, B. Tan, *Chem. Commun.* **2022**, *58*, 4392-4395, https://doi.org/10.1039/d2cc01212j.
[3] D.-L. Zhu, S. Jiang, D. J. Young, Q. Wu, H.-Y. Li, H.-X. Li, *Chem. Commun.* **2022**, *58*, 3637-3640, https://doi.org/10.1039/d1cc07127k.
[4] Y. Tan, M. Pei, K. Yang, T. Zhou, A. Hu, J.-J. Guo, *Org. Lett.* **2024**, *26*, 8084-8089, https://doi.org/10.1021/acs.orglett.4c02985.
[5] https://www.ambeed.com/products/112930-95-7.html.
[6] M. E. Schnute, M. Wennerstål, J. Alley, M. Bengtsson, J. R. Blinn, C. W. Bolten, T. Braden, T. Bonn, B. Carlsson, N. Caspers, M. Chen, C. Choi, L. P. Collis, K. Crouse, M. Färnegårdh, K. F. Fennell, S. Fish, A. C. Flick, A. Goos-Nilsson, H. Gullberg, P. K. Harris, S. E. Heasley, M. Hegen, A. E. Hromockyj, X. Hu, B. Husman, T. Janosik, P. Jones, N. Kaila, E. Kallin, B. Kauppi, J. R. Kiefer, J. Knafels, K. Koehler, L. Kruger, R. G. Kurumbail, J. Robert E. Kyne, W. Li, J. Löfstedt, S. A. Long, C. A. Menard, S. Mente, D. Messing, M. J. Meyers, L. Napierata, D. Nöteberg, P. Nuhant, M. J. Pelc, M. J. Prinsen, P. Rhönnstad, E. Backström-Rydin, J. Sandberg, M. Sandström, F. Shah, M. Sjöberg, A. Sundell, A. P. Taylor, A. Thorarensen, J. I. Trujillo, J. D. Trzupek, R. Unwalla, F. F. Vajdos, R. A. Weinberg, D. C. Wood, L. Xing, E. Zamaratski, C. W. Zapf, Y. Zhao, A. Wilhelmsson, G. Berstein, *J. Med. Chem.* **2018**, *61*, 10415-10439, https://doi.org/10.1021/acs.jmedchem.8b00392.
[7] J. J. A. C. Garwood, Andrew D.; Nagib, David A., *J. Am. Chem. Soc.* **2024**, *146*, 28034-28059, https://doi.org/10.1021/jacs.4c06774.
[8] Q. Shi, X. Huang, R. Yang, W. H. Liu, *Chem. Sci.* **2024**, *15*, 12442-12450, https://doi.org/10.1039/d4sc03739a.
[9] H. E. Askey, J. D. Grayson, J. D. Tibbetts, J. C. Turner-Dore, J. M. Holmes, G. Kociok-Kohn, G. L. Wrigley, A. J. Cresswell, *J. Am. Chem. Soc.* **2021**, *143*, 15936-15945, https://doi.org/10.1021/jacs.1c07401.
[10] The pandas development team. “pandas-dev/pandas: Pandas”, **2020**, *latest*, https://doi.org/10.5281/zenodo.3509134.
[11] M. Waskom, *J. Open Source Softw.* **2021**, *6*, 3021, https://doi.org/10.21105/joss.03021.
[12] a) F. V. Pedregosa, Gaël; Gramfort, Alexandre; Michel, Vincent; Thirion, Bertrand; Grisel, Olivier; Blondel, Mathieu; Prettenhofer, Peter; Weiss, Ron; Dubourg, Vincent; Vanderplas, Jake; Passos, Alexandre; Cournapeau, David; Brucher, Matthieu; Perrot, Matthieu; Duchesnay, Édouard, *J. Mach. Learn. Res.* **2011**, *12*, 2825-2830. b) J. D. Hunter, *Comput. Sci. Eng.* **2007**, *9*, 90-95, https://doi.org/10.1109/MCSE.2007.55.
[13] a) *Rowan Scientific*, can be found under https://www.rowansci.com **2025** (accessed 2025-10-03). b) C. Wagen, A. Wagen, *ChemRxiv* **2024**, https://doi.org/10.26434/chemrxiv-2024-8489b. c) H. Neugebauer, F. Bohle, M. Bursch, A. Hansen, S. Grimme, *J. Phys. Chem. A* **2020**, *124*, 7166-7176, https://doi.org/10.1021/acs.jpca.0c05052. d) C. Bannwarth, S. Ehlert, S. Grimme, *J. Chem. Theory Comput.* **2019**, *15*, 1652-1671, https://doi.org/10.1021/acs.jctc.8b01176.

17. HRMS Data

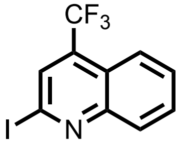

2-Iodo-4-(trifluoromethyl)quinoline (**6c'**)

HRMS (ESI-FTICR) *m/z* = 323.9492 calcd for $C_{10}H_6F_3IN^+$ $[M+H]^+$, found 323.9493.

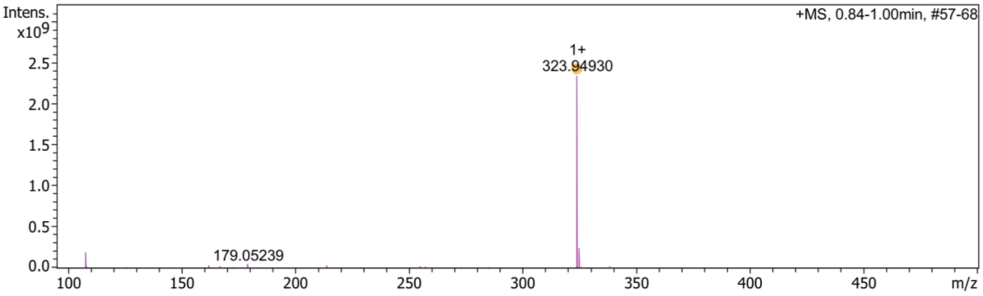

Meas. m/z	#	Ion Formula	Score	m/z	err [ppm]	|err| [mDa]	Mean err [ppm]	mSigma	rdb (neutral)	e⁻ Conf	N-Rule
323.949297	1	C10H6F3IN	100.00	323.949157	-0.4	0.1	-0.9	3.5	10.0	even	ok

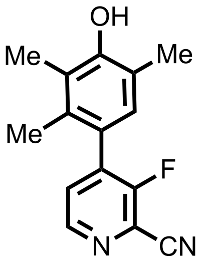

3-Fluoro-4-(4-hydroxy-2,3,5-trimethylphenyl)picolinonitrile (**7a**)

HRMS (ESI-FTICR) *m/z* = 255.0939 calcd for $C_{15}H_{12}FN_2O^-$ [M–H]$^-$, found 255.0941.

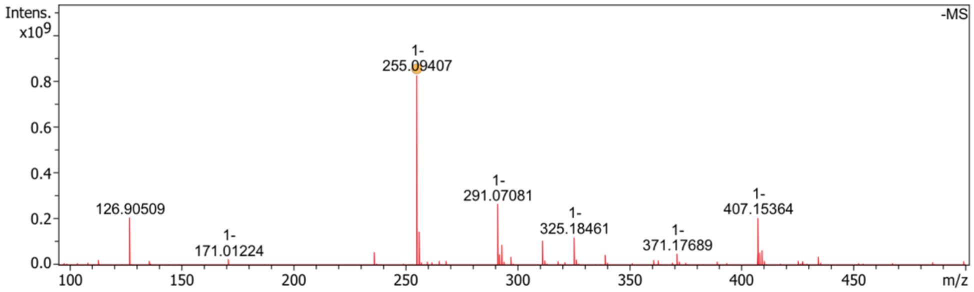

Meas. m/z	#	Ion Formula	Score	m/z	err [ppm]	|err| [mDa]	Mean err [ppm]	mSigma	rdb (neutral)	e⁻ Conf	N-Rule
255.094071	1	C15H12FN2O	100.00	255.093915	-0.6	0.2	-0.9	5.7	10.0	even	ok
255.094071	2	C10H11F2N5O	33.53	255.093715	-1.4	0.4	-1.7	38.3	6.5	odd	ok

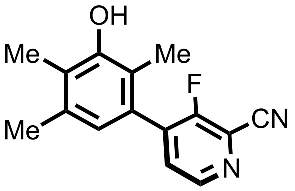

3-Fluoro-4-(3-hydroxy-2,4,5-trimethylphenyl)picolinonitrile (**7a'**)

HRMS (ESI-FTICR) *m/z* = 257.1085 calcd for $C_{15}H_{14}FN_2O^+$ [M+H]$^+$, found 257.1078.

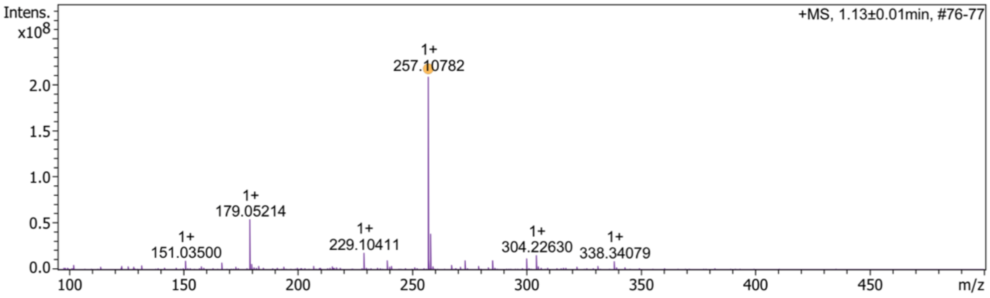

Meas. m/z	#	Ion Formula	Score	m/z	err [ppm]	Mean err [ppm]	mSigma	rdb (neutral)	e⁻ Conf	N-Rule
257.107825	1	C15H14FN2O	100.00	257.108468	2.5	2.2	10.5	10.0	even	ok

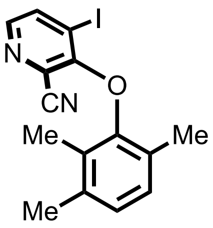

4-Iodo-3-(2,3,6-trimethylphenoxy)picolinonitrile (**7aa**)

HRMS (ESI-FTICR) *m/z* = 365.0145 calcd for $C_{15}H_{14}IN_2O^+$ [M+H]$^+$, found 365.0146.

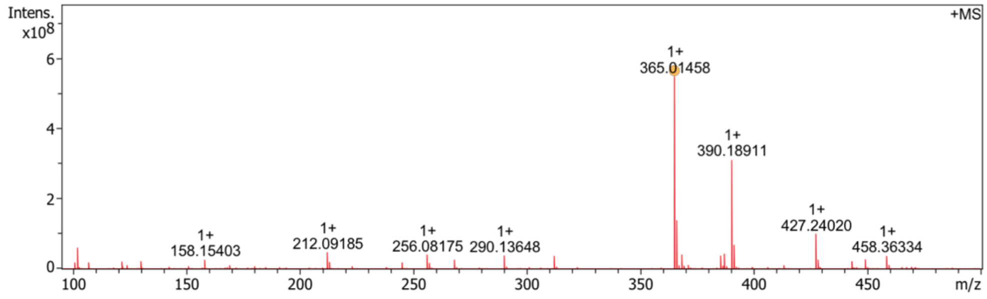

Meas. m/z	#	Ion Formula	Score	m/z	err [ppm]	|err| [mDa]	Mean err [ppm]	mSigma	rdb (neutral)	e⁻ Conf	N-Rule
365.014582	1	C15H14IN2O	100.00	365.014536	-0.1	0.0	-0.7	51.3	13.0	even	ok

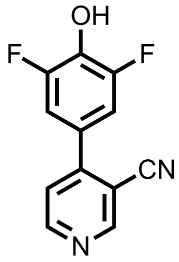

4-(3,5-Difluoro-4-hydroxyphenyl)nicotinonitrile (**7b**)

HRMS (ESI-FTICR) *m/z* = 233.05209 calcd for $C_{12}H_7F_2N_2O^+$ [M+H]$^+$, found 233.05213.

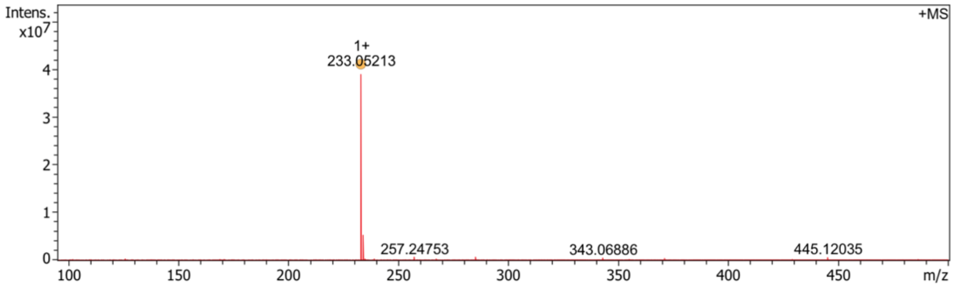

Meas. m/z	#	Ion Formula	Score	m/z	err [ppm]	|err| [mDa]	Mean err [ppm]	mSigma	rdb (neutral)	e⁻ Conf	N-Rule
233.052131	1	C12H7F2N2O	100.00	233.052096	-0.2	0.0	-0.3	1.3	10.0	even	ok
233.052131	2	C7H6F3N5O	35.75	233.051896	-1.0	0.2	-1.1	33.1	6.5	odd	ok

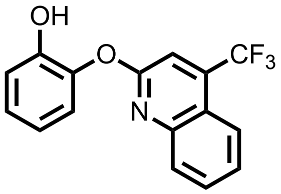

2-((4-(Trifluoromethyl)quinolin-2-yl)oxy)phenol (**7c**)

HRMS (ESI-FTICR) *m/z* = 306.07364 calcd for $C_{16}H_{11}F_3NO_2^+$ [M+H]$^+$, found 306.07366.

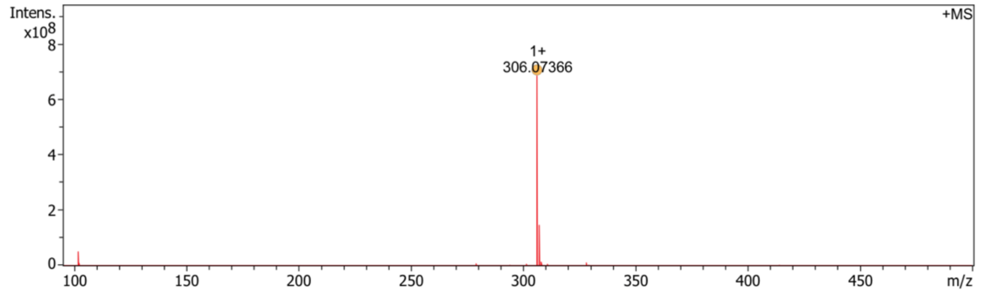

Meas. m/z	#	Ion Formula	Score	m/z	err [ppm]	|err| [mDa]	Mean err [ppm]	mSigma	rdb (neutral)	e⁻ Conf	N-Rule
306.073659	1	C16H11F3NO2	100.00	306.073640	-0.1	0.0	-0.3	24.4	11.0	even	ok

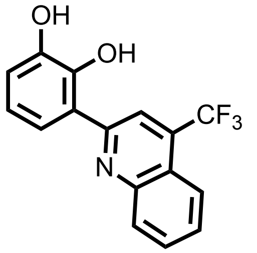

3-(4-(Trifluoromethyl)quinolin-2-yl)benzene-1,2-diol (**7c'**)

HRMS (ESI-FTICR) *m/z* = 306.07364 calcd for $C_{16}H_{11}F_3NO_2^+$ [M+H]$^+$, found 306.07361.

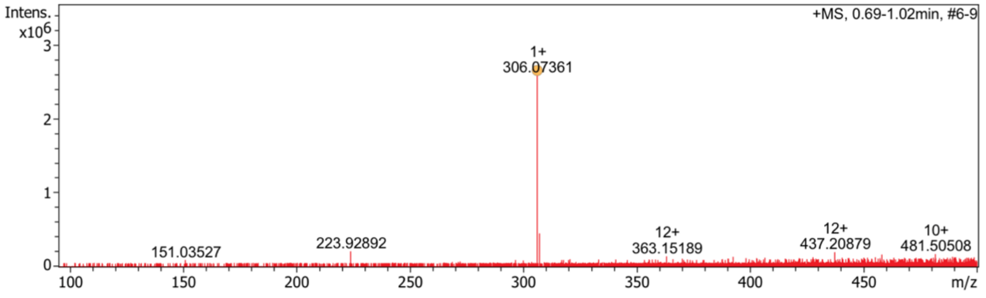

Meas. m/z	#	Ion Formula	Score	m/z	err [ppm]	Mean err [ppm]	mSigma	rdb (neutral)	e⁻ Conf	N-Rule
306.073612	1	C16H11F3NO2	100.00	306.073640	0.1	0.0	10.6	11.0	even	ok

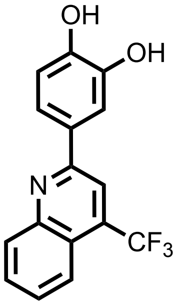

4-(4-(Trifluoromethyl)quinolin-2-yl)benzene-1,2-diol (**7c''**)

HRMS (ESI-FTICR) *m/z* = 306.07364 calcd for $C_{16}H_{11}F_3NO_2^+$ [M+H]$^+$, found 306.07359.

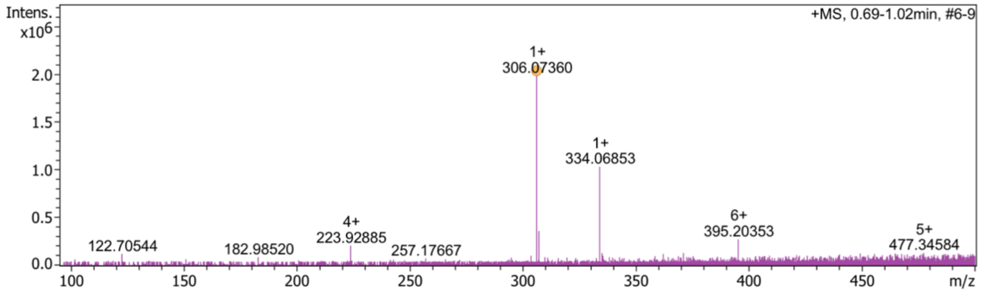

Meas. m/z	#	Ion Formula	Score	m/z	err [ppm]	Mean err [ppm]	mSigma	rdb (neutral)	e⁻ Conf	N-Rule
306.073596	1	C16H11F3NO2	100.00	306.073640	0.1	2.3	5.0	11.0	even	ok

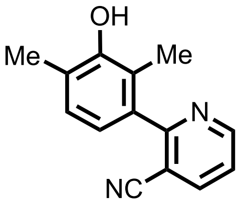

2-(3-Hydroxy-2,4-dimethylphenyl)nicotinonitrile (**7d**)

HRMS (ESI-FTICR) *m/z* = 225.10224 calcd for $C_{14}H_{13}N_2O^+$ [M+H]$^+$, found 225.10220.

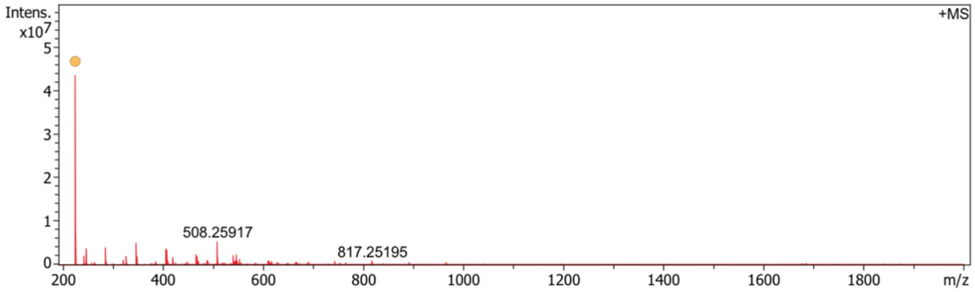

Meas. m/z	#	Ion Formula	Score	m/z	err [ppm]	|err| [mDa]	Mean err [ppm]	mSigma	rdb	e⁻ Conf	N-Rule
225.102202	1	C14H13N2O	100.00	225.102239	0.2	0.0	0.2	4.1	10.0	even	ok

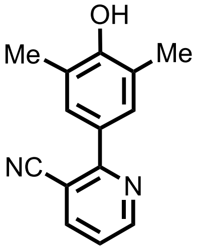

2-(4-Hydroxy-3,5-dimethylphenyl)nicotinonitrile (**7d'**)

HRMS (ESI-FTICR) *m/z* = 225.102239 calcd for $C_{14}H_{13}N_2O^+$ [M+H]$^+$, found 225.102236.

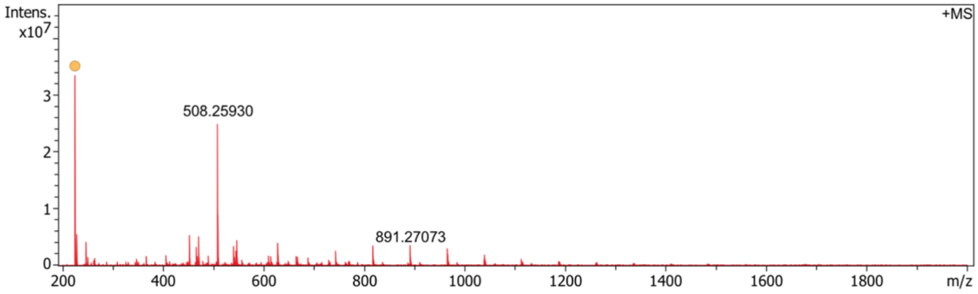

Meas. m/z	#	Ion Formula	Score	m/z	err [ppm]	|err| [mDa]	Mean err [ppm]	mSigma	rdb	e⁻ Conf	N-Rule
225.102236	1	C14H13N2O	100.00	225.102239	0.0	0.0	0.1	2.8	10.0	even	ok

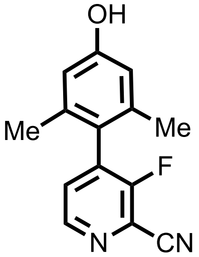

3-Fluoro-4-(4-hydroxy-2,6-dimethylphenyl)picolinonitrile (**7d''**)

HRMS (ESI-Orbitrap) *m/z* = 243.0928 calcd for $C_{14}H_{12}FN_2O^+$ [M+H]$^+$, found 243.0925.

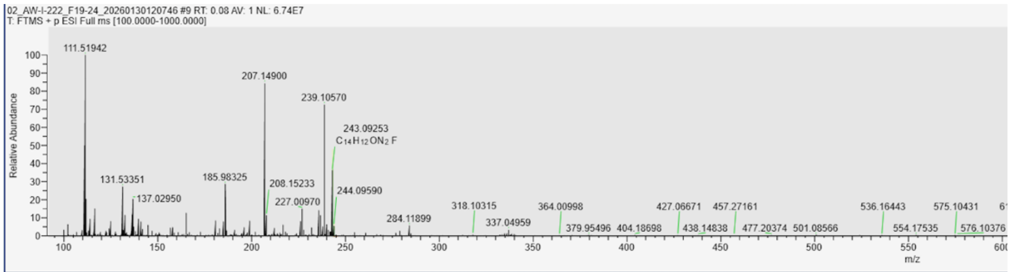

Elemental Composition Results - Spectrum 1

Peak Mass	Display Formula	S Fit	RDB	Delta [ppm]	Theo. mass	Rank
243.09253	$C_{12}H_{10}N_5F$	24.20	10.00	4.34	243.09148	1
243.09253	$C_{14}H_{12}ON_2F$	15.36	9.50	-1.19	243.09282	2

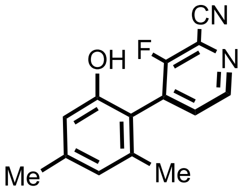

3-Fluoro-4-(2-hydroxy-4,6-dimethylphenyl)picolinonitrile (**7d’’’**)

HRMS (ESI-Orbitrap) *m/z* = 243.0928 calcd for $C_{14}H_{12}FN_2O^+$ [M+H]$^+$, found 243.0923.

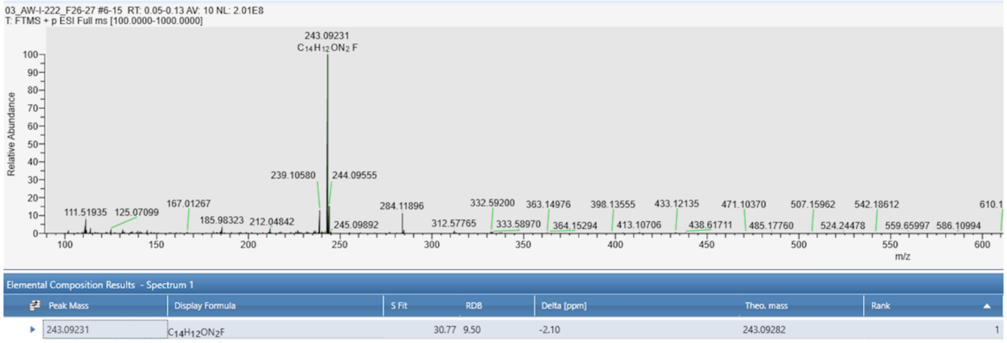

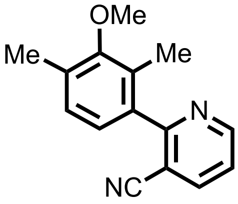

2-(3-Methoxy-2,4-dimethylphenyl)nicotinonitrile (**7e**)

HRMS (ESI-FTICR) *m/z* = 239.11789 calcd for $C_{15}H_{15}N_2O^+$ [M+H]$^+$, found 239.11792.

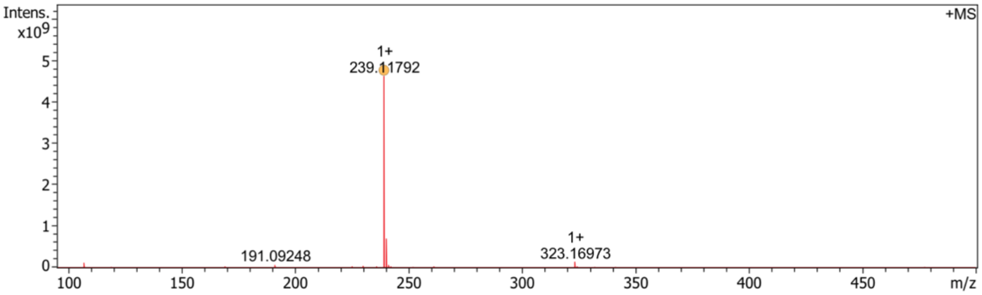

Meas. m/z	#	Ion Formula	Score	m/z	err [ppm]	|err| [mDa]	Mean err [ppm]	mSigma	rdb (neutral)	e⁻ Conf	N-Rule
239.117923	1	C15H15N2O	100.00	239.117890	-0.1	0.0	-0.6	6.6	10.0	even	ok

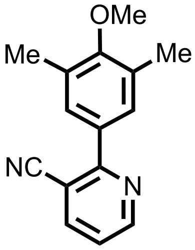

2-(4-Methoxy-3,5-dimethylphenyl)nicotinonitrile (**7e'**)

HRMS (ESI-FTICR) *m/z* = 239.1179 calcd for $C_{15}H_{15}N_2O^+$ [M+H]$^+$, found 239.1178.

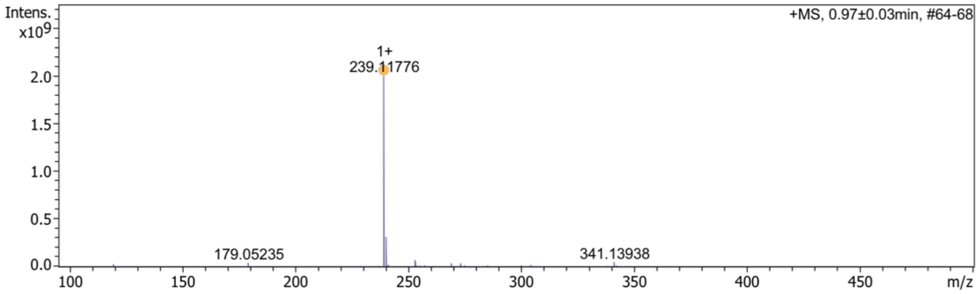

Meas. m/z	#	Ion Formula	Score	m/z	err [ppm]	Mean err [ppm]	mSigma	rdb (neutral)	e⁻ Conf	N-Rule
239.117763	1	C15H15N2O	100.00	239.117890	0.5	0.4	4.4	10.0	even	ok

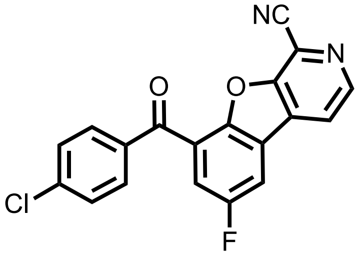

8-(4-Chlorobenzoyl)-6-fluorobenzofuro[2,3-c]pyridine-1-carbonitrile (**7l**)

HRMS (ESI-FTICR) *m/z* = 351.0331 calcd for $C_{19}H_9ClFN_2O_2^+$ [M+H]$^+$, found 351.0329.

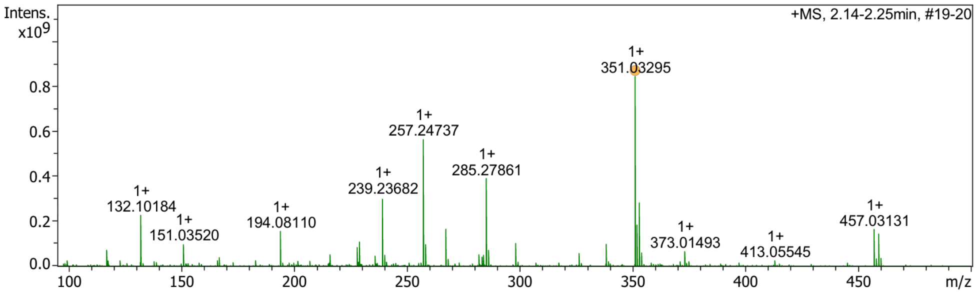

Meas. m/z	#	Ion Formula	Score	m/z	err [ppm]	Mean err [ppm]	mSigma	rdb (neutral)	e⁻ Conf	N-Rule
351.032949	1	C19H9ClFN2O2	100.00	351.033110	0.5	0.3	13.8	19.0	even	ok

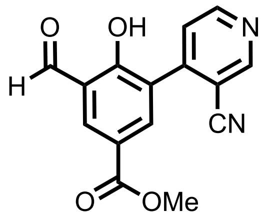

Methyl 3-(3-cyanopyridin-4-yl)-5-formyl-4-hydroxybenzoate (**7m**)

HRMS (ESI-FTICR) *m/z* = 283.0713 calcd for $C_{15}H_{11}N_2O_4^+$ [M+H]$^+$, found 283.0715.

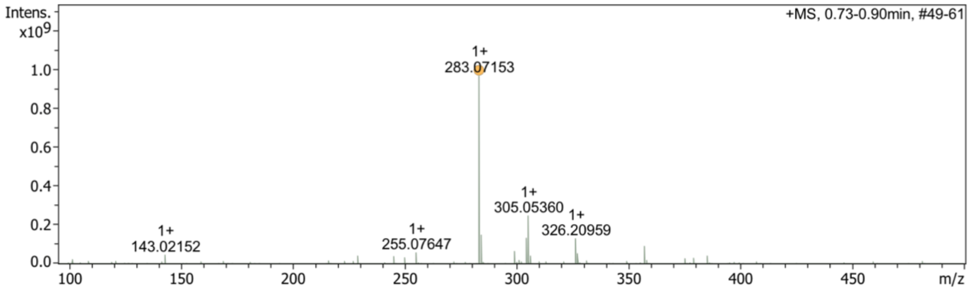

Meas. m/z	#	Ion Formula	Score	m/z	err [ppm]	Mean err [ppm]	mSigma	rdb (neutral)	e⁻ Conf	N-Rule
283.071533	1	C15H11N2O4	100.00	283.071333	-0.7	0.4	6.2	12.0	even	ok

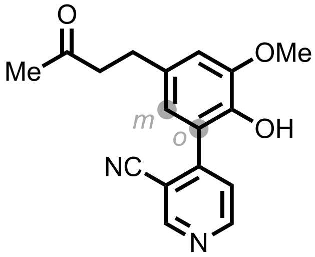

4-(2-Hydroxy-3-methoxy-5-(3-oxobutyl)phenyl)nicotinonitrile (**7n**)

HRMS (ESI-FTICR) *m/z* = 297.1234 calcd for $C_{17}H_{17}N_2O_3^+$ [M+H]$^+$, found 297.1240.

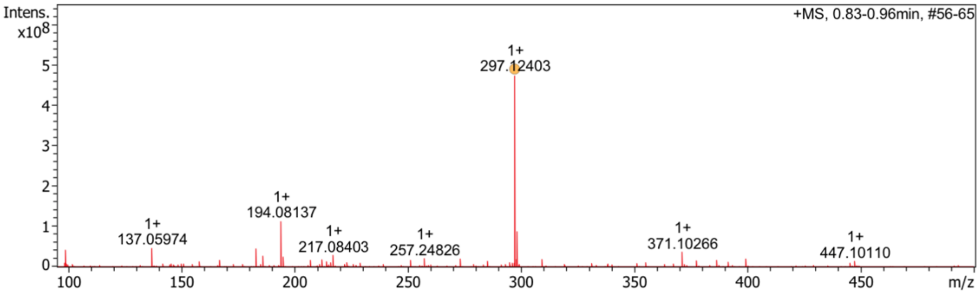

Meas. m/z	#	Ion Formula	Score	m/z	err [ppm]	|err| [mDa]	Mean err [ppm]	mSigma	rdb (neutral)	e⁻ Conf	N-Rule
297.124030	1	C17H17N2O3	100.00	297.123369	-2.2	0.7	-2.5	4.0	11.0	even	ok

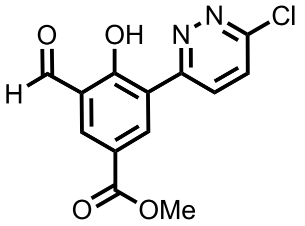

Methyl 3-(6-chloropyridazin-3-yl)-5-formyl-4-hydroxybenzoate (**7o**)

HRMS (ESI-FTICR) *m/z* = 293.0323 calcd for $C_{13}H_{10}ClN_2O_4^+$ [M+H]$^+$, found 293.0324.

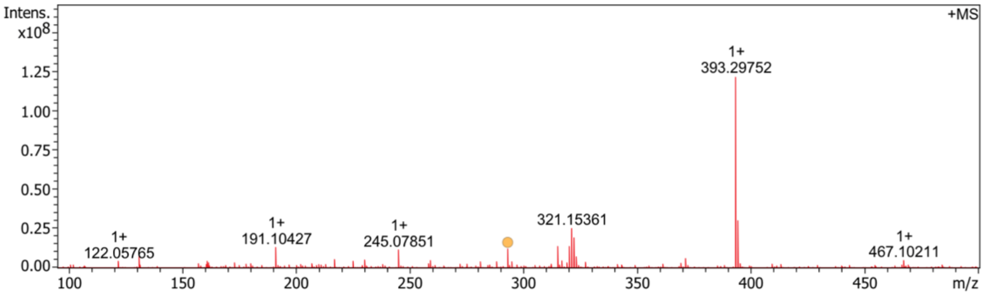

Meas. m/z	#	Ion Formula	Score	m/z	err [ppm]	|err| [mDa]	Mean err [ppm]	mSigma	rdb (neutral)	e⁻ Conf	N-Rule
293.032438	1	C13H10ClN2O4	100.00	293.032361	-0.3	0.1	-0.4	9.3	13.0	even	ok

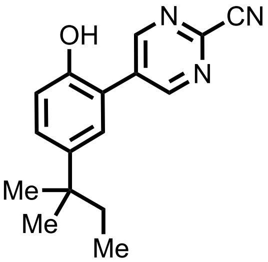

5-(2-Hydroxy-5-(tert-pentyl)phenyl)pyrimidine-2-carbonitrile (**7p**)

HRMS (ESI-FTICR) *m/z* = 268.1444 calcd for $C_{16}H_{18}N_3O^+$ [M+H]$^+$, found 268.1446.

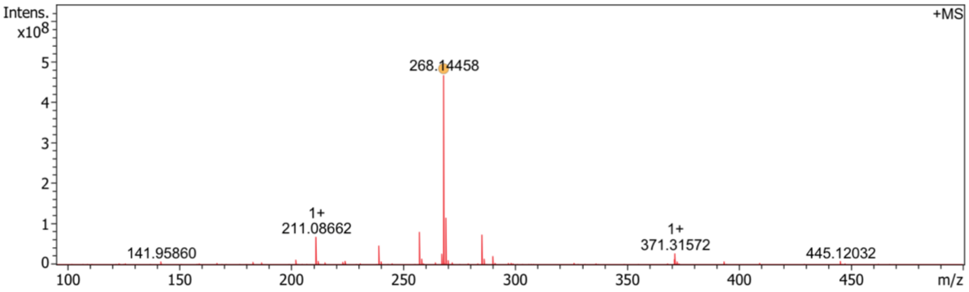

Meas. m/z	#	Ion Formula	Score	m/z	err [ppm]	|err| [mDa]	Mean err [ppm]	mSigma	rdb (neutral)	e⁻ Conf	N-Rule
268.144578	1	C16H18N3O	100.00	268.144439	-0.5	0.1	-0.5	37.5	10.0	even	ok

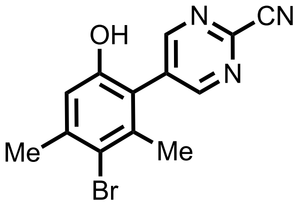

5-(3-Bromo-6-hydroxy-2,4-dimethylphenyl)pyrimidine-2-carbonitrile (**7q**)

HRMS (ESI-FTICR) *m/z* = 304.0080 calcd for $C_{13}H_{11}BrN_3O^+$ $[M+H]^+$, found 304.0079.

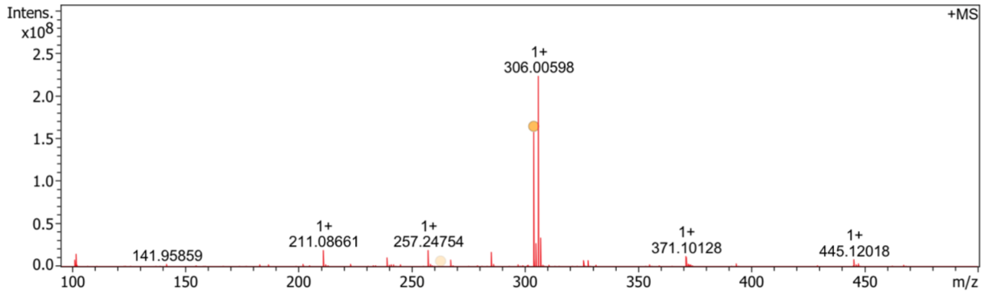

Meas. m/z	#	Ion Formula	Score	m/z	err [ppm]	|err| [mDa]	Mean err [ppm]	mSigma	rdb (neutral)	e⁻ Conf	N-Rule
304.007948	1	C13H11BrN3O	100.00	304.008001	0.2	0.1	-0.1	111.1	12.0	even	ok

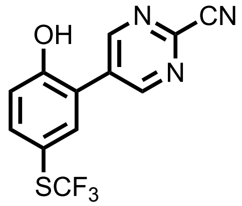

5-(2-Hydroxy-5-((trifluoromethyl)thio)phenyl)pyrimidine-2-carbonitrile (**7r**)

HRMS (ESI-FTICR) *m/z* = 298.0256 calcd for $C_{12}H_7F_3N_3OS^+$ $[M+H]^+$, found 298.0255.

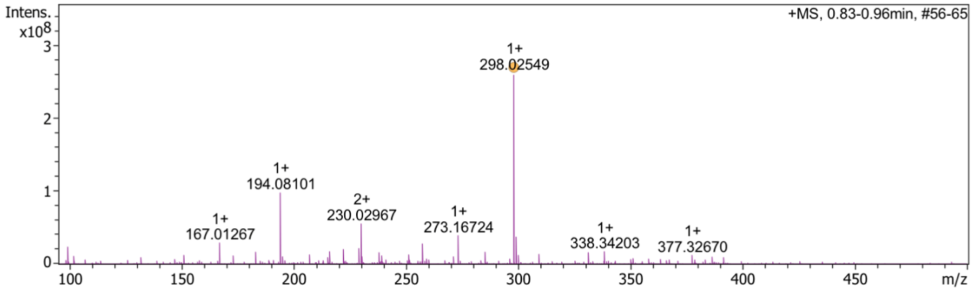

Meas. m/z	#	Ion Formula	Score	m/z	err [ppm]	Mean err [ppm]	mSigma	rdb (neutral)	e⁻ Conf	N-Rule
298.025491	1	C12H7F3N3OS	100.00	298.025644	0.5	-1.0	5.2	12.0	even	ok

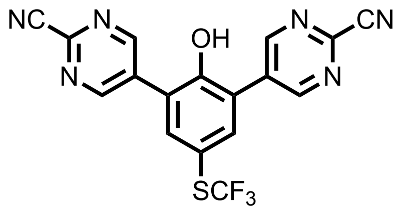

5,5'-(2-Hydroxy-5-((trifluoromethyl)thio)-1,3-phenylene)bis(pyrimidine-2-carbonitrile) (**7r'**)

HRMS (ESI-FTICR) *m/z* = 401.04269 calcd for $C_{17}H_8F_3N_6OS^+$ [M+H]$^+$, found 401.04274.

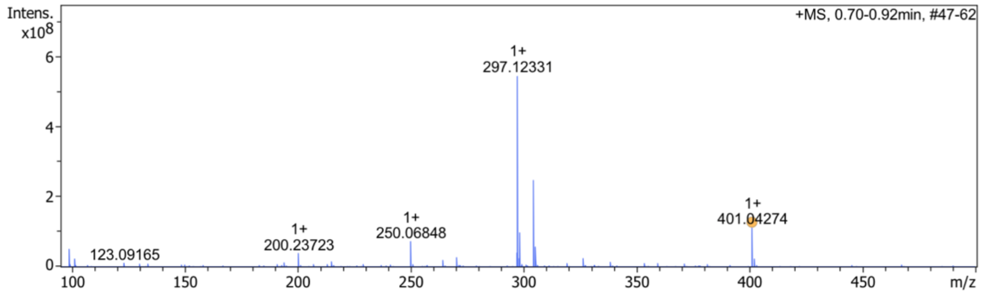

Meas. m/z	#	Ion Formula	Score	m/z	err [ppm]	Mean err [ppm]	mSigma	rdb (neutral)	e⁻ Conf	N-Rule
401.042740	1	C17H8F3N6OS	100.00	401.042691	-0.1	-0.6	14.1	18.0	even	ok

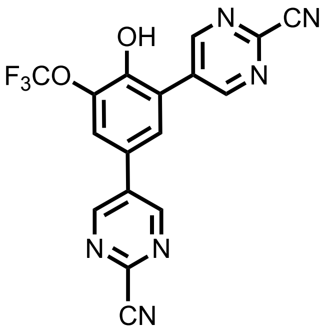

5,5'-(4-Hydroxy-5-(trifluoromethoxy)-1,3-phenylene)bis(pyrimidine-2-carbonitrile) (**7s**)

HRMS (ESI-FTICR) *m/z* = 385.0655 calcd for $C_{17}H_8F_3N_6O_2^+$ [M+H]$^+$, found 385.0664.

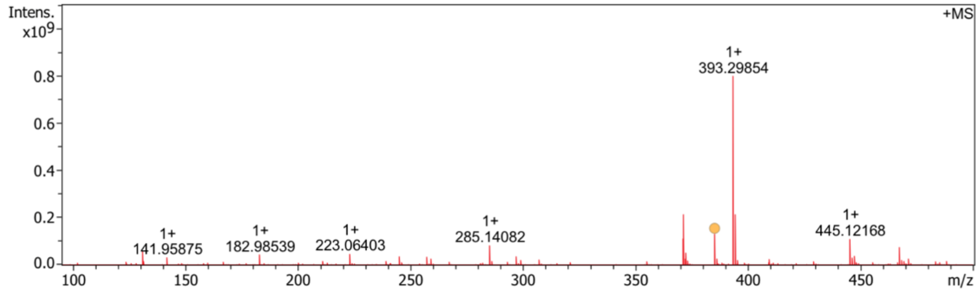

Meas. m/z	#	Ion Formula	Score	m/z	err [ppm]	|err| [mDa]	Mean err [ppm]	mSigma	rdb (neutral)	e⁻ Conf	N-Rule
385.066447	1	C17H8F3N6O2	100.00	385.065534	-2.4	0.9	-3.3	10.1	16.0	even	ok

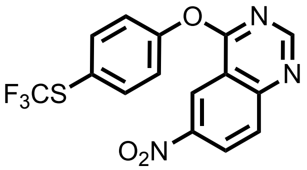

6-Nitro-4-(4-((trifluoromethyl)thio)phenoxy)quinazoline (**7t**)

HRMS (ESI-FTICR) *m/z* = 368.0311 calcd for $C_{15}H_9F_3N_3O_3S^+$ [M+H]$^+$, found 368.0310.

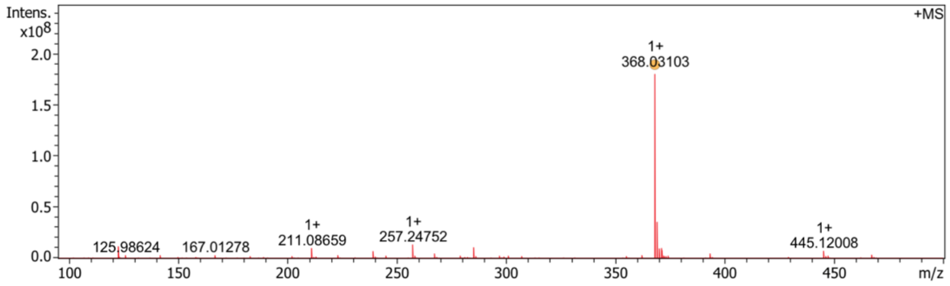

Meas. m/z	#	Ion Formula	Score	m/z	err [ppm]	|err| [mDa]	Mean err [ppm]	mSigma	rdb (neutral)	e¯ Conf	N-Rule
368.031029	1	C15H9F3N3O3S	100.00	368.031123	0.3	0.1	-0.7	12.6	14.0	even	ok

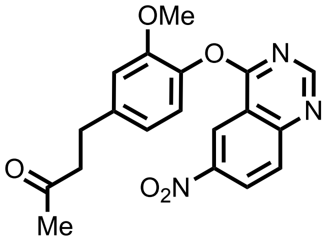

4-(3-Methoxy-4-((6-nitroquinazolin-4-yl)oxy)phenyl)butan-2-one (**7u**)

HRMS (ESI-FTICR) *m/z* = 368.1241 calcd for $C_{19}H_{18}N_3O_5^+$ [M+H]$^+$, found 368.1247.

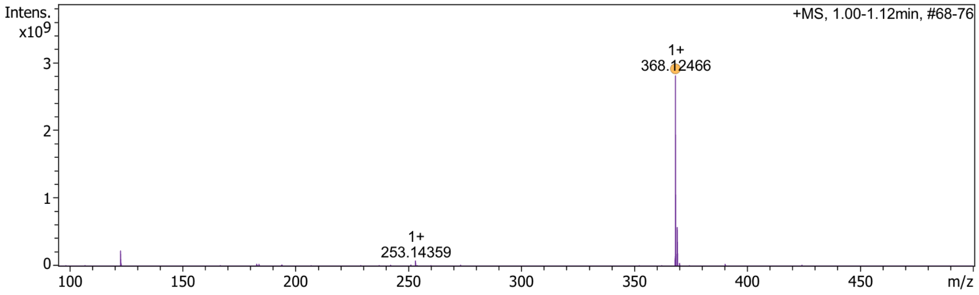

Meas. m/z	#	Ion Formula	Score	m/z	err [ppm]	Mean err [ppm]	mSigma	rdb (neutral)	e¯ Conf	N-Rule
368.124665	1	C19H18N3O5	100.00	368.124097	-1.5	-1.4	9.4	13.0	even	ok

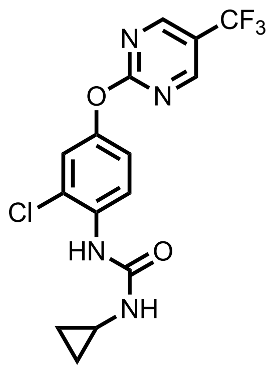

1-(2-Chloro-4-((5-(trifluoromethyl)pyrimidin-2-yl)oxy)phenyl)-3-cyclopropylurea (**7v**)
HRMS (ESI-FTICR) *m/z* = 373.0674 calcd for $C_{15}H_{13}ClF_3N_4O_2^+$ [M+H]$^+$, found 373.0678.

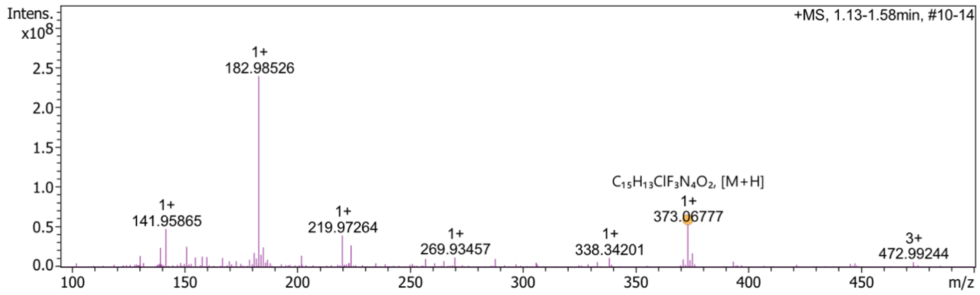

Meas. m/z	#	Ion Formula	Score	m/z	err [ppm]	|err| [mDa]	Mean err [ppm]	mSigma	rdb (neutral)	e⁻ Conf	N-Rule
373.067775	1	C15H13ClF3N4O2	100.00	373.067364	-1.1	0.4	-2.3	9.0	13.0	even	ok

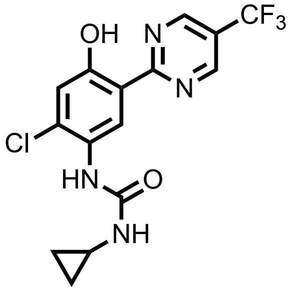

1-(2-Chloro-4-hydroxy-5-(5-(trifluoromethyl)pyrimidin-2-yl)phenyl)-3-cyclopropylurea (**7v'**)
HRMS (ESI-FTICR) *m/z* = 373.06736 calcd for $C_{15}H_{13}ClF_3N_4O_2^+$ [M+H]$^+$, found 373.06741.

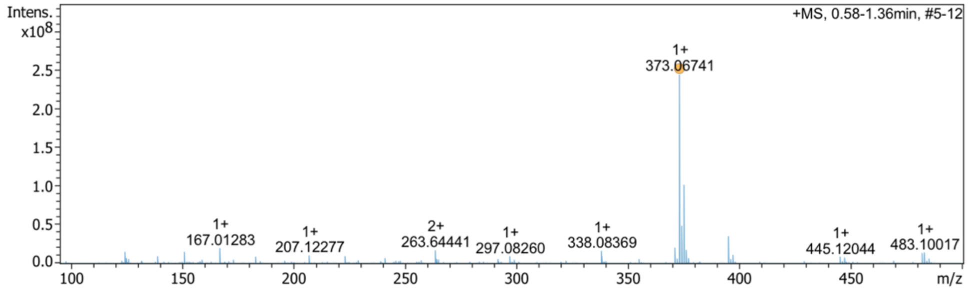

Meas. m/z	#	Ion Formula	Score	m/z	err [ppm]	Mean err [ppm]	mSigma	rdb (neutral)	e⁻ Conf	N-Rule
373.067409	1	C15H13ClF3N4O2	100.00	373.067364	-0.1	-1.1	42.3	13.0	even	ok

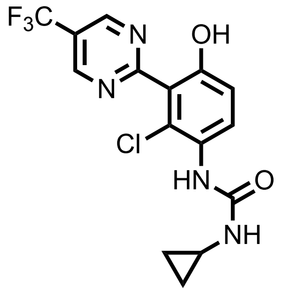

1-(2-Chloro-4-hydroxy-3-(5-(trifluoromethyl)pyrimidin-2-yl)phenyl)-3-cyclopropylurea (**7v''**)
HRMS (ESI-FTICR) *m/z* = 373.06736 calcd for $C_{15}H_{13}ClF_3N_4O_2^+$ [M+H]$^+$, found 373.06742.

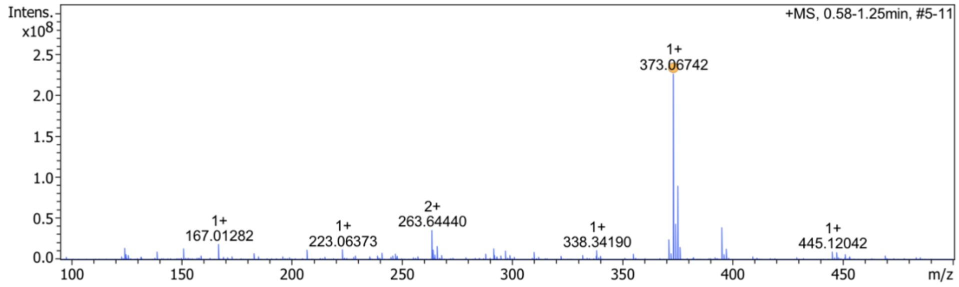

Meas. m/z	#	Ion Formula	Score	m/z	err [ppm]	Mean err [ppm]	mSigma	rdb (neutral)	e⁻ Conf	N-Rule
373.067416	1	C15H13ClF3N4O2	100.00	373.067364	-0.1	-1.1	33.6	13.0	even	ok

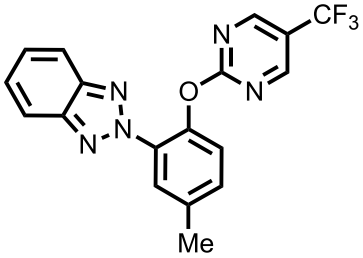

2-(5-Methyl-2-((5-(trifluoromethyl)pyrimidin-2-yl)oxy)phenyl)-2H-benzo[d][1,2,3]triazole (**7w**)
HRMS (ESI-FTICR) *m/z* = 372.1067 calcd for $C_{18}H_{13}F_3N_5O^+$ [M+H]$^+$, found 372.1066.

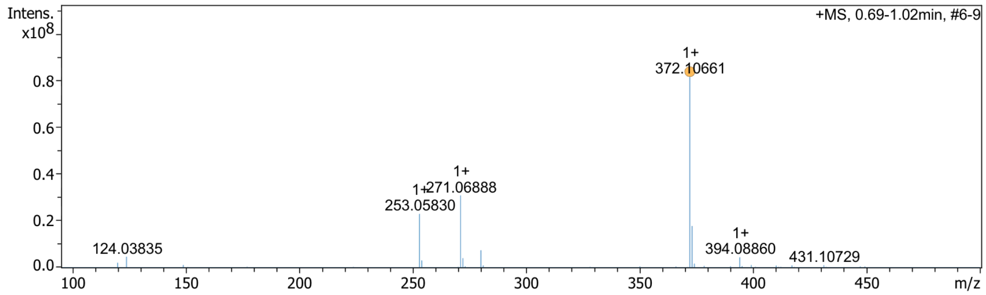

Meas. m/z	#	Ion Formula	Score	m/z	err [ppm]	Mean err [ppm]	mSigma	rdb (neutral)	e⁻ Conf	N-Rule
372.106606	1	C18H13F3N5O	100.00	372.106671	0.2	-0.1	14.7	14.0	even	ok

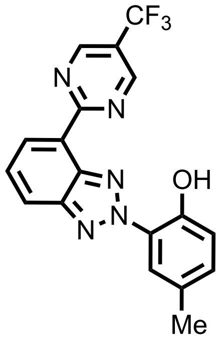

4-Methyl-2-(4-(5-(trifluoromethyl)pyrimidin-2-yl)-2H-benzo[d][1,2,3]triazol-2-yl)phenol (**7w'**)
HRMS (ESI-FTICR) *m/z* = 372.1067 calcd for $C_{18}H_{13}F_3N_5O^+$ [M+H]$^+$, found 372.1074.

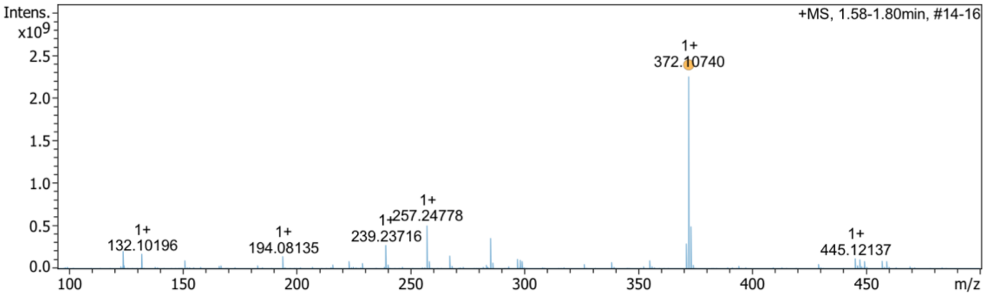

Meas. m/z	#	Ion Formula	Score	m/z	err [ppm]	Mean err [ppm]	mSigma	rdb (neutral)	e⁻ Conf	N-Rule
372.107397	1	C18H13F3N5O	100.00	372.106671	-2.0	-2.8	8.8	14.0	even	ok

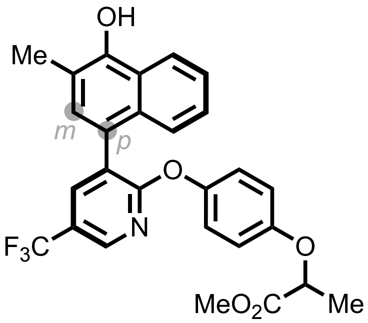

Methyl 2-(4-((3-(4-hydroxy-3-methylnaphthalen-1-yl)-5-(trifluoromethyl)pyridin-2-yl)oxy)phenoxy)propanoate (**7x**)
HRMS (ESI-FTICR) *m/z* = 498.1523 calcd for $C_{27}H_{23}F_3NO_5^+$ [M+H]$^+$, found 498.1528.

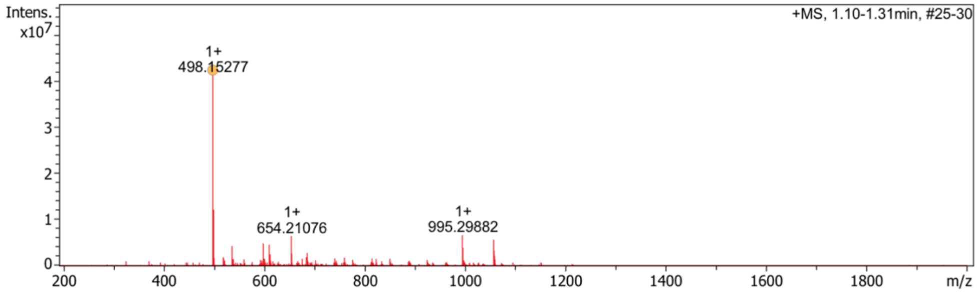

Meas. m/z	#	Ion Formula	Score	m/z	err [ppm]	|err| [mDa]	Mean err [ppm]	mSigma	rdb (neutral)	e⁻ Conf	N-Rule
498.152767	1	C27H23F3NO5	100.00	498.152284	-1.0	0.5	-1.2	8.2	16.0	even	ok

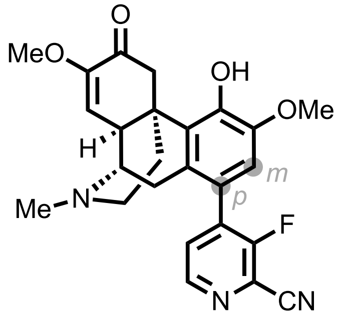

3-Fluoro-4-((4bR,8aS,9S)-4-hydroxy-3,7-dimethoxy-11-methyl-6-oxo-6,8a,9,10-tetrahydro-5H-9,4b-(epiminoethano)phenanthren-1-yl)picolinonitrile (**7y**)

HRMS (ESI-FTICR) *m/z* = 450.1824 calcd for $C_{25}H_{25}FN_3O_4^+$ [M+H]$^+$, found 450.1825.

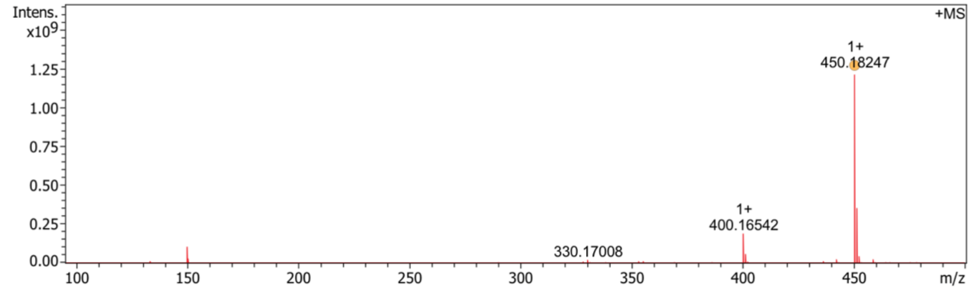

Meas. m/z	#	Ion Formula	Score	m/z	err [ppm]	|err| [mDa]	Mean err [ppm]	mSigma	rdb (neutral)	e⁻ Conf	N-Rule
450.182470	1	C25H25FN3O4	100.00	450.182361	-0.2	0.1	0.4	8.5	15.0	even	ok
450.182470	1	C26H24FN4NaO	100.00	450.182635	0.4	0.2	1.1	3.9	16.5	odd	ok

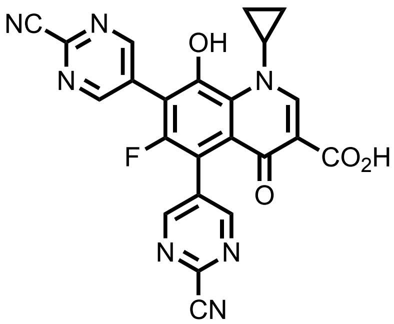

5,7-Bis(2-cyanopyrimidin-5-yl)-1-cyclopropyl-6-fluoro-8-hydroxy-4-oxo-1,4-dihydroquinoline-3-carboxylic acid (**7z**)

HRMS (ESI-FTICR) *m/z* = 468.0862 calcd for $C_{23}H_{11}FN_7O_4^-$ [M–H]$^-$, found 468.0861.

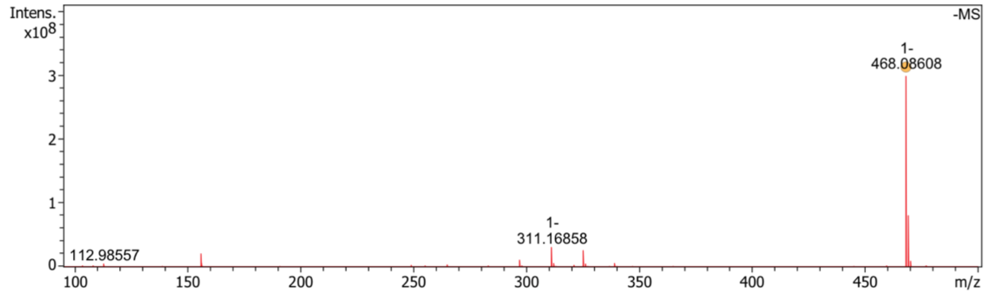

Meas. m/z	#	Ion Formula	Score	m/z	err [ppm]	|err| [mDa]	Mean err [ppm]	mSigma	rdb (neutral)	e⁻ Conf	N-Rule
468.086084	1	C23H11FN7O4	100.00	468.086204	0.3	0.1	0.8	9.1	21.0	even	ok
468.086084	2	C24H17FO9	69.34	468.086209	0.3	0.1	-0.3	13.1	15.5	odd	ok

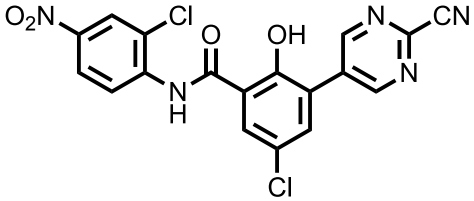

5-Chloro-N-(2-chloro-4-nitrophenyl)-3-(2-cyanopyrimidin-5-yl)-2-hydroxybenzamide (**7za**)
HRMS (ESI-FTICR) *m/z* = 427.99588 calcd for $C_{18}H_8Cl_2N_5O_4^-$ [M–H]⁻, found 427.99595.

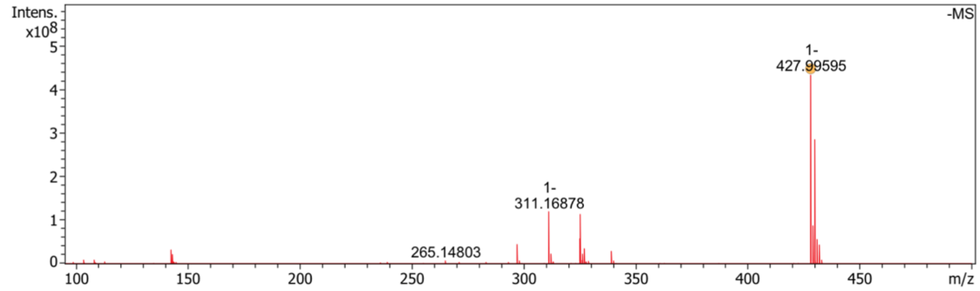

Meas. m/z	#	Ion Formula	Score	m/z	err [ppm]	|err| [mDa]	Mean err [ppm]	mSigma	rdb	e⁻ Conf	N-Rule
427.995953	1	C18H8Cl2N5O4	100.00	427.995883	-0.2	0.1	-0.8	6.2	22.0	even	ok

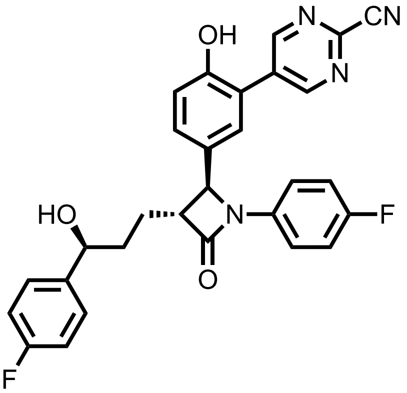

5-(5-((2S,3R)-1-(4-Fluorophenyl)-3-((S)-3-(4-fluorophenyl)-3-hydroxypropyl)-4-oxoazetidin-2-yl)-2-hydroxyphenyl)pyrimidine-2-carbonitrile (**7zb**)
HRMS (ESI-FTICR) *m/z* = 513.17327 calcd for $C_{29}H_{23}F_2N_4O_3^+$ [M+H]⁺, found 513.17326.

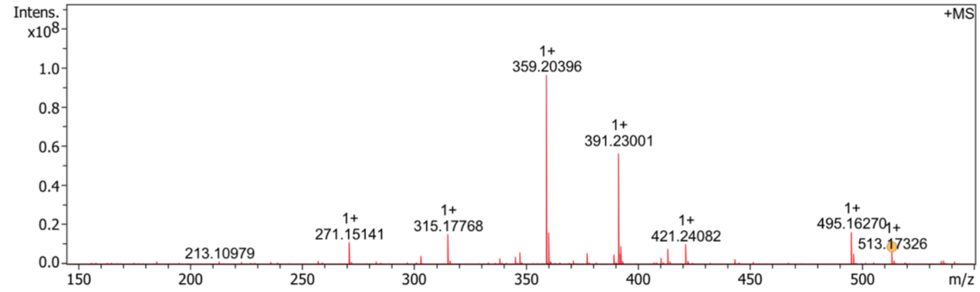

Meas. m/z	#	Ion Formula	Score	m/z	err [ppm]	|err| [mDa]	Mean err [ppm]	mSigma	rdb (neutral)	e⁻ Conf	N-Rule
513.173260	1	C29H23F2N4O3	100.00	513.173273	0.0	0.0	-0.2	9.6	20.0	even	ok

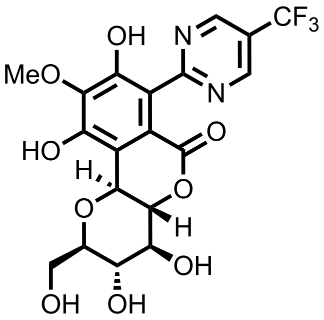

(2R,3S,4S,4aR,10bS)-3,4,8,10-Tetrahydroxy-2-(hydroxymethyl)-9-methoxy-7-(5-(trifluoromethyl)pyrimidin-2-yl)-3,4,4a,10b-tetrahydropyrano[3,2-c]isochromen-6(2H)-one (**7zc**)

HRMS (ESI-FTICR) *m/z* = 475.0959 calcd for $C_{19}H_{18}F_3N_2O_9^+$ [M+H]$^+$, found 475.0963.

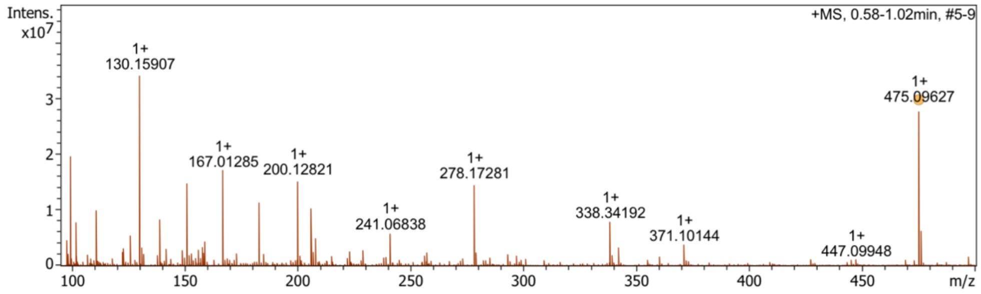

Meas. m/z	#	Ion Formula	Score	m/z	err [ppm]	Mean err [ppm]	mSigma	rdb (neutral)	e⁻ Conf	N-Rule
475.096273	1	C19H18F3N2O9	100.00	475.095891	-0.8	-1.3	12.3	11.0	even	ok

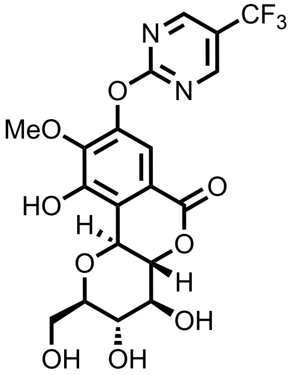

(2R,3S,4S,4aR,10bS)-3,4,10-Trihydroxy-2-(hydroxymethyl)-9-methoxy-8-((5-(trifluoromethyl)pyrimidin-2-yl)oxy)-3,4,4a,10b-tetrahydropyrano[3,2-c]isochromen-6(2H)-one (**7zc'**)

HRMS (ESI-FTICR) *m/z* = 475.09589 calcd for $C_{19}H_{18}F_3N_2O_9^+$ [M+H]$^+$, found 475.09592.

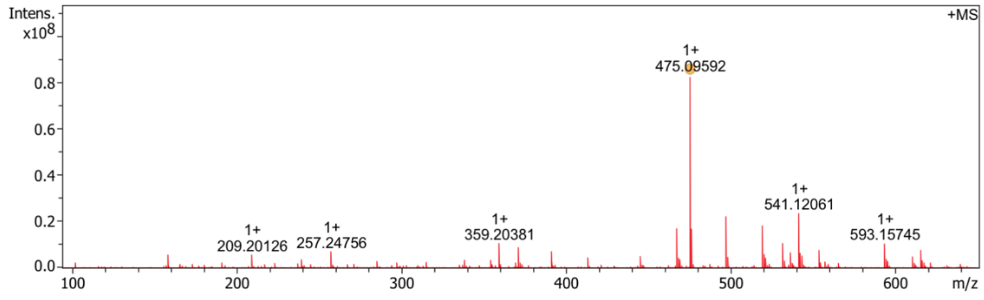

Meas. m/z	#	Ion Formula	Score	m/z	err [ppm]	|err| [mDa]	Mean err [ppm]	mSigma	rdb (neutral)	e⁻ Conf	N-Rule
475.095918	1	C19H18F3N2O9	100.00	475.095891	-0.1	0.0	-0.6	15.3	11.0	even	ok

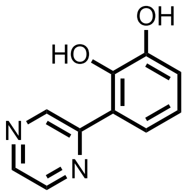

***3-(Pyrazin-2-yl)benzene-1,2-diol** (**o-Periplanpyrazine A**)* (**7zd**)

HRMS (ESI-FTICR) *m/z* = 189.0659 calcd for $C_{10}H_9N_2O_2^+$ [M+H]$^+$, found 189.0656.

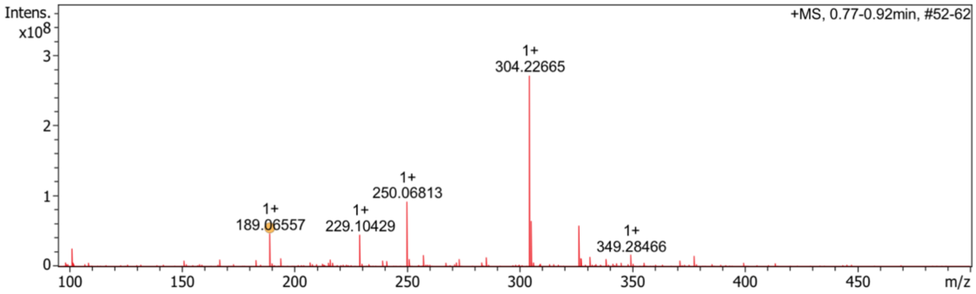

Meas. m/z	#	Ion Formula	Score	m/z	err [ppm]	Mean err [ppm]	mSigma	rdb (neutral)	e⁻ Conf	N-Rule
189.065569	1	C10H9N2O2	100.00	189.065854	1.5	1.2	7.5	8.0	even	ok

Note: m/z 250 and 304 are contaminants in the HRMS.

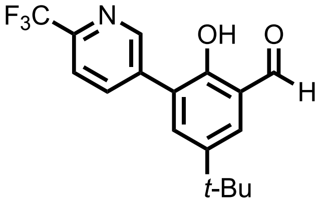

5-(Tert-butyl)-2-hydroxy-3-(6-(trifluoromethyl)pyridin-3-yl)benzaldehyde (**7ze**)

HRMS (ESI-FTICR) *m/z* = 324.1206 calcd for $C_{17}H_{17}F_3NO_2^+$ [M+H]$^+$, found 324.1201.

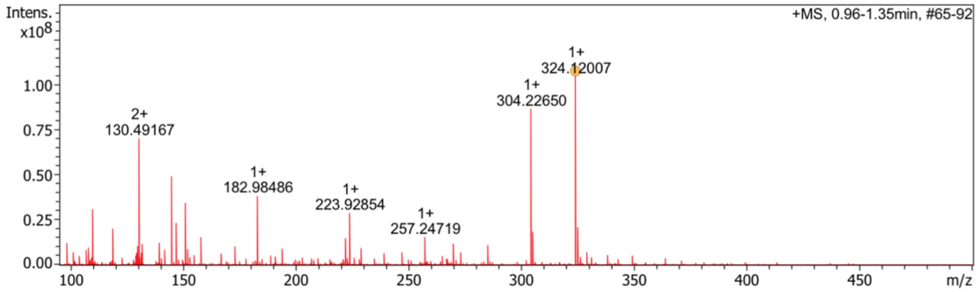

Meas. m/z	#	Ion Formula	Score	m/z	err [ppm]	Mean err [ppm]	mSigma	rdb (neutral)	e⁻ Conf	N-Rule
324.120070	1	C17H17F3NO2	100.00	324.120590	1.6	1.1	9.1	9.0	even	ok

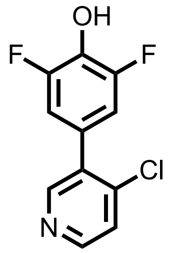

4-(4-Chloropyridin-3-yl)-2,6-difluorophenol (**7zg**)
HRMS (ESI-FTICR) *m/z* = 242.0179 calcd for $C_{11}H_7ClF_2NO^+$ [M+H]$^+$, found 242.0175.

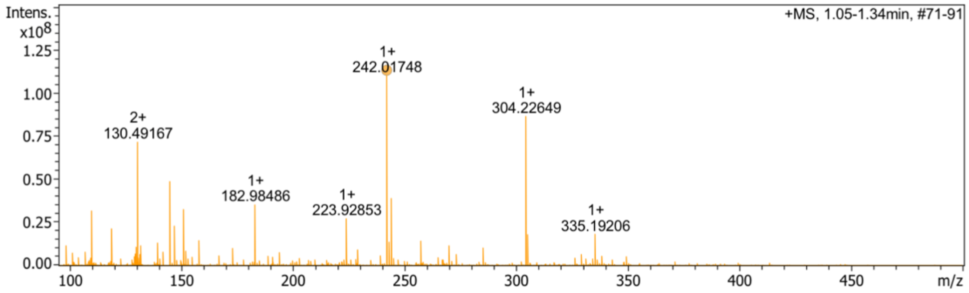

Meas. m/z	#	Ion Formula	Score	m/z	err [ppm]	Mean err [ppm]	mSigma	rdb (neutral)	e⁻ Conf	N-Rule
242.017481	1	C11H7ClF2NO	100.00	242.017874	1.6	1.4	15.0	11.0	even	ok

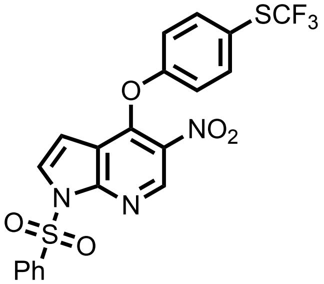

5-Nitro-1-(phenylsulfonyl)-4-(4-((trifluoromethyl)thio)phenoxy)-1H-pyrrolo[2,3-b]pyridine (**7zh**)
HRMS (ESI-FTICR) *m/z* = 496.0243 calcd for $C_{20}H_{13}F_3N_3O_5S_2^+$ [M+H]$^+$, found 496.0237.

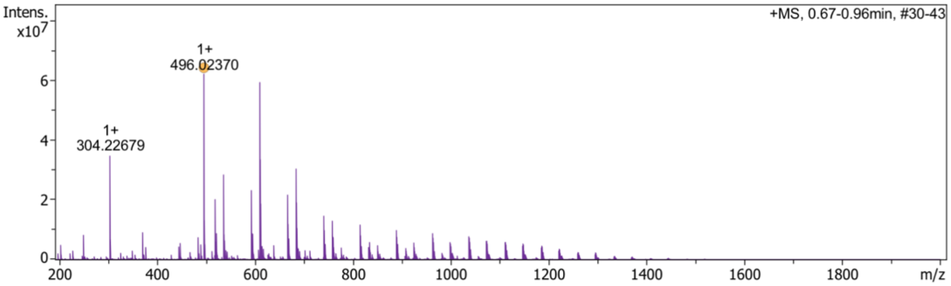

Meas. m/z	#	Ion Formula	Score	m/z	err [ppm]	|err| [mDa]	Mean err [ppm]	mSigma	rdb (neutral)	e⁻ Conf	N-Rule
496.023696	1	C20H13F3N3O5S2	96.99	496.024324	1.3	0.6	1.8	12.8	19.0	even	ok

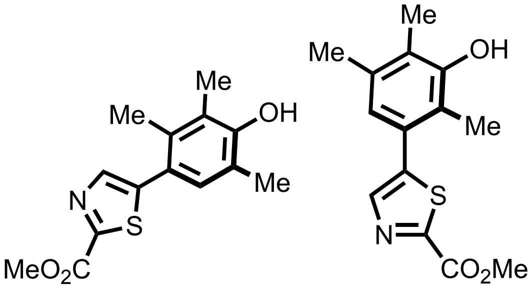

Methyl 5-(4-hydroxy-2,3,5-trimethylphenyl)thiazole-2-carboxylate (**7zi**)
Methyl 5-(3-hydroxy-2,4,5-trimethylphenyl)thiazole-2-carboxylate (**7zi'**)
HRMS (ESI-FTICR) *m/z* = 278.0845 calcd for $C_{14}H_{16}NO_3S^+$ $[M+H]^+$, found 278.0851.

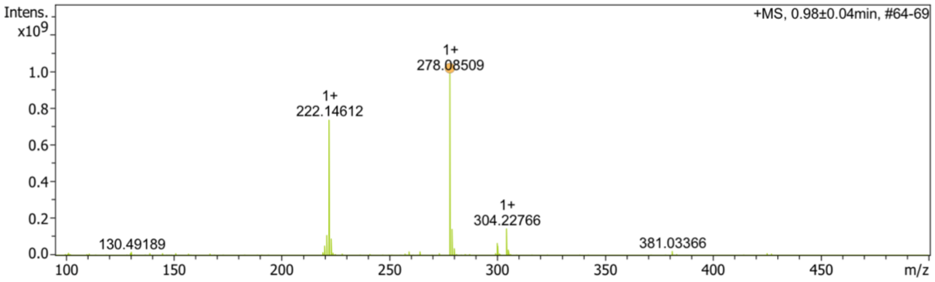

Meas. m/z	#	Ion Formula	Score	m/z	err [ppm]	|err| [mDa]	Mean err [ppm]	mSigma	rdb (neutral)	e⁻ Conf	N-Rule
278.085093	1	C14H16NO3S	100.00	278.084541	-2.0	0.6	-1.4	6.9	10.0	even	ok

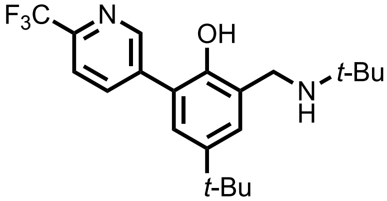

4-(Tert-butyl)-2-((tert-butylamino)methyl)-6-(6-(trifluoromethyl)pyridin-3-yl)phenol (JPC-2997) (**7zf**)
HRMS (ESI-FTICR): *m/z* = 381.2148 calcd for $C_{21}H_{28}F_3N_2O^+$ $[M+H]^+$, found 381.2141.

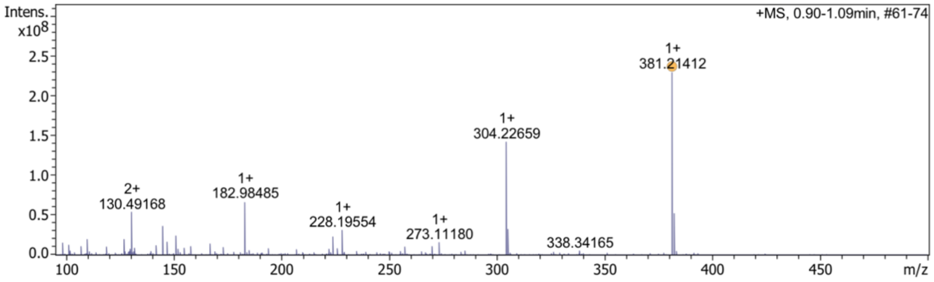

Meas. m/z	#	Ion Formula	Score	m/z	err [ppm]	Mean err [ppm]	mSigma	rdb (neutral)	e⁻ Conf	N-Rule
381.214119	1	C21H28F3N2O	100.00	381.214824	1.9	1.1	3.4	8.0	even	ok

18. NMR Spectral Data

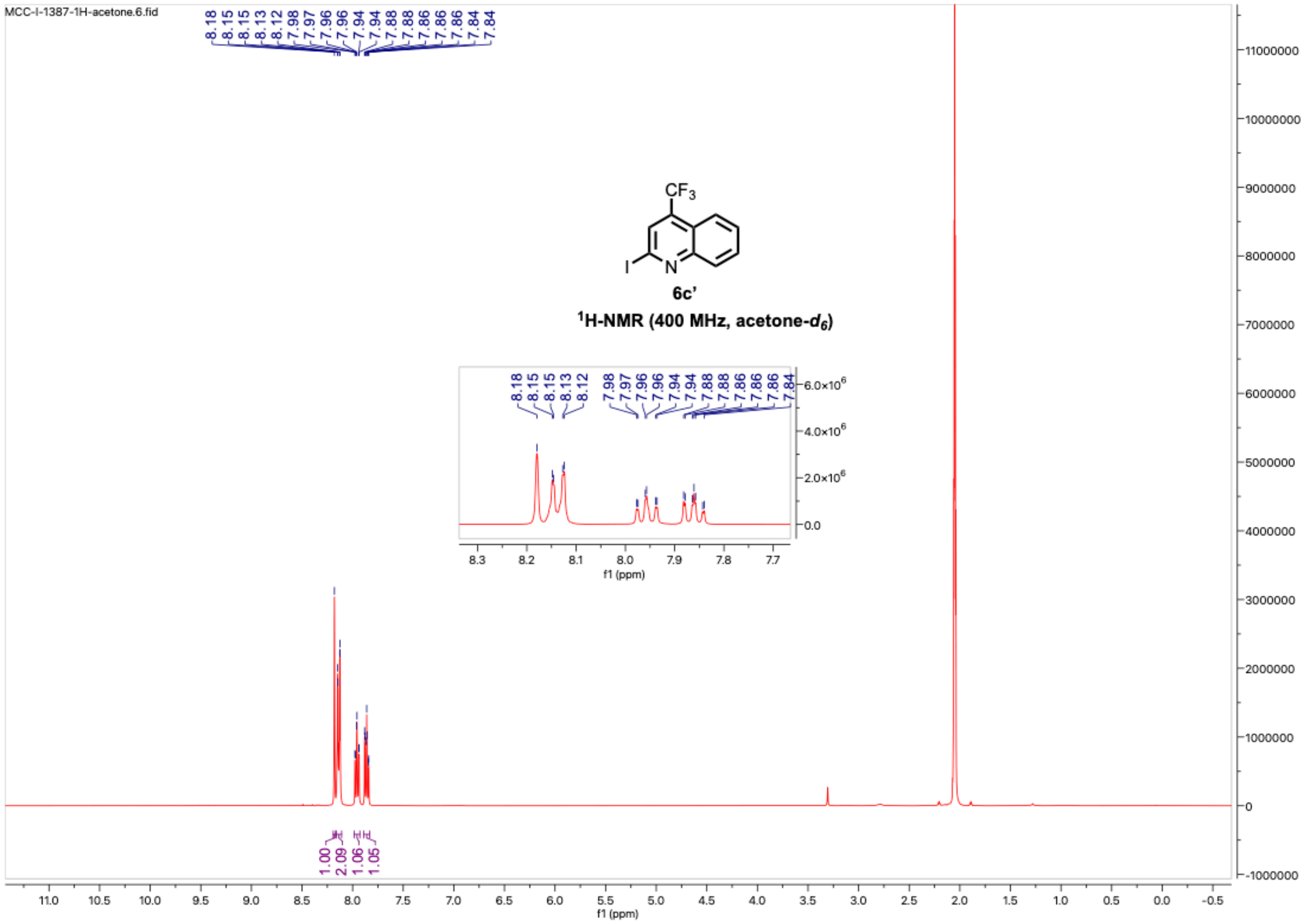

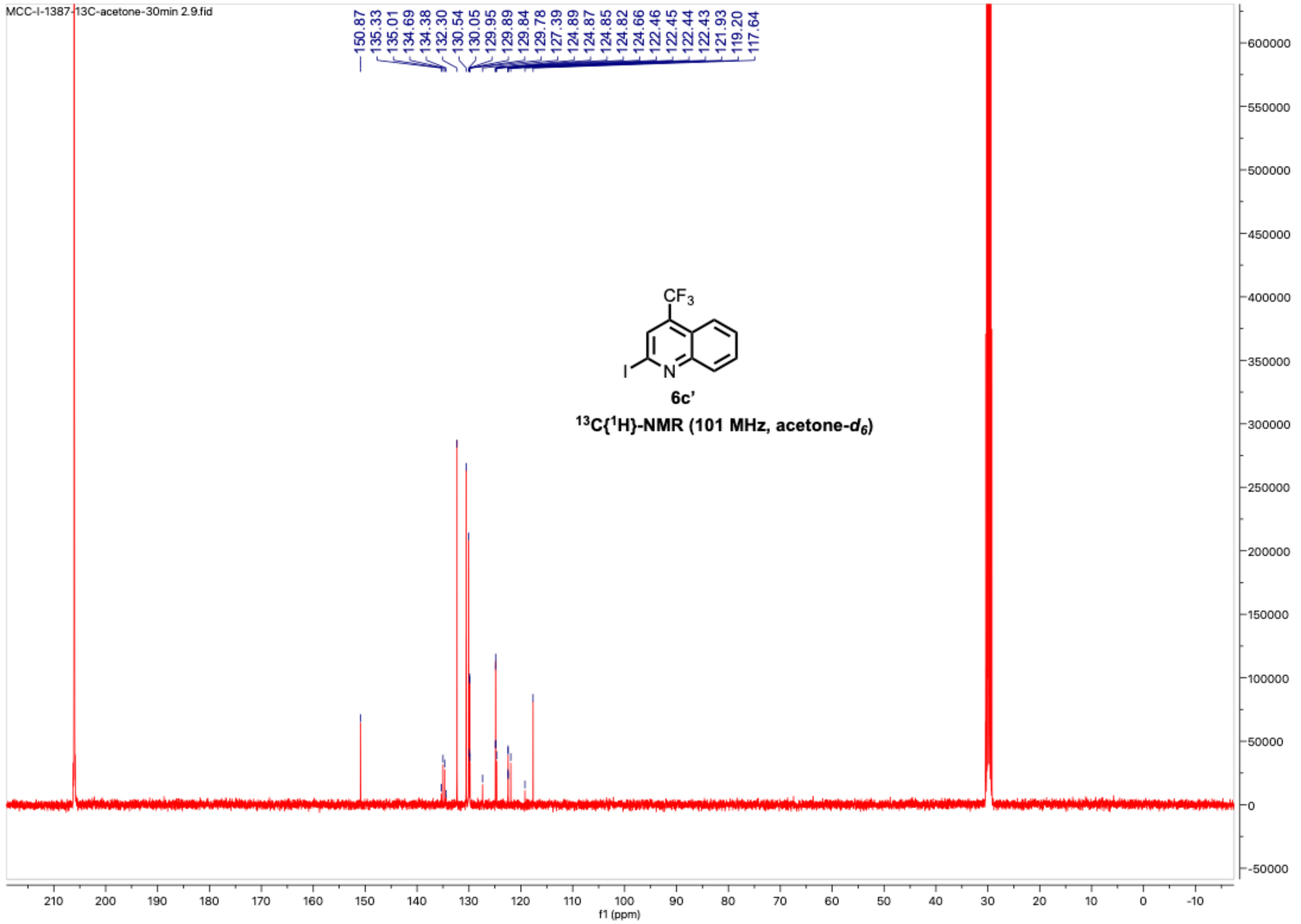

MCC-I-1387-13C-acetone-30min 2.9.fid
150.87
135.33
135.01
134.69
134.38
132.30
130.54
130.05
129.95
129.89
129.84
129.78
127.39
124.89
124.87
124.85
124.82
124.66
122.46
122.45
122.44
122.43
121.93
119.20
117.64
CF3
I
N
6c'
13C{1H}-NMR (101 MHz, acetone-d6)
f1 (ppm)

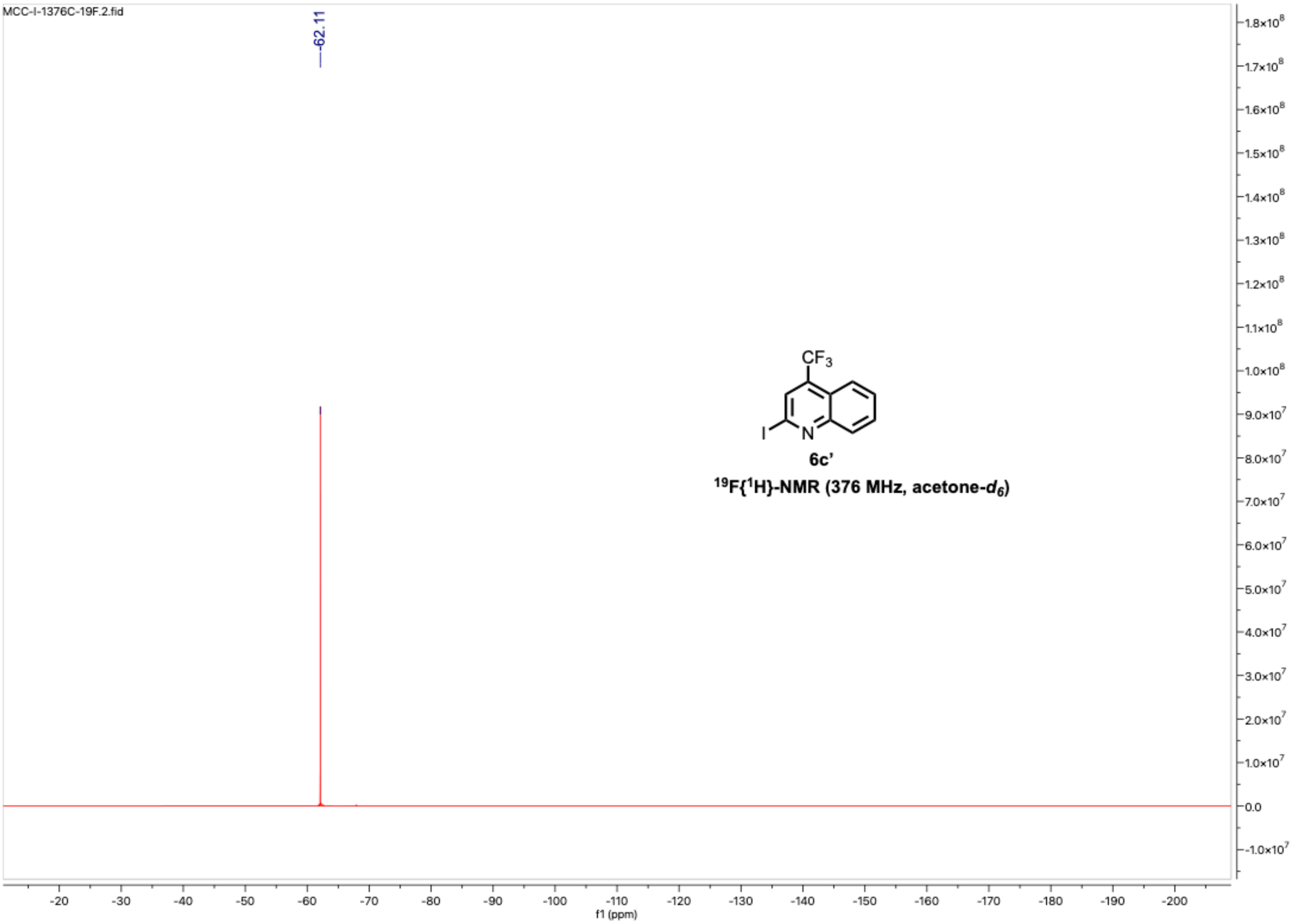

6c’

^{19}F{^{1}H}-NMR (376 MHz, acetone-d_6)

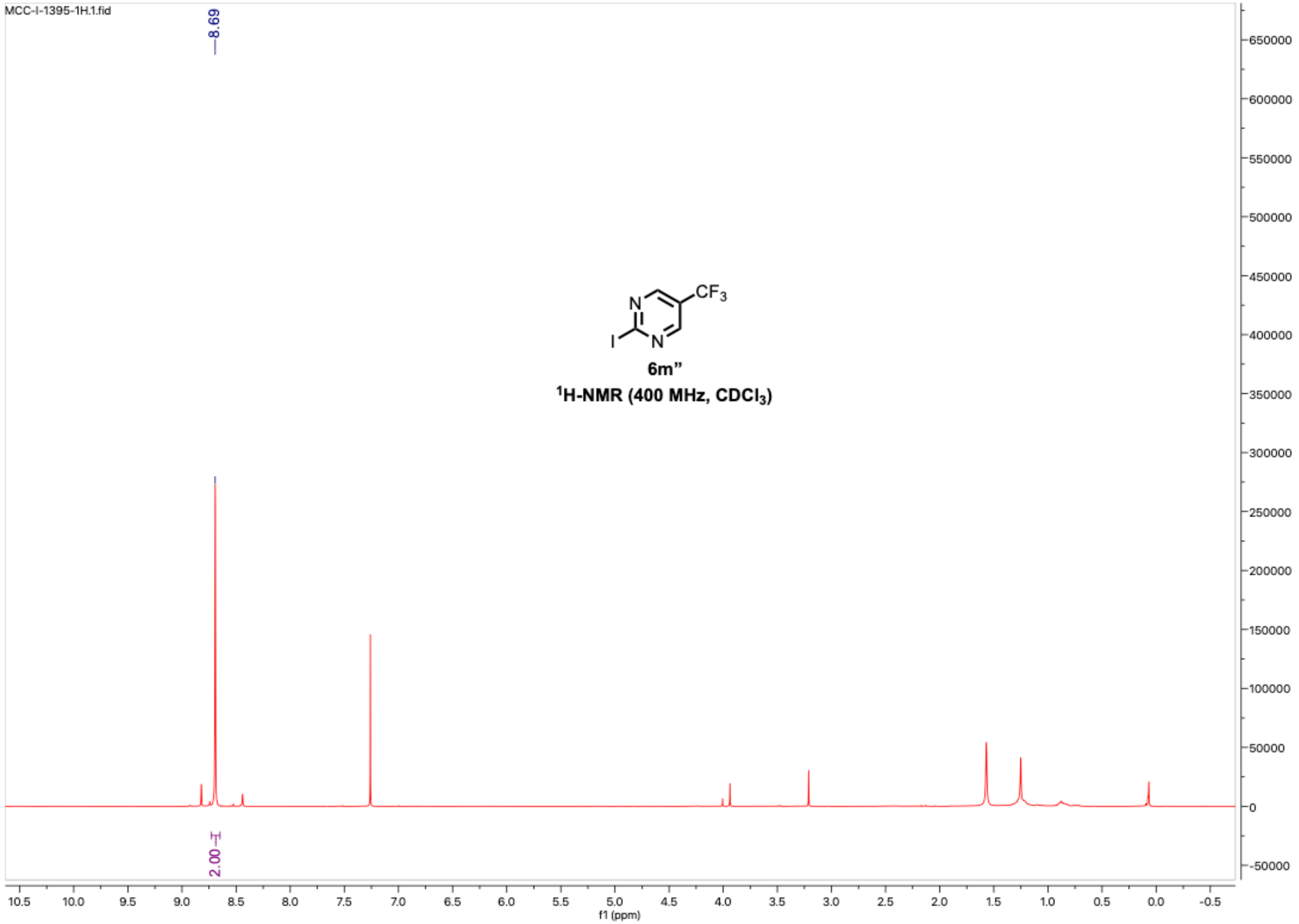

MCC-I-1395-1H.1.fid
8.69
6m"
1H-NMR (400 MHz, CDCl3)
2.00
f1 (ppm)

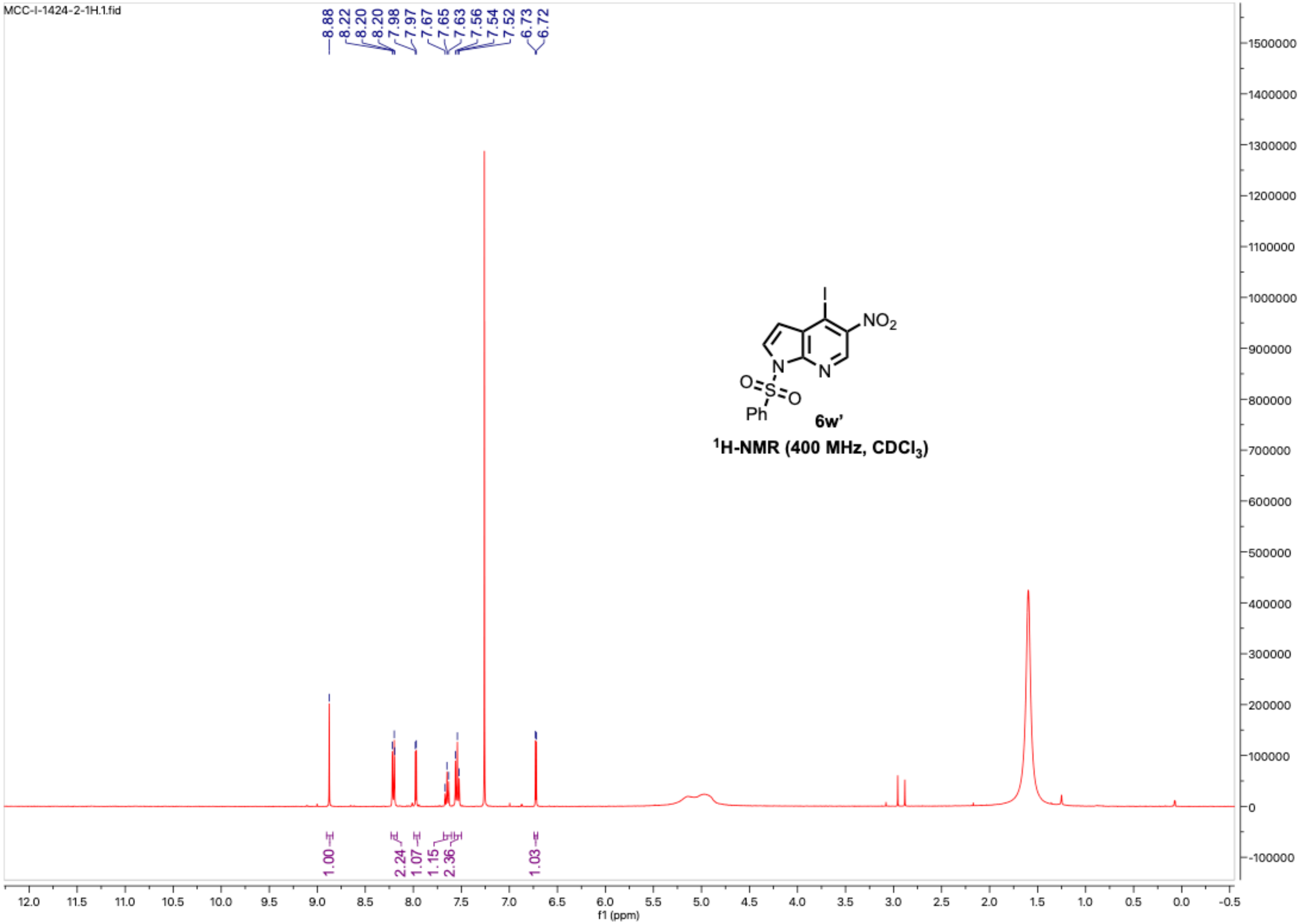

6w'

^{1}H-NMR (400 MHz, $CDCl_3$)

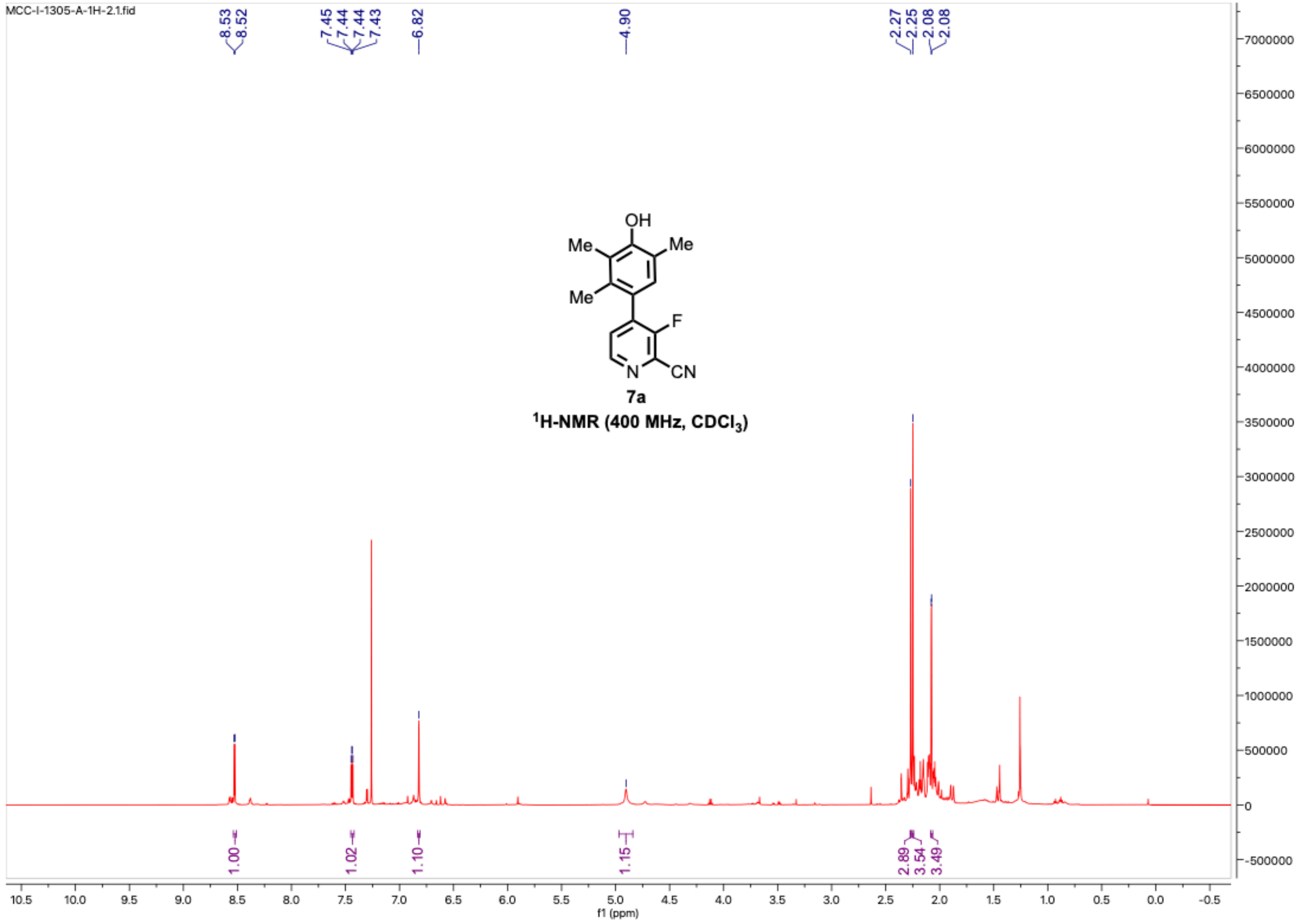
MCC-I-1305-A-1H-2.1.fid
8.53
8.52
7.45
7.44
7.44
7.43
6.82
4.90
2.27
2.25
2.08
2.08
OH
Me
Me
Me
F
N
CN
7a
1H-NMR (400 MHz, CDCl3)
1.00
1.02
1.10
1.15
2.89
3.54
3.49
f1 (ppm)

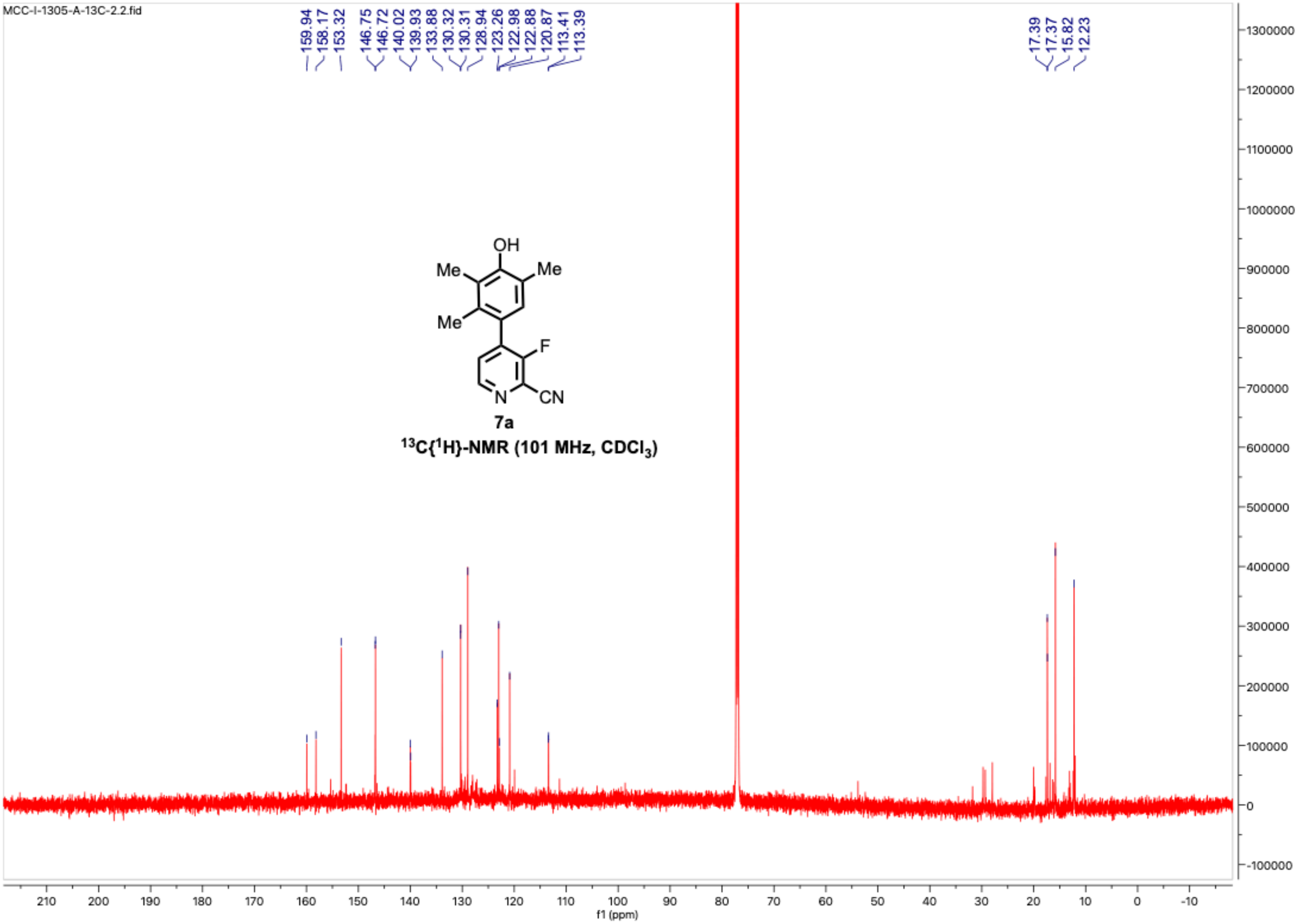
MCC-I-1305-A-13C-2.2.fid
159.94
158.17
153.32
146.75
146.72
140.02
139.93
133.88
130.32
130.31
128.94
123.26
122.98
122.88
120.87
113.41
113.39
17.39
17.37
15.82
12.23
OH
Me
Me
Me
F
N
CN
7a
13C{1H}-NMR (101 MHz, CDCl3)
f1 (ppm)

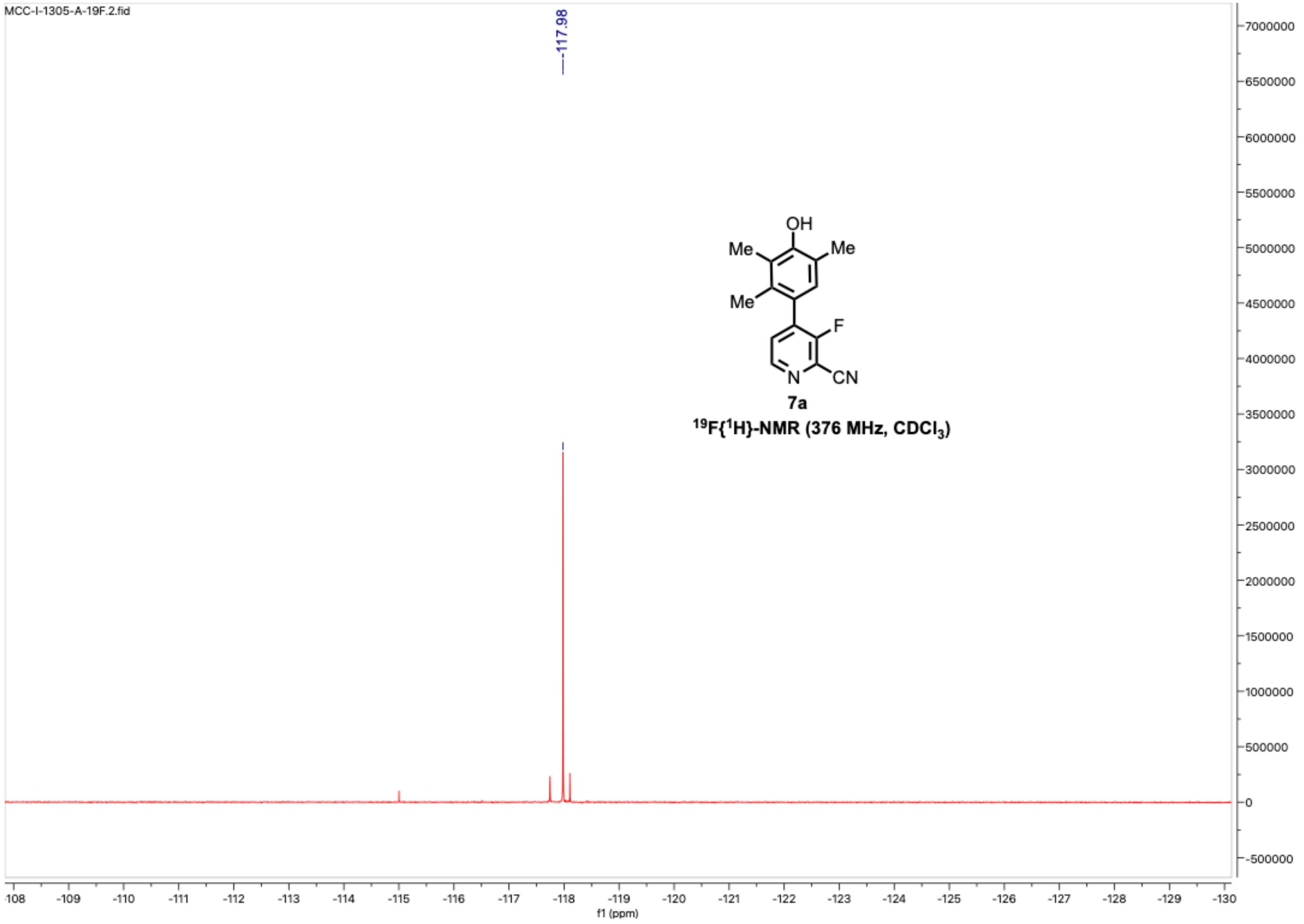
MCC-I-1305-A-19F.2.fid
-117.98
OH
Me
Me
Me
F
N
CN
7a
19F{1H}-NMR (376 MHz, CDCl3)
f1 (ppm)

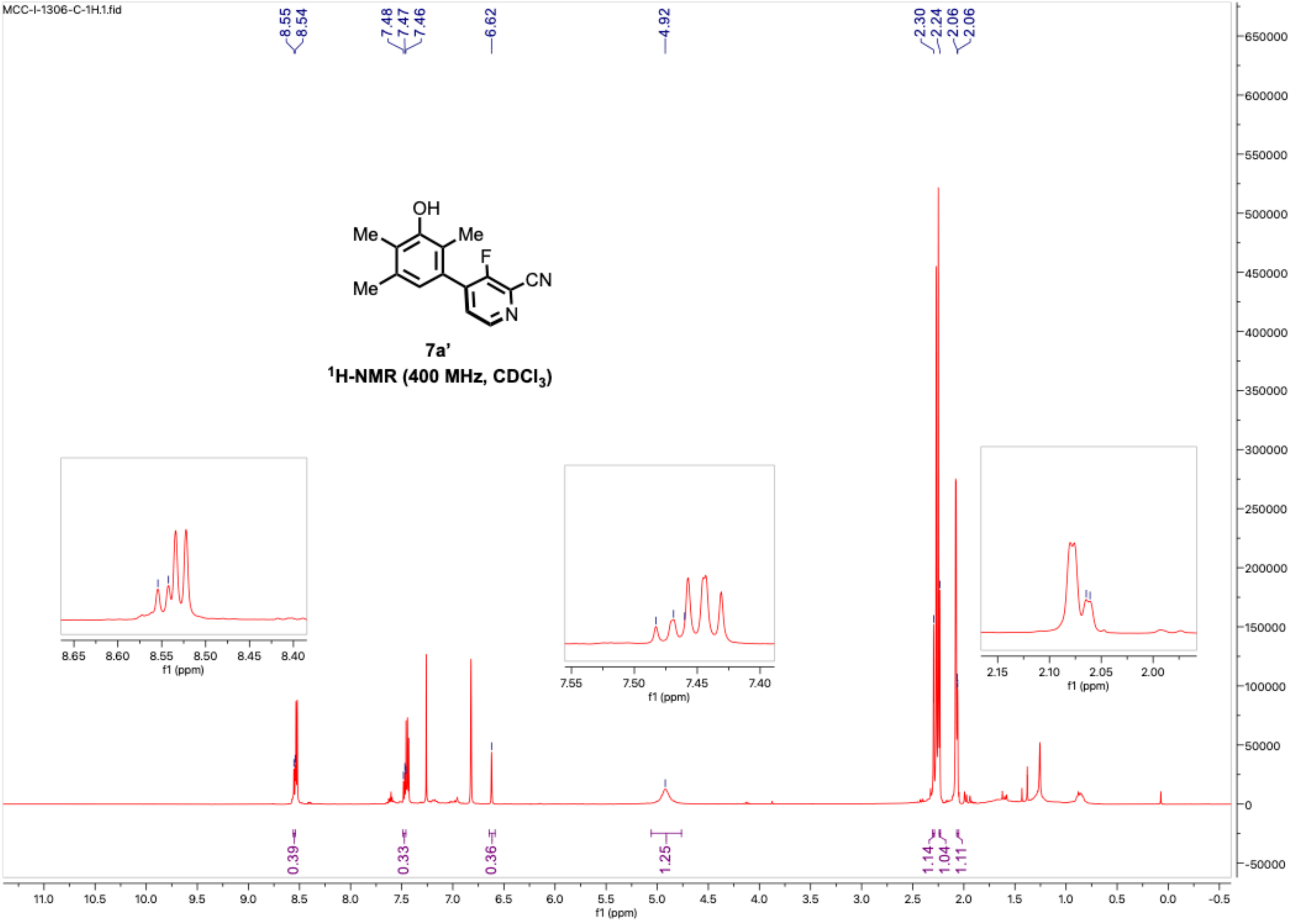

MCC-I-1306-C-1H.1.fid
8.55
8.54
7.48
7.47
7.46
6.62
4.92
2.30
2.24
2.06
2.06
OH
Me
Me
F
CN
Me
N
7a'
1H-NMR (400 MHz, CDCl3)
0.39
0.33
0.36
1.25
1.14
1.04
1.11
f1 (ppm)

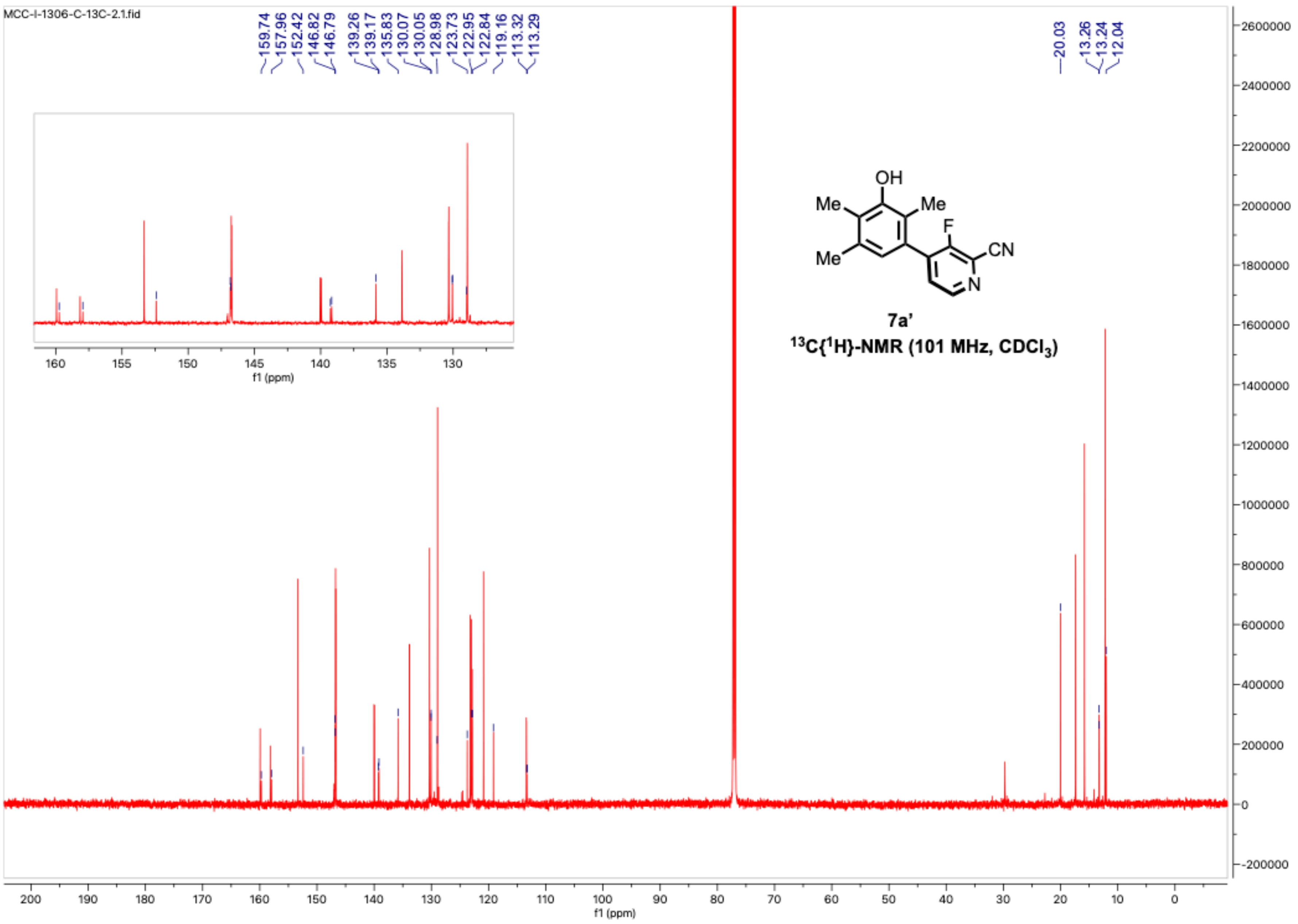

MCC-I-1306-C-13C-2.1.fid
159.74
157.96
152.42
146.82
146.79
139.26
139.17
135.83
130.07
130.05
128.98
123.73
122.95
122.84
119.16
113.32
113.29
20.03
13.26
13.24
12.04
OH
Me
Me
Me
F
CN
N
7a'
13C{1H}-NMR (101 MHz, CDCl3)
f1 (ppm)

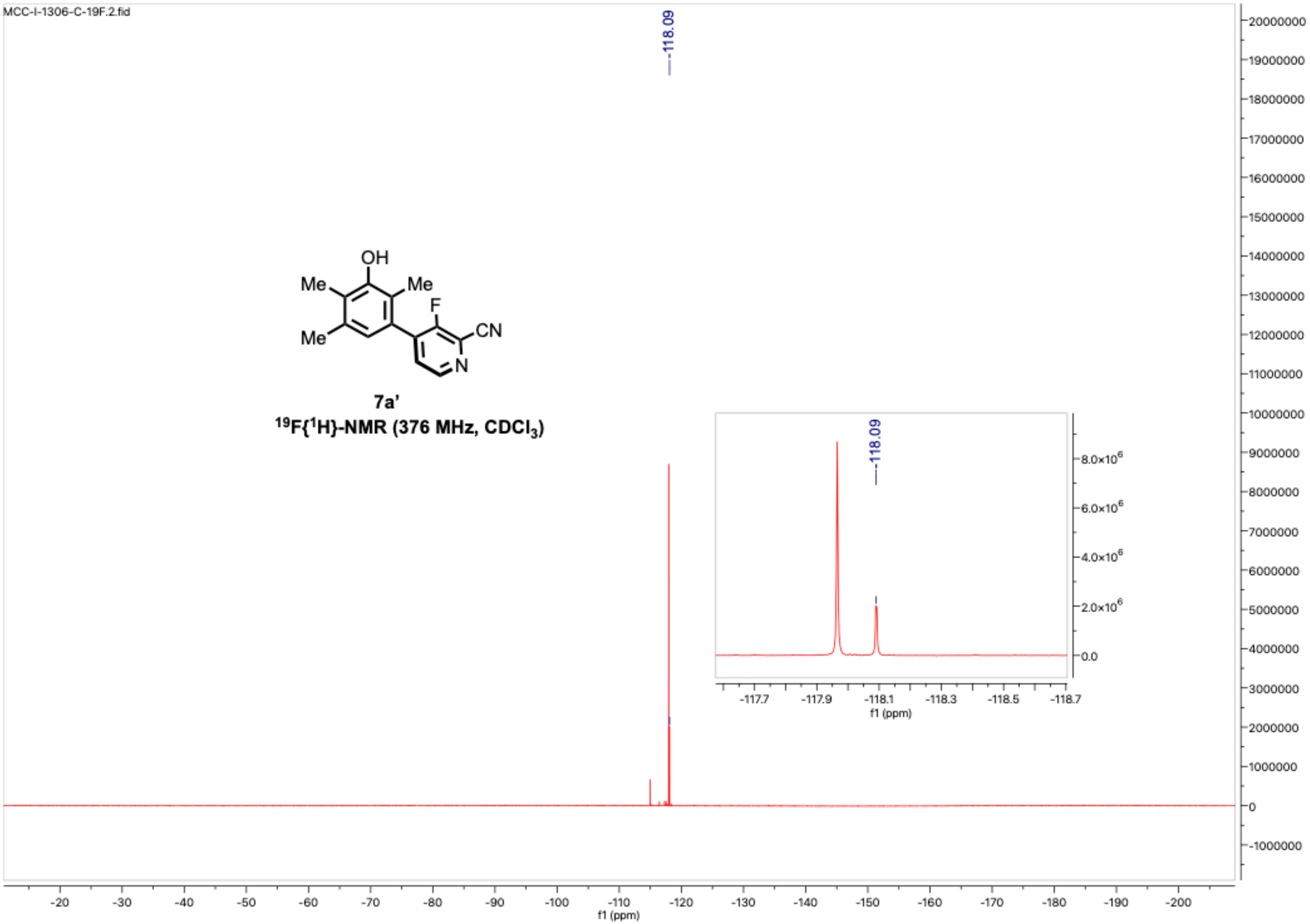

7a'

$^{19}F\{^{1}H\}$-NMR (376 MHz, $CDCl_3$)

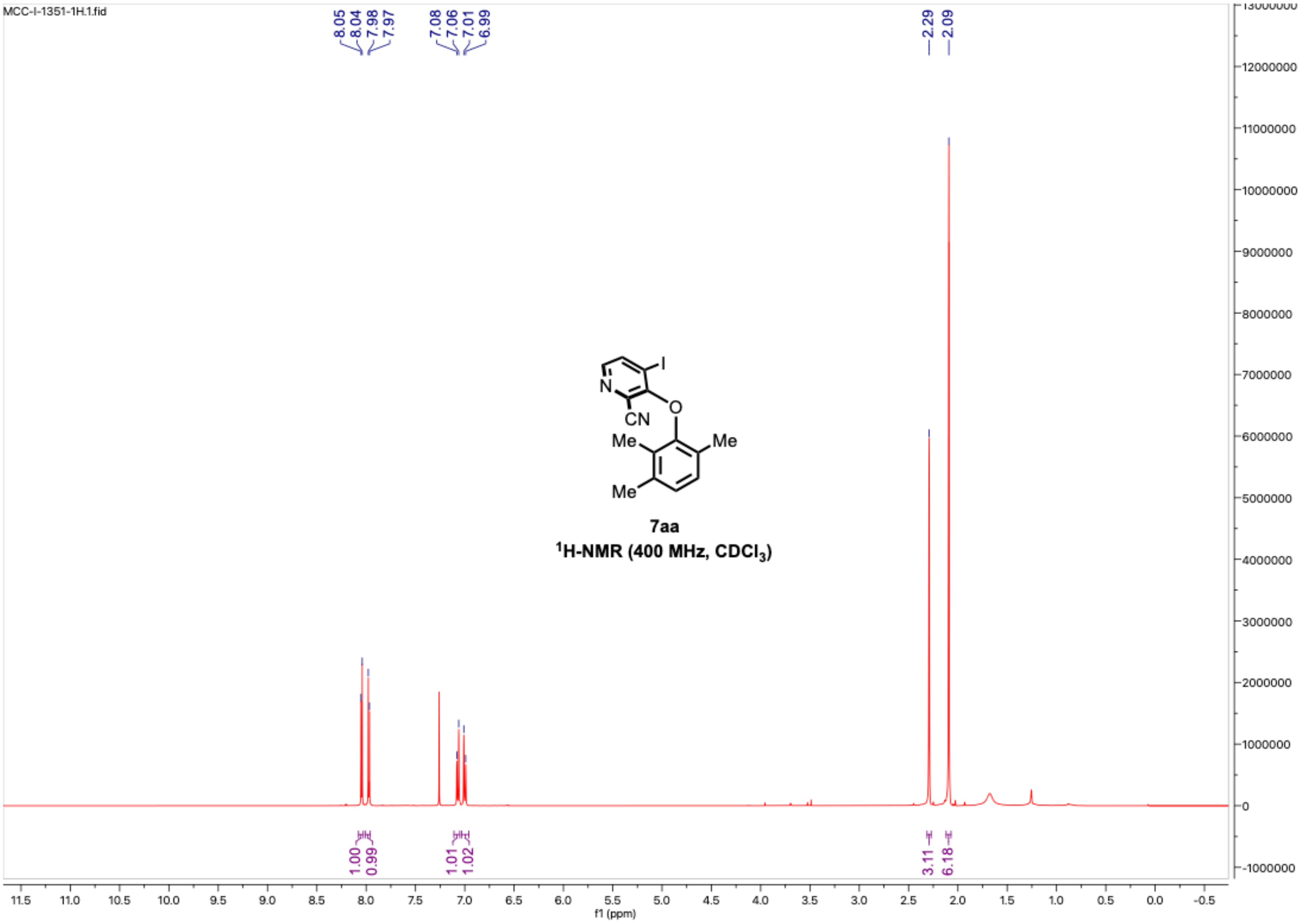
MCC-I-1351-1H.1.fid
8.05
8.04
7.98
7.97
7.08
7.06
7.01
6.99
2.29
2.09
I
N
O
CN
Me
Me
Me
7aa
1H-NMR (400 MHz, CDCl3)
1.00
0.99
1.01
1.02
3.11
6.18
f1 (ppm)

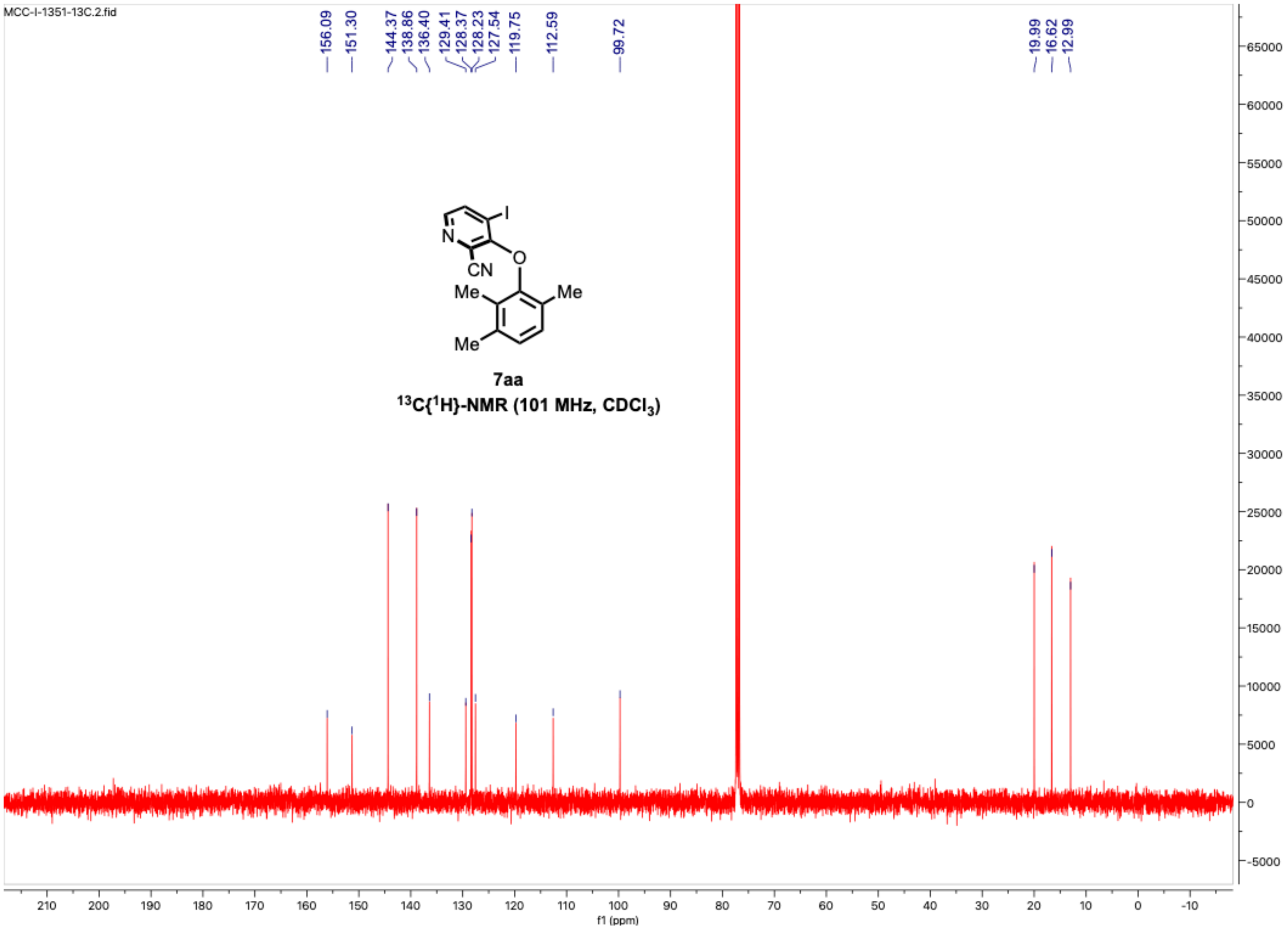
MCC-I-1351-13C.2.fid
156.09
151.30
144.37
138.86
136.40
129.41
128.37
128.23
127.54
119.75
112.59
99.72
19.99
16.62
12.99
7aa
13C{1H}-NMR (101 MHz, CDCl3)
f1 (ppm)

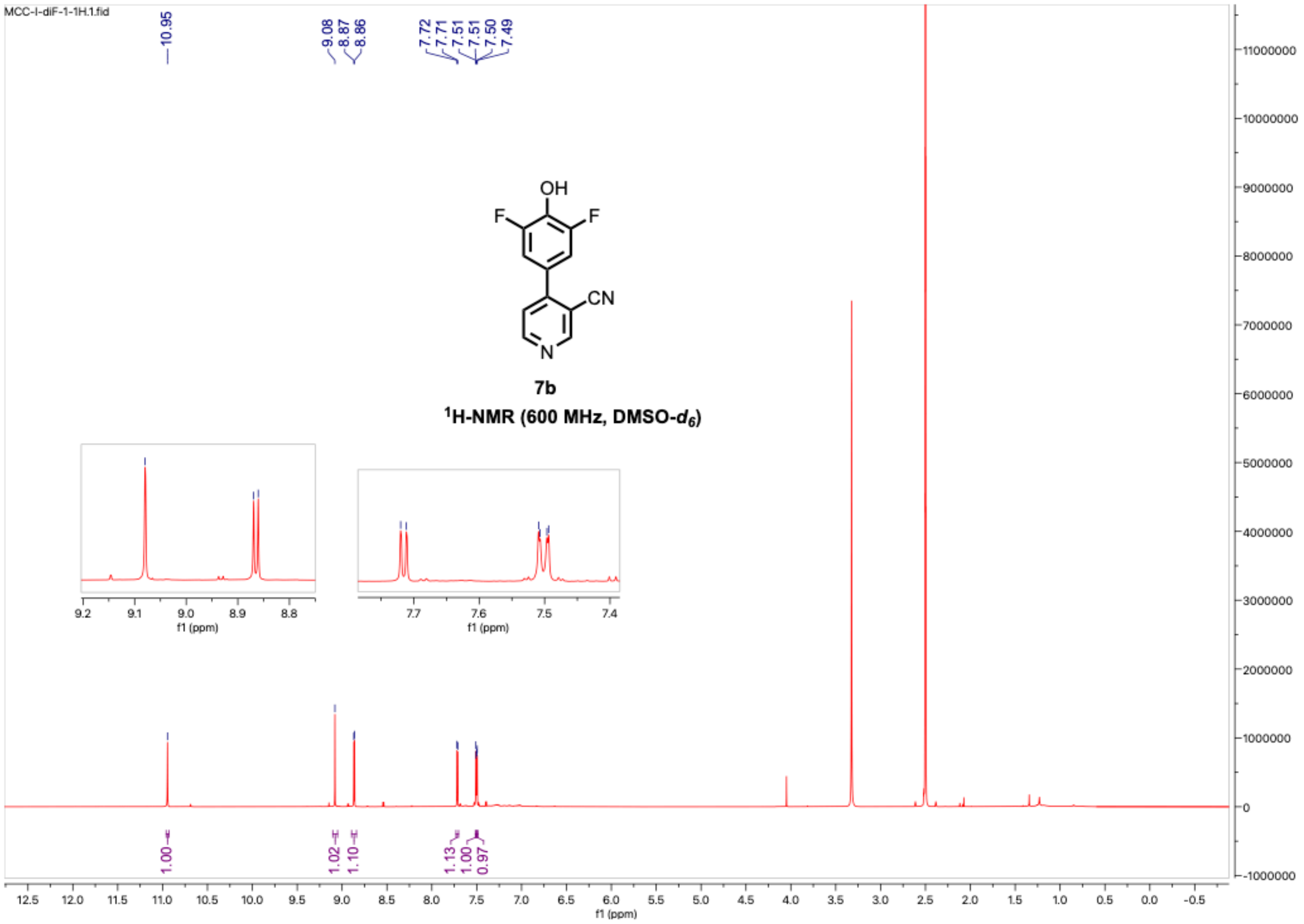

MCC-I-diF-1-1H.1.fid
10.95
9.08
8.87
8.86
7.72
7.71
7.51
7.51
7.50
7.49
OH
F
F
CN
N
7b
^{1}H-NMR (600 MHz, DMSO-d_6)
1.00
1.02
1.10
1.13
1.00
0.97
f1 (ppm)

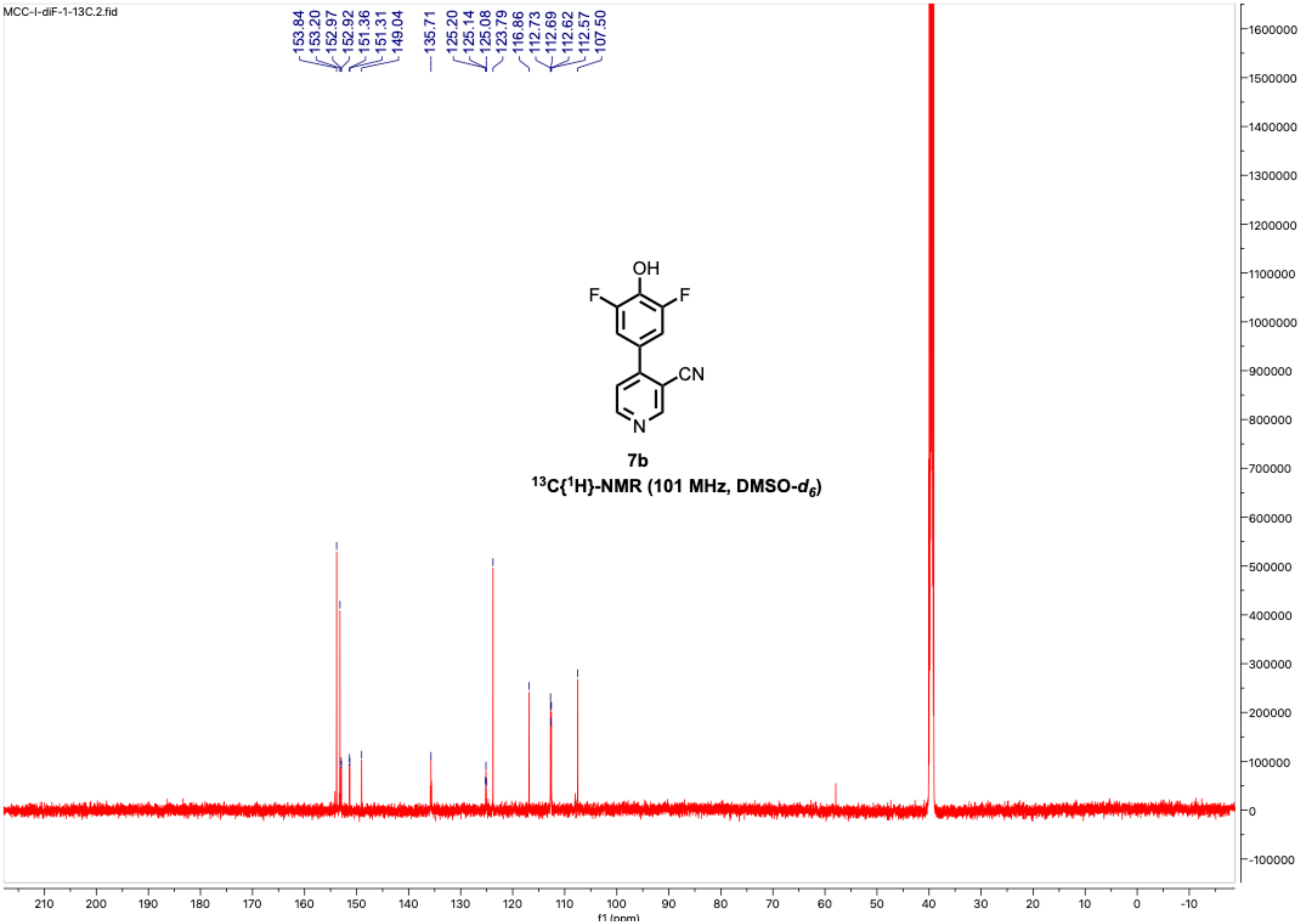
MCC-I-diF-1-13C.2.fid
153.84
153.20
152.97
152.92
151.36
151.31
149.04
135.71
125.20
125.14
125.08
123.79
116.86
112.73
112.69
112.62
112.57
107.50
OH
F
F
CN
N
7b
13C{1H}-NMR (101 MHz, DMSO-d6)
f1 (ppm)

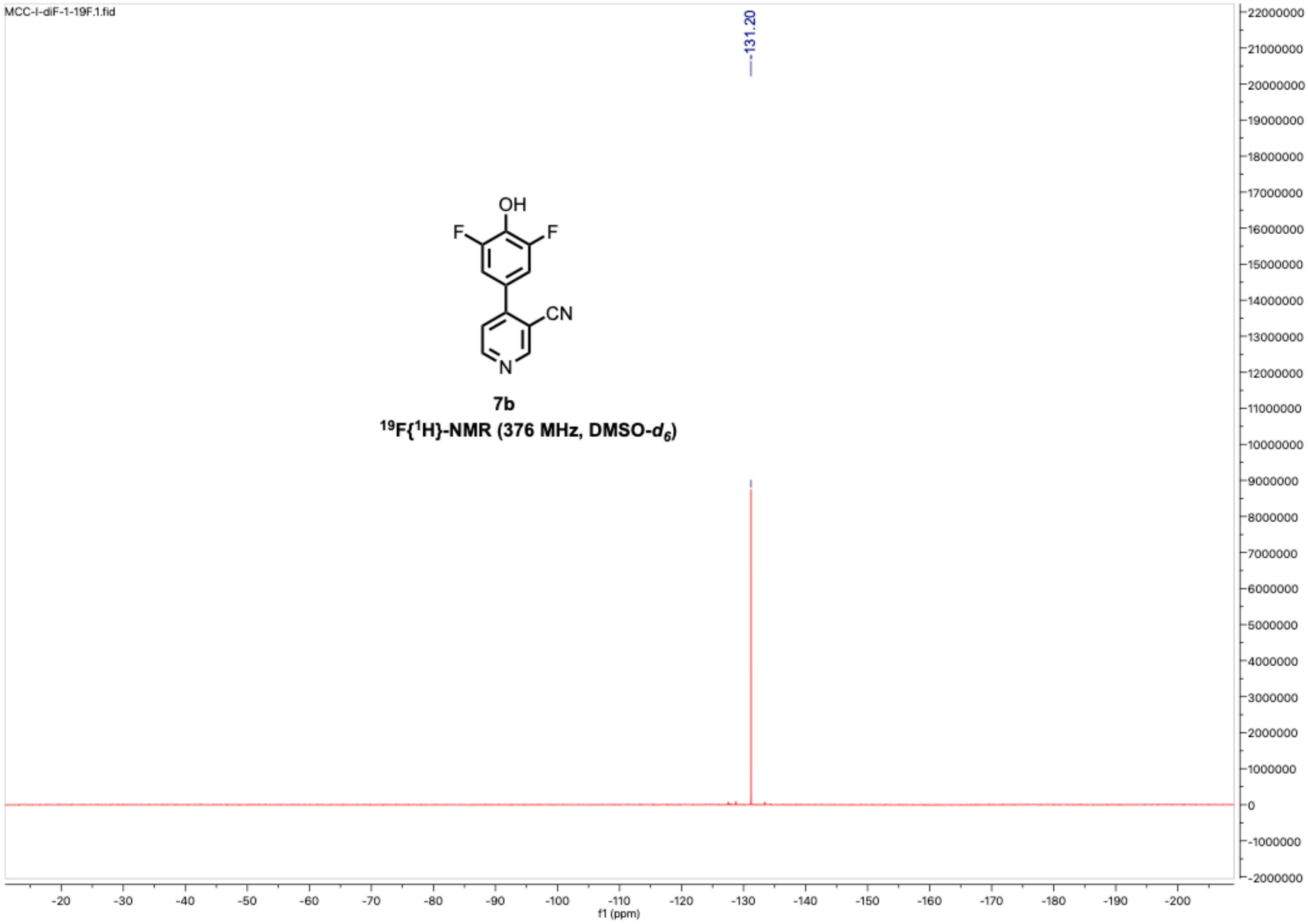

MCC-I-diF-1-19F.1.fid
-131.20
OH
F
F
CN
N
7b
19F{1H}-NMR (376 MHz, DMSO-d6)
f1 (ppm)

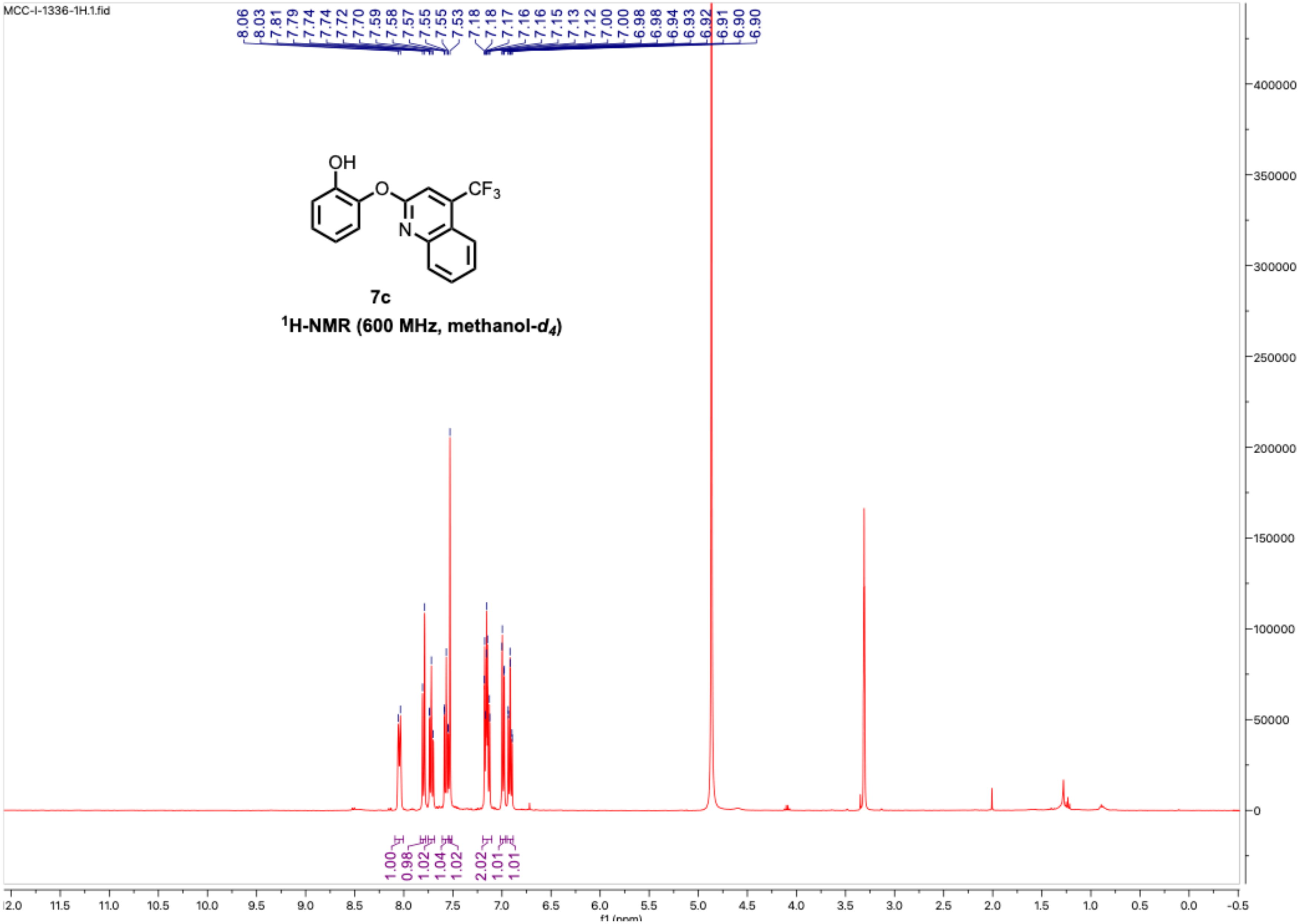
MCC-I-1336-1H.1.fid
7c
^{1}H-NMR (600 MHz, methanol-d_4)
f1 (ppm)

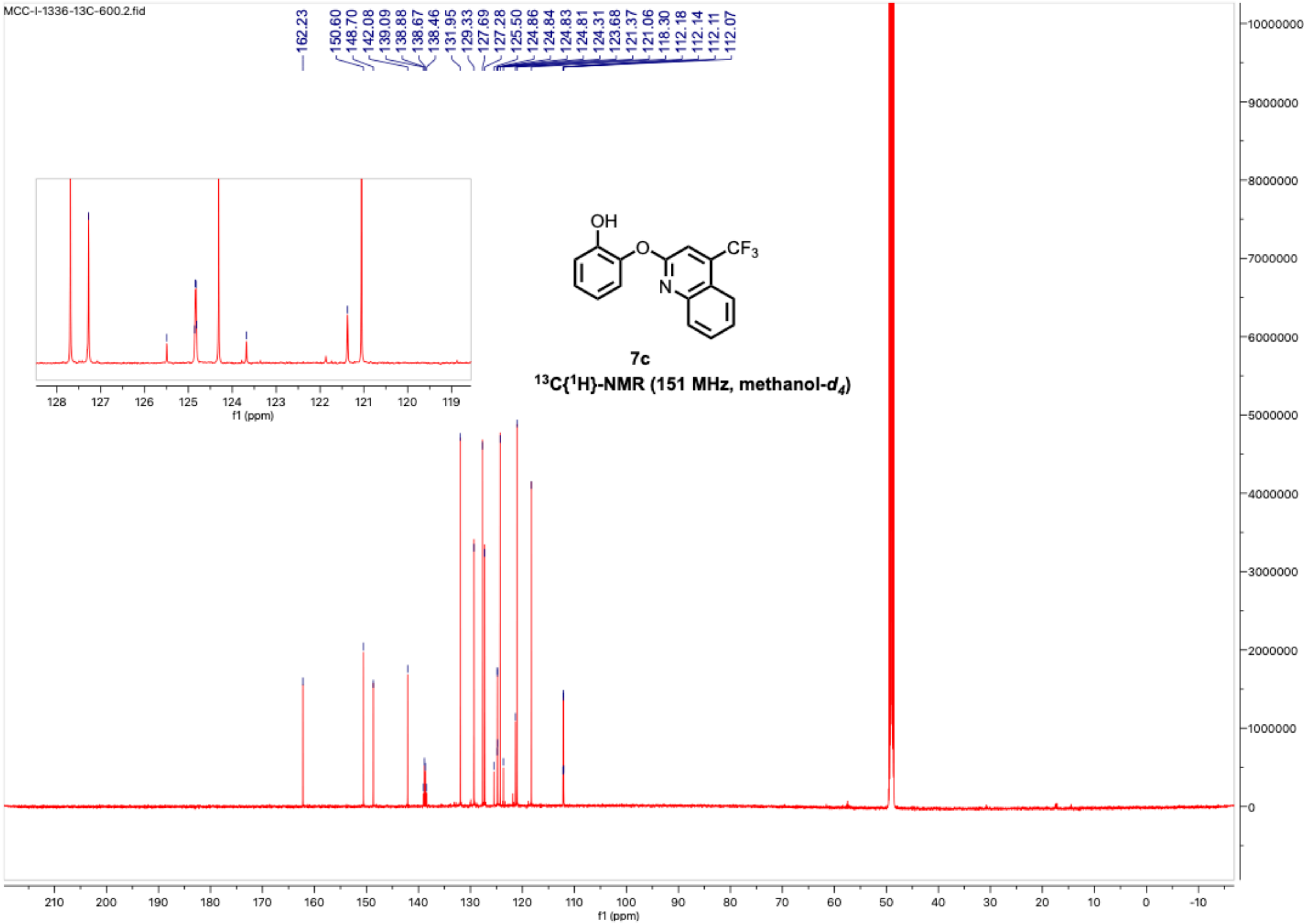
MCC-I-1336-13C-600.2.fid
162.23
150.60
148.70
142.08
139.09
138.88
138.67
138.46
131.95
129.33
127.69
127.28
125.50
124.86
124.84
124.83
124.81
124.31
123.68
121.37
121.06
118.30
112.18
112.14
112.11
112.07
OH
O
CF3
N
7c
13C{1H}-NMR (151 MHz, methanol-d4)
f1 (ppm)

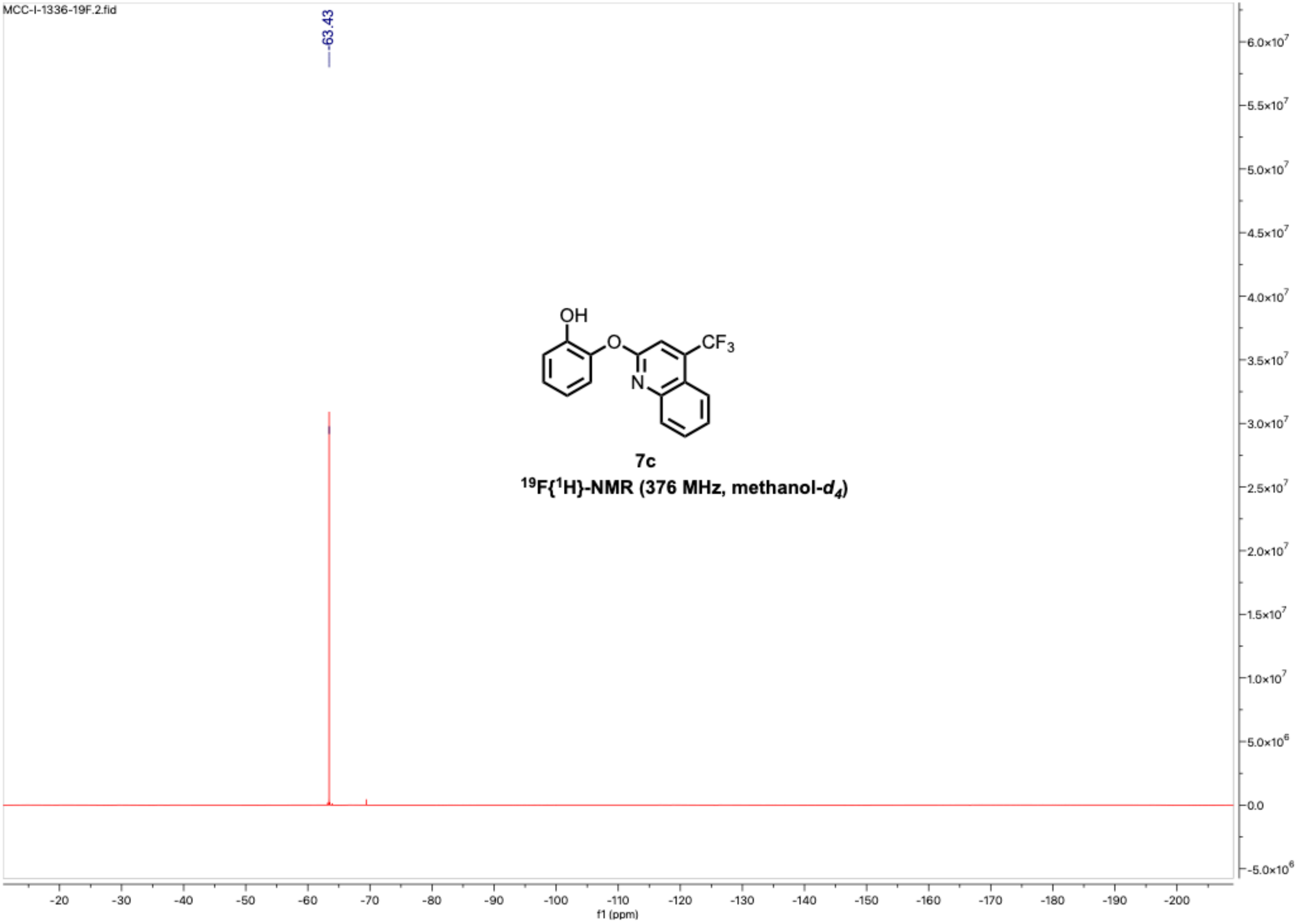
MCC-I-1336-19F.2.fid
-63.43
OH
O
N
CF3
7c
19F{1H}-NMR (376 MHz, methanol-d4)
f1 (ppm)

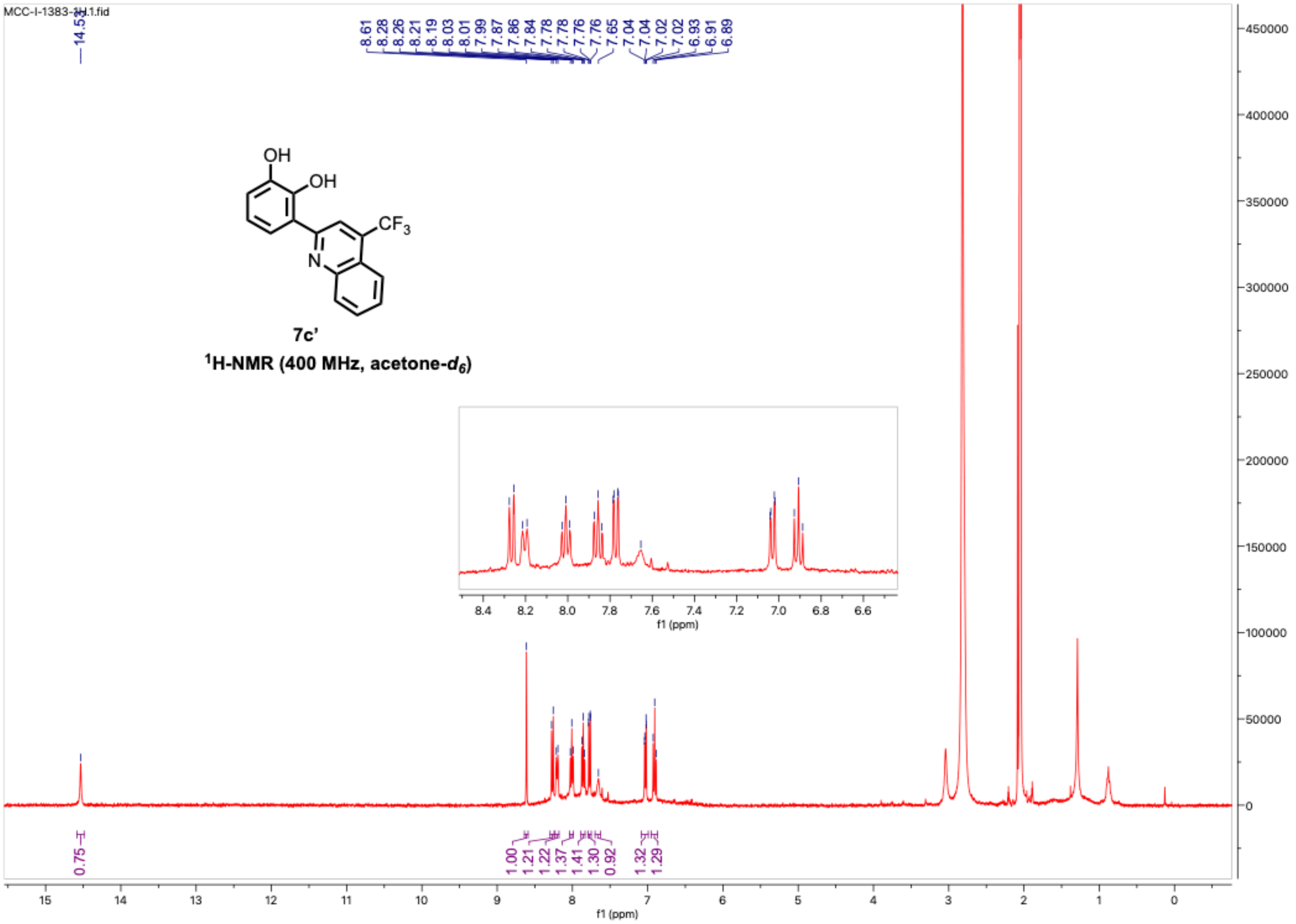
MCC-I-1383-1H.1.fid
14.53
8.61 8.28 8.26 8.21 8.19 8.03 8.01 7.99 7.87 7.86 7.84 7.78 7.78 7.76 7.76 7.65 7.04 7.04 7.02 7.02 6.93 6.91 6.89
OH
OH
CF3
N
7c'
1H-NMR (400 MHz, acetone-d6)
0.75
1.00 1.21 1.22 1.37 1.41 1.30 0.92 1.32 1.29
f1 (ppm)

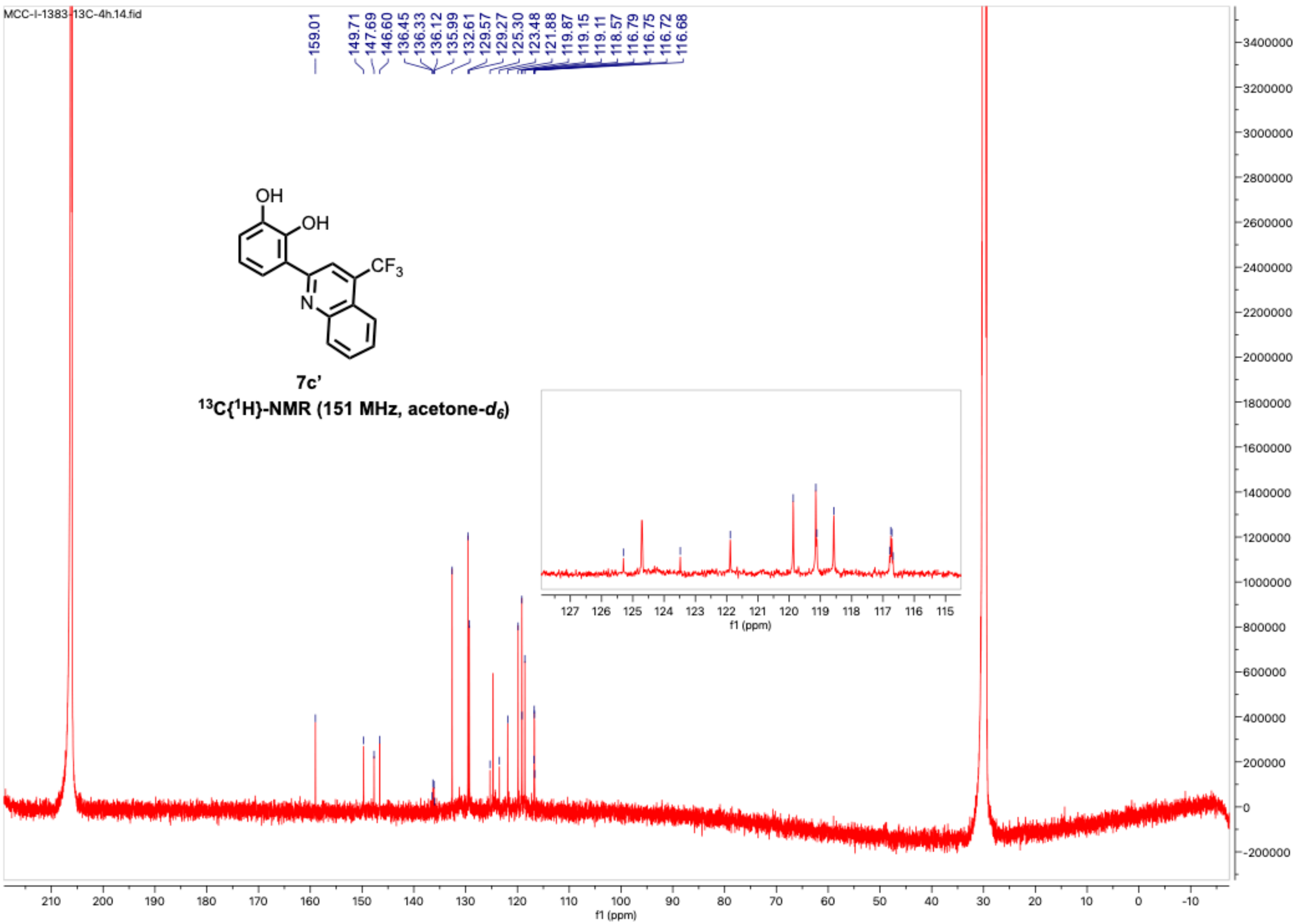
MCC-I-1383-13C-4h.14.fid
159.01
149.71
147.69
146.60
136.45
136.33
136.12
135.99
132.61
129.57
129.27
125.30
123.48
121.88
119.87
119.15
119.11
118.57
116.79
116.75
116.72
116.68
OH
OH
CF3
N
7c'
13C{1H}-NMR (151 MHz, acetone-d6)
f1 (ppm)

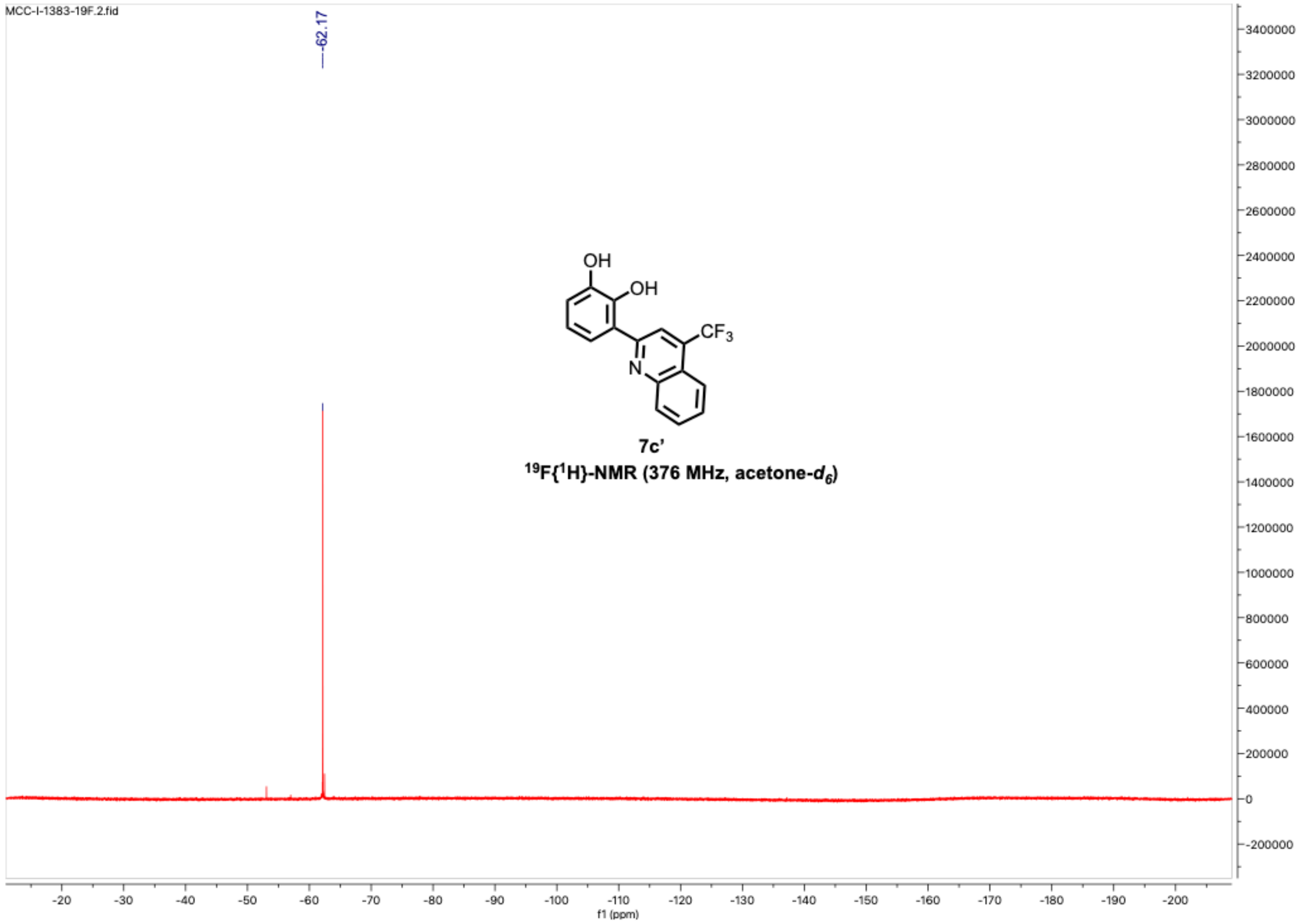

7c'

$^{19}F\{^1H\}$-NMR (376 MHz, acetone-d_6)

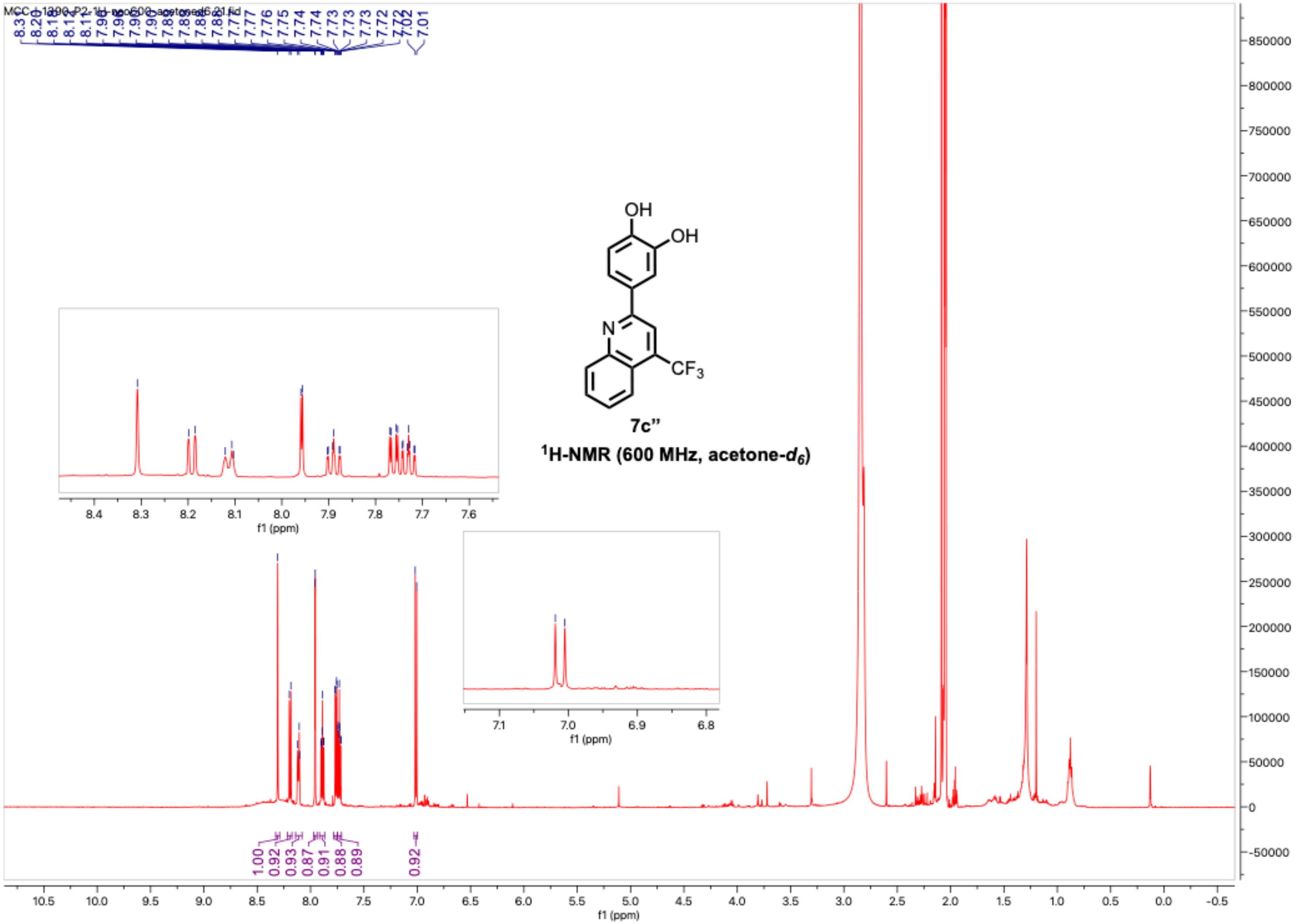

OH
OH
N
CF3
7c"
1H-NMR (600 MHz, acetone-d6)
f1 (ppm)

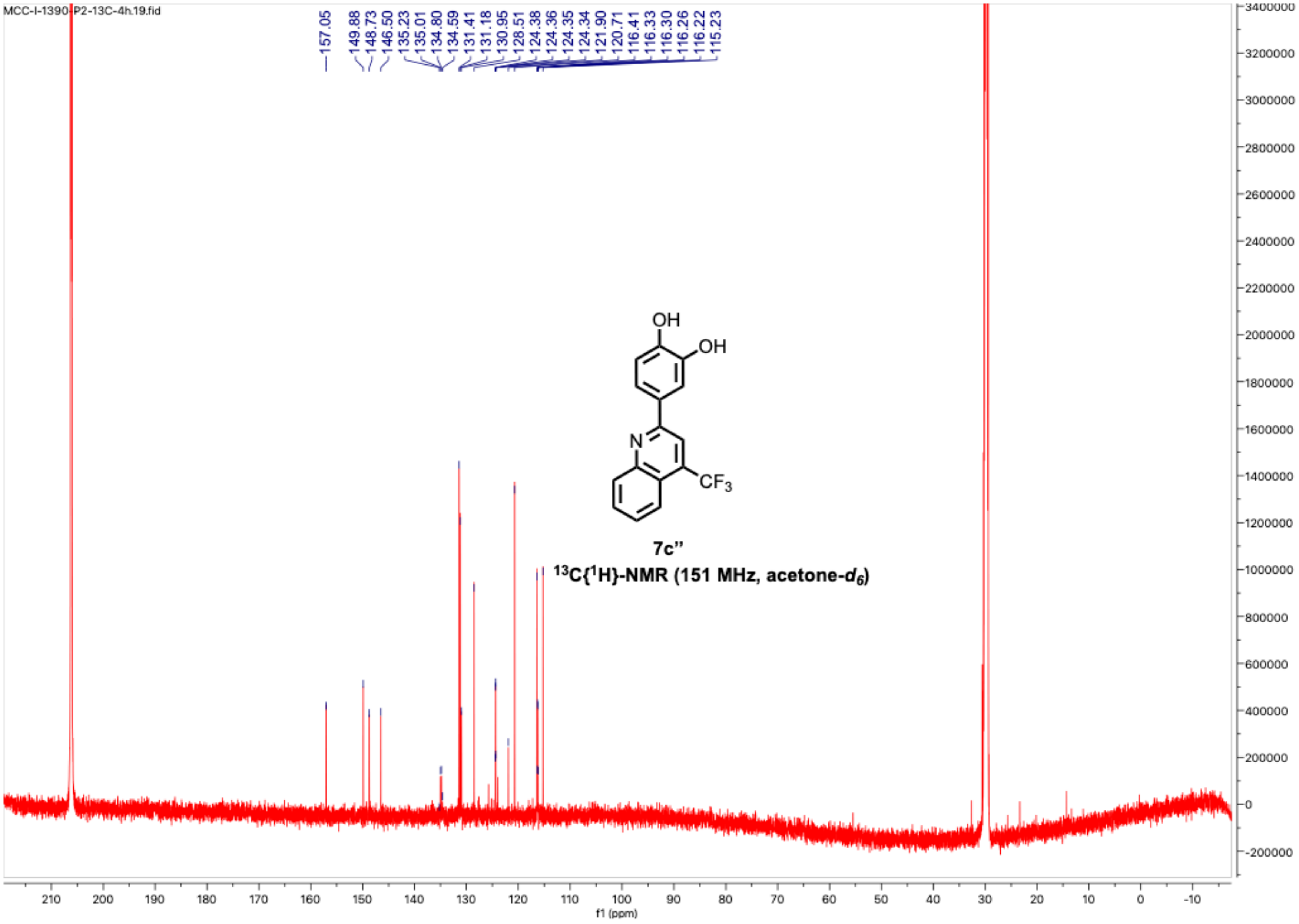

MCC-I-1390-P2-13C-4h.19.fid
157.05
149.88
148.73
146.50
135.23
135.01
134.80
134.59
131.41
131.18
130.95
128.51
124.38
124.36
124.35
124.34
121.90
120.71
116.41
116.33
116.30
116.26
116.22
115.23
OH
OH
N
CF3
7c”
13C{1H}-NMR (151 MHz, acetone-d6)
f1 (ppm)

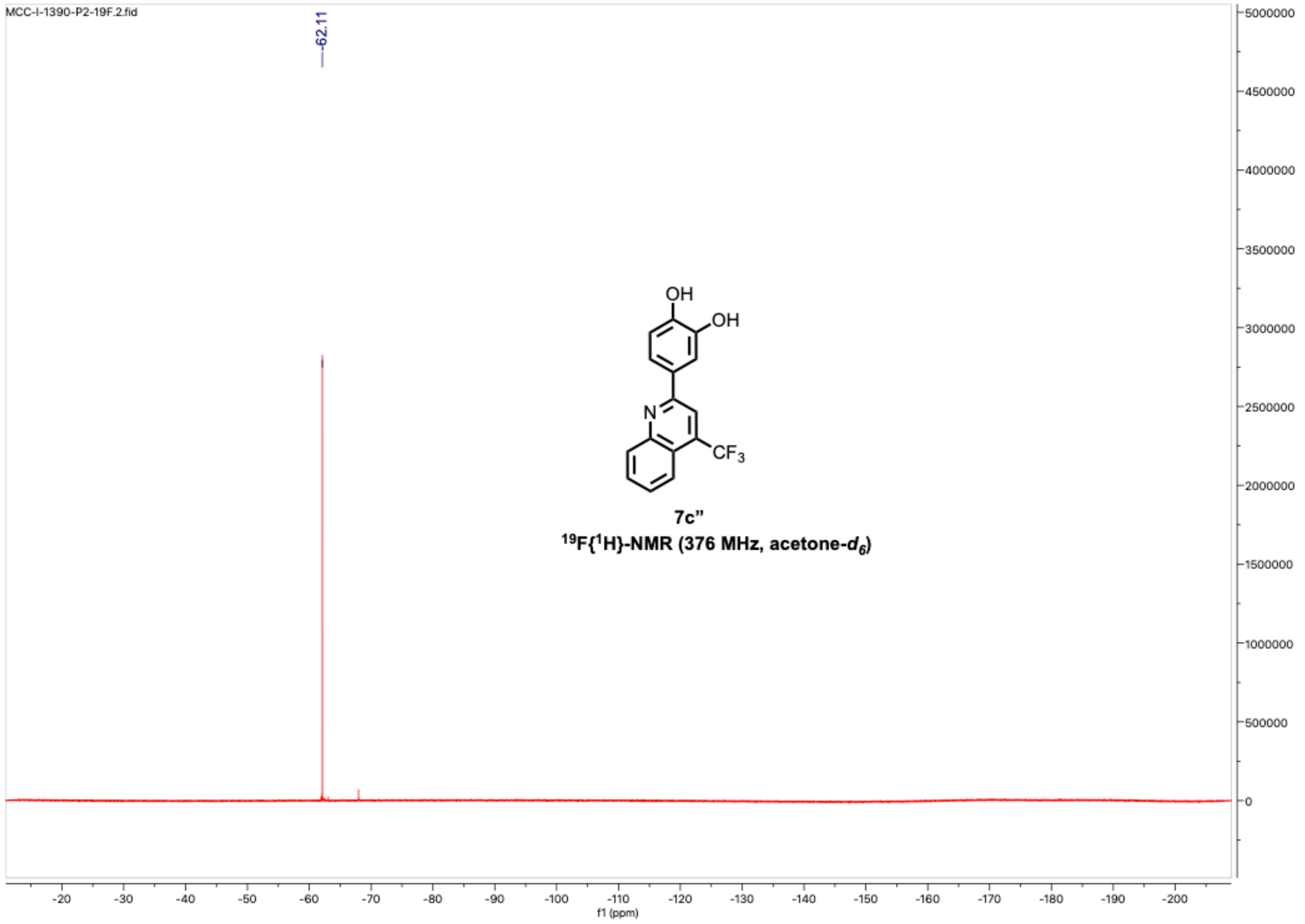

7c"

$^{19}F\{^{1}H\}$-NMR (376 MHz, acetone-d_6)

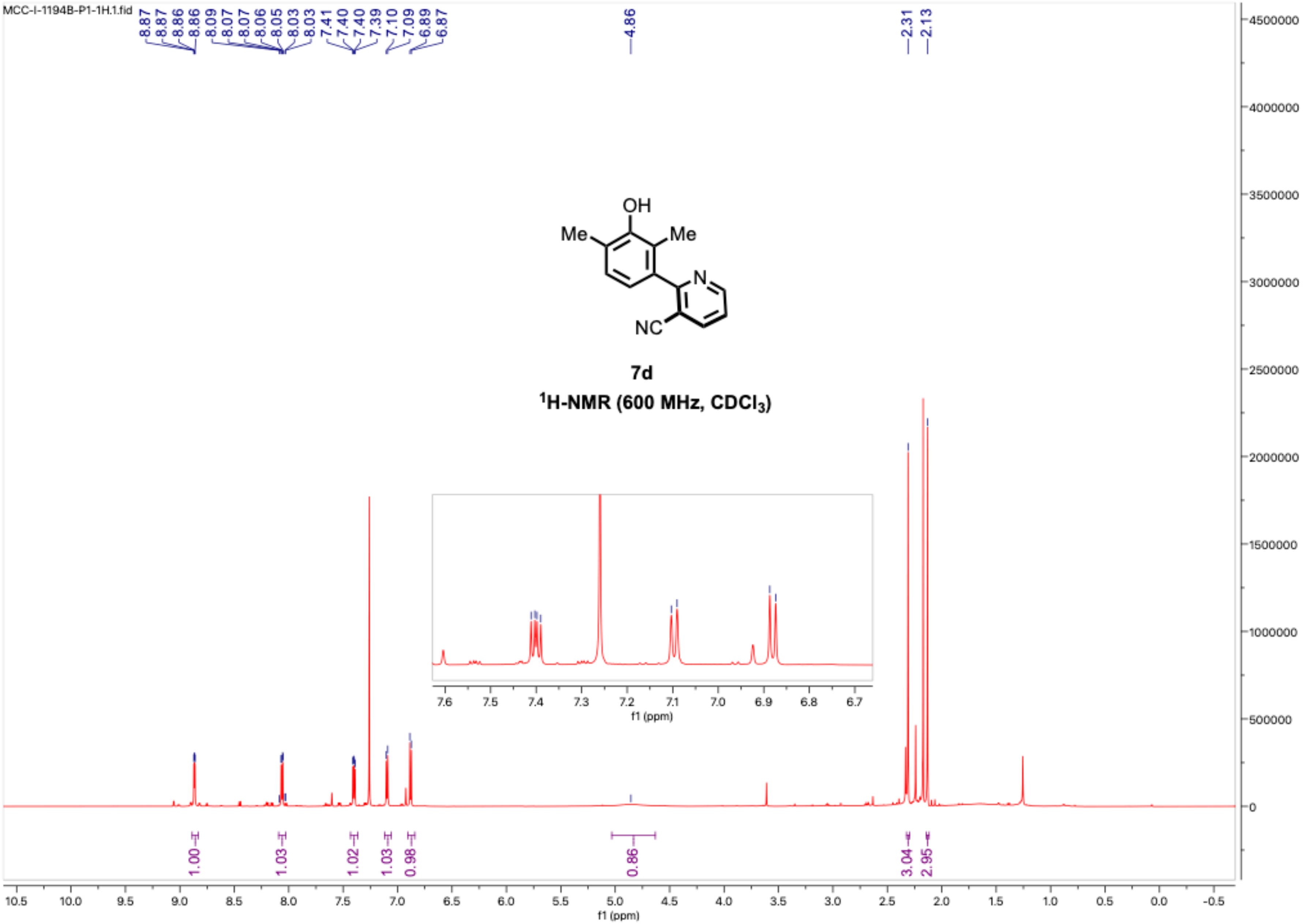

MCC-I-1194B-P1-1H.1.fid
8.87
8.87
8.86
8.86
8.09
8.07
8.07
8.06
8.05
8.03
8.03
7.41
7.40
7.40
7.39
7.10
7.09
6.89
6.87
4.86
2.31
2.13
OH
Me
Me
N
NC
7d
^{1}H-NMR (600 MHz, $CDCl_3$)
1.00
1.03
1.02
1.03
0.98
0.86
3.04
2.95
f1 (ppm)

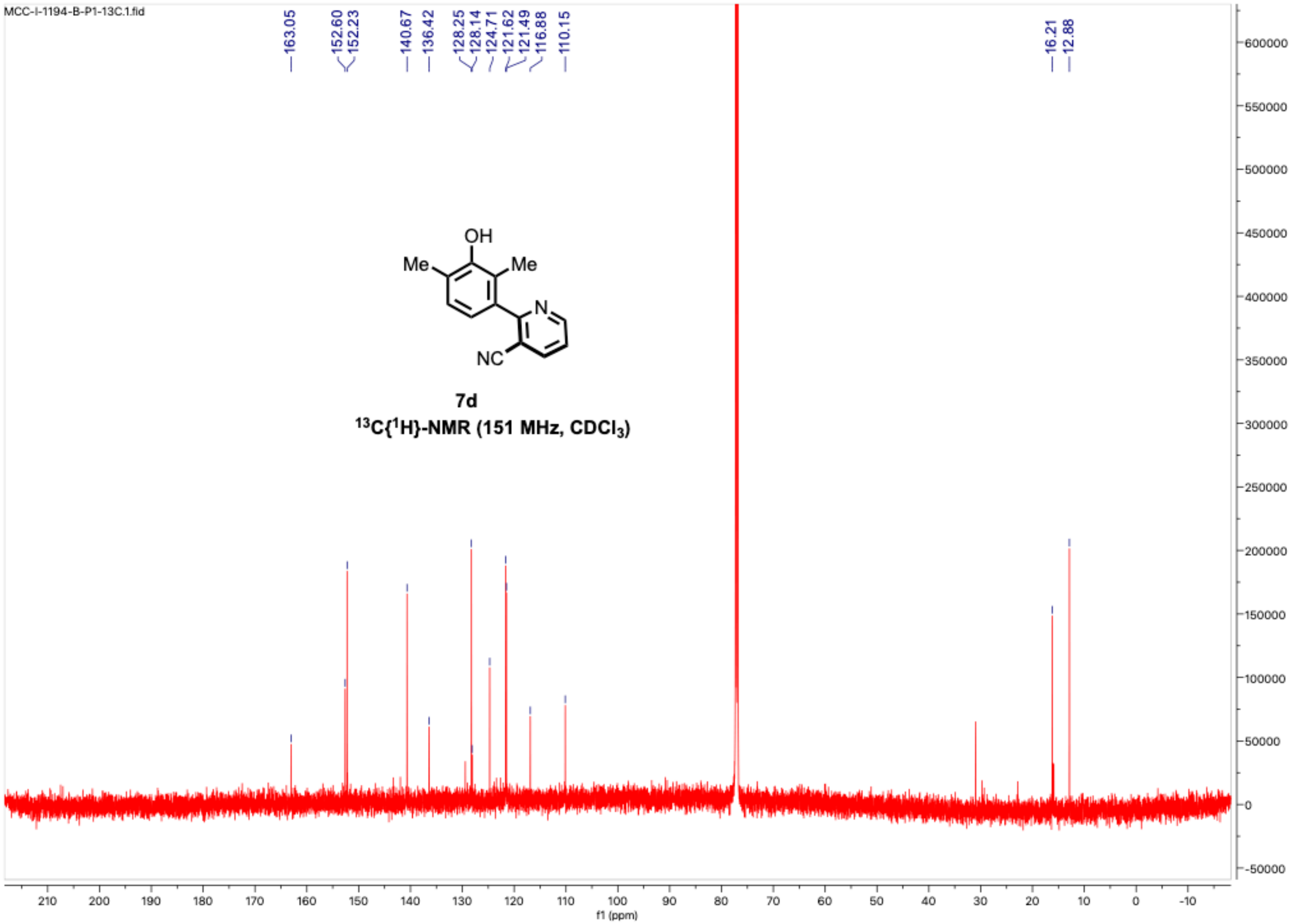
MCC-I-1194-B-P1-13C.1.fid
163.05
152.60
152.23
140.67
136.42
128.25
128.14
124.71
121.62
121.49
116.88
110.15
16.21
12.88
OH
Me
Me
N
NC
7d
$^{13}C\{^{1}H\}$-NMR (151 MHz, $CDCl_3$)
f1 (ppm)

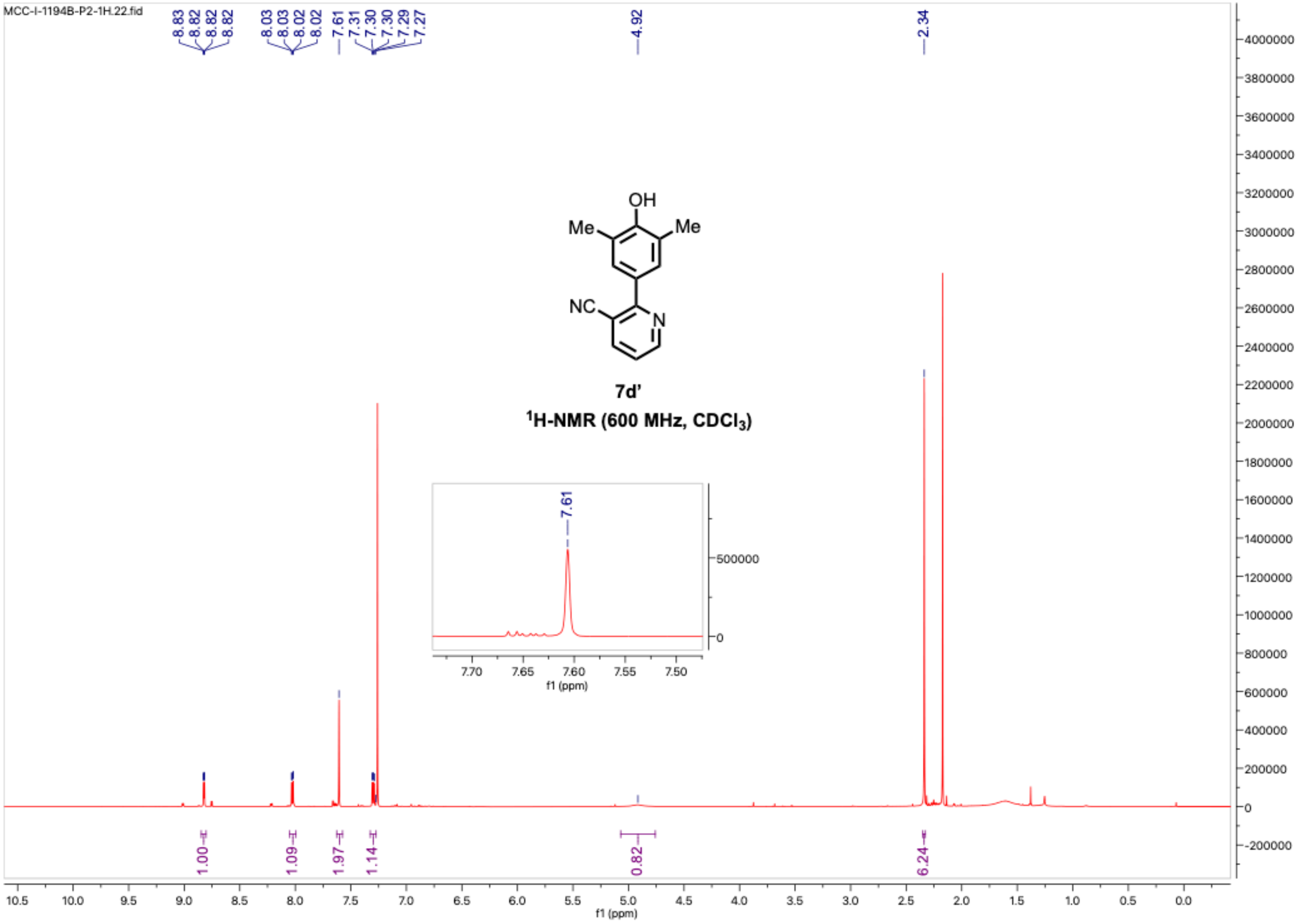
MCC-I-1194B-P2-1H.22.fid
8.83
8.82
8.82
8.82
8.03
8.03
8.02
8.02
7.61
7.31
7.30
7.30
7.29
7.27
4.92
2.34
OH
Me
Me
NC
N
7d'
¹H-NMR (600 MHz, CDCl₃)
7.61
500000
0
7.70
7.65
7.60
7.55
7.50
f1 (ppm)
1.00
1.09
1.97
1.14
0.82
6.24
f1 (ppm)

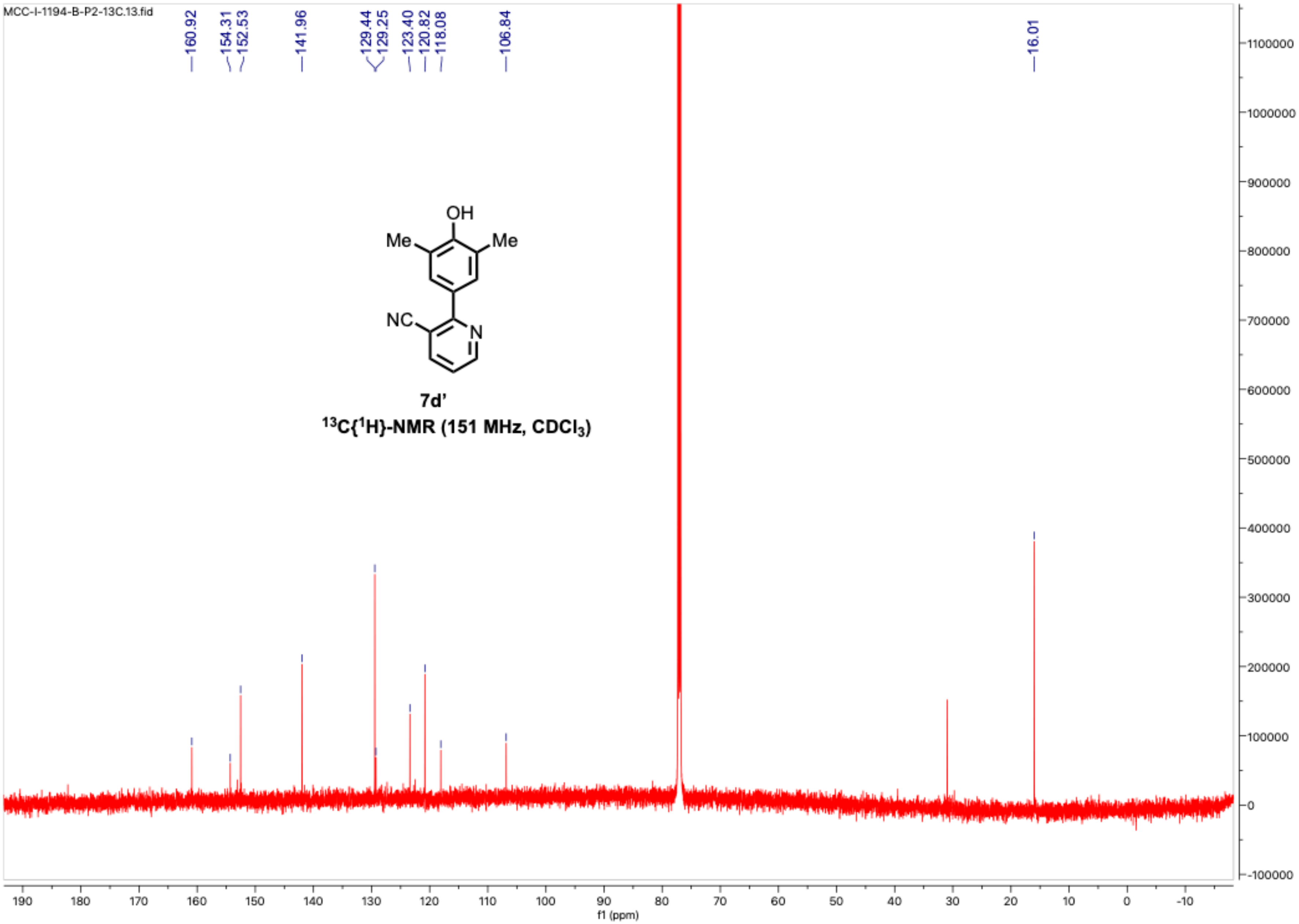
MCC-I-1194-B-P2-13C.13.fid
160.92
154.31
152.53
141.96
129.44
129.25
123.40
120.82
118.08
106.84
16.01
OH
Me
Me
NC
N
7d'
13C{1H}-NMR (151 MHz, CDCl3)
f1 (ppm)

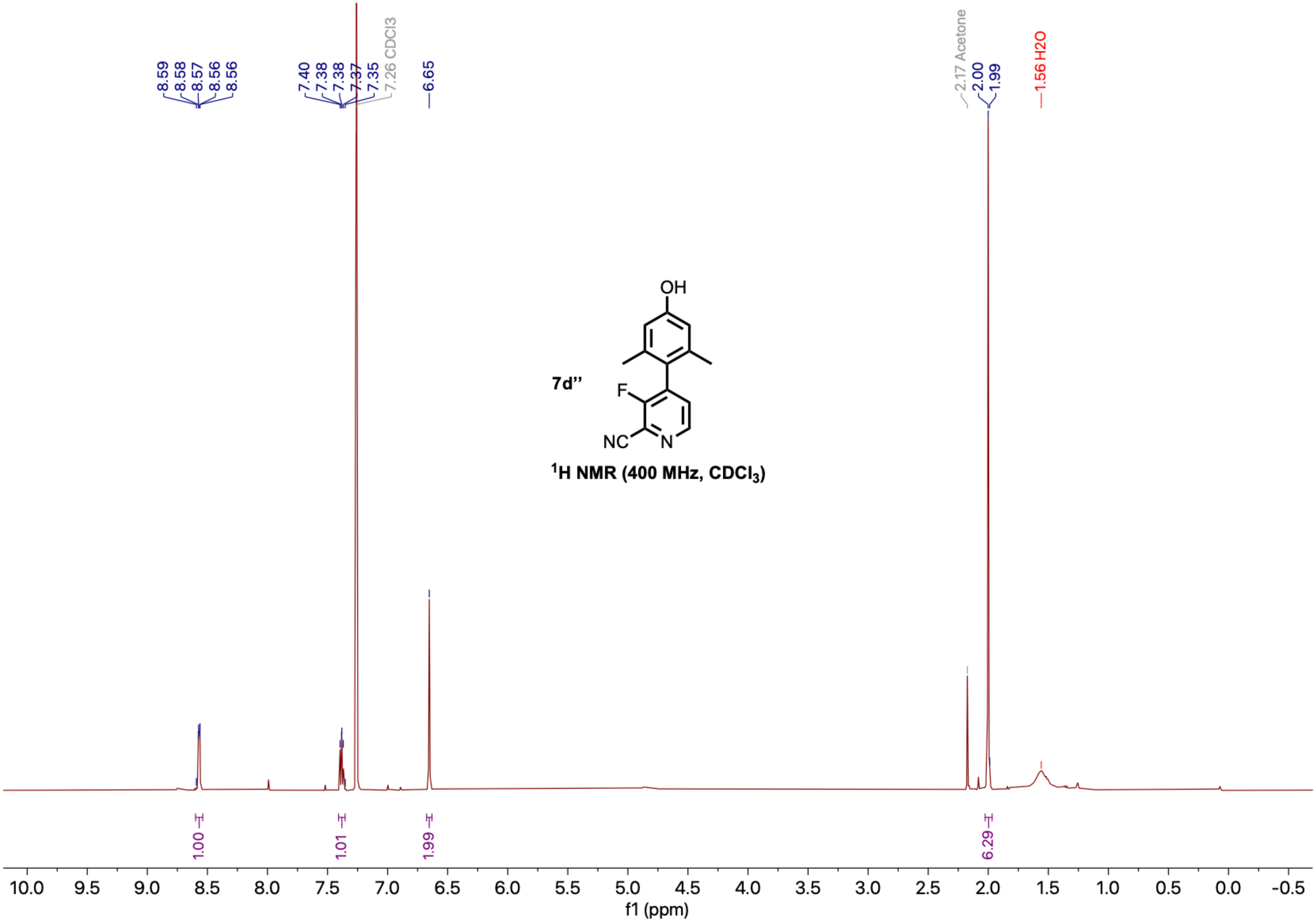

8.59
8.58
8.57
8.56
8.56
7.40
7.38
7.38
7.37
7.35
7.26 CDCl3
6.65
2.17 Acetone
2.00
1.99
1.56 H2O
OH
F
NC
N
7d''
1H NMR (400 MHz, CDCl3)
1.00
1.01
1.99
6.29
10.0
9.5
9.0
8.5
8.0
7.5
7.0
6.5
6.0
5.5
5.0
4.5
4.0
3.5
3.0
2.5
2.0
1.5
1.0
0.5
0.0
-0.5
f1 (ppm)

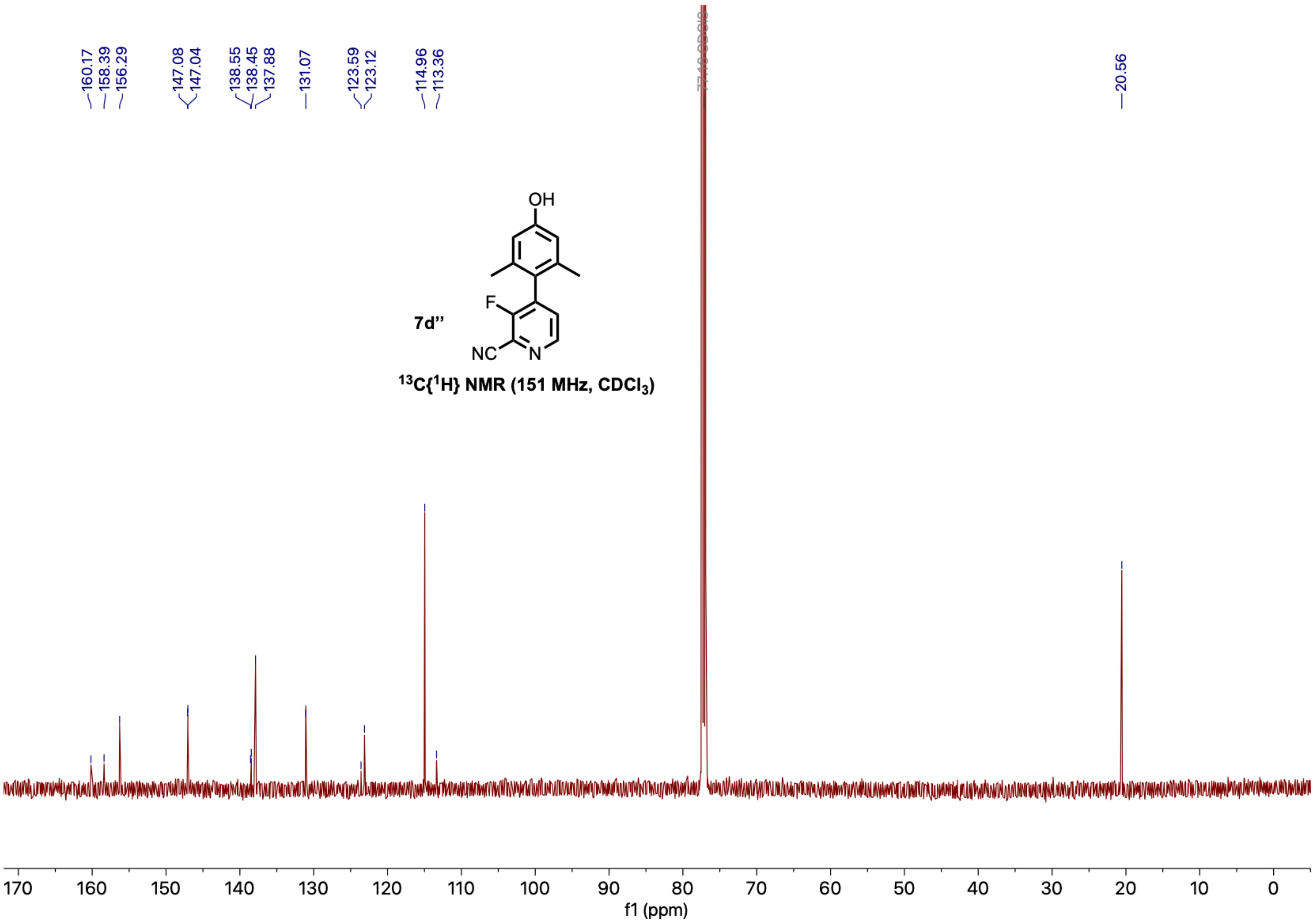
160.17
158.39
156.29
147.08
147.04
138.55
138.45
137.88
131.07
123.59
123.12
114.96
113.36
20.56
OH
F
NC
N
7d''
$^{13}C\{^1H\}$ NMR (151 MHz, $CDCl_3$)
170 160 150 140 130 120 110 100 90 80 70 60 50 40 30 20 10 0
f1 (ppm)

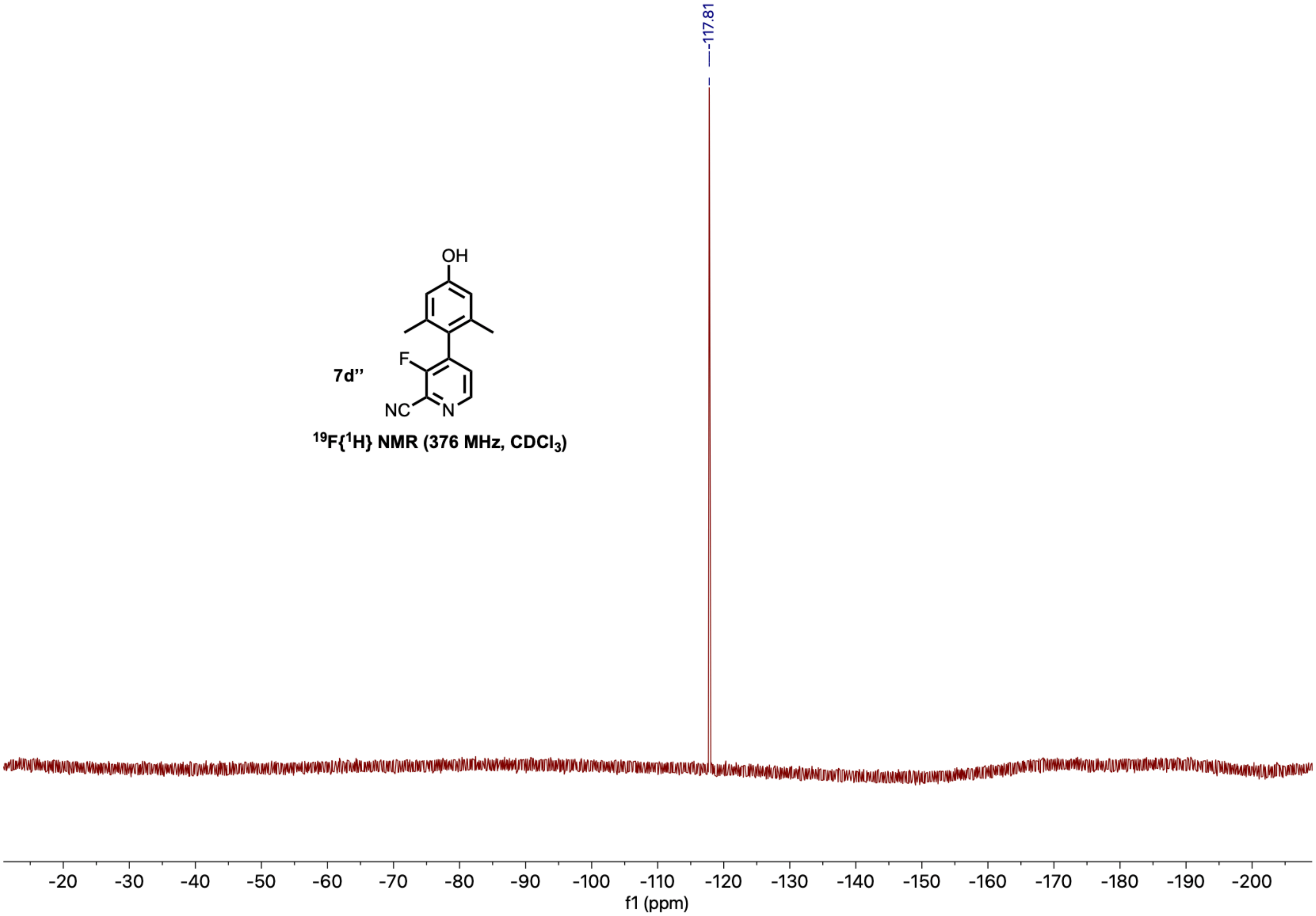

-117.81
7d''
OH
F
NC
N
$^{19}F\{^{1}H\}$ NMR (376 MHz, $CDCl_3$)
-20 -30 -40 -50 -60 -70 -80 -90 -100 -110 -120 -130 -140 -150 -160 -170 -180 -190 -200
f1 (ppm)

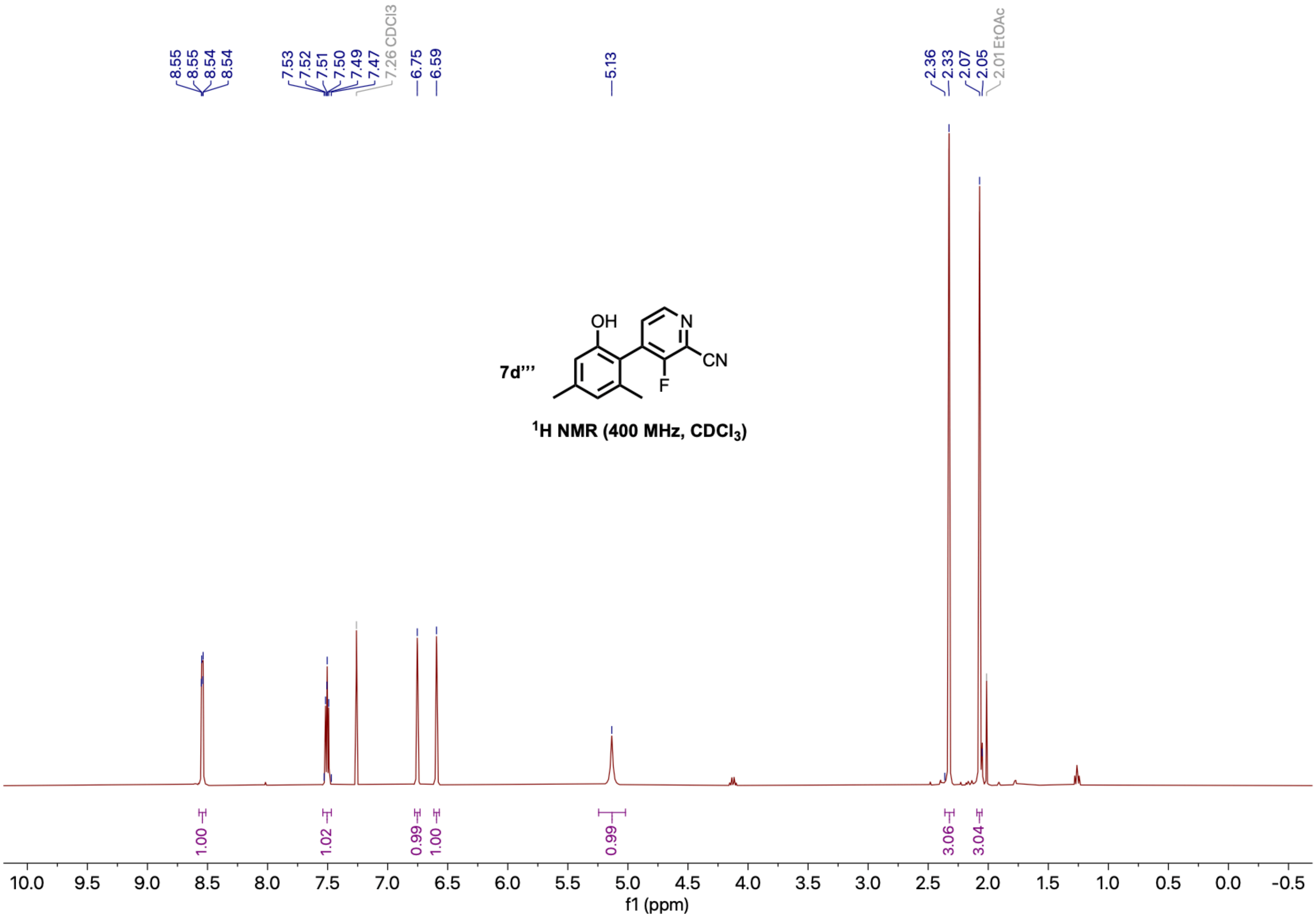
8.55
8.55
8.54
8.54
7.53
7.52
7.51
7.50
7.49
7.47
7.26 CDCl3
6.75
6.59
5.13
2.36
2.33
2.07
2.05
2.01 EtOAc
OH
N
CN
F
7d'''
1H NMR (400 MHz, CDCl3)
1.00
1.02
0.99
1.00
0.99
3.06
3.04
10.0
9.5
9.0
8.5
8.0
7.5
7.0
6.5
6.0
5.5
5.0
4.5
4.0
3.5
3.0
2.5
2.0
1.5
1.0
0.5
0.0
-0.5
f1 (ppm)

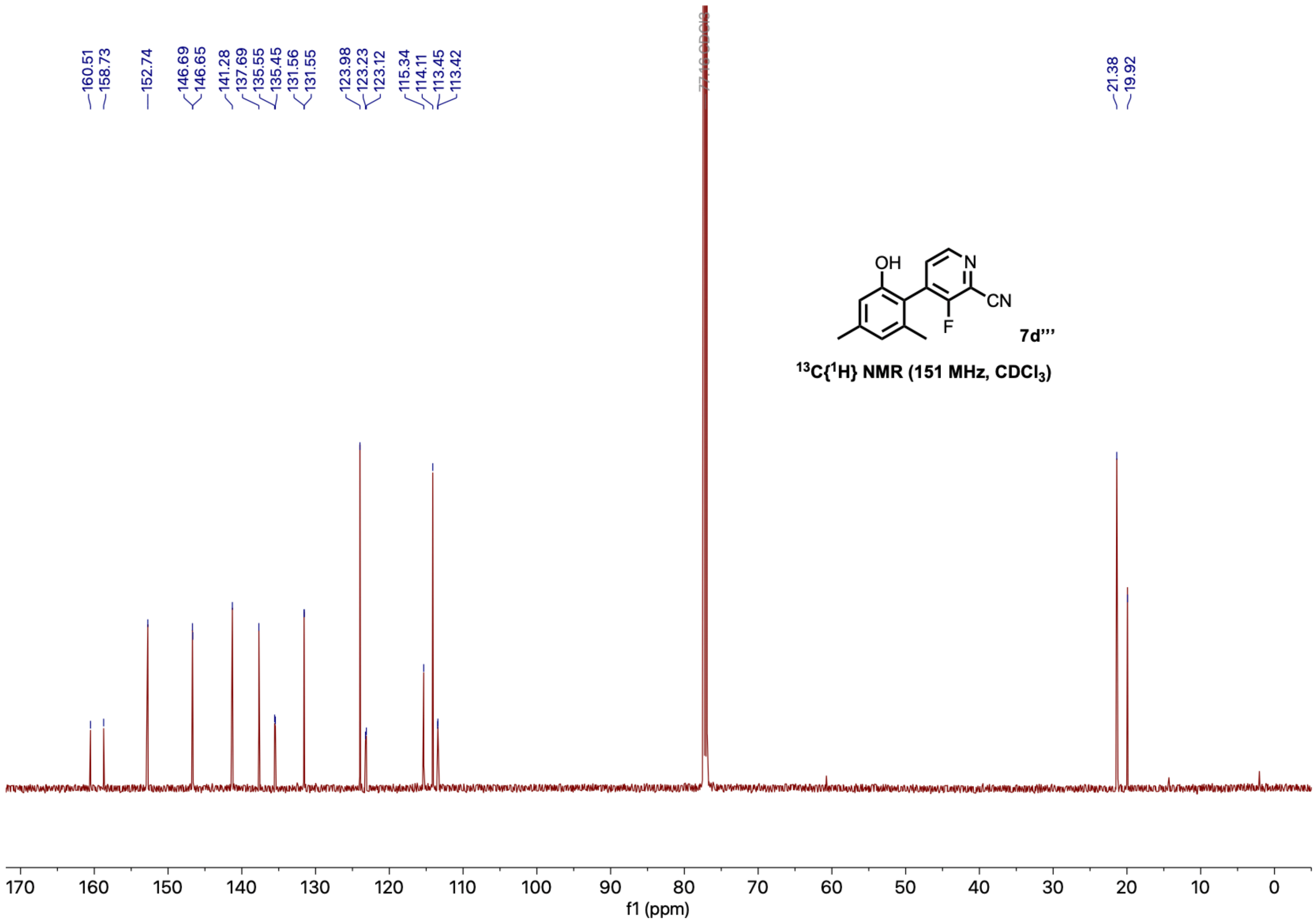
160.51
158.73
152.74
146.69
146.65
141.28
137.69
135.55
135.45
131.56
131.55
123.98
123.23
123.12
115.34
114.11
113.45
113.42
21.38
19.92
OH
N
CN
F
7d'''
$^{13}C\{^{1}H\}$ NMR (151 MHz, CDCl$_3$)
f1 (ppm)

7d'''

$^{19}F\{^1H\}$ NMR (376 MHz, $CDCl_3$)

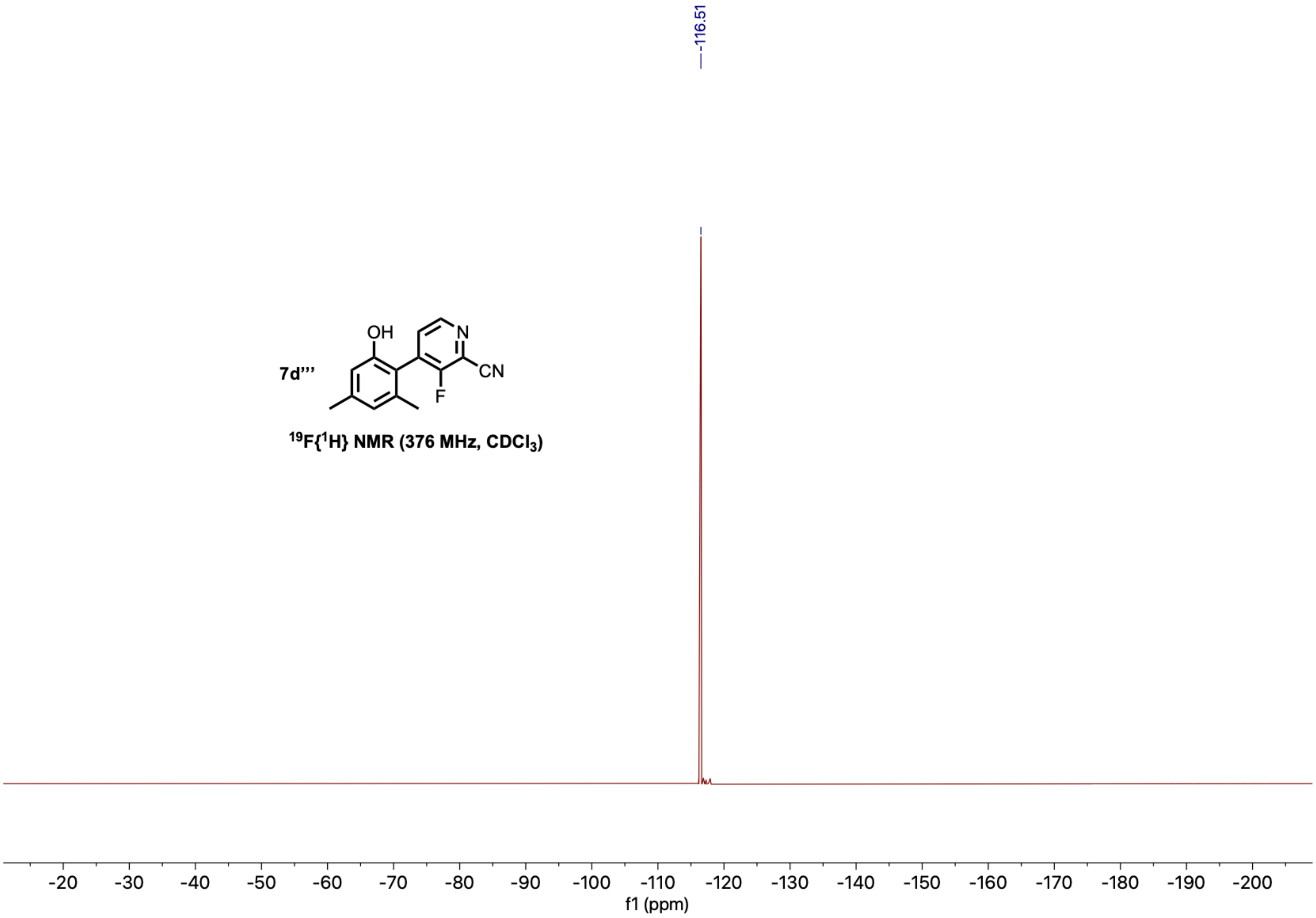

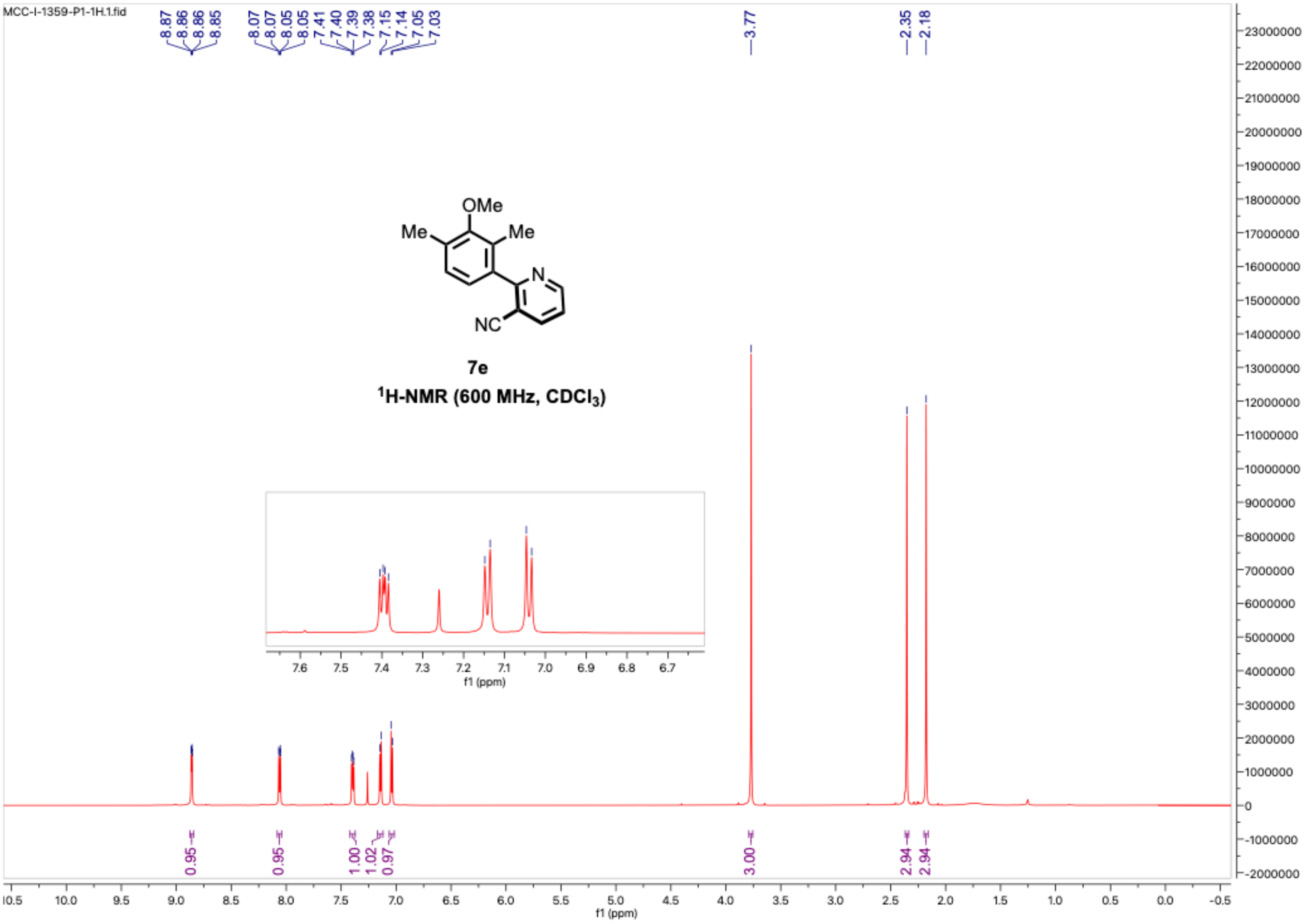
MCC-I-1359-P1-1H.1.fid
8.87
8.86
8.86
8.85
8.07
8.07
8.05
8.05
7.41
7.40
7.39
7.38
7.15
7.14
7.05
7.03
3.77
2.35
2.18
OMe
Me
Me
N
NC
7e
1H-NMR (600 MHz, CDCl3)
0.95
0.95
1.00
1.02
0.97
3.00
2.94
2.94
f1 (ppm)

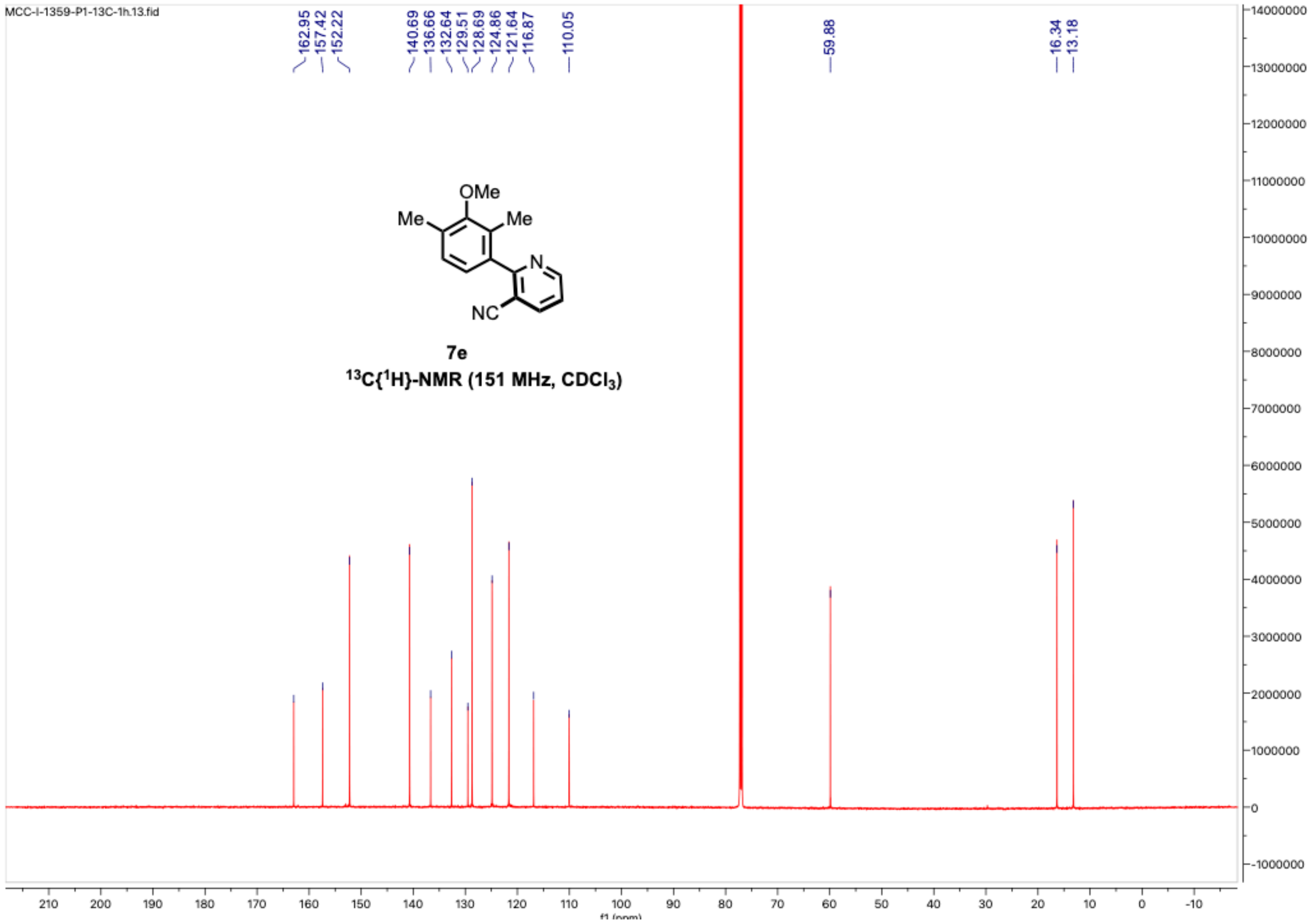

MCC-I-1359-P1-13C-1h.13.fid
162.95
157.42
152.22
140.69
136.66
132.64
129.51
128.69
124.86
121.64
116.87
110.05
59.88
16.34
13.18
OMe
Me
Me
N
NC
7e
13C{1H}-NMR (151 MHz, CDCl3)
f1 (ppm)

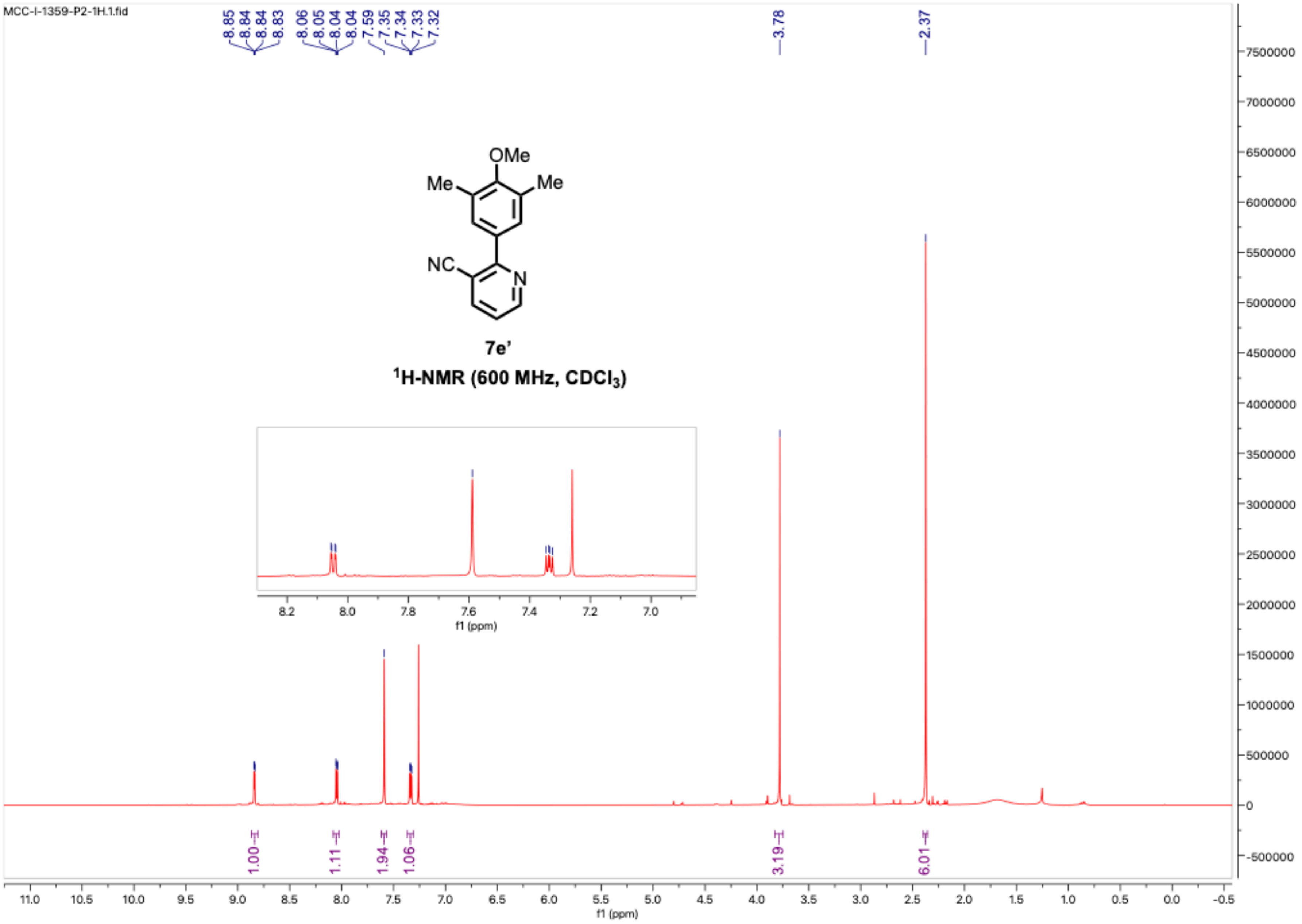
MCC-I-1359-P2-1H.1.fid
8.85
8.84
8.84
8.83
8.06
8.05
8.04
8.04
7.59
7.35
7.34
7.33
7.32
3.78
2.37
OMe
Me
Me
NC
N
7e'
1H-NMR (600 MHz, CDCl3)
1.00
1.11
1.94
1.06
3.19
6.01
f1 (ppm)

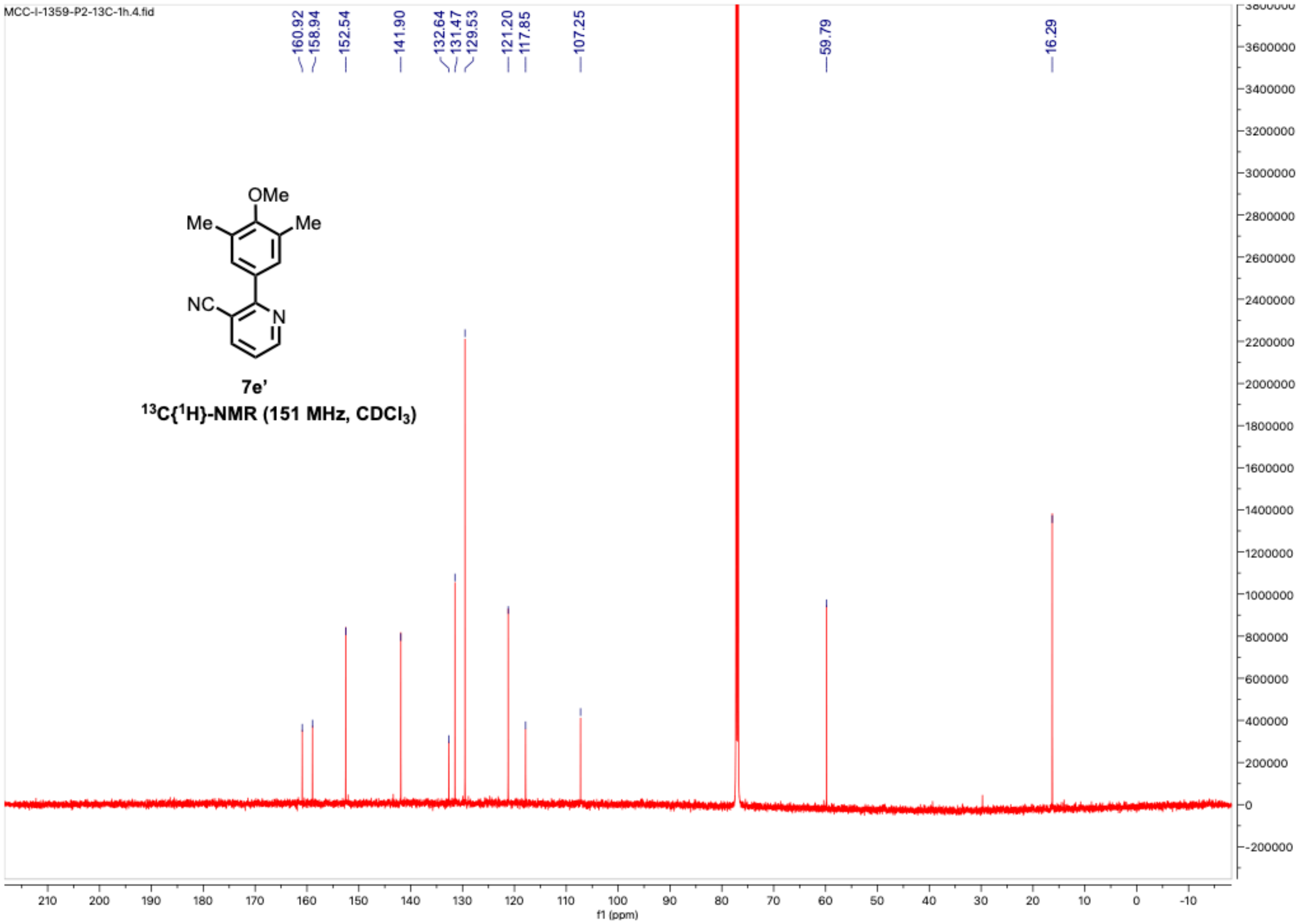
MCC-I-1359-P2-13C-1h.4.fid
160.92
158.94
152.54
141.90
132.64
131.47
129.53
121.20
117.85
107.25
59.79
16.29
OMe
Me
Me
NC
N
7e'
$^{13}C\{^1H\}$-NMR (151 MHz, $CDCl_3$)
f1 (ppm)

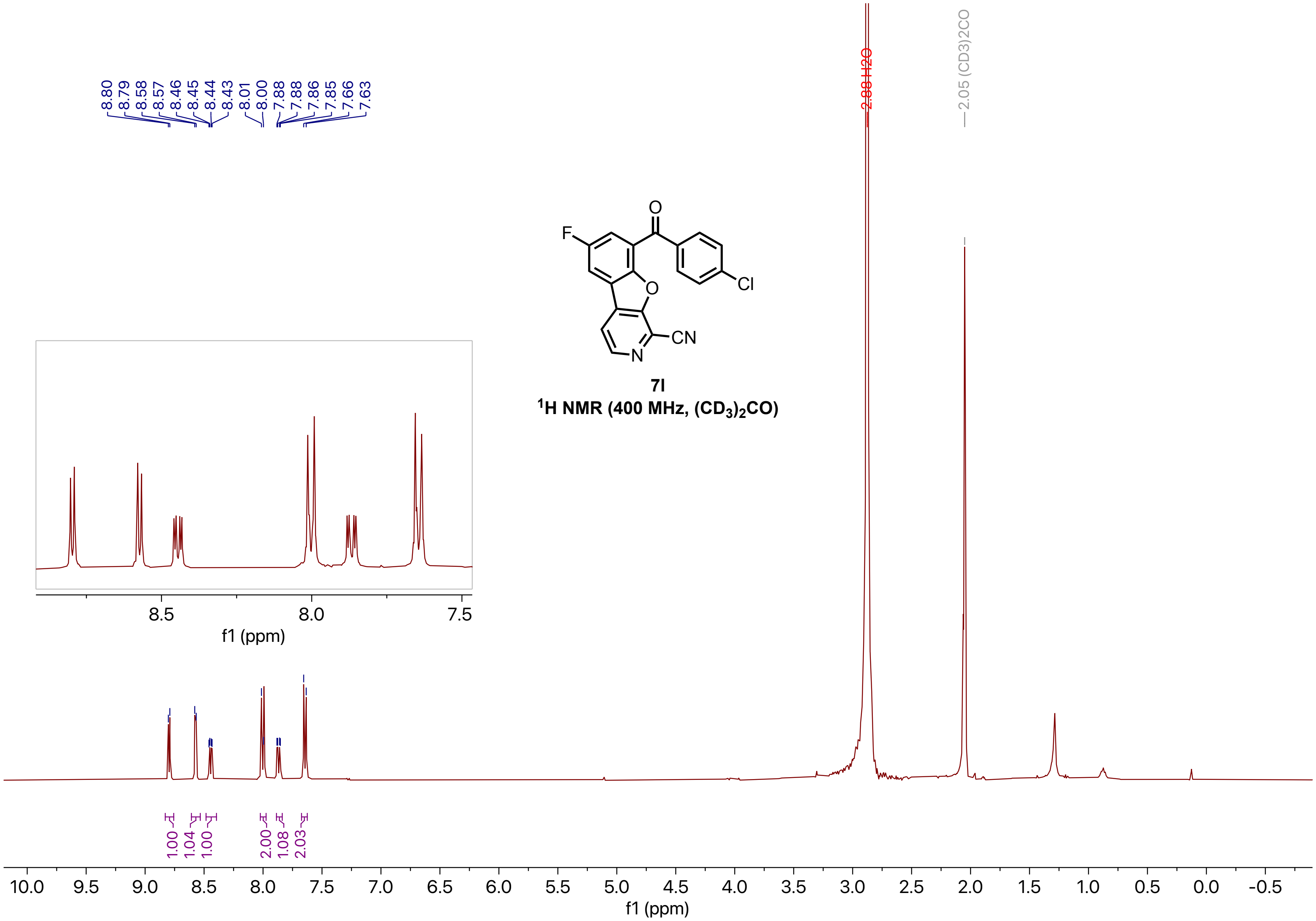

8.80
8.79
8.58
8.57
8.46
8.45
8.44
8.43
8.01
8.00
7.88
7.88
7.86
7.85
7.66
7.63
2.88 H2O
2.05 (CD3)2CO
F
O
Cl
CN
N
7l
^{1}H NMR (400 MHz, $(CD_3)_2CO$)
8.5
8.0
7.5
f1 (ppm)
1.00
1.04
1.00
2.00
1.08
2.03
10.0 9.5 9.0 8.5 8.0 7.5 7.0 6.5 6.0 5.5 5.0 4.5 4.0 3.5 3.0 2.5 2.0 1.5 1.0 0.5 0.0 -0.5
f1 (ppm)

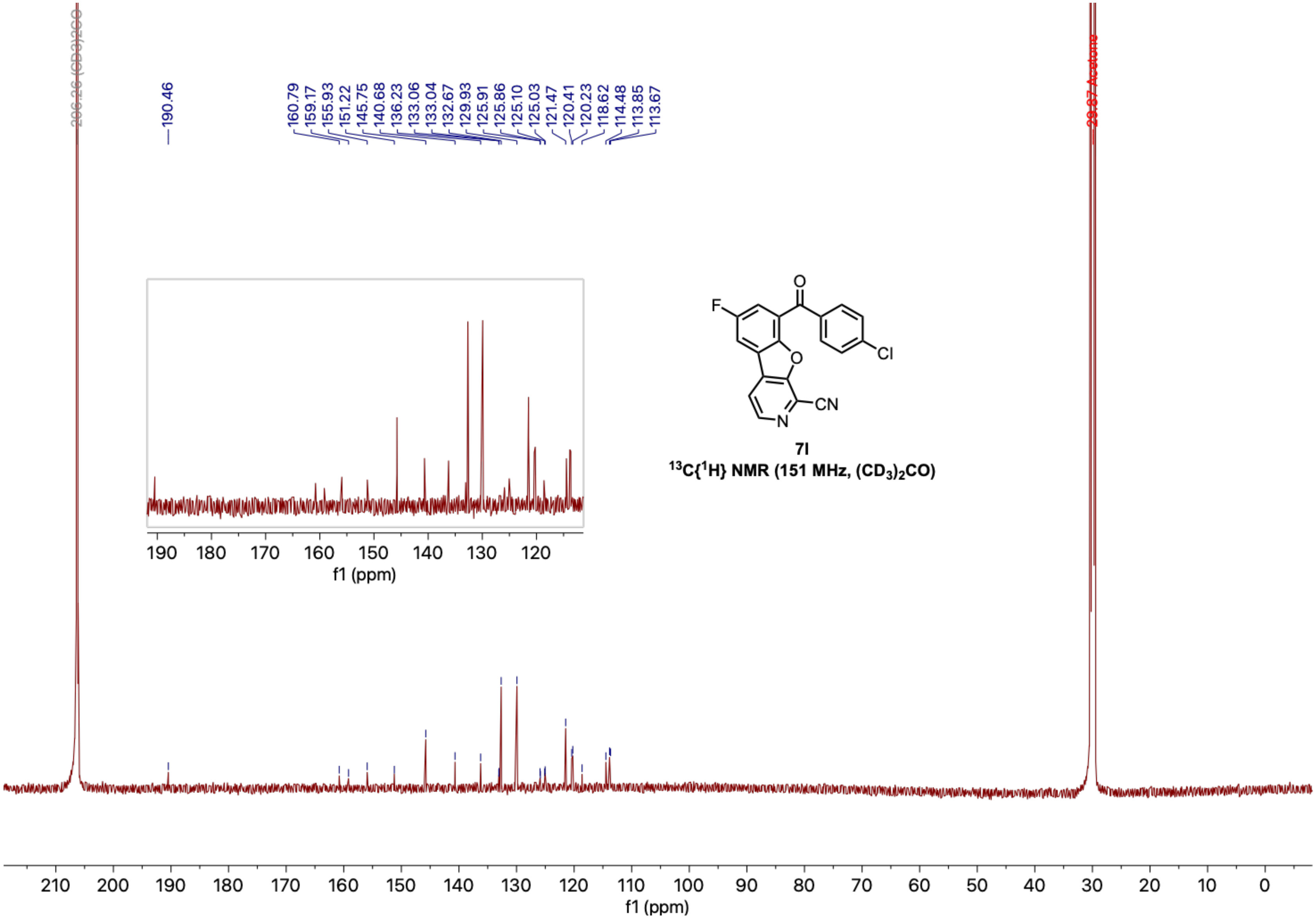

7l
$^{13}C\{^{1}H\}$ NMR (151 MHz, $(CD_3)_2CO$)

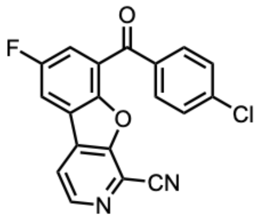

7l

$^{19}F\{^1H\}$ NMR (376 MHz, $(CD_3)_2CO$)

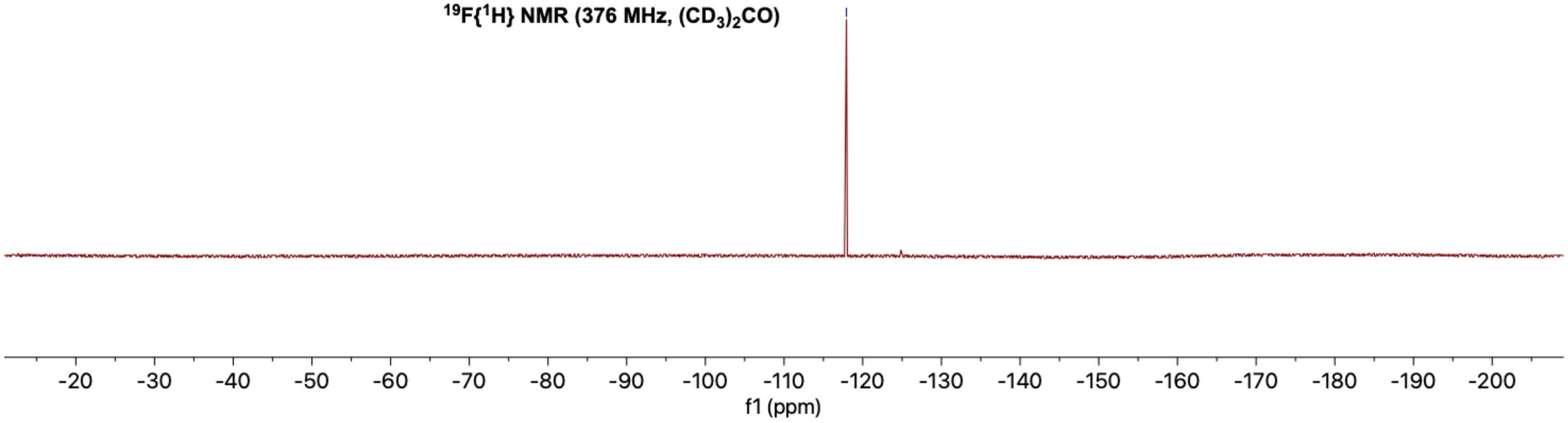

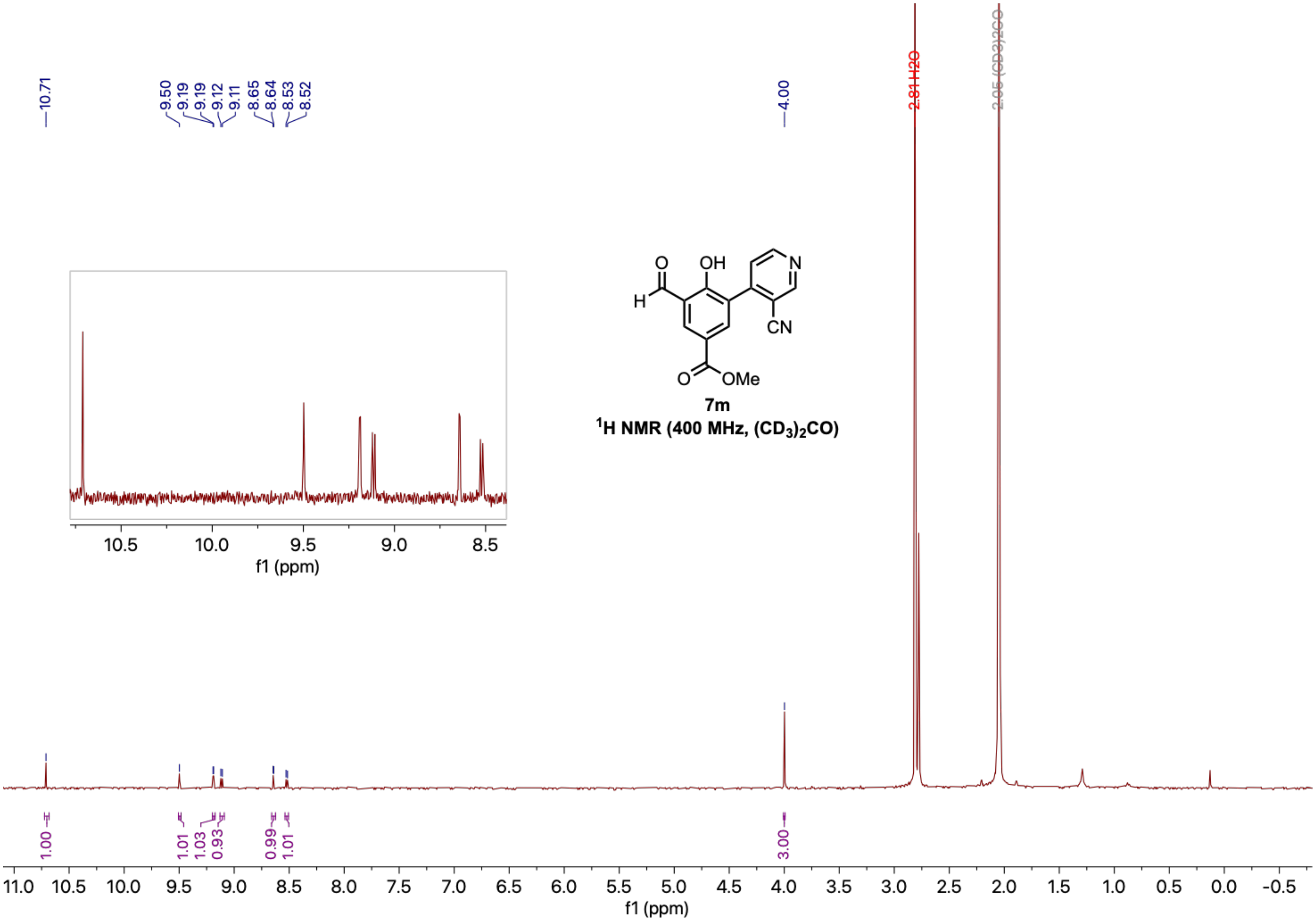

7m

^{1}H NMR (400 MHz, $(CD_3)_2CO$)

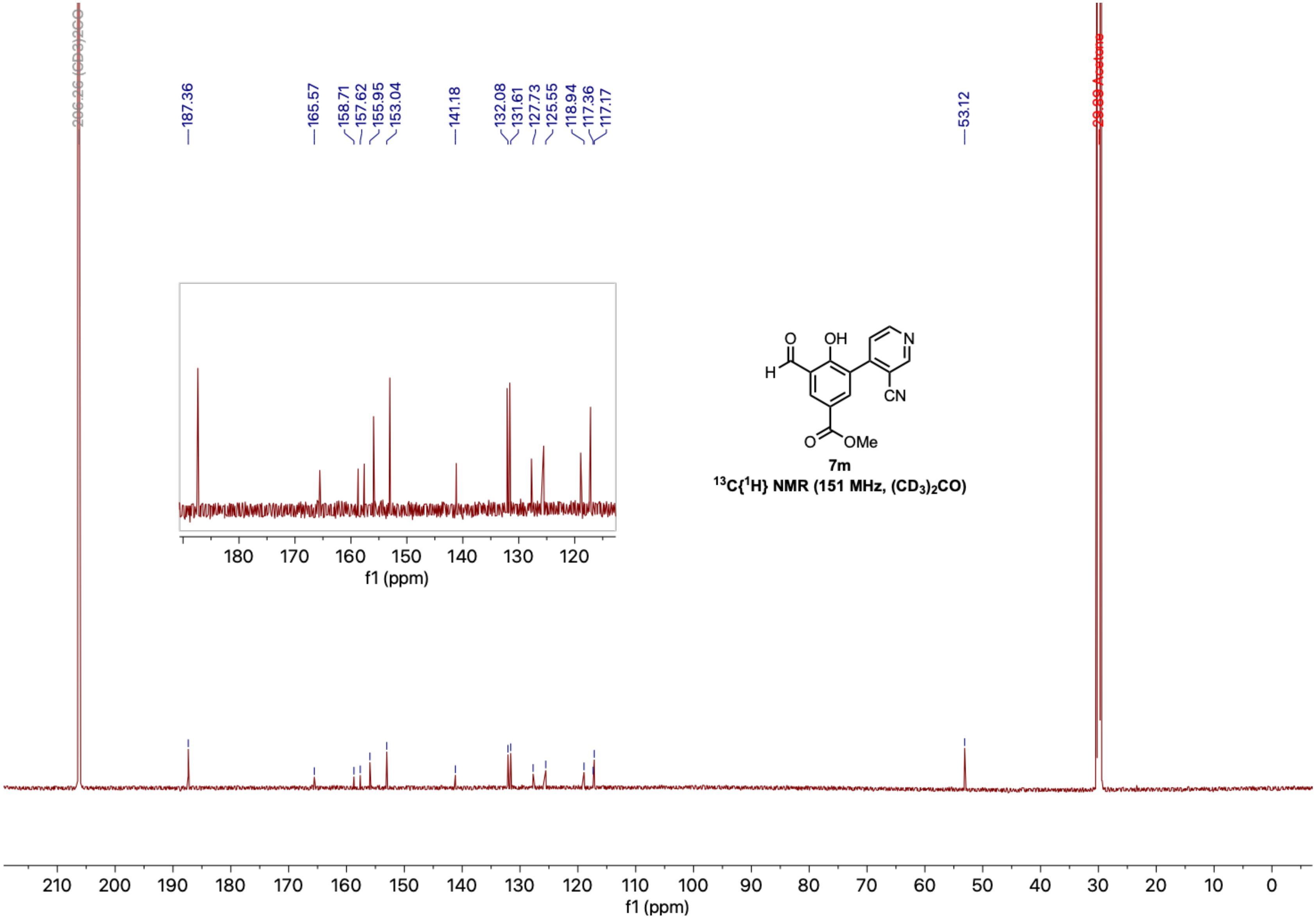

7m
$^{13}C\{^{1}H\}$ NMR (151 MHz, $(CD_3)_2CO$)

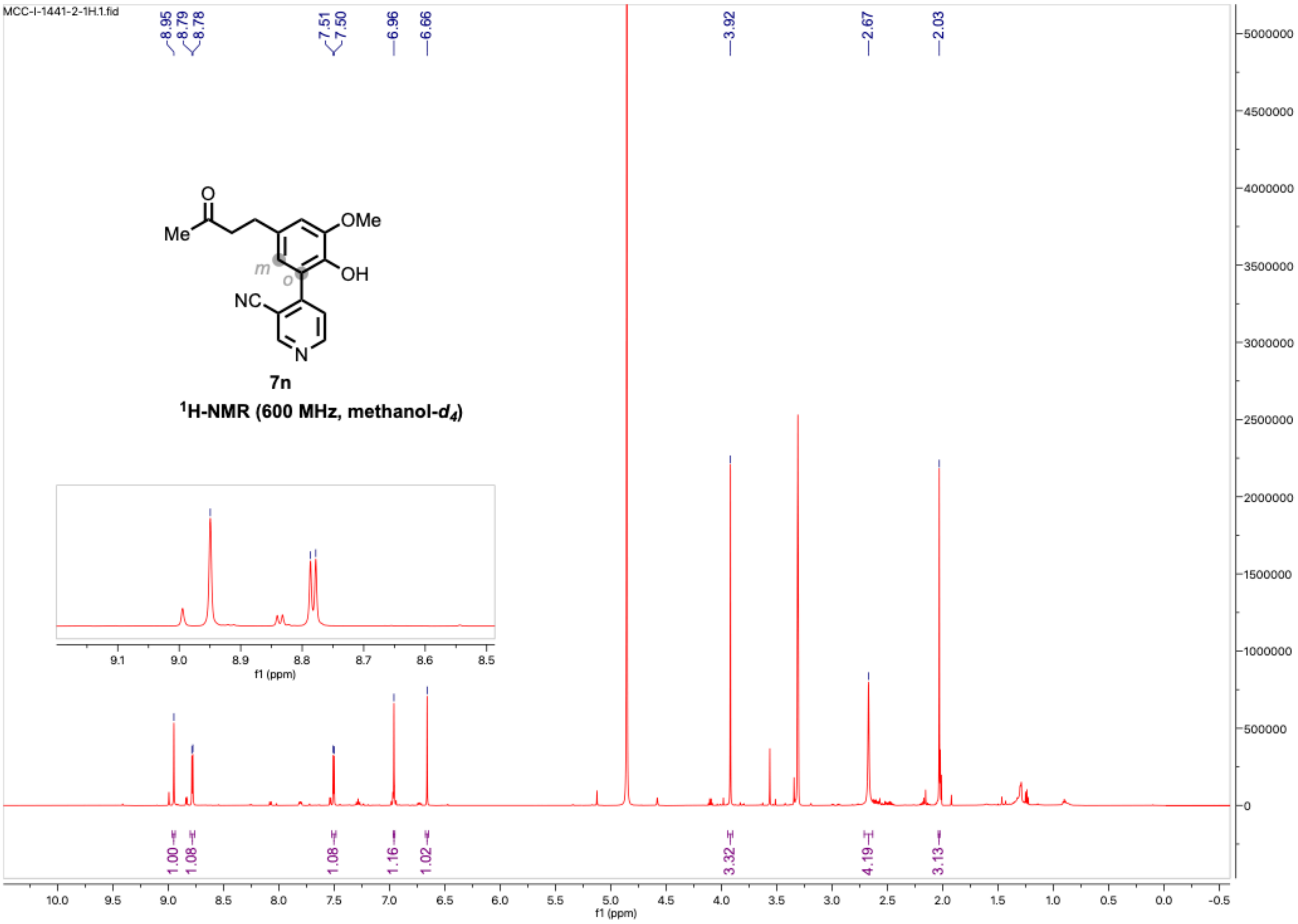
MCC-I-1441-2-1H.1.fid
8.95
8.79
8.78
7.51
7.50
6.96
6.66
3.92
2.67
2.03
O
Me
OMe
OH
m
o
NC
N
7n
1H-NMR (600 MHz, methanol-d4)
9.1
9.0
8.9
8.8
8.7
8.6
8.5
f1 (ppm)
1.00
1.08
1.08
1.16
1.02
3.32
4.19
3.13
10.0
9.5
9.0
8.5
8.0
7.5
7.0
6.5
6.0
5.5
5.0
4.5
4.0
3.5
3.0
2.5
2.0
1.5
1.0
0.5
0.0
-0.5
f1 (ppm)
5000000
4500000
4000000
3500000
3000000
2500000
2000000
1500000
1000000
500000
0

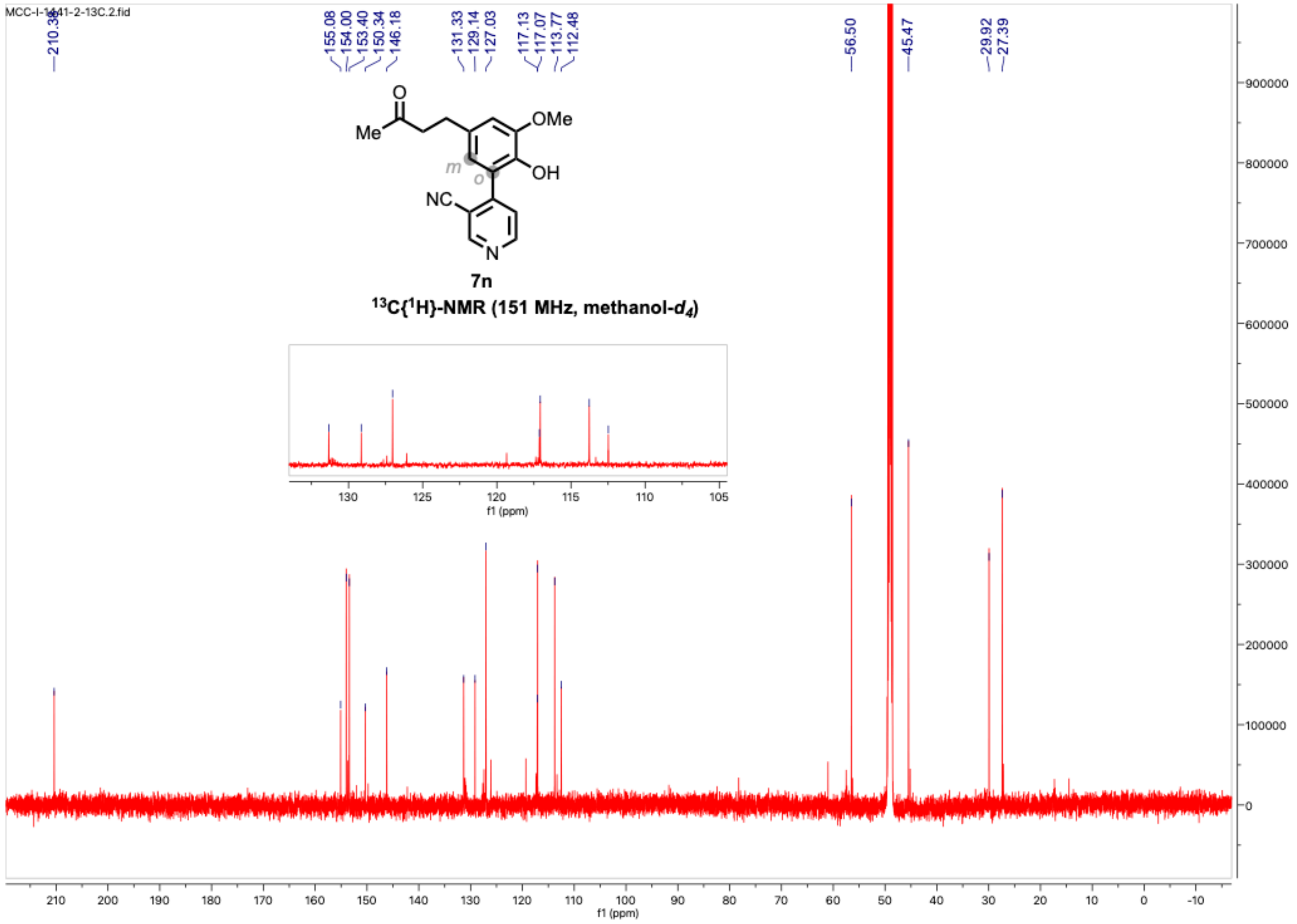
MCC-I-1441-2-13C.2.fid
210.38
155.08
154.00
153.40
150.34
146.18
131.33
129.14
127.03
117.13
117.07
113.77
112.48
56.50
45.47
29.92
27.39
OMe
OH
Me
O
NC
N
m
o
7n
$^{13}C\{^1H\}$-NMR (151 MHz, methanol-d_4)
f1 (ppm)

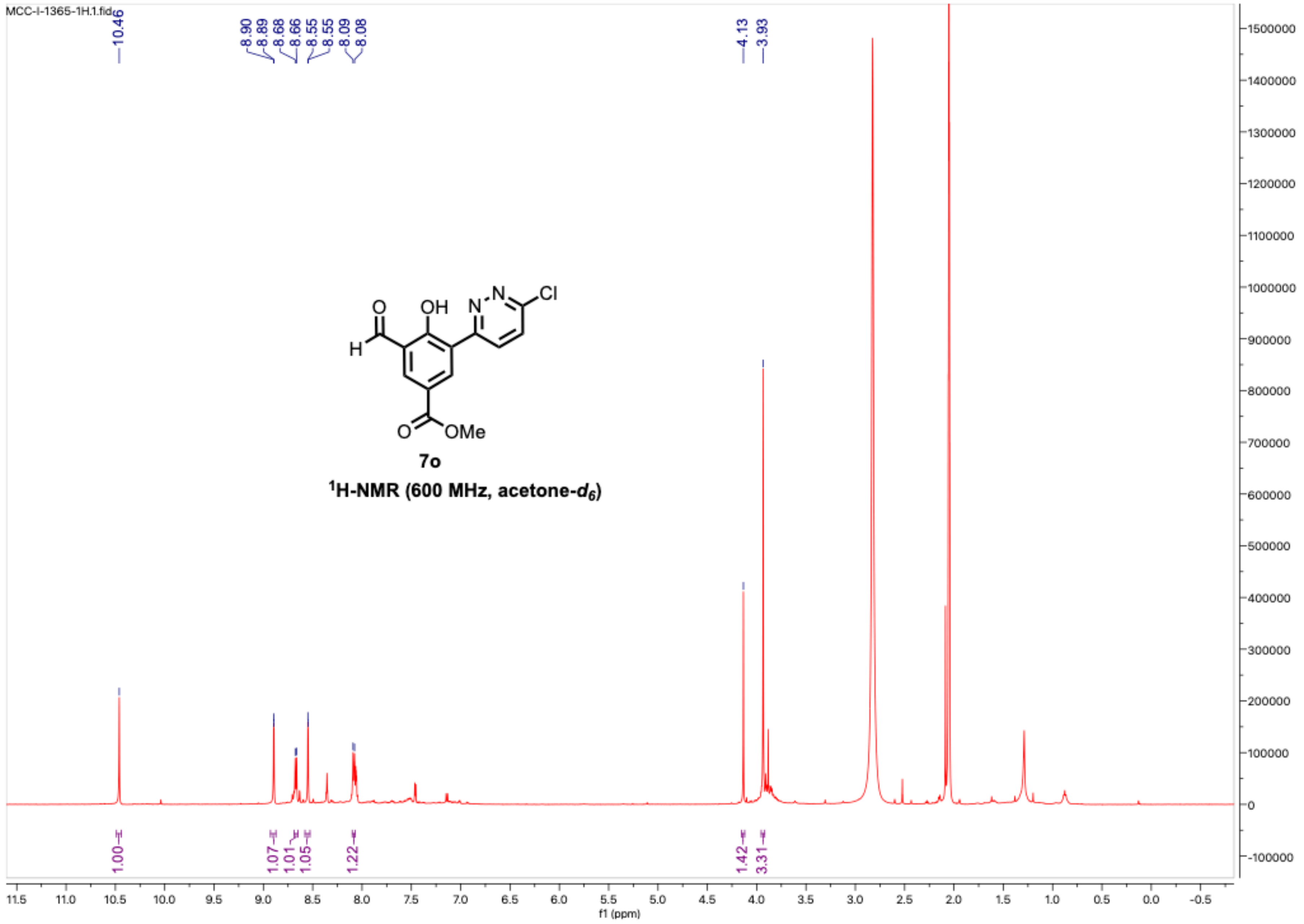

MCC-I-1365-1H.1.fid
10.46
8.90
8.89
8.68
8.66
8.55
8.55
8.09
8.08
4.13
3.93
O
OH
N
N
Cl
H
O
OMe
7o
^{1}H-NMR (600 MHz, acetone-d6)
1.00
1.07
1.01
1.05
1.22
1.42
3.31
f1 (ppm)

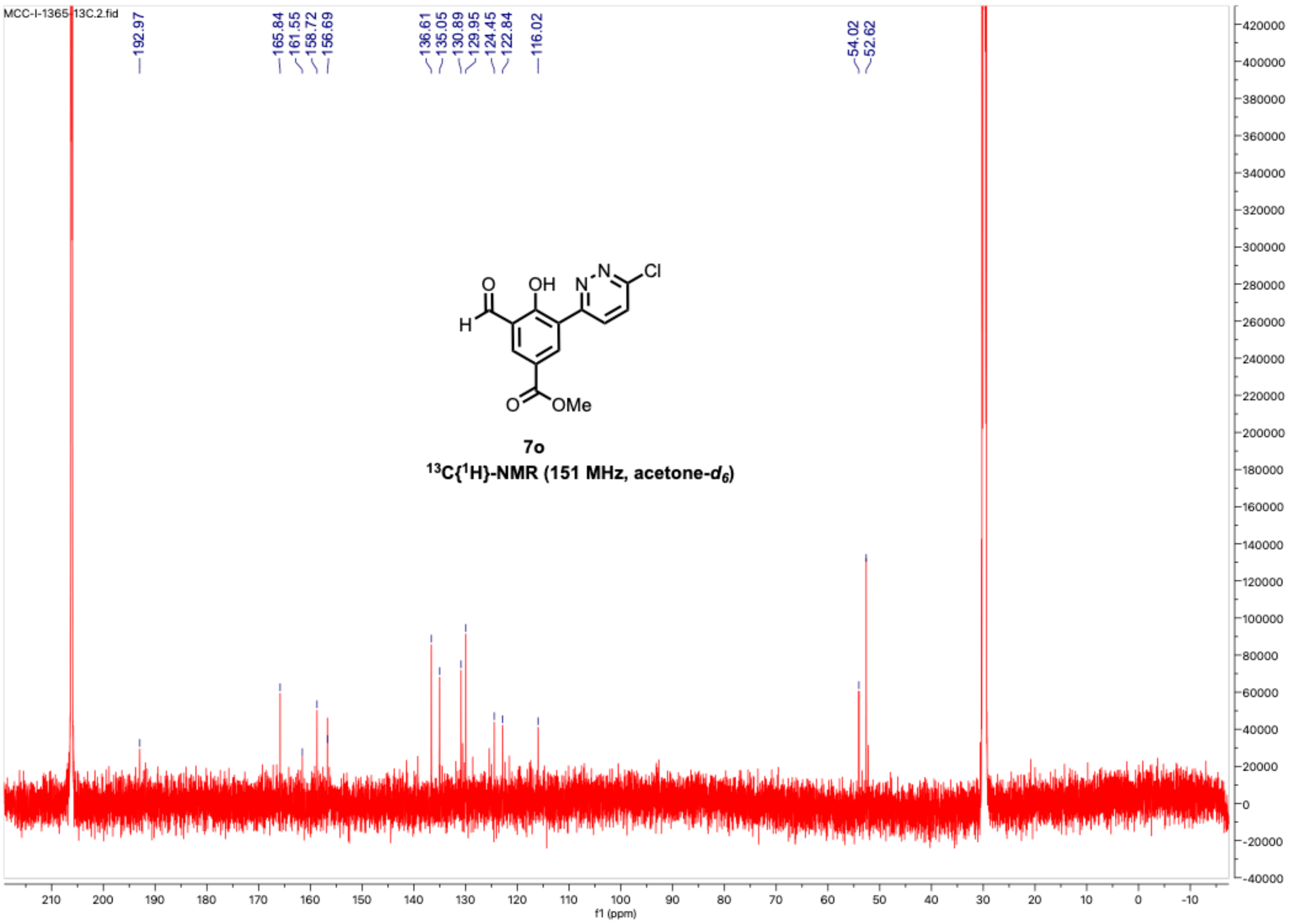
MCC-I-1365-13C.2.fid
192.97
165.84
161.55
158.72
156.69
136.61
135.05
130.89
129.95
124.45
122.84
116.02
54.02
52.62
O
OH
N
N
Cl
H
O
OMe
7o
$^{13}C\{^1H\}$-NMR (151 MHz, acetone-d_6)
f1 (ppm)

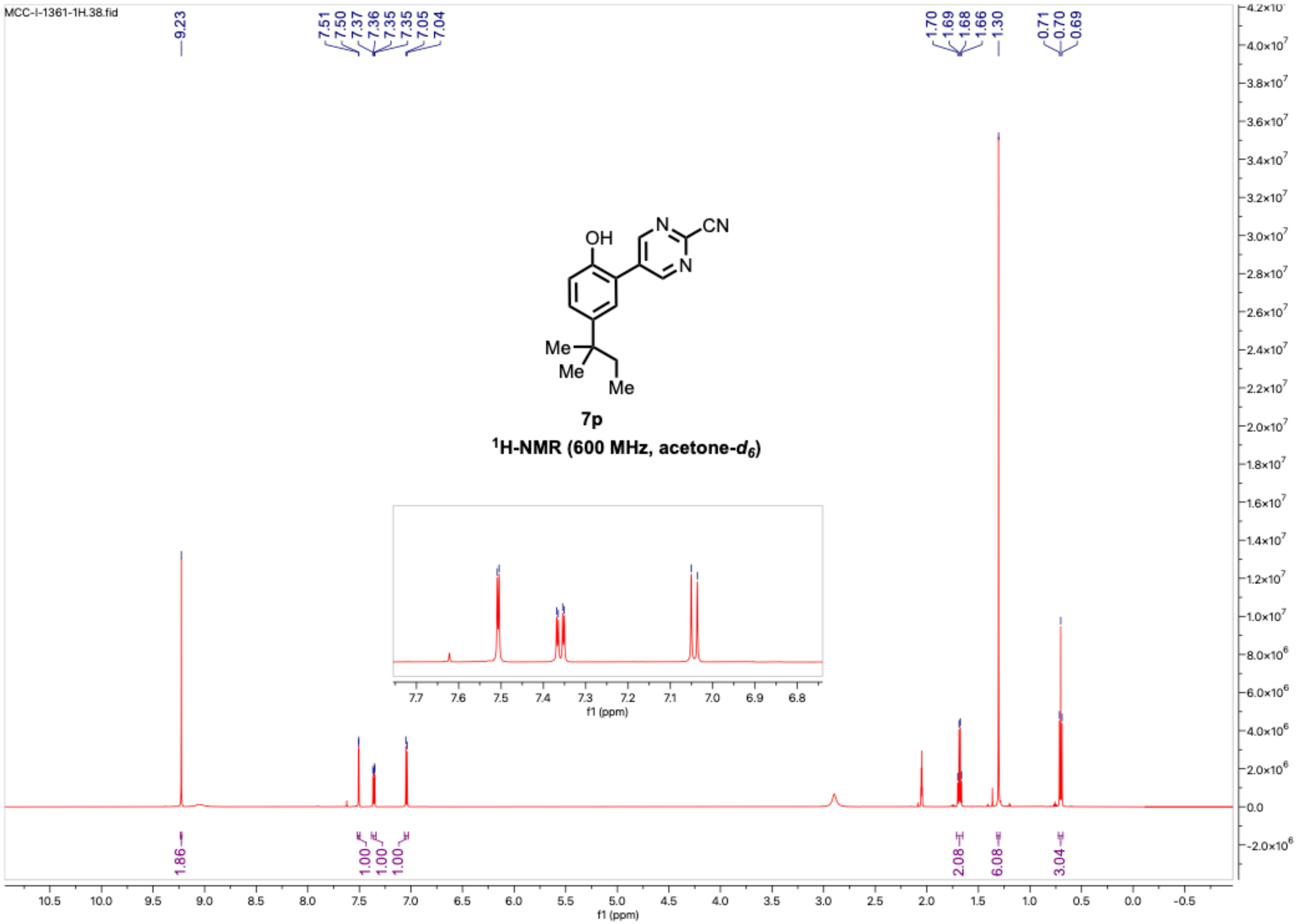
MCC-I-1361-1H.38.fid
9.23
7.51
7.50
7.37
7.36
7.35
7.35
7.05
7.04
1.70
1.69
1.68
1.66
1.30
0.71
0.70
0.69
OH
N
CN
N
Me
Me
Me
7p
1H-NMR (600 MHz, acetone-d6)
1.86
1.00
1.00
1.00
2.08
6.08
3.04
f1 (ppm)
f1 (ppm)

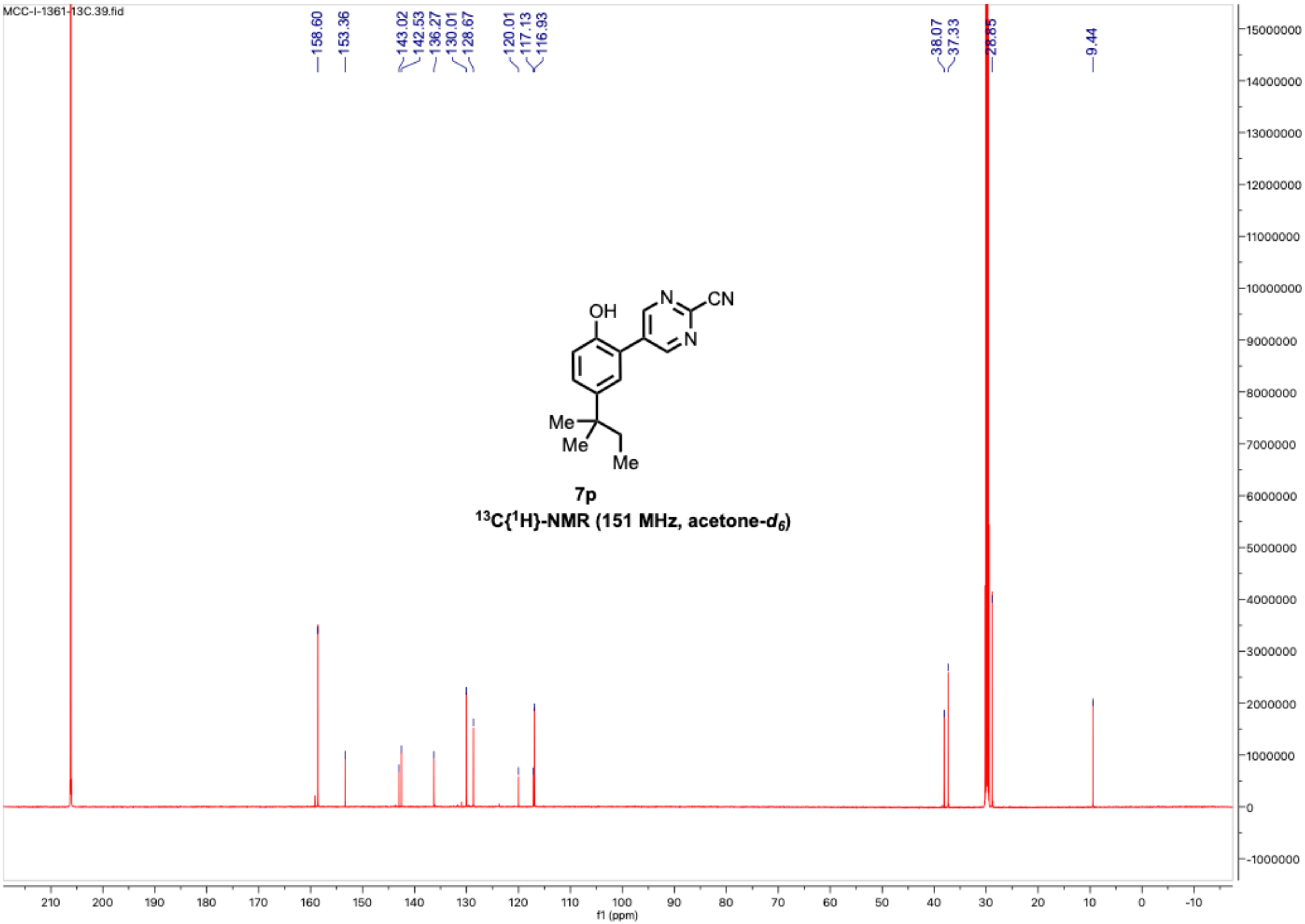

7p

$^{13}C\{^{1}H\}$-NMR (151 MHz, acetone-d_6)

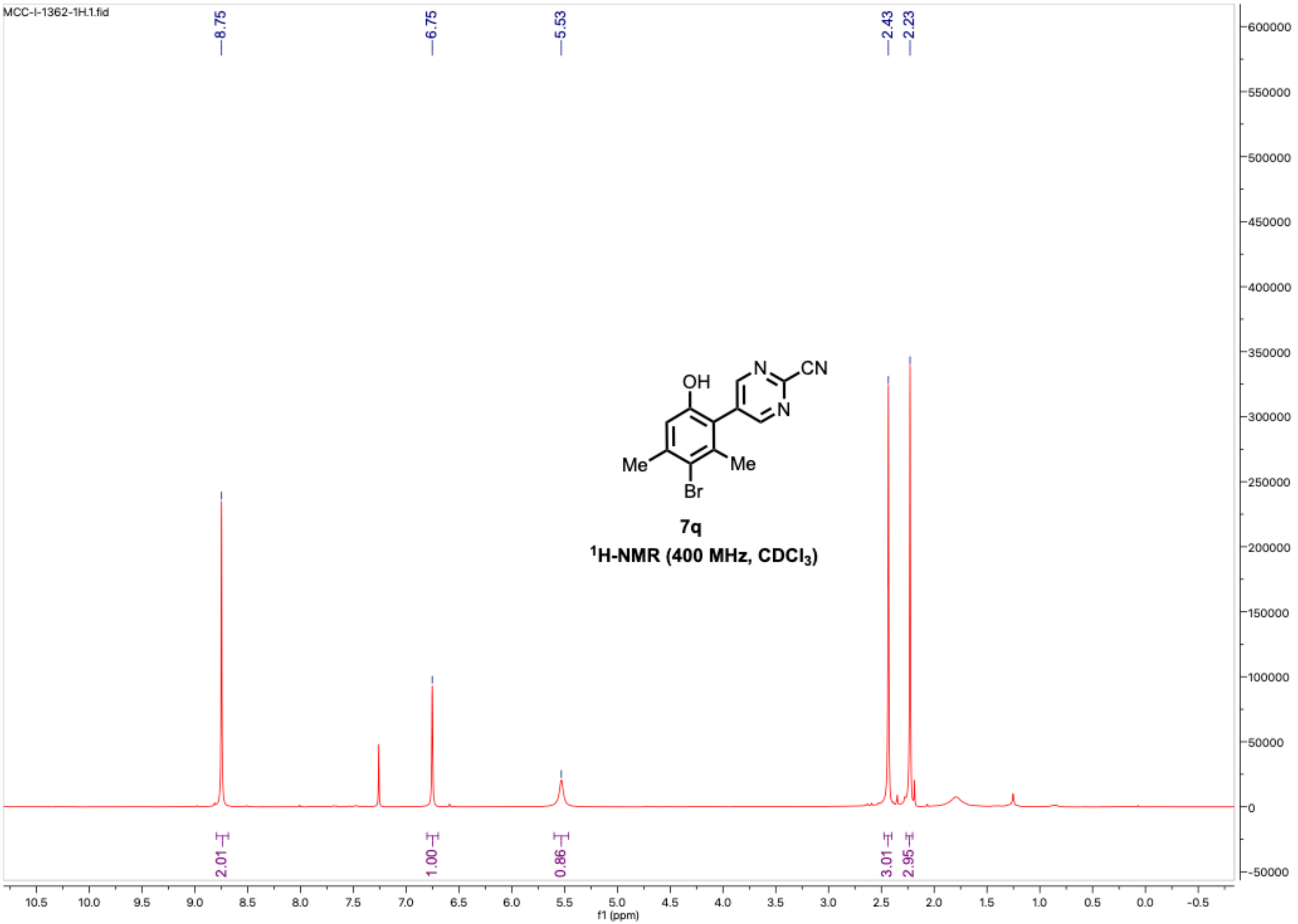
MCC-I-1362-1H.1.fid
8.75
6.75
5.53
2.43
2.23
OH
N
CN
N
Me
Me
Br
7q
1H-NMR (400 MHz, CDCl3)
2.01
1.00
0.86
3.01
2.95
f1 (ppm)

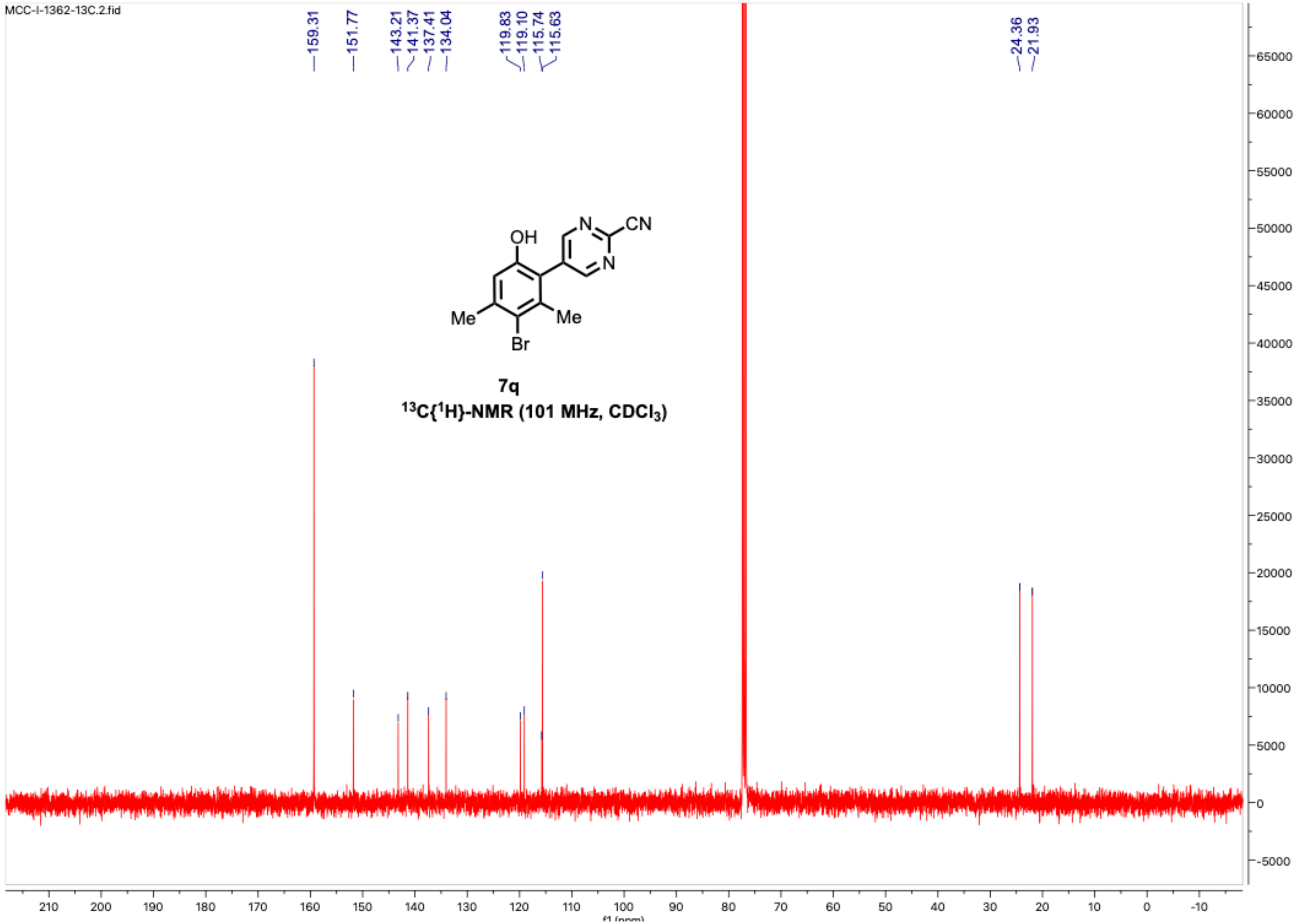

MCC-I-1362-13C.2.fid
159.31
151.77
143.21
141.37
137.41
134.04
119.83
119.10
115.74
115.63
24.36
21.93
OH
N
CN
N
Me
Me
Br
7q
$^{13}C\{^{1}H\}$-NMR (101 MHz, $CDCl_3$)
f1 (ppm)

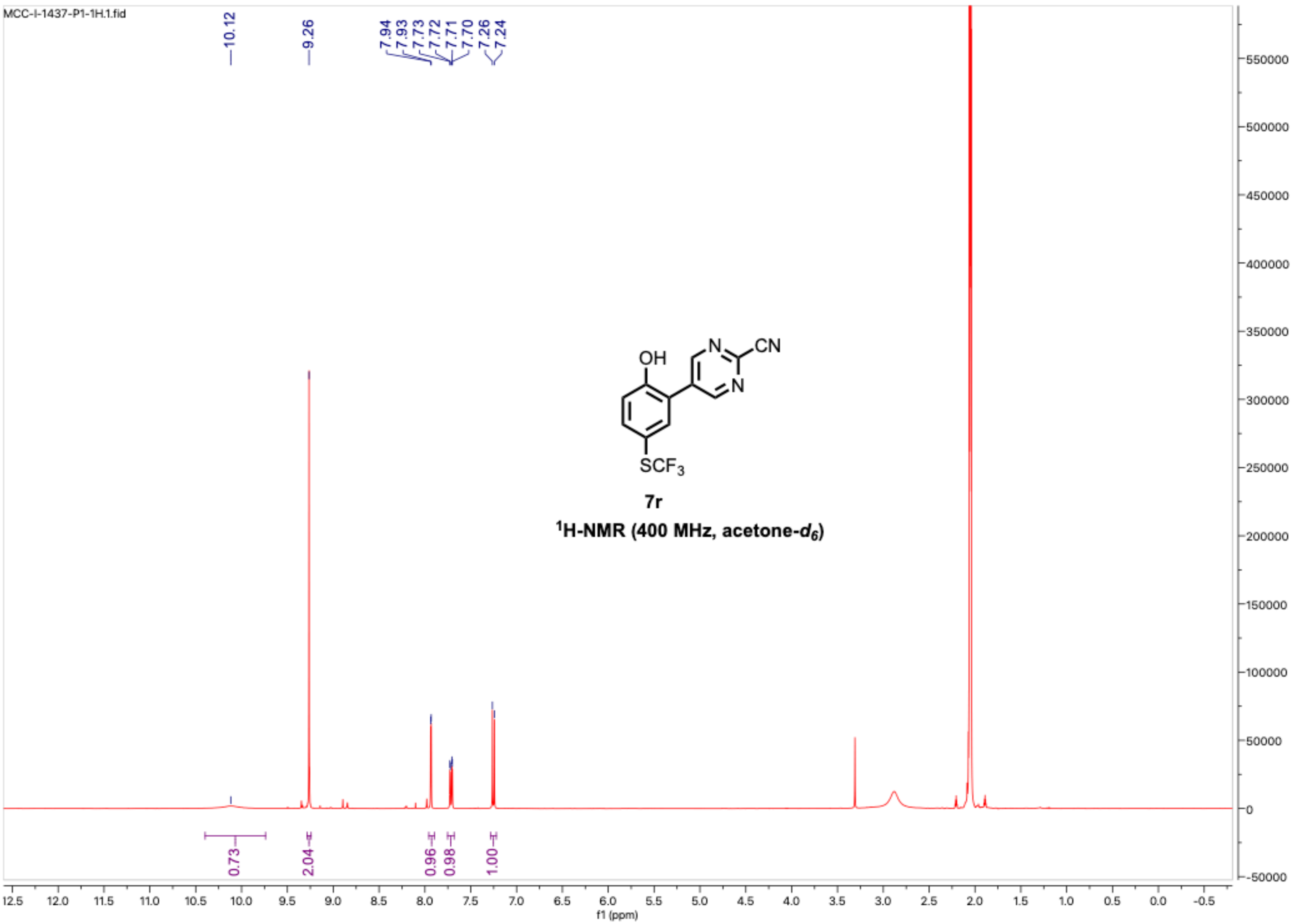
MCC-I-1437-P1-1H.1.fid
10.12
9.26
7.94
7.93
7.73
7.72
7.71
7.70
7.26
7.24
OH
N
CN
N
SCF3
7r
1H-NMR (400 MHz, acetone-d6)
0.73
2.04
0.96
0.98
1.00
f1 (ppm)

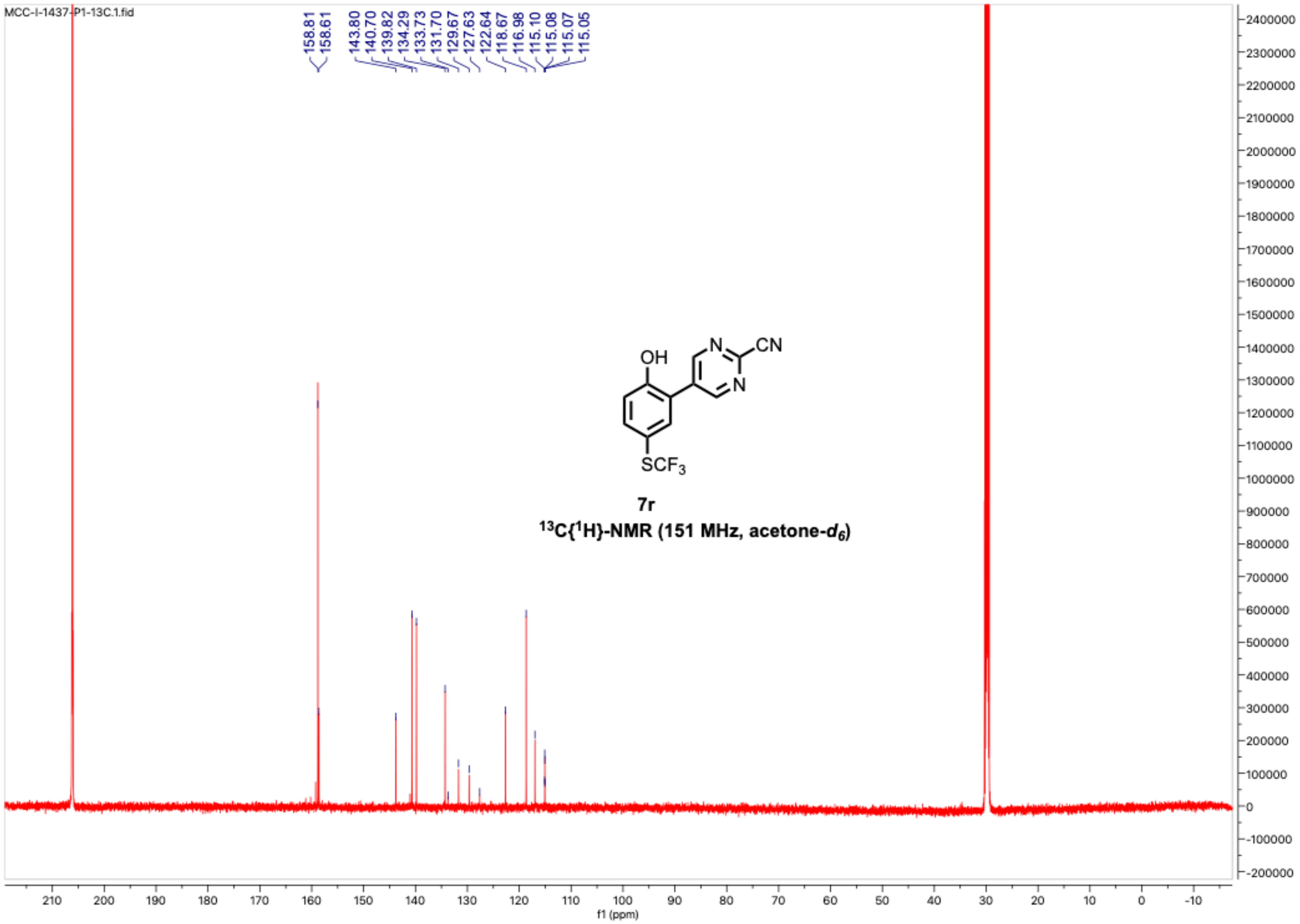

7r

$^{13}C\{^{1}H\}$-NMR (151 MHz, acetone-d_6)

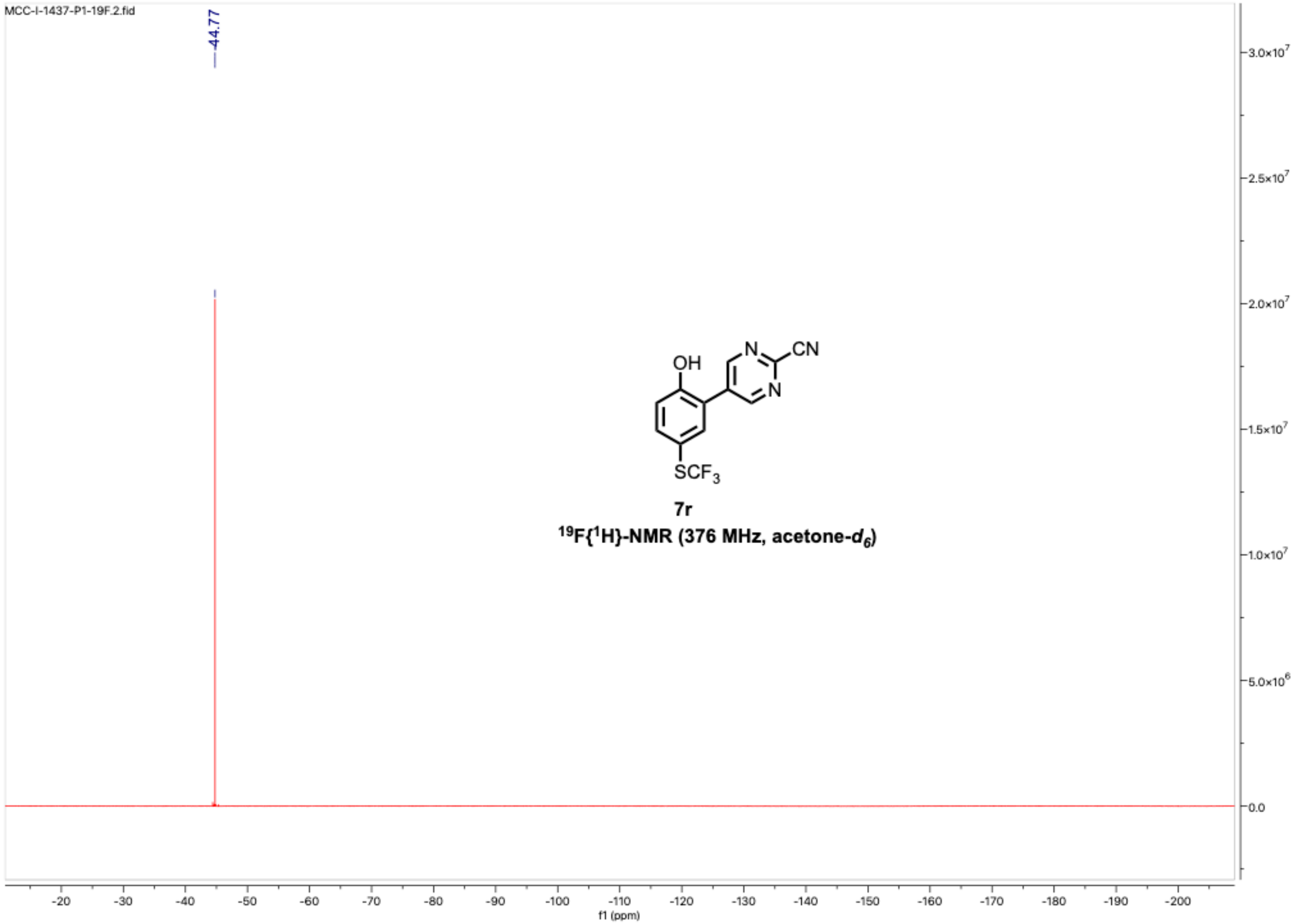
MCC-I-1437-P1-19F.2.fid
-44.77
7r
$^{19}F\{^{1}H\}$-NMR (376 MHz, acetone-d_6)
f1 (ppm)

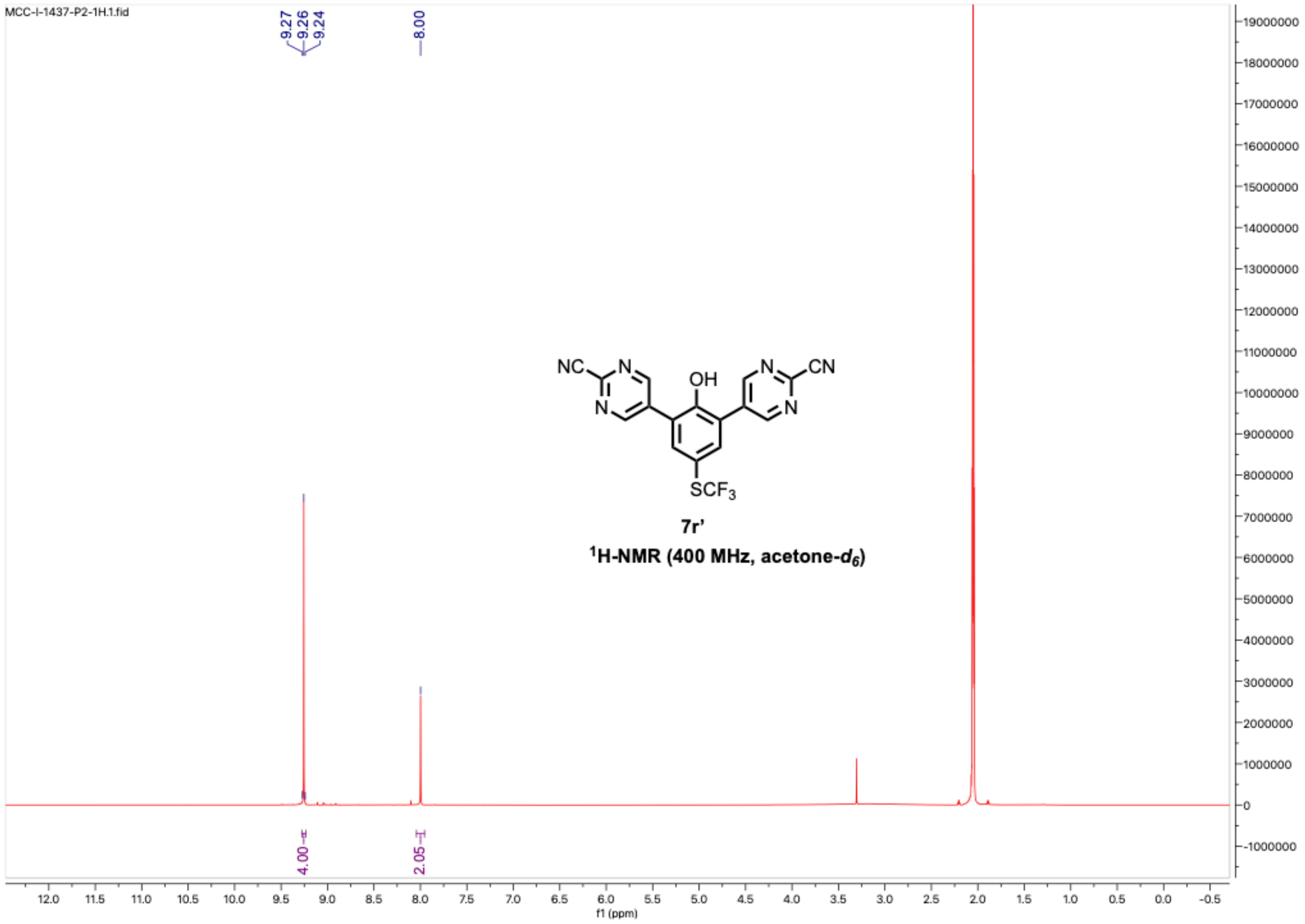
MCC-I-1437-P2-1H.1.fid
9.27
9.26
9.24
8.00
NC
N
N
OH
N
CN
N
SCF3
7r’
1H-NMR (400 MHz, acetone-d6)
4.00
2.05
f1 (ppm)

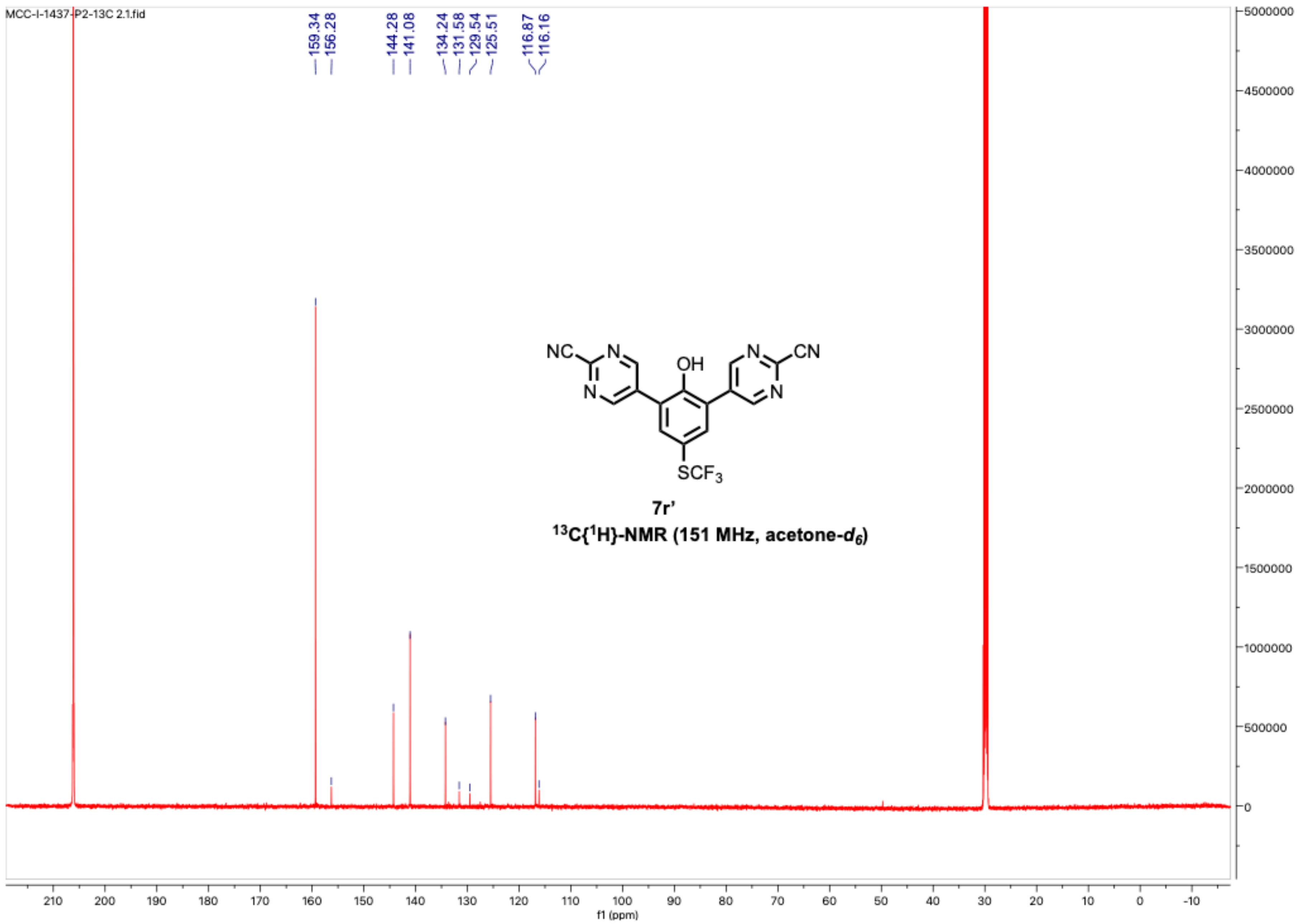
MCC-I-1437-P2-13C 2.1.fid
159.34
156.28
144.28
141.08
134.24
131.58
129.54
125.51
116.87
116.16
NC
N
N
OH
N
N
CN
SCF3
7r'
13C{1H}-NMR (151 MHz, acetone-d6)
f1 (ppm)

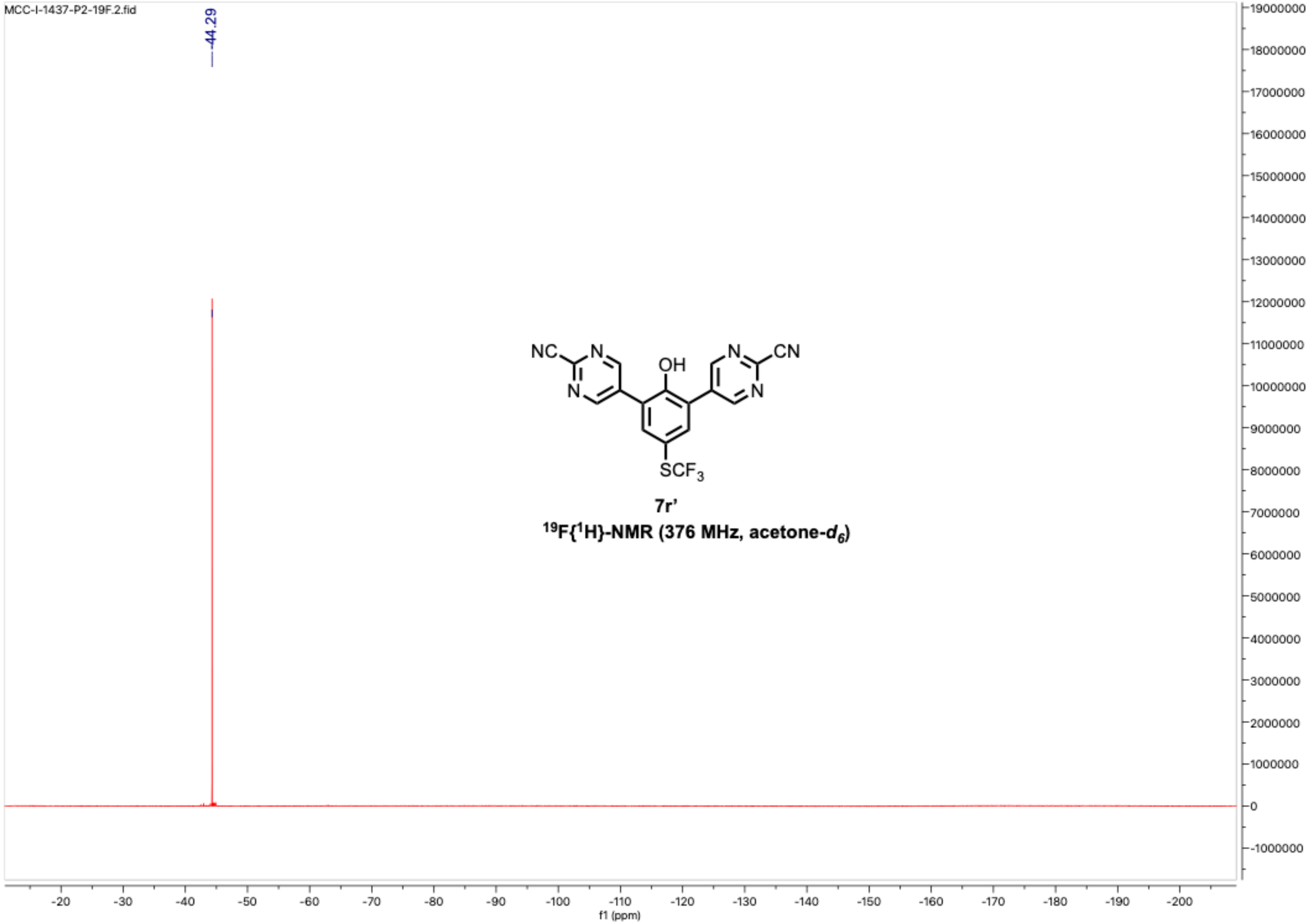
MCC-I-1437-P2-19F.2.fid
-44.29
NC
N
N
OH
N
N
CN
SCF3
7r’
19F{1H}-NMR (376 MHz, acetone-d6)
f1 (ppm)
-20 -30 -40 -50 -60 -70 -80 -90 -100 -110 -120 -130 -140 -150 -160 -170 -180 -190 -200
19000000 18000000 17000000 16000000 15000000 14000000 13000000 12000000 11000000 10000000 9000000 8000000 7000000 6000000 5000000 4000000 3000000 2000000 1000000 0 -1000000

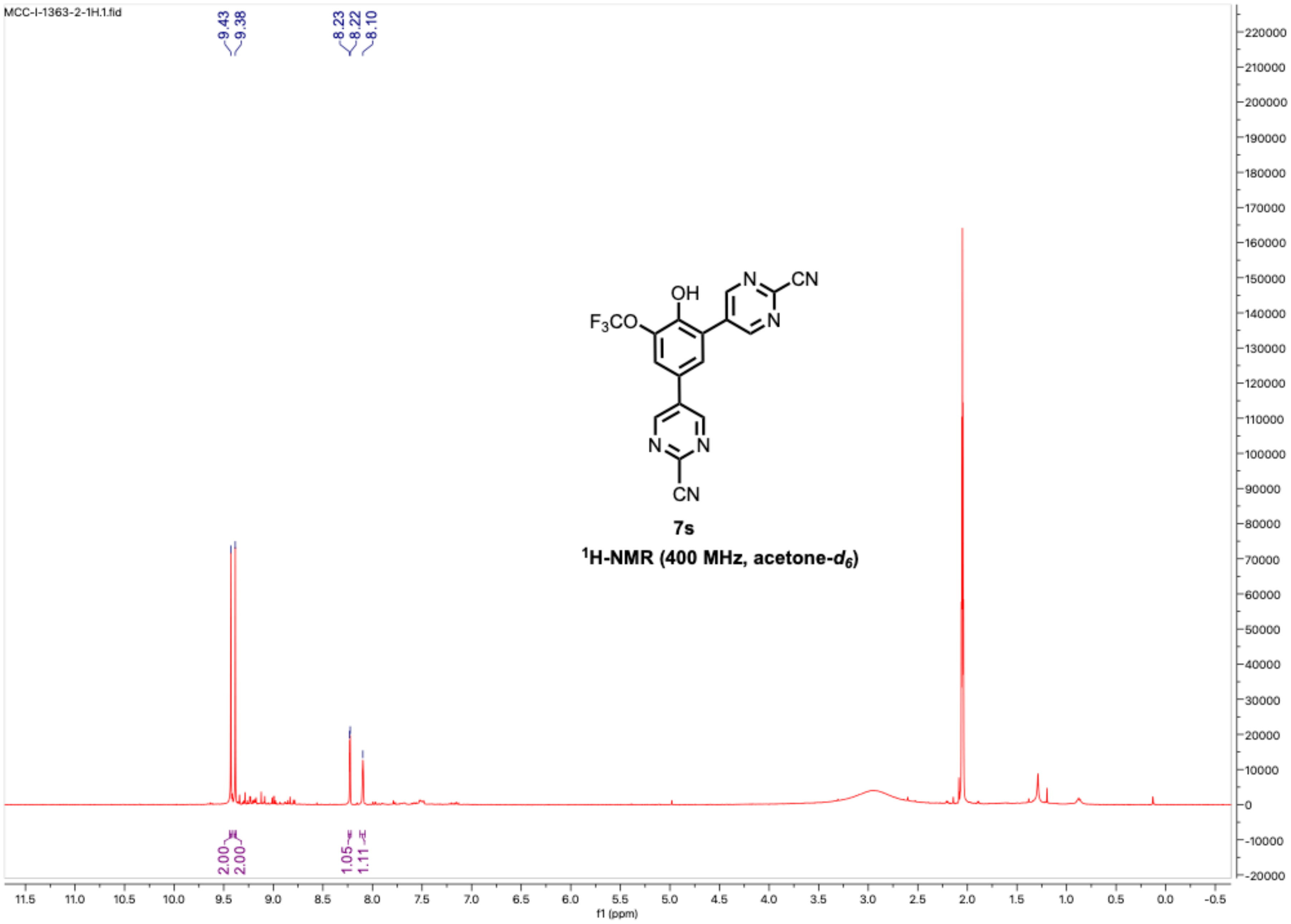
MCC-I-1363-2-1H.1.fid
9.43
9.38
8.23
8.22
8.10
OH
F3CO
N
CN
N
N
N
CN
7s
1H-NMR (400 MHz, acetone-d6)
2.00
2.00
1.05
1.11
f1 (ppm)

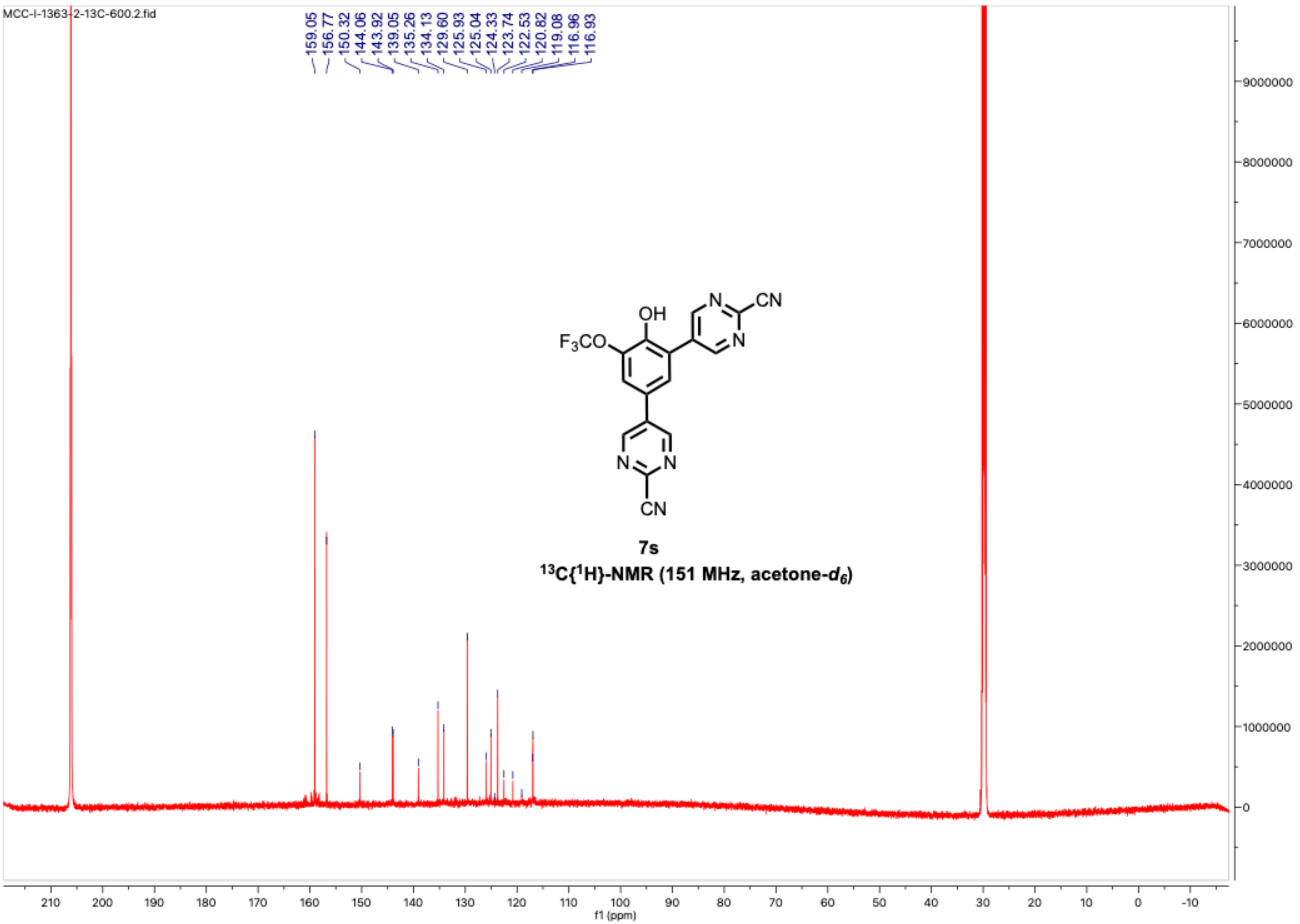
MCC-I-1363-2-13C-600.2.fid
159.05
156.77
150.32
144.06
143.92
139.05
135.26
134.13
129.60
125.93
125.04
124.33
123.74
122.53
120.82
119.08
116.96
116.93
OH
F3CO
N
CN
7s
13C{1H}-NMR (151 MHz, acetone-d6)
f1 (ppm)

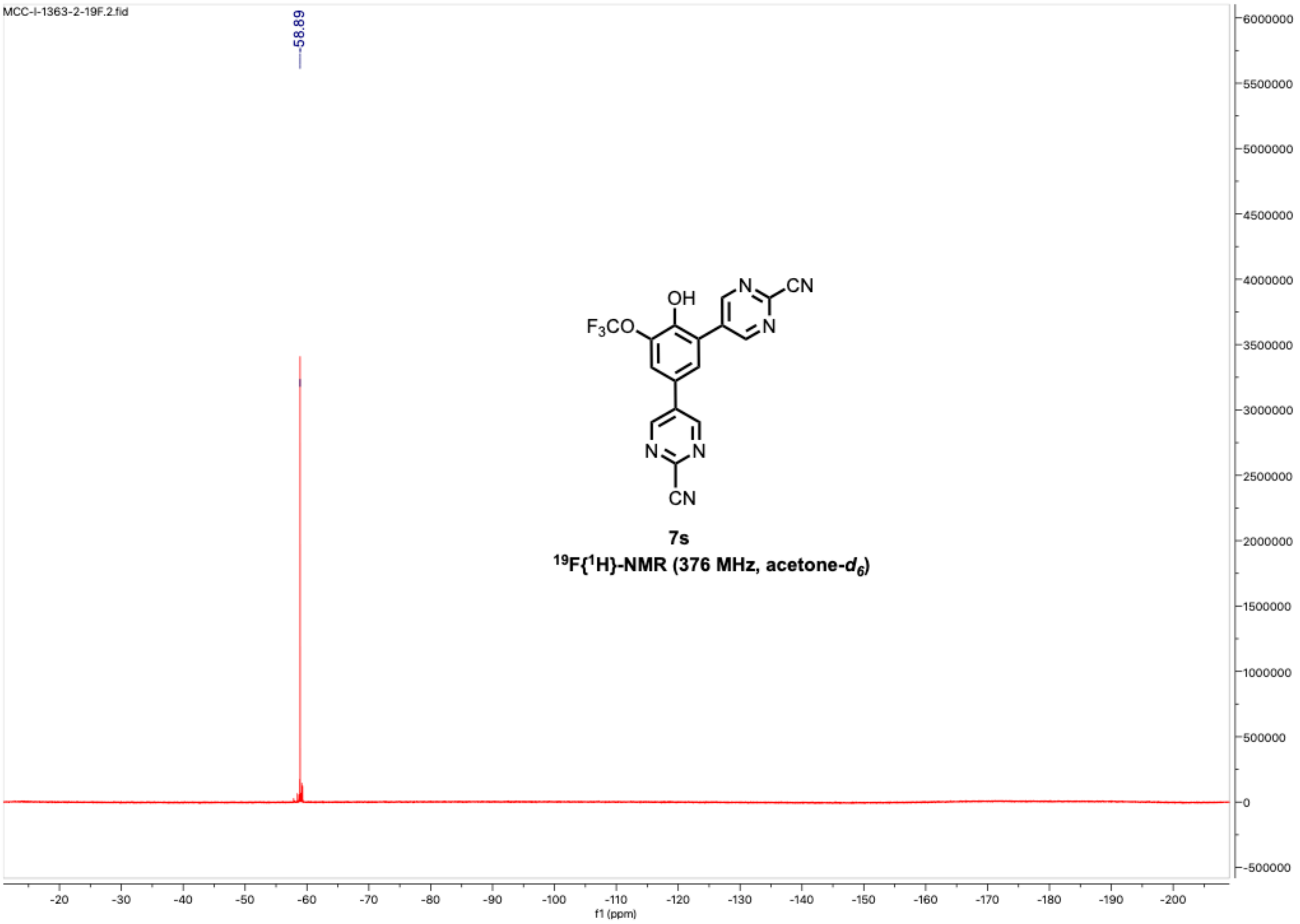
MCC-I-1363-2-19F.2.fid
-58.89
OH
N
CN
F_3CO
N
N
N
CN
7s
$^{19}F\{^1H\}$-NMR (376 MHz, acetone-d_6)
f1 (ppm)
-20
-30
-40
-50
-60
-70
-80
-90
-100
-110
-120
-130
-140
-150
-160
-170
-180
-190
-200
6000000
5500000
5000000
4500000
4000000
3500000
3000000
2500000
2000000
1500000
1000000
500000
0
-500000

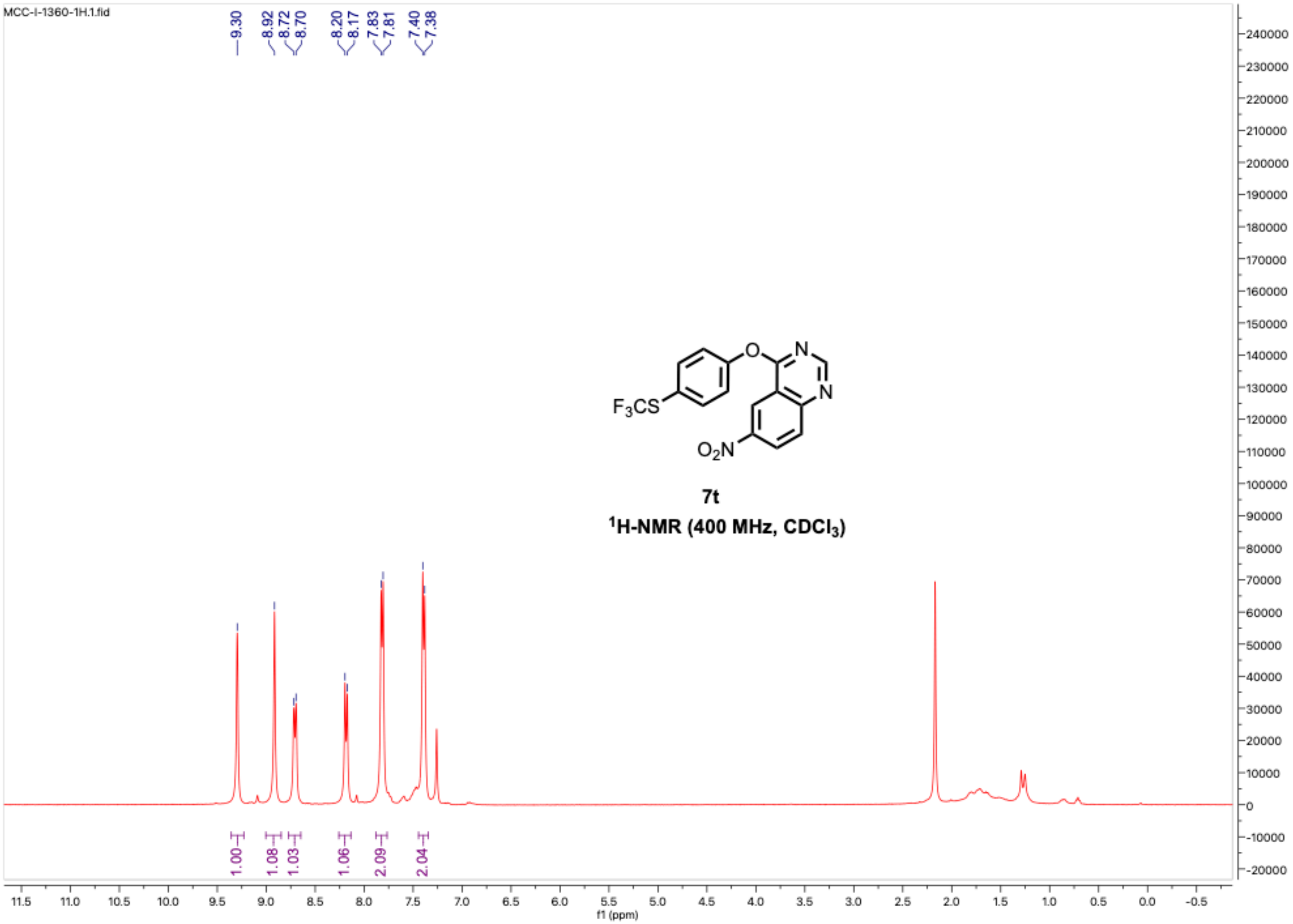

MCC-I-1360-1H.1.fid
9.30
8.92
8.72
8.70
8.20
8.17
7.83
7.81
7.40
7.38
F3CS
O
N
N
O2N
7t
1H-NMR (400 MHz, CDCl3)
1.00
1.08
1.03
1.06
2.09
2.04
f1 (ppm)

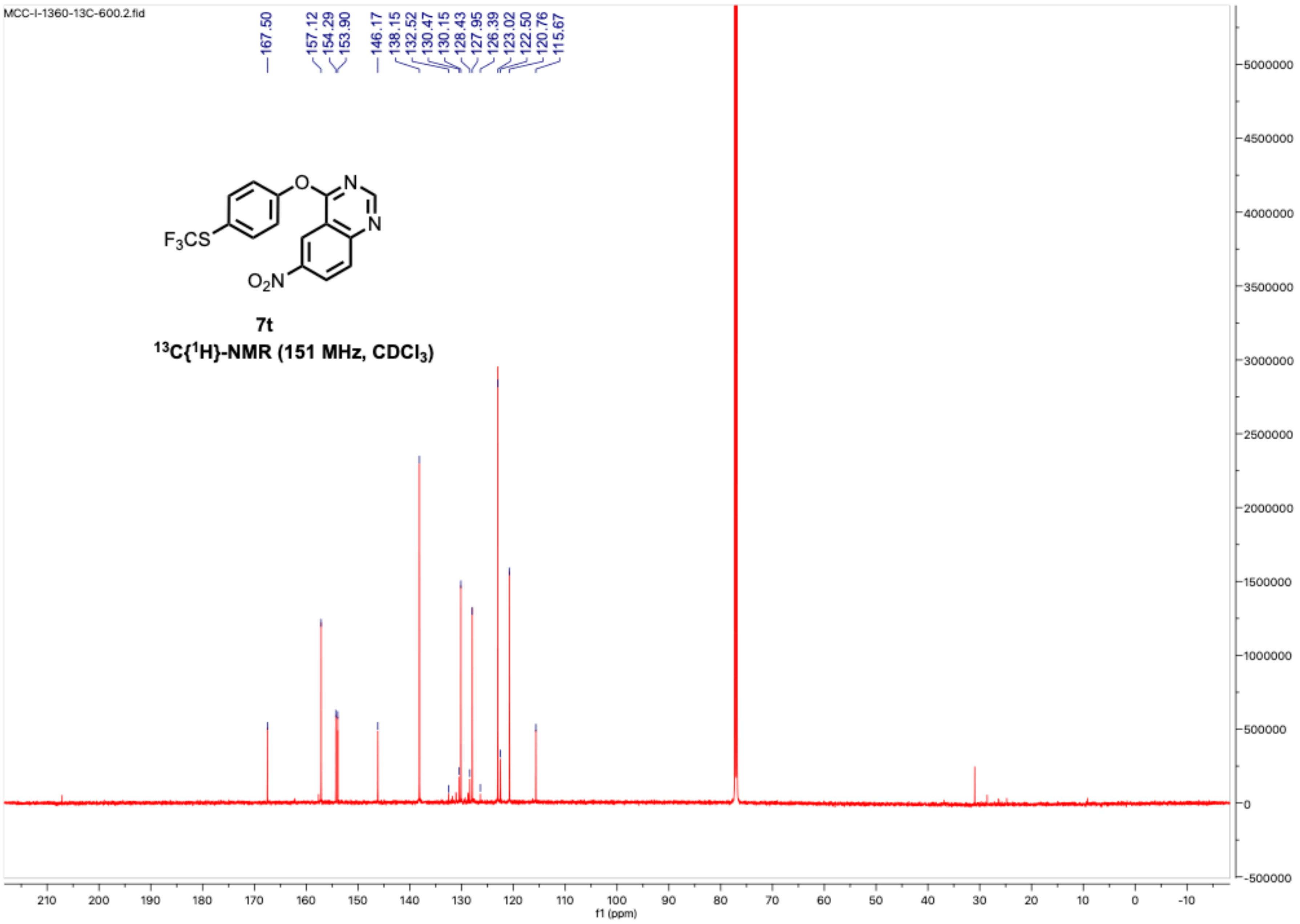
MCC-I-1360-13C-600.2.fid
167.50
157.12
154.29
153.90
146.17
138.15
132.52
130.47
130.15
128.43
127.95
126.39
123.02
122.50
120.76
115.67
F3CS
O
N
N
O2N
7t
13C{1H}-NMR (151 MHz, CDCl3)
f1 (ppm)

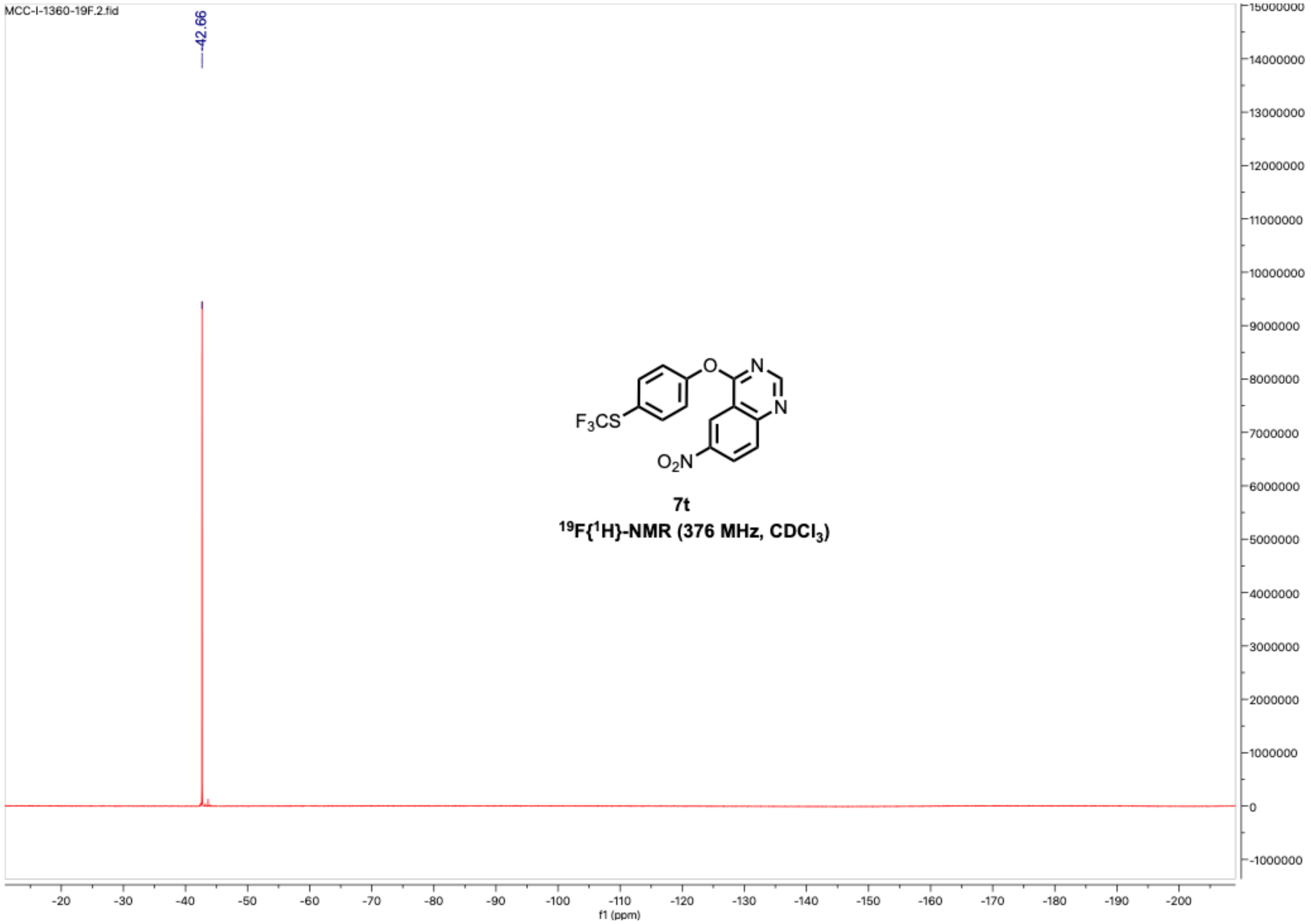
MCC-I-1360-19F.2.fid
-42.66
F3CS
O
N
N
O2N
7t
19F{1H}-NMR (376 MHz, CDCl3)
15000000
14000000
13000000
12000000
11000000
10000000
9000000
8000000
7000000
6000000
5000000
4000000
3000000
2000000
1000000
0
-1000000
-20
-30
-40
-50
-60
-70
-80
-90
-100
-110
-120
-130
-140
-150
-160
-170
-180
-190
-200
f1 (ppm)

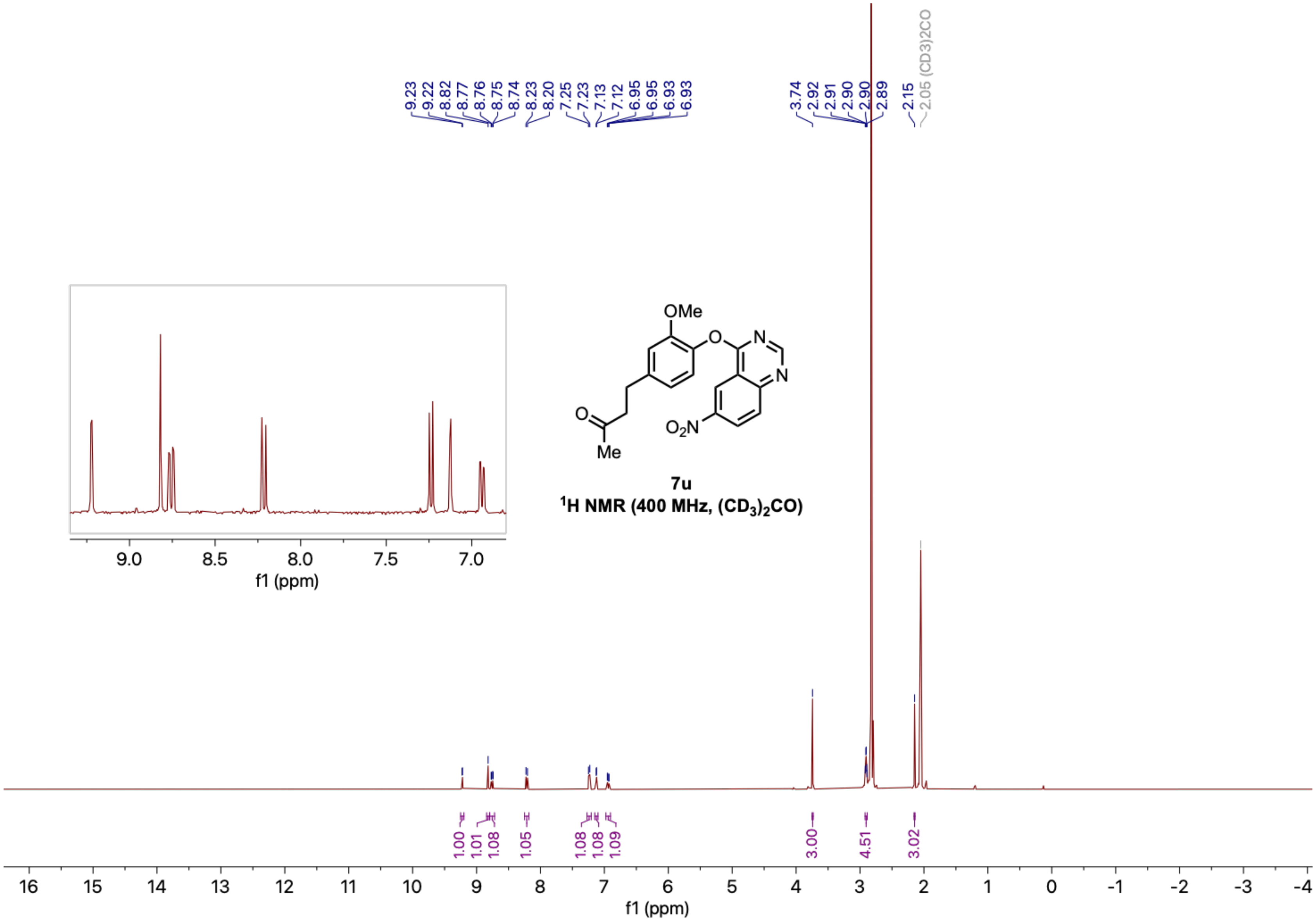
9.23
9.22
8.82
8.77
8.76
8.75
8.74
8.23
8.20
7.25
7.23
7.13
7.12
6.95
6.95
6.93
6.93
3.74
2.92
2.91
2.90
2.90
2.89
2.15
2.05 (CD3)2CO
OMe
O
N
N
O
Me
O2N
7u
1H NMR (400 MHz, (CD3)2CO)
9.0
8.5
8.0
7.5
7.0
f1 (ppm)
1.00
1.01
1.08
1.05
1.08
1.08
1.09
3.00
4.51
3.02
16 15 14 13 12 11 10 9 8 7 6 5 4 3 2 1 0 -1 -2 -3 -4
f1 (ppm)

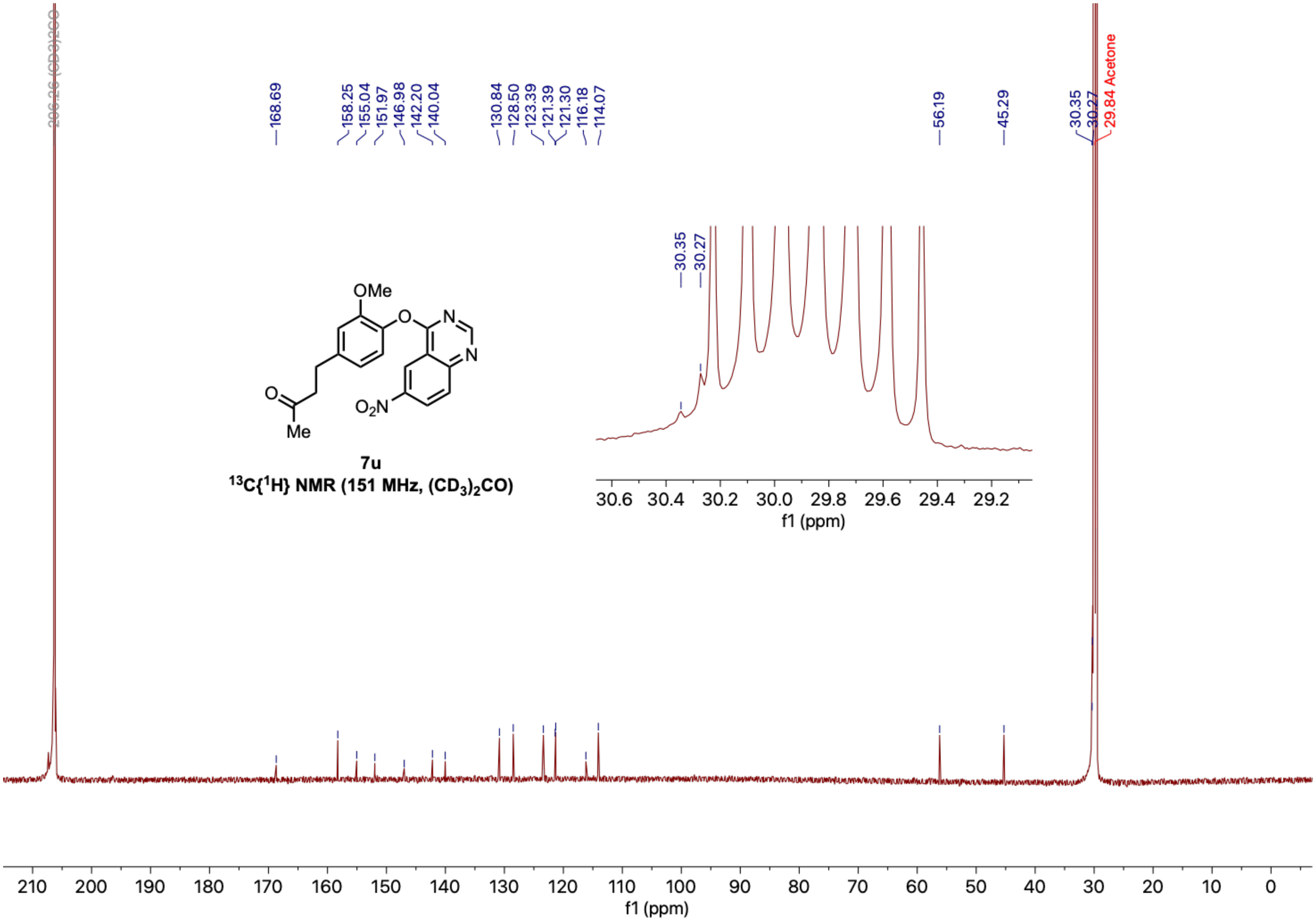

206.26 (CD3)2CO
168.69
158.25
155.04
151.97
146.98
142.20
140.04
130.84
128.50
123.39
121.39
121.30
116.18
114.07
56.19
45.29
30.35
30.27
29.84 Acetone
OMe
O
N
N
O2N
O
Me
7u
13C{1H} NMR (151 MHz, (CD3)2CO)
30.35
30.27
30.6 30.4 30.2 30.0 29.8 29.6 29.4 29.2
f1 (ppm)
210 200 190 180 170 160 150 140 130 120 110 100 90 80 70 60 50 40 30 20 10 0
f1 (ppm)

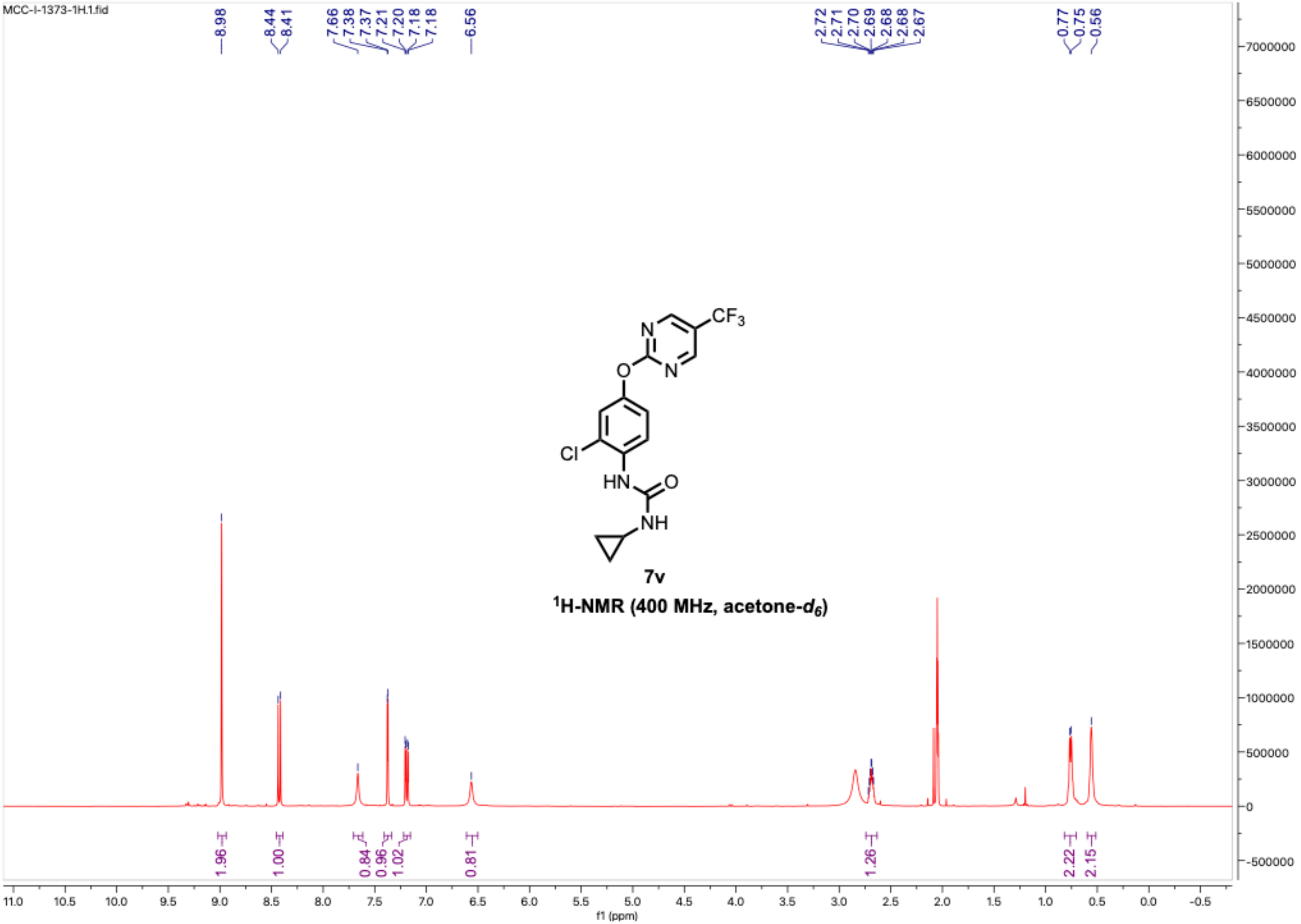

7v

^{1}H-NMR (400 MHz, acetone-d_6)

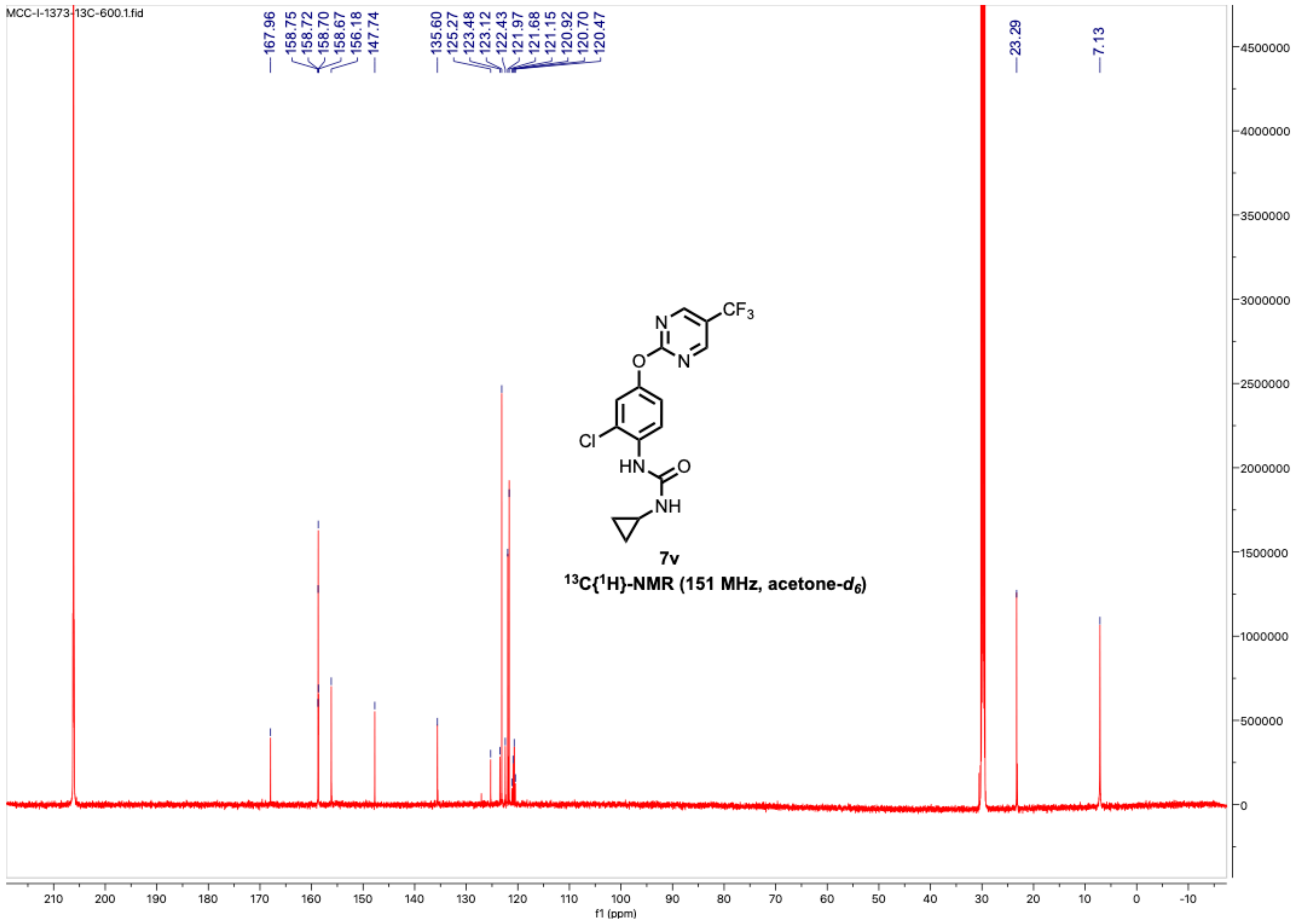

MCC-I-1373-13C-600.1.fid
167.96
158.75
158.72
158.70
158.67
156.18
147.74
135.60
125.27
123.48
123.12
122.43
121.97
121.68
121.15
120.92
120.70
120.47
23.29
7.13
CF3
N
N
O
Cl
HN
O
NH
7v
13C{1H}-NMR (151 MHz, acetone-d6)
f1 (ppm)

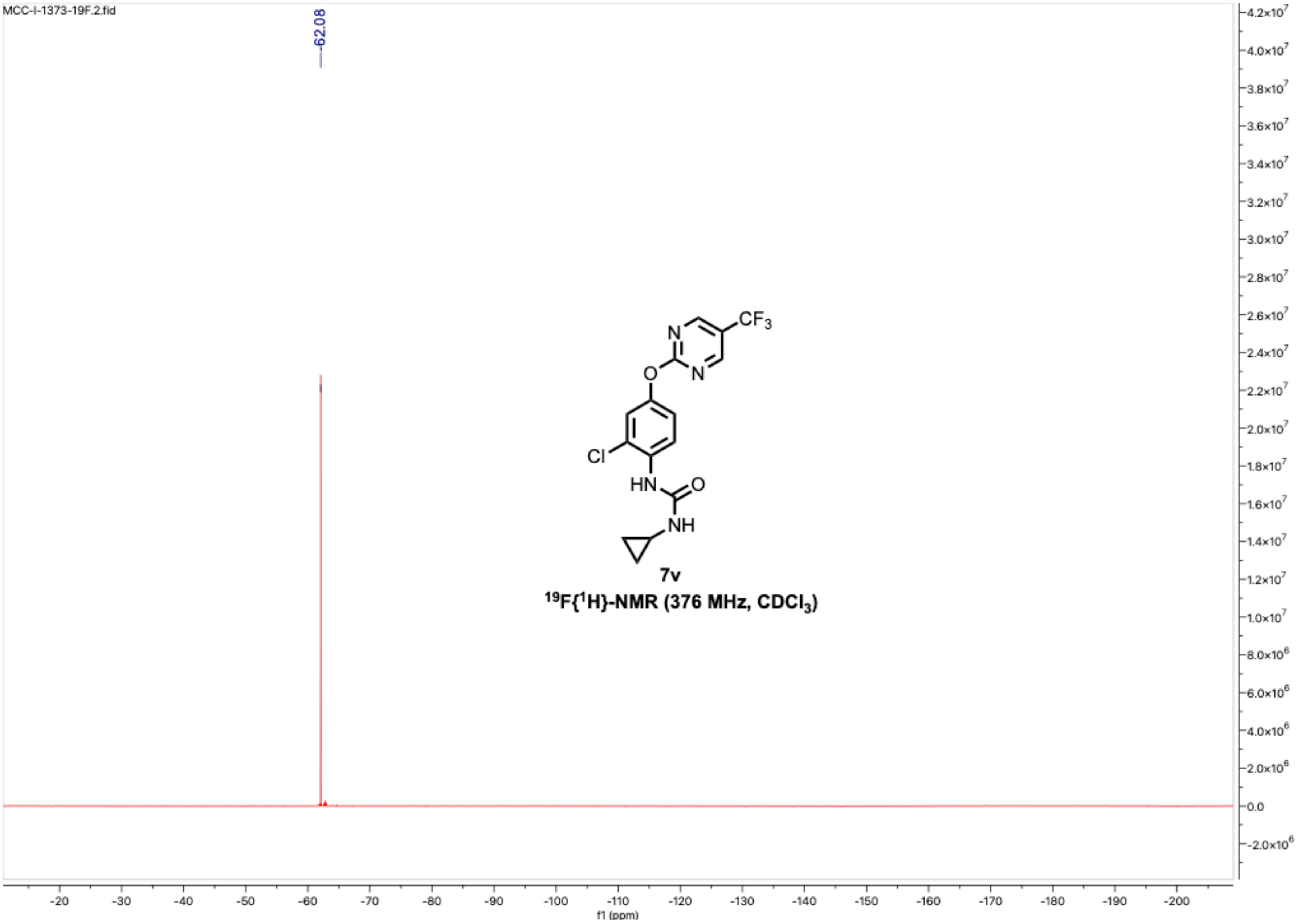

7v

$^{19}F\{^1H\}$-NMR (376 MHz, $CDCl_3$)

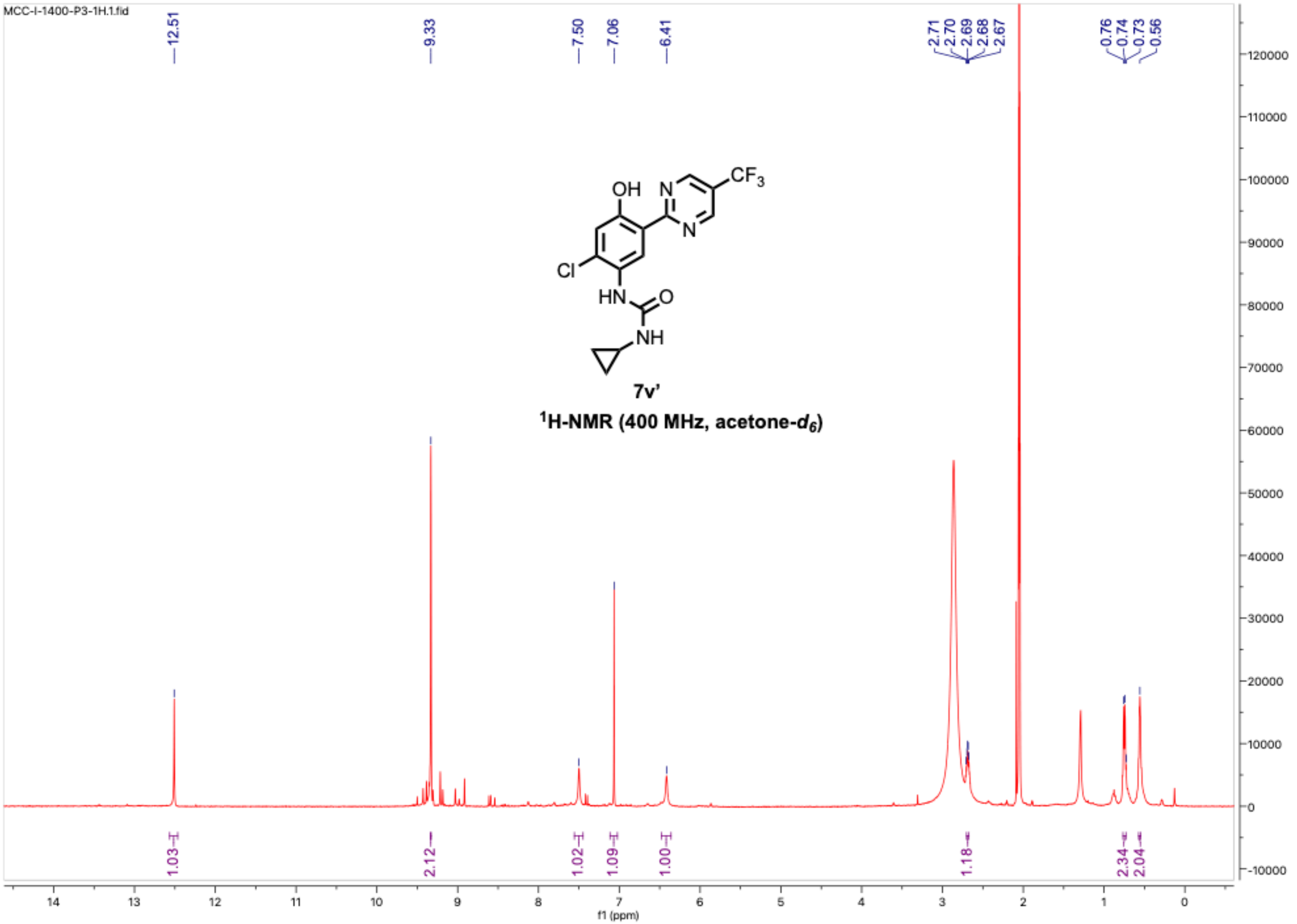

7v'

^{1}H-NMR (400 MHz, acetone-d_6)

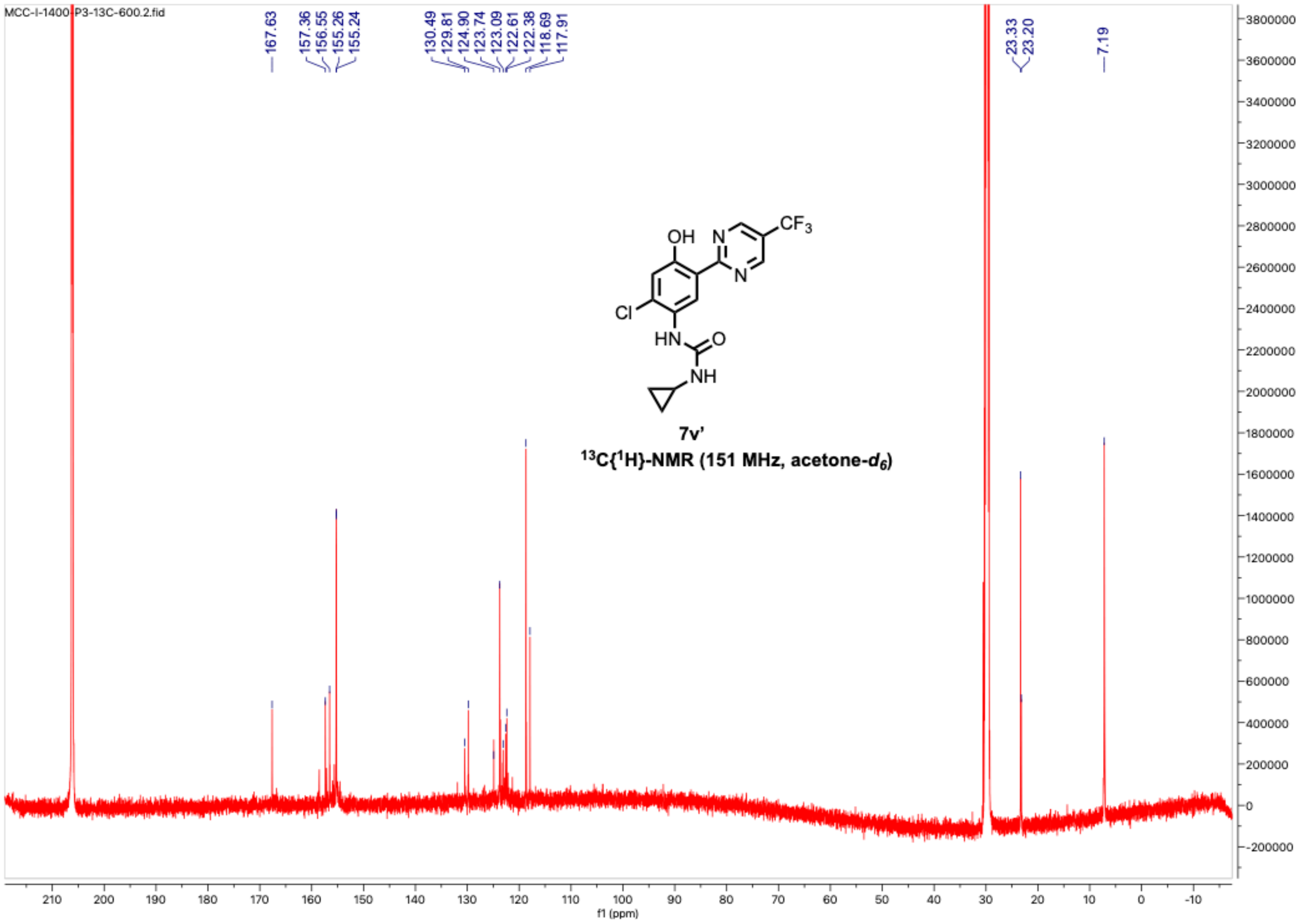
MCC-I-1400-P3-13C-600.2.fid
167.63
157.36
156.55
155.26
155.24
130.49
129.81
124.90
123.74
123.09
122.61
122.38
118.69
117.91
23.33
23.20
7.19
OH
N
N
CF3
Cl
HN
O
NH
7v'
13C{1H}-NMR (151 MHz, acetone-d6)
f1 (ppm)

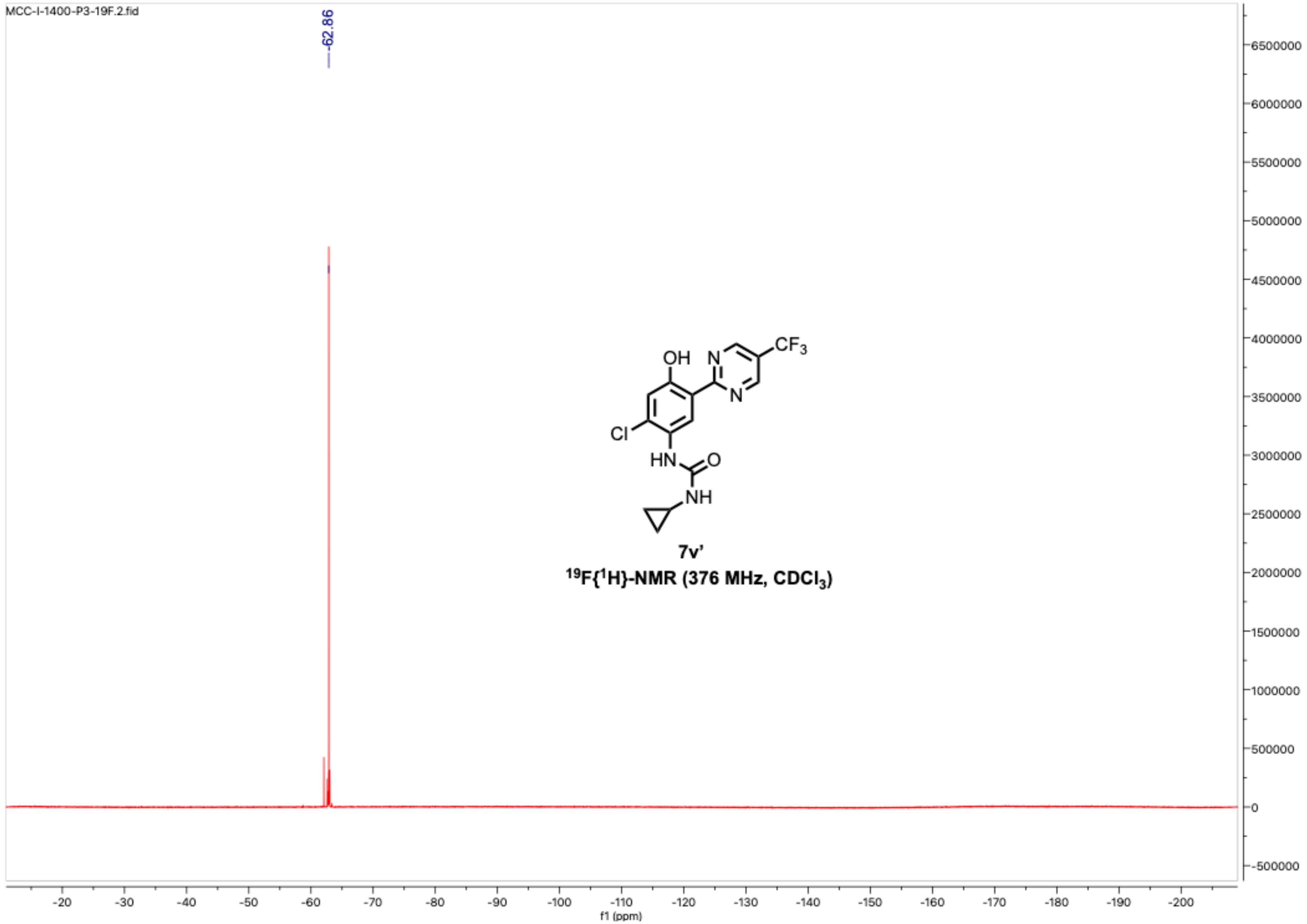

7v'

^{19}F{^{1}H}-NMR (376 MHz, $CDCl_3$)

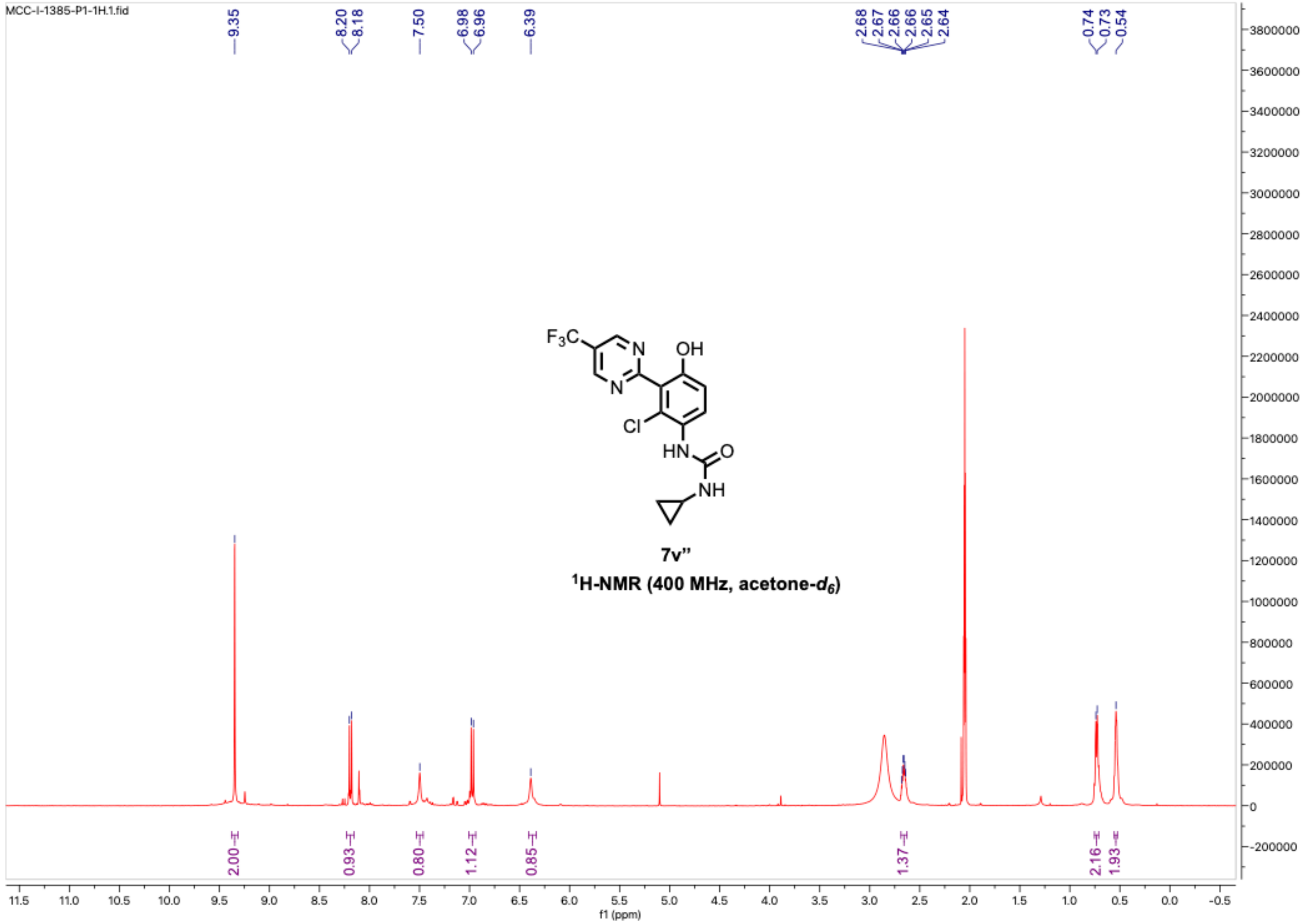
MCC-I-1385-P1-1H.1.fid
9.35
8.20
8.18
7.50
6.98
6.96
6.39
2.68
2.67
2.66
2.66
2.65
2.64
0.74
0.73
0.54
F3C
N
N
OH
Cl
HN
O
NH
7v''
1H-NMR (400 MHz, acetone-d6)
2.00
0.93
0.80
1.12
0.85
1.37
2.16
1.93
f1 (ppm)

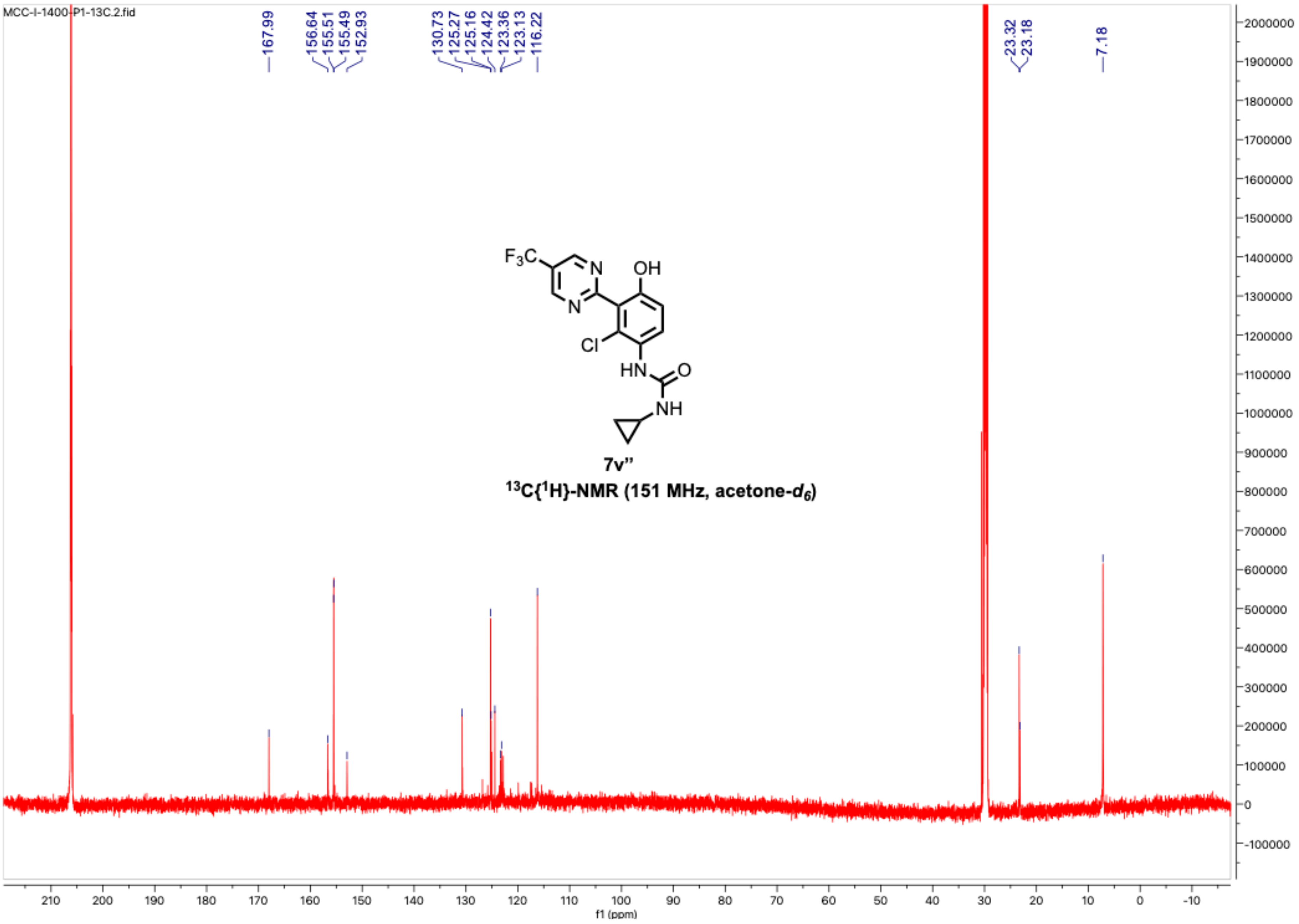

MCC-I-1400-P1-13C.2.fid
167.99
156.64
155.51
155.49
152.93
130.73
125.27
125.16
124.42
123.36
123.13
116.22
23.32
23.18
7.18
F_3C
N
N
OH
Cl
HN
O
NH
7v''
$^{13}C\{^{1}H\}$-NMR (151 MHz, acetone-d_6)
f1 (ppm)

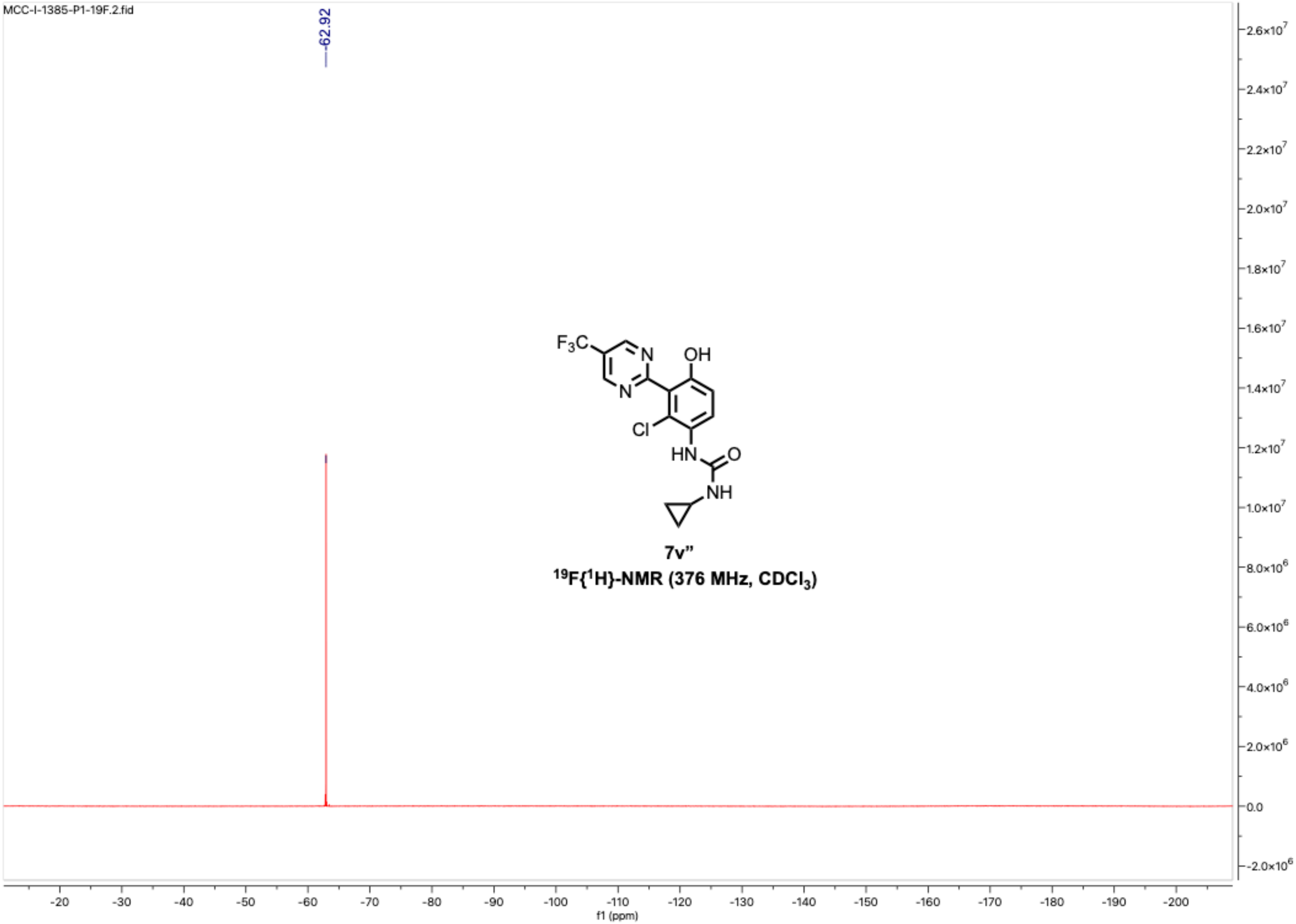
MCC-I-1385-P1-19F.2.fid
-62.92
F3C
N
N
OH
Cl
HN
O
NH
7v”
19F{1H}-NMR (376 MHz, CDCl3)
f1 (ppm)

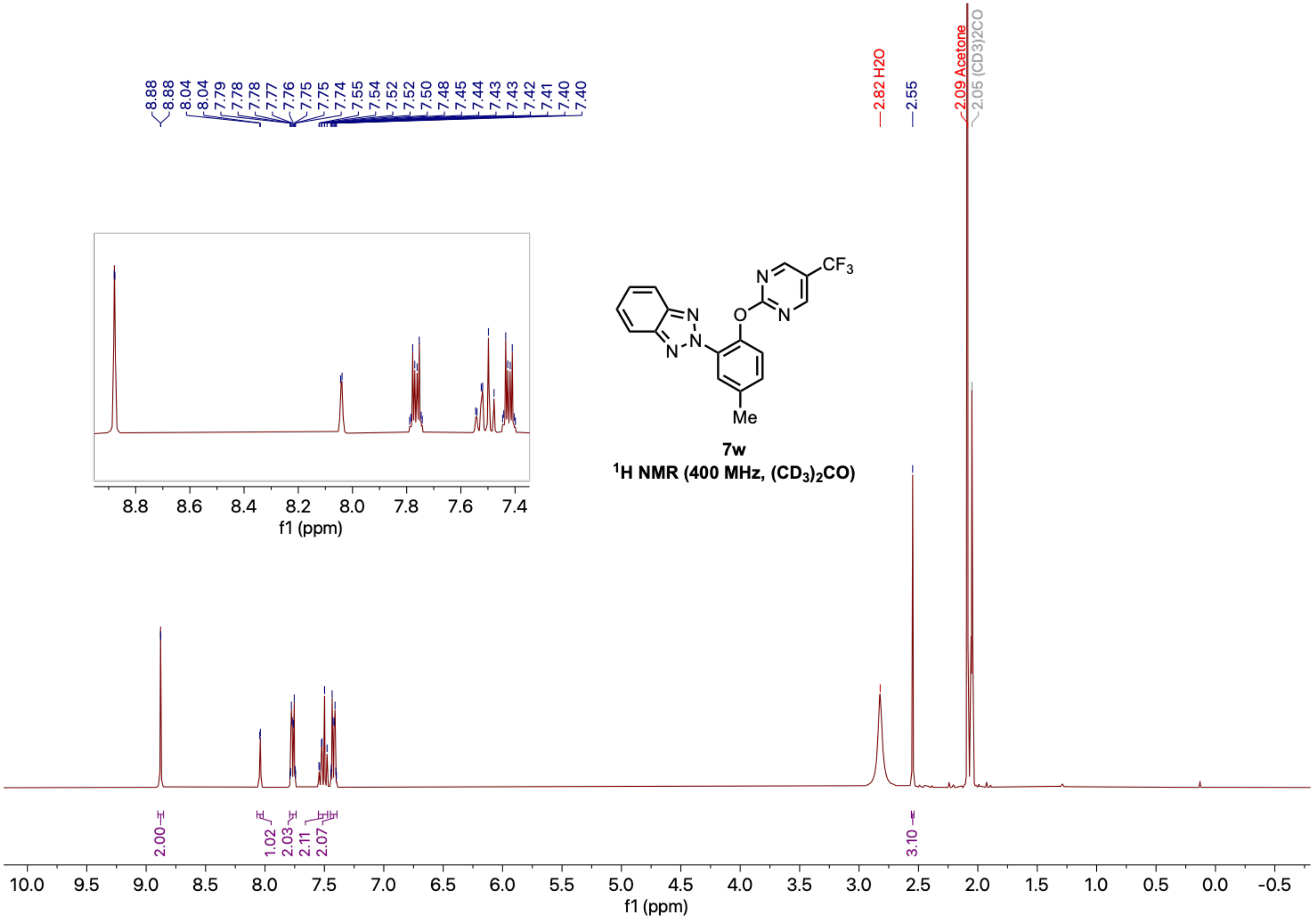
8.88
8.88
8.04
7.79
7.78
7.78
7.77
7.76
7.75
7.75
7.74
7.55
7.54
7.52
7.52
7.50
7.48
7.45
7.44
7.43
7.43
7.42
7.41
7.40
7.40
2.82 H2O
2.55
2.09 Acetone
2.05 (CD3)2CO
CF3
N
N
O
N
N
N
Me
7w
1H NMR (400 MHz, (CD3)2CO)
2.00
1.02
2.03
2.11
2.07
3.10
f1 (ppm)

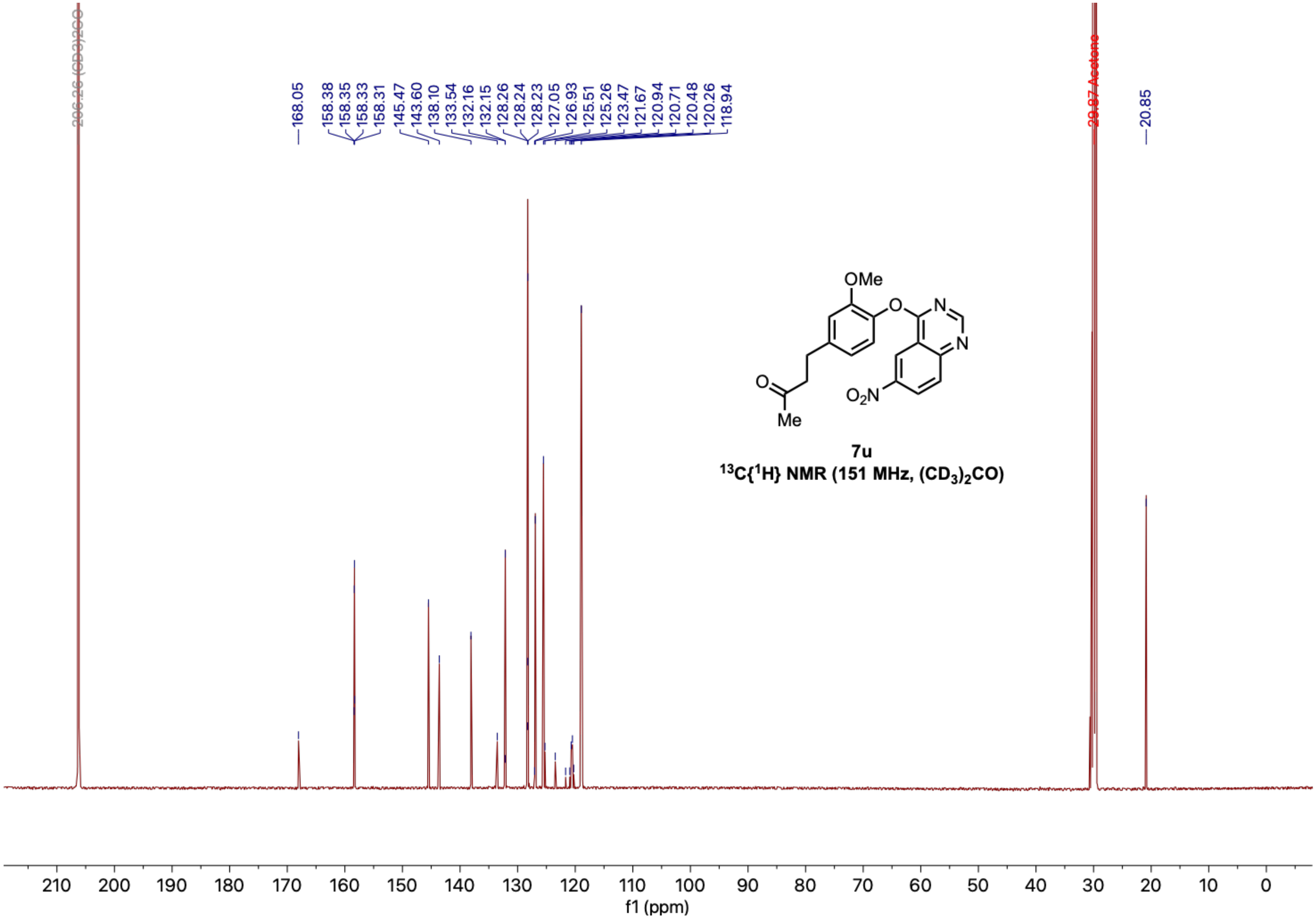

206.26 (CD3)2CO
168.05
158.38
158.35
158.33
158.31
145.47
143.60
138.10
133.54
132.16
132.15
128.26
128.24
128.23
127.05
126.93
125.51
125.26
123.47
121.67
120.94
120.71
120.48
120.26
118.94
29.87 Acetone
20.85
OMe
O
N
N
O
Me
O2N
7u
13C{1H} NMR (151 MHz, (CD3)2CO)
210 200 190 180 170 160 150 140 130 120 110 100 90 80 70 60 50 40 30 20 10 0
f1 (ppm)

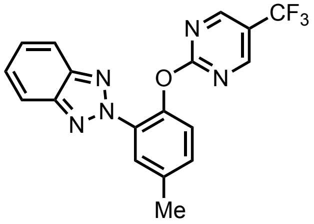

7w

$^{19}F\{^1H\}$ NMR (376 MHz, $(CD_3)_2CO$)

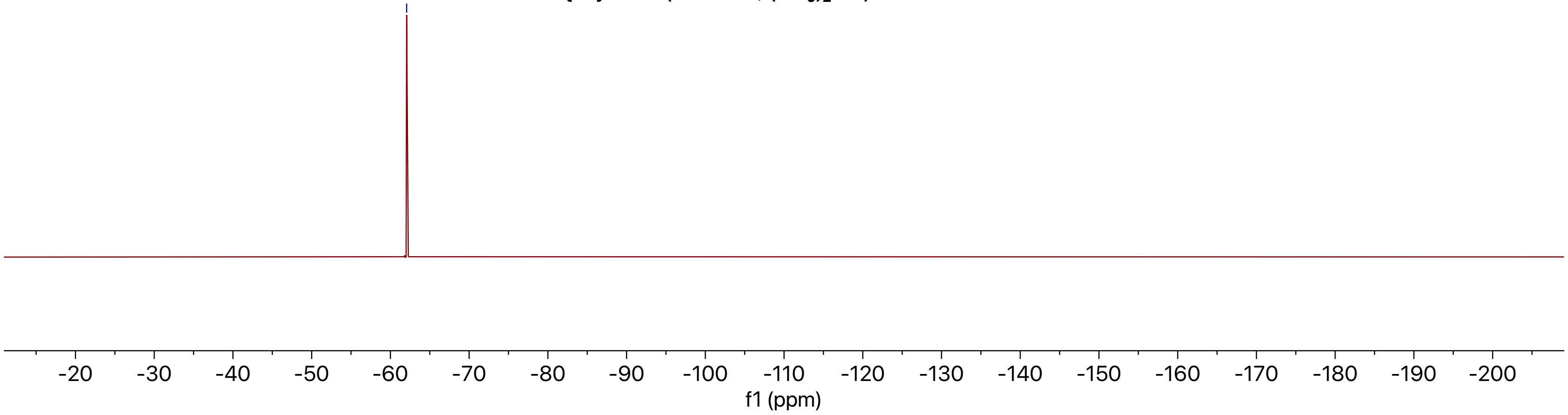

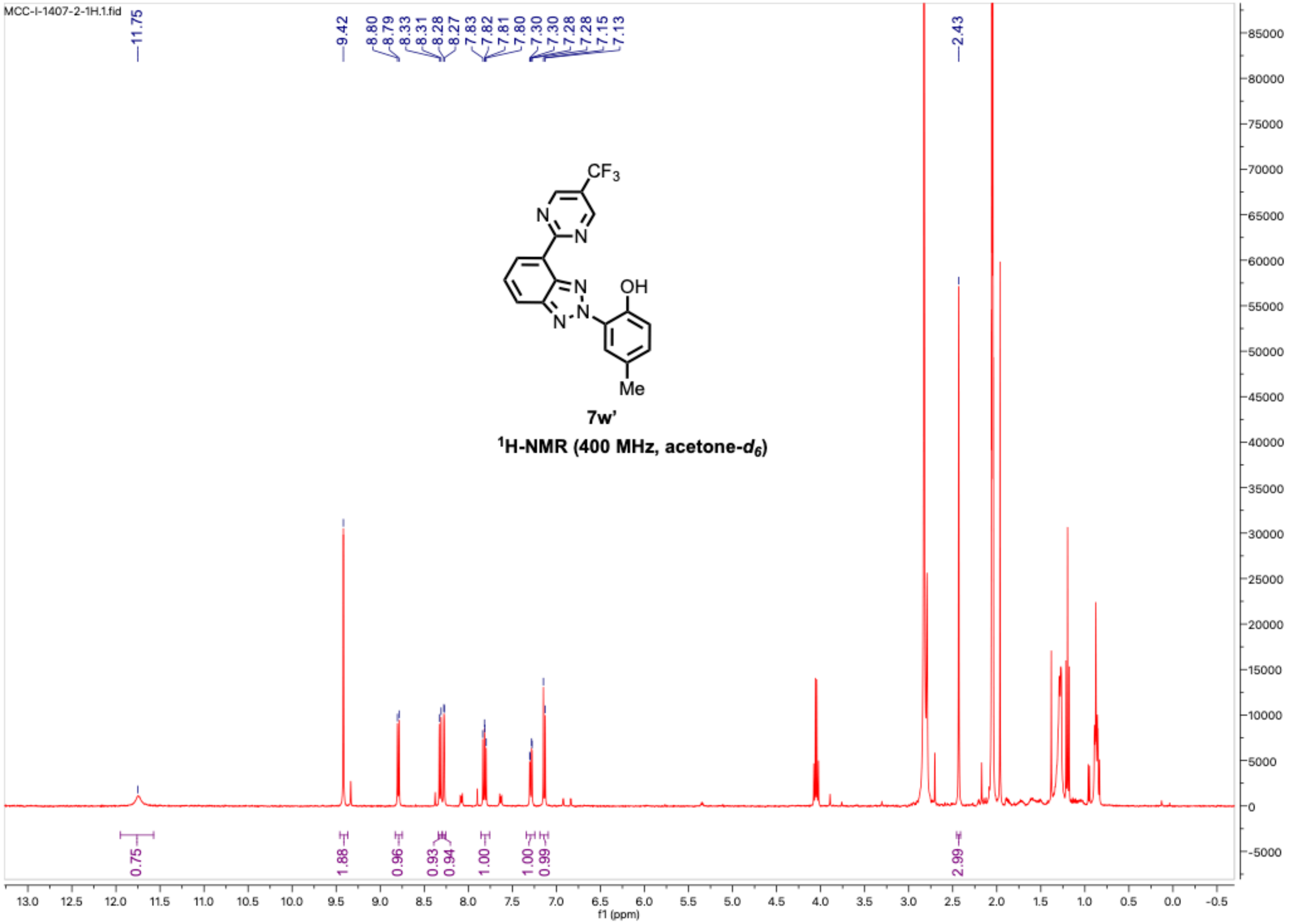
MCC-I-1407-2-1H.1.fid
7w'
^{1}H-NMR (400 MHz, acetone-d_6)
CF3
N
N
N
N
N
OH
Me
11.75
9.42
8.80
8.79
8.33
8.31
8.28
8.27
7.83
7.82
7.81
7.80
7.30
7.30
7.28
7.28
7.15
7.13
2.43
0.75
1.88
0.96
0.93
0.94
1.00
1.00
0.99
2.99
f1 (ppm)

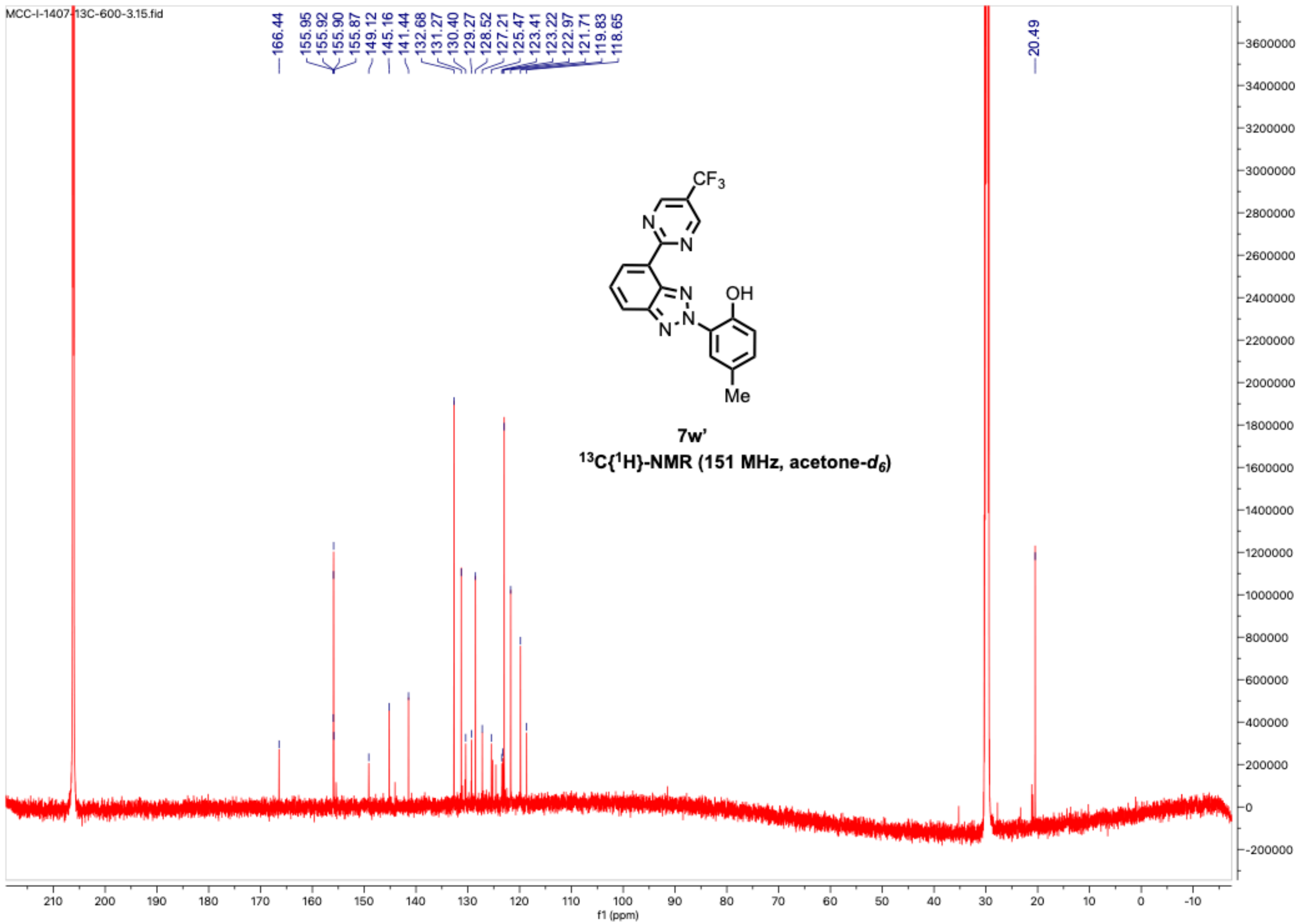

7w'

$^{13}C\{^1H\}$-NMR (151 MHz, acetone-d_6)

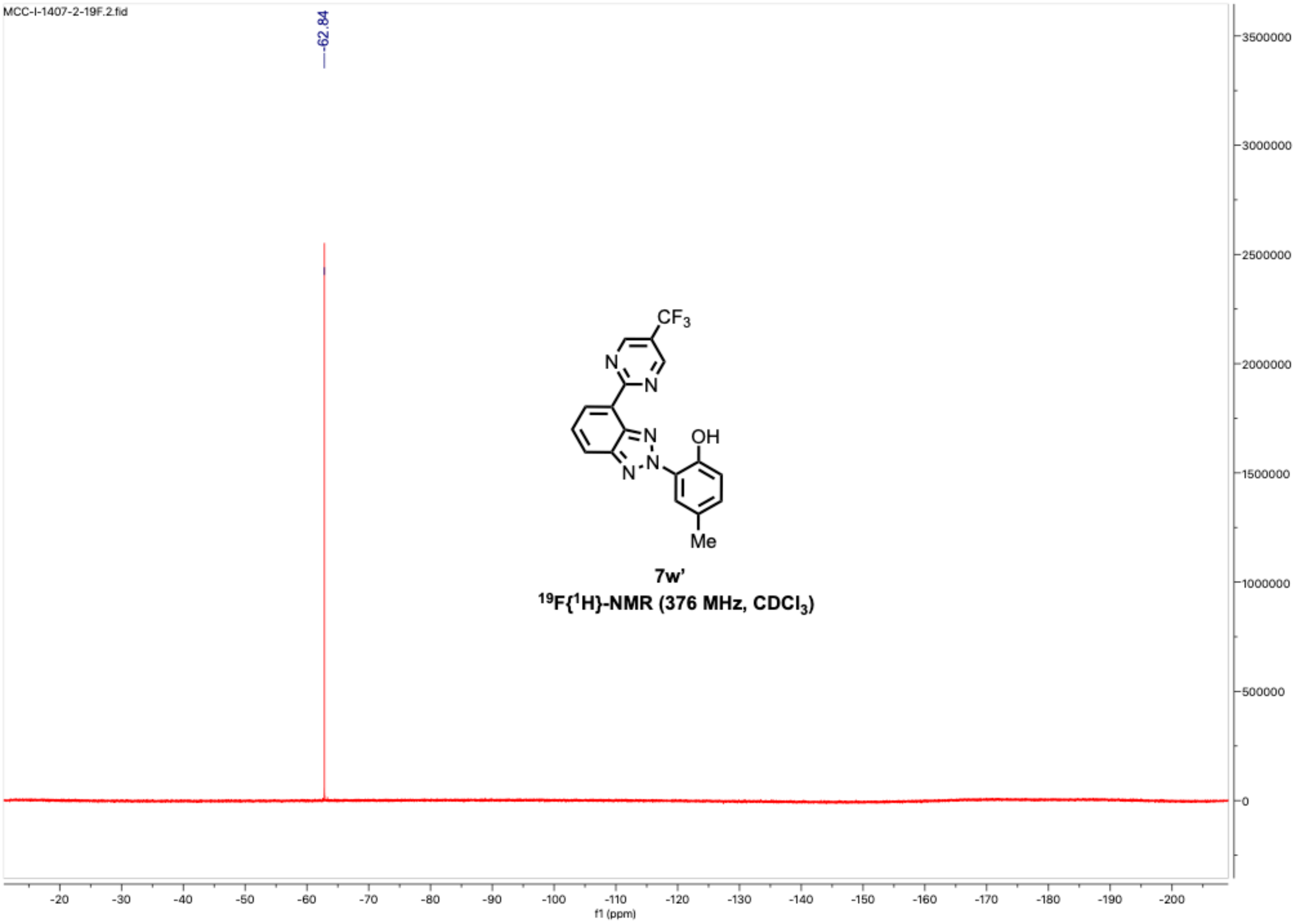

MCC-I-1407-2-19F.2.fid
-62.84
CF3
N
N
N
N
N
OH
Me
7w'
19F{1H}-NMR (376 MHz, CDCl3)
3500000
3000000
2500000
2000000
1500000
1000000
500000
0
-20
-30
-40
-50
-60
-70
-80
-90
-100
-110
-120
-130
-140
-150
-160
-170
-180
-190
-200
f1 (ppm)

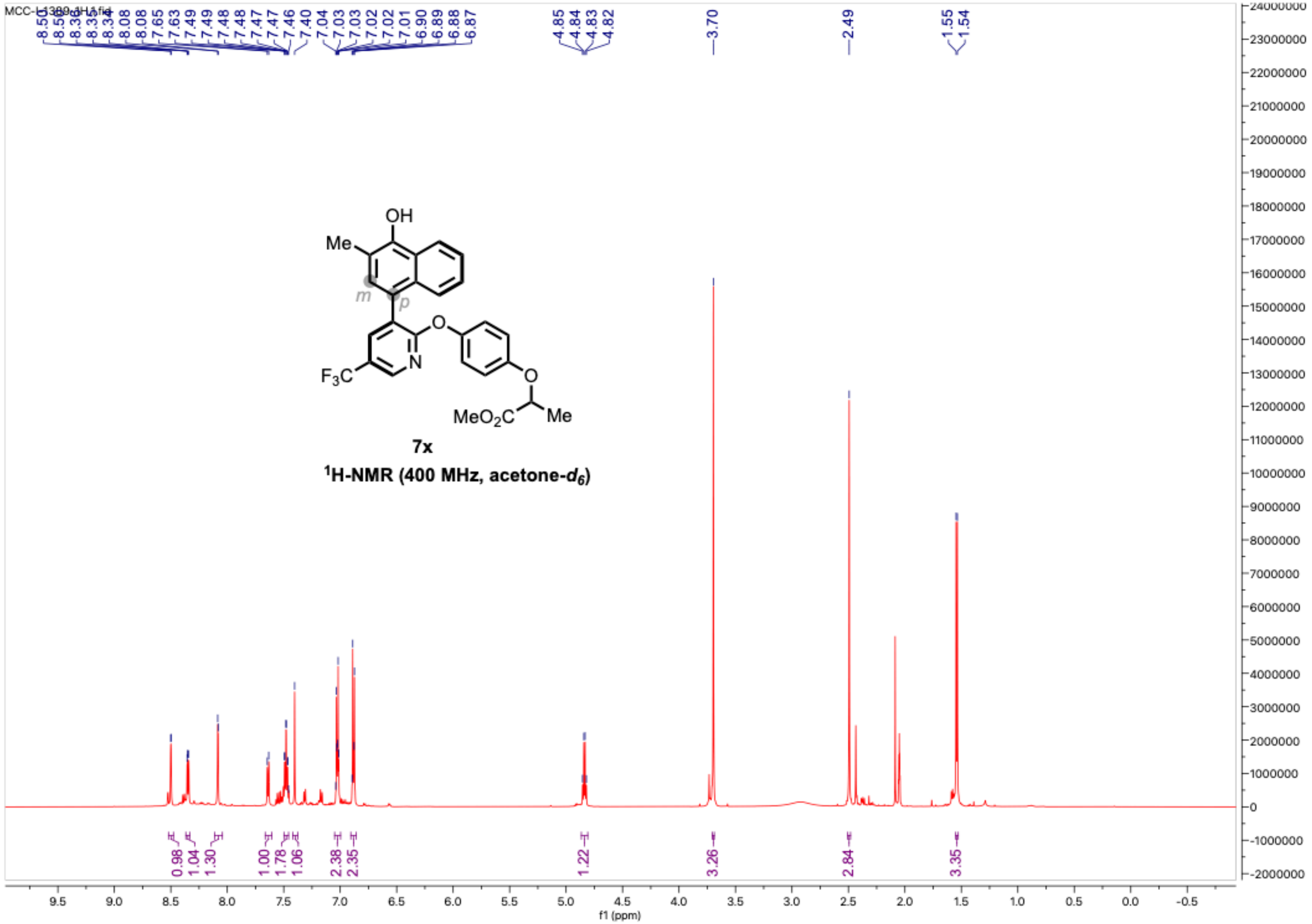
OH
Me
m
p
O
F3C
N
O
MeO2C
Me
7x
1H-NMR (400 MHz, acetone-d6)
f1 (ppm)

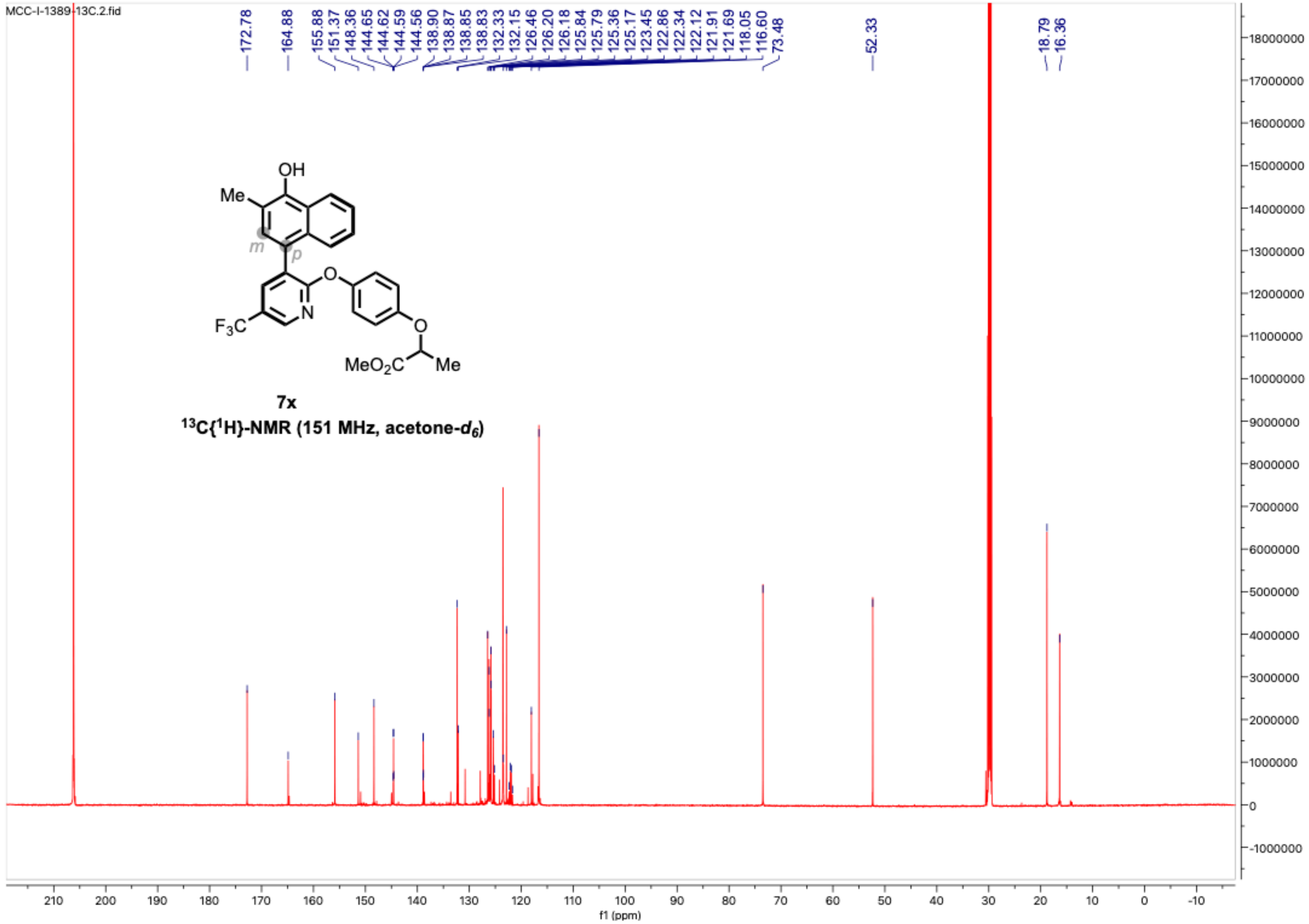
MCC-I-1389-13C.2.fid
172.78
164.88
155.88
151.37
148.36
144.65
144.62
144.59
144.56
138.90
138.87
138.85
138.83
132.33
132.15
126.46
126.20
126.18
125.84
125.79
125.36
125.17
123.45
122.86
122.34
122.12
121.91
121.69
118.05
116.60
73.48
52.33
18.79
16.36
OH
Me
m
p
O
F3C
N
O
MeO2C
Me
7x
13C{1H}-NMR (151 MHz, acetone-d6)
f1 (ppm)

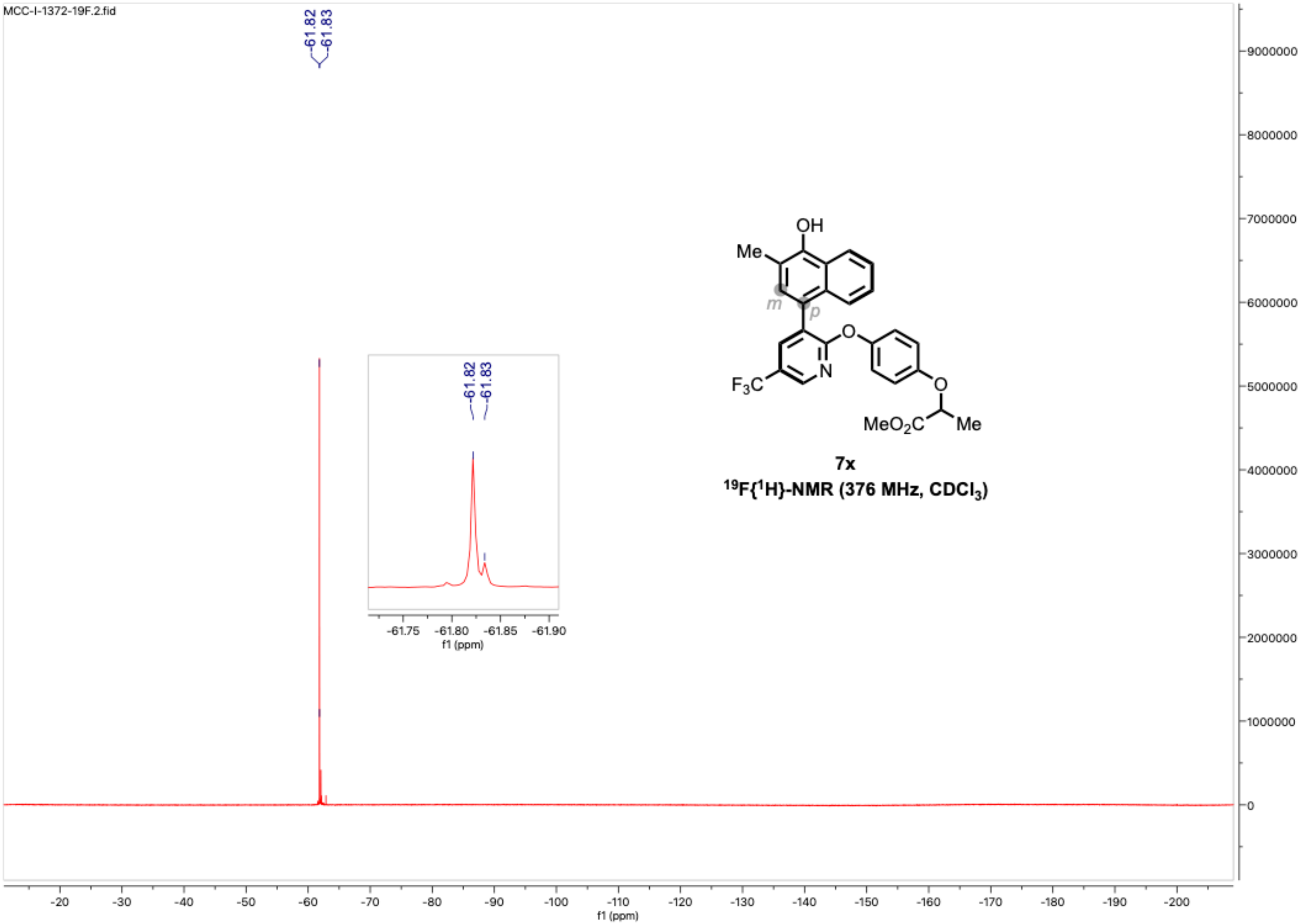

7x

$^{19}F\{^{1}H\}$-NMR (376 MHz, $CDCl_3$)

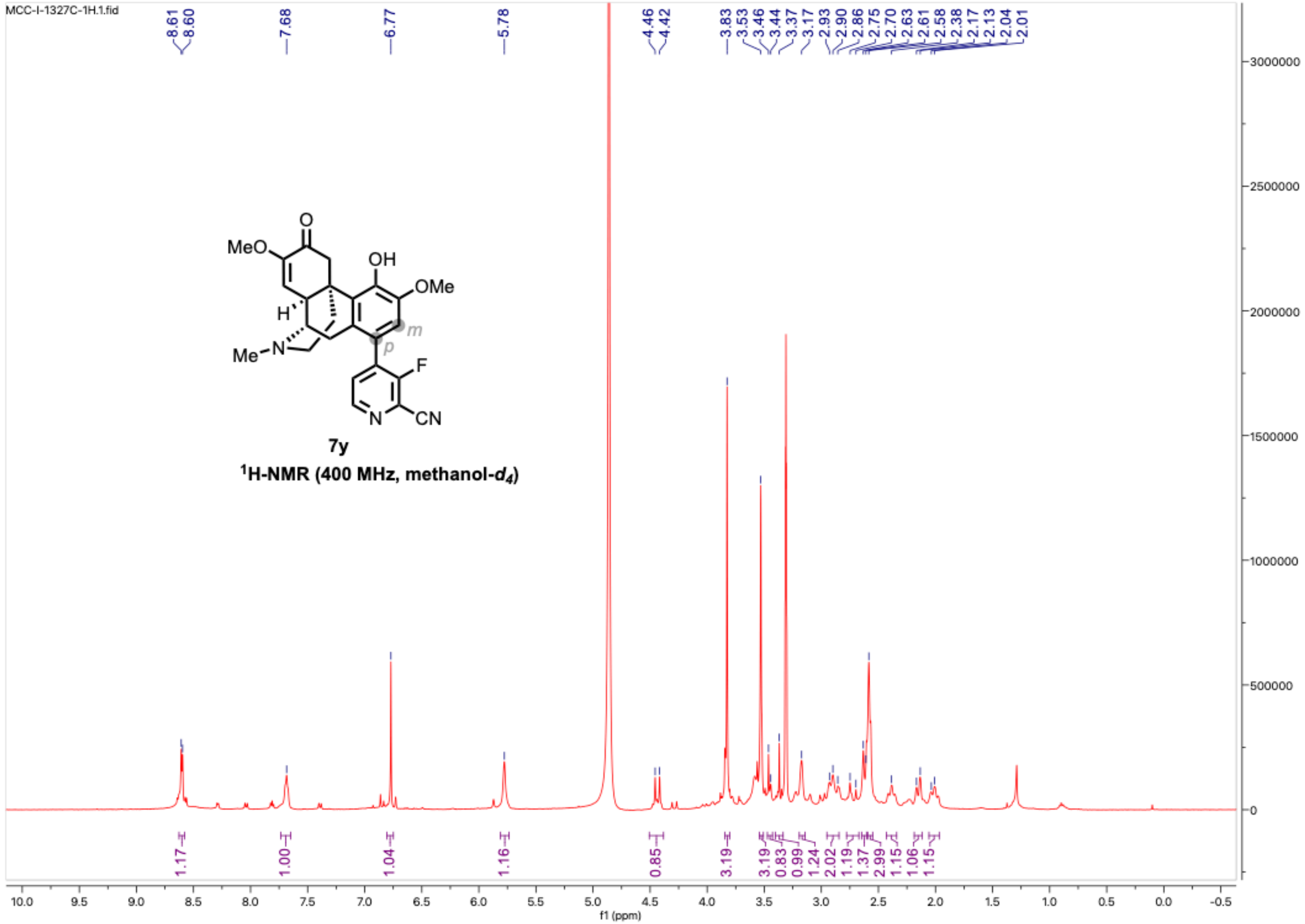
MCC-I-1327C-1H.1.fid
8.61
8.60
7.68
6.77
5.78
4.46
4.42
3.83
3.53
3.46
3.44
3.37
3.17
2.93
2.90
2.86
2.75
2.70
2.63
2.61
2.58
2.38
2.17
2.13
2.04
2.01
MeO
O
OH
OMe
H
Me–N
m
p
F
N
CN
7y
1H-NMR (400 MHz, methanol-d4)
1.17
1.00
1.04
1.16
0.85
3.19
3.19
0.83
0.99
1.24
2.02
1.19
1.37
2.99
1.15
1.06
1.15
3000000
2500000
2000000
1500000
1000000
500000
0
10.0
9.5
9.0
8.5
8.0
7.5
7.0
6.5
6.0
5.5
5.0
4.5
4.0
3.5
3.0
2.5
2.0
1.5
1.0
0.5
0.0
-0.5
f1 (ppm)

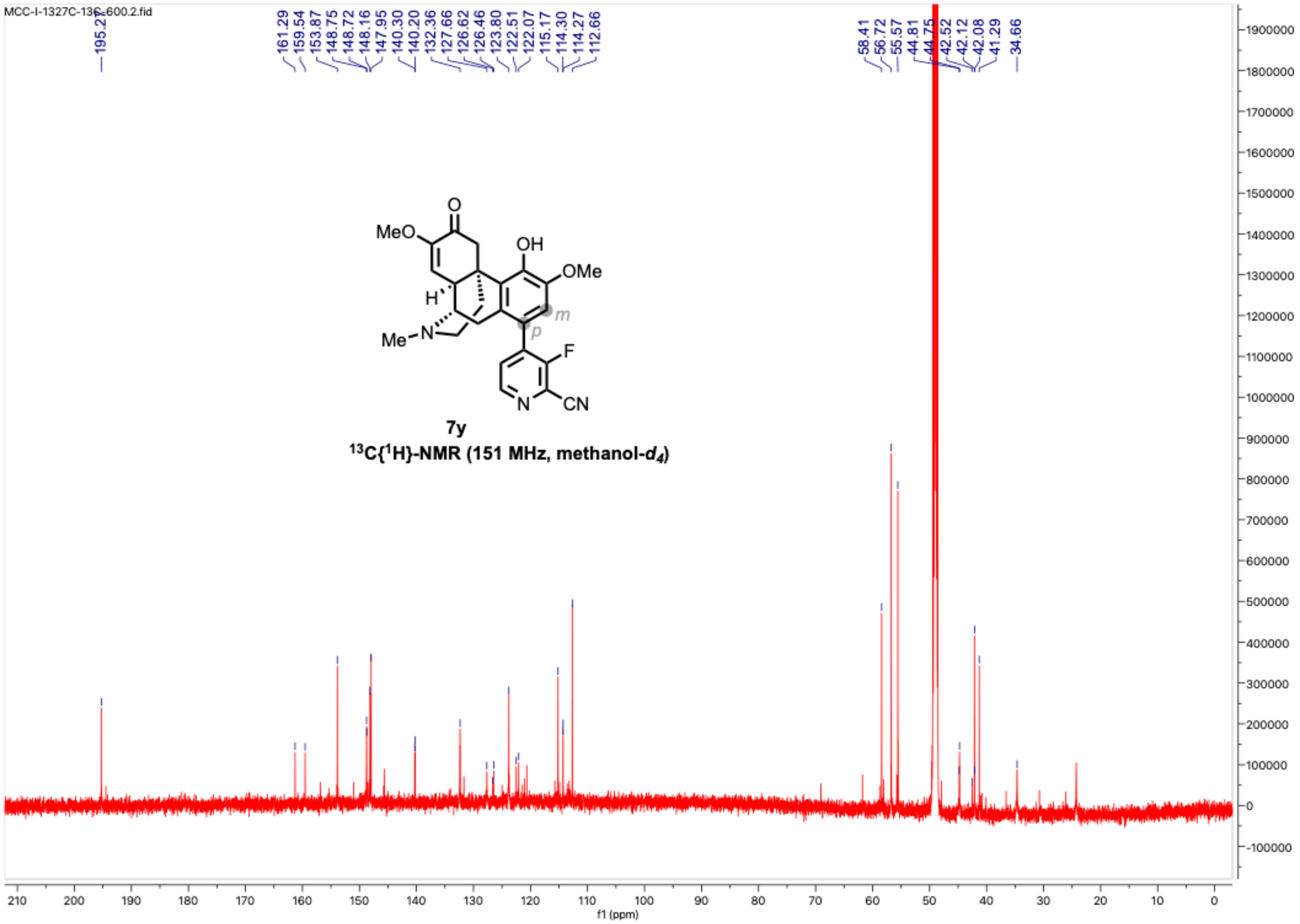

MCC-I-1327C-13C-600.2.fid
195.27
161.29
159.54
153.87
148.75
148.72
148.16
147.95
140.30
140.20
132.36
127.66
126.62
126.46
123.80
122.51
122.07
115.17
114.30
114.27
112.66
58.41
56.72
55.57
44.81
44.75
42.52
42.12
42.08
41.29
34.66
MeO
O
OH
OMe
H
Me
N
m
p
F
N
CN
7y
$^{13}C\{^1H\}$-NMR (151 MHz, methanol-d_4)
f1 (ppm)

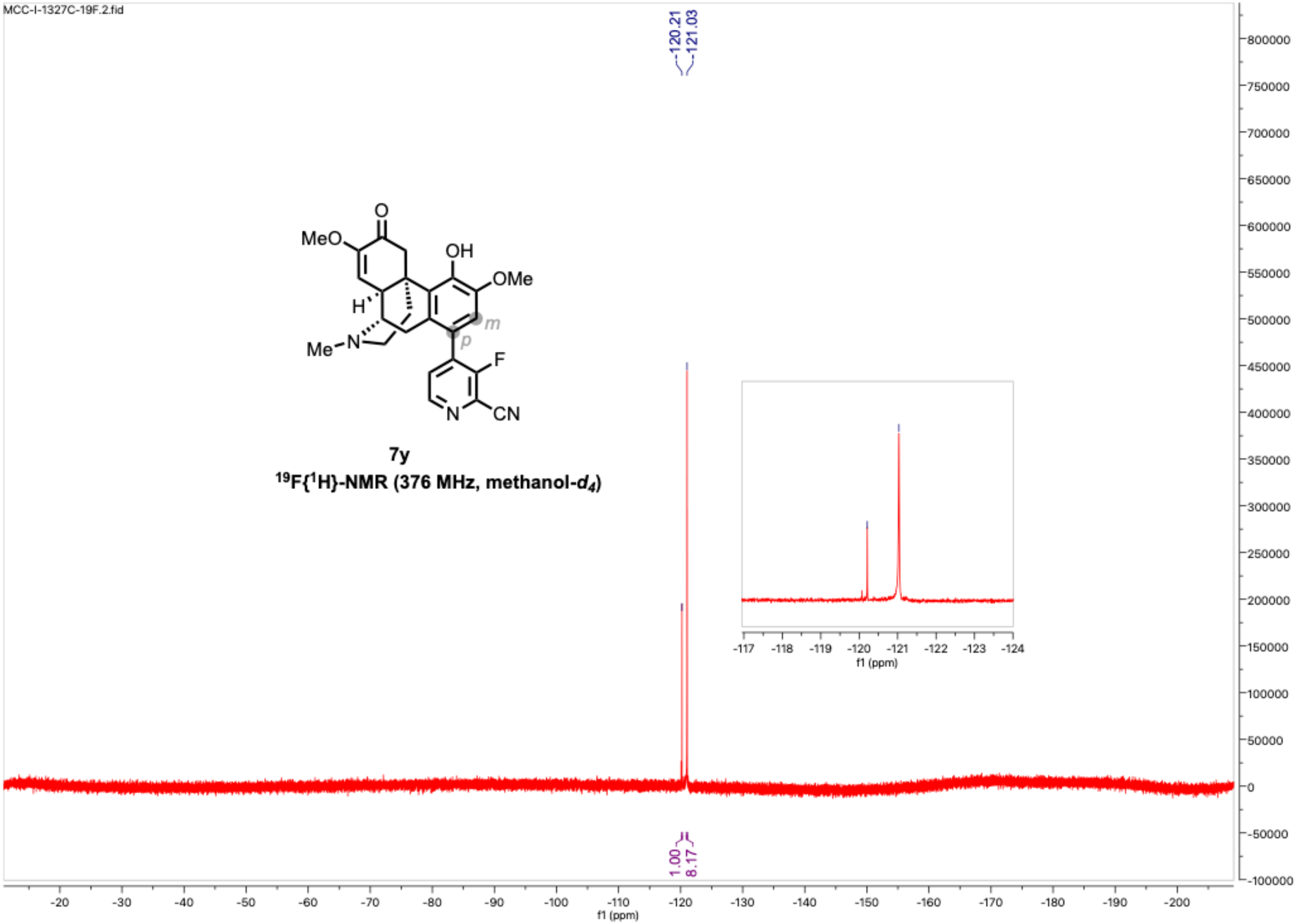
MCC-I-1327C-19F.2.fid
-120.21
-121.03
MeO
O
OH
OMe
H
Me-N
m
p
F
N
CN
7y
19F{1H}-NMR (376 MHz, methanol-d4)
1.00
8.17
f1 (ppm)

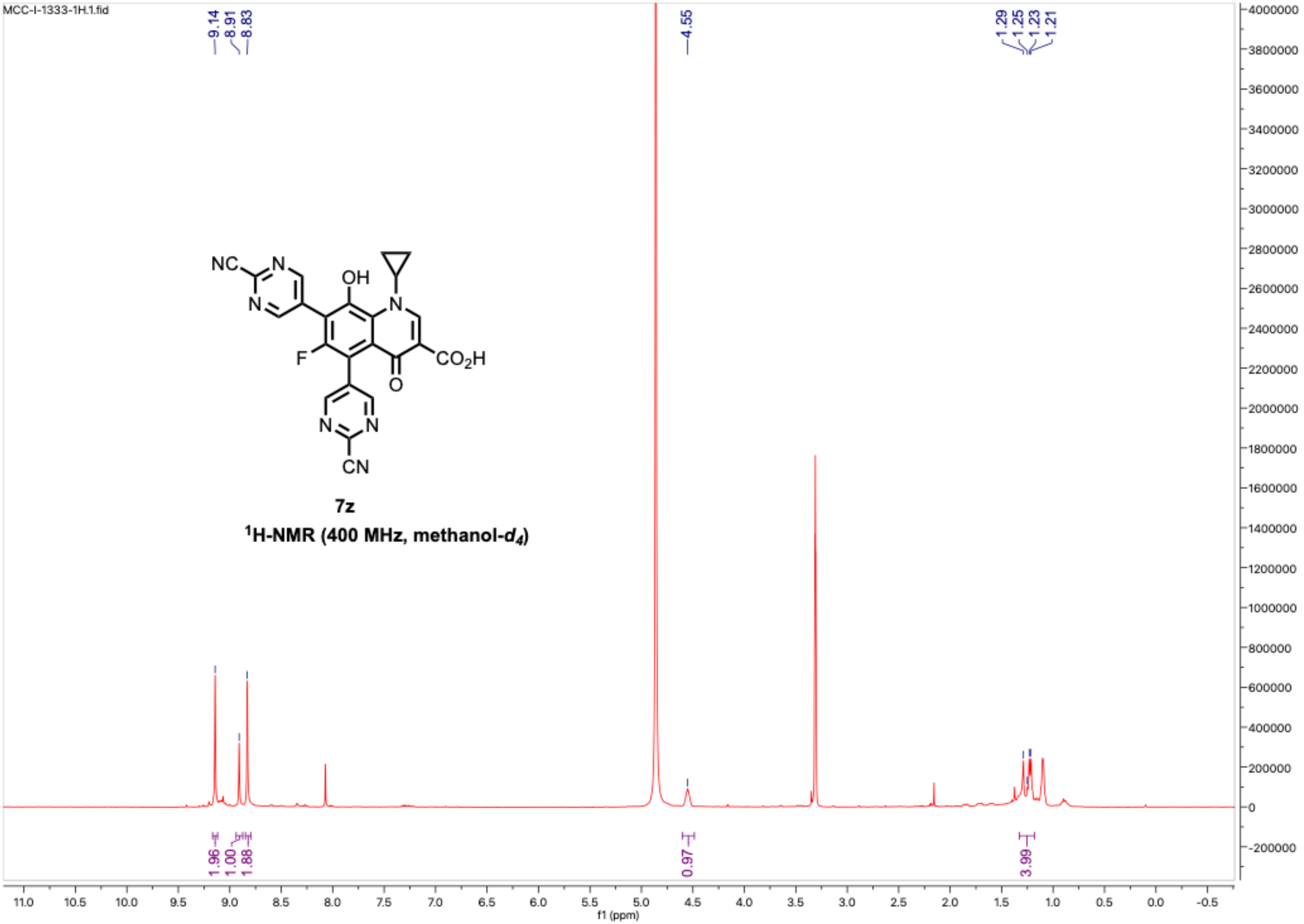

7z

^{1}H-NMR (400 MHz, methanol-d_4)

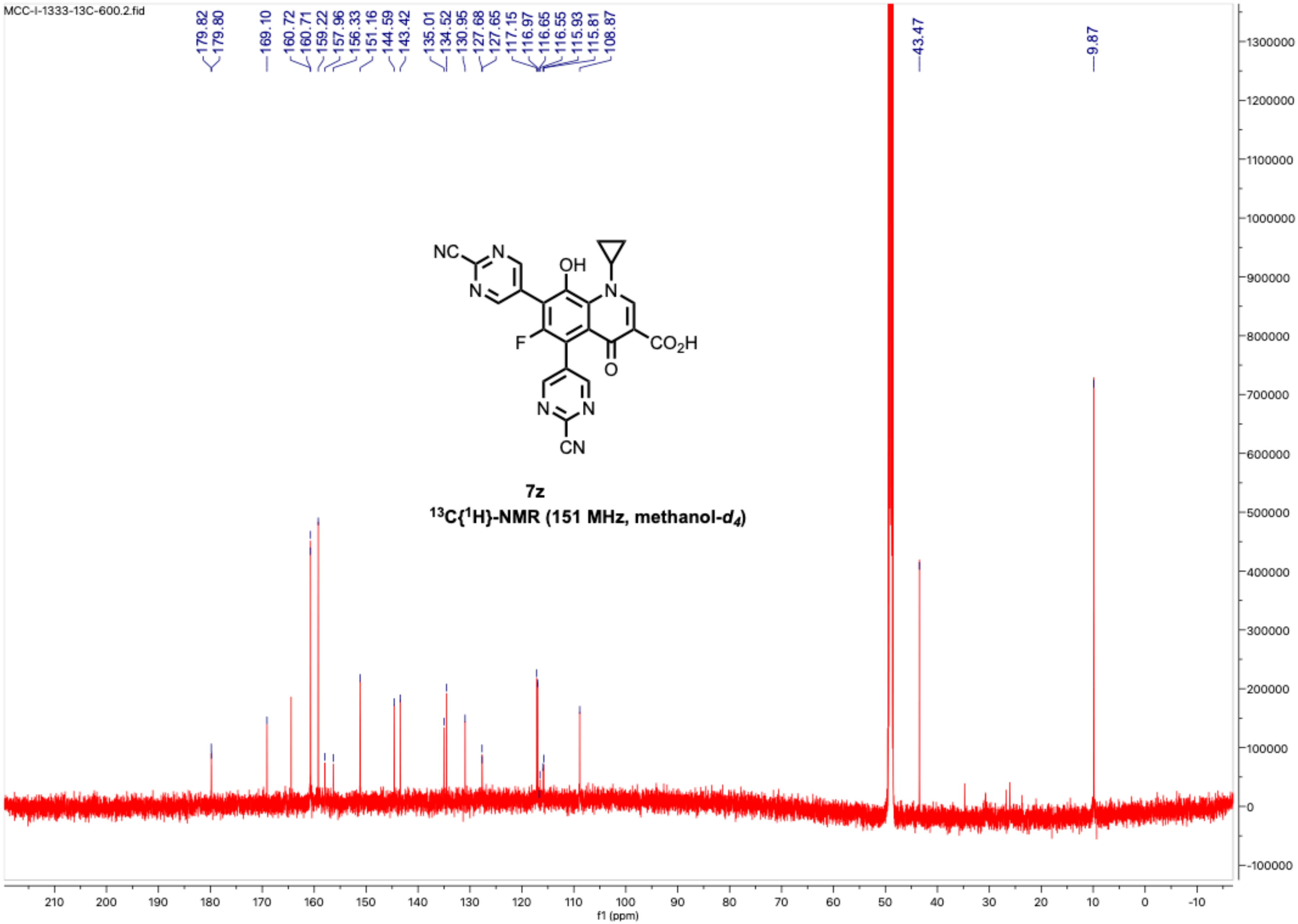
MCC-I-1333-13C-600.2.fid
179.82
179.80
169.10
160.72
160.71
159.22
157.96
156.33
151.16
144.59
143.42
135.01
134.52
130.95
127.68
127.65
117.15
116.97
116.65
116.55
115.93
115.81
108.87
43.47
9.87
NC
N
N
OH
N
F
CO2H
O
N
N
CN
7z
13C{1H}-NMR (151 MHz, methanol-d4)
f1 (ppm)

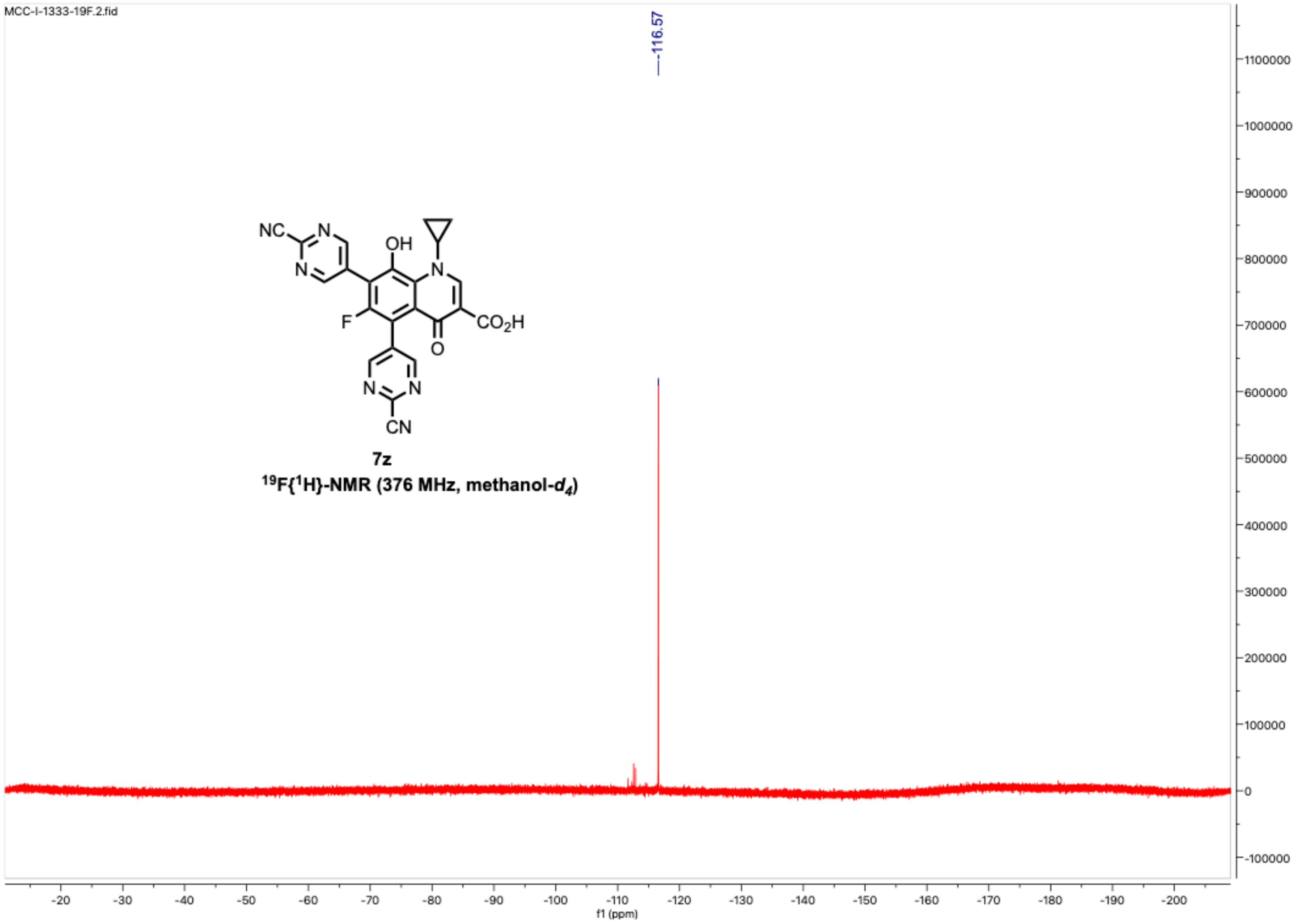

7z

$^{19}F\{^{1}H\}$-NMR (376 MHz, methanol-d_4)

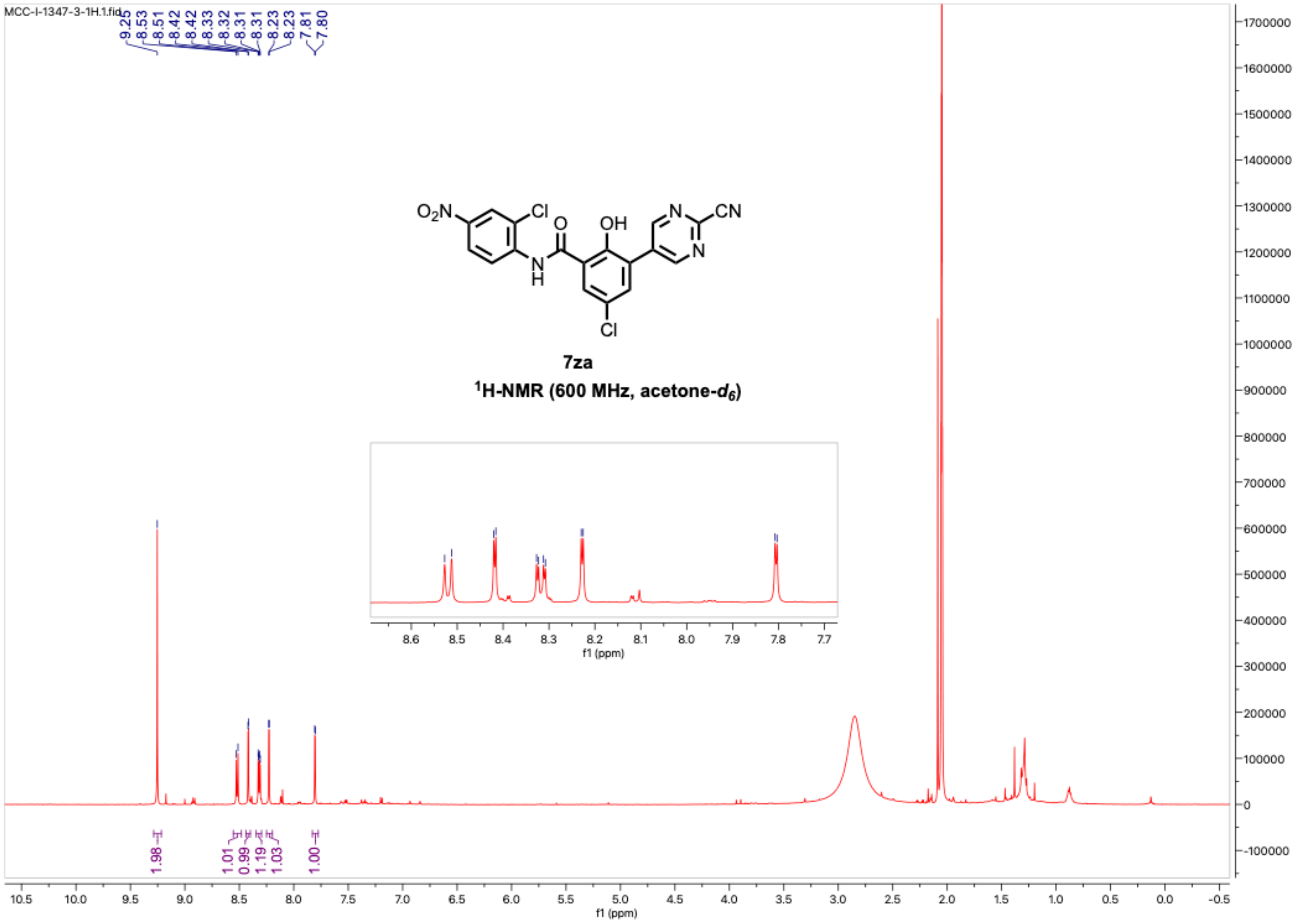
MCC-I-1347-3-1H.1.fid
9.25
8.53
8.51
8.42
8.42
8.33
8.32
8.31
8.31
8.23
8.23
7.81
7.80
O_2N
Cl
O
OH
N
CN
N
N
H
Cl
7za
^{1}H-NMR (600 MHz, acetone-d_6)
1.98
1.01
0.99
1.19
1.03
1.00
f1 (ppm)

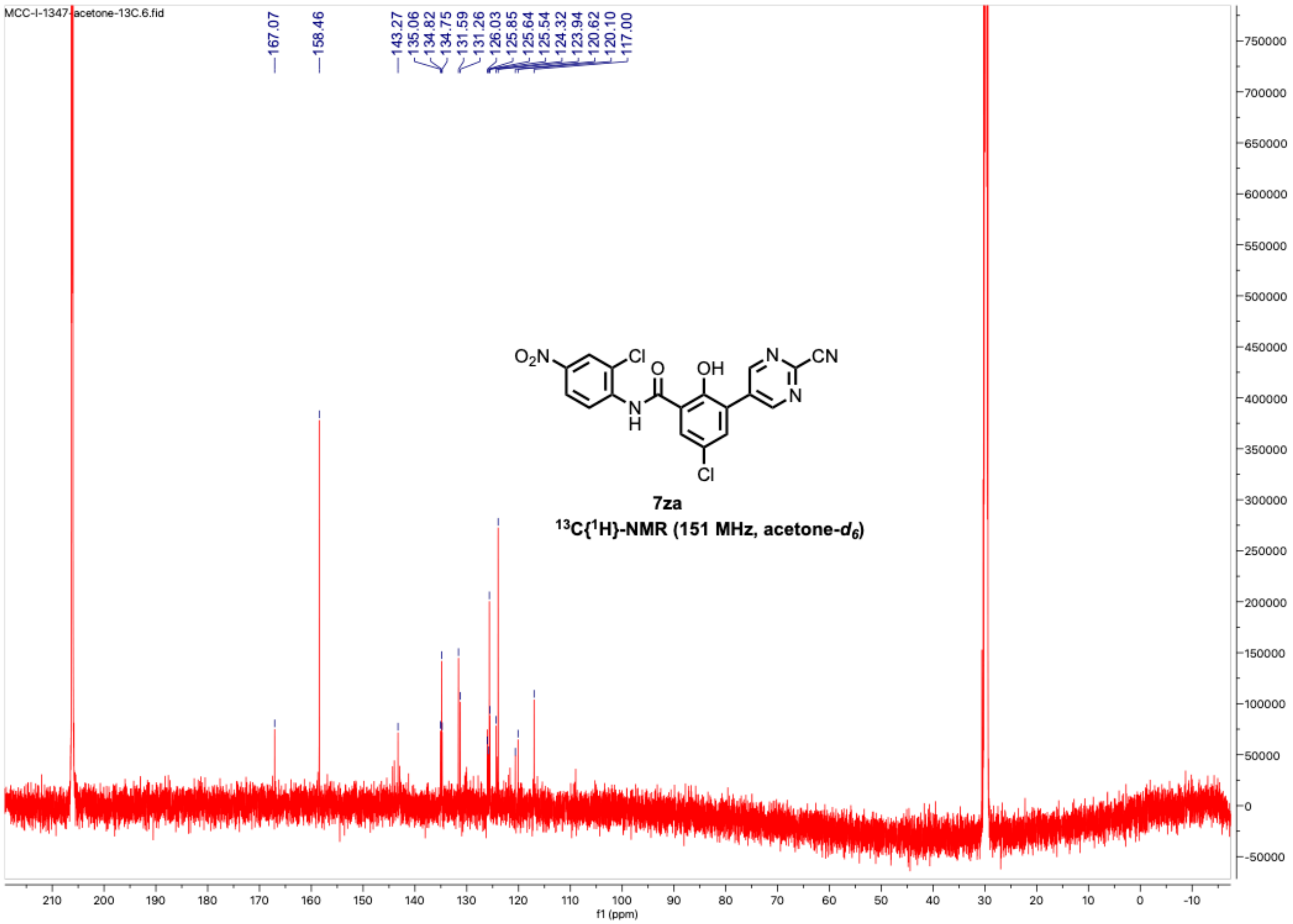
MCC-I-1347-acetone-13C.6.fid
167.07
158.46
143.27
135.06
134.82
134.75
131.59
131.26
126.03
125.85
125.64
125.54
124.32
123.94
120.62
120.10
117.00
7za
13C{1H}-NMR (151 MHz, acetone-d6)
f1 (ppm)

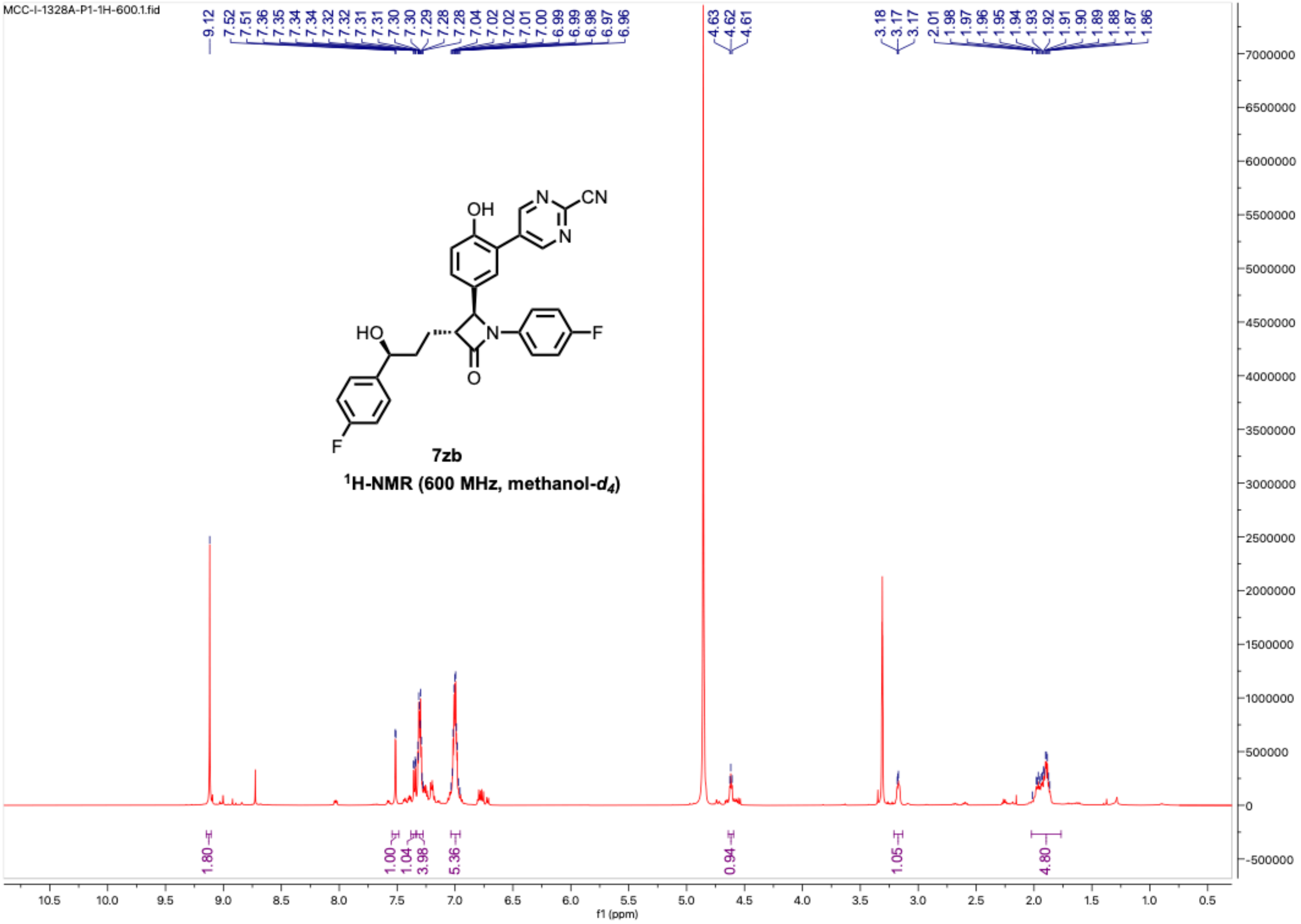
MCC-I-1328A-P1-1H-600.1.fid
7zb
^{1}H-NMR (600 MHz, methanol-d_4)
f1 (ppm)

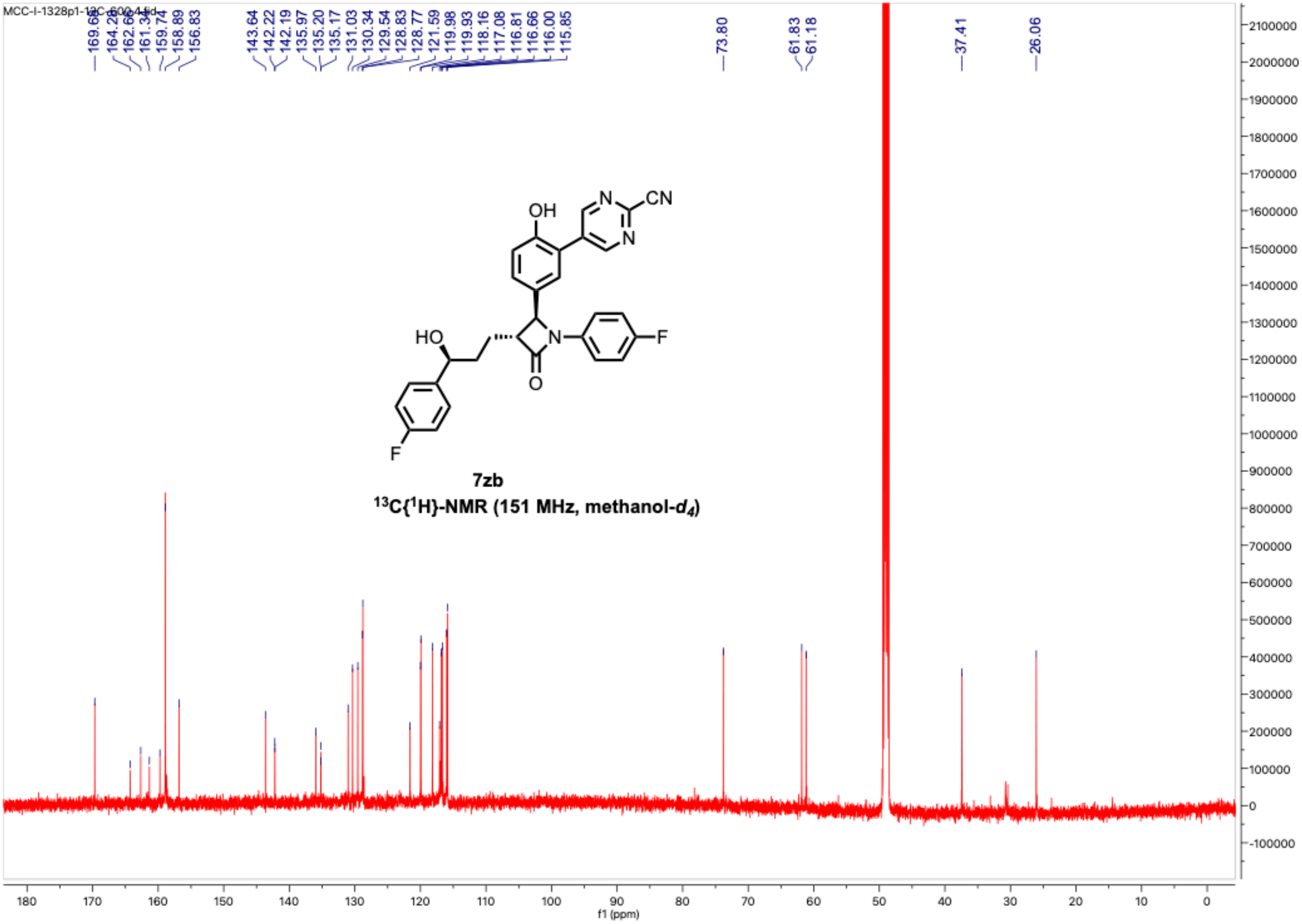

7zb

$^{13}C\{^1H\}$-NMR (151 MHz, methanol-d_4)

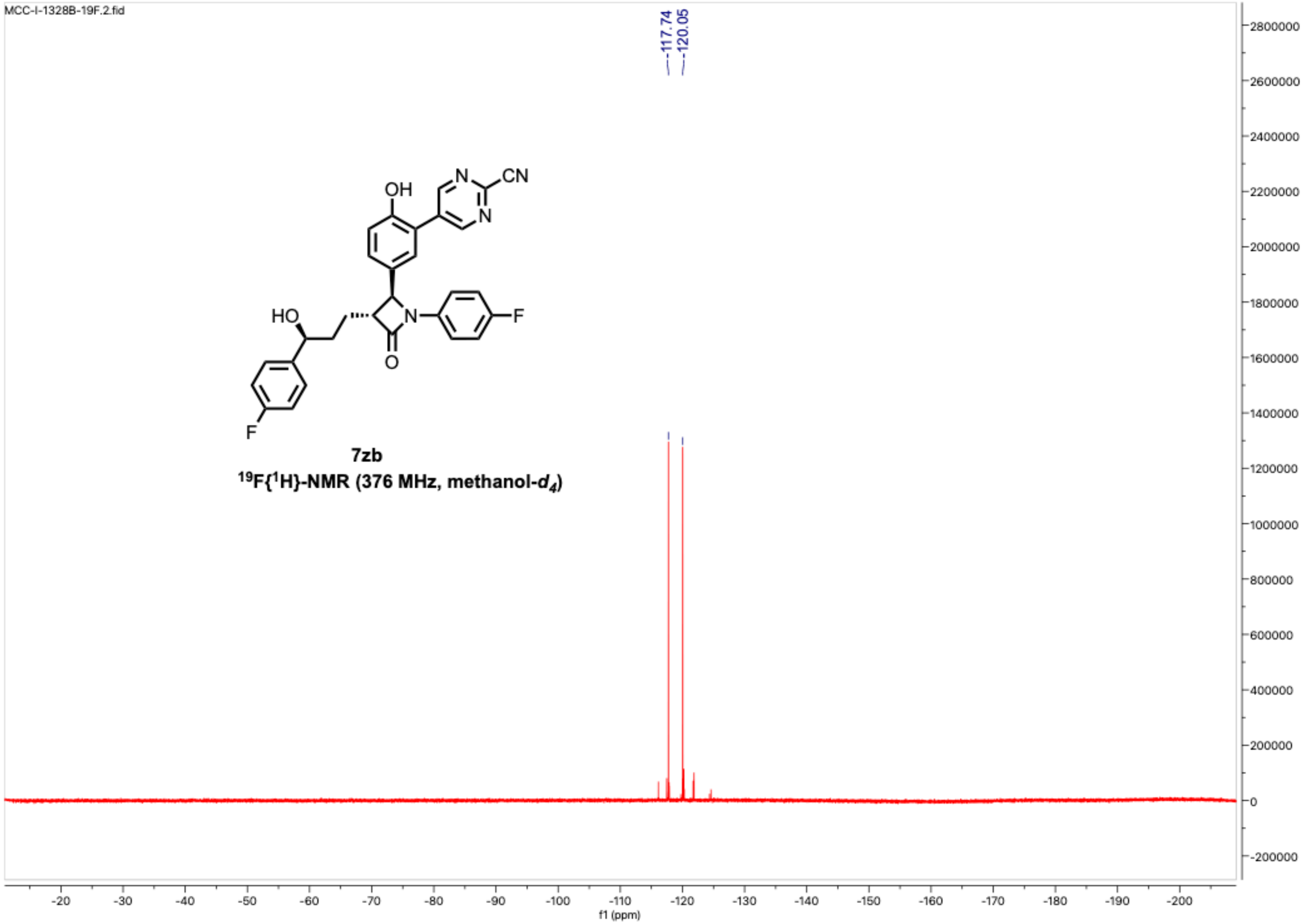

7zb

$^{19}F\{^{1}H\}$-NMR (376 MHz, methanol-*d*$_4$)

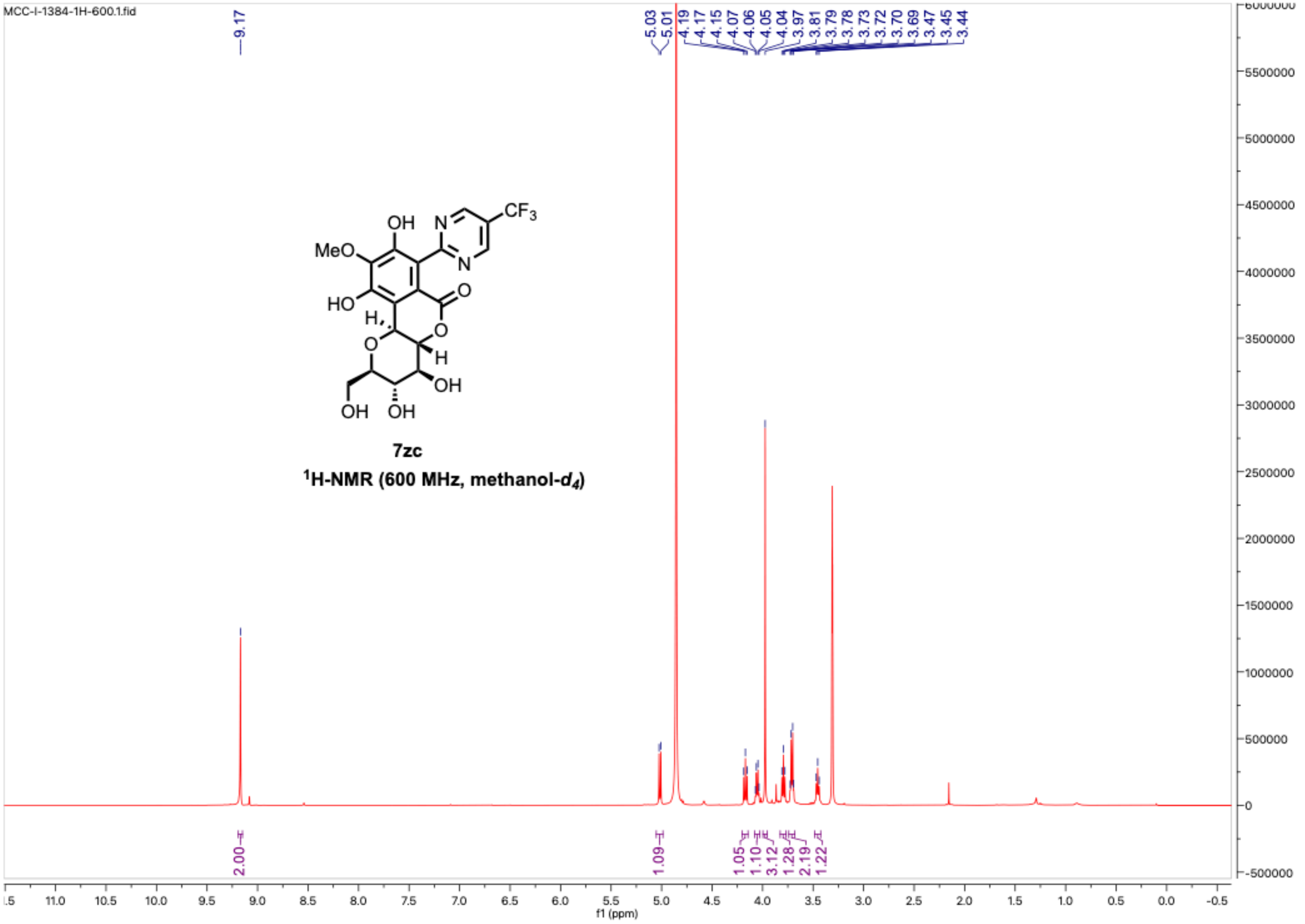
MCC-I-1384-1H-600.1.fid
9.17
5.03
5.01
4.19
4.17
4.15
4.07
4.06
4.05
4.04
3.97
3.81
3.79
3.78
3.73
3.72
3.70
3.69
3.47
3.45
3.44
OH
MeO
HO
N
N
CF3
O
O
O
H
H
OH
OH
OH
7zc
1H-NMR (600 MHz, methanol-d4)
2.00
1.09
1.05
1.10
3.12
1.28
2.19
1.22
f1 (ppm)

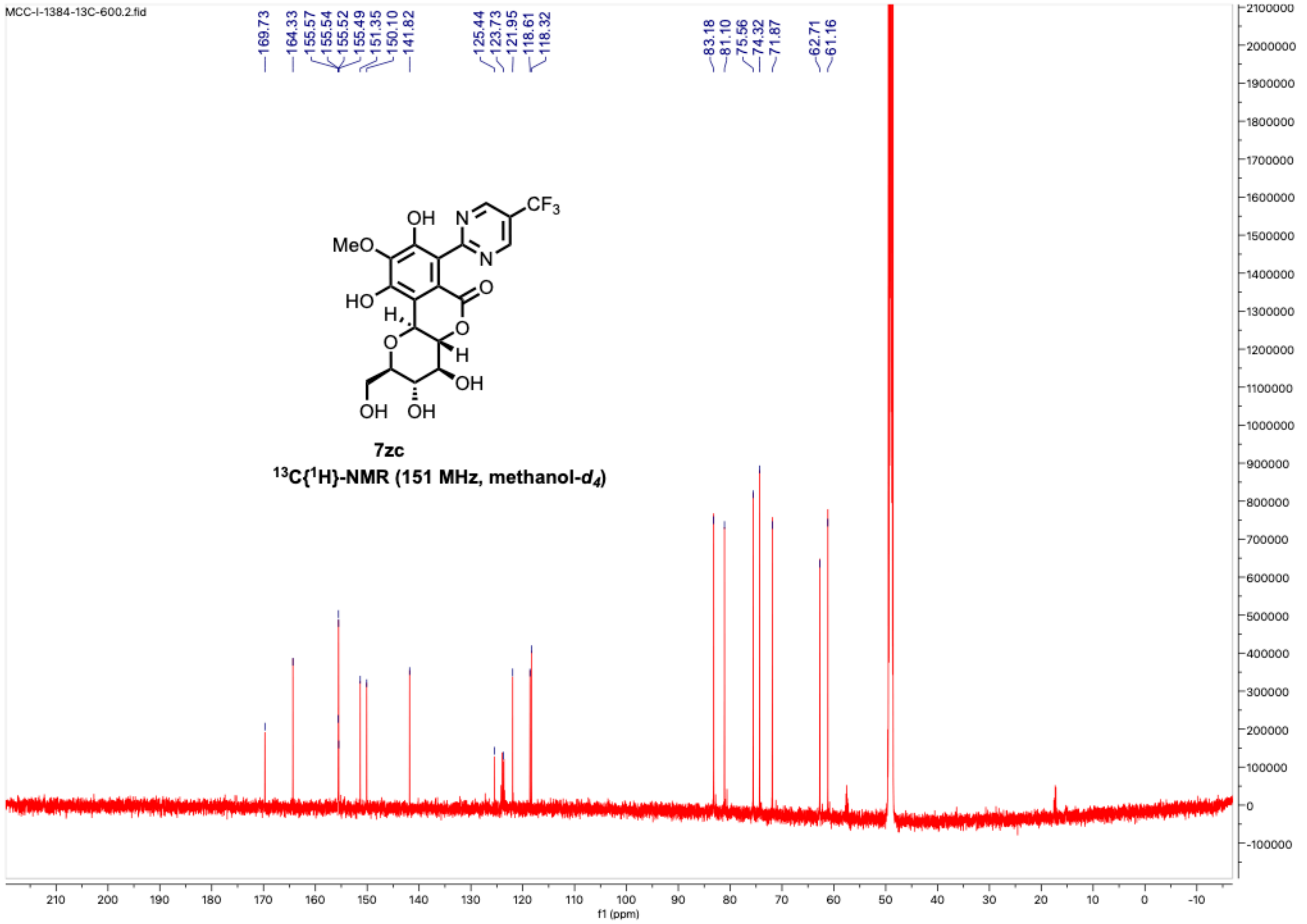
MCC-I-1384-13C-600.2.fid
169.73
164.33
155.57
155.54
155.52
155.49
151.35
150.10
141.82
125.44
123.73
121.95
118.61
118.32
83.18
81.10
75.56
74.32
71.87
62.71
61.16
OH
MeO
HO
N
N
CF3
O
O
H
H
O
OH
OH
OH
7zc
13C{1H}-NMR (151 MHz, methanol-d4)
f1 (ppm)

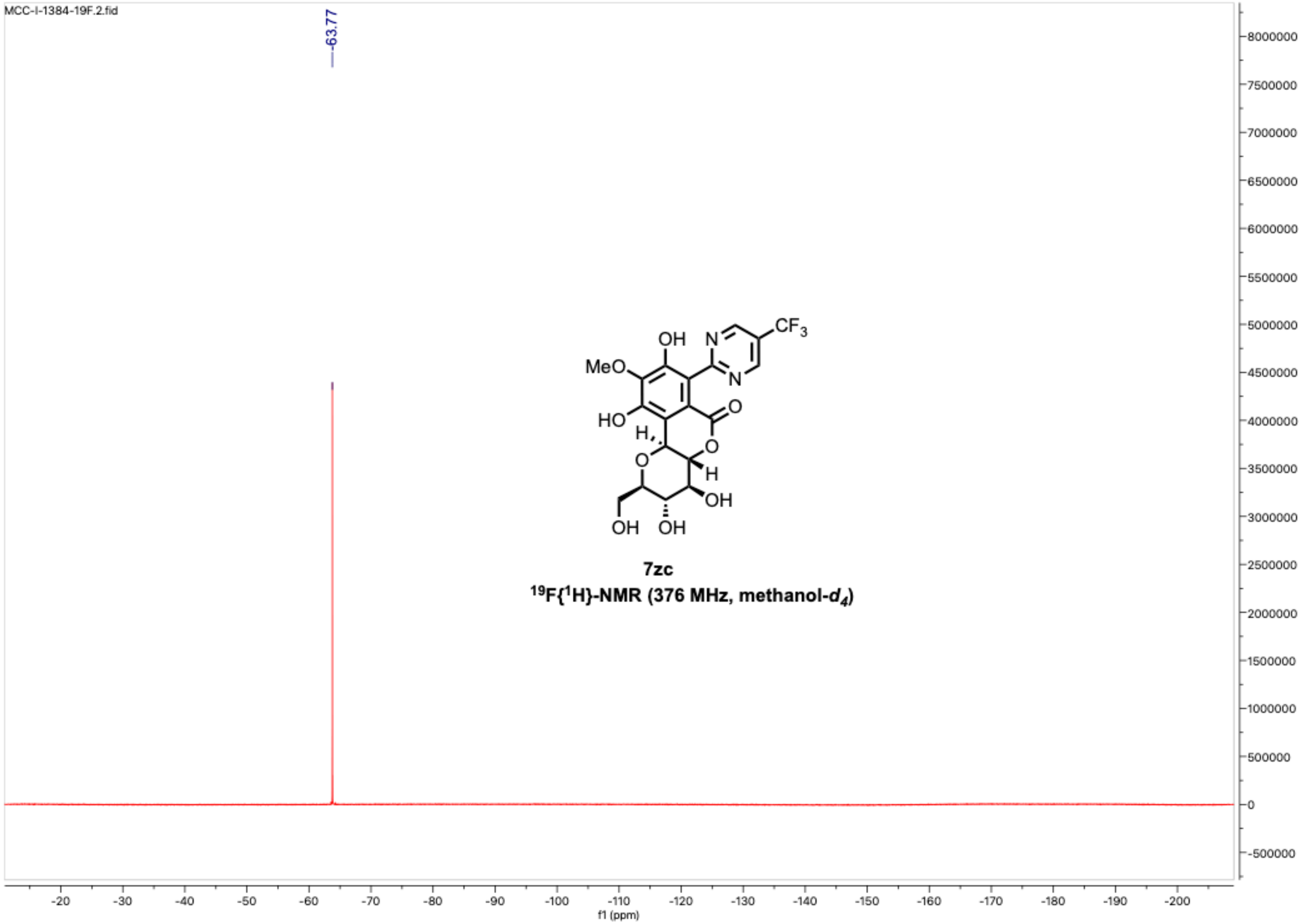

7zc

$^{19}F\{^{1}H\}$-NMR (376 MHz, methanol-d_4)

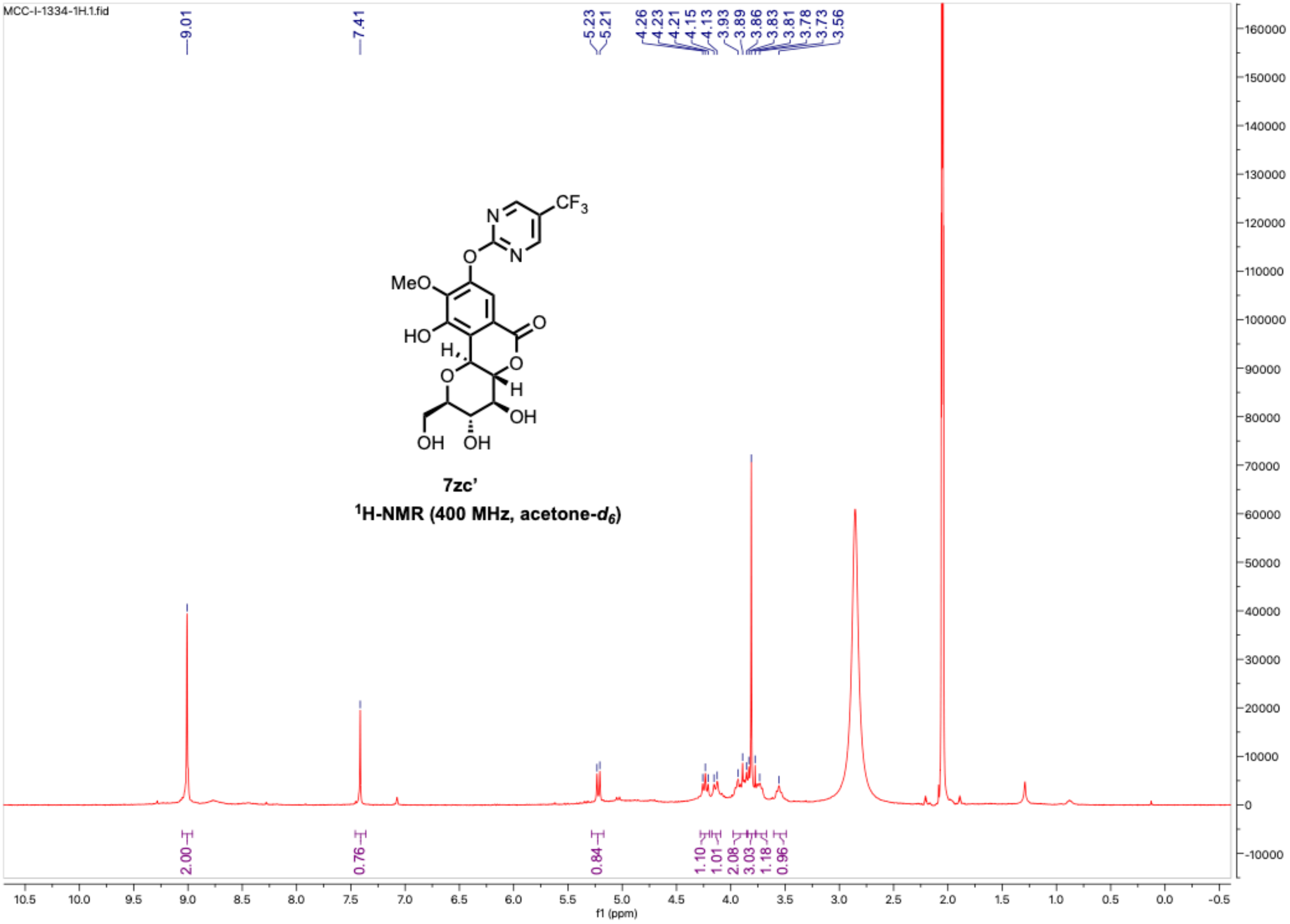
MCC-I-1334-1H.1.fid
9.01
7.41
5.23
5.21
4.26
4.23
4.21
4.15
4.13
3.93
3.89
3.86
3.83
3.81
3.78
3.73
3.56
CF3
N
N
O
MeO
HO
H
O
O
O
H
OH
OH
OH
7zc'
1H-NMR (400 MHz, acetone-d6)
2.00
0.76
0.84
1.10
1.01
2.08
3.03
1.18
0.96
f1 (ppm)

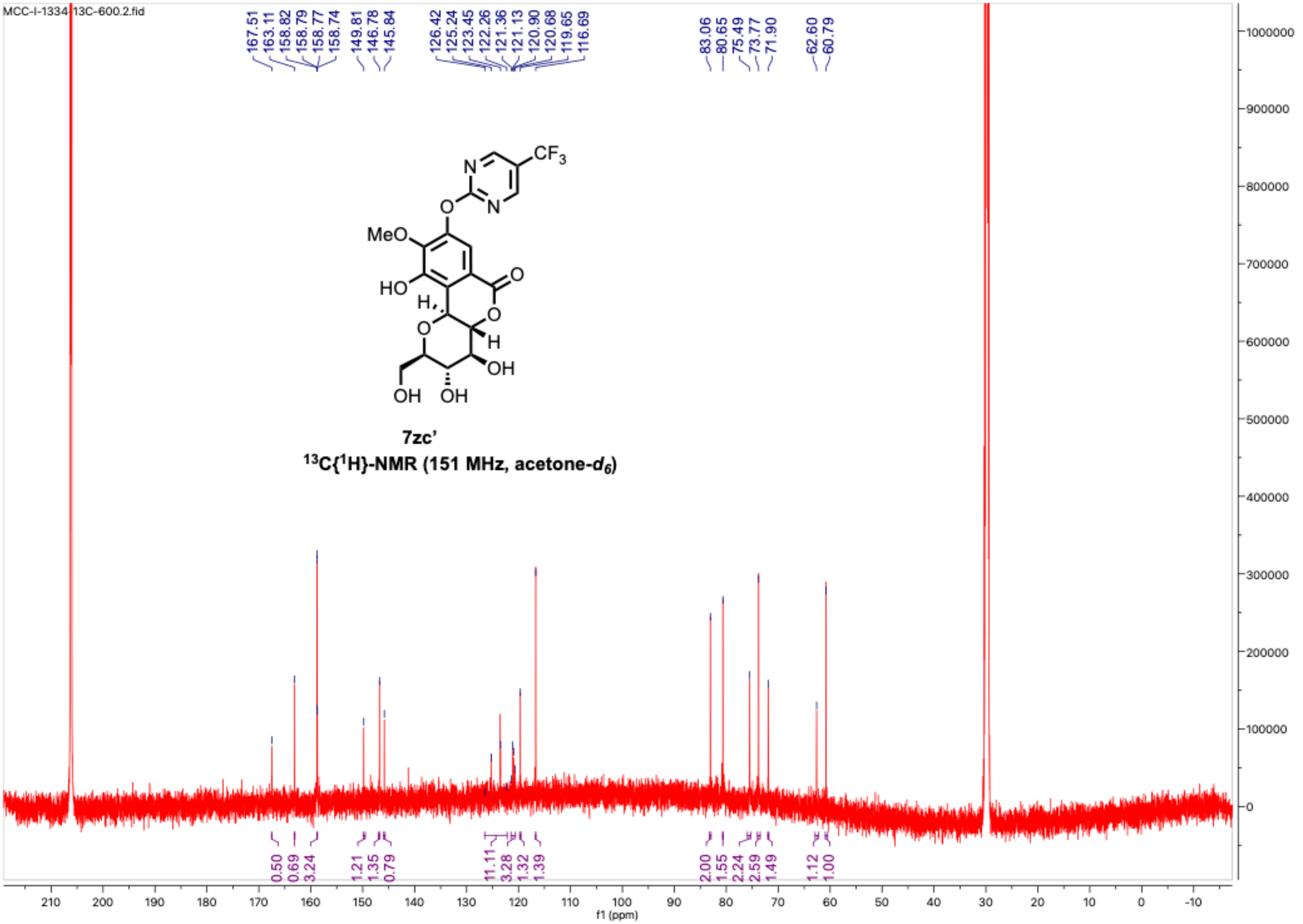
MCC-I-1334-13C-600.2.fid
167.51
163.11
158.82
158.79
158.77
158.74
149.81
146.78
145.84
126.42
125.24
123.45
122.26
121.36
121.13
120.90
120.68
119.65
116.69
83.06
80.65
75.49
73.77
71.90
62.60
60.79
CF3
N
N
O
MeO
HO
O
H
O
O
H
OH
OH
OH
7zc'
13C{1H}-NMR (151 MHz, acetone-d6)
0.50
0.69
3.24
1.21
1.35
0.79
11.11
3.28
1.32
1.39
2.00
1.55
2.24
2.59
1.49
1.12
1.00
f1 (ppm)

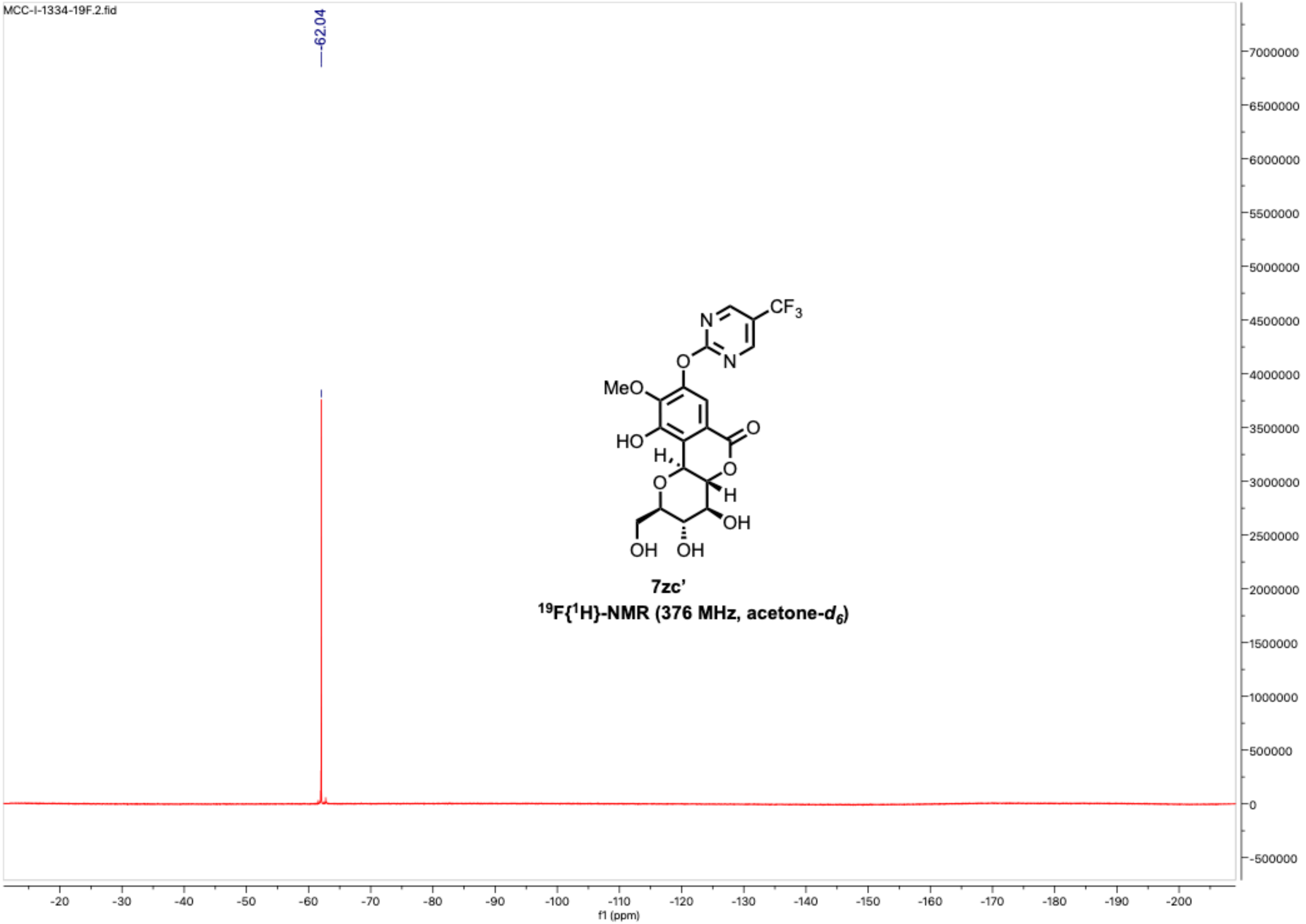
MCC-I-1334-19F.2.fid
-62.04
CF3
N
N
O
MeO
HO
O
H
O
H
O
OH
OH
OH
7zc'
19F{1H}-NMR (376 MHz, acetone-d6)
7000000
6500000
6000000
5500000
5000000
4500000
4000000
3500000
3000000
2500000
2000000
1500000
1000000
500000
0
-500000
-20
-30
-40
-50
-60
-70
-80
-90
-100
-110
-120
-130
-140
-150
-160
-170
-180
-190
-200
f1 (ppm)

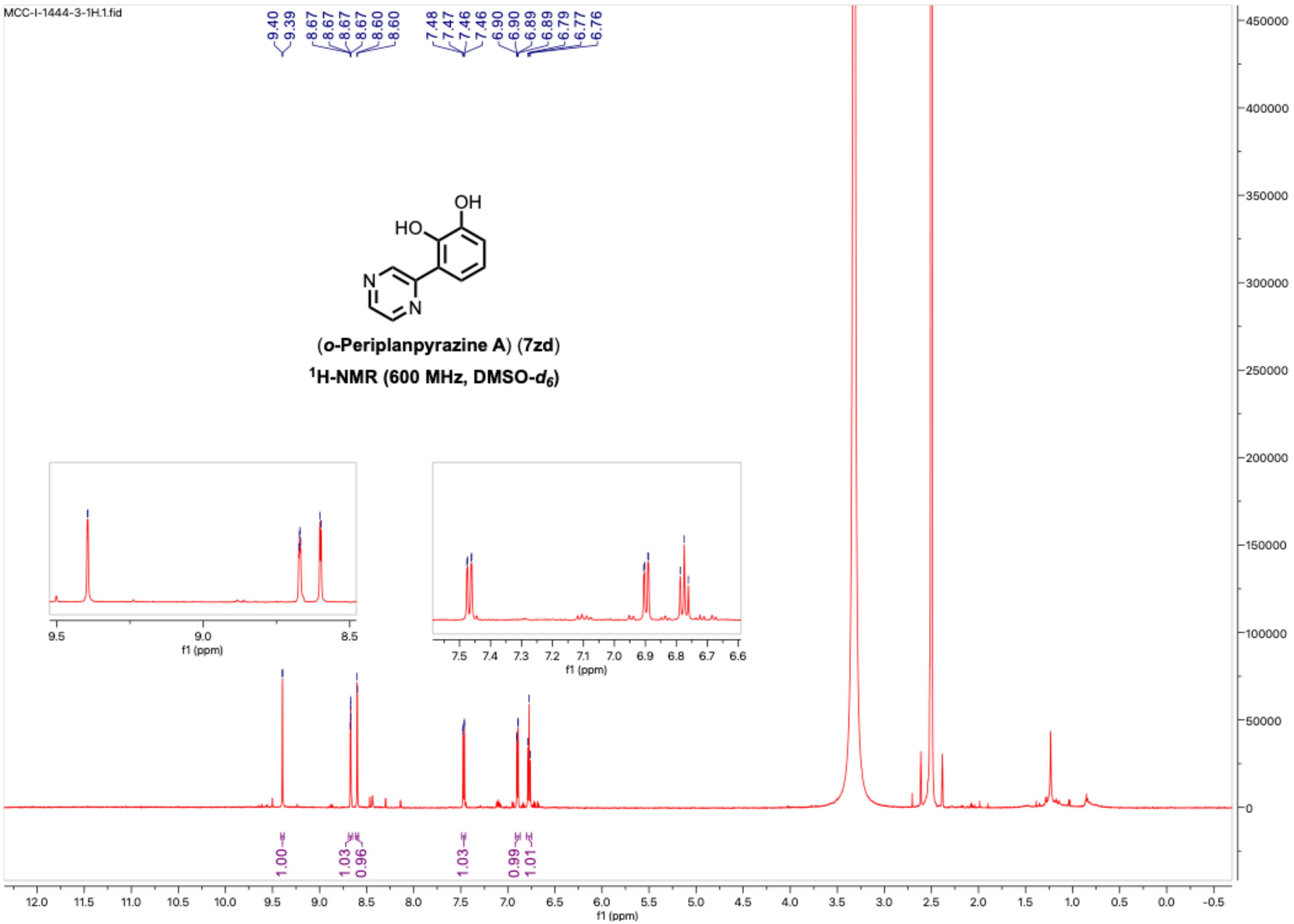
MCC-I-1444-3-1H.1.fid
9.40
9.39
8.67
8.67
8.67
8.67
8.60
8.60
7.48
7.47
7.46
7.46
6.90
6.90
6.89
6.89
6.79
6.77
6.76
OH
HO
N
N
(o-Periplanpyrazine A) (7zd)
1H-NMR (600 MHz, DMSO-d6)
1.00
1.03
0.96
1.03
0.99
1.01
f1 (ppm)

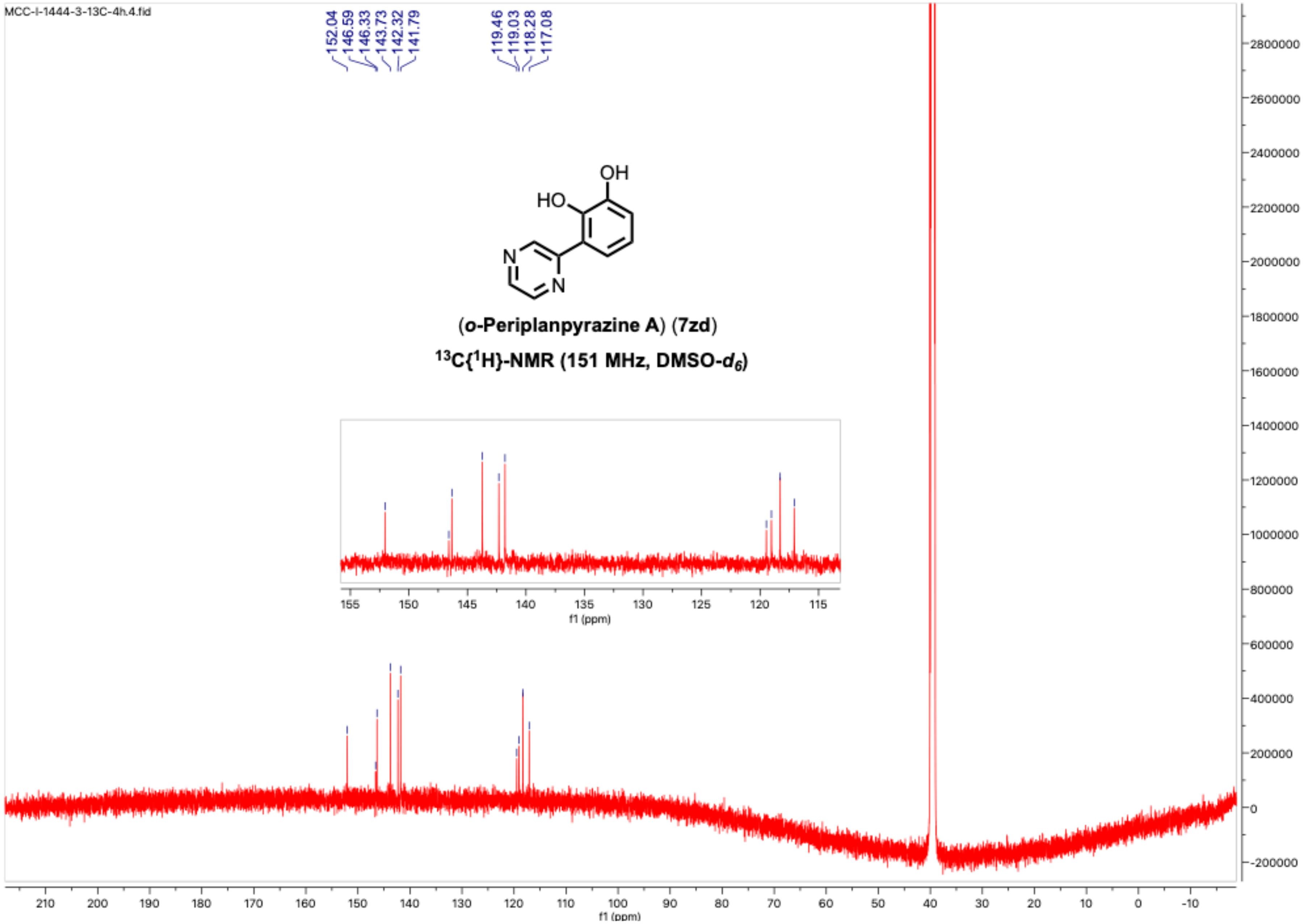
MCC-I-1444-3-13C-4h.4.fid
152.04
146.59
146.33
143.73
142.32
141.79
119.46
119.03
118.28
117.08
OH
HO
N
N
(o-Periplanpyrazine A) (7zd)
13C{1H}-NMR (151 MHz, DMSO-d6)
f1 (ppm)

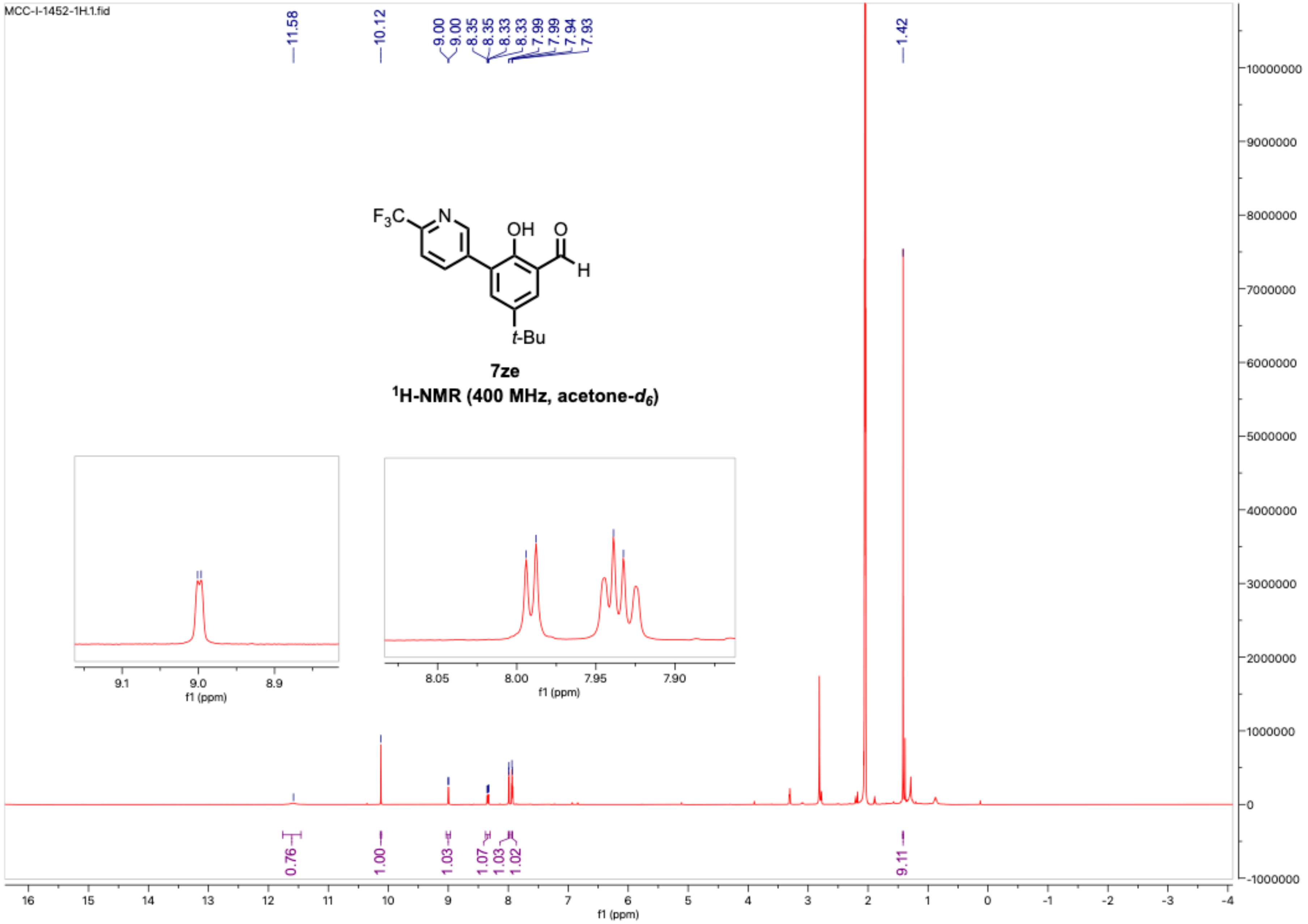
MCC-I-1452-1H.1.fid
11.58
10.12
9.00
9.00
8.35
8.35
8.33
8.33
7.99
7.99
7.94
7.93
1.42
7ze
^{1}H-NMR (400 MHz, acetone-d_6)
0.76
1.00
1.03
1.07
1.03
1.02
9.11
f1 (ppm)

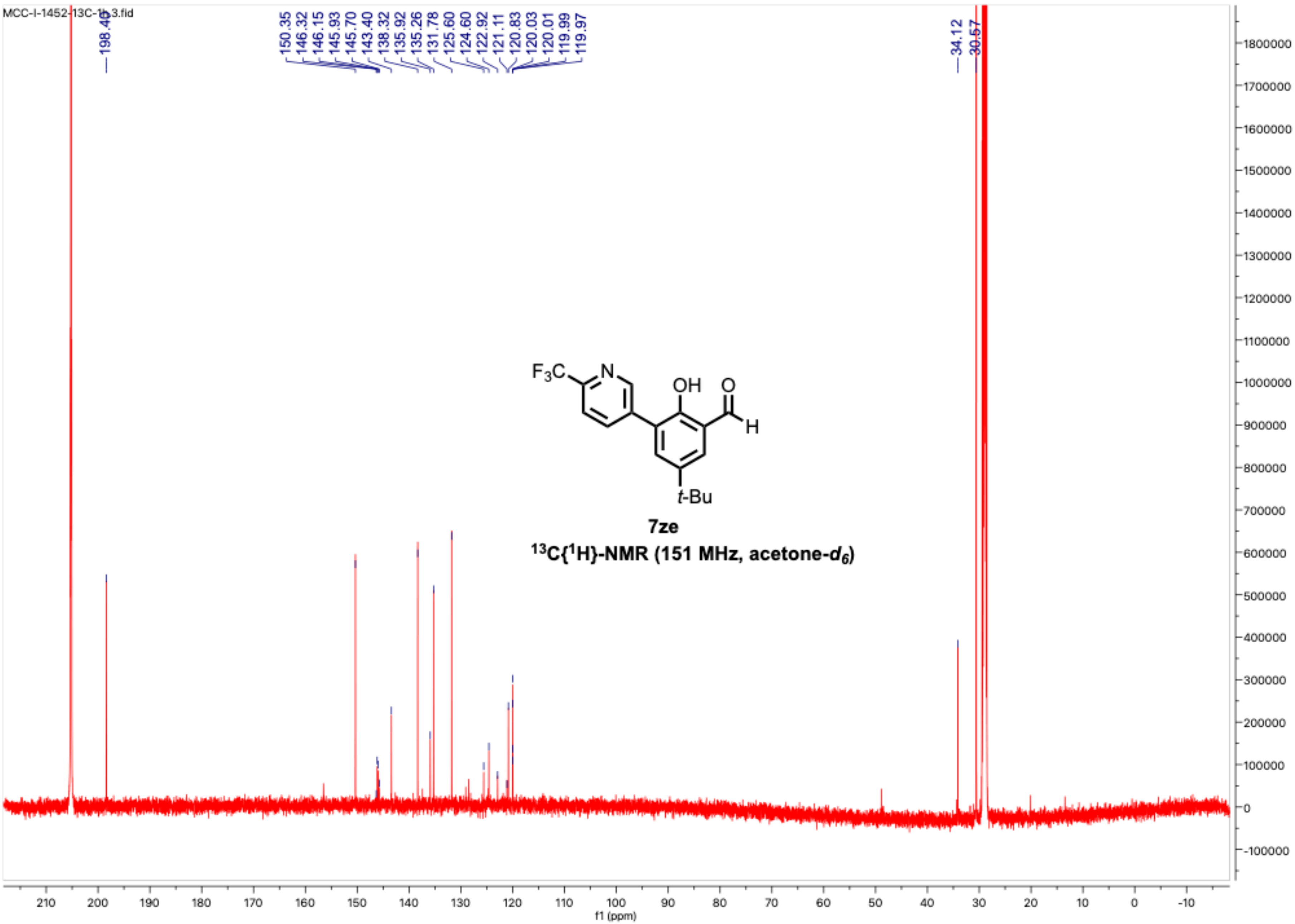

7ze

^{13}C{^{1}H}-NMR (151 MHz, acetone-d_6)

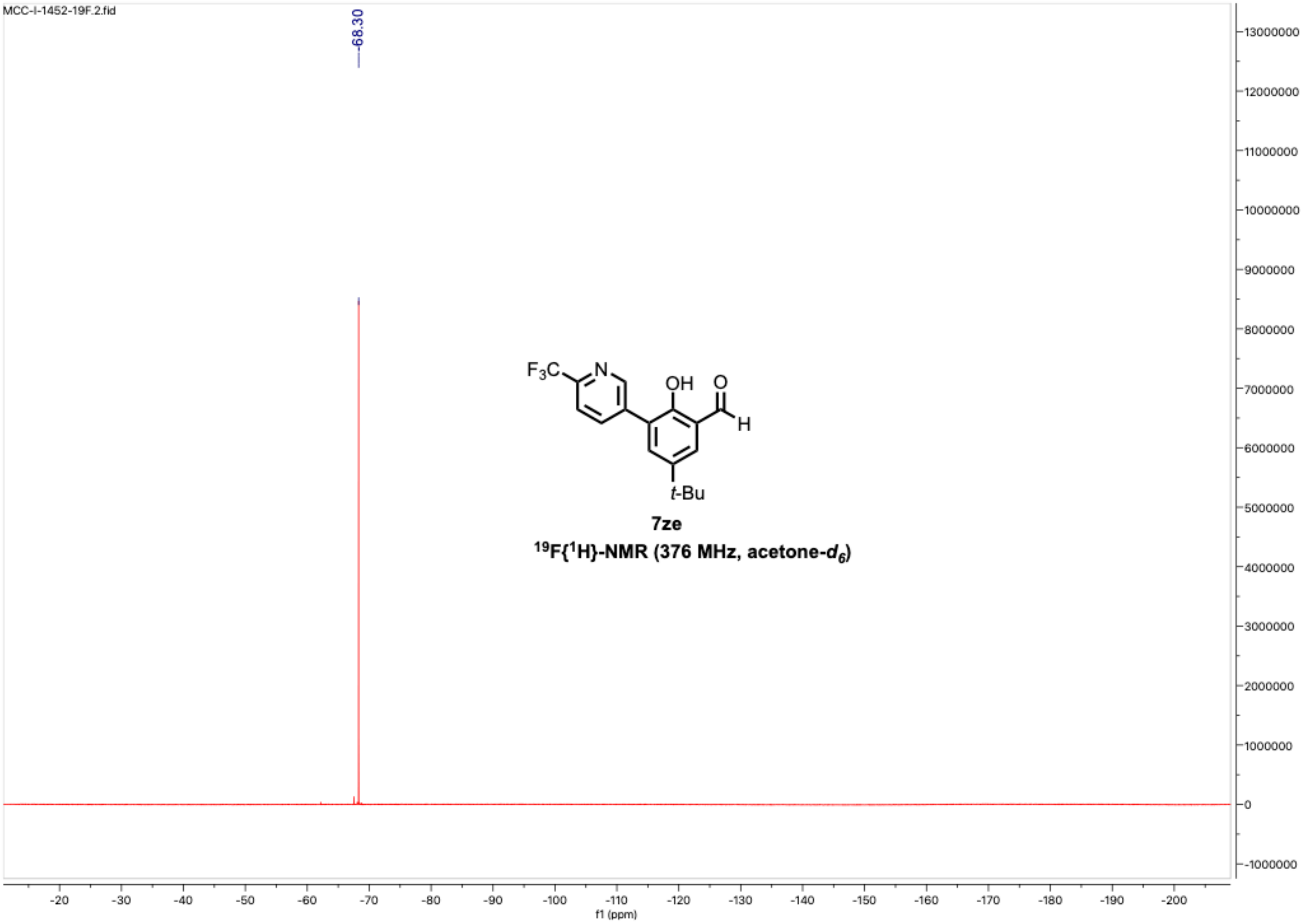
MCC-I-1452-19F.2.fid
-68.30
F3C
N
OH
O
H
t-Bu
7ze
19F{1H}-NMR (376 MHz, acetone-d6)
f1 (ppm)

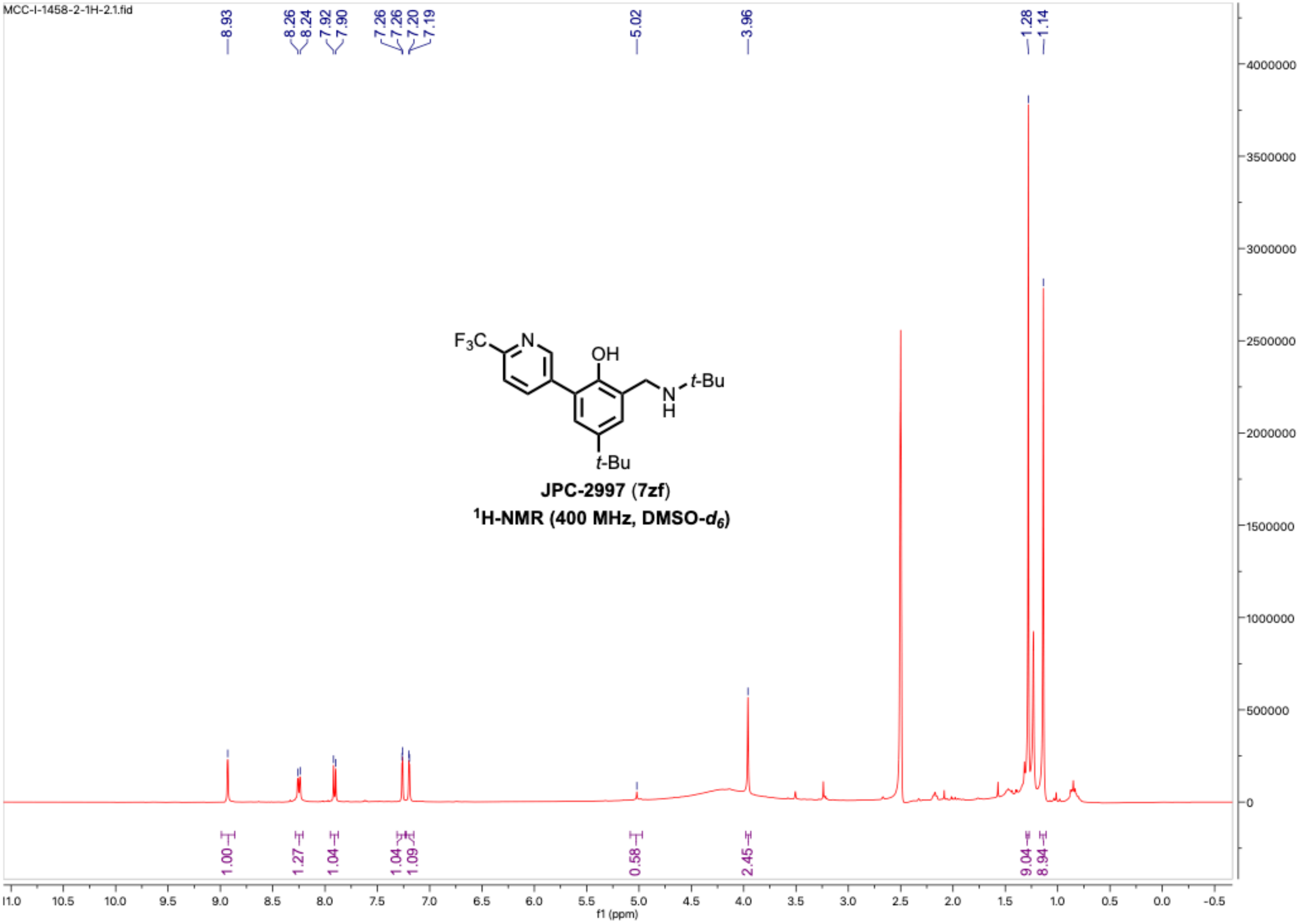
MCC-I-1458-2-1H-2.1.fid
8.93
8.26
8.24
7.92
7.90
7.26
7.26
7.20
7.19
5.02
3.96
1.28
1.14
F3C
N
OH
N
H
t-Bu
t-Bu
JPC-2997 (7zf)
1H-NMR (400 MHz, DMSO-d6)
1.00
1.27
1.04
1.04
1.09
0.58
2.45
9.04
8.94
f1 (ppm)

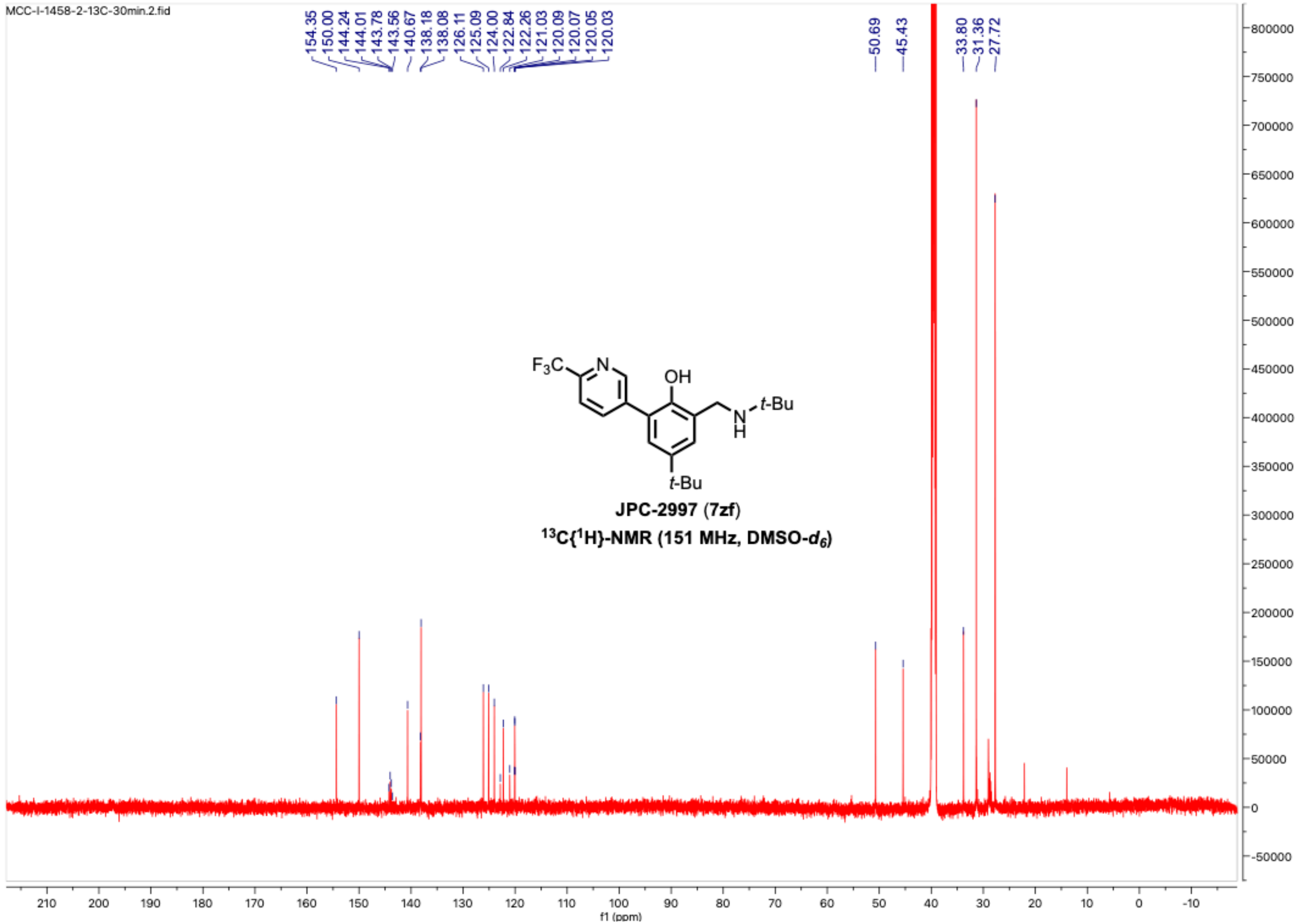
MCC-I-1458-2-13C-30min.2.fid
154.35
150.00
144.24
144.01
143.78
143.56
140.67
138.18
138.08
126.11
125.09
124.00
122.84
122.26
121.03
120.09
120.07
120.05
120.03
50.69
45.43
33.80
31.36
27.72
F3C
N
OH
N
H
t-Bu
t-Bu
JPC-2997 (7zf)
13C{1H}-NMR (151 MHz, DMSO-d6)
f1 (ppm)

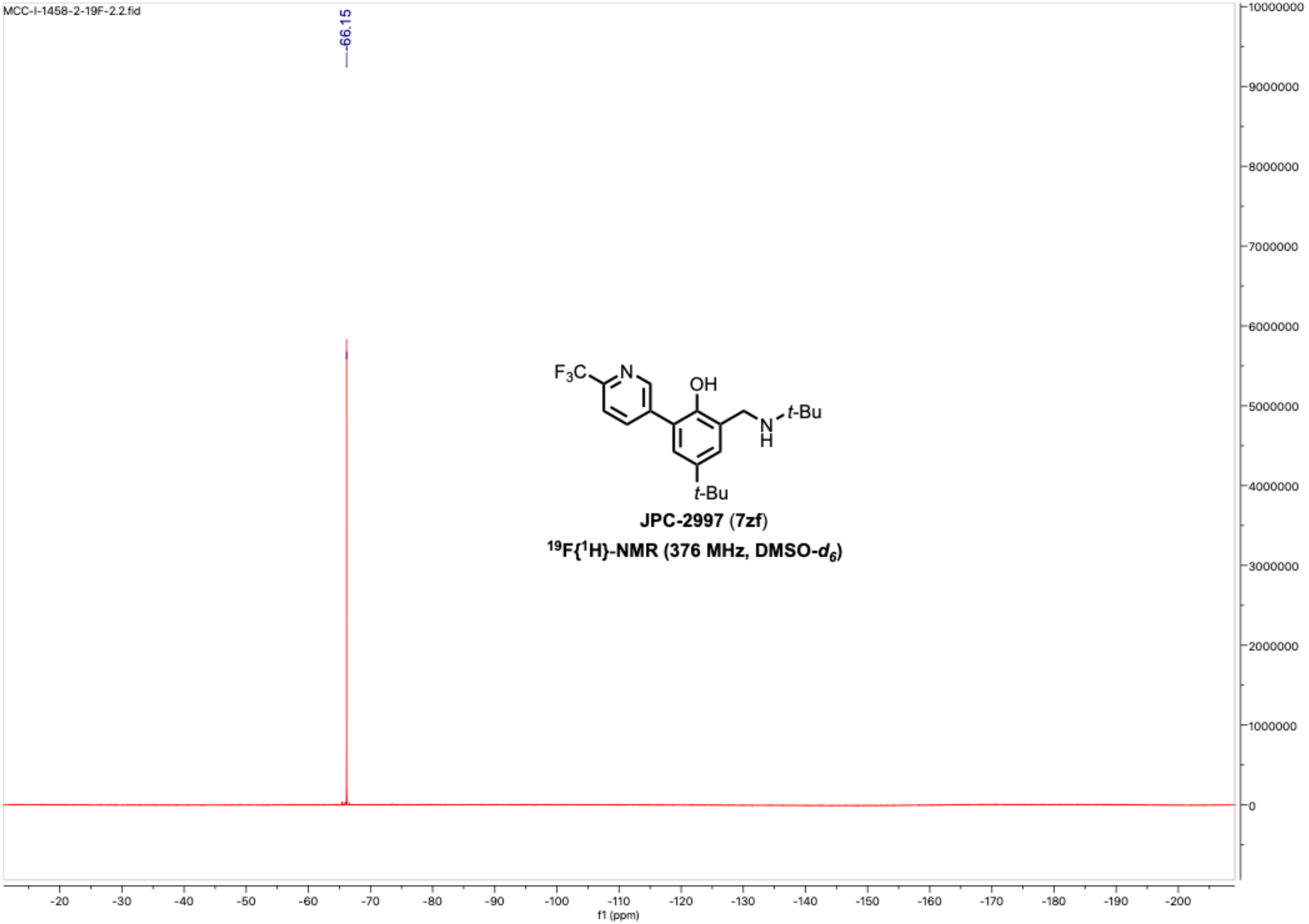

JPC-2997 (7zf)

$^{19}F\{^{1}H\}$-NMR (376 MHz, DMSO-*d*$_6$)

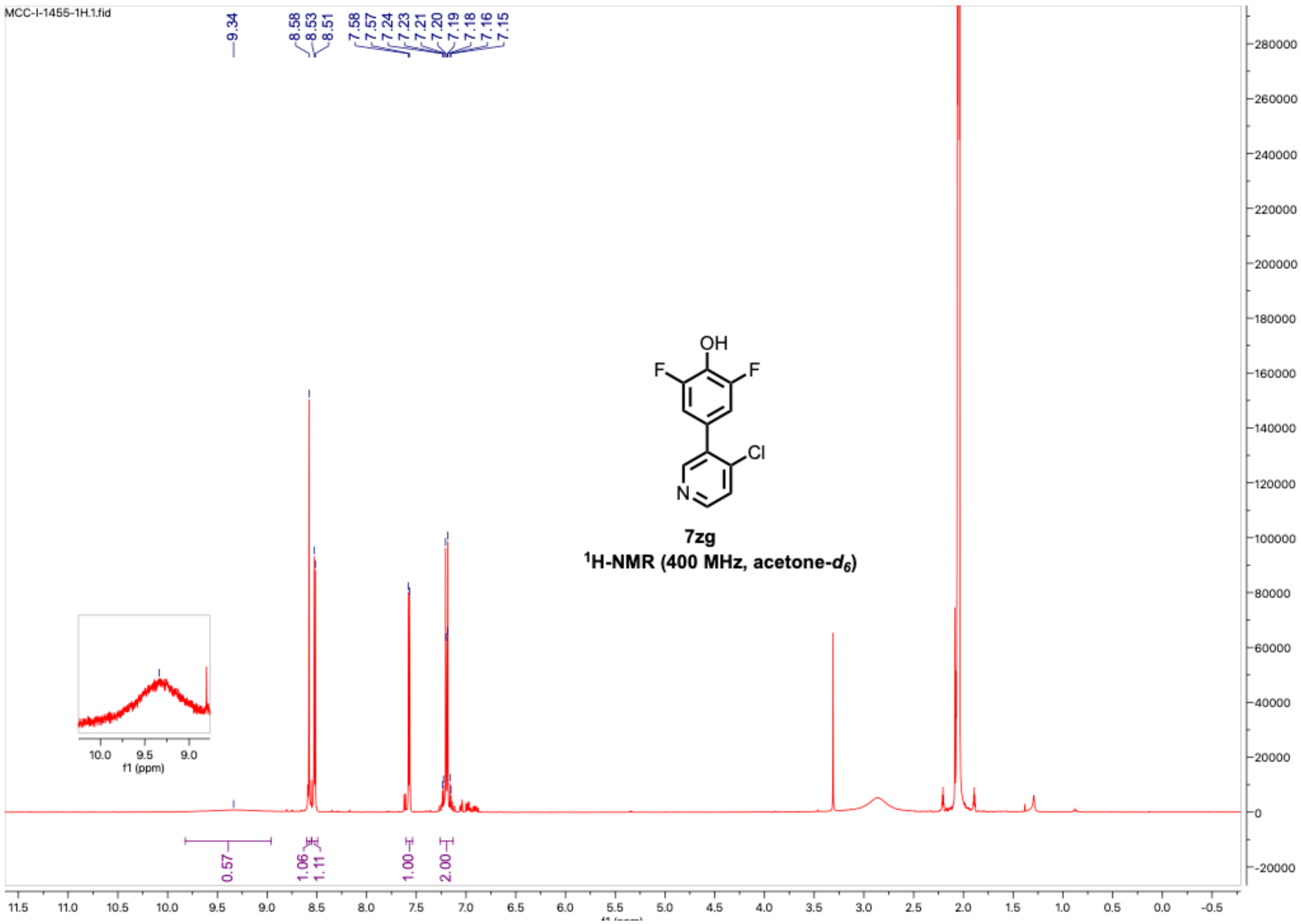
MCC-I-1455-1H.1.fid
9.34
8.58
8.53
8.51
7.58
7.57
7.24
7.23
7.21
7.20
7.19
7.18
7.16
7.15
OH
F
F
Cl
N
7zg
1H-NMR (400 MHz, acetone-d6)
0.57
1.06
1.11
1.00
2.00
f1 (ppm)

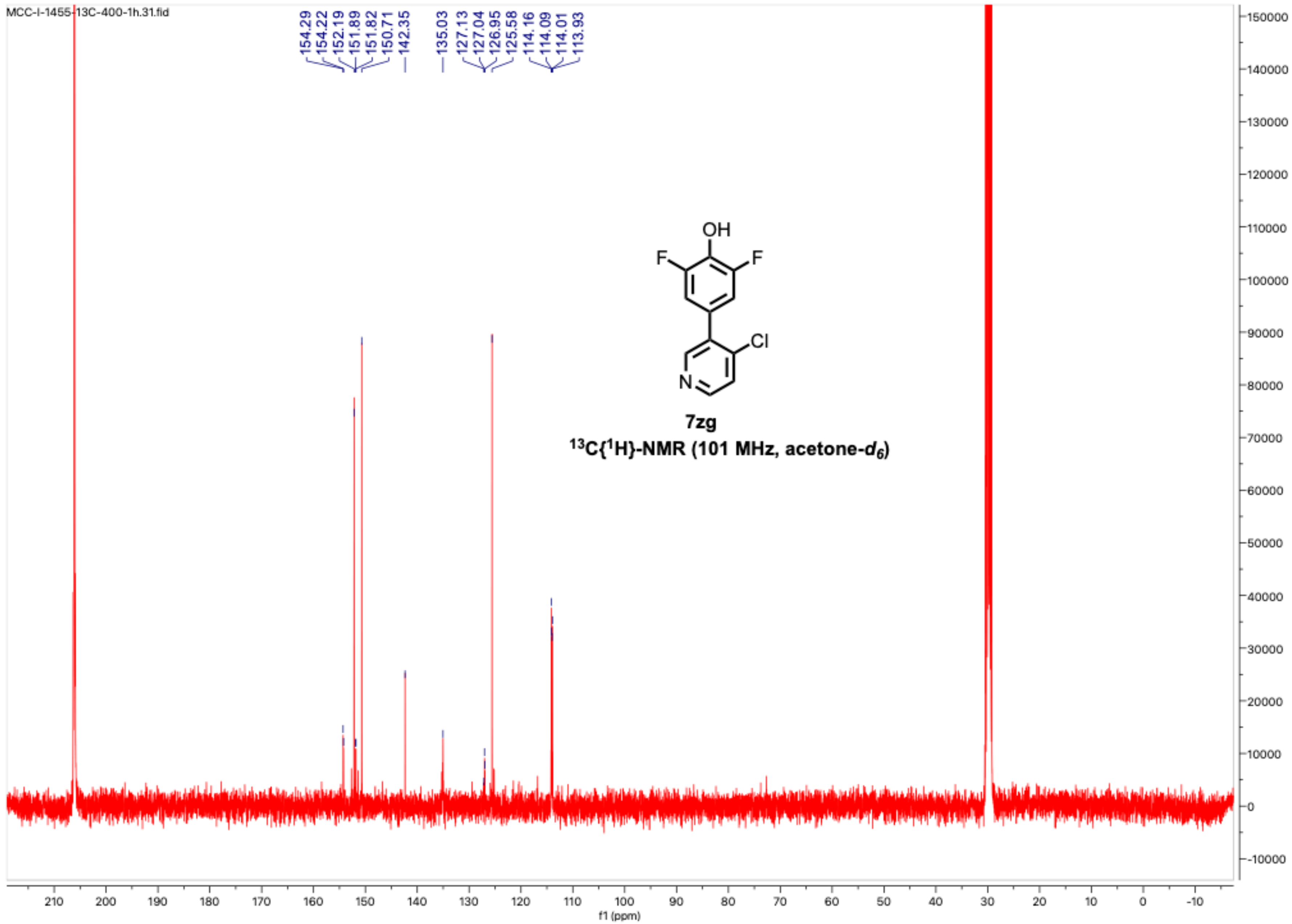
MCC-I-1455-13C-400-1h.31.fid
154.29
154.22
152.19
151.89
151.82
150.71
142.35
135.03
127.13
127.04
126.95
125.58
114.16
114.09
114.01
113.93
OH
F
F
Cl
N
7zg
13C{1H}-NMR (101 MHz, acetone-d6)
f1 (ppm)

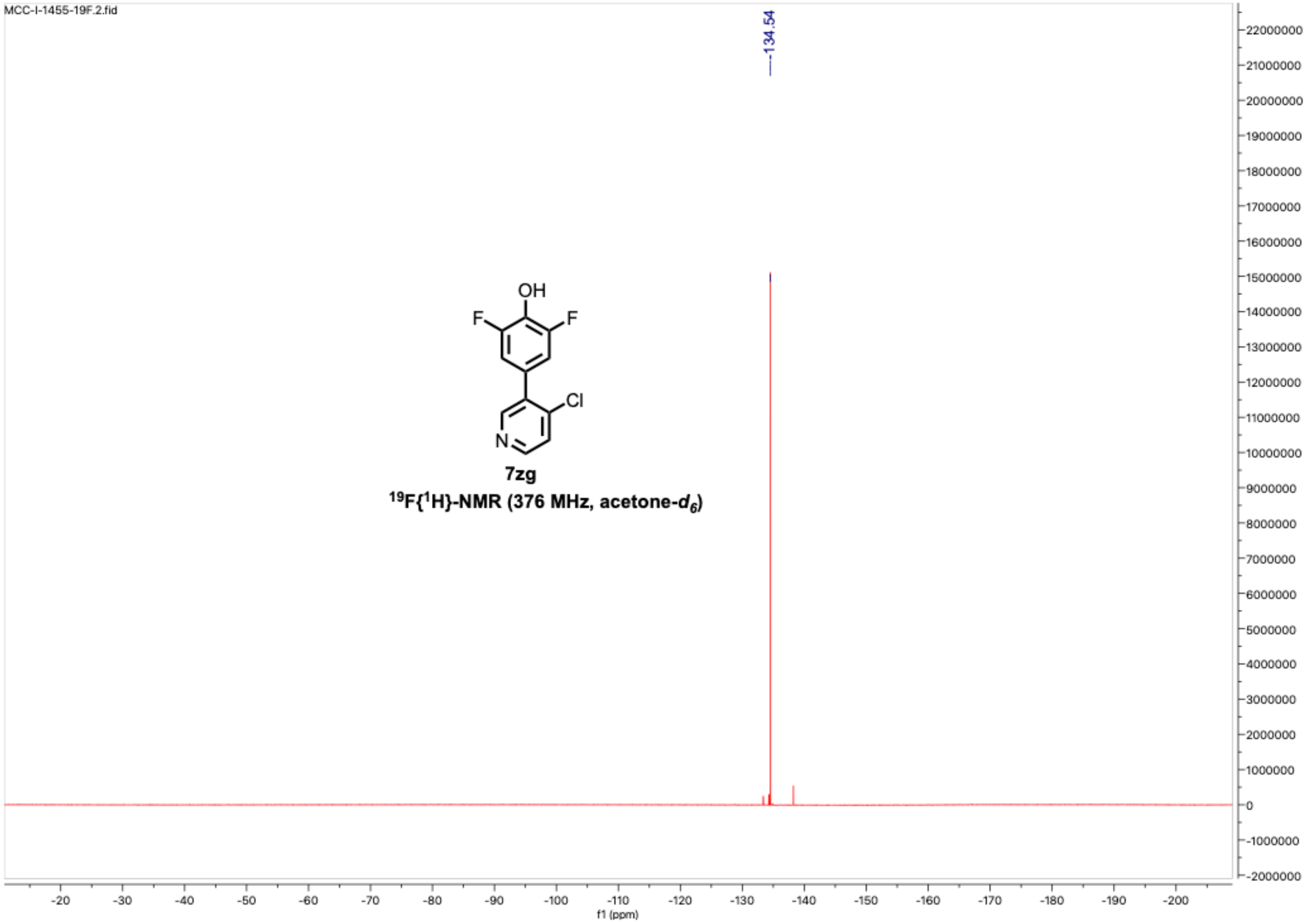
MCC-I-1455-19F.2.fid
-134.54
OH
F
F
Cl
N
7zg
19F{1H}-NMR (376 MHz, acetone-d6)
f1 (ppm)

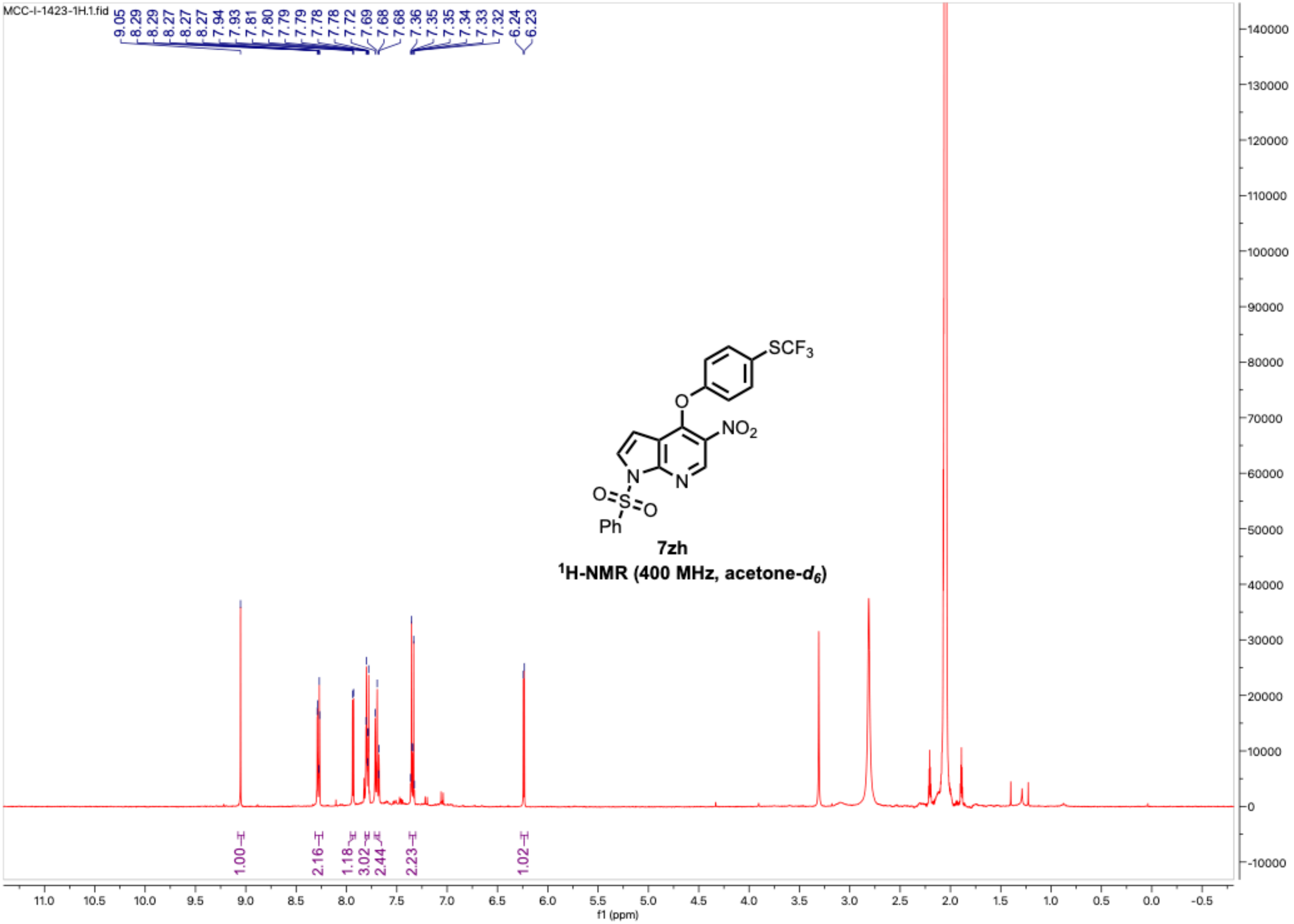
MCC-I-1423-1H.1.fid
9.05
8.29
8.29
8.27
8.27
8.27
7.94
7.93
7.81
7.80
7.79
7.79
7.78
7.78
7.72
7.69
7.68
7.68
7.36
7.35
7.35
7.34
7.33
7.32
6.24
6.23
SCF3
O
NO2
N
N
O
S
O
Ph
7zh
1H-NMR (400 MHz, acetone-d6)
1.00
2.16
1.18
3.02
2.44
2.23
1.02
f1 (ppm)

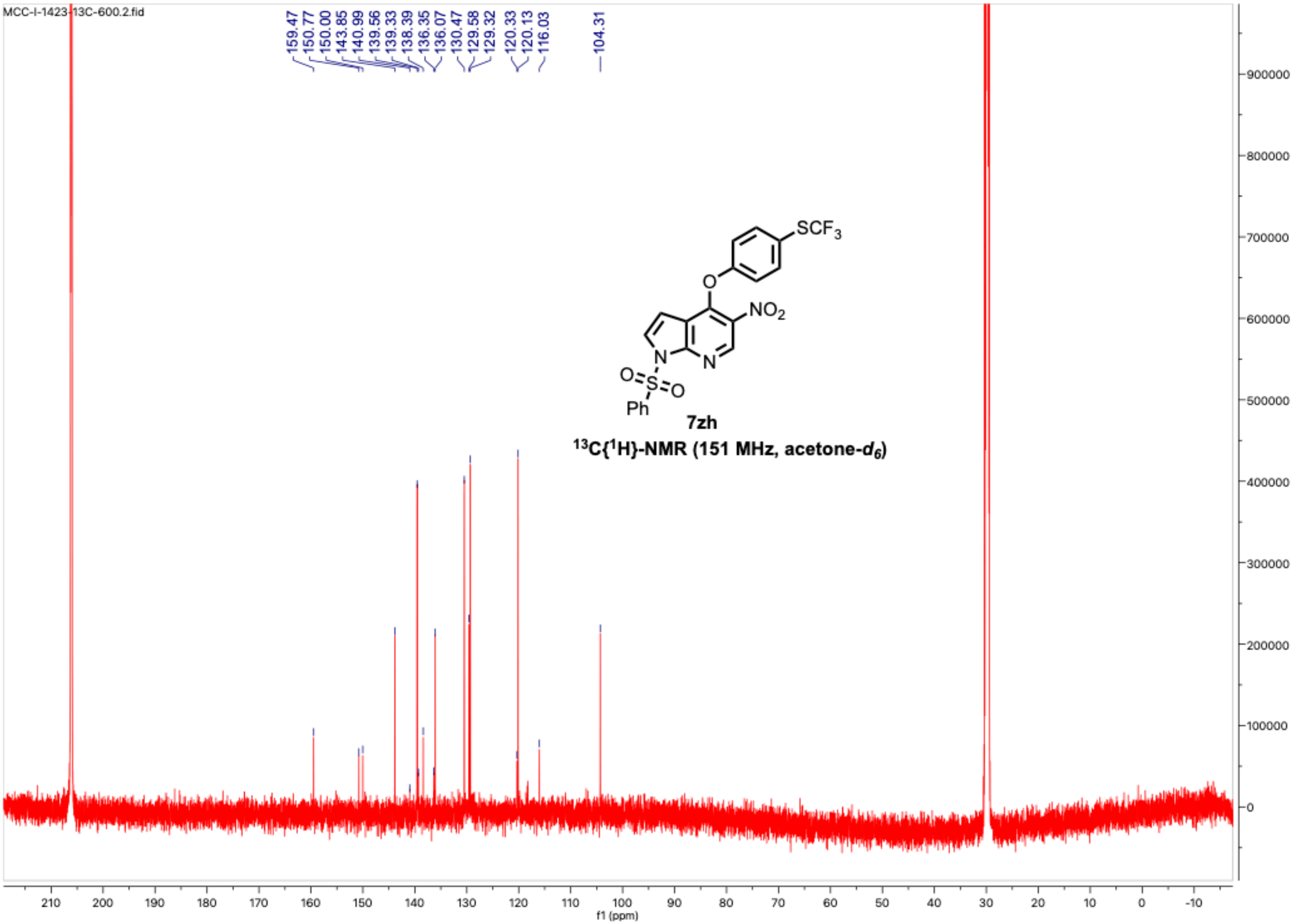

MCC-I-1423-13C-600.2.fid
159.47
150.77
150.00
143.85
140.99
139.56
139.33
138.39
136.35
136.07
130.47
129.58
129.32
120.33
120.13
116.03
104.31
SCF3
O
NO2
N
N
O=S=O
Ph
7zh
13C{1H}-NMR (151 MHz, acetone-d6)
f1 (ppm)

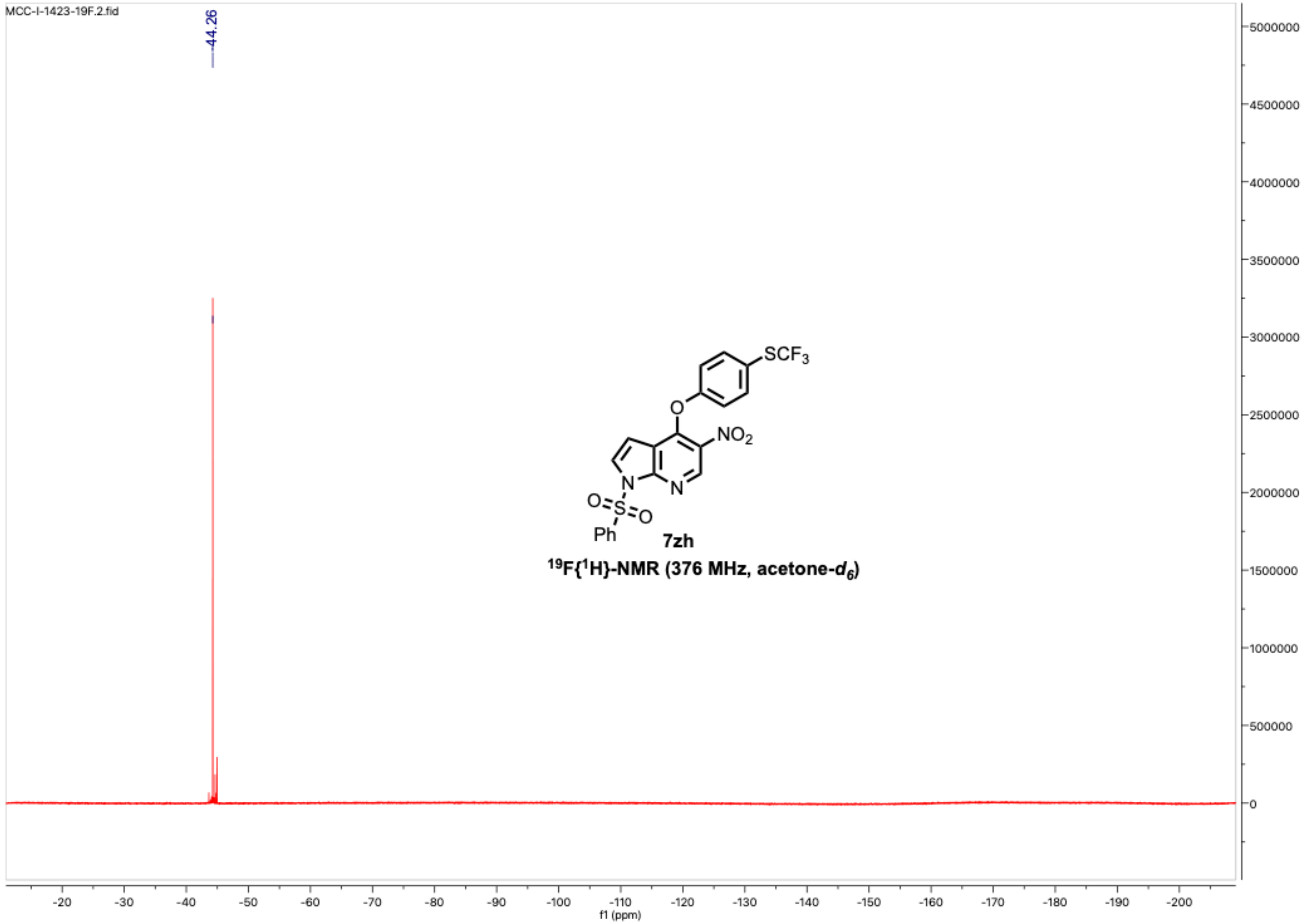
MCC-I-1423-19F.2.fid
-44.26
SCF3
NO2
N
N
O
O
S
O
Ph
7zh
19F{1H}-NMR (376 MHz, acetone-d6)
5000000
4500000
4000000
3500000
3000000
2500000
2000000
1500000
1000000
500000
0
-20
-30
-40
-50
-60
-70
-80
-90
-100
-110
-120
-130
-140
-150
-160
-170
-180
-190
-200
f1 (ppm)

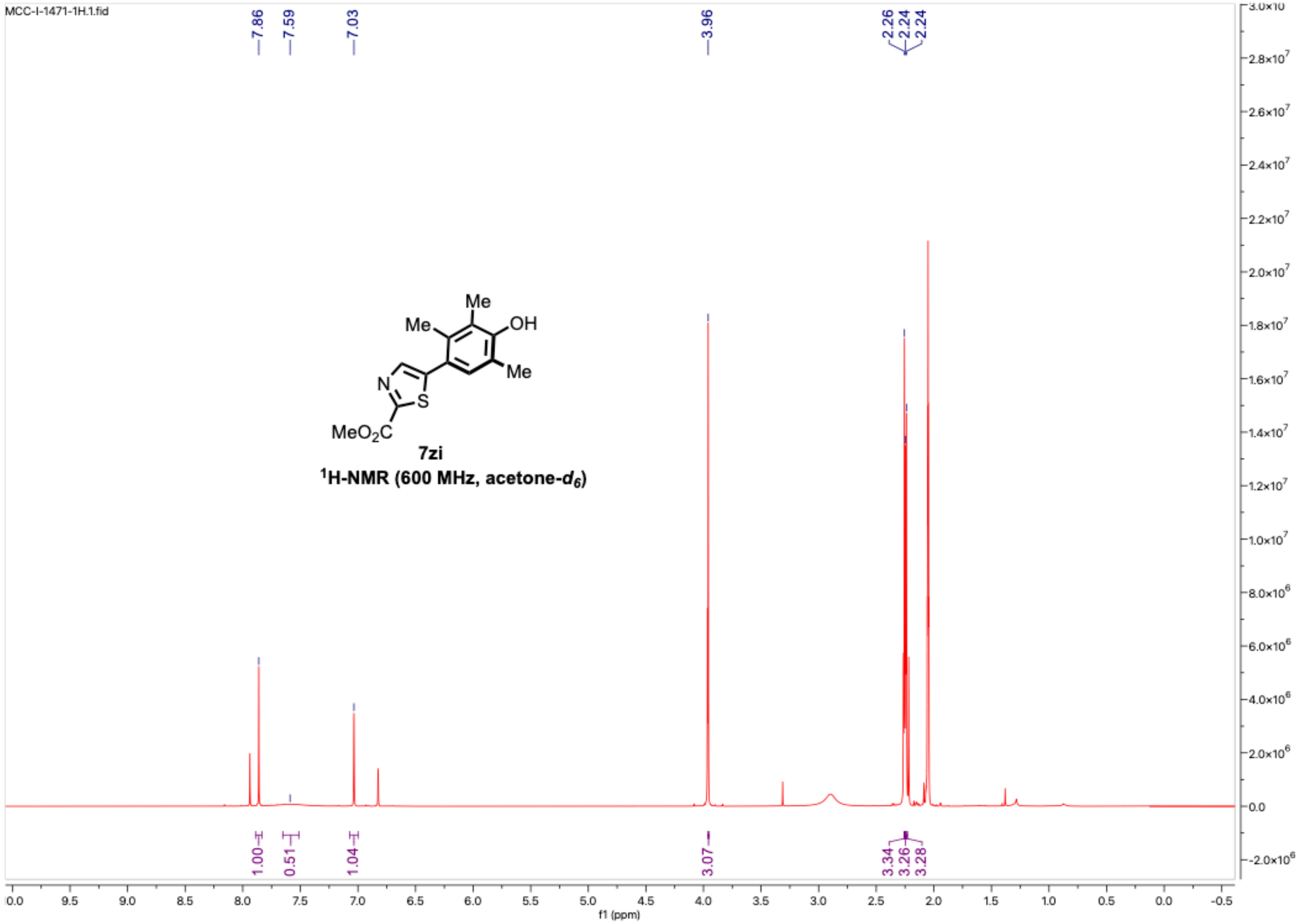

7zi

^{1}H-NMR (600 MHz, acetone-d_6)

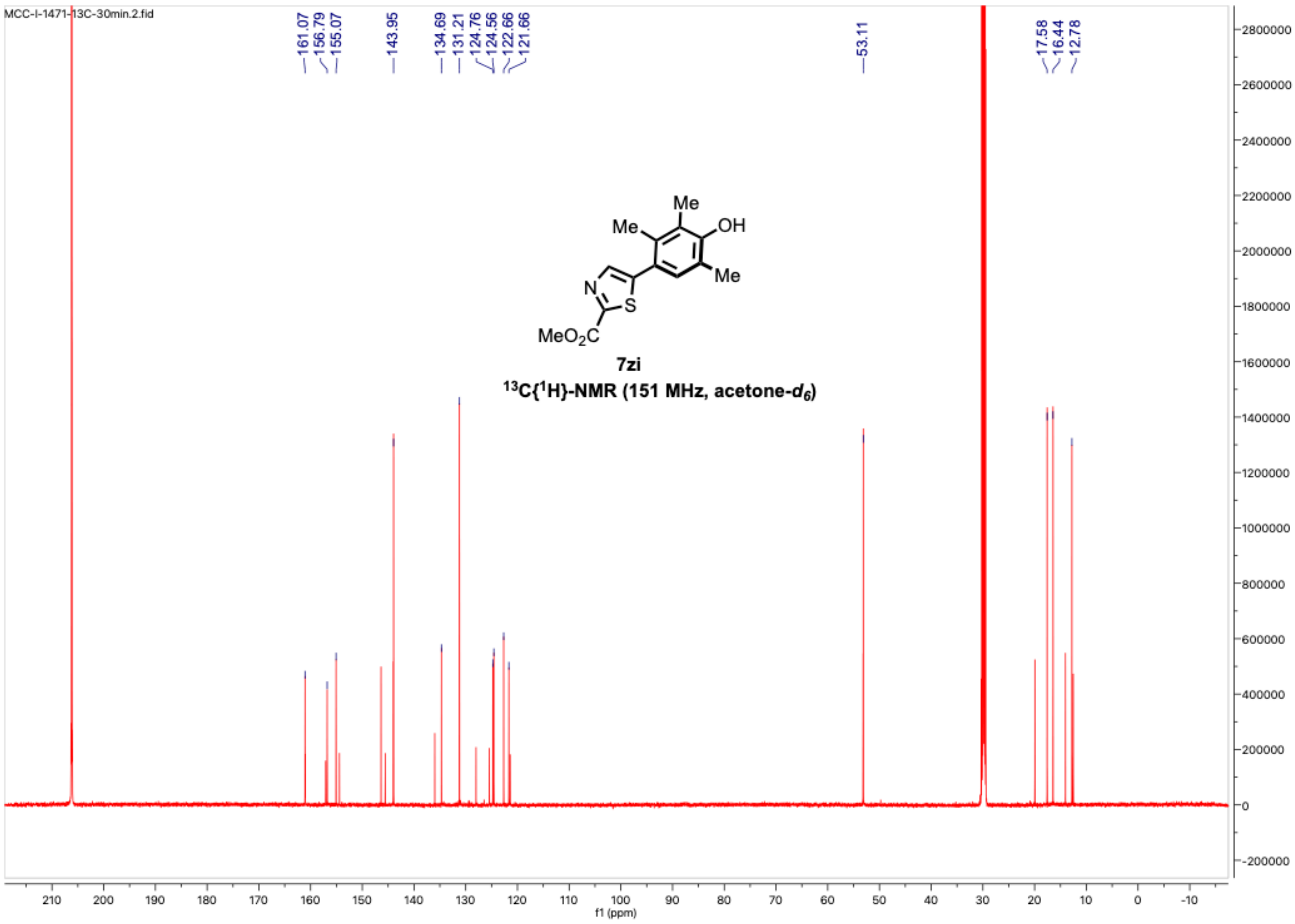
MCC-I-1471-13C-30min.2.fid
161.07
156.79
155.07
143.95
134.69
131.21
124.76
124.56
122.66
121.66
53.11
17.58
16.44
12.78
Me
Me
OH
Me
N
S
MeO2C
7zi
13C{1H}-NMR (151 MHz, acetone-d6)
f1 (ppm)

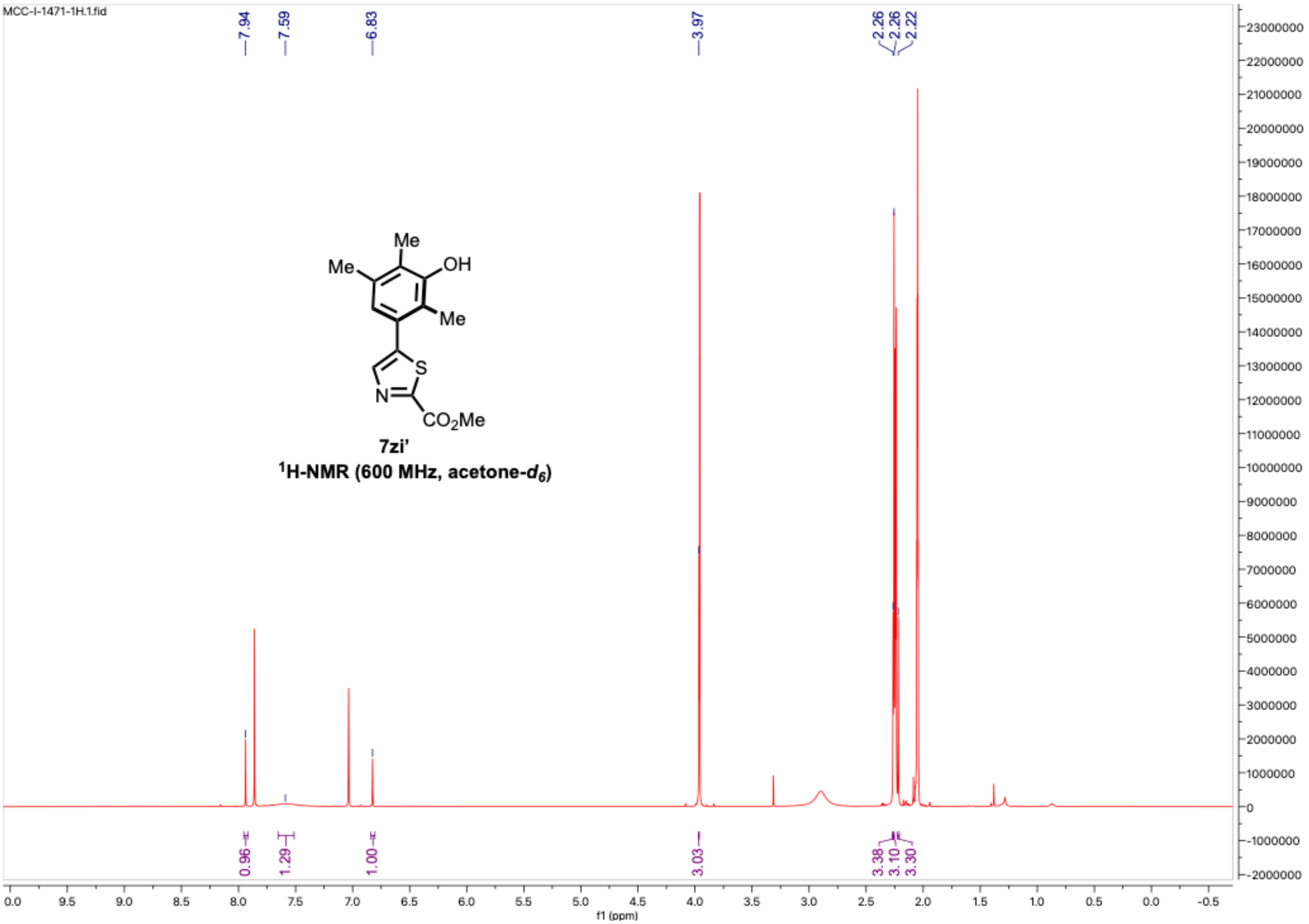
MCC-I-1471-1H.1.fid
7.94
7.59
6.83
3.97
2.26
2.26
2.22
Me
Me
OH
Me
S
N
CO2Me
7zi'
1H-NMR (600 MHz, acetone-d6)
0.96
1.29
1.00
3.03
3.38
3.10
3.30
f1 (ppm)

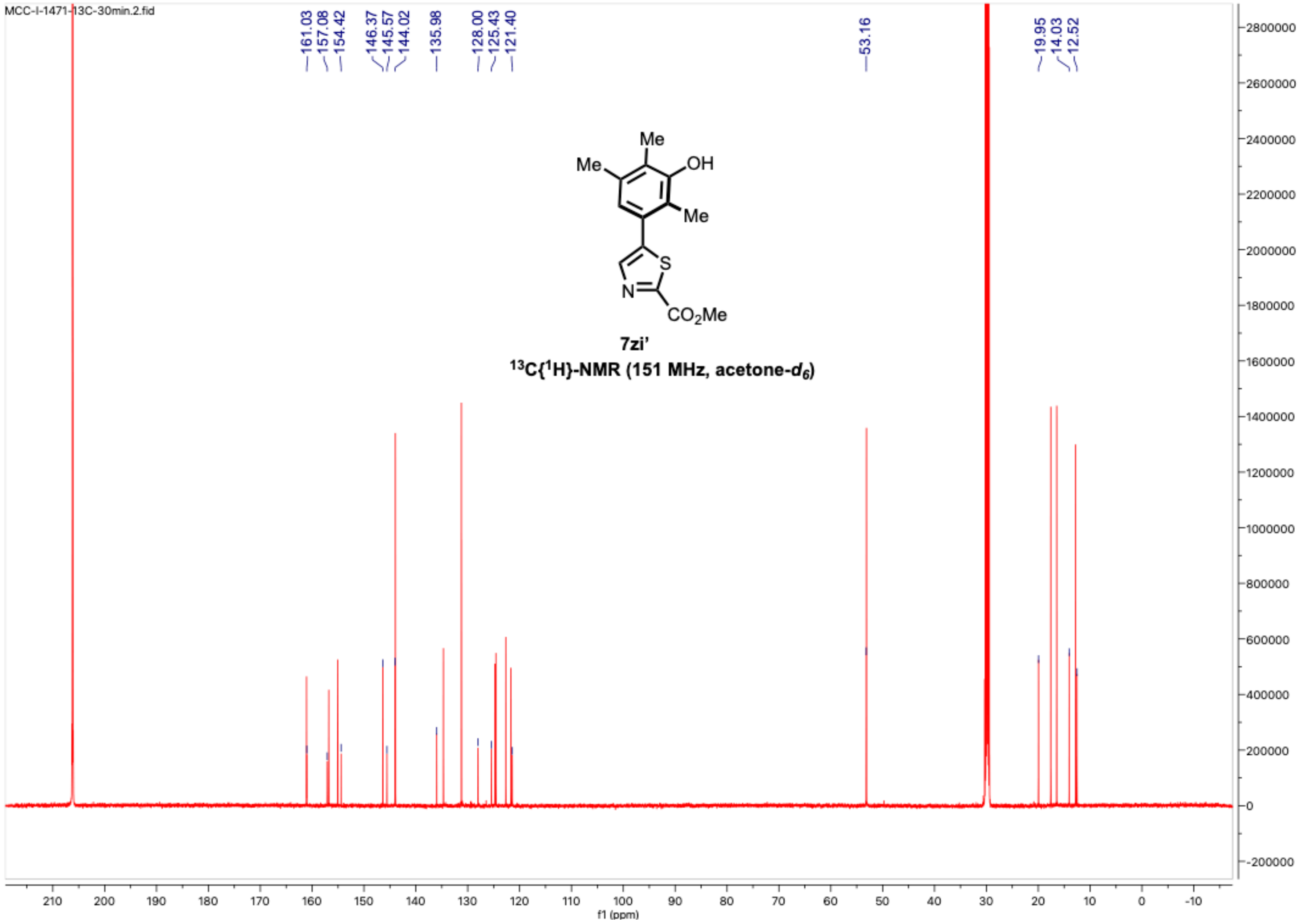
MCC-I-1471-13C-30min.2.fid
161.03
157.08
154.42
146.37
145.57
144.02
135.98
128.00
125.43
121.40
53.16
19.95
14.03
12.52
Me
Me
OH
Me
S
N
CO2Me
7zi'
13C{1H}-NMR (151 MHz, acetone-d6)
f1 (ppm)

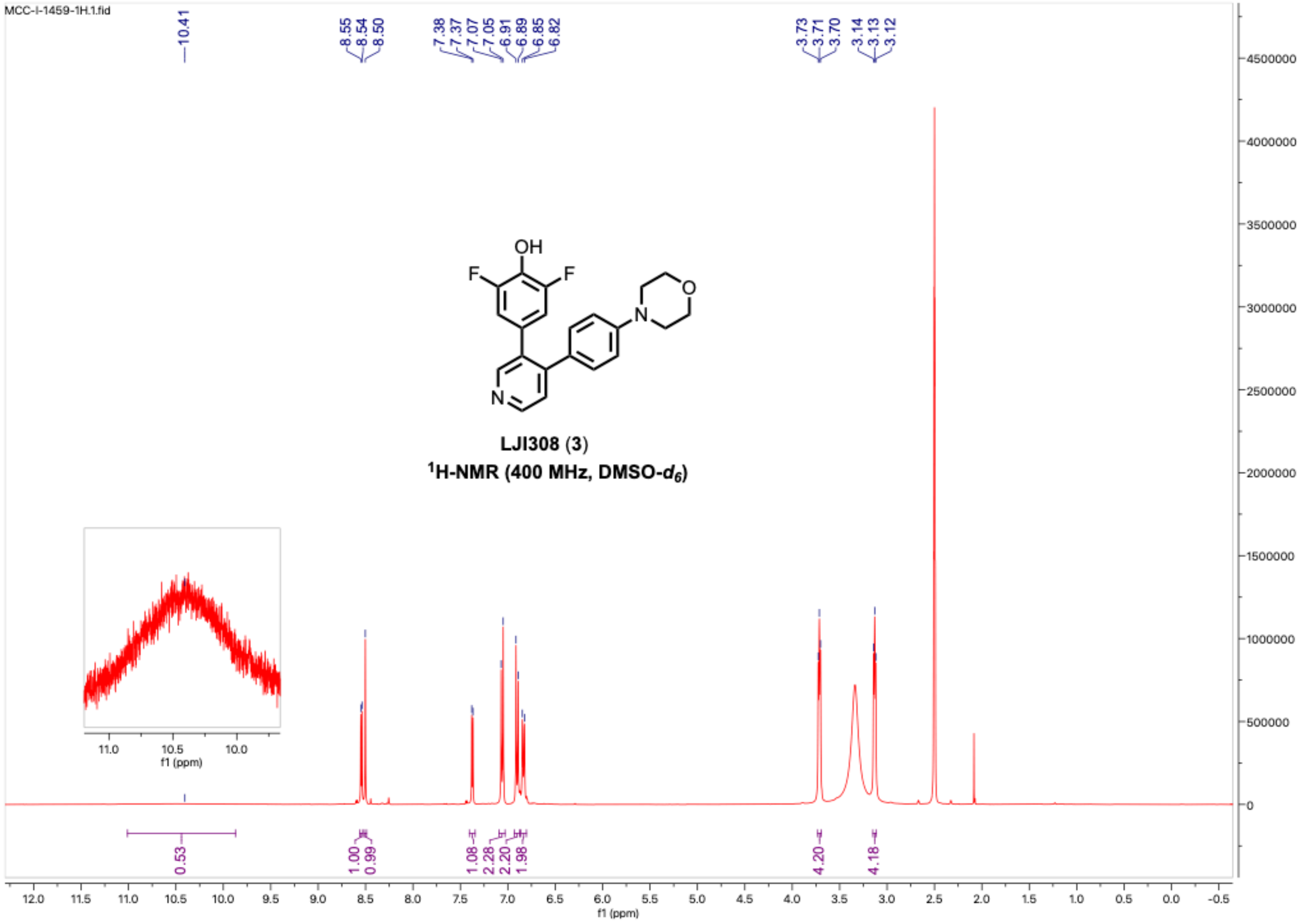
MCC-I-1459-1H.1.fid
LJI308 (3)
^{1}H-NMR (400 MHz, DMSO-d_6)
f1 (ppm)
