# Supplementary material for: Photocatalytic Cross‐Coupling of Phenols and Heteroaryl Halides With Machine Learning‐Guided Reaction Prediction
Source: Angew Chem Int Ed Engl. 2026 Mar 5;65(16):e8222003. doi: 10.1002/anie.8222003 (PMC13080434; doi:10.1002/anie.8222003)
Supplement: Supplementary file 2 — Supporting File 2: anie71621‐sup‐0002‐Data.zip. [file ANIE-65-e8222003-s001.zip › anie71621-sup-0002-Data/NMR FAIR Data 2.zip!/NMR FAIR Data/7za/1H NMR/MCC-I-1347-3-1H/1/pdata/1/email_MCC-I-1347-3-1H_1_1.pdf]

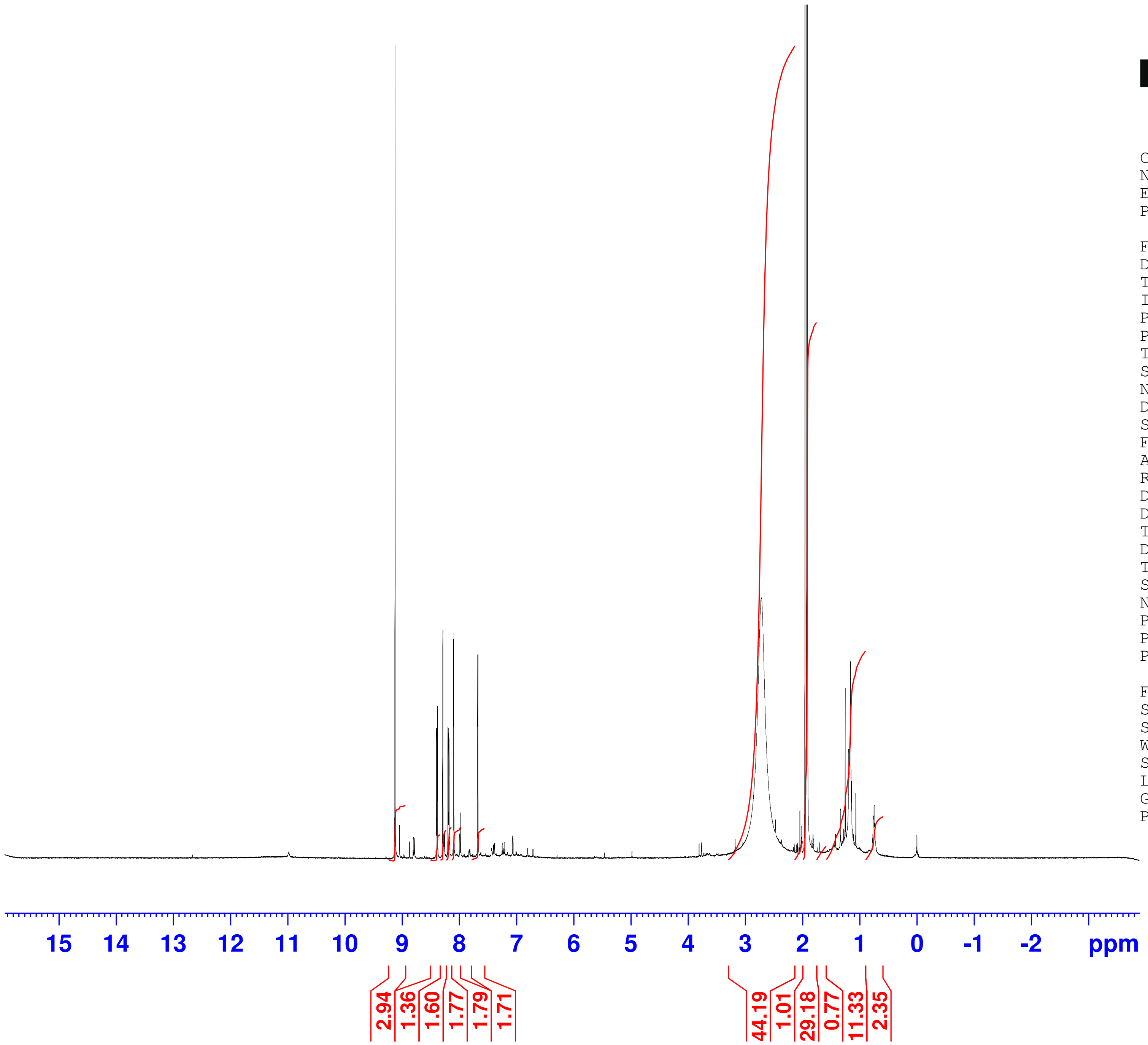

BRUKER

Current Data Parameters
NAME MCC-I-1347-3-1H
EXPNO 1
PROCNO 1

F2 - Acquisition Parameters

Date_	20250709	
Time	11.14	h
INSTRUM	Avance 4 NEO 600	
PROBHD	Z168773_0007 (	
PULPROG	zg30	
TD	65536	
SOLVENT	Acetone	
NS	16	
DS	2	
SWH	11904.762	Hz
FIDRES	0.363304	Hz
AQ	2.7525120	sec
RG	101	
DW	42.000	usec
DE	24.20	usec
TE	298.0	K
D1	1.00000000	sec
TD0	1	
SFO1	600.1337058	MHz
NUC1	1H	
P0	4.00	usec
P1	12.00	usec
PLW1	18.62700081	W

F2 - Processing parameters

SI	65536	
SF	600.1300882	MHz
WDW	EM	
SSB	0	
LB	0.30	Hz
GB	0	
PC	1.00	
