# Supplementary material for: Photocatalytic Cross‐Coupling of Phenols and Heteroaryl Halides With Machine Learning‐Guided Reaction Prediction
Source: Angew Chem Int Ed Engl. 2026 Mar 5;65(16):e8222003. doi: 10.1002/anie.8222003 (PMC13080434; doi:10.1002/anie.8222003)
Supplement: Supplementary file 2 — Supporting File 2: anie71621‐sup‐0002‐Data.zip. [file ANIE-65-e8222003-s001.zip › anie71621-sup-0002-Data/NMR FAIR Data 2.zip!/NMR FAIR Data/7za/13C NMR/MCC-I-1347-acetone-13C/6/pdata/1/email_MCC-I-1347-acetone-13C_6_1.pdf]

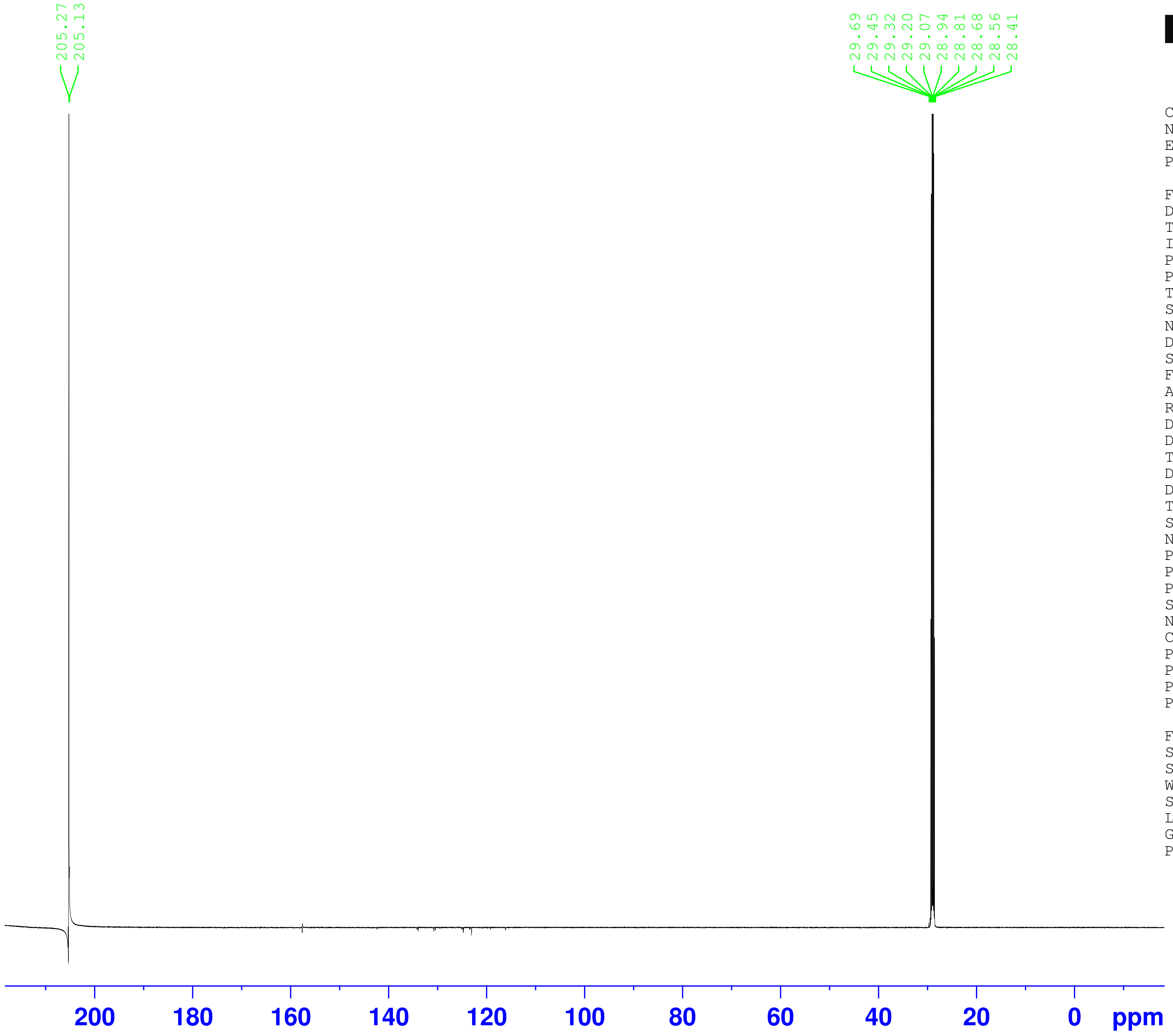

BRUKER

```
Current Data Parameters
NAME       MCC-I-1347-acetone-13C
EXPNO                        6
PROCNO                       1

F2 - Acquisition Parameters
Date_               20250702
Time                   21.07 h
INSTRUM   Avance 4 NEO 600
PROBHD    Z168773_0007 (
PULPROG               zgpg30
TD                     65536
SOLVENT              Acetone
NS                      1024
DS                         4
SWH              35714.286 Hz
FIDRES            1.089913 Hz
AQ               0.9175040 sec
RG                       101
DW                    14.000 usec
DE                     18.00 usec
TE                     298.0 K
D1                2.00000000 sec
D11               0.03000000 sec
TD0                        1
SFO1           150.9178988 MHz
NUC1                     13C
P0                      3.50 usec
P1                     10.50 usec
PLW1             68.97799683 W
SFO2           600.1324005 MHz
NUC2                      1H
CPDPRG[2             waltz65
PCPD2                  70.00 usec
PLW2             18.62700081 W
PLW12             0.54741001 W
PLW13             0.27533999 W

F2 - Processing parameters
SI                     32768
SF             150.9028085 MHz
WDW                       EM
SSB                        0
LB                      1.00 Hz
GB                         0
PC                      1.40
```
