# Supplementary material for: Photocatalytic Cross‐Coupling of Phenols and Heteroaryl Halides With Machine Learning‐Guided Reaction Prediction
Source: Angew Chem Int Ed Engl. 2026 Mar 5;65(16):e8222003. doi: 10.1002/anie.8222003 (PMC13080434; doi:10.1002/anie.8222003)
Supplement: Supplementary file 2 — Supporting File 2: anie71621‐sup‐0002‐Data.zip. [file ANIE-65-e8222003-s001.zip › anie71621-sup-0002-Data/NMR FAIR Data 2.zip!/NMR FAIR Data/7d'/13C NMR/MCC-I-1194-B-P2-13C/13/pdata/1/email_MCC-I-1194-B-P2-13C_13_1.pdf]

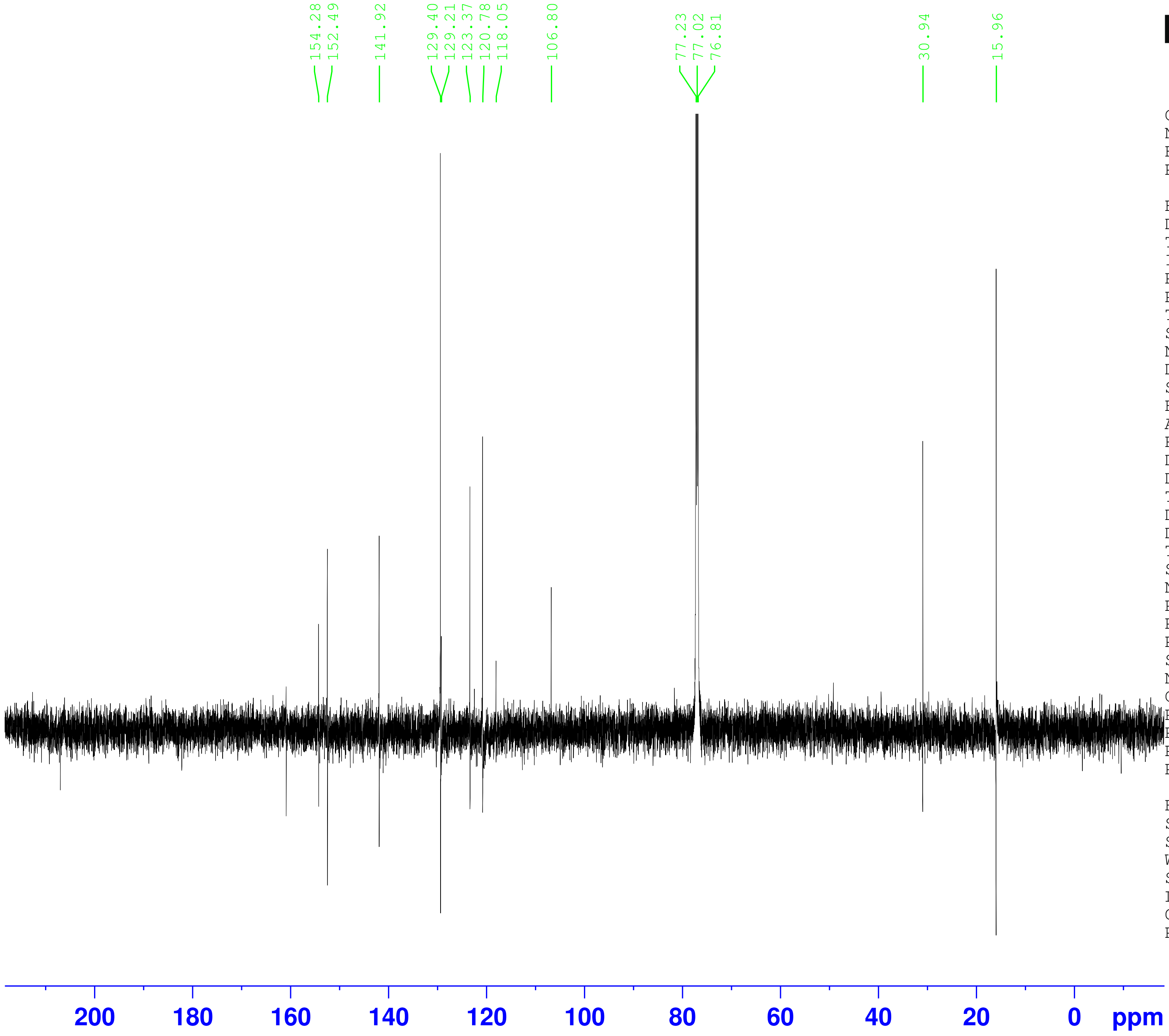

BRUKER

```
Current Data Parameters
NAME      MCC-I-1194-B-P2-13C
EXPNO                      13
PROCNO                      1

F2 - Acquisition Parameters
Date_              20241026
Time                   8.00 h
INSTRUM   Avance 4 NEO 600
PROBHD    Z168773_0007 (
PULPROG            zgpg30
TD                  65536
SOLVENT             CDCl3
NS                    512
DS                      4
SWH             35714.286 Hz
FIDRES           1.089913 Hz
AQ              0.9175040 sec
RG                    101
DW                 14.000 usec
DE                  18.00 usec
TE                  298.0 K
D1             2.00000000 sec
D11            0.03000000 sec
TD0                     1
SFO1          150.9178988 MHz
NUC1                  13C
P0                   3.50 usec
P1                  10.50 usec
PLW1          68.97799683 W
SFO2          600.1324005 MHz
NUC2                   1H
CPDPRG[2          waltz65
PCPD2               70.00 usec
PLW2          18.62700081 W
PLW12          0.54741001 W
PLW13          0.27533999 W

F2 - Processing parameters
SI                  32768
SF            150.9028085 MHz
WDW                    EM
SSB                     0
LB                   1.00 Hz
GB                      0
PC                   1.40
```
