# Supplementary material for: Photocatalytic Cross‐Coupling of Phenols and Heteroaryl Halides With Machine Learning‐Guided Reaction Prediction
Source: Angew Chem Int Ed Engl. 2026 Mar 5;65(16):e8222003. doi: 10.1002/anie.8222003 (PMC13080434; doi:10.1002/anie.8222003)
Supplement: Supplementary file 2 — Supporting File 2: anie71621‐sup‐0002‐Data.zip. [file ANIE-65-e8222003-s001.zip › anie71621-sup-0002-Data/NMR FAIR Data 2.zip!/NMR FAIR Data/7d/13C NMR/MCC-I-1194-B-P1-13C/1/pdata/1/email_MCC-I-1194-B-P1-13C_1_1.pdf]

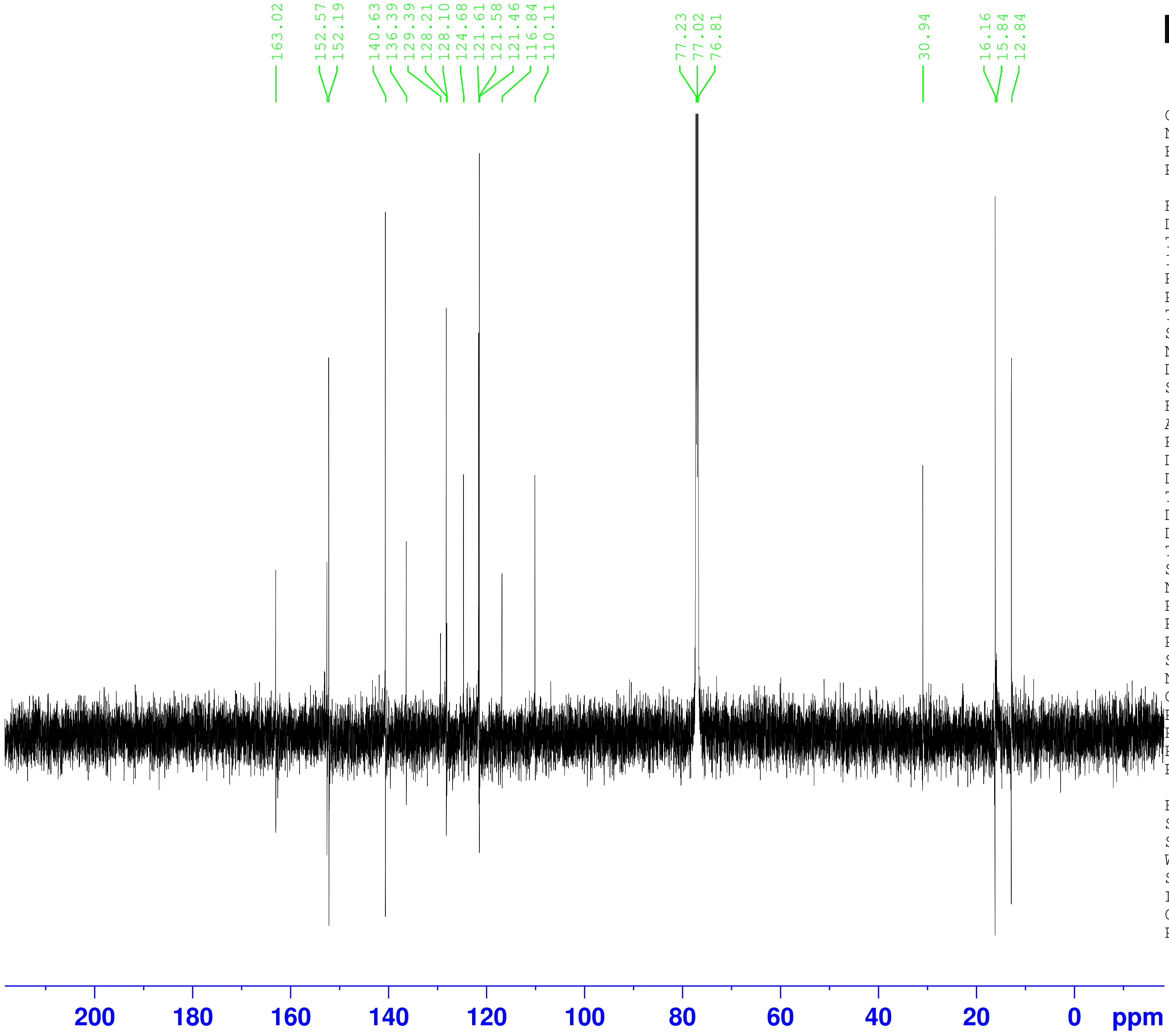

Current Data Parameters

NAME	MCC-I-1194-B-P1-13C
EXPNO	1
PROCNO	1

F2 - Acquisition Parameters

Date_	20241025
Time	13.44 h
INSTRUM	Avance 4 NEO 600
PROBHD	Z168773_0007 (
PULPROG	zgpg30
TD	65536
SOLVENT	CDCl3
NS	256
DS	4
SWH	35714.286 Hz
FIDRES	1.089913 Hz
AQ	0.9175040 sec
RG	101
DW	14.000 usec
DE	18.00 usec
TE	298.0 K
D1	2.00000000 sec
D11	0.03000000 sec
TD0	1
SFO1	150.9178988 MHz
NUC1	13C
P0	3.50 usec
P1	10.50 usec
PLW1	68.97799683 W
SFO2	600.1324005 MHz
NUC2	1H
CPDPRG[2	waltz65
PCPD2	70.00 usec
PLW2	18.62700081 W
PLW12	0.54741001 W
PLW13	0.27533999 W

F2 - Processing parameters

SI	32768
SF	150.9028085 MHz
WDW	EM
SSB	0
LB	1.00 Hz
GB	0
PC	1.40
