# Supplementary material for: Photocatalytic Cross‐Coupling of Phenols and Heteroaryl Halides With Machine Learning‐Guided Reaction Prediction
Source: Angew Chem Int Ed Engl. 2026 Mar 5;65(16):e8222003. doi: 10.1002/anie.8222003 (PMC13080434; doi:10.1002/anie.8222003)
Supplement: Supplementary file 2 — Supporting File 2: anie71621‐sup‐0002‐Data.zip. [file ANIE-65-e8222003-s001.zip › anie71621-sup-0002-Data/NMR FAIR Data 2.zip!/NMR FAIR Data/7zc'/13C NMR/MCC-I-1334-13C-600/2/pdata/1/email_MCC-I-1334-13C-600_2_1.pdf]

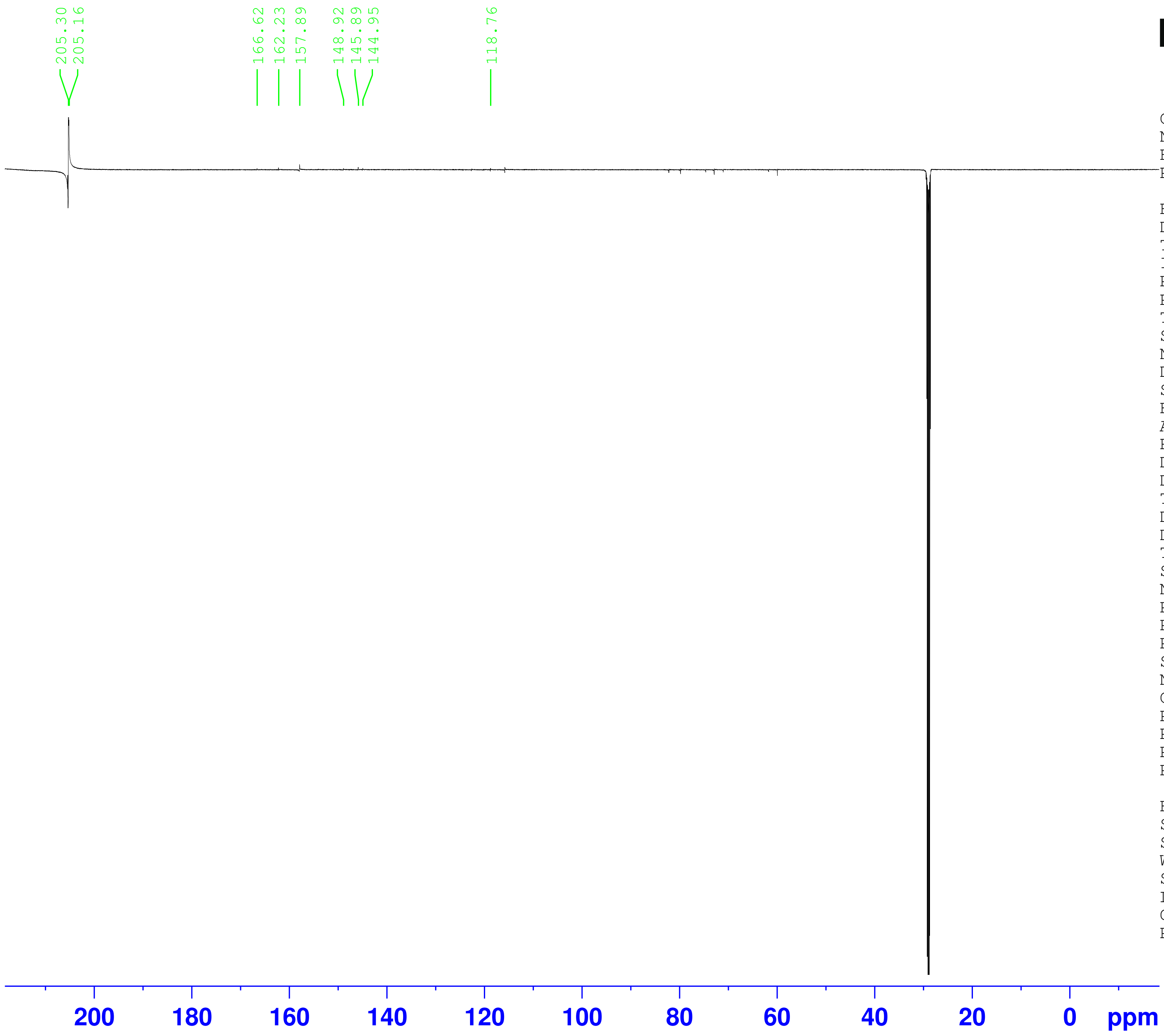

BRUKER

Current Data Parameters
NAME MCC-I-1334-13C-600
EXPNO 2
PROCNO 1

F2 - Acquisition Parameters

Date_	20250612	
Time	1.55	h
INSTRUM	Avance 4 NEO 600	
PROBHD	Z168773_0007 (	
PULPROG	zgpg30	
TD	65536	
SOLVENT	Acetone	
NS	1024	
DS	4	
SWH	35714.286	Hz
FIDRES	1.089913	Hz
AQ	0.9175040	sec
RG	101	
DW	14.000	usec
DE	18.00	usec
TE	298.0	K
D1	2.00000000	sec
D11	0.03000000	sec
TD0	1	
SFO1	150.9178988	MHz
NUC1	13C	
P0	3.50	usec
P1	10.50	usec
PLW1	68.97799683	W
SFO2	600.1324005	MHz
NUC2	1H	
CPDPRG[2	waltz65	
PCPD2	70.00	usec
PLW2	18.62700081	W
PLW12	0.54741001	W
PLW13	0.27533999	W

F2 - Processing parameters

SI	32768	
SF	150.9028085	MHz
WDW	EM	
SSB	0	
LB	1.00	Hz
GB	0	
PC	1.40	
