# Supplementary material for: Photocatalytic Cross‐Coupling of Phenols and Heteroaryl Halides With Machine Learning‐Guided Reaction Prediction
Source: Angew Chem Int Ed Engl. 2026 Mar 5;65(16):e8222003. doi: 10.1002/anie.8222003 (PMC13080434; doi:10.1002/anie.8222003)
Supplement: Supplementary file 2 — Supporting File 2: anie71621‐sup‐0002‐Data.zip. [file ANIE-65-e8222003-s001.zip › anie71621-sup-0002-Data/NMR FAIR Data 2.zip!/NMR FAIR Data/7a'/13C NMR/MCC-I-1306-C-13C-2/1/pdata/1/email_MCC-I-1306-C-13C-2_1_1.pdf]

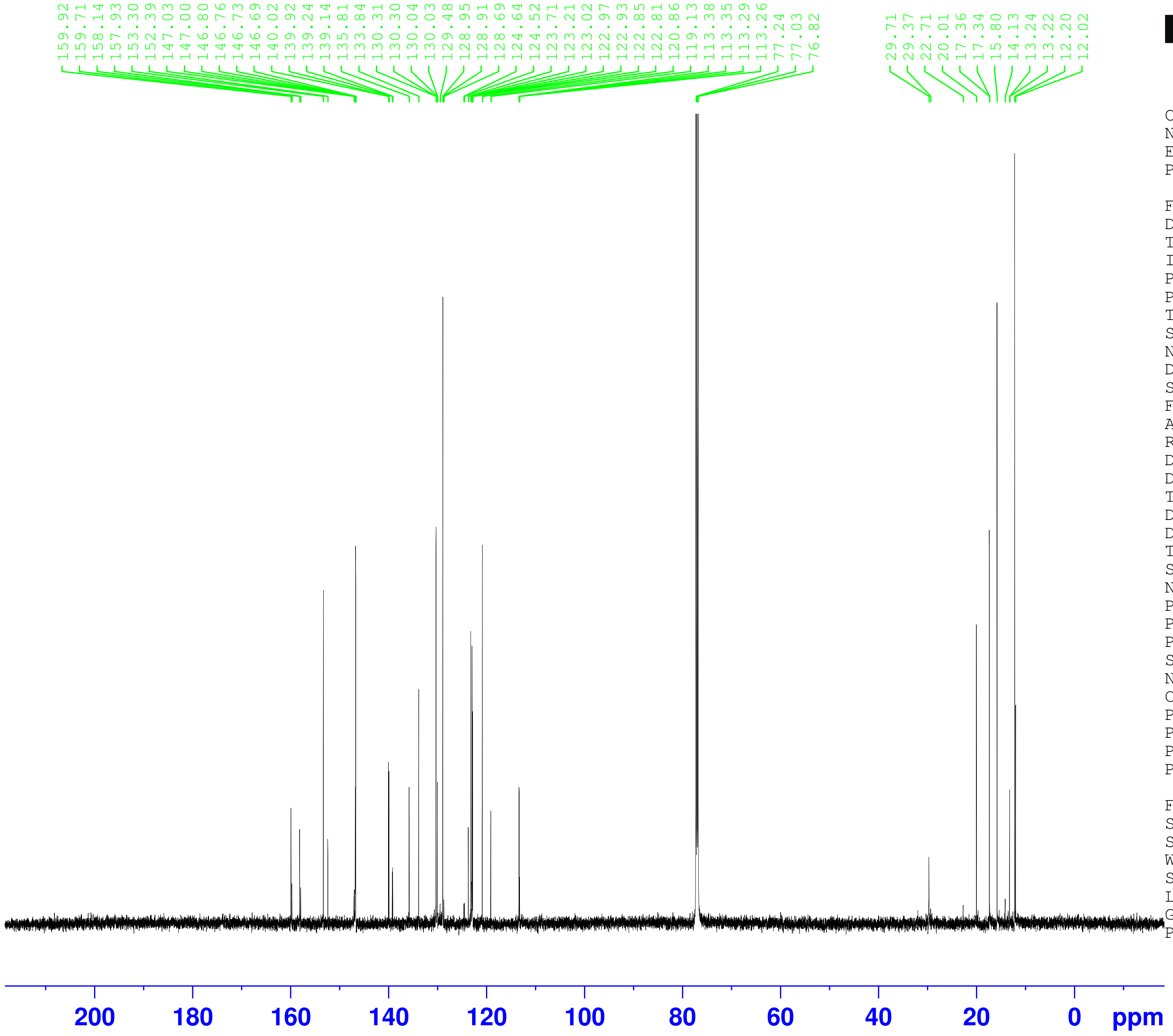


```
Current Data Parameters
NAME       MCC-I-1306-C-13C-2
EXPNO                       1
PROCNO                      1

F2 - Acquisition Parameters
Date_              20250513
Time                  11.47 h
INSTRUM    Avance 4 NEO 600
PROBHD     Z168773_0007 (
PULPROG              zgpg30
TD                    65536
SOLVENT               CDCl3
NS                      512
DS                        4
SWH               35714.286 Hz
FIDRES             1.089913 Hz
AQ                0.9175040 sec
RG                      101
DW                   14.000 usec
DE                    18.00 usec
TE                    298.0 K
D1               2.00000000 sec
D11              0.03000000 sec
TD0                       1
SFO1            150.9178988 MHz
NUC1                    13C
P0                     3.50 usec
P1                    10.50 usec
PLW1            68.97799683 W
SFO2            600.1324005 MHz
NUC2                     1H
CPDPRG[2            waltz65
PCPD2                 70.00 usec
PLW2            18.62700081 W
PLW12            0.54741001 W
PLW13            0.27533999 W

F2 - Processing parameters
SI                    32768
SF              150.9028085 MHz
WDW                      EM
SSB                       0
LB                     1.00 Hz
GB                        0
PC                     1.40
```
