# Supplementary material for: Photocatalytic Cross‐Coupling of Phenols and Heteroaryl Halides With Machine Learning‐Guided Reaction Prediction
Source: Angew Chem Int Ed Engl. 2026 Mar 5;65(16):e8222003. doi: 10.1002/anie.8222003 (PMC13080434; doi:10.1002/anie.8222003)
Supplement: Supplementary file 2 — Supporting File 2: anie71621‐sup‐0002‐Data.zip. [file ANIE-65-e8222003-s001.zip › anie71621-sup-0002-Data/NMR FAIR Data 2.zip!/NMR FAIR Data/7s/13C NMR/MCC-I-1363-2-13C-600/2/pdata/1/email_MCC-I-1363-2-13C-600_2_1.pdf]

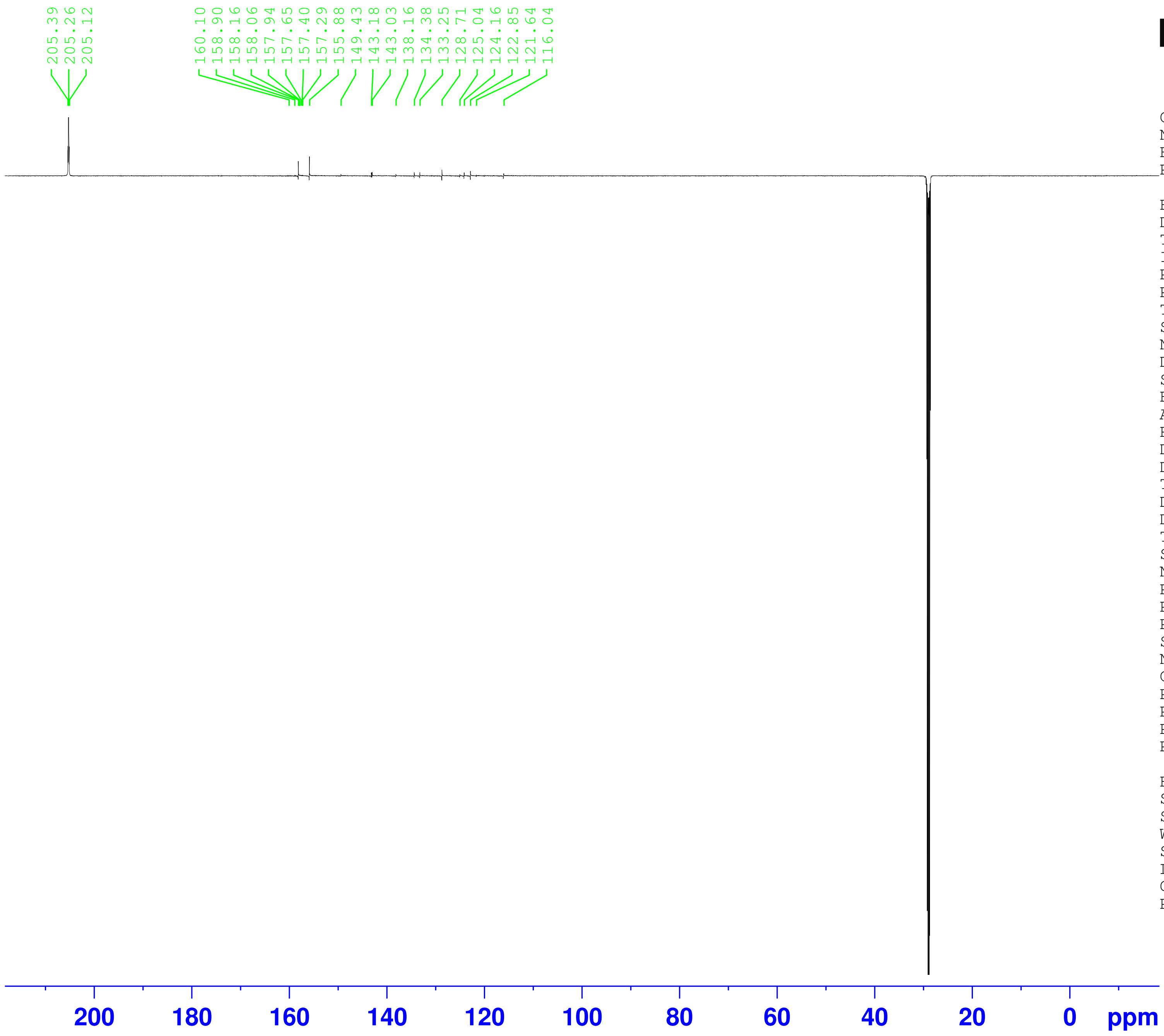

Current Data Parameters
NAME MCC-I-1363-2-13C-600
EXPNO 2
PROCNO 1

F2 - Acquisition Parameters
Date_ 20250809
Time 3.05 h
INSTRUM Avance 4 NEO 600
PROBHD Z168773_0007 (
PULPROG zgpg30
TD 65536
SOLVENT Acetone
NS 5000
DS 4
SWH 35714.286 Hz
FIDRES 1.089913 Hz
AQ 0.9175040 sec
RG 101
DW 14.000 usec
DE 18.00 usec
TE 298.0 K
D1 2.00000000 sec
D11 0.03000000 sec
TD0 1
SFO1 150.9178988 MHz
NUC1 13C
P0 3.50 usec
P1 10.50 usec
PLW1 68.97799683 W
SFO2 600.1324005 MHz
NUC2 1H
CPDPRG[2 waltz65
PCPD2 70.00 usec
PLW2 18.62700081 W
PLW12 0.54741001 W
PLW13 0.27533999 W

F2 - Processing parameters
SI 32768
SF 150.9028085 MHz
WDW EM
SSB 0
LB 1.00 Hz
GB 0
PC 1.40
