# Supplementary material for: Photocatalytic Cross‐Coupling of Phenols and Heteroaryl Halides With Machine Learning‐Guided Reaction Prediction
Source: Angew Chem Int Ed Engl. 2026 Mar 5;65(16):e8222003. doi: 10.1002/anie.8222003 (PMC13080434; doi:10.1002/anie.8222003)
Supplement: Supplementary file 2 — Supporting File 2: anie71621‐sup‐0002‐Data.zip. [file ANIE-65-e8222003-s001.zip › anie71621-sup-0002-Data/NMR FAIR Data 2.zip!/NMR FAIR Data/7t/13C NMR/MCC-I-1360-13C-600/2/pdata/1/email_MCC-I-1360-13C-600_2_1.pdf]

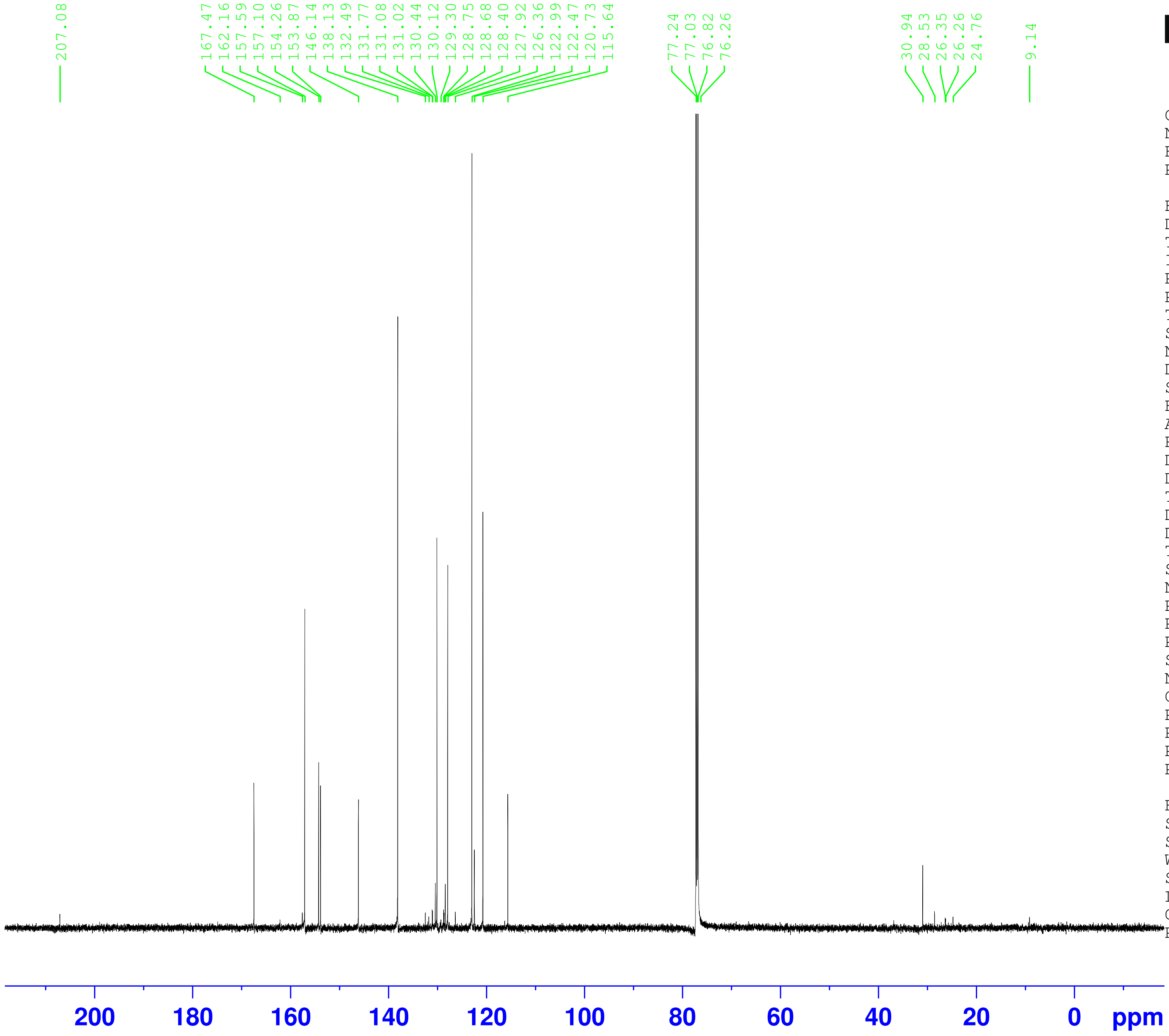


BRUKER

Current Data Parameters
NAME MCC-I-1360-13C-600
EXPNO 2
PROCNO 1

F2 - Acquisition Parameters

| | | |
|---|---|---|
| Date_ | 20250806 | |
| Time | 13.47 | h |
| INSTRUM | Avance 4 NEO 600 | |
| PROBHD | Z168773_0007 ( | |
| PULPROG | zgpg30 | |
| TD | 65536 | |
| SOLVENT | CDCl3 | |
| NS | 512 | |
| DS | 4 | |
| SWH | 35714.286 | Hz |
| FIDRES | 1.089913 | Hz |
| AQ | 0.9175040 | sec |
| RG | 101 | |
| DW | 14.000 | usec |
| DE | 18.00 | usec |
| TE | 298.0 | K |
| D1 | 2.00000000 | sec |
| D11 | 0.03000000 | sec |
| TD0 | 1 | |
| SFO1 | 150.9178988 | MHz |
| NUC1 | 13C | |
| P0 | 3.50 | usec |
| P1 | 10.50 | usec |
| PLW1 | 68.97799683 | W |
| SFO2 | 600.1324005 | MHz |
| NUC2 | 1H | |
| CPDPRG[2 | waltz65 | |
| PCPD2 | 70.00 | usec |
| PLW2 | 18.62700081 | W |
| PLW12 | 0.54741001 | W |
| PLW13 | 0.27533999 | W |

F2 - Processing parameters

| | | |
|---|---|---|
| SI | 32768 | |
| SF | 150.9028085 | MHz |
| WDW | EM | |
| SSB | 0 | |
| LB | 1.00 | Hz |
| GB | 0 | |
| PC | 1.40 | |
